# eCyclopropanation – a safe and scalable electrochemical route to cyclopropanes

**DOI:** 10.1039/d5sc08940a

**Published:** 2026-02-11

**Authors:** Jamie M. Walsh, Marco Galzignato, Shusuke Hattori, Marylise Triacca, Kevin Lam

**Affiliations:** a School of Science, Faculty of Engineering and Science, University of Greenwich Chatham Maritime Chatham Kent ME4 4TB UK k.lam@greenwich.ac.uk

## Abstract

Diazo compounds are among the most versatile intermediates in organic synthesis, enabling high-value transformations such as cyclopropanation, X–H insertion, and heterocycle formation. However, their intrinsic instability and hazardous nature have severely restricted their practical use, particularly at scale, largely due to the need to generate, isolate, or accumulate diazo species in solution. Here, we report a safe, one-pot, scalable, and operationally simple electrochemical strategy for the *in situ* generation of diazo compounds from *tert*-butylhydrazones, directly coupled to Rh(ii)-catalysed cyclopropanation. In contrast to established approaches, the diazo intermediates in this platform are generated and consumed continuously, with no detectable accumulation at any stage of the process. This transient mode of operation fundamentally alters the safety profile of diazo chemistry, enabling electricity-driven oxidation under mild conditions without hazardous oxidants, isolated diazo compounds, or highly toxic additives. The use of a bench-stable mono-protected hydrazone precursor and a weakly nucleophilic electrolyte ensures efficient carbene transfer, delivering cyclopropanes in high yields across a broad range of hydrazones and olefins. The method readily translates to continuous flow, enabling gram-scale synthesis with good productivity. By preventing the accumulation of free diazo species in solution, this work removes a key barrier to the safe and scalable use of diazo chemistry and provides a general blueprint for modern carbene-transfer processes that are inherently safer, more sustainable, and industrially relevant.

## Abstract 2

Electrochemical generation of diazo compounds from *tert*-butyl hydrazones enables synchronised Rh-catalysed carbene transfer without diazo accumulation. This inventory-free strategy improves safety and allows gram-scale cyclopropanation in batch and flow.

## Graphical abstract

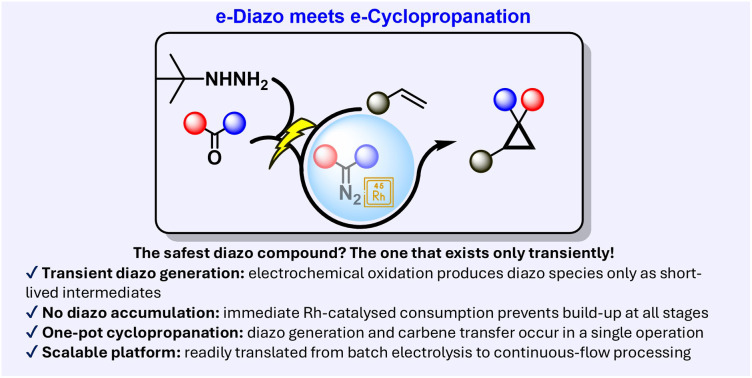

## Introduction

**Fig. 1:**
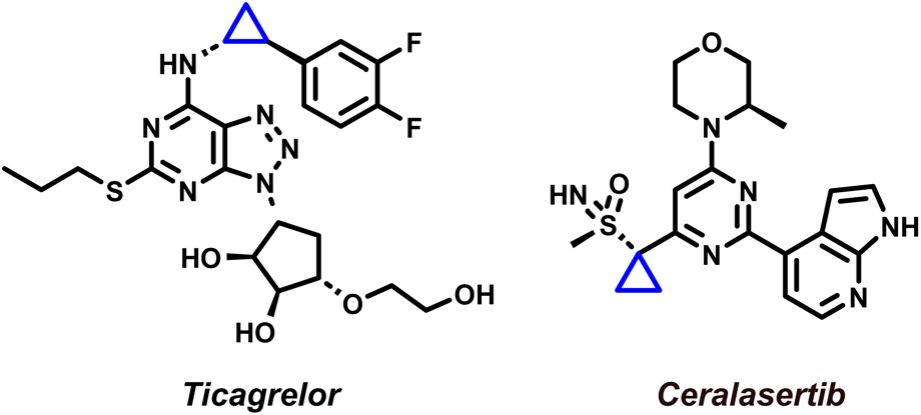
Marketed drugs containing cyclopropane moiety.

**Scheme 1:**
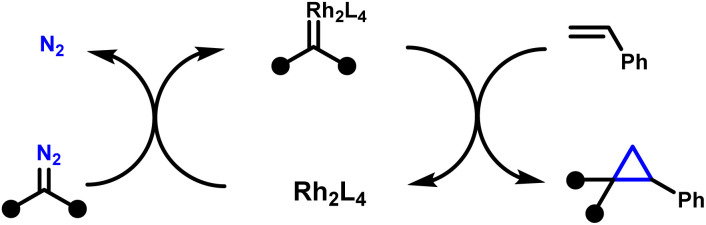
General mechanism of rhodium(ii) catalysed cyclopropanation.^15^

Cyclopropanes are privileged motifs in synthetic chemistry and drug discovery, where their high strain, conformational control, and metabolic stability can translate into improved potency and physicochemical profiles (Fig. 1).^1–11^ Among the available approaches to access these scaffolds, transition-metal-catalysed cyclopropanation of alkenes using diazo-derived carbenes remains the benchmark method, combining broad substrate scope with high functional-group tolerance and excellent stereocontrol.^12–20^ In this context, dirhodium(ii) paddlewheel complexes, notably Rh_2_(OAc)_4_ and chiral congeners, have become the workhorse catalysts for diastereo- and enantioselective cyclopropanation across a wide range of olefins.^15^ Mechanistically, these catalysts mediate controlled diazo decomposition to generate Rh-carbenoid intermediates, which typically engage alkenes through a concerted [2 + 1] cycloaddition to deliver cyclopropanes with high levels of selectivity (Scheme 1).^21–23^

**Scheme 2:**
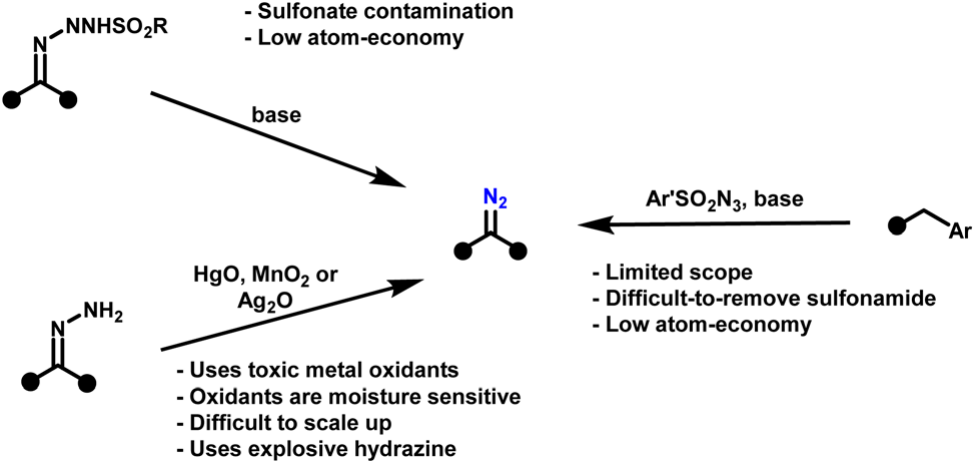
Overview of the traditional methods for the synthesis of diazo compounds.

The broader application of diazo-based cyclopropanation, especially at scale, has been hampered by the inherent dangers of diazo chemistry. Diazo compounds are extraordinarily reactive intermediates,^24–27^ enabling transformations such as cyclopropanation,^28,29^ 1,3-dipolar cycloadditions,^30^ and X–H insertions.^31^ Yet this same reactivity, while a synthetic strength, has also been their greatest liability. Their instability and propensity for energetic decomposition pose serious safety risks, particularly in scale-up. Many diazo compounds are thermally labile, light- and shock-sensitive, and can undergo violent exothermic decomposition with rapid nitrogen gas release.^32^ Consequently, established synthetic approaches to diazo compounds remain problematic (Scheme 2). Common methods such as the Regitz diazo transfer,^33^ Bamford–Stevens reaction,^34^ and hydrazone oxidation,^35–37^ typically rely on hazardous reagents, show poor atom economy, and generate substantial waste. In addition, these reactions often afford crude mixtures contaminated with sulfonyl- or hydrazine-derived by-products, complicating handling and purification. Importantly, sulfonyl azides used in Regitz-type diazo transfer reactions can be as explosive as, or even more hazardous than, the diazo compounds they are intended to produce.^32^ This undermines the perception that such precursors provide a safer alternative and highlights a fundamental paradox: many strategies developed to avoid diazo hazards simply displace, rather than eliminate, the underlying risk.

Meanwhile, oxidative hydrazone-to-diazo conversion, while attractive in principle due to its atom economy, has traditionally relied on stoichiometric amounts of costly and hazardous oxidants, including Ag_2_O,^38^ HgO,^39^ MnO_2_,^40^ and Pb(OAc)_4_.^41^ Although greener variants employing air as the terminal oxidant have been reported,^42^ these approaches still depend on copper catalysis and introduce significant scale-up challenges, notably the risk of flammable solvent–oxygen mixtures when oxygen concentrations approach or exceed the solvent flashpoint. Even the formation of hydrazones themselves requires hydrazine, a volatile, carcinogenic, and explosive reagent, which is subject to strict regulatory control. Moreover, hydrazine may condense at both nitrogen atoms, yielding undesired bis-hydrazones/azines that compete with productive diazo formation. Together, these issues form a critical barrier to progress: diazo chemistry remains powerful but impractical, particularly for large-scale or continuous applications.

**Scheme 3:**
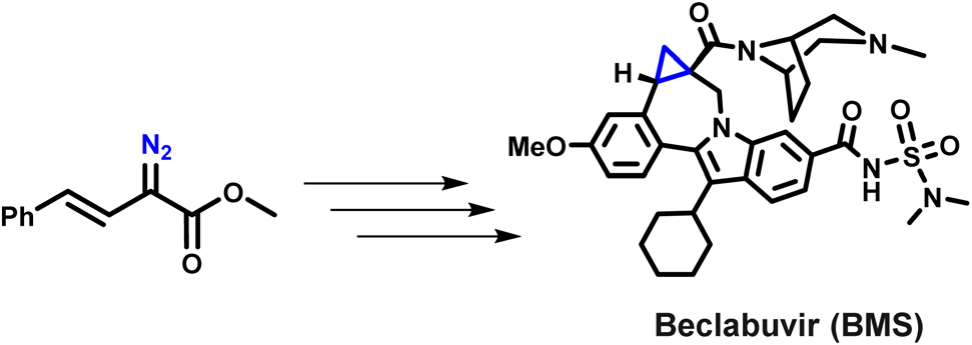
BMS beclabuvir case study: scale-up evaluation of diazo cyclopropanation.

Bristol-Myers Squibb's reported synthesis of the HCV therapeutic beclabuvir represents a rare and instructive case study in the industrial consideration of diazo chemistry. A chiral Rh(ii)-catalysed enantioselective cyclopropanation of a styryl diazo ester was developed and evaluated at scale through extensive safety engineering, strict containment measures, and rigorous process optimisation (Scheme 3).^43^ However, despite demonstrating technical feasibility, this approach was ultimately not implemented in routine manufacturing, underscoring the substantial practical and safety challenges associated with large-scale diazo handling. This example highlights that while industrial diazo chemistry can, in principle, be rendered viable, it currently requires exceptional resources and risk-mitigation strategies. As a result, there is a clear need for new methodologies that are not only greener and more sustainable but also intrinsically safer, specifically approaches that avoid both hazardous reagents and the accumulation of free diazo intermediates.

**Scheme 4:**
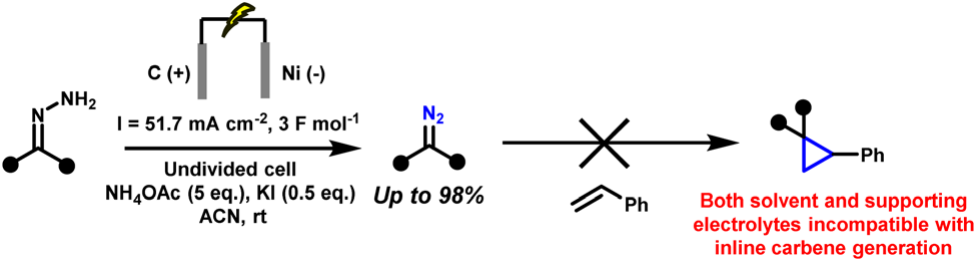
Previous electrochemical conditions for diazo synthesis, incompatible with downstream cyclopropanation.^56^

Electrochemical strategies have recently emerged as a promising alternative for the generation of diazo compounds^44,45^ and cyclopropanes.^46,47^ By replacing stoichiometric oxidants with electricity, anodic oxidation of hydrazones offers a direct, atom-economical route under mild, oxidant-free conditions.^48–50^ This not only reduces chemical waste but also allows fine control over redox potentials and reaction parameters, improving both selectivity and safety.^51–54^ Initial studies demonstrated the feasibility of generating diazo species electrochemically, but these reports were typically limited to one or two model substrates and often required isolation of the diazo intermediates before further use.^55–57^ More recently, our group, in collaboration with the Ollevier laboratory, reported a greener electrochemical method for generating stabilised diazo from simple hydrazones.^44^ The method proceeds under ambient conditions, tolerates air and moisture, and accommodates a range of substrates. However, even this approach remained incompatible with *in situ* transformations. The presence of additives such as potassium iodide and ammonium acetate, and the use of acetonitrile as solvent, led to side reactions that precluded direct downstream reactivity. In particular, these conditions were incompatible with carbene transfer, meaning that the diazo compounds, though generated in a greener way, still had to be isolated and purified. Thus, the intrinsic safety issues of diazo chemistry remained unresolved (Scheme 4).

To address the safety and scalability challenges associated with diazo chemistry, we set out to develop an integrated electrochemical–catalytic platform that enables the *in situ* generation and immediate consumption of diazo compounds under mild conditions. Drawing inspiration from earlier advances in electrochemical hydrazone oxidation, we identified *tert*-butylhydrazine as a practical and bench-stable precursor that could deliver diazo intermediates upon anodic oxidation. By coupling this transformation with Rh(ii)-catalysed carbene transfer, we envisioned a system in which transient diazo species would be intercepted as they form, enabling efficient and selective cyclopropanation of alkenes without the need for pre-isolated or excess diazo reagents. A key design feature of this approach is the ability to control diazo concentration electrochemically, matching its rate of generation to catalytic turnover and thereby suppressing accumulation. Achieving this required a fundamental re-evaluation of the electrochemical conditions to ensure compatibility with reactive carbenoid intermediates. Here, we report a safe, scalable, and operationally simple electrochemical protocol for Rh(ii)-catalysed cyclopropanation, *via* real-time diazo generation from mono-protected hydrazones, enabling direct carbene transfer in batch and flow.

## Results and discussion

We began our optimisation investigations by adapting our previously reported electrochemical diazo synthesis conditions,^44^ which were not compatible with Rh(ii)-catalysed carbene transfer. Our objective was to establish a single, integrated protocol that enables efficient and selective cyclopropanation directly from hydrazones under electrochemical conditions. To achieve this, we focused on four key criteria: (i) generating the diazo compound in high yield, (ii) ensuring that the electrochemical conditions do not inhibit downstream carbene formation, (iii) controlling the rate of diazo generation so that it is immediately consumed and does not accumulate, and (iv) maintaining operational simplicity and scalability. Particular attention was given to replacing hydrazine-derived hydrazones, which posed safety and selectivity issues, and eliminating ammonium acetate, which releases acetic acid and acts as a carbene trap. We systematically evaluated electrochemical parameters, including the supporting electrolyte, electrode material, solvent, and current density, with the goal of identifying conditions that generate the diazo intermediate using minimal additives while enabling productive Rh(ii)-catalysed cyclopropanation across a broad substrate scope. Full optimisation data, including control experiments and condition screens, are provided in the SI; only the most salient results are discussed here.

First, we decoupled the transformation by optimising the generation of stabilised diazo under conditions compatible with the generation of carbenes.

Several critical insights emerged from the optimisation studies, foremost among them the choice of hydrazone precursor. Conventional hydrazones derived from hydrazine hydrate present two fundamental limitations: their synthesis is frequently complicated by bis-hydrazone formation due to condensation at both nucleophilic NH_2_ sites, and hydrazine itself is highly toxic, carcinogenic, and potentially explosive, rendering it unsuitable for industrial use. To overcome these issues, a series of mono-protected hydrazones derived from *tert*-butyl hydrazine hydrochloride, *tert*-butoxycarbonyl hydrazine (BocNHNH_2_), tosyl hydrazine (TsNHNH_2_), and benzyl hydrazine (BnNHNH_2_) were evaluated under the previously optimised electrochemical conditions in acetonitrile containing ammonium acetate and potassium iodide. Among these candidates, *tert-*butyl hydrazones proved optimal, delivering the corresponding diazo compounds cleanly and in good yield (Table 1, entry 5). In addition to their favourable reactivity, *tert-*butyl hydrazones exhibited excellent bench stability, likely arising from steric shielding imparted by the bulky *tert-butyl* substituent. Importantly, safety considerations further reinforced this choice: differential scanning calorimetry (DSC) analysis revealed that *tert*-butyl hydrazones possess significantly higher thermal stability than tosyl hydrazones, a classic diazo precursor, and display a markedly attenuated exothermic profile and no abrupt decomposition events under identical conditions (see SI).^32^

**Table 1:** Optimisation of starting material for previous anodic oxidation conditions.^56^ Reactions were carried out on a 0.4 mmol scale at room temperature (rt) in a 5 mL ElectraSyn cell equipped with carbon and nickel electrodes, and yields were determined by ^1^H NMR using CH_2_Br_2_ as an internal standard

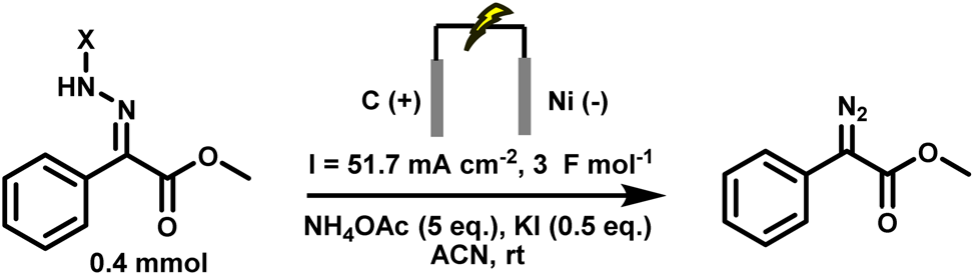		
Entry	Starting material	Yield
1	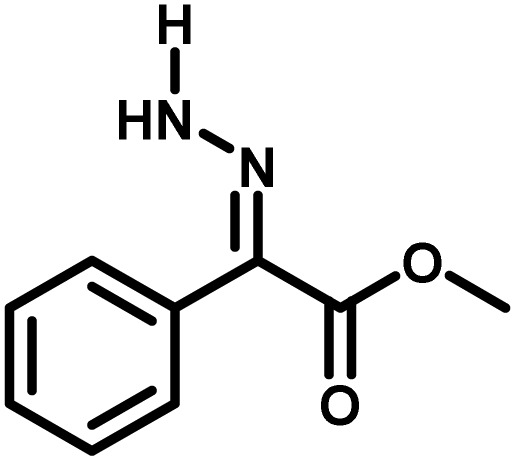	98%
2	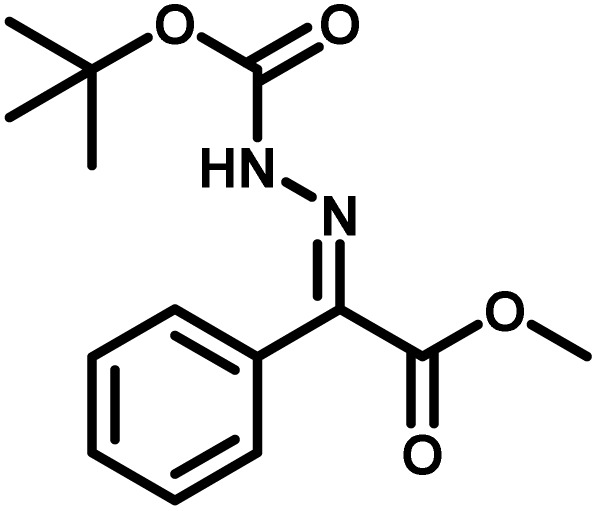	0%
3	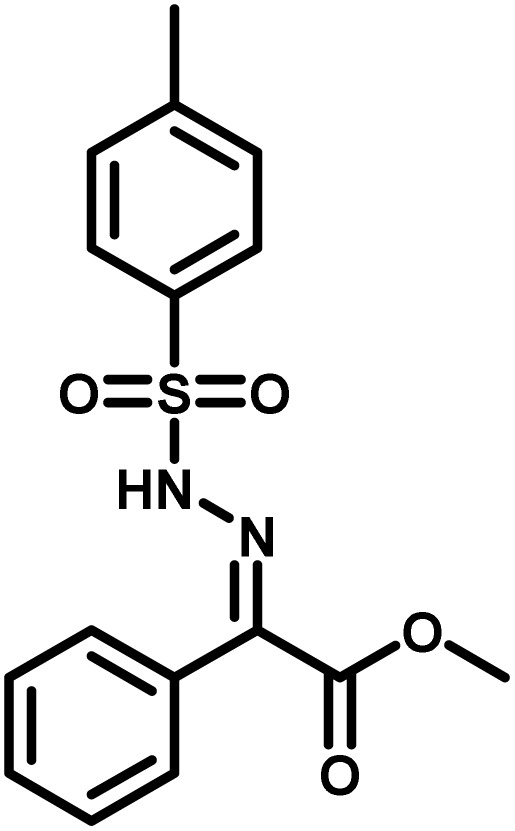	0%
4	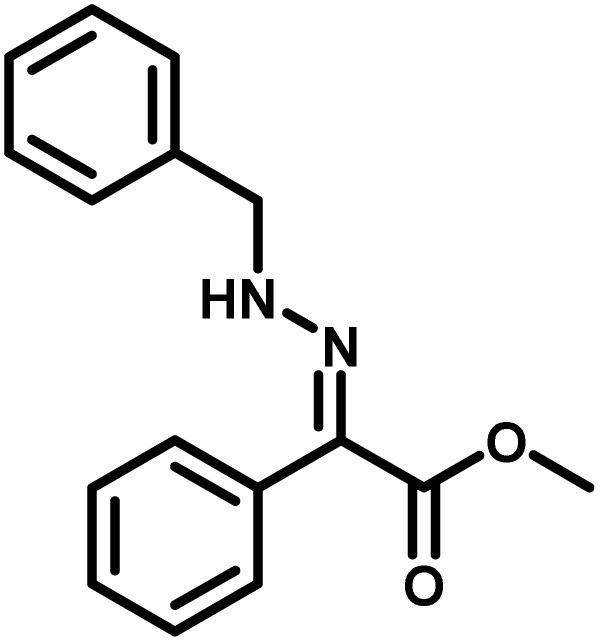	31%
5	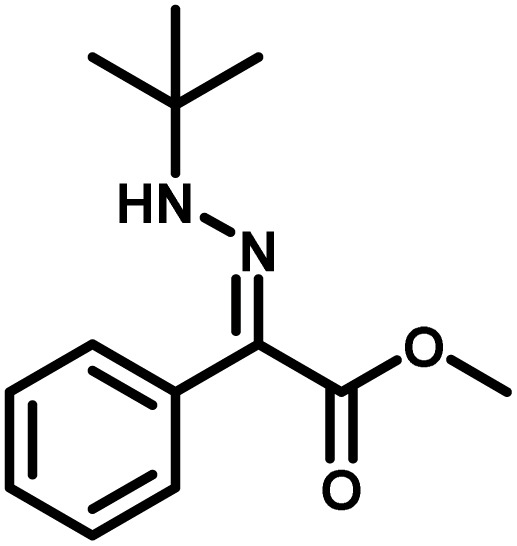	**94%**

Concurrently, the supporting electrolyte was optimised to avoid nucleophilic counterions that could intercept the carbene. Collidinium tetrafluoroborate (Coll·HBF_4_) emerged as the optimal choice: unlike ammonium acetate, it does not release any carboxylic acid *in situ*, thereby eliminating a major O–H insertion pathway for the carbene intermediate. In addition, it supplies protons for cathodic H_2_ evolution, maintaining charge balance without engaging in deleterious side reactions. Electrode material selection also played a key role. Graphite was retained as the anode due to its low cost, wide electrochemical window, and oxidative stability. Stainless steel was selected as the cathode in place of previously used nickel, offering a lower hydrogen overpotential for efficient H_2_ evolution while aligning with standard materials used in industrial flow reactors. The solvent dichloromethane (DCM) was chosen based on precedent demonstrating its inertness toward carbenes, unlike acetonitrile or methanol, which are known to quench carbene intermediates.^58^ Applying a low current density of 5.2 mA cm^−2^ and a total charge of 1.5 F mol^−1^ was critical to synchronise diazo generation with downstream carbene and cyclopropane formation, thereby preventing diazo accumulation in solution. Higher current densities led to potentially hazardous accumulation of diazo intermediates, as detected by HPLC-MS, and triggered side reactions: deactivation of the Rh catalyst, overoxidation of the hydrazone at the anode, and undesired reduction of the diazo species at the cathode. Under the optimised conditions, *tert*-butyl hydrazone (0.4 mmol), graphite anode, stainless steel cathode, Coll·HBF_4_ (2 eq.), DCM (5 mL), current density of 5.2 mA cm^−2^, and 1.5 F mol^−1^ charge, the diazo compound 4.1 was obtained in 89% yield, providing a safer and more practical platform for downstream carbene transformations (Table 2, entry 16).

**Table 2:** Optimisation of anodic oxidation of hydrazone 2.1. Reactions were carried out on a 0.4 mmol scale at room temperature (rt) in a 5 mL ElectraSyn cell, and yields were determined by ^1^H NMR using CH_2_Br_2_ as an internal standard

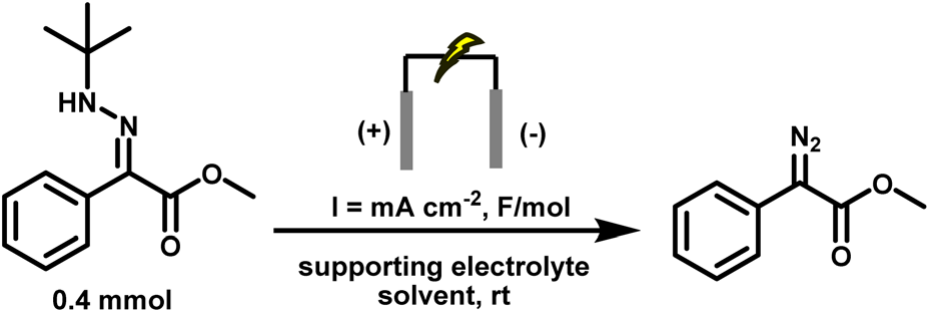					
Entry	(+)/(−)	Supporting electrolytes	Solvent	*I*	Yield
1	C (+)	Collidine (1 eq.)	ACN	51.7 mA cm^−2^	51%
	Ni (−)	AcOH (1 eq.)		3 F mol^−1^	
		KI (0.5 eq.)			
2	C (+)	Collidine (1 eq.)	ACN	51.7 mA cm^−2^	31%
	Ni (−)	Coll·HBF_4_ (1 eq.)		3 F mol^−1^	
		KI (0.5 eq.)			
3	C (+)	Et_4_NI (2 eq.)	DCM	10.3 mA cm^−2^	73%
	Ni (−)	Coll·AcOH (1 eq.)		5 F mol^−1^	
4	C (+)	Et_4_NI (0.5 eq.)	DCM	10.3 mA cm^−2^	71%
	Ni (−)	Coll·AcOH (1 eq.)		5 F mol^−1^	
5	C (+)	TBAI (0.5 eq.)	DCM	20.7 mA cm^−2^	82%
	Ni (−)	Coll·AcOH (2 eq.)		3 F mol^−1^	
6	C (+)	TBAI (0.5 eq.)	DCM	20.7 mA cm^−2^	88%
	SS (−)	Coll·AcOH (2 eq.)		3 F mol^−1^	
7	C (+)	TBAI (0.5 eq.)	DCM	20.7 mA cm^−2^	84%
	SS (−)	Coll·AcOH (3 eq.)		3 F mol^−1^	
8	C (+)	TBAI (0.5 eq.)	DCM	31 mA cm^−2^	62%
	SS (−)	Coll·AcOH (2 eq.)		3 F mol^−1^	
9	C (+)	TBAI (0.5 eq.)	DCM	20.7 mA cm^−2^	71%
	SS (−)	Coll·TFA (2 eq.)		4 F mol^−1^	
10	C (+)	Coll·TFA (3 eq.)	DCM	10.3 mA cm^−2^	0%
	SS (−)			4 F mol^−1^	
11	C (+)	TBAI (0.5 eq.)	DCM	20.7 mA cm^−2^	79%
	SS (−)	Coll·TfOH (2 eq.)		4 F mol^−1^	
12	C (+)	Coll·TfOH (2 eq.)	DCM	20.7 mA cm^−2^	0%
	SS (−)			4 F mol^−1^	
13	C (+)	Coll·HBF_4_ (3 eq.)	DCM	10.3 mA cm^−2^	64%
	SS (−)			1.5 F mol^−1^	
14	C (+)	Coll·HBF_4_ (3 eq.)	DCM	20.7 mA cm^−2^	33%
	SS (−)			2 F mol^−1^	
15	C (+)	Coll·HBF_4_ (3 eq.)	DCM	5.2 mA cm^−2^	78%
	SS (−)			1.5 F mol^−1^	
**16**	**C (+)**	**Coll·HBF**_**4**_ **(2 eq.)**	**DCM**	**5.2 mA cm^−^** ^**2**^	**89%**
	**SS** (−)			**1.5 F mol^−^** ^**1**^	
17	C (+)	Coll·HBF_4_ (2 eq.)	DCM	2.1 mA cm^−2^	71%
	SS (−)			1.5 F mol^−1^	
18	C (+)	Coll·HBF_4_ (1.5 eq.)	DCM	5.2 mA cm^−2^	56%
	SS (−)			1.5 F mol^−1^	

**Fig. 2:**
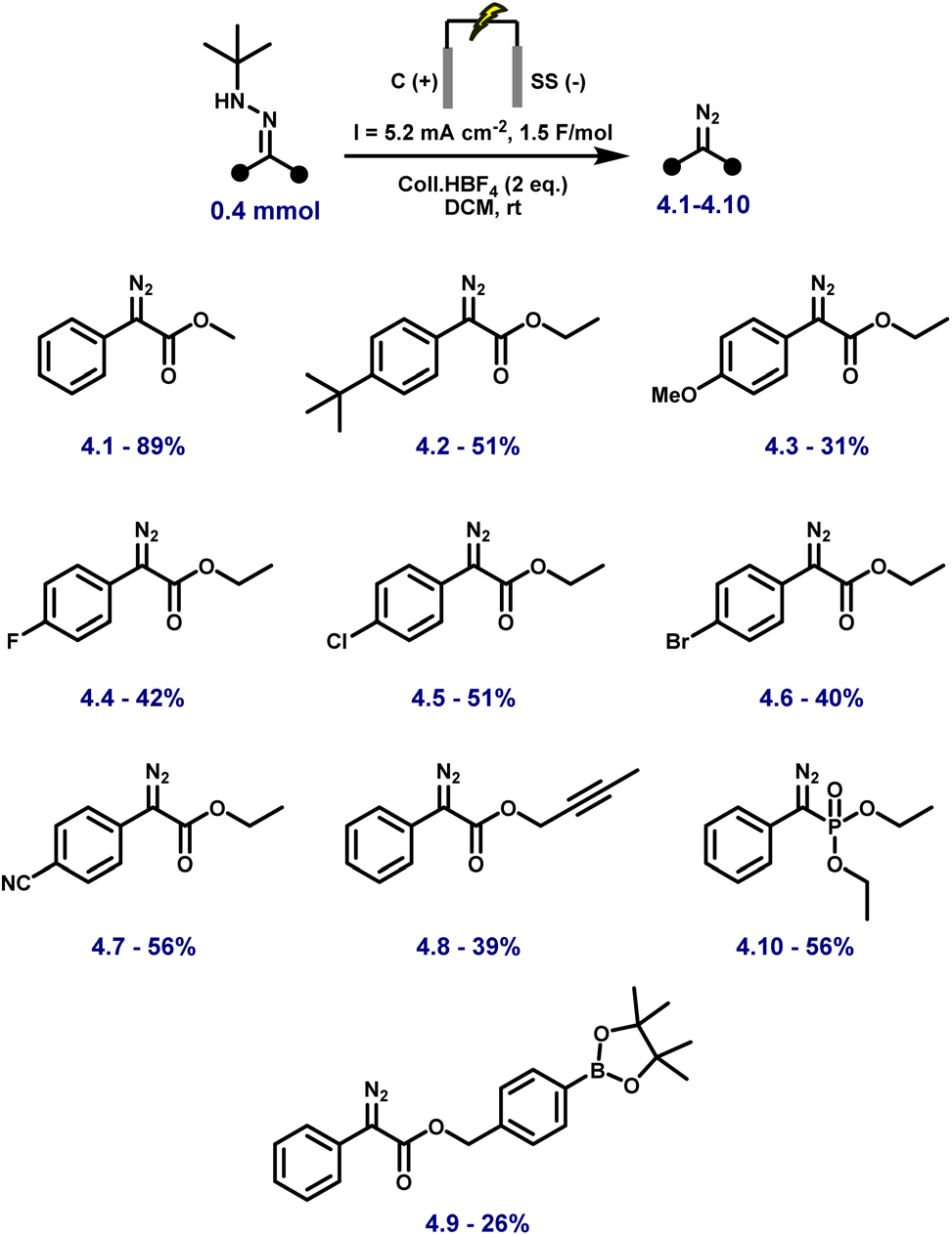
Substrate scope for the electrosynthesis of diazo compounds using optimised conditions. Reactions were carried out on a 0.4 mmol scale at rt in a 5 mL ElectraSyn cell equipped with C and SS electrodes, and yields are reported for pure, isolated products.

With the optimal conditions in hand, we started to explore the scope and limitations of our novel anodic synthesis of (semi)stabilised diazo compounds (Fig. 2). A diverse range of aryl–ester diazo compounds were obtained in moderate yields with excellent functional group tolerance (Fig. 2). Both electron-donating (4.2–4.3) and electron-withdrawing (4.4–4.7) substituents on the aromatic ring were well tolerated. Variation of the ester moiety also proved unproblematic, with successful diazo formation observed from a propargylic ester (4.8) and a sterically hindered benzylic ester bearing a boronic ester functionality (4.9). Notably, the synthesis of a diazo phosphonate (4.10, 56% yield) demonstrates the method's ability to access structurally diverse and synthetically valuable diazo compounds, including motifs that are challenging to obtain *via* conventional protocols. While the isolated yields are slightly lower than those obtained using our previously reported KI-mediated electrochemical method (53–99%),^44^ the present protocol employs a solvent (DCM) and supporting electrolyte (Coll·HBF_4_) that are directly compatible with downstream Rh-catalysed cyclopropanation. This eliminates the need for solvent switching or additional purification steps, streamlining tandem diazo generation–carbene transfer sequences.

Having confirmed that diazo compounds could be generated in good yields under milder electrochemical conditions compatible with downstream carbene formation, we next turned to the second stage of the transformation: integrating Rh(ii)-catalysed carbene transfer to achieve inline cyclopropanation from transient, non-accumulating diazo intermediates.

Much to our delight, initial experiments using Rh_2_(OAc)_4_ (1 mol%) and styrene (10 eq.) under our previously optimised electrochemical conditions delivered the desired cyclopropane product, confirming that diazo formation and carbene transfer could indeed be merged in a single operation (Table 3, entry 1). However, the yield was moderate (43%), and residual starting hydrazone indicated incomplete conversion under these conditions. Yield improved markedly upon lowering the current density. Excellent yields (up to 97%) were obtained at both 1.0 and 3.1 mA cm^−2^; however, 3.1 mA cm^−2^ was selected as the standard condition because it delivers the same efficiency with a shorter electrolysis time (Table 3, entries 3 and 5). This time-controlled approach proved crucial: it minimised side reactions by ensuring that diazo/carbene formation and trapping occurred concurrently. Supporting electrolyte loading was reduced to 0.5 eq., maintaining conductivity while further suppressing side reactions. Catalyst loading of 1 mol% was optimal, as lower concentrations led to diminished efficiency.

**Table 3:** Optimisation of conditions for the electrosynthesis of cyclopropane 3.1. Reactions were carried out on a 0.4 mmol scale at room temperature (rt) in a 5 mL ElectraSyn cell equipped with carbon and stainless steel electrodes, and yields were determined by ^1^H NMR using CH_2_Br_2_ as an internal standard

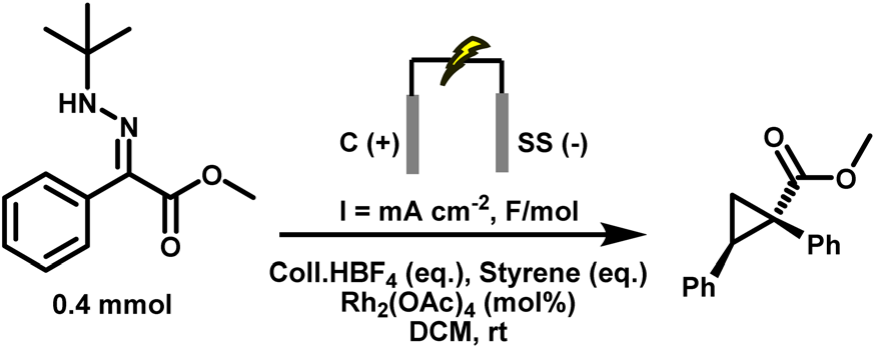						
Entry	Coll·HBF_4_ (eq.)	Styrene (eq.)	Rh_2_(OAc)_4_ (mol%)	Current (mA cm^−2^)	Charge (F mol^−1^)	Yield
1	2	10	1	5.2	1.5	43%
2	2	10	1	1	2.5	61%
3	0.5	10	1	1	2.2	97%
4	0.5	10	1	2.1	2	95%
**5**	**0.5**	**10**	**1**	**3.1**	**2**	**97%**
6	0.5	10	1	5.2	2	90%
7	1	10	1	7.2	2.3	83%
8	0.5	10	0.5	3.1	2.2	41%
9	1	10	1	10.3	2.3	78%
10	2.5	10	1	20.7	8	55%
11	0.5	10	N/A	3.1	2	0%
12	0.5	10	1	N/A	N/A	0%
13	N/A	10	1	3.1	2	17%

Under these optimised conditions, *tert*-butyl hydrazone (0.4 mmol), graphite anode, stainless steel cathode, Coll·HBF_4_ (0.5 equiv.), DCM solvent, 1 mol% Rh_2_(OAc)_4_, styrene (10 eq.), a current density of 3.1 mA cm^−2^, and a total charge of 2 F mol^−1^, the target cyclopropane 3.1 was obtained in 97% isolated yield, which matched the ^1^H NMR yield. Importantly, no accumulation of diazo intermediates was observed at any point during the electrolysis, as confirmed by in-line HPLC-MS and UV (PDA) monitoring (see SI for details). Under current control, diazo generation and Rh-catalysed consumption remain strictly synchronised. This result represents a significant advance, demonstrating that diazo compounds can be generated and consumed in real time under synthetically useful conditions, bypassing the need for isolation or storage.

A series of control experiments confirmed the essential role of each component and the electrochemical nature of the transformation (Table 3, entries 11–13). No product formation was observed in the absence of either applied current or Rh_2_(OAc)_4_. Exclusion of Coll·HBF_4_ led to a sharp drop in yield, with substantial recovery of unreacted hydrazone and minimal diazo formation, alongside the appearance of a reduced diazo compound (indicative of N_2_ loss). This outcome highlights the dual role of Coll·HBF_4_: it maintains ionic conductivity and supplies protons to enable cathodic hydrogen evolution, thereby suppressing undesired reduction of the diazo intermediate. In its absence, increased cell resistance, voltage buildup, and impaired current flow collectively diminished overall reaction efficiency.

Mechanistic studies support a stepwise anodic oxidation pathway leading to diazo formation. Cyclic voltammetry (CV) of the *tert*-butyl hydrazone reveals a two-electron oxidation event, consistent with sequential single-electron transfers. It is proposed that the first oxidation generates a radical cation, which undergoes *tert*-butyl cation elimination to yield a neutral radical; the second oxidation then produces a diazonium species, which is deprotonated to afford the diazo compound. The corresponding CV traces are provided in the SI. Protons supplied by Coll·HBF_4_ facilitate cathodic hydrogen evolution, thereby suppressing undesired side reactions. The *in situ* generated diazo intermediate subsequently undergoes Rh(ii)-catalysed N_2_ extrusion to form a reactive rhodium carbenoid, which engages the alkene in a concerted [2 + 1] cycloaddition to furnish the desired cyclopropane product and regenerate the catalyst (see SI for Mechanistic Scheme).^15^

**Fig. 3:**
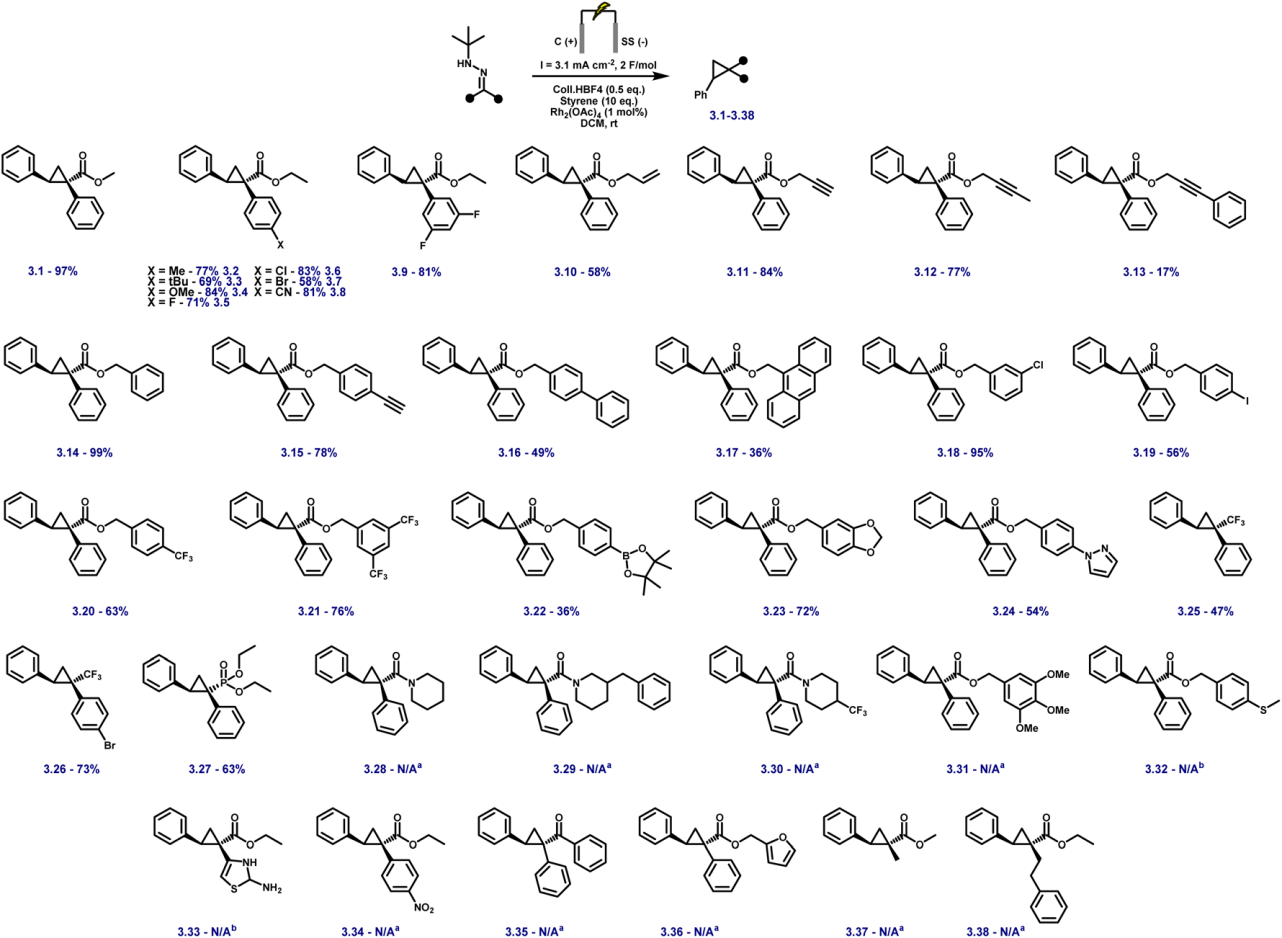
Substrate scope for the electrosynthesis of cyclopropanes with varying hydrazones using optimised conditions. Reactions were carried out on a 0.4 mmol scale at rt in a 5 mL ElectraSyn cell equipped with C and SS electrodes; yields are reported for pure, isolated products. ^*a*^No diazo formation; hydrazone recovered. ^*b*^Decomposition observed.

With the optimised electrochemical conditions in place, the scope of hydrazone substrates was explored using styrene as the olefin partner (Fig. 3). A broad range of aryl hydrazones bearing electron-donating, electron-withdrawing, and sterically demanding groups underwent smooth cyclopropanation, delivering functionalised products in good to excellent yields. Electron-rich substrates (3.2–3.4) exhibited high reactivity, while electron-deficient analogues containing fluoro, chloro, bromo, or cyano substituents (3.5–3.8) were equally well tolerated. Notably, the *m*,*m'*-difluoro derivative (3.9) underwent clean conversion without any dehalogenation.

**Fig. 4:**
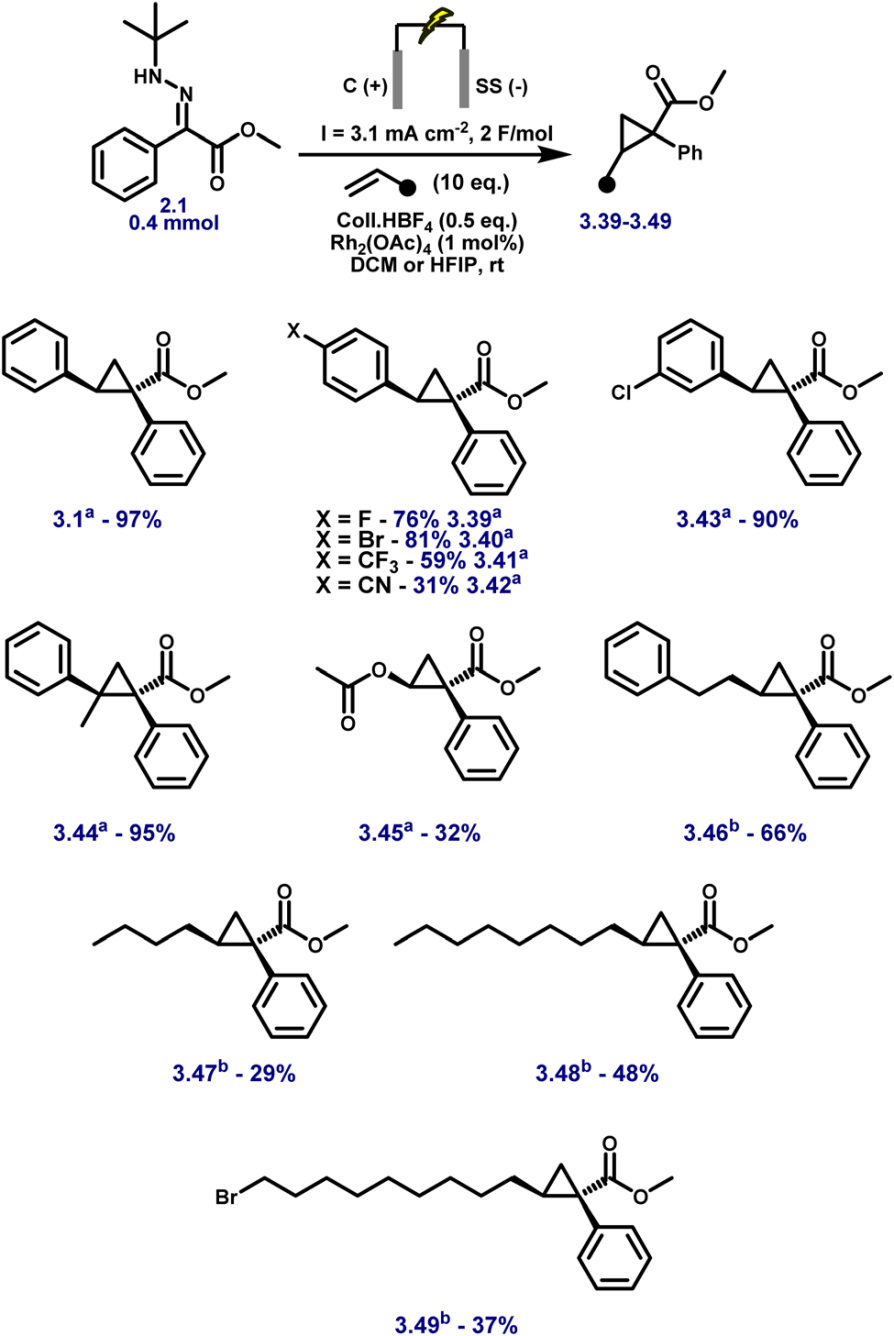
Substrate scope for the electrosynthesis of cyclopropanes with varying olefins using optimised conditions. Reactions were carried out on a 0.4 mmol scale at rt in a 5 mL ElectraSyn cell equipped with C and SS electrodes, and yields are reported for pure, isolated products. Solvent: (a) DCM; (b) HFIP.

Aryl esters incorporating allylic, propargylic, and internal alkyne groups (3.10–3.13) also performed well, and benzylic esters gave near-quantitative yields (3.14). Substrates bearing extended π-systems (3.15–3.17), strongly electron-withdrawing substituents such as –Cl, –I, –CF_3_, and bis-CF_3_ (3.18–3.21), and sterically hindered motifs including Bpin, methylenedioxy, and pyrazole (3.22–3.24) all proved compatible. Crucially, the method enabled efficient cyclopropanation of challenging diazo precursors such as trifluoromethyl hydrazones (3.25–3.26) and phosphonates (3.27), which are often unstable or poorly reactive under conventional conditions. Together, these results demonstrate the broad applicability, high functional group tolerance, and robustness of the electrochemical platform for diazo-based carbene transfer. While the protocol demonstrates broad substrate scope, several hydrazones failed to yield the desired cyclopropanes, highlighting limitations associated with redox properties, steric hindrance, and electronic structure. Piperidine-containing hydrazones (3.28–3.30) and methoxy-substituted analogues (3.31) remained unreacted, likely due to their elevated oxidation potentials arising from strong electron-donating groups, with additional steric congestion in the latter further impeding reactivity. Sulfur-containing substrates (3.32, 3.33) were also incompatible, consistent with their propensity for direct anodic oxidation. Notably, nitrophenyl hydrazone 3.34 failed to cyclopropanate, despite the typically beneficial electron-withdrawing nature of the nitro group; this may result from competitive oxidation of the nitroarene and diminished nucleophilicity at the hydrazone carbon, obstructing diazo formation or subsequent carbene transfer. Similarly, 1,2-diphenylhydrazone 3.35 was unreactive, likely due to π-donation from both aryl groups, which stabilises the putative carbene and attenuates its electrophilicity, disfavouring cyclopropanation. Furan-containing substrate 3.36 gave no product, presumably due to anodic oxidation of the electron-rich heterocycle. Lastly, unactivated aliphatic hydrazones (3.37, 3.38) also failed, underscoring the critical role of aromatic substitution in stabilising key intermediates during diazo formation and carbene transfer. For compounds 3.1–3.27, faradaic efficiencies, averaging 45%, were measured under representative reaction conditions and are reported in Table S8 (SI), confirming efficient charge utilisation across the reaction manifold. Having established the hydrazone scope (Fig. 3), we next varied the olefin coupling partner while keeping the hydrazone constant (Fig. 4). A broad range of styrene derivatives bearing electron-donating and electron-withdrawing substituents underwent efficient cyclopropanation, affording products (3.39–3.43) in moderate to excellent yields. Functional groups such as alkyl, halogen, and nitrile were well tolerated, demonstrating the method's broad applicability. A key finding was the critical role of solvent in enabling reactivity beyond activated olefins. Substituting dichloromethane (DCM) with hexafluoroisopropanol (HFIP) enabled the cyclopropanation of unactivated alkenes, including vinyl acetate and terminal aliphatic alkenes (3.46–3.49), which remained unreactive in DCM.

Importantly, using only a few equivalents of HFIP in DCM was insufficient to trigger reactivity; HFIP had to be used as the bulk solvent to enable efficient carbene transfer. Upon switching to HFIP, a visible colour change of the Rh(ii) catalyst was observed, potentially indicating ligand exchange between HFIP and the acetate bridges of Rh_2_(OAc)_4_. This effect, combined with HFIP's strong hydrogen-bonding ability and electron-withdrawing nature, likely enhances catalyst solvation, increases rhodium electrophilicity, and stabilises reactive intermediates, collectively facilitating cyclopropanation under otherwise unreactive conditions.^59–61^

Once again, no accumulation of diazo intermediates was detected, as confirmed by HPLC-MS and UV analysis of aliquots taken during electrolysis. This reinforces the transient nature of the diazo species and underscores the intrinsic safety of this real-time generation–consumption system. However, the relatively high acidity of HFIP (p*K*_a_ ≈ 9.3) led to uncontrolled polymerisation when applied to styrene substrates,^62,63^ thus limiting its use to non-activated olefins. As a result, DCM and HFIP emerge as complementary solvent systems: DCM enables efficient cyclopropanation of activated olefins such as styrenes, while HFIP expands the scope to include challenging unactivated alkenes that are otherwise unreactive under standard conditions.

The electrochemical cyclopropanation protocol exhibits excellent scalability, operational robustness, and an exceptional safety profile. The batch process was successfully scaled from 0.4 mmol to both 1.0 mmol and 1.0 g of hydrazone without any loss in efficiency, consistently affording the desired cyclopropane product (3.1) in ∼90% yield. Just as on small scale, no accumulation of diazo intermediates was observed on larger scale, confirming their transient nature and the tightly coupled generation–consumption sequence of this transformation. This continuous and controlled reactivity significantly reduces thermal and reactive risk, making the process particularly attractive for industrial implementation and flow applications.

Beyond its safety and scalability, the method offers several key advantages over traditional diazo protocols. It proceeds under mild, environmentally benign conditions, using electricity as the sole oxidant and avoiding hazardous reagents such as hydrazine or its hydrate. Despite employing a rhodium catalyst, the low loading (1 mol%) and high turnover render the process both economically and environmentally favourable. The reaction is operationally simple, requires minimal optimisation, and proceeds in a single step without the need for intermediate isolation or multistep setups, delivering high yields in short reaction times.

In efforts to further improve sustainability and reduce reliance on molecular rhodium, we evaluated Rh-plated electrodes as a potential heterogeneous alternative. However, these attempts proved unsuccessful: no product formation was observed, indicating that the homogeneous Rh_2_(OAc)_4_ catalyst remains essential for effective carbene transfer under the current conditions.

Further optimisation focused on reducing the need for a large excess of olefin, typically 10 equivalents. This is standard practice in the literature for diazo-based cyclopropanation reactions, where a large excess of alkene, often styrene, is routinely employed to outcompete side reactions and maximise yields.^64,65^ However, such conditions are impractical for synthetic applications, especially at scale or with valuable olefin partners. To address this, we explored strategies to lower the olefin loading while maintaining high efficiency. By increasing the overall reaction concentration, we successfully reduced the styrene stoichiometry to just 2 equivalents without significantly compromising yield, thereby improving atom economy and synthetic practicality. The 2 equivalent protocol is general for styrenes and selected substrates, while 10 equivalents remain optimal for certain unactivated or less reactive olefins due to slower carbene capture.

**Fig. 5:**
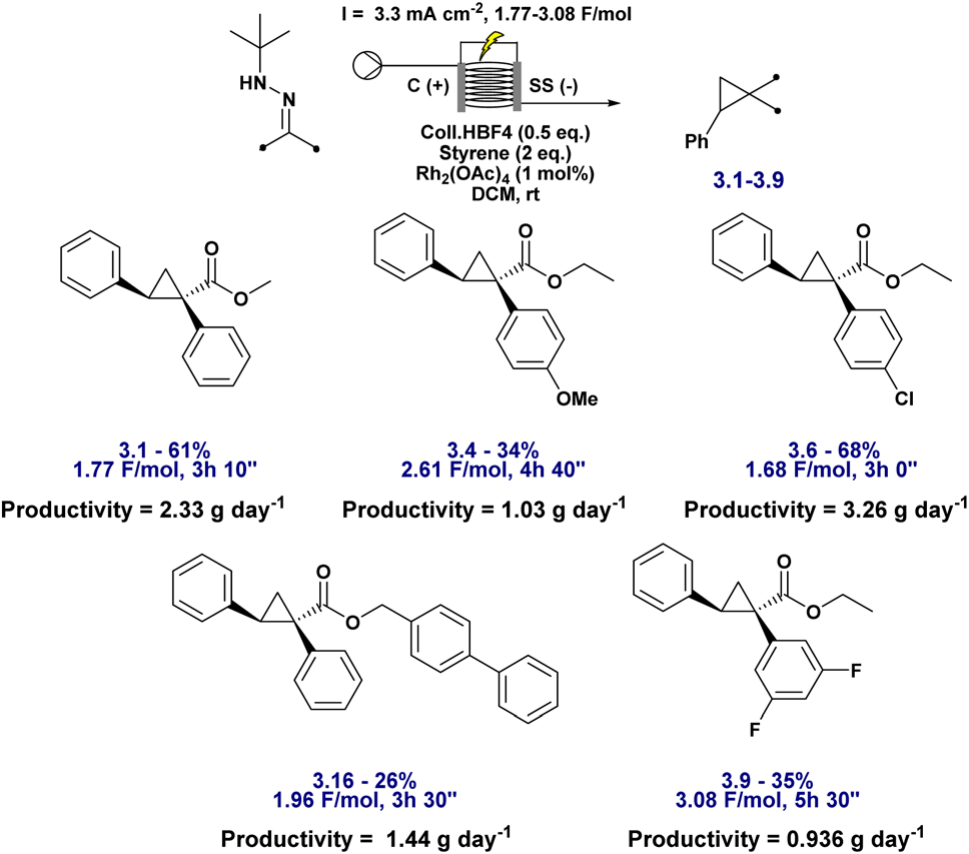
Substrate scope for the electrosynthesis of cyclopropanes with varying hydrazones using high concentration conditions. Reactions were carried out on a 2 mmol scale at rt in a 5 mL ElectraSyn cell equipped with C and SS electrodes, and yields are reported for pure, isolated products.

Under these modified conditions, the reaction was performed on a 2 mmol scale in a 5 mL ElectraSyn cell using carbon graphite and stainless-steel electrodes, 1 mol% Rh_2_(OAc)_4_, and 0.5 equivalents of Coll·HBF_4_ in DCM (Table 4, entry 4). A current density of 10.7 mA cm^−2^ and a charge of 2 F mol^−1^ were applied, consistent with previous conditions. Despite the fivefold reduction in olefin, good yields were obtained across a small set of substrates, demonstrating the feasibility of using more synthetically relevant alkene stoichiometries without compromising efficiency (Fig. 5).

**Table 4:** Optimisation of conditions for the electrosynthesis of cyclopropane 3.1. Reactions were carried out at room temperature (rt) in a 5 mL ElectraSyn cell equipped with carbon and stainless steel electrodes, and yields were determined by ^1^H NMR using CH_2_Br_2_ as an internal standard

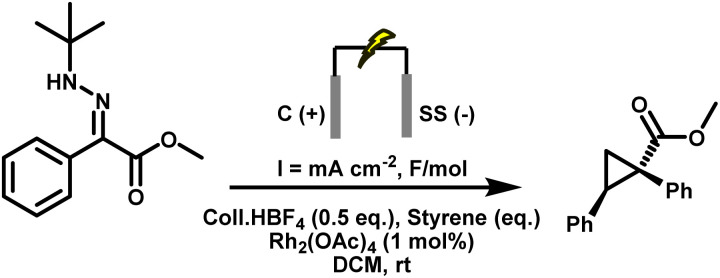				
Entry	Hydrazone (eq.)	Styrene (eq.)	Conditions	Yield
1	2	1	3.1 mA cm^−2^, 2 F mol^−1^	30%
2	2	1.1	3.1 mA cm^−2^, 2 F mol^−1^	61%
3	2	2	3.1 mA cm^−2^, 2 F mol^−1^	72%
**4**	**2**	**2**	**10.7 mA cm^−^** ^**2**^ **, 2 F mol^−^** ^**1**^	**70%**
5	4.3	2	10.7 mA cm^−2^, 2 F mol^−1^	55%

**Scheme 5:**
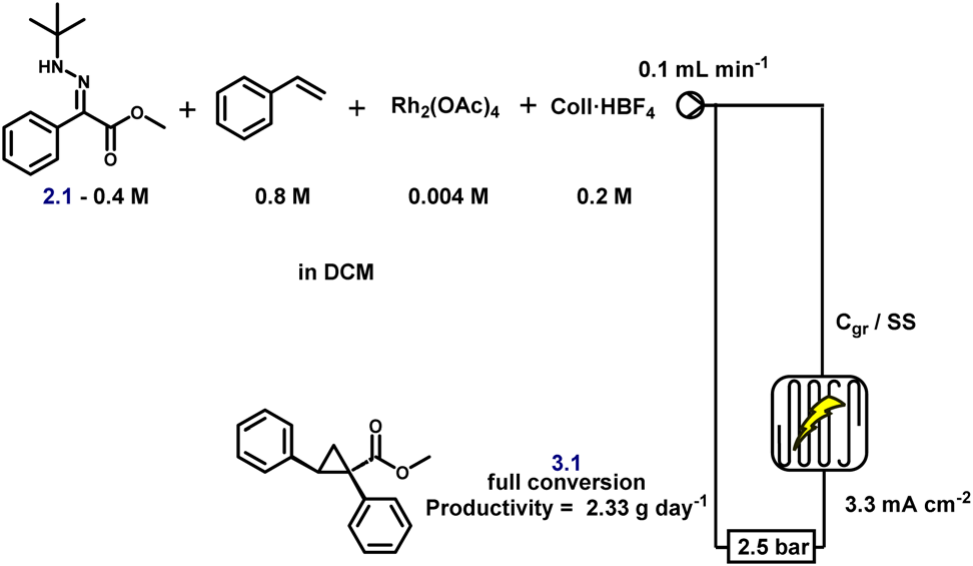
Continuous flow eCyclopropanation of hydrazone.

As a proof of concept, and to further enhance both scalability and operational safety, we translated the batch protocol to a continuous flow electrochemical setup. Flow electrochemistry offers intrinsic advantages for scale-up,^66^ particularly in tandem processes where reactive intermediates, such as diazo compounds, must be generated and consumed in real time. We first evaluated whether full conversion could be achieved in a single pass or whether recirculation was required. A 0.4 M solution of hydrazone and 0.8 M styrene was passed through the flow cell containing 1 mol% Rh_2_(OAc)_4_ and 0.5 equivalents of Coll·HBF_4_ in DCM at a flow rate of 0.1 mL min^−1^ (Scheme 5). Electrolysis was performed between a carbon graphite anode and a stainless steel cathode under a current density of 3.3 mA cm^−2^. Under these concentrated conditions, conversion was incomplete after a single pass, necessitating recirculation to reach full conversion. Importantly, this does not present a technical barrier: it simply reflects the need for a longer electrolysis path or cell residence time if single-pass processing is desired, both of which are straightforward adjustments in flow reactor design.

**Fig. 6:**
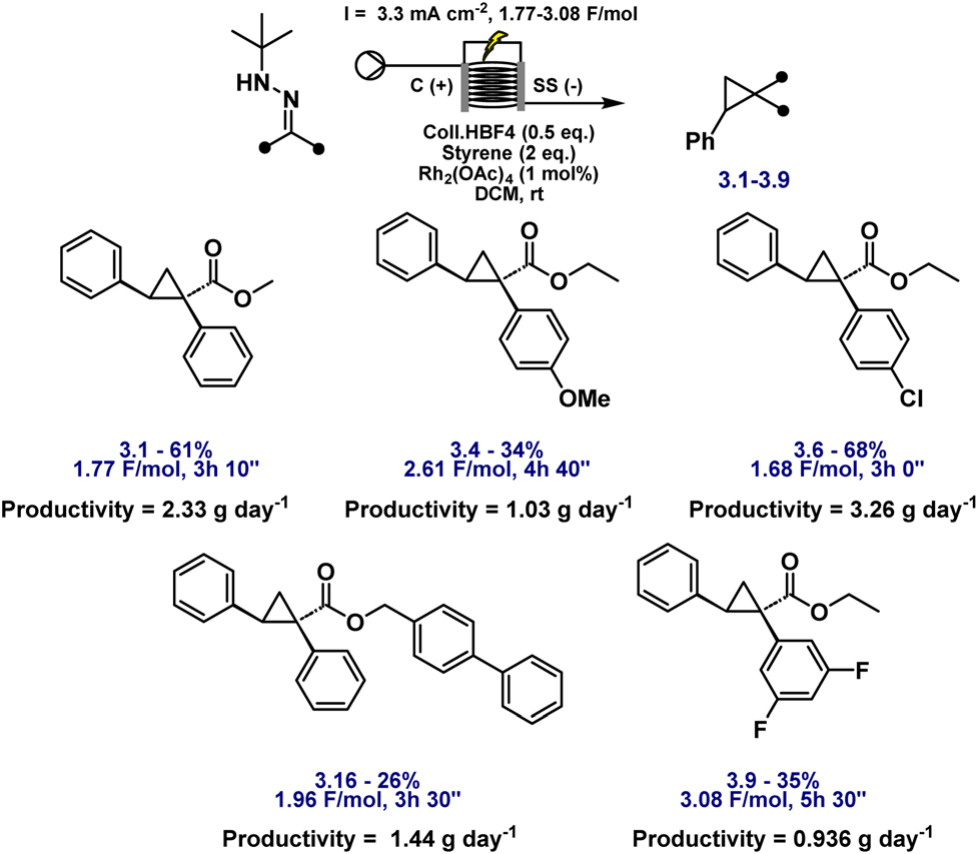
Substrate scope for the flow electrosynthesis of cyclopropanes with varying hydrazones using unoptimized high concentration conditions.

A preliminary substrate scope was evaluated under flow conditions (Fig. 6). While yields varied across substrates, highlighting the need for substrate-specific fine-tuning, these experiments demonstrate the feasibility of continuous, safe, and scalable cyclopropane synthesis *via* electrochemical diazo generation. Crucially, HPLC analysis confirmed the complete absence of diazo intermediate accumulation at any stage, underscoring the transient nature of these species under flow. The system achieved a productivity of up to 3.26 g day^−1^, underscoring its potential for practical scale-up and industrial relevance. As anticipated, the space-time yield is deliberately constrained by the requirement to closely match the electrochemical diazo generation rate with catalytic turnover while operating within safe current densities. Importantly, this productivity represents a proof of concept obtained under unoptimised conditions, reflecting a design strategy that prioritises controlled, transient diazo formation over maximal throughput. Further gains are expected through standard process intensification strategies, including optimisation of reactor design, electrode surface area, and flow path length; however, such engineering development lies beyond the scope of the present study.

To further probe scalability within this safety-led framework, a 1 g-scale experiment was performed on substrate 2.1 using a larger Ammonite8 flow cell (see SI for details). In this case, the isolated yield decreased to 40 percent, which was attributed to selectivity erosion during the extended recirculation period. While this experiment likewise represents a proof of concept rather than a fully optimised process, it highlights a clear and safety-consistent route forward: implementation of a longer flow reactor would enable single-pass operation, thereby avoiding prolonged recirculation, maintaining transient diazo concentrations, and enabling higher yields.

## Conclusions

We have developed a one-pot, safe, scalable, and operationally simple electrochemical method for Rh(ii)-catalysed cyclopropanation *via* the *in situ* generation of diazo compounds from *tert*-butyl hydrazones. This integrated platform addresses long-standing limitations of diazo chemistry, including hazardous precursors, intermediate instability, and poor scalability, by merging anodic oxidation and catalytic carbene transfer into a single, continuous operation. The use of a bench-stable mono-protected hydrazone, thereby avoiding hazardous hydrazine monohydrate, together with a non-nucleophilic supporting electrolyte and mild reaction conditions, enables high yields across a broad range of hydrazone and olefin substrates with excellent functional group tolerance.

Crucially, the protocol prevents accumulation of diazo intermediates at any stage of the process, ensuring that these high-energy species are generated and consumed in real time. This transient mode of operation removes the need for isolation or handling of free diazo compounds and fundamentally improves the safety profile of diazo chemistry by enabling precise electrochemical control over diazo generation. Translation to continuous flow further highlights the robustness and industrial relevance of the approach, providing a scalable route to cyclopropanes while maintaining stringent safety constraints.

By demonstrating that electrochemistry can deliver both the control and intrinsic safety required to manage diazo intermediates, this work offers a practical solution to a long-standing challenge in synthetic chemistry. More broadly, it redefines how highly reactive intermediates can be harnessed through safety-by-design principles, opening new opportunities for sustainable and scalable carbene transfer chemistry. Future efforts will focus on expanding substrate scope, broadening catalyst compatibility, and further enhancing the versatility of this electrochemical platform.

## Author contributions

J. M. W., M. G., S. H. and M. T. conducted all experimental work. J. M. W. collected and refined data analysis. K. L. directed the research project. All authors were involved in writing the manuscript.

## Conflicts of interest

The authors declare no conflicts of interest.

## Supplementary Material

SC-017-D5SC08940A-s001

## Data Availability

All data supporting the findings of this study, including experimental procedures, optimisation data, and NMR spectra, are provided in the supplementary information (SI). Supplementary information is available. See DOI: https://doi.org/10.1039/d5sc08940a.
