# Supplementary material for: eCyclopropanation – a safe and scalable electrochemical route to cyclopropanes
Source: Chem Sci. 2026 Feb 11;17(14):7135–45. doi: 10.1039/d5sc08940a (PMC12910288; doi:10.1039/d5sc08940a)
Supplement: SC-017-D5SC08940A-s001 [file SC-017-D5SC08940A-s001.pdf]

Supplementary Information (SI) for Chemical Science.
This journal is © The Royal Society of Chemistry 2026

Electronic Supporting Information

eCyclopropanation – A Safe and Scalable Electrochemical Route to Cyclopropanes

Dr Jamie M. Walsh[a], Marco Galzignato[a], Dr Shusuke Hattori[a], Dr Marylise Triacca[a], and Prof. Dr Kevin Lam[a]*

Table of Contents

Materials and Methods 2

General Experimental Procedures 2

General Procedure A for the synthesis of ketone starting materials[1] 4

General Procedure B for the synthesis of ketone starting materials[2] 4

General Procedure C for the synthesis of ketone starting materials[3] 4

General Procedure D for the synthesis of ester/amide/ketone hydrazones[8] 10

General Procedure E for the synthesis of aryl-CF_3 hydrazones[9] 10

Batch-Electrochemical Reaction Setup 19

Flow-Electrochemical Reaction Setup 19

Cyclic Voltammetry Studies 20

DSC Studies 21

HPLC-UV traces 21

Proposed Reaction Mechanism 22

Optimisation tables for electrochemical synthesis of diazo and cyclopropane compounds 24

General Procedure F for the electrochemical cyclopropanation of activated hydrazones 30

Optimisation for electrochemical cyclopropanation of non-activated olefins 37

General Procedure G for the electrochemical cyclopropanation of hydrazones with varying olefins 38

General Procedure H for the electrosynthesis of stabilised diazo compounds 41

General Procedure I for the electrochemical cyclopropanation of activated hydrazones with decreased olefin concentration 43

General Procedure J for the electrochemical cyclopropanation of activated hydrazones in flow 45

Faradaic Efficiency 47

References 48

NMR spectra of previously described compounds 49

Materials and Methods

General Experimental Procedures

All reactions were carried out under aerobic conditions unless otherwise stated. All solvents and commercially available reagents were purchased from standard vendors and used without further purification unless otherwise stated. Electrolyses were performed using an IKA Electrasyn 2.0 using carbon graphite working and counter electrode. Analytical thin-layer chromatography (TLC) was performed using silica gel plates (0.25 mm thickness) on aluminum support. Visualization was accomplished by irradiation with a UV lamp and/or staining with either $KMnO_4$ or ninhydrin. Column chromatography was performed over Silica gel 60 Å (40-63µ mesh) using a CombiFlash Rf Lumen automatic flash chromatography system. Residual solvent was removed using a static oil pump (< 10 mbar). The cooling of reaction mixtures was achieved using an ice bath (0 °C).

NMR spectra were obtained using a JEOL ECZR 400 (^{1}H 399.78 MHz; ^{19}F 376.17 MHz; ^{13}C 100.53 MHz) or ECA 500 (^{1}H 500.16 MHz; ^{13}C 125.77 MHz) spectrometer and are reported relative to the residual solvent resonances. All heteronuclear NMR spectra were ^{1}H-decoupled and recorded at room temperature unless otherwise stated. Data for ^{1}H NMR spectra are reported as follows: chemical shift (δ, ppm), coupling constant (Hz), multiplicity (s, singlet; d, doublet; t, triplet; m, multiplet; br, broad) and integration. Data for ^{13}C and ^{19}F NMR are reported in terms of chemical shift (δ, ppm). IR spectra were recorded on a Perkin Elmer Spectrum Two instrument as neat samples.

High Resolution Mass Spectrometry (HRMS) data were obtained by Dr. Iain Goodall and Dr. Bini Claringbold of the University of Greenwich Mass Spectrometry Service using a Waters Synapt G2 hybrid Quadrupole-orthogonal acceleration time-of-flight configuration (Waters, Manchester, UK) operating in Resolution Mode (M/ΔM ≥ 18,000), fitted with a Waters Acquity UPLC binary solvent chromatographic pump system. The column used was a reversed-phase Acquity BEH C18 2.1 x 50 mm, 1.7-micron bead, running a 3- minute separation with an A:B eluent mixture comprising of either deionised water with 0.1% (v:v) formic acid and acetonitrile with 0.1% (v:v) formic acid (negative mode) respectively or deionised water with 0.1% (v:v) ammonium hydroxide and acetonitrile with 0.1% (v:v) ammonium hydroxide (positive mode) respectively. Mass calibration of the instrument was performed using sodium formate cluster ions, and an orthogonal Lock-SprayTM ESI probe was used with a lock mass calibrant, leucine-enkephalin. The pseudomolecular leucine-enkephalin ion at *m/z* = 554.2615 (Negative Ion Mode), and *m/z* = 556.2771 (Positive Ion Mode), was used as the internal mass correction calibrant. Additional samples were analyzed on a Thermo LTQ Orbitrap XL coupled with a heated electrospray source (HESI). The capillary temperature was set to 275 °C and a voltage of 21 V. The sheath gas and auxiliary gas flow were set to 10 and 5 L h^{-1} respectively and the source current and voltage set to 100 µA and 5 kV. A solution of analyte (0.1 mg/ml) and sodium formate (1% v/v) in acetonitrile was added by direct infusion (10 µL/min) into the mass spectrometer using a Hamilton syringe (250 µL).

Gas-Chromatography Mass Spectrometry (GC-MS) data were obtained using a Shimadzu Nexis GC-2030 gas chromatograph connected to a GCMS-QP2020 NX gas chromatograph mass spectrometer, equipped with an AOC-20i Plus auto injector. The column was a CD-5MS capillary column (30 m x 0.25 mm x 0.25 µm), with helium as the carrier gas. The sample injection volume was 1 µL, and separations run over a 5-minute period with an increasing oven temperature (gradient) between 40 – 280 °C. Results were visualised and manipulated using LabSolutions GCMS solution version 4.50.

High-Performance Liquid Chromatography-Mass Spectrometry (HPLC-MS) data were obtained using a Shimadzu LC-2050C 3D coupled with a Shimadzu LCMS-2020 FCV-20AH2. The column was an Ascentis Express 90Å AQ-C18, 2.7 µm. Results were visualised and manipulated using LabSolutions GCMS solution version 5.114.

DSC data were obtained using a Discovery DSC 2500 with RSC90 cooling accessory (heat flux DSC) (TA Instruments, Waters, USA). Calibrate with indium for temperature and cell constant according to the manufacturer's instructions. Nitrogen was used as purge gas at 50mL/min flow rate. The sample mass was ca 2.5 mg on Tzero aluminum pans and hermetic lids (TA Instruments, Waters, USA). The temperature programme scanned from 0 C to 200 C at 10 C/min scan rate.

The 1g flow experiment was carried out using the Ammonite 8 flowcell. The cell was manufactured by Cambridge reactor Design Ltd. The cell design is based on two circular plate electrodes, diameter 149 mm and a spiral solution channel, width 5 mm where the electrolyte flows from the center of the discs to their perimeter. The spiral solution channel was created by machining a spiral groove (2 mm in width and 0.5 mm in depth) into the stainless steel cathode electrode so that there was a 5 mm spacing between neighboring sections of the groove. A polymer gasket/spacer, thickness 1 mm, was lazer cut so that it fitted into the groove. When compressed against a flat carbon filled polyvinylidene fluoride (C/PVDF) plate electrode, this creates a channel 2 m long, 5 mm wide with an interelectrode gap of 0.5 mm.

Cyclic voltammetry studies were carried out using an Autolab 302N potentiostat interfaced through Nova 2.1 software to a personal computer. Electrochemical measurements were performed in a glovebox under an atmosphere of dinitrogen with oxygen and water levels of less than 5 ppm at 298 K, with solvents that had been thoroughly degassed and purified by passing through an alumina-based purification system. Sample concentrations of 1.0 mM were used, alongside 0.1 M $[^{n}Bu_4N][PF_6]$ supporting electrolyte concentrations. Experiments were conducted using a standard three-electrode setup comprising of a glassy carbon disc working electrode, platinum wire counter electrode, and AgCl-coated silver wire as a pseudo-reference electrode. Potentials are reported relative to the $[FeCp_2]^{+/0}$ redox couple, obtained through the addition of ferrocene to the analyte solution.

General Procedure A for the synthesis of ketone starting materials[1]

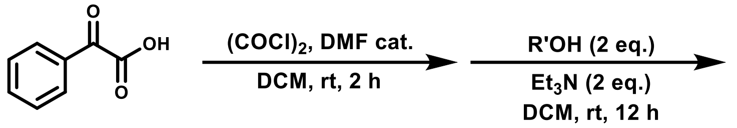

To a stirred solution of 2-oxo-2-phenylacetic acid (20 mmol, 1 eq.) in DCM (20 mL) was added oxalyldichloride (2.79 g, 1.1 eq.) and two drops of DMF. The mixture was stirred for 2 h at room temperature. The solvent was then removed under reduced pressure and the resulting α-acyl chloride was dissolved in DCM (10 mL) and added to a 100 mL round-bottomed flask containing alcohol R'OH (40 mmol), triethylamine (40 mmol, 4.0 g) and DCM (20 mL) at 0 °C and stirred at room temperature overnight. Upon completion, the resulting mixture was washed with water and extracted with DCM (3 x 20 mL). The combined organics were dried over $MgSO_4$ and the solvent removed under reduced pressure. The crude residue was purified by silica gel flash chromatography (EtOAc/Pentane = 1/30) to afford the benzoylformate esters.

General Procedure B for the synthesis of ketone starting materials[2]

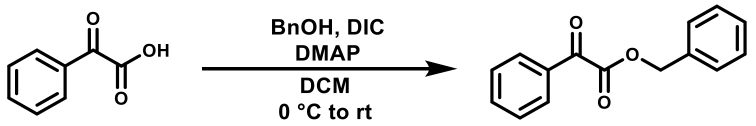

To a stirred solution of 2-oxo-2-phenylacetic acid (1.09 g, 7.26 mmol) and DMAP (0.237 g, 1.94 mmol) in DCM (24 mL) was added dropwise benzyl alcohol (0.5 mL, 4.84 mmol). N,N '-diisopropylcarbodiimide (DIC, 2.20 mL, 14.5 mmol) was added dropwise at 0 °C. The mixture was warmed to room temperature and stirred overnight. Excess DIC was consumed by the addition of AcOH and EtOH. After 30 min, the mixture was quenched with 1M HCl and neutralized with saturated $NaHCO_3$ aqueous solution and diluted with DCM followed by washing with water and brine. The combined organics were dried over $MgSO_4$ and purified by silica gel flash chromatography (EtOAc/Hexane = 1/100) to afford the desired ketone.

General Procedure C for the synthesis of ketone starting materials[3]

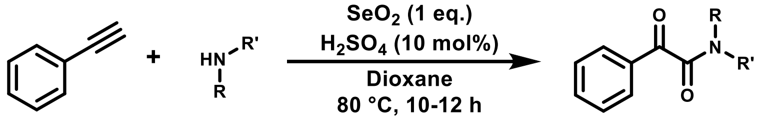

SeO_2 (20 mmol) was added to a solution of phenylacetylene (20 mmol) in dioxane (20 ml) followed by the addition of 10 mol% of H_2SO_4 and amine (20 mmol). The reaction mixture was then heated at 80 °C for 10-12 h and the product formation was monitored by TLC. After completion, the reaction mixture was extracted with ethyl acetate (3 x 100 ml) and washed with water and brine. The combined organics were dried over $MgSO_4$ and the solvent removed under reduced pressure. The crude residue was purified by silica gel flash chromatography (EtOAc/Hexane = 1/100) to afford the desired ketone.

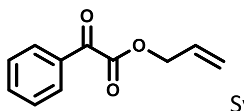

Synthesised according to general procedure A from prop-2-en-1-ol (2.3232 g, 40 mmol) to afford ketone **1.1** (3.1573 g, 83%) as a brown liquid. Ketone **1.1**: ^{1}H NMR (400 MHz, $CDCl_3$): δ_H 8.02-8.00 (m, 2H), 7.68-7.64 (m, 1H), 7.51 (t, J = 7.85 Hz, 2H), 6.07-5.97 (m, 1H), 5.47-5.33 (m, 2H), 4.89-4.86 (m, 1H); ^{13}C {^{1}H} NMR (101 MHz, $CDCl_3$): δ_C 186.2, 163.6, 135.1, 132.6, 130.9, 130.2, 129.0, 120.2, 66.7; IR (υ, cm^{-1}, neat): 3041, 1743, 1690; HRMS (ESI): m/z calcd for $C_{11}H_{10}O_3$: 191.0708 $[M+H]^+$; found: 191.0724

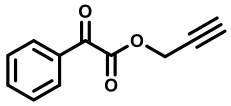 Synthesised according to general procedure A from prop-2-yn-1-ol (2.2424 g, 40 mmol) to afford ketone **1.2** (1.7689 g, 47%) as a yellow oil. Ketone **1.2**: ^{1}H NMR (400 MHz, $CDCl_3$): δ_H 8.04-8.01 (m, 2H), 7.73-7.68 (m, 1H), 7.56-7.51 (m, 2H), 4.97 (d, J = 2.48 Hz, 2H), 2.60 (t, J = 2.48 Hz, 1H); ^{13}C {^{1}H} NMR (101 MHz, $CDCl_3$): δ_C 185.5, 162.9, 135.4, 132.4, 130.3, 129.1, 76.5, 76.4, 53.5; IR (υ, cm^{-1}, neat): 3315, 3073, 1744, 1690; HRMS (ESI): m/z calcd for $C_{11}H_8O_3$: 189.0552 [M+H]$^+$; found: 189.0564

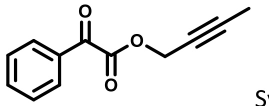 Synthesised according to general procedure A from but-2-yn-1-ol (2.8036 g, 40 mmol) to afford ketone **1.3** (3.0332 g, 75%) as a yellow oil. Ketone **1.3**: ^{1}H NMR (400 MHz, $CDCl_3$): δ_H 8.03-8.01 (m, 2H), 7.69-7.65 (m, 1H), 7.54-7.51 (m, 2H), 7.94 (q, J = 2.45 Hz, 2H), 1.89 (t, J = 2.45 Hz, 3H); ^{13}C {^{1}H} NMR (101 MHz, $CDCl_3$): δ_C 185.7, 163.2, 135.2, 132.5, 130.3, 129.1, 84.9, 72.1, 54.5, 3.8; IR (υ, cm^{-1}, neat): 3301, 3023, 1754, 1693; HRMS (ESI): m/z calcd for $C_{12}H_{10}O_3$: 203.0708 [M+H]$^+$; found: 203.0720

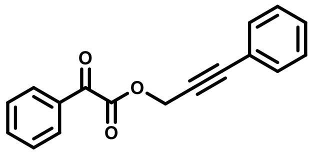 Synthesised according to general procedure A from 3-phenylprop-2-yn-1-ol (5.2864 g, 40 mmol) to afford ketone **1.4** (4.5456 g, 86%) as a yellow oil. Ketone **1.4**: ^{1}H NMR (400 MHz, $CDCl_3$): δ_H 8.05-8.03 (m, 2H), 7.64-7.61 (m, 1H), 7.49-7.46 (m, 4H), 7.33-7.30 (m, 3H), 5.20 (s, 2H); ^{13}C {^{1}H} NMR (101 MHz, $CDCl_3$): δ_C 185.5, 163.1, 135.2, 132.5, 132.0, 130.2, 129.2, 129.1, 128.5, 122.0, 88.2, 81.8, 54.4; IR (υ, cm^{-1}, neat): 3298, 2997, 1722, 1689; HRMS (ESI): m/z calcd for $C_{17}H_{12}O_3$: 265.0865 [M+H]$^+$; found: 265.0877

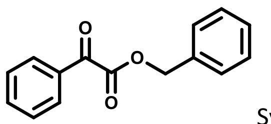 Synthesised according to general procedure B from benzyl alcohol (0.5236 g, 4.84 mmol) to afford ketone **1.5** (1.0815 g, 93%) as a colourless oil. Ketone **1.5**: ^{1}H NMR (400 MHz, $CDCl_3$): δ_H 7.98-7.96 (m, 2H), 7.66-7.63 (m, 1H), 7.51-7.44 (m, 4H), 7.42-7.38 (m, 3H), 5.42 (s, 2H); ^{13}C {^{1}H} NMR (101 MHz, $CDCl_3$): δ_C 186.2, 163.8, 135.1, 134.7, 132.5, 130.2, 129.0, 129.0, 128.9, 128.7, 67.9; IR (υ, cm^{-1}, neat): 2941, 1726, 1688; HRMS (ESI): m/z calcd for $C_{15}H_{12}O_3$: 241.0865 [M+H]$^+$; found: 241.0876

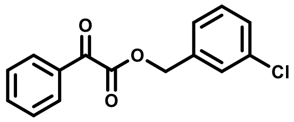 Synthesised according to general procedure B from 3-chlorobenzyl alcohol (0.6901 g, 4.84 mmol) to afford ketone **1.6** (1.1567 g, 87%) as a yellow oil. Ketone **1.6**: ^{1}H NMR (400 MHz, $CDCl_3$): δ_H 7.99-7.97 (m, 2H), 7.68-7.65 (m, 1H), 7.52-7.49 (m, 2H), 7.44-7.43 (m, 1H), 7.34-7.32 (m, 3H), 5.38 (s, 2H); ^{13}C {^{1}H} NMR (101 MHz, $CDCl_3$): δ_C 185.9, 163.5, 136.6, 135.2, 134.8, 132.5, 130.2, 130.2, 129.1, 129.1, 128.7, 126.7, 66.9; IR (υ, cm^{-1}, neat): 2993, 1744, 1694; HRMS (ESI): m/z calcd for $C_{15}H_{11}ClO_3$: 275.0475 [M+H]$^+$; found: 275.0479

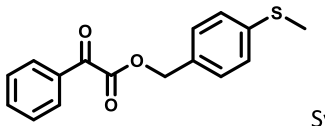 Synthesised according to general procedure B from 4-(methylthio)benzyl alcohol (0.7465 g, 4.84 mmol) to afford ketone **1.7** (1.1226 g, 81%) as a yellow oil. Ketone **1.7**: ^{1}H NMR (400 MHz, $CDCl_3$): δ_H 7.97-7.95 (m, 2H), 7.66-7.63 (m, 1H), 7.50-7.47 (m, 2H), 7.38-7.36 (m, 2H), 7.27-7.25 (m, 2H), 5.36 (s, 2H), 2.49 (s, 3H); ^{13}C {^{1}H} NMR (101 MHz, $CDCl_3$): δ_C 186.1, 163.7, 139.8, 135.1, 132.5, 131.2, 130.1, 129.4, 129.0, 126.5, 67.6, 15.6; IR (υ, cm^{-1}, neat): 3025, 1701, 1634; HRMS (ESI): m/z calcd for $C_{16}H_{14}O_3S$: 309. 0561 [M+Na]$^+$; found: 309.0570

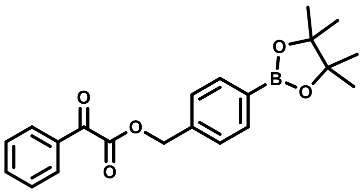

Synthesised according to general procedure B from (4-(4,4,5,5-tetramethyl-1,3,2-dioxaborolan-2-yl)phenyl)methanol (1.1330 g, 4.84 mmol) to afford ketone **1.8** (1.2053 g, 68%) as a yellow oil. Ketone **1.8**: ^{1}H NMR (400 MHz, $CDCl_3$): δ_H 7.97-7.95 (m, 2H), 7.85-7.83 (m, 2H), 7.66-7.63 (m, 1H), 7.50-7.47 (m, 2H), 7.45-7.44 (m, 2H), 5.43 (s, 2H), 1.35 (s, 12H); ^{13}C {^{1}H} NMR (101 MHz, $CDCl_3$): δ_C 186.1, 163.7, 137.6, 135.3, 135.1, 132.5, 130.2, 129.0, 127.7, 84.1, 67.7, 31.7, 25.0, 22.8, 14.3; IR (υ, cm^{-1}, neat): 3176, 2986, 1750, 1698; HRMS (ESI): m/z calcd for $C_{21}H_{23}BO_5$: 384.1982 $[M+NH_4]^+$; found: 384.1994

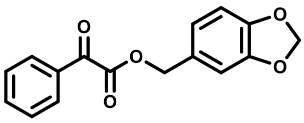

Synthesised according to general procedure B from piperonol (0.7364 g, 4.84 mmol) to afford ketone **1.9** (1.2108 g, 88%) as a yellow oil. Ketone **1.9**: ^{1}H NMR (400 MHz, $CDCl_3$): δ_H 7.97-7.95 (m, 2H), 7.66-7.63 (m, 1H), 7.51-7.48 (m, 2H), 6.94-6.93 (m, 2H), 6.82-6.80 (m, 1H), 5.98 (s, 2H), 5.31 (s, 2H); ^{13}C {^{1}H} NMR (101 MHz, $CDCl_3$): δ_C 186.2, 163.9, 148.2, 148.1, 135.1, 132.6, 130.2, 129.1, 128.3, 123.1, 109.6, 108.5, 101.5, 68.0; IR (υ, cm^{-1}, neat): 3166, 1739, 1687; HRMS (ESI): m/z calcd for $C_{16}H_{12}O_5$: 307.0582 $[M+Na]^+$; found: 307.0596

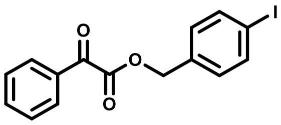

Synthesised according to general procedure B from 4-iodobenzyl alcohol (1.1328 g, 4.84 mmol) to afford ketone **1.10** (1.1165 g, 63%) as a yellow oil. Ketone **1.10**: ^{1}H NMR (400 MHz, $CDCl_3$): δ_H 7.94-7.92 (m, 2H), 7.64-7.62 (m, 2H), 7.58-7.54 (m, 1H), 7.43-7.39 (m, 2H), 7.13-7.11 (m, 2H), 5.30 (s, 2H); ^{13}C {^{1}H} NMR (101 MHz, $CDCl_3$): δ_C 185.5, 163.1, 137.5, 134.8, 134.0, 131.9, 130.1, 129.7, 128.7, 94.6, 66.7; IR (υ, cm^{-1}, neat): 3175, 1716, 1697; HRMS (ESI): m/z calcd for $C_{15}H_{11}IO_3$: 366.9904 $[M+H]^+$; found: 366.9906

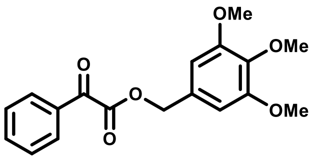

Synthesised according to general procedure B from (3,4,5-trimethoxyphenyl)methanol (0.9593 g, 4.84 mmol) to afford ketone **1.11** (1.3750 g, 86%) as a yellow oil. Ketone **1.11**: ^{1}H NMR (400 MHz, $CDCl_3$): δ_H 7.98-7.96 (m, 2H), 7.67-7.63 (m, 1H), 7.51-7.47 (m, 2H), 6.66 (s, 2H), 5.34 (s, 2H), 3.86 (s, 6H), 3.84 (s, 3H); ^{13}C {^{1}H} NMR (101 MHz, $CDCl_3$): δ_C 186.2, 163.7, 153.6, 153.3, 138.4, 135.2, 132.5, 130.2, 130.1, 129.1, 125.9, 105.8, 68.1, 61.0, 56.3; IR (υ, cm^{-1}, neat): 3187, 2932, 1753, 1689; HRMS (ESI): m/z calcd for $C_{18}H_{18}O_6$: 331.1182 $[M+H]^+$; found: 331.1194

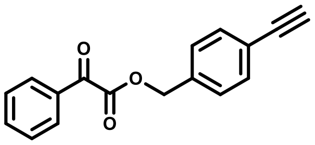

Synthesised according to general procedure B from (4-ethynylphenyl)methanol (0.6397 g, 4.84 mmol) to afford ketone **1.12** (1.0617 g, 83%) as a yellow oil. Ketone **1.12**: ^{1}H NMR (400 MHz, $CDCl_3$): δ_H 7.96-7.93 (m, 2H), 7.61-7.56 (m, 1H), 7.49-7.41 (m, 4H), 7.38-7.35 (m, 2H), 5.37 (s, 2H), 3.15 (s, 1H); ^{13}C {^{1}H} NMR (101 MHz, $CDCl_3$): δ_C 185.8, 163.4, 135.1, 135.0, 132.3, 132.2, 129.9, 128.8, 128.3, 122.5, 83.0, 78.2, 67.0; IR (υ, cm^{-1}, neat): 3307, 3054, 1740, 1695; HRMS (ESI): m/z calcd for $C_{17}H_{12}O_3$: 282.1130 $[M+NH_4]^+$; found: 282.1145

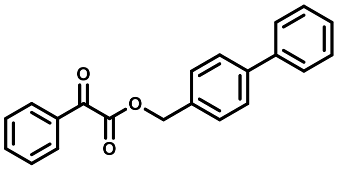

Synthesised according to general procedure B from biphenyl-4-yl methanol (0.8917 g, 4.84 mmol) to afford ketone **1.13** (1.3321 g, 87%) as a yellow oil. Ketone **1.13**: ^{1}H NMR (400 MHz, $CDCl_3$): δ_H 8.11-8.09 (m, 2H), 7.68-7.63 (m, 4H), 7.61-7.57 (m, 3H), 7.52-7.47 (m, 4H), 7.44-7.40 (m, 1H), 5.52 (s, 2H); ^{13}C {^{1}H} NMR (101 MHz, $CDCl_3$): δ_C 185.9, 163.5, 141.3, 140.1, 134.8, 133.4, 132.2, 129.8, 128.9, 128.7, 127.4, 127.2, 126.9, 67.2; IR (υ, cm^{-1}, neat): 3088, 1722, 1693; HRMS (ESI): m/z calcd for $C_{21}H_{16}O_3$: 334.1443 $[M+NH_4]^+$; found: 334.1454

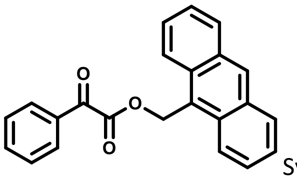

Synthesised according to general procedure B from 9-hydroxymethylanthracene (1.0080 g, 4.84 mmol) to afford ketone **1.14** (0.7249 g, 44%) as a yellow oil. Ketone **1.14**: ^{1}H NMR (400 MHz, $CDCl_3$): δ_H 8.51 (s, 1H), 8.43-8.40 (m, 2H), 8.04-8.01 (m, 2H), 7.92-7.90 (m, 2H), 7.63-7.59 (m, 2H), 7.57-7.49 (m, 3H), 7.39-7.35 (m, 2H), 6.46 (s, 2H); ^{13}C {^{1}H} NMR (101 MHz, $CDCl_3$): δ_C 186.2, 164.2, 134.9, 132.4, 131.4, 131.2, 130.0, 129.3, 128.9, 127.1, 125.3, 124.7, 123.7, 60.6; IR (υ, cm^{-1}, neat): 3016, 1717, 1689; HRMS (ESI): m/z calcd for $C_{23}H_{16}O_3$: 358.1443 $[M+NH_4]^+$; found: 358.1454

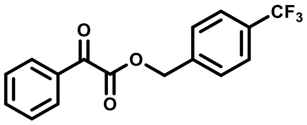

Synthesised according to general procedure B from 4-(trifluoromethyl)benzyl alcohol (0.8525 g, 4.84 mmol) to afford ketone **1.15** (1.2980 g, 87%) as a yellow oil. Ketone **1.15**: ^{1}H NMR (400 MHz, $CDCl_3$): δ_H 8.00-7.97 (m, 2H), 7.69-7.65 (m, 3H), 7.58-7.49 (m, 4H), 5.46 (s, 2H); ^{13}C {^{1}H} NMR (101 MHz, $CDCl_3$): δ_C 185.7, 163.4, 138.6, 135.3, 132.5, 131.3, 130.2, 129.1, 128.6, 126.0, 125.9, 125.9, 125.8, 66.8; ^{19}F NMR (471 MHz, $CDCl_3$): δ -62.6; IR (υ, cm^{-1}, neat): 3180, 2939, 1758, 1673; HRMS (ESI): m/z calcd for $C_{16}H_{11}F_3O_3$: 326.1004 $[M+NH_4]^+$; found: 326.1014

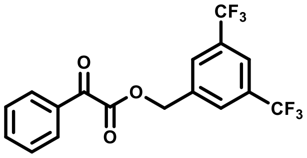

Synthesised according to general procedure B from 3,5-(trifluoromethyl)benzyl alcohol (1.1816 g, 4.84 mmol) to afford ketone **1.16** (1.5479 g, 85%) as a yellow oil. Ketone **1.16**: ^{1}H NMR (400 MHz, $CDCl_3$): δ_H 8.01-7.99 (m, 2H), 7.91-7.90 (m, 3H), 7.71-7.67 (m, 1H), 7.55-7.51 (m, 2H), 5.51 (s, 2H); ^{13}C {^{1}H} NMR (101 MHz, $CDCl_3$): δ_C 185.3, 163.1, 137.2, 135.4, 132.6, 132.3, 130.2, 129.2, 128.4, 124.5, 122.9, 121.8, 65.9 ^{19}F NMR (471 MHz, $CDCl_3$): δ -62.6; IR (υ, cm^{-1}, neat): 3166, 3129, 1756, 1690; HRMS (ESI): m/z calcd for $C_{17}H_{10}F_6O_3$: 394.0912 $[M+NH_4]^+$; found: 394.0918

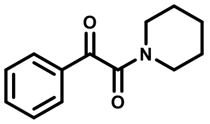

Synthesised according to general procedure C from piperidine (1.7030 g, 20 mmol) to afford ketone **1.17** (2.7811 g, 64%) as a yellow oil. Ketone **1.17**: ^{1}H NMR (400 MHz, $CDCl_3$): δ_H 7.96-7.94 (m, 2H), 7.66-7.62 (m, 1H), 7.53-7.50 (m, 2H), 3.72-3.70 (m, 2H), 3.30-3.28 (m, 2H), 1.71-1.69 (m, 4H), 1.58-1.55 (m, 2H); ^{13}C {^{1}H} NMR (101 MHz, $CDCl_3$): δ_C 192.1, 165.6, 134.8, 133.4, 129.7, 129.1, 47.2, 42.3, 26.3, 25.3, 24.5; IR (υ, cm^{-1}, neat): 3008, 1672, 1649; HRMS (ESI): m/z calcd for $C_{13}H_{15}NO_2$: 218.1181 $[M+H]^+$; found: 218.1190

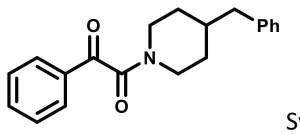

Synthesised according to general procedure C from 4-benzylpiperidine (3.5056 g, 20 mmol) to afford ketone **1.18** (5.2256 g, 85%) as a yellow oil. Ketone **1.18**: ^{1}H NMR (400 MHz, $CDCl_3$): δ_H 7.95-7.93 (m, 2H), 7.66-7.63 (m, 1H), 7.53-7.50 (m, 2H), 7.30-7.27 (m, 2H), 7.22-7.19 (m, 1H), 7.14-7.12 (m, 2H), 4.68-4.64 (m, 1H), 3.56-3.52 (m, 1H), 3.04-2.99 (m, 1H), 2.77-2.71 (m, 1H), 2.62-2.53 (m, 2H), 1.84-1.81 (m, 2H), 1.65-1.62 (m, 2H), 1.32-1.30 (m, 1H); ^{13}C {^{1}H} NMR (101 MHz, $CDCl_3$): δ_C 192.0, 165.5, 139.7, 134.8, 133.3, 129.7, 129.2, 129.1, 128.5, 126.3, 46.4, 42.9, 41.6, 38.3, 32.4, 31.7; IR (υ, cm^{-1}, neat): 3014, 1697, 1654; HRMS (ESI): m/z calcd for $C_{20}H_{21}NO_2$: 308.1651 $[M+H]^+$; found: 308.1664

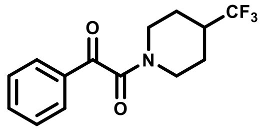

Synthesised according to general procedure C from 4-trifluoromethylpiperidine (3.0630 g, 20 mmol) to afford ketone **1.19** (3.8226 g, 67%) as a yellow oil. Ketone **1.19**: ^{1}H NMR (400 MHz, $CDCl_3$): δ_H 7.96-7.94 (m, 2H), 7.66-7.65 (m, 1H), 7.55-7.51 (m, 2H), 4.81-4.78 (m, 1H), 3.70-3.67 (m, 1H), 3.10-3.07 (m, 1H), 2.84-2.78 (m, 1H), 2.35-2.32 (m, 1H), 2.07-2.04 (m, 1H), 1.89-1.86 (m, 1H), 1.70-1.55 (m, 2H); ^{13}C {^{1}H} NMR (101 MHz, $CDCl_3$): δ_C 191.5, 165.6, 135.1, 133.1, 129.8, 129.3, 45.0, 40.5, 40.2, 25.1, 24.3; ^{19}F NMR (471 MHz, $CDCl_3$): δ -73.7; IR (υ, cm^{-1}, neat): 3038, 2963, 1687, 1645; HRMS (ESI): m/z calcd for $C_{14}H_{14}F_3NO_2$: 286.1055 $[M+H]^+$; found: 286.1077

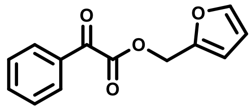

To a solution of benzoylformic acid (0.4999 g, 3.33 mmol) in CH_2Cl_2 (25 mL) was added furfuryl alcohol (0.2178 g, 2.22 mmol), *N*,*N*-dimethylaminopyridine (0.0269 g, 0.22 mmol), and dicyclohexylcarbodiimide (0.6871 g, 3.33 mmol). After stirring for 6 h, the solution was filtered, diluted with saturated aqueous $NaHCO_3$, and extracted with DCM (3×25 mL). The combined organic layers were dried with Na_2SO_4, and the solvent removed under reduced pressure. The crude oil was purified by silica gel flash chromatography to give ketone **1.20** (0.2147 g, 42%) as a yellow oil.[4] Ketone **1.20**: ^{1}H NMR (400 MHz, $CDCl_3$): δ_H 7.97-7.96 (m, 2H), 7.66-7.63 (m, 1H), 7.51-7.48 (m, 2H), 7.46-7.45 (m, 1H), 6.55-6.54 (m, 1H), 6.40-6.39 (m, 1H), 5.37 (s, 2H); ^{13}C {^{1}H} NMR (101 MHz, $CDCl_3$): δ_C 185.9, 163.5, 148.2, 144.0, 135.1, 134.4, 130.2, 129.0, 112.1, 110.9, 59.4; IR (υ, cm^{-1}, neat): 3006, 2866, 1731, 1695, 1473; HRMS (ESI): m/z calcd for $C_{13}H_{10}O_4$: 231.0739 $[M+H]^+$; found: 231.0744

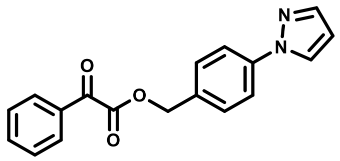

A 10 mL round bottomed flask was charged with Mukaiyama's reagent (2.1 mmol), carboxylic acid of choice (2.0 mmol), alcohol of choice (2.0 mmol), and dimethyl carbonate (4 mL). The suspension was stirred at room temperature and 2,6-lutidine was added (556 µL, 4.8 mmol). The reaction was then heated to 60 °C and stirred at room temperature for 24 hours. Reaction mixture was filtered and directly purified by silica gel flash chromatography (EtOAc/Hexane = 1/100) to afford the desired ketone **1.21** (0.3370 g, 55%) as a yellow oil.[5] Ketone **1.21**: ^{1}H NMR (400 MHz, $CDCl_3$): δ_H 7.95-7.90 (m, 3H), 7.70-7.68 (m, 3H), 7.60-7.56 (m, 1H), 7.49-7.41 (m, 4H), 6.42-6.41 (m, 1H), 5.38 (s, 2H); ^{13}C {^{1}H} NMR (101 MHz, $CDCl_3$): δ_C 186.0, 163.6, 141.4, 140.4, 135.1, 132.6, 132.3, 130.1, 130.0, 129.0, 128.0, 126.8, 119.2, 108.0, 67.2; IR (υ, cm^{-1}, neat): 3120, 1727, 1695; HRMS (ESI): m/z calcd for $C_{18}H_{14}N_2O_3$: 324.1359 $[M+NH_4]^+$; found: 324.1353

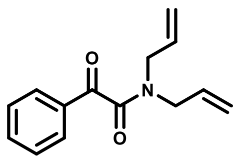

Thionyl chloride (0.96 mL, 13.32 mmol) was added dropwise to a stirred mixture of 2-oxo-2-phenylacetic acid (1.0 g, 6.66 mmol) and Et_3N (1.86 mL, 13.32 mmol) in DCM (10 mL) at 0 °C under nitrogen atmosphere. The stirring was continued for 20 min. Then a suspension of diallylamine (0.6471 g, 6.66 mmol) in

DCM (10 mL) was added slowly to the reaction mixture at 0 °C under nitrogen. The stirring was continued at room temperature and the completion of the reaction was monitored through TLC. A saturated aqueous solution of $NaHCO_3$ (10 mL) was added slowly under stirring to the reaction mixture. The organic layer was washed with water (3 × 15 mL) and the solvent removed under reduced pressure. The crude residue was purified by silica gel flash chromatography (petroleum ether/EtOAc = 20/1) to afford ketone **1.22** (1.2980 g, 85%) as a colourless liquid.[6] Ketone **1.22**: ^{1}H NMR (400 MHz, $CDCl_3$): δ_H 7.91-7.89 (m, 2H), 7.60-7.57 (m, 1H), 7.47-7.44 (m, 2H), 5.84-5.78 (m, 1H), 5.68-5.62 (m, 1H), 5.25-5.22 (m, 2H), 5.15-5.12 (m, 2H), 4.10-4.09 (m, 2H), 3.77-3.75 (m, 2H); ^{13}C {^{1}H} NMR (101 MHz, $CDCl_3$): δ_C 191.2, 166.9, 134.7, 133.1, 132.0, 131.7, 129.7, 129.0, 119.4, 118.6, 118.6, 49.4, 45.8, 45.8; IR (υ, cm^{-1}, neat): 2934, 2350, 1701, 1659; HRMS (ESI): m/z calcd for $C_{14}H_{15}NO_2$: 230.1181 $[M+H]^+$; found: 230.1197

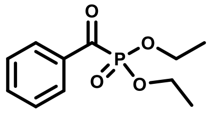

As reported in the literature, a Michaelis-Arbuzov reaction of benzoyl chloride (10.0 g, 71 mmol) with triethyl phosphite (11.8 g, 74 mmol), and distillation of the resulting mixture under reduced pressure gave ketone **1.23** (14.7893 g, 86%) as a yellow oil.[7] Ketone **1.23**: ^{1}H NMR (400 MHz, $CDCl_3$): δ_H 8.27-8.25 (m, 2H), 7.63-7.61 (m, 1H), 7.52-7.48 (m, 2H), 4.31-4.24 (m, 4H), 1.39-1.36 (m, 6H); ^{13}C {^{1}H} NMR (101 MHz, $CDCl_3$): δ_C 200.0, 198.3, 136.0, 135.4, 134.9, 130.0, 129.0, 64.2, 64.1, 16.5, 16.5; ^{31}P NMR (202 MHz, $CDCl_3$): δ -0.7; IR (υ, cm^{-1}, neat): 2986, 2898, 1671, 1582; HRMS (ESI): m/z calcd for $C_{11}H_{15}O_4P$: 243.0822 $[M+H]^+$; found: 243.0829

General Procedure D for the synthesis of ester/amide/ketone hydrazones[8]

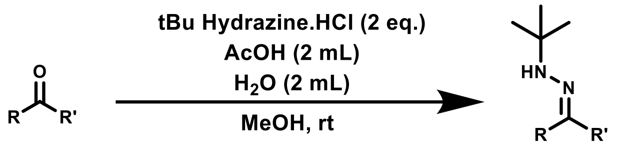

AcOH (2 mL) and H_2O (2 mL) were stirred and cooled to 0 °C before *tert*-Butylhydrazine hydrochloride (50 mmol, 6.2305 g, 2 equiv.) was added with continued stirring. The a-ketoester (25 mmol, 1 equiv.) was then added. The mixture was warmed to room temperature and MeOH (50 mL) was added to homogenize the solution. The mixture was stirred at room temperature overnight and quenched with H_2O upon completion. The reaction mixture was extracted with 3 x EtOAc, 1 x sat. aq. $NaHCO_3$, and 1 x brine. The combined organics were dried over $MgSO_4$ and the solvent removed under reduced pressure. The crude residue was purified by silica gel flash chromatography (Hexane/EtOAc = 9:1 to 7:3) to afford the desired hydrazone.

General Procedure E for the synthesis of aryl-CF_3 hydrazones[9]

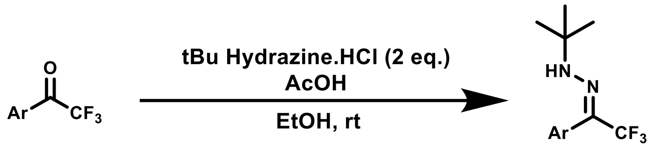

The ketone (25 mmol) was dissolved in EtOH (50 mL) at room temperature before *tert*-Butylhydrazine hydrochloride (50 mmol, 6.2305 g, 2 equiv.) was added and stirred for 5 min. A few drops of AcOH were then added and the mixture was refluxed overnight. The reaction was quenched by the addition of H_2O and extracted with 3 x EtOAc, 1 x sat. aq. $NaHCO_3$, and 1 x brine. The combined organics were dried over $MgSO_4$ and the solvent removed under reduced pressure. The crude residue was purified by silica gel flash chromatography (100% diethyl ether) to afford the desired hydrazone.

tBu-hydrazine (and its salts) still require standard hydrazine-class precautions; the advantage here is operational handling and avoidance of hydrazine hydrate.

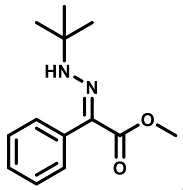

Synthesised according to general procedure D from methyl 2-oxo-2-phenylacetate (4.1040 g, 25 mmol) to afford hydrazone **2.1** (5.7404 g, 98%) as a colourless oil. Hydrazone **2.1**: ^{1}H NMR (400 MHz, $CDCl_3$): δ_H 7.56-7.54 (m, 2H), 7.34-7.31 (m, 2H), 7.26-7.23 (m, 1H), 3.79 (s, 3H), 1.33 (s, 9H); ^{13}C {^{1}H} NMR (101 MHz, $CDCl_3$): δ_C 164.2, 137.7, 128.3, 127.9, 126.7, 123.8, 55.3, 51.2, 28.9; IR (υ, cm^{-1}, neat): 3381, 3185, 2944, 1669, 1372; HRMS (ESI): m/z calcd for $C_{13}H_{18}N_2O_2$: 235.1447 $[M+H]^+$; found: 235.1458

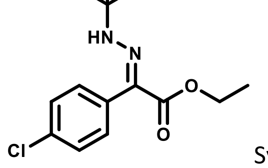

Synthesised according to general procedure D from ethyl 2-(4-chlorophenyl)-2-oxoacetate (5.3158 g, 25 mmol) to afford hydrazone **2.2** (6.5744 g, 93%) as a white solid. Hydrazone **2.2**: ^{1}H NMR (400 MHz, $CDCl_3$): δ_H 10.69 (s, 1H), 7.53-7.51 (m, 2H), 7.29-7.26 (m, 2H), 4.30-4.26 (q, J = 6.96 Hz, 2H), 1.35-1.32 (t, J = 7.15 Hz, 3H), 1.33 (s, 9H); ^{13}C {^{1}H} NMR (101 MHz, $CDCl_3$): δ_C 163.6, 136.3, 132.3, 129.4, 128.0, 127.9, 122.8, 60.4, 55.4, 28.9, 14.4; IR (υ, cm^{-1}, neat): 3380, 3178, 2940, 1674; HRMS (ESI): m/z calcd for $C_{14}H_{19}ClN_2O_2$: 283.1213 $[M+H]^+$; found: 283.1230

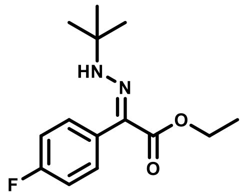 Synthesised according to general procedure D from ethyl 2-(4-fluorophenyl)-2-oxoacetate (4.9045 g, 25 mmol) to afford hydrazone **2.3** (4.1280 g, 62%) as a yellow oil. Hydrazone **2.3**: ^{1}H NMR (400 MHz, $CDCl_3$): δ_H 7.55-7.52 (m, 2H), 7.02-6.98 (m, 2H), 4.29-4.25 (q, J = 7.17 Hz, 2H), 1.34-1.31 (t, J = 7.10 Hz, 3H), 1.33 (s, 9H); ^{13}C {^{1}H} NMR (101 MHz, $CDCl_3$): δ_C 163.6, 162.9, 160.9, 133.9, 129.9, 129.8, 123.1, 114.5, 60.3, 55.2, 28.9, 14.4; ^{19}F NMR (471 MHz, $CDCl_3$): δ -116.4; IR (υ, cm^{-1}, neat): 3378, 3193, 2934, 1673; HRMS (ESI): m/z calcd for $C_{14}H_{19}FN_2O_2$: 267.1509 [M+H]$^+$; found: 267.1509

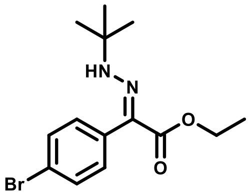 Synthesised according to general procedure D from ethyl 2-(4-bromophenyl)-2-oxoacetate (6.4270 g, 25 mmol) to afford hydrazone **2.4** (4.7447 g, 58%) as a yellow oil. Hydrazone **2.4**: ^{1}H NMR (400 MHz, $CDCl_3$): δ_H 10.70 (s, 1H), 7.47-7.42 (m, 4H), 4.29-4.25 (q, J = 7.07 Hz, 2H), 1.34-1.31 (t, J = 7.16 Hz, 3H), 1.33 (s, 9H); ^{13}C {^{1}H} NMR (101 MHz, $CDCl_3$): δ_C 163.5, 136.8, 130.9, 129.8, 122.8, 120.5, 60.4, 55.4, 28.9, 14.4; IR (υ, cm^{-1}, neat): 3376, 2941, 1668, 1354; HRMS (ESI): m/z calcd for $C_{14}H_{19}BrN_2O_2$: 327.0708 [M+H]$^+$; found: 327.0707

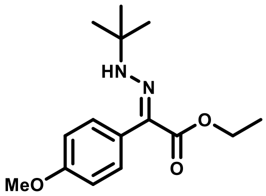 Synthesised according to general procedure C from ethyl 2-(4-methoxyphenyl)-2-oxoacetate (5.2053 g, 25 mmol) to afford hydrazone **2.5** (5.7492 g, 87%) as a yellow oil. Hydrazone **2.5**: ^{1}H NMR (400 MHz, $CDCl_3$): δ_H 10.27 (s, 1H), 7.30-7.28 (m, 2H), 6.68-6.66 (m, 2H), 4.08-4.04 (q, J = 7.12 Hz, 2H), 3.58 (s, 3H), 1.14-1.11 (t, J = 7.18 Hz, 3H), 1.11 (s, 9H); ^{13}C {^{1}H} NMR (101 MHz, $CDCl_3$): δ_C 163.8, 158.5, 130.6, 129.5, 124.0, 113.3, 60.2, 55.4, 51.2, 28.9, 14.4; IR (υ, cm^{-1}, neat): 3388, 2938, 1671, 1365; HRMS (ESI): m/z calcd for $C_{14}H_{20}N_2O_3$: 279.1709 [M+H]$^+$; found: 279.1736

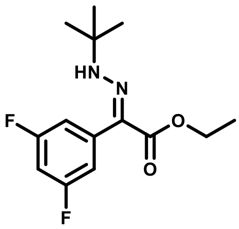 Synthesised according to general procedure D from ethyl 2-(3,5-difluorophenyl)-2-oxoacetate (5.3543 g, 25 mmol) to afford hydrazone **2.6** (5.4730 g, 77%) as a yellow oil. Hydrazone **2.6**: ^{1}H NMR (400 MHz, $CDCl_3$): δ_H 10.87 (s, 1H), 7.19-7.16 (m, 2H), 6.67-6.63 (m, 1H), 4.33-4.28 (q, J = 7.19 Hz, 2H), 1.38-1.35 (t, J = 7.10 Hz, 3H), 1.35 (s, 9H); ^{13}C {^{1}H} NMR (101 MHz, $CDCl_3$): δ_C 163.7, 163.6, 163.4, 161.7, 161.6, 141.0, 140.9, 140.8, 121.3, 110.5, 110.5, 110.4, 110.3, 101.7, 101.5, 101.3, 60.6, 55.8, 28.9, 14.4; ^{19}F NMR (471 MHz, $CDCl_3$): δ -111.5; IR (υ, cm^{-1}, neat): 3372, 3167, 2934, 1674, 1351; HRMS (ESI): m/z calcd for $C_{14}H_{18}F_2N_2O_2$: 285.1415 [M+H]$^+$; found: 285.1413

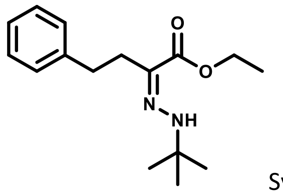 Synthesised according to general procedure D from ethyl 2-oxo-4-phenylbutanoate (5.1560 g, 25 mmol) to afford hydrazone **2.7** (6.2876 g, 91%) as a yellow oil. Hydrazone **2.7**: ^{1}H NMR (400 MHz,

$CDCl_3$): δ_H 10.02 (s, 1H), 7.29-7.25 (m, 2H), 7.22-7.20 (m, 2H), 7.19-7.16 (m, 1H), 4.20-4.16 (q, J = 7.23 Hz, 2H), 2.86-2.83 (t, J = 7.23 Hz, 2H), 2.72-2.69 (t, J = 7.23 Hz, 2H), 1.32-1.29 (t, J = 7.23 Hz, 3H), 1.22 (s, 9H); $^{13}C\ \{^1H\}$ NMR (101 MHz, $CDCl_3$): δ_C 163.7, 142.6, 128.7, 125.7, 124.0, 59.8, 54.3, 50.9, 34.7, 34.4, 28.8, 14.5; IR (υ, cm^{-1}, neat): 3406, 2940, 1668, 1349; HRMS (ESI): m/z calcd for $C_{16}H_{24}N_2O_2$: 277.1916 $[M+H]^+$; found: 277.1920

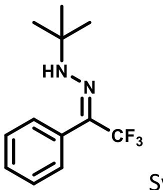

Synthesised according to general procedure E from 2,2,2-trifluoro-1-phenylethan-1-one (4.3530 g, 25 mmol) to afford hydrazone **2.8** (4.3356 g, 71%) as a yellow oil. Hydrazone **2.8**: ^1H NMR (400 MHz, $CDCl_3$): δ_H 7.52-7.44 (m, 3H), 7.35-7.33 (m, 2H), 1.19 (s, 9H); $^{13}C\ \{^1H\}$ NMR (101 MHz, $CDCl_3$): δ_C 135.7, 129.9, 129.6, 129.1, 128.2, 123.1, 120.4, 54.5, 28.6; ^{19}F NMR (471 MHz, $CDCl_3$): δ -65.9; IR (υ, cm^{-1}, neat): 3370, 3186; HRMS (ESI): m/z calcd for $C_{12}H_{15}F_3N_2$: 245.1415 $[M+H]^+$; found: 245.1413

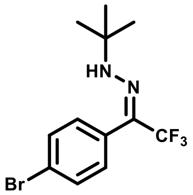

Synthesised according to general procedure E from 1-(4-bromophenyl)-2,2,2-trifluoroethan-1-one (6.3255 g, 25 mmol) to afford hydrazone **2.9** (5.4937 g, 68%) as a yellow oil. Hydrazone **2.9**: ^1H NMR (400 MHz, $CDCl_3$): δ_H 7.66-7.62 (m, 2H), 7.24-7.21 (m, 2H), 1.19 (s, 9H); $^{13}C\ \{^1H\}$ NMR (101 MHz, $CDCl_3$): δ_C 133.0, 130.9, 128.8, 128.4, 128.1, 127.7, 127.0, 125.6, 124.4, 122.9, 120.2, 117.5, 54.7, 28.6; ^{19}F NMR (471 MHz, $CDCl_3$): δ -65.8; IR (υ, cm^{-1}, neat): 3379, 3186; HRMS (ESI): m/z calcd for $C_{12}H_{14}BrF_3N_2$: 323.0371 $[M+H]^+$; found: 323.0370

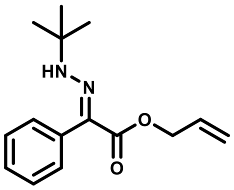

Synthesised according to general procedure D from allyl 2-oxo-2-phenylacetate (4.7550 g, 25 mmol) to afford hydrazone **2.10** (3.3193 g, 51%) as a yellow oil. Hydrazone **2.10**: ^1H NMR (400 MHz, $CDCl_3$): δ_H 7.59-7.57 (m, 2H), 7.34-7.31 (m, 2H), 7.26-7.22 (m, 1H), 5.98-5.94 (m, 1H), 5.36-5.31 (m, 1H), 5.26-5.23 (m, 1H), 4.73-4.71 (m, 2H), 1.34 (s, 9H); $^{13}C\ \{^1H\}$ NMR (101 MHz, $CDCl_3$): δ_C 163.4, 137.8, 132.2, 128.3, 127.8, 126.7, 123.7, 118.3, 64.9, 55.4, 28.9; IR (υ, cm^{-1}, neat): 3383, 3189, 2984, 2947, 1673; HRMS (ESI): m/z calcd for $C_{15}H_{20}N_2O_2$: 261.1603 $[M+H]^+$; found: 261.1601

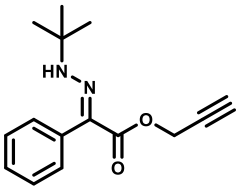

Synthesised according to general procedure D from prop-2-yn-1-yl 2-oxo-2-phenylacetate (4.7045 g, 25 mmol) to afford hydrazone **2.11** (3.9394 g, 61%) as a yellow oil. Hydrazone **2.11**: ^1H NMR (400 MHz, $CDCl_3$): δ_H 10.67 (s, 1H), 7.59-7.56 (m, 2H), 7.36-7.32 (m, 2H), 7.27-7.23 (m, 1H), 4.80 (d, J = 2.53 Hz, 2H), 2.49 (t, J = 2.42 Hz, 1H), 1.34 (s, 9H); $^{13}C\ \{^1H\}$ NMR (101 MHz, $CDCl_3$): δ_C 162.7, 137.4, 128.4, 127.9, 126.8, 122.9, 78.0, 75.0, 55.6, 51.4, 28.9; IR (υ, cm^{-1}, neat): 3492, 3373, 3181, 2947, 1670; HRMS (ESI): m/z calcd for $C_{15}H_{18}N_2O_2$: 259.1447 $[M+H]^+$; found: 259.1450

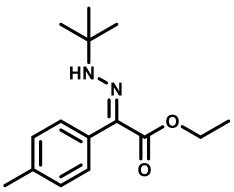

Synthesised according to general procedure D from ethyl 2-oxo-2-(p-tolyl)acetate (4.8053 g, 25 mmol) to afford hydrazone **2.12** (4.2632 g, 65%) as a yellow oil. Hydrazone **2.12**: ^1H NMR (400 MHz, $CDCl_3$):

δ_H 10.54 (s, 1H), 7.48-7.43 (m, 2H), 7.15-7.13 (m, 2H), 4.30-4.25 (q, J = 7.16 Hz, 2H), 2.36 (s, 3H), 1.35-1.32 (t, J = 7.11 Hz, 3H), 1.33 (s, 9H); ^{13}C {^{1}H} NMR (101 MHz, $CDCl_3$): δ_C 163.9, 136.3, 135.1, 128.5, 128.2, 124.2, 60.2, 55.1, 28.9, 21.3, 14.4; IR (υ, cm^{-1}, neat): 3374, 2978, 2940, 1667; HRMS (ESI): m/z calcd for $C_{15}H_{22}N_2O_2$: 263.1760 $[M+H]^+$; found: 263.1765

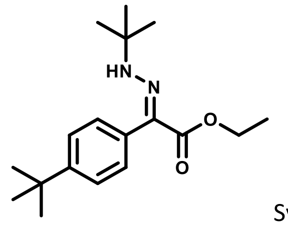
Synthesised according to general procedure D from ethyl 2-(4-(tert-butyl)phenyl)-2-oxoacetate (5.8575 g, 25 mmol) to afford hydrazone **2.13** (6.7736 g, 89%) as a yellow oil. Hydrazone **2.13**: ^{1}H NMR (400 MHz, $CDCl_3$): δ_H 10.56 (s, 1H), 7.54-7.52 (m, 2H), 7.36-7.34 (m, 2H), 4.31-4.26 (q, J = 7.10 Hz, 2H), 1.36-1.34 (t, J = 7.16 Hz, 3H), 1.34 (s, 9H), 1.33 (s, 9H); ^{13}C {^{1}H} NMR (101 MHz, $CDCl_3$): δ_C 163.9, 149.4, 135.0, 127.9, 124.8, 124.0, 60.2, 55.1, 34.6, 31.5, 29.0, 14.5; IR (υ, cm^{-1}, neat): 3378, 2977, 2959, 2942, 1670; HRMS (ESI): m/z calcd for $C_{18}H_{28}N_2O_2$: 305.2229 $[M+H]^+$; found: 305.2246

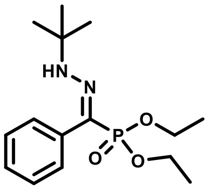
Synthesised according to general procedure D from diethyl benzoylphosphonate (6.0553 g, 25 mmol) to afford hydrazone **2.14** (7.5745 g, 97%) as a yellow oil. Hydrazone **2.14**: ^{1}H NMR (400 MHz, $CDCl_3$): δ_H 9.99 (s, 1H), 7.67-7.65 (m, 2H), 7.29-7.26 (m, 2H), 7.18-7.15 (m, 1H), 4.13-4.05 (m, 2H), 4.01-3.93 (m, 2H), 1.31 (s, 9H), 1.26-1.23 (t, J = 7.20 Hz, 6H); ^{13}C {^{1}H} NMR (101 MHz, $CDCl_3$): δ_C 138.1, 137.8, 127.9, 126.3, 125.4, 125.4, 121.9, 120.7, 61.8, 61.8, 54.6, 28.7, 16.1, 16.0; ^{31}P NMR (202 MHz, $CDCl_3$): δ 12.45; IR (υ, cm^{-1}, neat): 3364, 3178, 2944, 2907; HRMS (ESI): m/z calcd for $C_{14}H_{23}N_2O_3P$: 313.1681 $[M+H]^+$; found: 313.1684

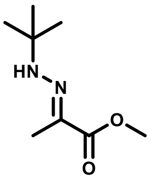
Synthesised according to general procedure D from methyl 2-oxopropanoate (2.5523 g, 25 mmol) to afford hydrazone **2.15** (4.0043 g, 93%) as a yellow oil. Hydrazone **2.15**: ^{1}H NMR (400 MHz, $CDCl_3$): δ_H 3.63 (s, 3H), 1.19 (s, 3H), 1.18 (s, 9H); ^{13}C {^{1}H} NMR (101 MHz, $CDCl_3$): δ_C 174.0, 81.2, 67.9, 51.3, 26.6, 17.3; IR (υ, cm^{-1}, neat): 3378, 3015, 2938, 1668, 1370; HRMS (ESI): m/z calcd for $C_8H_{16}N_2O_2$: 173.1290 $[M+H]^+$; found: 173.1304

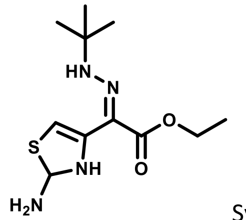
Synthesised according to general procedure D from ethyl 2-(2-amino-2,3-dihydrothiazol-4-yl)-2-oxoacetate (5.0558 g, 25 mmol) to afford hydrazone **2.16** (4.0174 g, 59%) as a yellow oil. Hydrazone **2.16**: ^{1}H NMR (400 MHz, $CDCl_3$): δ_H 10.68 (s, 1H), 5.47 (s, 1H), 4.26-4.22 (q, J = 7.05 Hz, 2H), 2.02 (s, 1H), 1.33-1.30 (t, J = 7.02 Hz, 3H), 1.29 (s, 2H), 1.28 (s, 9H); ^{13}C {^{1}H} NMR (101 MHz, $CDCl_3$): δ_C 166.2, 165.7, 143.6, 120.5, 108.1, 60.3, 54.8, 28.7, 14.3; IR (υ, cm^{-1}, neat): 3384, 3350, 3145, 2942, 1670; HRMS (ESI): m/z calcd for $C_{11}H_{20}N_4O_2S$: 271.1229 $[M-H]^+$; found: 271.1270

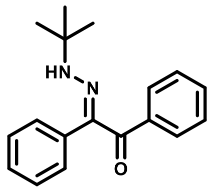

Synthesised according to general procedure D from benzil (5.2558 g, 25 mmol) to afford hydrazone **2.17** (6.2382 g, 89%) as a . Hydrazone **2.17**: ^{1}H NMR (400 MHz, CDCl$_3$): δ_H 7.98-7.96 (m, 2H), 7.54-7.51 (m, 2H), 7.49-7.47 (m, 1H), 7.44-7.41 (m, 3H), 7.31-7.29 (m, 2H), 1.21 (s, 9H); ^{13}C {^{1}H} NMR (101 MHz, CDCl$_3$): δ_C 191.2, 140.2, 139.1, 131.0, 130.8, 130.5, 129.4, 129.0, 127.5, 55.4, 28.8; IR (υ, cm^{-1}, neat): 3369, 3183, 2982, 1683; HRMS (ESI): m/z calcd for $C_{18}H_{20}N_2O$: 281.1654 [M+H]$^+$; found: 281.1693

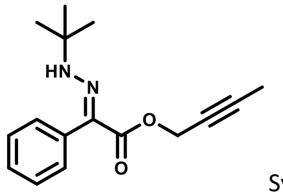

Synthesised according to general procedure D from but-2-yn-1-yl 2-oxo-2-phenylacetate (5.0553 g, 25 mmol) to afford hydrazone **2.18** (6.4002 g, 94%) as a colourless liquid. Hydrazone **2.18**: ^{1}H NMR (400 MHz, CDCl$_3$): δ_H 10.79 (s, 1H), 7.70-7.67 (m, 2H), 7.44-7.39 (m, 2H), 7.34-7.30 (m, 1H), 4.85-4.83 (q, J = 2.49 Hz, 2H), 1.90 (t, J = 2.49 Hz, 3H), 1.43 (s, 9H); ^{13}C {^{1}H} NMR (101 MHz, CDCl$_3$): δ_C 162.8, 137.4, 128.2, 128.1, 127.6, 127.6, 126.5, 123.2, 82.9, 73.4, 55.2, 52.0, 28.7, 3.6; IR (υ, cm^{-1}, neat): 3376, 3178, 2947, 1671; HRMS (ESI): m/z calcd for $C_{16}H_{20}N_2O_2$: 273.1603 [M+H]$^+$; found: 273.1641

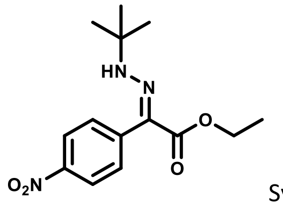

Synthesised according to general procedure D from ethyl 2-(4-nitrophenyl)-2-oxoacetate (5.5795 g, 25 mmol) to afford hydrazone **2.19** (5.4998 g, 75%) as a yellow oil. Hydrazone **2.19**: ^{1}H NMR (400 MHz, CDCl$_3$): δ_H 11.05 (s, 1H), 8.12-8.09 (m, 2H), 7.77-7.72 (m, 2H), 4.30-4.26 (q, J = 7.20 Hz, 2H), 1.33 (s, 9H), 1.33-1.31 (t, J = 7.12 Hz, 3H); ^{13}C {^{1}H} NMR (101 MHz, CDCl$_3$): δ_C 163.2, 145.7, 144.1, 127.8, 123.0, 121.3, 60.5, 55.9, 28.7, 14.2; IR (υ, cm^{-1}, neat): 3374, 3189, 2936, 1674, 1472; HRMS (ESI): m/z calcd for $C_{14}H_{19}N_3O_4$: 294.1454 [M+H]$^+$; found: 294.1472

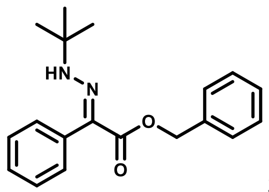

Synthesised according to general procedure D from benzyl 2-oxo-2-phenylacetate (6.0065 g, 25 mmol) to afford hydrazone **2.20** (7.1392 g, 92%) as a yellow oil. Hydrazone **2.20**: ^{1}H NMR (400 MHz, CDCl$_3$): δ_H 7.48-7.45 (m, 2H), 7.40-7.31 (m, 5H), 7.29-7.25 (m, 3H), 5.26 (s, 2H), 1.24 (s, 9H); ^{13}C {^{1}H} NMR (101 MHz, CDCl$_3$): δ_C 164.7, 136.9, 131.3, 130.9, 129.3, 129.0, 128.5, 127.8, 127.7, 66.1, 55.1, 28.9; IR (υ, cm^{-1}, neat): 3374, 3189, 2950, 1674; HRMS (ESI): m/z calcd for $C_{19}H_{22}N_2O_2$: 311.1760 [M+H]$^+$; found: 311.1798

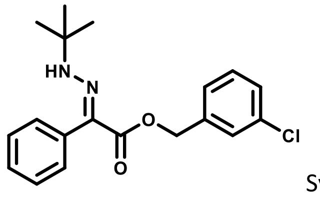

Synthesised according to general procedure D from 3-chlorobenzyl 2-oxo-2-phenylacetate (6.8675 g, 25 mmol) to afford hydrazone **2.21** (7.3279 g, 85%) as a yellow oil. Hydrazone **2.21**: ^{1}H NMR (400 MHz, CDCl$_3$): δ_H 7.45-7.42 (m, 2H), 7.37-7.32 (m, 2H), 7.23-7.19 (m, 5H), 5.18 (s, 2H), 1.21 (s, 9H); ^{13}C {^{1}H} NMR (101 MHz, CDCl$_3$): δ_C 164.5, 139.0, 134.4, 131.0, 130.8, 129.8, 129.4, 129.3, 129.1, 127.9, 127.7, 125.6, 65.1, 55.2, 28.9; IR (υ, cm^{-1}, neat): 3377, 3190, 2951, 1675; HRMS (ESI): m/z calcd for $C_{19}H_{21}ClN_2O_2$: 345.1370 [M+H]$^+$; found: 345.1400

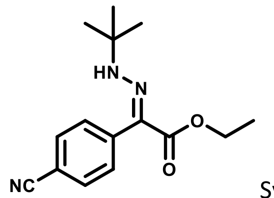

Synthesised according to general procedure D from ethyl 2-(4-cyanophenyl)-2-oxoacetate (5.0800 g, 25 mmol) to afford hydrazone **2.22** (4.1001 g, 60%) as a colourless oil. Hydrazone **2.22**: ^{1}H NMR (400 MHz, $CDCl_3$): δ_H 10.72 (s, 1H), 7.46-7.44 (m, 2H), 7.28-7.26 (m, 2H), 4.03-3.99 (q, J = 7.06 Hz, 2H), 1.06 (s, 9H), 1.06-1.04 (t, J = 7.16 Hz, 3H); ^{13}C {^{1}H} NMR (101 MHz, $CDCl_3$): δ_C 162.9, 141.9, 131.1, 127.8, 121.4, 119.0, 109.0, 60.2, 55.4, 28.5, 14.0; IR (υ, cm^{-1}, neat): 3372, 3189, 2943, 2257, 1671; HRMS (ESI): m/z calcd for $C_{15}H_{19}N_3O_2$: 274.1556 $[M+H]^+$; found: 274.1567

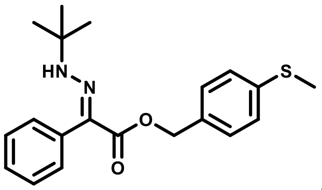

Synthesised according to general procedure D from 4-(methylthio)benzyl 2-oxo-2-phenylacetate (7.1585 g, 25 mmol) to afford hydrazone **2.23** (6.9514 g, 78%) as a yellow oil. Hydrazone **2.23**: ^{1}H NMR (400 MHz, $CDCl_3$): δ_H 7.61-7.59 (m, 2H), 7.35-7.31 (m, 4H), 7.27-7.25 (m, 3H), 5.24 (s, 2H), 2.49 (s, 3H), 1.36 (s, 9H); ^{13}C {^{1}H} NMR (101 MHz, $CDCl_3$): δ_C 163.3, 138.7, 137.6, 132.7, 128.8, 128.3, 127.7, 126.6, 126.5, 123.5, 65.4, 55.3, 28.9, 15.7; IR (υ, cm^{-1}, neat): 3379, 3186, 2955, 1677; HRMS (ESI): m/z calcd for $C_{20}H_{24}N_2O_2S$: 357.1637 $[M+H]^+$; found: 357.1647

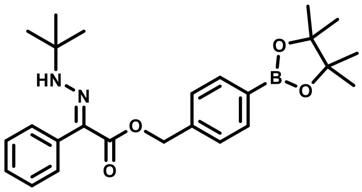

Synthesised according to general procedure D from 4-(4,4,5,5-tetramethyl-1,3,2-dioxaborolan-2-yl)benzyl 2-oxo-2-phenylacetate (9.1555 g, 25 mmol) to afford hydrazone **2.24** (10.2545 g, 94%) as a yellow oil. Hydrazone **2.24**: ^{1}H NMR (400 MHz, $CDCl_3$): δ_H 10.60 (s, 1H), 7.77-7.86 (m, 2H), 7.54-7.52 (m, 2H), 7.33-7.31 (m, 2H), 7.27-7.24 (m, 2H), 7.19-7.16 (m, 1H), 5.22 (s, 2H), 1.29 (s, 12H), 1.28 (s, 9H); ^{13}C {^{1}H} NMR (101 MHz, $CDCl_3$): δ_C 163.3, 139.1, 137.6, 135.1, 128.3, 127.7, 127.1, 126.6, 123.5, 83.9, 65.7, 55.3, 28.9, 24.9; IR (υ, cm^{-1}, neat): 3379, 3185, 2953, 1673; HRMS (ESI): m/z calcd for $C_{25}H_{33}BN_2O_4$: 437.2612 $[M+H]^+$; found: 437.2625

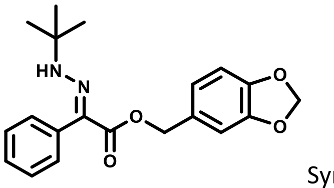

Synthesised according to general procedure D from benzo[d][1,3]dioxol-5-ylmethyl 2-oxo-2-phenylacetate (7.1068 g, 25 mmol) to afford hydrazone **2.25** (3.6327 g, 41%) as a white powder. Hydrazone **2.25**: ^{1}H NMR (400 MHz, $CDCl_3$): δ_H 10.62 (s, 1H), 7.53-7.51 (m, 2H), 7.26-7.23 (m, 2H), 7.17-7.14 (m, 1H), 6.80-6.76 (m, 2H), 6.70-6.67 (m, 1H), 5.80 (s, 2H), 5.08 (s, 2H), 1.27 (s, 9H); ^{13}C {^{1}H} NMR (101 MHz, $CDCl_3$): δ_C 163.3, 147.8, 147.6, 137.6, 129.7, 128.2, 127.7, 126.5, 123.6, 122.0, 108.9, 108.2, 101.1, 65.7, 55.2, 28.8; IR (υ, cm^{-1}, neat): 3388, 3173, 2948, 2933, 1676; HRMS (ESI): m/z calcd for $C_{20}H_{22}N_2O_4$: 355.1658 $[M+H]^+$; found: 355.1678

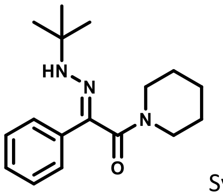

Synthesised according to general procedure D from 1-phenyl-2-(piperidin-1-yl)ethane-1,2-dione (5.4318 g, 25 mmol) to afford hydrazone **2.26** (5.9638 g, 83%) as a yellow oil. Hydrazone **2.26**: ^{1}H NMR (400 MHz, $CDCl_3$): δ_H 7.59-7.56 (m, 2H), 7.35-7.31 (m, 2H), 7.28-7.24 (m, 1H), 3.76-3,73 (t, J = 5.53 Hz, 2H), 3.27-

3.24 (t, J = 5.66 Hz, 2H), 1.66-1.64 (m, 4H), 1.48-1.45 (m, 2H), 1.26 (s, 9H); $^{13}C\ \{^1H\}$ NMR (101 MHz, CDCl3): δ_C 164.6, 139.6, 135.0, 128.6, 128.0, 124.9, 54.3, 47.2, 42.0, 28.9, 26.8, 26.0, 24.6; IR (υ, cm^{-1}, neat): 3370, 3178, 2984, 1663; HRMS (ESI): m/z calcd for $C_{17}H_{25}N_3O$: 288.2104 $[M+H]^+$; found: 288.2116

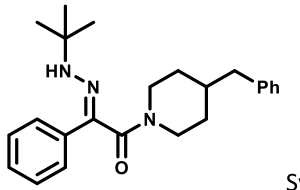

Synthesised according to general procedure D from 1-(4-benzylpiperidin-1-yl)-2-phenylethane-1,2-dione (7.6848 g, 25 mmol) to afford hydrazone **2.27** (7.6449 g, 81%) as a yellow oil. Hydrazone **2.27**: ^1H NMR (400 MHz, CDCl3): δ_H 7.57-7.55 (m, 2H), 7.35-7.31 (m, 2H), 7.30-7.25 (m, 3H), 7.22-7.18 (m, 1H), 7.13-7.11 (m, 2H), 4.76-4.72 (m, 1H), 3.61-3.58 (m, 1H), 2.93-2.87 (m, 1H), 2.75-2.70 (m, 1H), 2.55 (d, J = 7.50 Hz, 2H), 1.82-1.76 (m, 2H), 1.59-1.56 (m, 1H), 1.25 (s, 9H), 1.16-1.08 (m, 1H), 0.93-0.84 (m, 1H); $^{13}C\ \{^1H\}$ NMR (101 MHz, CDCl3): δ_C 164.7, 139.8, 139.5, 135.0, 129.2, 128.6, 128.4, 128.0, 126.2, 124.9, 54.4, 46.5, 42.9, 41.3, 38.2, 32.9, 32.0, 28.9; IR (υ, cm^{-1}, neat): 3373, 3164, 2988, 1668; HRMS (ESI): m/z calcd for $C_{24}H_{31}N_3O$: 378.2545 $[M+H]^+$; found: 378.2559

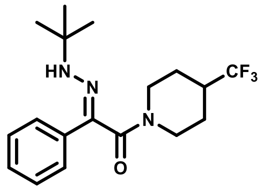

Synthesised according to general procedure D from 1-phenyl-2-(4-(trifluoromethyl)piperidin-1-yl)ethane-1,2-dione (7.1318 g, 25 mmol) to afford hydrazone **2.28** (3.3764 g, 38%) as a yellow oil. Hydrazone **2.28**: ^1H NMR (400 MHz, CDCl3): δ_H 7.55-7.53 (m, 2H), 7.36-7.32 (m, 2H), 7.29-7.26 (m, 1H), 4.88 (d, J = 13.49 Hz, 1H), 3.71 (d, J = 13.49 Hz, 1H), 2.95 (t, J = 13.49 Hz, 1H), 2.77 (t, J = 13.49 Hz, 1H), 2.28-2.25 (m, 1H), 2.04 (d, J = 13.49 Hz, 1H), 1.78 (d, J = 13.49 Hz, 1H), 1.57-1.48 (m, 1H), 1.25 (s, 9H), 0.91-0.86 (m, 1H); $^{13}C\ \{^1H\}$ NMR (101 MHz, CDCl3): δ_C 165.1, 139.4, 134.7, 128.8, 128.3, 124.8, 54.5, 45.2, 40.0, 31.6, 30.3, 28.8, 24.7; ^{19}F NMR (471 MHz, CDCl3): δ -73.7; IR (υ, cm^{-1}, neat): 3374, 3180, 2879, 1671; HRMS (ESI): m/z calcd for $C_{18}H_{24}F_3N_3O$: 356.2003 $[M+H]^+$; found: 356.2006

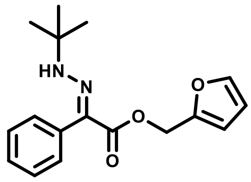

Synthesised according to general procedure D from furan-2-ylmethyl 2-oxo-2-phenylacetate (5.7555 g, 25 mmol) to afford hydrazone **2.29** (6.6079 g, 88%) as a yellow oil. Hydrazone **2.29**: ^1H NMR (400 MHz, CDCl3): δ_H 10.76 (s, 1H), 7.70-7.68 (m, 2H), 7.50 (m, 1H), 7.42-7.39 (m, 2H), 7.34-7.30 (m, 1H), 6.52-6.51 (m, 1H), 6.43-6.42 (m, 1H), 5.31 (s, 2H), 1.45 (s, 9H); $^{13}C\ \{^1H\}$ NMR (101 MHz, CDCl3): δ_C 163.0, 149.5, 143.2, 137.5, 128.1, 127.7, 126.5, 123.4, 110.6, 110.5, 57.4, 55.2, 28.8; IR (υ, cm^{-1}, neat): 3373, 3188, 3062, 2991, 2950, 1676; HRMS (ESI): m/z calcd for $C_{17}H_{20}N_2O_3$: 301.1652 $[M+H]^+$; found: 301.1661

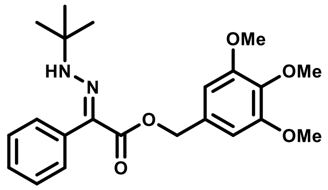

Synthesised according to general procedure D from 3,4,5-trimethoxybenzyl 2-oxo-2-phenylacetate (8.2585 g, 25 mmol) to afford hydrazone **2.30** (4.5054 g, 45%) as a yellow oil. Hydrazone **2.30**: ^1H NMR (400 MHz, CDCl3): δ_H 10.58 (s, 1H), 7.53-7.51 (m, 2H), 7.26-7.23 (m, 2H), 7.18-7.15 (s, 1H), 6.50 (s, 2H), 5.13 (s, 2H), 3.77 (s, 3H), 3.74 (s, 6H), 1.27 (s, 9H); $^{13}C\ \{^1H\}$ NMR (101 MHz, CDCl3): δ_C 163.2, 153.3, 137.7, 137.6, 131.6, 128.3, 127.7, 126.6, 123.6, 104.5, 65.6, 60.9, 56.1, 55.3, 28.8; IR (υ, cm^{-1}, neat): 3384, 3184, 2954, 1677, 1370; HRMS (ESI): m/z calcd for $C_{22}H_{28}N_2O_5$: 401.2076 $[M+H]^+$; found: 401.2086

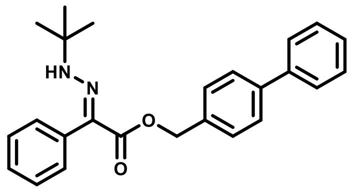

Synthesised according to general procedure D from [1,1'-biphenyl]-4-ylmethyl 2-oxo-2-phenylacetate (7.9090 g, 25 mmol) to afford hydrazone **2.31** (8.6960 g, 90%) as a yellow oil. Hydrazone **2.31**: ^{1}H NMR (400 MHz, $CDCl_3$): δ_H 10.56 (s, 1H), 7.51-7.49 (m, 2H), 7.46-7.42 (m, 4H), 7.31-7.26 (m, 4H), 7.22-7.18 (m, 3H), 7.13-7.08 (m, 1H), 5.16 (s, 2H), 1.21 (s, 9H); ^{13}C {^{1}H} NMR (101 MHz, $CDCl_3$): δ_C 163.4, 141.1, 140.6, 137.6, 135.0, 128.9, 128.5, 128.3, 127.8, 127.5, 127.3, 127.1, 126.6, 123.6, 65.5, 55.3, 28.9; IR (υ, cm^{-1}, neat): 3374, 3187, 2951, 1675; HRMS (ESI): m/z calcd for $C_{25}H_{26}N_2O_2$: 387.2073 [M+H]$^+$; found: 387.2082

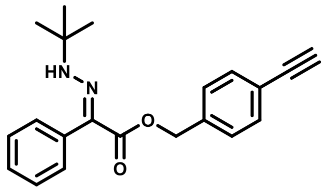

Synthesised according to general procedure D from 4-ethynylbenzyl 2-oxo-2-phenylacetate (6.6070 g, 25 mmol) to afford hydrazone **2.32** (6.1032 g, 73%) as a colourless liquid. Hydrazone **2.32**: ^{1}H NMR (400 MHz, $CDCl_3$): δ_H 10.54 (s, 1H), 7.45-7.43 (m, 2H), 7.33-7.30 (m, 2H), 7.17-7.13 (m, 4H), 7.10-7.06 (m, 1H), 5.08 (s, 2H), 2.92 (s, 1H), 1.19 (s, 9H); ^{13}C {^{1}H} NMR (101 MHz, $CDCl_3$): δ_C 163.1, 137.5, 136.7, 132.3, 128.2, 127.8, 127.7, 126.6, 123.3, 121.9, 83.3, 77.8, 65.2, 55.3, 28.8; IR (υ, cm^{-1}, neat): 3491, 3371, 3187, 2948, 1675; HRMS (ESI): m/z calcd for $C_{21}H_{22}N_2O_2$: 335.1760 [M+H]$^+$; found: 335.1770

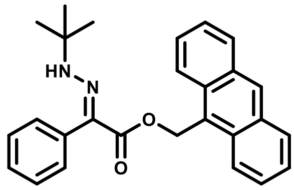

Synthesised according to general procedure D from anthracen-9-ylmethyl 2-oxo-2-phenylacetate (8.5095 g, 25 mmol) to afford hydrazone **2.33** (3.5921 g, 35%) as a colourless liquid. Hydrazone **2.33**: ^{1}H NMR (400 MHz, $CDCl_3$): δ_H 10.43 (s, 1H), 8.25-8.23 (m, 3H), 7.81-7.77 (m, 2H), 7.40-7.36 (m, 2H), 7.30-7.27 (m, 4H), 6.99-6.95 (m, 3H), 6.09 (s, 2H), 1.15 (s, 9H); ^{13}C {^{1}H} NMR (101 MHz, $CDCl_3$): δ_C 163.4, 137.5, 131.4, 131.1, 129.3, 129.1, 128.1, 127.6, 126.6, 126.4, 126.3, 126.3, 125.1, 125.0, 124.3, 123.7, 66.6, 58.4, 55.3, 28.9; IR (υ, cm^{-1}, neat): 3374, 3184, 3045, 2961, 1673; HRMS (ESI): m/z calcd for $C_{27}H_{26}N_2O_2$: 411.2073 [M+H]$^+$; found: 411.2070

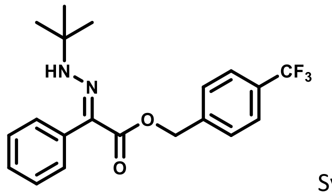

Synthesised according to general procedure D from 4-(trifluoromethyl)benzyl 2-oxo-2-phenylacetate (7.7065 g, 25 mmol) to afford hydrazone **2.34** (4.3516 g, 46%) as a yellow oil. Hydrazone **2.34**: ^{1}H NMR (400 MHz, $CDCl_3$): δ_H 10.81 (s, 1H), 7.66 (d, J = 8.03 Hz, 2H), 7.62 (d, J = 8.03 Hz, 2H), 7.45 (d, J = 8.03 Hz, 2H), 7.37 (t, J = 8.03 Hz, 2H), 7.28 (t, J = 8.03 Hz, 1H), 5.30 (s, 2H), 1.39 (s, 9H); ^{13}C {^{1}H} NMR (101 MHz, $CDCl_3$): δ_C 163.1, 140.1, 137.6, 130.7, 130.4, 130.1, 129.7, 128.3, 127.9, 127.8, 126.7, 125.5, 123.2, 64.8, 55.4, 28.8; ^{19}F NMR (471 MHz, $CDCl_3$): δ -62.3; IR (υ, cm^{-1}, neat): 3379, 3188, 3147, 2946, 1671; HRMS (ESI): m/z calcd for $C_{20}H_{21}F_3N_2O_2$: 379.1633 [M+H]$^+$; found: 379.1638

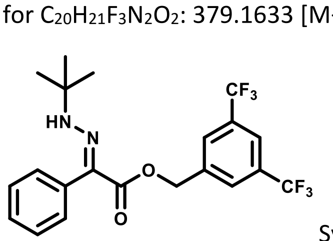

Synthesised according to general procedure D from 3,5-bis(trifluoromethyl)benzyl 2-oxo-2-phenylacetate (9.4063 g, 25 mmol) to afford hydrazone **2.35** (3.8226 g, 67%) as a yellow oil. Ketone **2.35**: ^{1}H NMR (400 MHz, $CDCl_3$): δ_H 10.75 (s, 1H), 7.84 (d, J = 12.23 Hz, 3H), 7.59 (d, J = 7.66 Hz, 2H), 7.35 (t, J =

7.66 Hz, 2H), 7.27 (t, J = 7.66 Hz, 1H), 5.31 (s, 2H), 1.37 (s, 9H); ^{13}C {^{1}H} NMR (101 MHz, $CDCl_3$): δ_C 162.7, 138.8, 137.4, 132.5, 132.2, 131.9, 131.5, 128.4, 127.9, 127.8, 126.9, 124.7, 123.0, 122.0, 64.0, 55.5, 28.8; ^{19}F NMR (471 MHz, $CDCl_3$): δ -62.9; IR (υ, cm^{-1}, neat): 3375, 3194, 3149, 2949, 1670; HRMS (ESI): m/z calcd for $C_{21}H_{20}F_6N_2O_2$: 447.1507 $[M+H]^+$; found: 447.1517

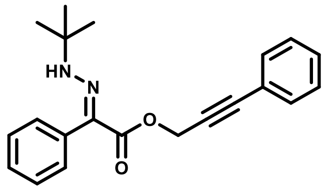

Synthesised according to general procedure D from 3-phenylprop-2-yn-1-yl 2-oxo-2-phenylacetate (6.6070 g, 25 mmol) to afford hydrazone **2.36** (5.5179 g, 66%) as a yellow oil. Hydrazone **2.36**: ^{1}H NMR (400 MHz, $CDCl_3$): δ_H 10.94 (s, 1H), 7.85 (d, J = 8.23 Hz, 2H), 7.63-7.60 (m, 2H), 7.52 (t, J = 8.23 Hz, 2H), 7.44-7.40 (m, 4H), 5.18 (s, 2H), 1.52 (s, 9H); ^{13}C {^{1}H} NMR (101 MHz, $CDCl_3$): δ_C 162.5, 137.2, 131.8, 128.6, 128.2, 128.1, 127.6, 126.5, 123.0, 122.1, 86.3, 83.2, 55.2, 52.0, 28.6; IR (υ, cm^{-1}, neat): 3378, 3180, 2949, 1673; HRMS (ESI): m/z calcd for $C_{21}H_{22}N_2O_2$: 335.1760 $[M+H]^+$; found: 335.1769

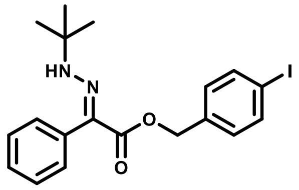

Synthesised according to general procedure D from 4-iodobenzyl 2-oxo-2-phenylacetate (9.1538 g, 25 mmol) to afford hydrazone **2.37** (7.9623 g, 73%) as a colourless oil. Hydrazone **2.37**: ^{1}H NMR (400 MHz, $CDCl_3$): δ_H 10.65 (s, 1H), 7.70-7.68 (m, 2H), 7.56-7.54 (m, 2H), 7.34-7.30 (m, 3H), 7.12-7.10 (m, 2H), 5.19 (s, 1H), 1.34 (s, 9H); ^{13}C {^{1}H} NMR (101 MHz, $CDCl_3$): δ_C 163.2, 137.8, 135.8, 129.9, 128.3, 128.2, 127.8, 127.8, 126.7, 93.9, 65.1, 55.5, 28.9; IR (υ, cm^{-1}, neat): 3379, 3183, 2950, 1670. 1626; HRMS (ESI): m/z calcd for $C_{19}H_{21}IN_2O_2$: 437.0726 $[M+H]^+$; found: 437.0733

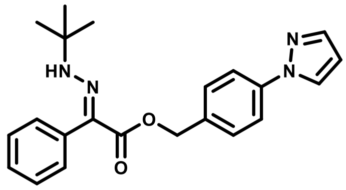

Synthesised according to general procedure D from 4-(1H-pyrazol-1-yl)benzyl 2-oxo-2-phenylacetate (7.6580 g, 25 mmol) to afford hydrazone **2.38** (6.2116 g, 66%) as a yellow oil. Hydrazone **2.38**: ^{1}H NMR (400 MHz, $CDCl_3$): δ_H 10.70 (s, 1H), 7.91-7.90 (m, 1H), 7.74-7.78 (m, 3H), 7.63-7.60 (m, 2H), 7.47-7.45 (m, 2H), 7.37-7.33 (m, 2H), 7.28-7.24 (m, 1H), 7.46-7.45 (m, 1H), 5.29 (s, 2H), 1.37 (s, 9H); ^{13}C {^{1}H} NMR (101 MHz, $CDCl_3$): δ_C 163.2, 141.2, 139.9, 137.5, 134.1, 129.2, 128.2, 127.7, 126.7, 126.6, 123.4, 119.1, 107.8, 65.1, 55.3, 28.8; IR (υ, cm^{-1}, neat): 3378, 3189, 3128, 2958, 1676; HRMS (ESI): m/z calcd for $C_{22}H_{24}N_4O_2$: 377.2031 $[M+H]^+$; found: 377.2022

Batch-Electrochemical Reaction Setup

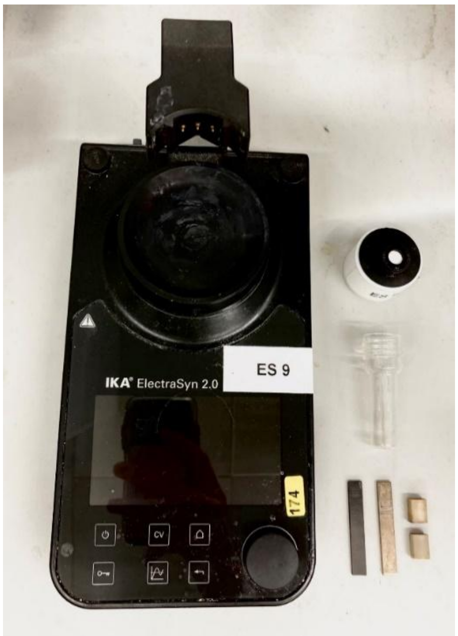

Figure S1. Batch electrochemical synthesis setup (disassembled).

Flow-Electrochemical Reaction Setup

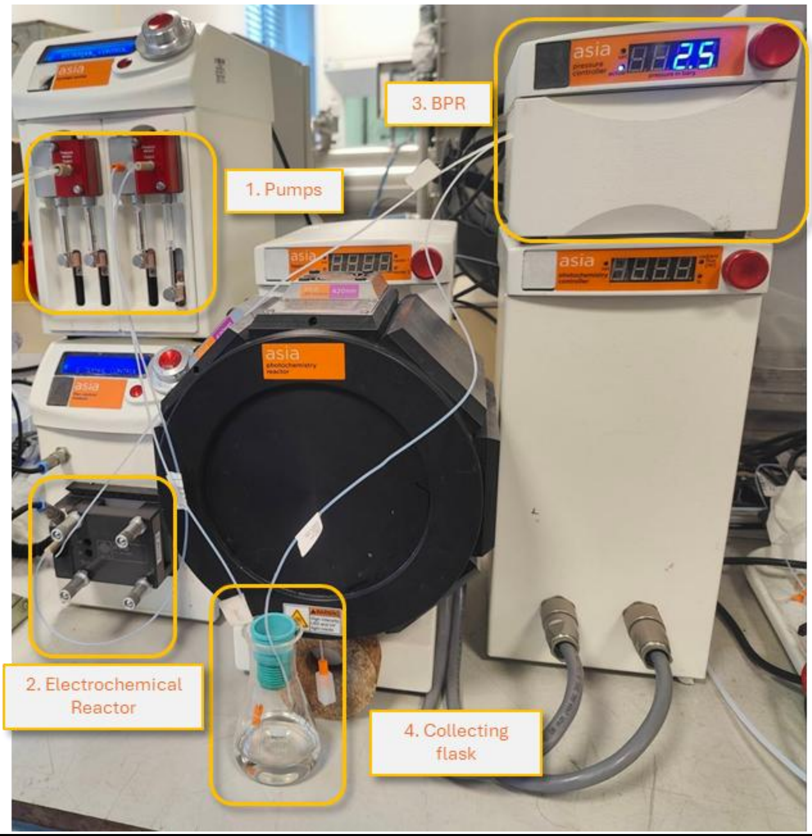

Figure S2. Flow electrochemical synthesis setup.

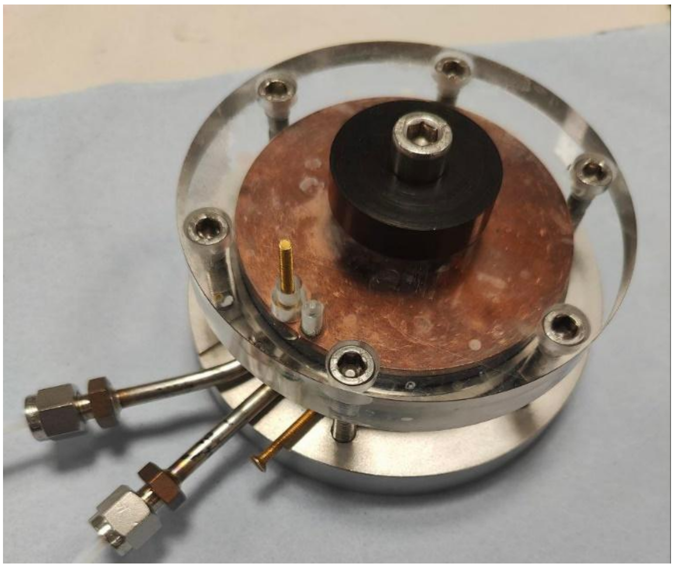

Figure S3. Ammonite 8 flowcell (closed)

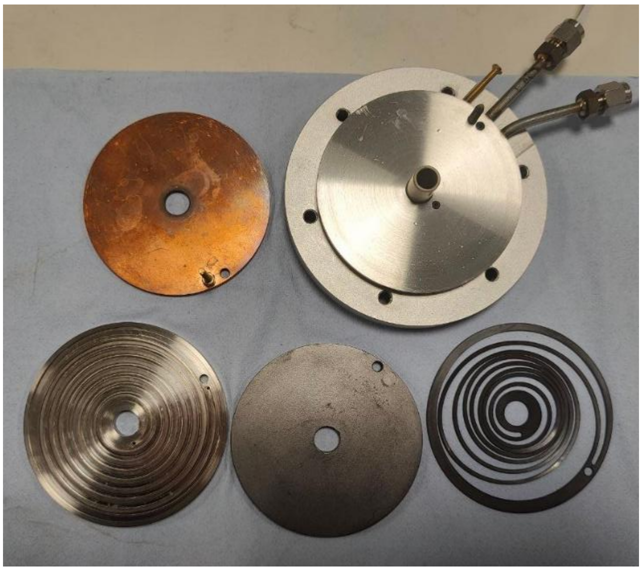

Figure S4. Ammonite 8 flowcell (open)

Cyclic Voltammetry Studies

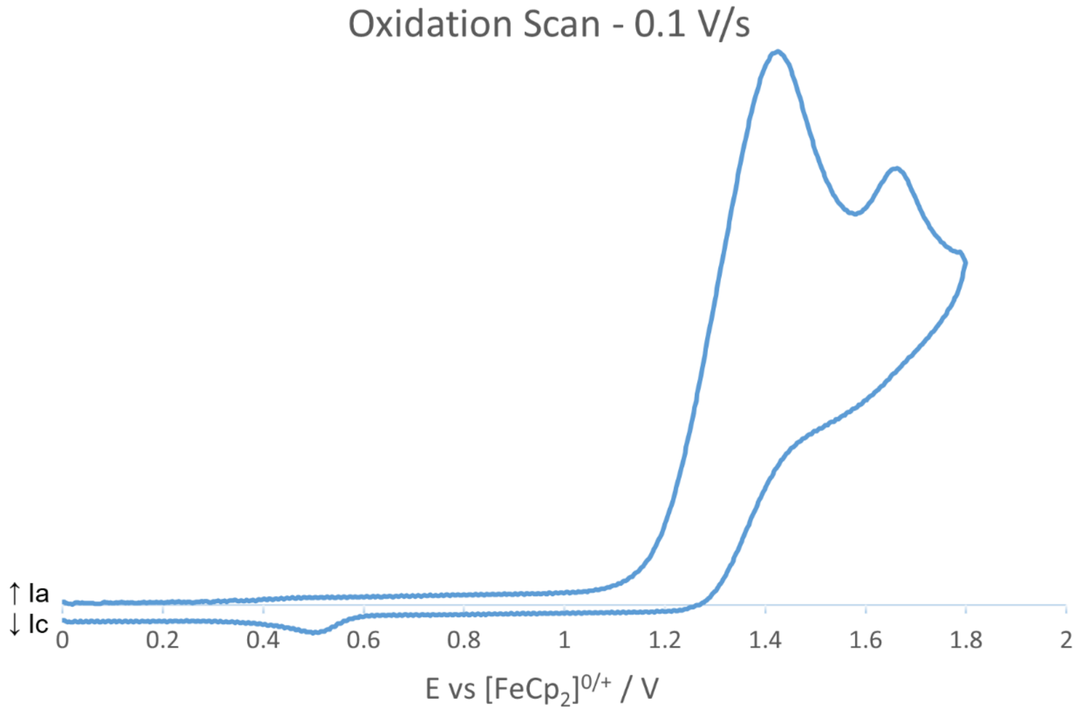

Figure S5. Cyclic voltammogram of hydrazone 3.5 as a solution in ACN (1 mM) with [$^nBu_4N$][PF_6] supporting electrolyte (0.1 M), 0.1 V s^{-1} scan rate.

DSC Studies

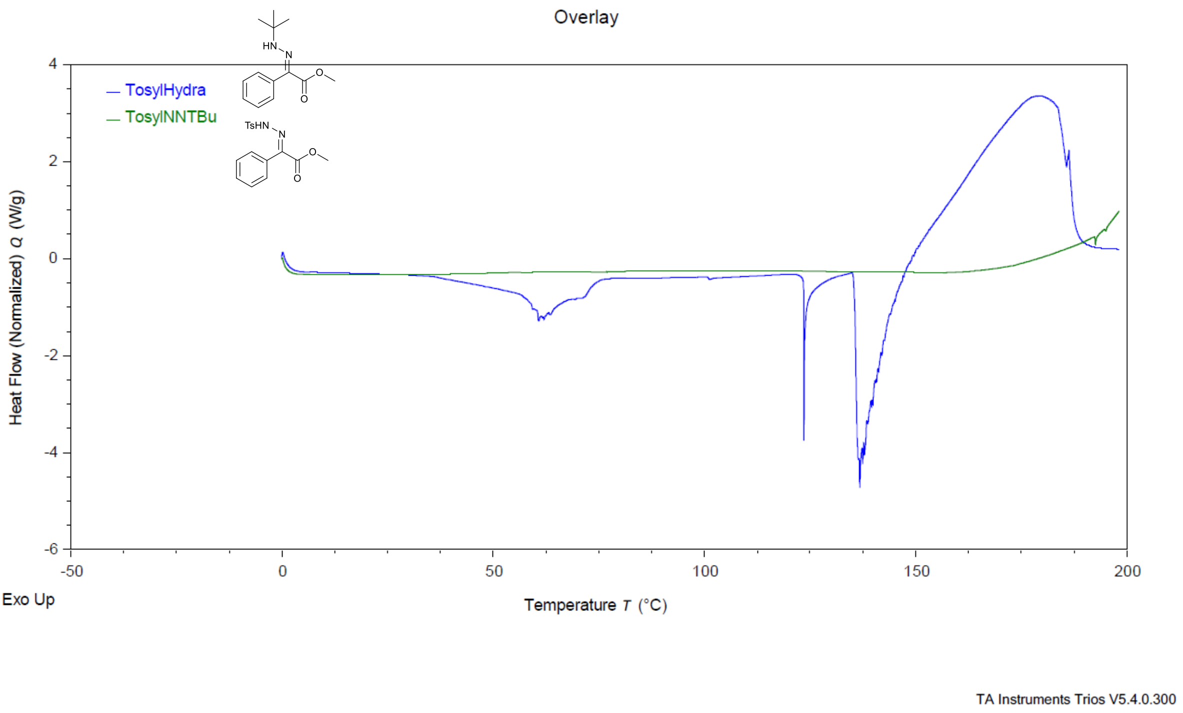

Figure S6. DSC scans of tosylhydrazone (blue trace) and *tert*-butylhydrazone (green trace)

HPLC-UV traces

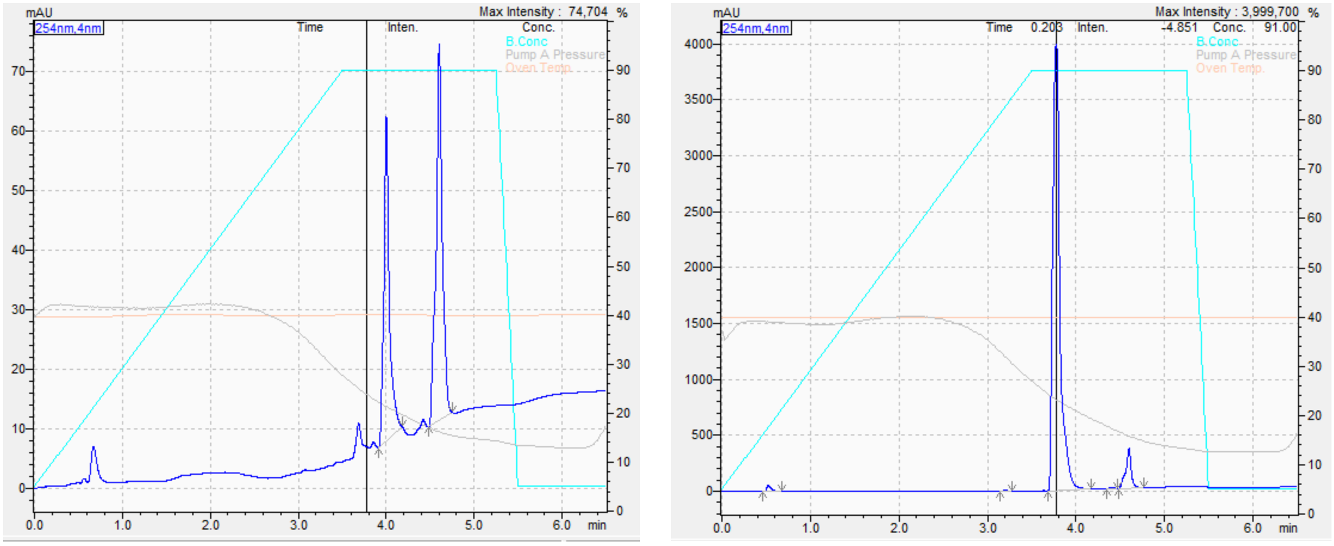

Figure S7. HPLC–UV traces showing the diazo compound (t_R = 4.0 min) and the corresponding *tert*-butyl hydrazone (t_R = 4.6 min) recorded independently (left), and the reaction mixture during the cyclopropanation process (right). The peak at t_R = 3.8 min corresponds to styrene, while the hydrazone is observed at t_R = 4.6 min. No accumulation of the diazo compound is detected under the reaction conditions. In addition, PDA spectral deconvolution was performed using Shimadzu's method, confirming the absence of any detectable diazo-derived signal.

Proposed Reaction Mechanism

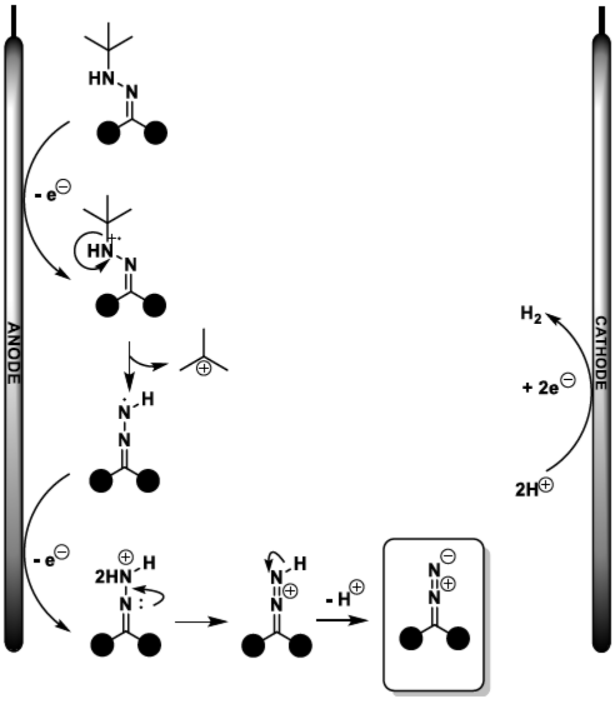

Scheme S1. Formation of diazo compounds from hydrazones with cationic leaving group.

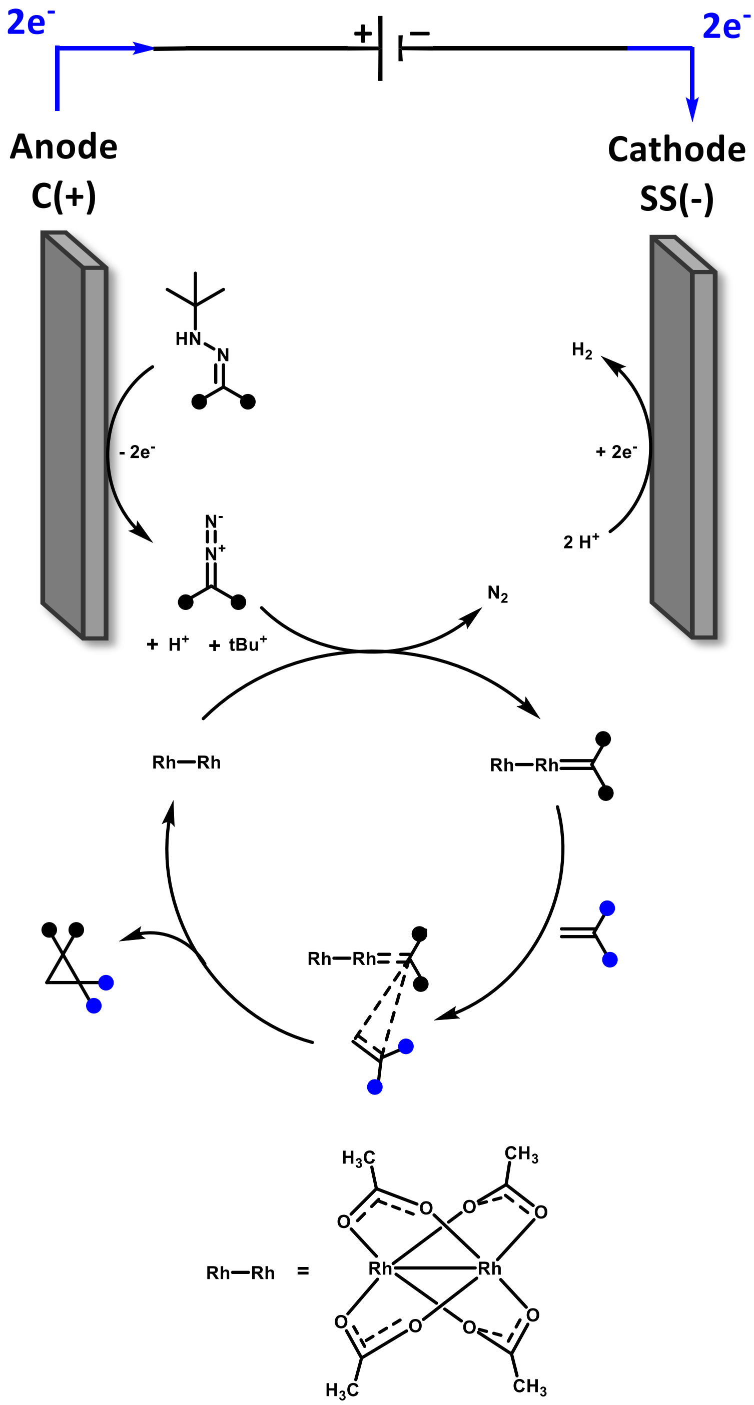

Scheme S2. Proposed reaction mechanism for the electrosynthesis of diazo compounds and rhodium(II) catalysed cyclopropanation.

Optimisation tables for electrochemical synthesis of diazo and cyclopropane compounds

Table S1. Optimisation of starting material for previous anodic oxidation conditions

Reactions were carried out on a 0.4 mmol scale at room temperature (rt) in a 5 mL ElectraSyn cell equipped with carbon and nickel electrodes, and yields were determined by ^{1}H NMR using CH_2Br_2 as an internal standard.

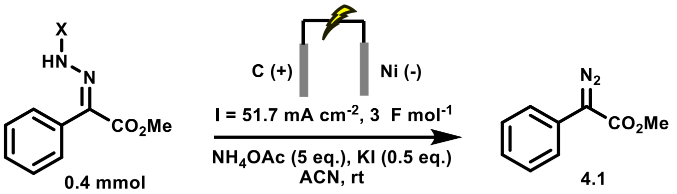

Entry	Starting Material	Yield
1	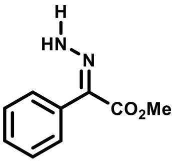	98%
2	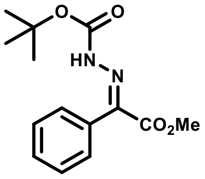	0%
3	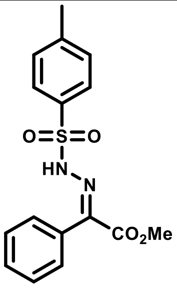	0%
4	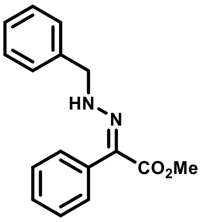	31%
5	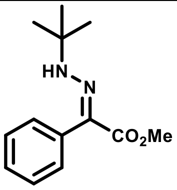	**94%**

Table S2. Optimisation of anodic oxidation of hydrazone 2.1.

Reactions were carried out on a 0.4 mmol scale at room temperature (rt) in a 5 mL ElectraSyn cell, and yields were determined by ^{1}H NMR using CH_2Br_2 as an internal standard.

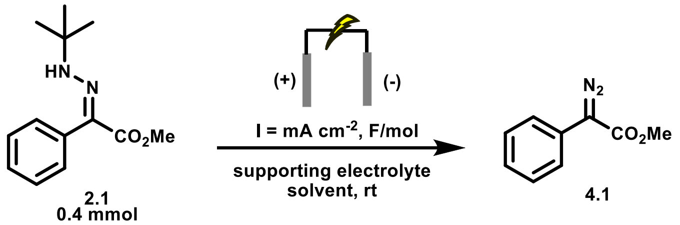

Entry	Electrodes	Supporting Electrolytes	Solvent	Conditions	Yield
**1**	C (+), Ni (-)	Collidine (5 eq.)<br>Acetic acid (4.5 eq.)<br>KI (0.5 eq.)	ACN	51.7 mA cm^{-2}, 3 F/mol	48%
**2**	**C (+), Ni (-)**	**Collidine (1 eq.)**<br>**Acetic acid (1 eq.)**<br>**KI (0.5 eq.)**	**ACN**	**51.7 mA cm^{-2}, 3 F/mol**	**51%**
**3**	C (+), Ni (-)	Coll·HBF_4 (5 eq.)<br>KI (0.5 eq.)	ACN	51.7 mA cm^{-2}, 3 F/mol	4%
**4**	C (+), Ni (-)	Coll·HBF_4 (1 eq.)<br>KI (0.5 eq.)	ACN	51.7 mA cm^{-2}, 3 F/mol	5%
**5**	C (+), Ni (-)	Collidine (2 eq.)<br>Coll·HBF_4 (2 eq.)<br>KI (0.5 eq.)	ACN	51.7 mA cm^{-2}, 3 F/mol	9%
**6**	C (+), Ni (-)	Collidine (1 eq.)<br>Coll·HBF_4 (1 eq.)<br>KI (0.5 eq.)	ACN	51.7 mA cm^{-2}, 3 F/mol	31%
**7**	C (+), Ni (-)	Collidine (3 eq.)<br>Coll·HBF_4 (1 eq.)<br>KI (0.5 eq.)	ACN	51.7 mA cm^{-2}, 3 F/mol	12%

Table S3. Optimisation of anodic oxidation of hydrazone 2.1.

Reactions were carried out on a 0.4 mmol scale at room temperature (rt) in a 5 mL ElectraSyn cell, and yields were determined by ^{1}H NMR using CH_2Br_2 as an internal standard.

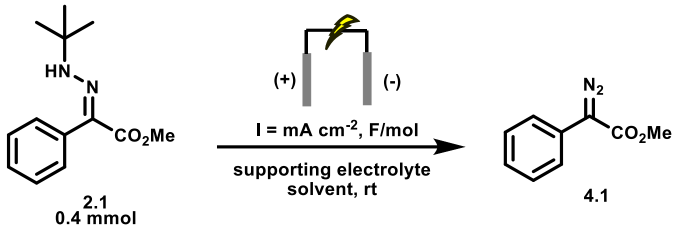

Entry	Electrodes	Supporting Electrolytes	Solvent	Conditions	Yield
**1**	C (+), Ni (-)	Et_4NI (0.5 eq.)	DCM	10.3 mA cm^{-2}, 3 F/mol	4%
**2**	C (+), Ni (-)	Et_4NI (0.5 eq.)<br>Collidine (1 eq.)	DCM	10.3 mA cm^{-2}, 3 F/mol	20%
**3**	C (+), Ni (-)	Et_4NI (1 eq.)	DCM	10.3 mA cm^{-2}, 3 F/mol	7%
**4**	C (+), Ni (-)	Et_4NI (0.5 eq.)	DCM	5.2 mA cm^{-2}, 3 F/mol	4%
**5**	C (+), Ni (-)	Et_4NI (1 eq.)<br>Coll·AcOH (1 eq.)<br>Collidine (1 eq.)	DCM	10.3 mA cm^{-2}, 5 F/mol	56%
**6**	C (+), Ni (-)	Et_4NI (2 eq.)<br>Coll·AcOH (1 eq.)	DCM	10.3 mA cm^{-2}, 5 F/mol	73%
**7**	C (+), Ni (-)	Et_4NI (0.5 eq.)<br>Coll·AcOH (1 eq.)	DCM	10.3 mA cm^{-2}, 5 F/mol	71%
**8**	C (+), Ni (-)	Et_4NI (1 eq.)<br>Coll·AcOH (1 eq.)	DCM	10.3 mA cm^{-2}, 5 F/mol	38%
**9**	C (+), Ni (-)	Et_4NI (0.5 eq.)<br>Coll·AcOH (1 eq.)	DCM	10.3 mA cm^{-2}, 3 F/mol	42%
**10**	C (+), Ni (-)	Et_4NI (0.5 eq.)<br>Coll·AcOH (1 eq.)	DCM	10.3 mA cm^{-2}, 4 F/mol	61%
**11**	C (+), Ni (-)	Et_4NI (0.5 eq.)<br>Coll·AcOH (1 eq.)	DCM	10.3 mA cm^{-2}, 6 F/mol	67%
**12**	C (+), Ni (-)	Et_4NI (0.5 eq.)<br>Coll·AcOH (2 eq.)	DCM	10.3 mA cm^{-2}, 5 F/mol	65%
**13**	C (+), Ni (-)	Et_4NI (0.5 eq.)<br>Coll·AcOH (0.5 eq.)	DCM	10.3 mA cm^{-2}, 5 F/mol	46%
**14**	C (+), Ni (-)	TBAI (0.5 eq.)<br>Coll·AcOH (1 eq.)	DCM	20.7 mA cm^{-2}, 3 F/mol	33%
**15**	C (+), Ni (-)	TBAI (0.5 eq.)<br>Coll·AcOH (2 eq.)	DCM	20.7 mA cm^{-2}, 3 F/mol	82%
**16**	C (+), Steel (-)	TBAI (0.5 eq.)<br>Coll·AcOH (1 eq.)	DCM	20.7 mA cm^{-2}, 3 F/mol	34%
**17**	**C (+), Steel (-)**	**TBAI (0.5 eq.)<br>Coll·AcOH (2 eq.)**	**DCM**	**20.7 mA cm^{-2}, 3 F/mol**	**88%**
**18**	C (+), Steel (-)	TBAI (0.5 eq.)<br>Coll·AcOH (3 eq.)	DCM	20.7 mA cm^{-2}, 3 F/mol	84%
**19**	C (+), Steel (-)	TBAI (0.5 eq.)<br>Coll·AcOH (2 eq.)	DCM	31 mA cm^{-2}, 3 F/mol	62%
**20**	C (+), Steel (-)	TBAI (0.5 eq.)<br>Coll·AcOH (2 eq.)	DCM	20.7 mA cm^{-2}, 4 F/mol	70%

Table S4. Optimisation of anodic oxidation of hydrazone 2.1: Screening of supporting electrolytes/acids

Reactions were carried out on a 0.4 mmol scale at room temperature (rt) in a 5 mL ElectraSyn cell, and yields were determined by ^1H NMR using CH_2Br_2 as an internal standard.

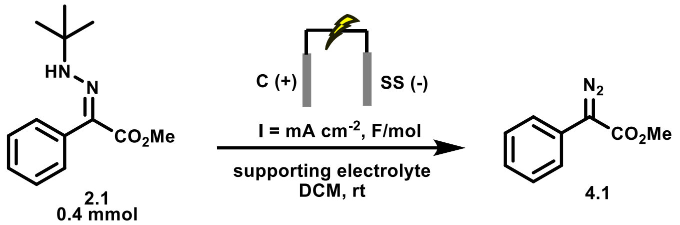

Entry	Supporting Electrolytes	Conditions	Yield
**1**	TBAI (0.5 eq.), Coll.TFA (2 eq.)	20.7 mA cm^{-2}, 3 F/mol	46%
**2**	**TBAI (0.5 eq.), Coll.TFA (2 eq.)**	**20.7 mA cm^{-2}, 4 F/mol**	**71%**
**3**	TBAI (0.5 eq.), Coll.TFA (2 eq.)	20.7 mA cm^{-2}, 5 F/mol	34%
**4**	TBAI (0.5 eq.), Coll.TFA (3 eq.)	20.7 mA cm^{-2}, 4 F/mol	56%
**5**	TBAI (0.5 eq.), Coll.TFA (2 eq.)	15.5 mA cm^{-2}, 3 F/mol	64%
**6**	TBAI (0.5 eq.), Coll.TFA (2 eq.)	25.8 mA cm^{-2}, 3 F/mol	51%
**7**	Coll.TFA (3 eq.)	10.3 mA cm^{-2}, 4 F/mol	N/A
**8**	**TBAI (0.5 eq.), Coll.TfOH (2 eq.)**	**20.7 mA cm^{-2}, 4 F/mol**	**79%**
**9**	Coll.TfOH (2 eq.)	20.7 mA cm^{-2}, 4 F/mol	N/A
**10**	Coll.TfOH (2 eq.)	10.3 mA cm^{-2}, 4 F/mol	N/A
**11**	Coll.TfOH (3 eq.)	10.3 mA cm^{-2}, 4 F/mol	N/A

Table S5. Optimisation of anodic oxidation of hydrazone 2.1. : effect of Coll·HBF4 loading, current density, and electrodes

Reactions were carried out on a 0.4 mmol scale at room temperature (rt) in a 5 mL ElectraSyn cell, and yields were determined by ^{1}H NMR using CH_2Br_2 as an internal standard.

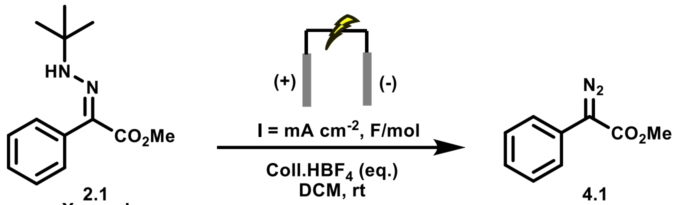

2.1
X mmol

4.1

**Entry**	**SM**	**Coll·HBF$_4$**	**Current (mA cm^{-2})**	**F/mol**	**DCM**	**Electrodes**	**Yield**
**1**	0.4 mmol	3 eq.	10.3	1.5	5 mL	C(+), SS(-)	64%
**2**	0.4 mmol	3 eq.	20.7	2	5 mL	C(+), SS(-)	33%
**3**	0.4 mmol	3 eq.	N/A	N/A	5 mL	C(+), SS(-)	0%
**4**	0.4 mmol	3 eq.	5.2	1	5 mL	C(+), SS(-)	42%
**5**	0.4 mmol	3 eq.	10.3	1	5 mL	C(+), SS(-)	20%
**6**	0.4 mmol	3 eq.	20.7	1	5 mL	C(+), SS(-)	37%
**7**	0.4 mmol	3 eq.	5.2	2	5 mL	C(+), SS(-)	24%
**8**	0.4 mmol	3 eq.	10.3	3	5 mL	C(+), SS(-)	0%
**9**	0.4 mmol	3 eq.	20.7	3	5 mL	C(+), SS(-)	0%
**10**	0.4 mmol	3 eq.	5.2	1.5	5 mL	C(+), SS(-)	78%
**11**	0.4 mmol	3 eq.	20.7	1.5	5 mL	C(+), SS(-)	41%
**12**	0.4 mmol	3 eq.	2.1	1.5	5 mL	C(+), SS(-)	71%
**13**	0.4 mmol	3 eq.	5.2	1.5	5 mL	C(+), Ni(-)	77%
**14**	0.2 mmol	3 eq.	5.2	1.5	5 mL	C(+), SS(-)	82%
**15**	**0.4 mmol**	**2 eq.**	**5.2**	**1.5**	**5 mL**	**C(+), SS(-)**	**89%**
**16**	0.4 mmol	5 eq.	5.2	1.5	5 mL	C(+), SS(-)	61%
**17**	0.4 mmol	3 eq.	5.2	1.2	5 mL	C(+), SS(-)	47%
**18**	0.4 mmol	3 eq.	5.2	1.4	5 mL	C(+), SS(-)	55%
**19**	0.4 mmol	3 eq.	5.2	1.6	5 mL	C(+), SS(-)	56%
**20**	0.4 mmol	1.5 eq.	5.2	1.5	5 mL	C(+), SS(-)	56%

Table S6. Optimisation of conditions for the electrosynthesis of cyclopropane 3.1.

Reactions were carried out on a 0.4 mmol scale at room temperature (rt) in a 5 mL ElectraSyn cell equipped with carbon and stainless steel electrodes, and yields were determined by ^1H NMR using CH_2Br_2 as an internal standard.

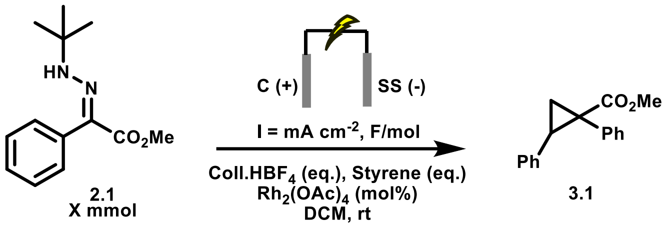

Entry	SM (mmol)	Coll·HBF$_4$ (eq.)	Styrene (eq.)	Rh$_2$(OAc)$_4$ (mol%)	DCM (mL)	Current (mA cm^{-2})	Charge (F/mol)	Yield
**1**	0.4	2	10	1	5	5.2	1.5	43%
**2**	0.4	2	10	1	5	1	2.5	61%
**3**	0.4	0.5	10	1	5	1	2.2	97%
**4**	0.4	0.5	10	1	5	2.1	2	95%
**5**	**0.4**	**0.5**	**10**	**1**	**5**	**3.1**	**2**	**97%**
**6**	0.4	0.5	10	1	5	5.2	2	90%
**7**	1	0.5	10	1	10	3.1	2.1	93%
**8**	0.8	0.5	5	1	5	3.1	2	75%
**9**	0.4	1	10	1	5	7.2	2.3	83%
**10**	0.4	0.5	10	0.5	5	3.1	2.2	41%
**11**	0.4	1	10	1	5	10.3	2.3	78%
**12**	0.4	2.5	10	1	5	20.7	8	55%
**13**	0.4	0.5	10	N/A	5	3.1	2	0%
**14**	0.4	0.5	10	1	5	N/A	N/A	0%
**15**	0.4	N/A	10	1	5	3.1	2	17%

General Procedure F for the electrochemical cyclopropanation of activated hydrazones

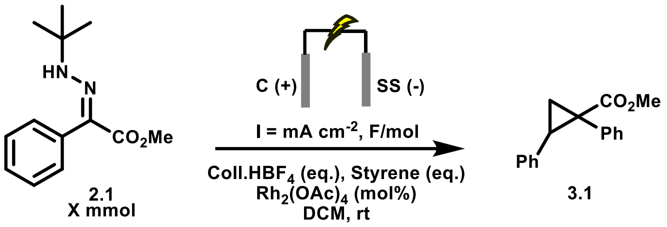

In a 5 mL ElectraSyn 2.0 vial, the hydrazone (0.4 mmol), Coll HBF_4 (0.2 mmol, 0.0418 g, 0.5 eq.), styrene (4 mmol, 0.4166 g, 10 eq.), and rhodium (II) acetate (1 mol%) were dissolved in DCM (5 mL). The ElectraSyn 2.0 cap was equipped with a C_{gr} anode (working electrode) and a stainless steel cathode (counter electrode). The reaction conditions were set to 3.1 mA cm^{-2} and 2 F/mol. The reaction was stirred vigorously and electrolysed at room temperature. The mixture was quenched with water and extracted with EtOAc (3 x 50 mL). The combined organics were dried over $MgSO_4$ and the solvent removed under reduced pressure. The crude residue was purified by silica gel flash chromatography (Hexane/EtOAc = 9:1 to 7:3) to afford the desired cyclopropane. The procedure was repeated with different hydrazones of varying functionality.

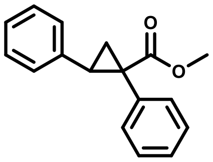

Synthesised according to general procedure F from hydrazone **2.1** (0.0937 g, 0.4 mmol) to afford cyclopropane **3.1** (0.0979 g, 97%) as a colourless oil. Cyclopropane **3.1**: ^{1}H NMR (400 MHz, $CDCl_3$): δ_H 7.15-7.13 (m, 3H), 7.07-7.03 (m, 5H), 6.79-6.77 (m, 2H), 3.67 (s, 3H), 3.14-3.11 (dd, J = 9.35, 7.20 Hz, 1H), 2.17-2.14 (dd, J = 9.29, 4.74 Hz, 1H), 1.91-1.88 (dd, J = 7.34, 4.97 Hz, 1H); ^{13}C {^{1}H} NMR (101 MHz, $CDCl_3$): δ_C 174.5, 136.5, 134.8, 132.1, 128.2, 127.8, 127.2, 126.4, 52.8, 37.5, 33.3, 20.6; IR (υ, cm^{-1}, neat): 1716; HRMS (ESI): m/z calcd for $C_{17}H_{16}O_2$: 253.1229 $[M+H]^+$; found: 253.1219

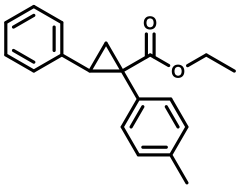

Synthesised according to general procedure F from hydrazone **2.12** (0.1049 g, 0.4 mmol) to afford cyclopropane **3.2** (0.0864 g, 77%) as a colourless oil. Cyclopropane **3.2**: ^{1}H NMR (400 MHz, $CDCl_3$): δ_H 7.07-7.05 (m, 3H), 6.94-6.89 (m, 4H), 6.79-6.77 (m, 2H), 4.19-4.08 (m, 2H), 3.08-3.05 (dd, J = 9.46, 7.29 Hz, 1H), 2.24 (s, 3H), 2.12-2.09 (dd, J = 9.46, 5.11 Hz, 1H), 1.84-1.82 (dd, J = 7.16, 5.11 Hz, 1H), 1.20-1.17 (t, J = 6.90 Hz, 3H); ^{13}C {^{1}H} NMR (101 MHz, $CDCl_3$): δ_C 174.1, 136.8, 136.6, 131.8, 128.5, 128.2, 127.8, 126.3, 61.4, 37.4, 33.0, 21.3, 20.4, 14.3; IR (υ, cm^{-1}, neat): 1723; HRMS (ESI): m/z calcd for $C_{19}H_{20}O_2$: 281.1542 $[M+H]^+$; found: 281.1545

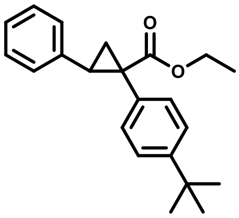

Synthesised according to general procedure F from hydrazone **2.13** (0.1218 g, 0.4 mmol) to afford cyclopropane **3.3** (0.0890 g, 69%) as a colourless oil. Cyclopropane **3.3**: ^{1}H NMR (400 MHz, $CDCl_3$): δ_H 7.13-7.10 (m, 2H), 7.04-7.02 (m, 3H), 6.94-6.92 (m, 2H), 6.74-6.72 (m, 2H), 4.20-4.08 (m, 2H), 3.06-3.03 (dd, J = 9.50, 7.24 Hz, 1H), 2.14-2.11 (dd, J = 9.39, 4.87 Hz, 1H), 1.84-1.82 (dd, J = 7.24, 4.87 Hz, 1H), 1.23 (s, 9H), 1.21-1.18 (t, J = 7.06 Hz, 3H); ^{13}C {^{1}H} NMR (101 MHz, $CDCl_3$): δ_C 174.1, 149.8, 136.8, 131.6, 130.2, 128.2, 127.7, 126.2, 124.6, 61.3, 37.4, 34.5, 33.0, 31.4, 20.5, 14.3; IR (υ, cm^{-1}, neat): 1725; HRMS (ESI): m/z calcd for $C_{22}H_{26}O_2$: 323.2011 $[M+H]^+$; found: 323.2014

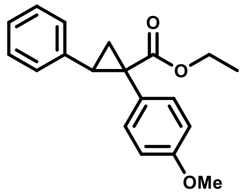

Synthesised according to general procedure F from hydrazone **2.5** (0.1057 g, 0.4 mmol) to afford cyclopropane **3.4** (0.0996 g, 84%) as a yellow oil. Cyclopropane **3.4**: ^{1}H NMR (400 MHz, $CDCl_3$): δ_H 7.07-7.06 (m, 3H), 6.94-6.93 (m, 2H), 6.79-6.77 (m, 2H), 6.67-6.65 (m, 2H), 4.18-4.08 (m, 2H), 3.72 (s, 3H), 3.07-3.04 (dd, J = 9.09, 7.18 Hz, 1H), 2.13-2.10 (dd, J = 9.33, 5.02 Hz, 1H), 1.83-1.80 (dd, J = 7.18, 4.79 Hz, 1H), 1.20-1.17 (t, J = 7.18 Hz, 3H); ^{13}C {^{1}H} NMR (101 MHz, $CDCl_3$): δ_C 174.2, 158.5, 136.8, 133.0, 128.2, 127.8, 127.1, 126.3, 113.2, 61.3, 55.2, 37.0, 33.1, 20.6, 14.3; IR (υ, cm^{-1}, neat): 1717; HRMS (ESI): m/z calcd for $C_{19}H_{20}O_3$: 297.1491 [M+H]$^+$; found: 297.1492

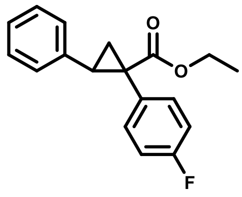

Synthesised according to general procedure F from hydrazone **2.3** (0.1065 g, 0.4 mmol) to afford cyclopropane **3.5** (0.0807 g, 71%) as a colourless oil. Cyclopropane **3.5**: ^{1}H NMR (400 MHz, $CDCl_3$): δ_H 7.08-7.07 (m, 3H), 7.00-6.97 (m, 2H), 6.82-6.76 (m, 5H), 4.17-4.09 (m, 2H), 3.11-3.07 (dd, J = 9.19, 7.24 Hz, 1H), 2.15-2.13 (dd, J = 9.19, 4.60 Hz, 1H), 1.85-1.83 (dd, J = 7.35, 5.05 Hz, 1H), 1.20-1.17 (t, J = 7.24 Hz, 3H); ^{13}C {^{1}H} NMR (101 MHz, $CDCl_3$): δ_C 173.7, 162.8, 160.9, 136.3, 133.6, 133.5, 130.9, 130.9, 128.2, 127.9, 126.5, 114.8, 114.6, 61.5, 36.9, 33.1, 20.4, 14.3; ^{19}F NMR (471 MHz, $CDCl_3$): δ -115.2; IR (υ, cm^{-1}, neat): 1718; HRMS (ESI): m/z calcd for $C_{18}H_{17}FO_2$: 285.1291 [M+H]$^+$; found: 285.1282

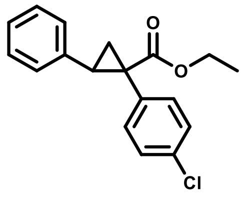

Synthesised according to general procedure F from hydrazone **2.2** (0.1131 g, 0.4 mmol) to afford cyclopropane **3.6** (0.0999 g, 83%) as a colourless liquid. Cyclopropane **3.6**: ^{1}H NMR (400 MHz, $CDCl_3$): δ_H 7.10-7.07 (m, 5H), 6.96-6.95 (m, 2H), 6.79-6.77 (m, 2H), 4.18-4.09 (m, 2H), 3.12-3.09 (dd, J = 9.68, 7.37 Hz, 1H), 2.15-2.12 (dd, J = 9.22, 4.95 Hz, 1H), 1.85-1.83 (dd, J = 7.37, 5.07 Hz, 1H), 1.20-1.17 (t, J = 7.37 Hz, 3H); ^{13}C {^{1}H} NMR (101 MHz, $CDCl_3$): δ_C 173.4, 136.1, 133.7, 133.3, 132.9, 128.1, 128.1, 128.0, 126.6, 61.5, 37.0, 33.1, 20.2, 14.3; IR (υ, cm^{-1}, neat): 1717; HRMS (ESI): m/z calcd for $C_{18}H_{17}ClO_2$: 301.0995 [M+H]$^+$; found: 301.0984

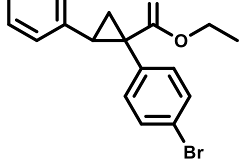

Synthesised according to general procedure F from hydrazone **2.4** (0.1309 g, 0.4 mmol) to afford cyclopropane **3.7** (0.0801 g, 58%) as a yellow oil. Cyclopropane **3.7**: ^{1}H NMR (400 MHz, $CDCl_3$): δ_H 7.26-7.25 (m, 2H), 7.09-7.08 (m, 3H), 6.90-6.88 (m, 2H), 6.79-6.77 (m, 2H), 4.18-4.08 (m, 2H), 3.11-3.08 (dd, J = 9.44, 7.50 Hz, 1H), 2.15-2.12 (dd, J = 9.15, 4.87 Hz, 1H), 1.84-1.82 (dd, J = 7.50, 5.26 Hz, 1H), 1.19-1.16 (t, J = 7.20 Hz, 3H); ^{13}C {^{1}H} NMR (101 MHz, $CDCl_3$): δ_C 173.4, 136.1, 133.7, 132.4, 130.9, 128.1, 128.0, 126.6, 121.2, 61.6, 37.1, 33.1, 20.2, 14.3; IR (υ, cm^{-1}, neat): 1720; HRMS (ESI): m/z calcd for $C_{18}H_{17}BrO_2$: 345.0490 [M+H]$^+$; found: 345.0472

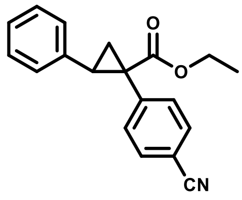

Synthesised according to general procedure F from hydrazone **2.22** (0.1093 g, 0.4 mmol) to afford cyclopropane **3.8** (0.0944 g, 81%) as a colourless oil. Cyclopropane **3.8**: ^{1}H NMR (400 MHz, $CDCl_3$): δ_H

7.42-7.39 (m, 2H), 7.14-7.06 (m, 5H), 6.77-6.75 (m, 2H), 4.19-4.09 (m, 2H), 3.19-3.14 (dd, J = 9.30, 7.07 Hz, 1H), 2.20-2.16 (dd, J = 9.36, 5.07 Hz, 1H), 1.92-1.89 (dd, J = 7.31, 5.07 Hz, 1H), 1.20-1.16 (t, J = 7.13 Hz, 3H); ^{13}C {^{1}H} NMR (101 MHz, $CDCl_3$): δ_C 172.6, 140.8, 135.5, 132.8, 131.6, 131.6, 128.2, 128.0, 127.0, 119.0, 110.9, 61.8, 37.5, 33.4, 19.8, 14.2; IR (υ, cm^{-1}, neat): 1713; HRMS (ESI): m/z calcd for $C_{19}H_{17}NO_2$: 292.1340 [M+H]$^+$; found: 292.1345

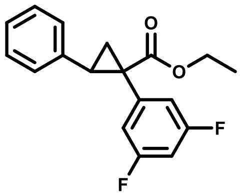

Synthesised according to general procedure F from hydrazone **2.6** (0.1137 g, 0.4 mmol) to afford cyclopropane **3.9** (0.0980 g, 81%) as a colourless liquid. Cyclopropane **3.9**: ^{1}H NMR (400 MHz, $CDCl_3$): δ_H 7.12-7.09 (m, 3H), 6.84-6.81 (m, 2H), 6.59-6.55 (m, 3H), 4.21-4.11 (m, 2H), 3.16-3.12 (dd, J = 9.49, 7.38 Hz, 1H), 2.14-2.11 (dd, J = 9.24, 5.19 Hz, 1H), 1.88-1.85 (dd, J = 7.38, 5.19 Hz, 1H), 1.20-1.16 (t, J = 7.05 Hz, 3H); ^{13}C {^{1}H} NMR (101 MHz, $CDCl_3$): δ_C 172.7, 163.4, 163.3, 161.4, 161.3, 139.0, 139.0, 135.5, 128.5, 128.1, 128.0, 126.9, 126.1, 115.0, 115.0, 114.8, 114.8, 103.0, 102.8, 102.6, 61.7, 37.1, 37.1, 33.3, 20.0, 14.2; ^{19}F NMR (471 MHz, $CDCl_3$): δ -111.1; IR (υ, cm^{-1}, neat): 1716; HRMS (ESI): m/z calcd for $C_{18}H_{16}F_2O_2$: 303.1197 [M+H]$^+$; found: 303.1177

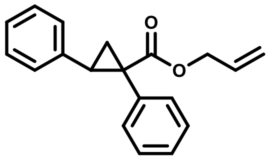

Synthesised according to general procedure F from hydrazone **2.10** (0.1041 g, 0.4 mmol) to afford cyclopropane **3.10** (0.0646 g, 58%) as a colourless liquid. Cyclopropane **3.10**: ^{1}H NMR (400 MHz, $CDCl_3$): δ_H 7.13-7.11 (m, 3H), 7.06-7.02 (m, 5H), 6.79-6.76 (m, 2H), 5.88-5.78 (m, 1H), 5.15-5.10 (m, 2H), 4.64-4.52 (m, 2H), 3.14-3.10 (dd, J = 9.31, 7.10 Hz, 1H), 2.14-2.11 (dd, J = 9.31, 5.10 Hz, 1H), 1.91-1.88 (dd, J = 7.43, 4.98 Hz, 1H); ^{13}C {^{1}H} NMR (101 MHz, $CDCl_3$): δ_C 173.6, 136.5, 134.8, 132.2, 132.1, 128.2, 127.8, 127.8, 127.1, 126.5, 117.4, 65.7, 33.2, 31.7, 14.3; IR (υ, cm^{-1}, neat): 1710; HRMS (ESI): m/z calcd for $C_{19}H_{18}O_2$: 279.1385 [M+H]$^+$; found: 279.1383

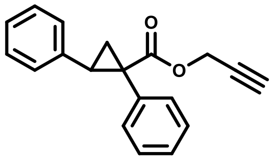

Synthesised according to general procedure F from hydrazone **2.11** (0.1033 g, 0.4 mmol) to afford cyclopropane **3.11** (0.0928 g, 84%) as a colourless liquid. Cyclopropane **3.11**: ^{1}H NMR (400 MHz, $CDCl_3$): δ_H 7.14-7.12 (m, 3H), 7.06-7.02 (m, 5H), 6.79-6.76 (m, 2H), 5.88-5.78 (m, 1H), 4.72-4.62 (m, 2H), 3.17-3.13 (dd, J = 9.52, 7.28 Hz, 1H), 2.43-2.42 (t, J = 2.52 Hz, 1H), 2.21-2.17 (dd, J = 9.32, 5.03 Hz, 1H), 1.94-1.91 (dd, J = 7.28, 5.03 Hz, 1H); ^{13}C {^{1}H} NMR (101 MHz, $CDCl_3$): δ_C 173.1, 136.2, 134.3, 132.1, 128.2, 127.9, 127.3, 126.6, 77.9, 74.8, 52.8, 37.4, 33.5, 20.6; IR (υ, cm^{-1}, neat): 1711; HRMS (ESI): m/z calcd for $C_{19}H_{16}O_2$: 277.1229 [M+H]$^+$; found: 277.1230

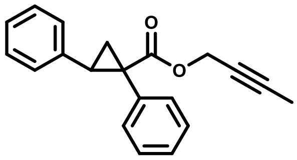

Synthesised according to general procedure F from hydrazone **2.18** (0.1089 g, 0.4 mmol) to afford cyclopropane **3.12** (0.0894 g, 77%) as a colourless liquid. Cyclopropane **3.12**: ^{1}H NMR (400 MHz, $CDCl_3$): δ_H 7.13-7.11 (m, 3H), 7.06-7.02 (m, 5H), 6.78-6.76 (m, 2H), 4.69-4.59 (m, 2H), 3.16-3.12 (dd, J = 9.45, 7.44 Hz, 1H), 2.19-2.16 (dd, J = 9.35, 5.03 Hz, 1H), 1.92-1.89 (dd, J = 7.44, 5.03 Hz, 1H), 1.84-1.83 (t, J = 2.40 Hz, 3H); ^{13}C {^{1}H} NMR (101 MHz, $CDCl_3$): δ_C 173.3, 136.4, 134.5, 132.1, 130.3, 128.2, 127.8, 127.2, 126.5, 83.0, 73.5, 53.7, 37.6, 33.3, 20.4, 3.9; IR (υ, cm^{-1}, neat): 1711; HRMS (ESI): m/z calcd for $C_{20}H_{18}O_2$: 291.1385 [M+H]$^+$; found: 291.1395

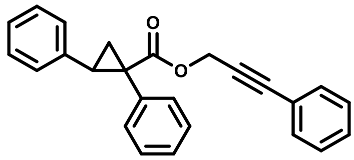

Synthesised according to general procedure F from hydrazone **2.36** (0.1338 g, 0.4 mmol) to afford cyclopropane **3.13** (0.0240 g, 17%) as a colourless liquid. Cyclopropane **3.13**: ^{1}H NMR (400 MHz, $CDCl_3$): δ_H 7.45-7.42 (m, 2H), 7.34-7.29 (m, 3H), 7.14-7.12 (m, 3H), 7.08-7.04 (m, 5H), 6.80-6.77 (m, 2H), 4.96-4.86 (m, 2H), 3.20-3.16 (dd, J = 9.16, 7.12 Hz, 1H), 2.23-2.20 (dd, J = 9.26, 4.78 Hz, 1H), 1.96-1.93 (dd, J = 7.43, 5.09 Hz, 1H); ^{13}C {^{1}H} NMR (101 MHz, $CDCl_3$): δ_C 173.2, 136.3, 134.5, 132.1, 132.1, 128.8, 128.4, 128.2, 127.8, 127.3, 126.5, 122.4, 86.4, 83.3, 53.7, 37.6, 33.4, 20.5; IR (υ, cm^{-1}, neat): 1714; HRMS (ESI): m/z calcd for $C_{25}H_{20}O_2$: 353.1542 $[M+H]^+$; found: 353.1554

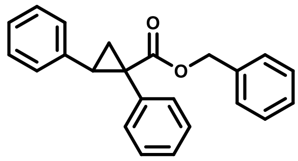

Synthesised according to general procedure F from hydrazone **2.20** (0.1242 g, 0.4 mmol) to afford cyclopropane **3.14** (0.1301 g, 99%) as a colourless liquid. Cyclopropane **3.14**: ^{1}H NMR (400 MHz, $CDCl_3$): δ_H 7.33-7.29 (m, 3H), 7.22-7.20 (m, 2H), 7.16-7.15 (m, 3H), 7.09-7.07 (m, 5H), 6.81-6.78 (m, 2H), 5.22-5.10 (m, 2H), 3.18-3.14 (dd, J = 9.21, 7.09 Hz, 1H), 2.21-2.17 (dd, J = 9.58, 4.58 Hz, 1H), 1.94-1.91 (dd, J = 7.09, 4.88 Hz, 1H); ^{13}C {^{1}H} NMR (101 MHz, $CDCl_3$): δ_C 173.6, 136.4, 136.3, 134.8, 132.1, 128.5, 128.4, 128.2, 128.0, 127.8, 127.8, 127.7, 127.4, 127.2, 126.4, 66.7, 37.7, 33.2, 20.4; IR (υ, cm^{-1}, neat): 1708; HRMS (ESI): m/z calcd for $C_{23}H_{20}O_2$: 329.1542 $[M+H]^+$; found: 329.1548

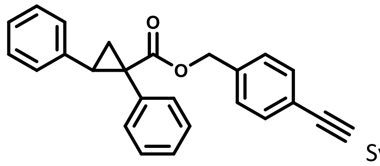

Synthesised according to general procedure F from hydrazone **2.18** (0.1089 g, 0.4 mmol) to afford cyclopropane **3.12** (0.0894 g, 77%) as a colourless liquid. Cyclopropane **3.12**: ^{1}H NMR (400 MHz, $CDCl_3$): δ_H 7.44-7.42 (m, 2H), 7.15-7.12 (m, 5H), 7.07-7.05 (m, 5H), 6.79-6.77 (m, 2H), 5.19-5.06 (m, 2H), 3.15-3.12 (dd, J = 9.26, 7.06 Hz, 1H), 3.07 (s, 1H), 2.19-2.16 (dd, J = 9.26, 5.18 Hz, 1H), 1.94-1.91 (dd, J = 7.39, 5.18 Hz, 1H); ^{13}C {^{1}H} NMR (101 MHz, $CDCl_3$): δ_C 173.6, 137.0, 136.3, 134.6, 132.3, 132.0, 128.2, 127.8, 127.8, 127.2, 127.2, 126.5, 121.7, 83.4, 77.6, 66.2, 37.6, 33.3, 20.6; IR (υ, cm^{-1}, neat): 3489, 1710; HRMS (ESI): m/z calcd for $C_{25}H_{20}O_2$: 353.1542 $[M+H]^+$; found: 353.1554

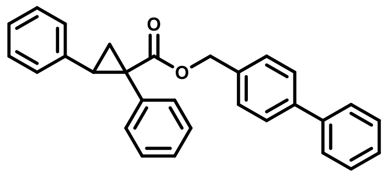

Synthesised according to general procedure F from hydrazone **2.31** (0.1546 g, 0.4 mmol) to afford cyclopropane **3.16** (0.0793 g, 49%) as a colourless liquid. Cyclopropane **3.16**: ^{1}H NMR (400 MHz, $CDCl_3$): δ_H 7.60-7.58 (m, 2H), 7.55-7.54 (m, 2H), 7.46-7.43 (m, 2H), 7.38-7.34 (m, 1H), 7.28-7.26 (m, 2H), 7.16-7.15 (m, 3H), 7.09-7.06 (m, 5H), 6.80-6.78 (m, 2H), 5.25-5.13 (m, 2H), 3.18-3.15 (dd, J = 9.42, 7.19 Hz, 1H), 2.21-2.19 (dd, J = 9.42, 4.86 Hz, 1H), 1.95-1.92 (dd, J = 7.48, 4.86 Hz, 1H); ^{13}C {^{1}H} NMR (400 MHz, $CDCl_3$): δ_C 173.7, 140.9, 140.8, 136.4, 135.3, 134.8, 132.1, 128.9, 128.2, 127.9, 127.8, 127.5, 127.3, 127.2, 127.2, 126.5, 66.5, 37.7, 33.2, 20.5; IR (υ, cm^{-1}, neat): 1713; HRMS (ESI): m/z calcd for $C_{29}H_{24}O_2$: 405.1855 $[M+H]^+$; found: 405.1856

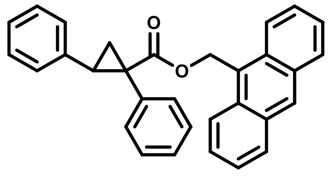

Synthesised according to general procedure F from hydrazone **2.33** (0.1642 g, 0.4 mmol) to afford cyclopropane **3.17** (0.0617 g, 36%) as a yellow oil. Cyclopropane **3.17**: ^{1}H NMR (400 MHz, $CDCl_3$): δ_H 8.49-8.28 (m, 4H), 8.03-8.01 (m, 2H), 7.82-7.80 (m, 2H), 7.54-7.47 (m, 4H), 7.07-7.05 (m, 2H), 7.00-6.94 (m,

4H), 6.68-6.66 (m, 2H), 6.23-6.07 (m, 2H), 3.05-3.02 (dd, J = 9.48, 7.23 Hz, 1H), 2.09-2.06 (dd, J = 9.48, 4.99 Hz, 1H), 1.84-1.82 (dd, J = 7.23, 4.99 Hz, 1H); ^{13}C {^{1}H} NMR (101 MHz, $CDCl_3$): δ_C 134.3, 132.0, 129.2, 129.1, 128.2, 127.7, 127.4, 127.1, 126.6, 126.4, 125.2, 125.1, 124.4, 60.1, 33.1, 20.2; IR (υ, cm^{-1}, neat): 1697; HRMS (ESI): m/z calcd for $C_{31}H_{24}O_2$: 429.3157 [M+H]$^+$; found: 429.3168

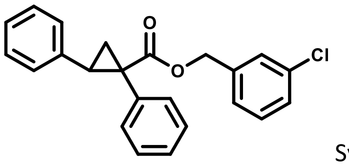

Synthesised according to general procedure F from hydrazone **2.21** (0.1379 g, 0.4 mmol) to afford cyclopropane **3.18** (0.1379 g, 95%) as a colourless liquid. Cyclopropane **3.18**: ^{1}H NMR (400 MHz, $CDCl_3$): δ_H 7.30-7.28 (m, 2H), 7.20-7.17 (m, 2H), 7.14-7.12 (m, 3H), 7.06-7.04 (m, 5H), 6.78-6.76 (m, 2H), 5.20-5.07 (m, 2H), 3.15-3.10 (dd, J = 9.34, 7.18 Hz, 1H), 2.18-2.15 (dd, J = 9.34, 5.11 Hz, 1H), 1.92-1.89 (dd, J = 7.18, 5.11 Hz, 1H); ^{13}C {^{1}H} NMR (101 MHz, $CDCl_3$): δ_C 173.7, 136.5, 136.3, 134.8, 132.1, 128.6, 128.2, 128.0, 127.8, 127.8, 127.4, 127.2, 126.5, 60.6, 37.7, 33.2, 22.8; IR (υ, cm^{-1}, neat): 1710; HRMS (ESI): m/z calcd for $C_{23}H_{19}ClO_2$: 363.1152 [M+H]$^+$; found: 363.1156

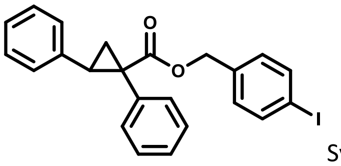

Synthesised according to general procedure F from hydrazone **2.37** (0.1745 g, 0.4 mmol) to afford cyclopropane **3.19** (0.1018 g, 56%) as a colourless liquid. Cyclopropane **3.19**: ^{1}H NMR (400 MHz, $CDCl_3$): δ_H 7.64-7.61 (m, 2H), 7.14-7.13 (m, 3H), 7.07-7.02 (m, 5H), 6.92-6.90 (m, 2H), 6.78-6.76 (m, 2H), 5.12-5.00 (m, 2H), 3.13-3.10 (dd, J = 9.50, 7.54 Hz, 1H), 2.17-2.14 (dd, J = 9.50, 5.19 Hz, 1H), 1.92-1.90 (dd, J = 7.54, 5.19 Hz, 1H); ^{13}C {^{1}H} NMR (101 MHz, $CDCl_3$): δ_C 173.6, 137.7, 136.3, 136.0, 134.6, 132.0, 129.3, 128.2, 127.9, 127.8, 127.2, 126.5, 93.6, 66.1, 37.6, 33.3, 20.6; IR (υ, cm^{-1}, neat): 1709; HRMS (ESI): m/z calcd for $C_{23}H_{19}IO_2$: 455.0508 [M+H]$^+$; found: 455.0511

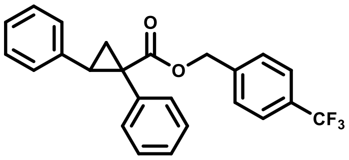

Synthesised according to general procedure F from hydrazone **2.34** (0.1514 g, 0.4 mmol) to afford cyclopropane **3.20** (0.0999 g, 63%) as a yellow oil. Cyclopropane **3.20**: ^{1}H NMR (400 MHz, $CDCl_3$): δ_H 7.57-7.55 (m, 2H), 7.27-7.25 (m, 2H), 7.17-7.15 (m, 3H), 7.09-7.06 (m, 5H), 6.81-6.78 (m, 2H), 5.25-5.12 (m, 2H), 3.18-3.14 (dd, J = 9.38, 7.14 Hz, 1H), 2.21-2.17 (dd, J = 9.38, 5.01 Hz, 1H), 1.96-1.93 (dd, J = 7.14, 5.01 Hz, 1H); ^{13}C {^{1}H} NMR (101 MHz, $CDCl_3$): δ_C 173.6, 140.3, 136.2, 134.6, 132.1, 128.2, 127.9, 127.8, 127.3, 127.3, 126.6, 125.6, 125.6, 125.5, 125.5, 65.8, 37.6, 33.4, 20.7; ^{19}F NMR (471 MHz, $CDCl_3$): δ -62.5; IR (υ, cm^{-1}, neat): 1712; HRMS (ESI): m/z calcd for $C_{24}H_{19}F_3O_2$: 397.1415 [M+H]$^+$; found: 397.1427

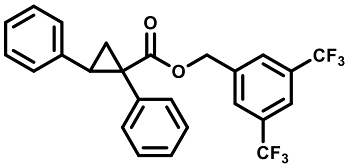

Synthesised according to general procedure F from hydrazone **2.35** (0.1786 g, 0.4 mmol) to afford cyclopropane **3.21** (0.1412 g, 76%) as a yellow oil. Cyclopropane **3.21**: ^{1}H NMR (400 MHz, $CDCl_3$): δ_H 7.81-7.76 (m, 2H), 7.53 (s, 1H), 7.18-7.17 (m, 3H), 7.09-7.05 (m, 5H), 6.82-6.79 (m, 2H), 5.29-5.15 (m, 2H), 3.18-3.14 (dd, J = 9.26, 7.34 Hz, 1H), 2.21-2.18 (dd, J = 9.26, 5.06 Hz, 1H), 1.98-1.95 (dd, J = 7.34, 5.06 Hz, 1H); ^{13}C {^{1}H} NMR (101 MHz, $CDCl_3$): δ_C 173.9, 138.9, 138.8, 137.8, 131.9, 128.4, 128.2, 128.0, 128.0, 127.6, 127.5, 127.3, 126.8, 126.7, 122.7, 121.8, 66.0, 39.3, 37.5, 25.9; ^{19}F NMR (471 MHz, $CDCl_3$): δ -62.9; IR (υ, cm^{-1}, neat): 1720; HRMS (ESI): m/z calcd for $C_{25}H_{18}F_6O_2$: 465.1392 [M+H]$^+$; found: 465.1392

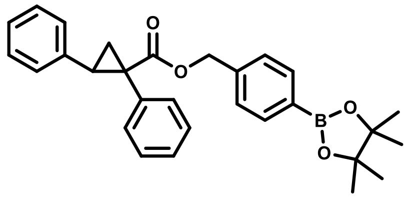
Synthesised according to general procedure F from hydrazone **2.24** (0.1745 g, 0.4 mmol) to afford cyclopropane **3.22** (0.0654 g, 36%) as a colourless liquid. Cyclopropane **3.22**: ^{1}H NMR (400 MHz, $CDCl_3$): δ_H 7.74-7.72 (m, 2H), 7.44-7.42 (m, 2H), 7.13-7.12 (m, 3H), 7.06-7.04 (m, 5H), 6.78-6.76 (m, 2H), 5.20-5.08 (m, 2H), 3.14-3.10 (dd, J = 9.43, 7.37 Hz, 1H), 2.17-2.14 (dd, J = 9.43, 4.91 Hz, 1H), 1.91-1.89 (dd, J = 7.37, 4.91 Hz, 1H), 1.33 (s, 12H); ^{13}C {^{1}H} NMR (101 MHz, $CDCl_3$): δ_C 135.0, 132.1, 129.4, 128.7, 128.2, 127.8, 126.4, 126.2, 84.0, 51.4, 45.3, 44.0, 25.0, 20.5; IR (υ, cm^{-1}, neat): 1725; HRMS (ESI): m/z calcd for $C_{29}H_{31}BO_4$: 455.2394 [M-H]$^-$; found: 455.2395

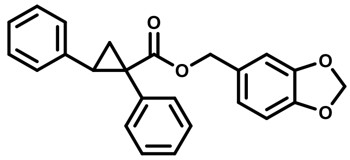
Synthesised according to general procedure F from hydrazone **2.25** (0.1418 g, 0.4 mmol) to afford cyclopropane **3.23** (0.1073 g, 72%) as a colourless liquid. Cyclopropane **3.23**: ^{1}H NMR (400 MHz, $CDCl_3$): δ_H 7.14-7.12 (m, 3H), 7.06-7.04 (m, 5H), 6.78-6.68 (m, 5H), 5.94 (s, 2H), 5.09-4.97 (m, 2H), 3.13-3.10 (dd, J = 9.44, 7.47 Hz, 1H), 2.16-2.13 (dd, J = 9.44, 4.83 Hz, 1H), 1.91-1.88 (dd, J = 7.47, 4.83 Hz, 1H); ^{13}C {^{1}H} NMR (101 MHz, $CDCl_3$): δ_C 173.7, 147.8, 147.5, 136.4, 134.8, 132.1, 130.1, 128.2, 127.8, 127.8, 127.2, 126.5, 121.4, 108.3, 101.2, 66.8, 37.7, 33.2, 20.5; IR (υ, cm^{-1}, neat): 2930, 1715; HRMS (ESI): m/z calcd for $C_{24}H_{20}O_4$: 373.1440 [M+H]$^+$; found: 373.1445

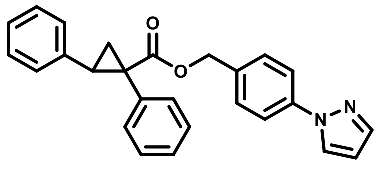
Synthesised according to general procedure F from hydrazone **2.38** (0.1506 g, 0.4 mmol) to afford cyclopropane **3.24** (0.0852 g, 54%) as a colourless liquid. Cyclopropane **3.24**: ^{1}H NMR (400 MHz, $CDCl_3$): δ_H 7.89-7.88 (d, J = 2.50 Hz, 1H), 7.71-7.70 (d, J = 1.65 Hz, 1H), 7.63-7.60 (m, 2H), 7.27-7.23 (m, 2H), 7.14-7.11 (m, 3H), 7.06-7.03 (m, 5H), 6.79-6.75 (m, 2H), 6.45-6.44 (t, J = 2.19 Hz, 1H), 5.20-5.07 (m, 2H), 3.15-3.11 (dd, J = 9.25, 7.24 Hz, 1H), 2.18-2.14 (dd, J = 9.25, 5.00 Hz, 1H), 1.92-1.89 (dd, J = 7.24, 5.00 Hz, 1H); ^{13}C {^{1}H} NMR (101 MHz, $CDCl_3$): δ_C 173.6, 141.3, 139.8, 136.3, 134.7, 134.5, 132.0, 128.6, 128.2, 127.8, 127.8, 127.2, 126.8, 126.5, 119.2, 107.8, 66.1, 37.7, 33.3, 20.5; IR (υ, cm^{-1}, neat): 3129, 1714; HRMS (ESI): m/z calcd for $C_{26}H_{22}N_2O_2$: 395.1760 [M+H]$^+$; found: 395.1765

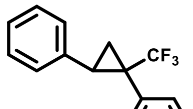
Synthesised according to general procedure F from hydrazone **2.8** (0.0977 g, 0.4 mmol) to afford cyclopropane **3.25** (0.0493 g, 47%) as a yellow oil. Cyclopropane **3.25**: ^{1}H NMR (400 MHz, $CDCl_3$): δ_H 7.20-7.12 (m, 5H), 7.09-7.07 (m, 3H), 6.78-6.76 (m, 2H), 2.86-2.82 (dd, J = 9.45, 6.75 Hz, 1H), 1.90-1.86 (dd, J = 9.66, 6.08 Hz, 1H), 1.70-1.66 (m, 1H); ^{13}C {^{1}H} NMR (101 MHz, $CDCl_3$): δ_C 135.8, 132.6, 131.6, 128.3, 128.1, 128.0, 128.0, 126.6, 25.8, 14.8; ^{19}F NMR (471 MHz, $CDCl_3$): δ -69.7; IR (υ, cm^{-1}, neat): 3184, 579; HRMS (ESI): m/z calcd for $C_{16}H_{13}F_3$: 263.1048 [M+H]$^+$; found: 263.1054

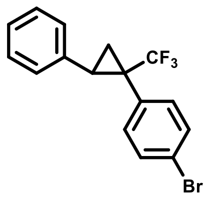
Synthesised according to general procedure F from hydrazone **2.9** (0.1293 g, 0.4 mmol) to afford cyclopropane **3.26** (0.0996 g, 73%) as a yellow oil. Cyclopropane **3.26**: ^{1}H NMR (400 MHz, $CDCl_3$): δ_H 7.31-7.28 (m, 2H), 7.14-7.10 (m, 3H), 7.01-6.99 (m, 2H), 6.80-6.77 (m, 2H), 2.87-2.83 (dd, J = 9.66, 6.94 Hz, 1H), 1.90-

1.86 (dd, J = 9.66, 6.15 Hz, 1H), 1.67-1.63 (m, 1H); ^{13}C {^1H} NMR (101 MHz, $CDCl_3$): δ_C 135.2, 134.2, 133.0, 131.4, 130.8, 128.2, 128.0, 126.9, 122.7, 28.6, 25.8, 14.7; ^{19}F NMR (471 MHz, $CDCl_3$): δ -69.7; IR (υ, cm^{-1}, neat): 3183, 580; HRMS (ESI): m/z calcd for $C_{16}H_{12}BrF_3$: 341.0153 $[M+H]^+$; found: 341.0179

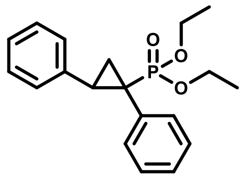

Synthesised according to general procedure F from hydrazone **2.14** (0.1249 g, 0.4 mmol) to afford cyclopropane **3.27** (0.0833 g, 63%) as a yellow oil. Cyclopropane **3.27**: ^1H NMR (400 MHz, $CDCl_3$): δ_H 7.12-7.11 (m, 3H), 7.08-7.05 (m, 5H), 6.76-6.73 (m, 2H), 4.11-4.02 (m, 4H), 3.04-2.96 (m, 1H), 2.10-2.02 (m, 1H), 1.74-1.68 (m, 1H), 1.30-1.27 (m, 3H), 1.25-1.22 (m, 3H); ^{13}C {^1H} NMR (101 MHz, $CDCl_3$): δ_C 132.5, 130.2, 129.4, 128.3, 128.1, 127.9, 127.8, 126.7, 126.3, 62.6, 55.3, 51.2, 28.9, 28.8, 16.5; ^{31}P NMR (202 MHz, $CDCl_3$): δ 27.0; IR (υ, cm^{-1}, neat): 2907; HRMS (ESI): m/z calcd for $C_{19}H_{23}O_3P$: 331.1463 $[M+H]^+$; found: 331.1467

Optimisation for electrochemical cyclopropanation of non-activated olefins

Table S7. Optimisation of electrolysis conditions for the electrosynthesis of cyclopropane 3.48

Reactions were carried out on a 0.4 mmol scale at room temperature (rt) in a 5 mL ElectraSyn cell equipped with carbon and stainless steel electrodes, and yields were determined by ^{1}H NMR using CH_2Br_2 as an internal standard.

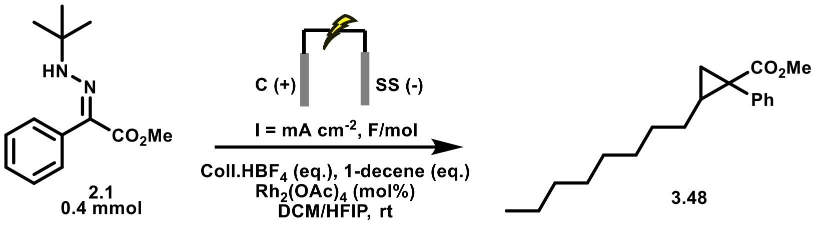

Entry	Coll. HBF_4 (eq.)	1-decene (eq.)	Rh_2OAc_4	DCM	HFIP	Current (mA)	F/mol	NMR Yield
1	0.5	10	1 mol %	5 mL	N/A	3	2	0%
2	0.5	10	1 mol %	5 mL	10 eq.	3	2	4%
**3**	**0.5**	**10**	**1 mol %**	**N/A**	**5 mL**	**3**	**2**	**48%**
4	0.5	5	1 mol %	N/A	5 mL	3	2	16%
5	0.5	10	1 mol %	N/A	5 mL	1	2	15%
6	0.5	10	1 mol %	N/A	5 mL	5	2	6%
7	0.5	10	1 mol %	N/A	5 mL	1	2.5	9%
8	0.5	10	N/A	N/A	5 mL	3	2	0%
9	0.5	10	1 mol %	N/A	5 mL	N/A	N/A	0%

General Procedure G for the electrochemical cyclopropanation of hydrazones with varying olefins

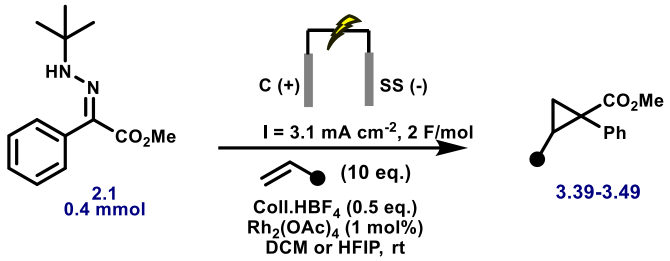

In a 5 mL ElectraSyn 2.0 vial, the hydrazone (0.4 mmol, 0.0937 g), Coll. HBF_4 (0.2 mmol, 0.0418 g, 0.5 eq.), olefin (4 mmol, 10 eq.), and rhodium (II) acetate (1 mol%) were dissolved in DCM (5 mL). The ElectraSyn 2.0 cap was equipped with a C_{gr} anode (working electrode) and a stainless steel cathode (counter electrode). The reaction conditions were set to 3.1 mA cm^{-2} and 2 F/mol. The reaction was stirred vigorously and electrolysed at room temperature. The mixture was quenched with water and extracted with EtOAc (3 x 50 mL). The combined organics were dried over $MgSO_4$ and the solvent removed under reduced pressure. The crude residue was purified by silica gel flash chromatography (Hexane/EtOAc = 9:1 to 7:3) to afford the desired cyclopropane. The procedure was repeated with different olefins of varying functionality.

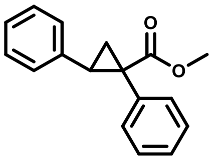

Synthesised according to general procedure G from styrene (0.4166 g, 4 mmol) and DCM as solvent to afford cyclopropane **3.1** (0.0979 g, 97%) as a colourless oil. Cyclopropane **3.1**: ^{1}H NMR (400 MHz, $CDCl_3$): δ_H 7.15-7.13 (m, 3H), 7.07-7.03 (m, 5H), 6.79-6.77 (m, 2H), 3.67 (s, 3H), 3.14-3.11 (dd, J = 9.35, 7.20 Hz, 1H), 2.17-2.14 (dd, J = 9.29, 4.74 Hz, 1H), 1.91-1.88 (dd, J = 7.34, 4.97 Hz, 1H); ^{13}C {^{1}H} NMR (101 MHz, $CDCl_3$): δ_C 174.5, 136.5, 134.8, 132.1, 128.2, 127.8, 127.2, 126.4, 52.8, 37.5, 33.3, 20.6; IR (υ, cm^{-1}, neat): 1716; HRMS (ESI): m/z calcd for $C_{17}H_{16}O_2$: 253.1229 $[M+H]^+$; found: 253.1219

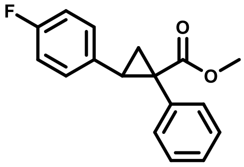

Synthesised according to general procedure G from 4-fluorostyrene (0.4886 g, 4 mmol) and DCM as solvent to afford cyclopropane **3.39** (0.0822 g, 76%) as a yellow oil. Cyclopropane **3.39**: ^{1}H NMR (400 MHz, $CDCl_3$): δ_H 7.15-7.13 (m, 3H), 7.02-7.00 (m, 2H), 6.77-6.71 (m, 4H), 3.66 (s, 3H), 3.11-3.07 (dd, J = 9.28, 7.09 Hz, 1H), 2.15-2.11 (dd, J = 9.28, 4.90 Hz, 1H), 1.84-1.81 (dd, J = 7.09, 4.90 Hz, 1H); ^{13}C {^{1}H} NMR (101 MHz, $CDCl_3$): δ_C 174.4, 162.8, 160.4, 134.7, 132.2, 132.0, 129.6, 129.5, 128.6, 127.9, 127.3, 126.7, 114.9, 114.6, 52.8, 37.4, 32.5, 20.7; ^{19}F NMR (471 MHz, $CDCl_3$): δ -116.4; IR (υ, cm^{-1}, neat): 1714; HRMS (ESI): m/z calcd for $C_{17}H_{15}FO_2$: 271.1239 $[M+H]^+$; found: 271.1233

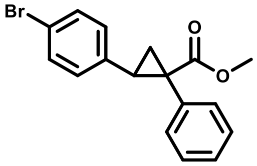

Synthesised according to general procedure G from 4-bromostyrene (0.7322 g, 4 mmol) and DCM as solvent to afford cyclopropane **3.40** (0.1073 g, 81%) as a colourless oil. Cyclopropane **3.40**: ^{1}H NMR (400 MHz, $CDCl_3$): δ_H 7.18-7.14 (m, 5H), 7.03-7.00 (m, 2H), 6.64-6.60 (m, 2H), 3.66 (s, 3H), 3.08-3.04 (dd, J = 9.44, 7.40 Hz, 1H), 2.16-2.12 (dd, J = 9.44, 4.85 Hz, 1H), 1.84-1.81 (dd, J = 7.40, 4.85 Hz, 1H); ^{13}C {^{1}H} NMR (101 MHz, $CDCl_3$): δ_C 174.2, 135.7, 134.4, 132.0, 130.9, 129.8, 128.0, 127.4, 120.3, 52.8, 37.6, 32.6, 20.8; IR (υ, cm^{-1}, neat): 1712; HRMS (ESI): m/z calcd for $C_{17}H_{15}BrO_2$: 331.0412 $[M+H]^+$; found: 331.0422

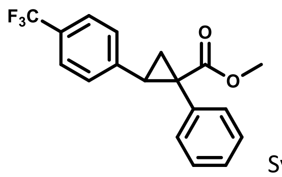
Synthesised according to general procedure G from 4-(trifluoromethyl)styrene (0.6886 g, 4 mmol) and DCM as solvent to afford cyclopropane **3.41** (0.0756 g, 59%) as a yellow oil. Cyclopropane **3.41**: ^{1}H NMR (400 MHz, $CDCl_3$): δ_H 7.32-7.29 (m, 2H), 7.16-7.13 (m, 3H), 7.02-7.00 (m, 2H), 6.86-6.83 (m, 2H), 3.67 (s, 3H), 3.17-3.13 (dd, J = 9.38, 7.21 Hz, 1H), 2.21-2.17 (dd, J = 9.38, 5.05 Hz, 1H), 1.91-1.88 (dd, J = 7.21, 5.05 Hz, 1H); ^{13}C {^{1}H} NMR (400 MHz, $CDCl_3$): δ_C 174.1, 141.0, 134.2, 131.9, 128.4, 128.1, 127.5, 124.8, 124.7, 124.7, 52.9, 38.1, 32.6, 21.0; ^{19}F NMR (471 MHz, $CDCl_3$): δ -62.3; IR (υ, cm^{-1}, neat): 1713; HRMS (ESI): m/z calcd for $C_{18}H_{15}F_3O_2$: 321.1112 $[M+H]^+$; found: 321.1111

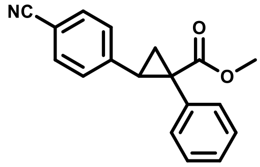
Synthesised according to general procedure G from 4-cyanostyrene (0.5166 g, 4 mmol) and DCM as solvent to afford cyclopropane **3.42** (0.0344 g, 31%) as a yellow oil. Cyclopropane **3.42**: ^{1}H NMR (400 MHz, $CDCl_3$): δ_H 7.34-7.32 (m, 2H), 7.17-7.13 (m, 3H), 7.00-6.98 (m, 2H), 6.84-6.82 (m, 2H), 3.67 (s, 3H), 3.15-3.12 (dd, J = 9.18, 7.29 Hz, 1H), 2.22-2.19 (dd, J = 9.18, 5.06 Hz, 1H), 1.92-1.90 (dd, J = 7.29, 5.06 Hz, 1H); ^{13}C {^{1}H} NMR (101 MHz, $CDCl_3$): δ_C 173.8, 142.6, 133.9, 132.9, 131.8, 131.6, 128.7, 128.2, 127.7, 126.7, 119.0, 110.1, 53.0, 38.4, 32.7, 21.1; IR (υ, cm^{-1}, neat): 2258, 1712; HRMS (ESI): m/z calcd for $C_{18}H_{15}NO_2$: 278.1264 $[M+H]^+$; found: 278.1257

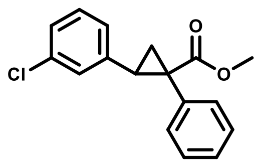
Synthesised according to general procedure G from 3-chlorostyrene (0.5544 g, 4 mmol) and DCM as solvent to afford cyclopropane **3.43** (0.1032 g, 90%) as a colourless liquid. Cyclopropane **3.43**: ^{1}H NMR (400 MHz, $CDCl_3$): δ_H 7.17-7.14 (m, 3H), 7.04-7.01 (m, 3H), 6.98-6.94 (t, J = 7.88 Hz, 1H), 6.80-6.79 (t, J = 2.09 Hz, 1H), 6.60-6.57 (m, 1H), 3.67 (s, 3H), 3.09-3.05 (dd, J = 9.48, 7.38 Hz, 1H), 2.16-2.12 (dd, J = 9.48, 5.29 Hz, 1H), 1.87-1.84 (dd, J = 7.38, 5.29 Hz, 1H); ^{13}C {^{1}H} NMR (101 MHz, $CDCl_3$): δ_C 174.2, 138.8, 134.4, 132.0, 129.0, 128.5, 128.0, 127.4, 126.6, 126.1, 52.9, 37.7, 32.6, 20.7; IR (υ, cm^{-1}, neat): 1718; HRMS (ESI): m/z calcd for $C_{17}H_{15}ClO_2$: 287.0839 $[M+H]^+$; found: 287.0851

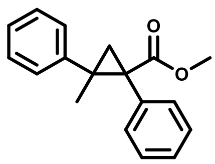
Synthesised according to general procedure G from alpha-methylstyrene (0.4727 g, 4 mmol) and DCM as solvent to afford cyclopropane **3.44** (0.1012 g, 95%) as a colourless liquid. Cyclopropane **3.44**: ^{1}H NMR (400 MHz, $CDCl_3$): δ_H 7.18-7.15 (m, 2H), 7.08-7.00 (m, 8H), 3.71 (s, 3H), 2.11-2.10 (d, J = 5.52 Hz, 1H), 1.95-1.94 (d, J = 5.52 Hz, 1H), 1.67 (s, 3H); ^{13}C {^{1}H} NMR (101 MHz, $CDCl_3$): δ_C 172.3, 141.1, 136.6, 131.5, 128.1, 127.8, 127.4, 126.7, 126.3, 52.5, 42.2, 34.0, 22.7, 22.4; IR (υ, cm^{-1}, neat): 1709; HRMS (ESI): m/z calcd for $C_{18}H_{18}O_2$: 267.1440 $[M+H]^+$; found: 267.1439

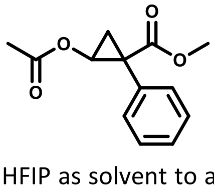
Synthesised according to general procedure G from vinyl acetate (0.3444 g, 4 mmol) and HFIP as solvent to afford cyclopropane **3.45** (0.0300 g, 32%) as a colourless liquid. Cyclopropane **3.45**: ^{1}H NMR (400 MHz, $CDCl_3$): δ_H 7.33-7.24 (m, 5H), 4.81-4.79 (dd, J = 7.12, 4.34 Hz, 1H), 3.65 (s, 3H), 1.98-1.94 (dd, J = 7.12, 6.31 Hz, 1H), 1.78 (s, 3H), 1.73-1.70 (dd, J = 6.31, 4.34 Hz, 1H); ^{13}C {^{1}H} NMR (101 MHz, $CDCl_3$): δ_C 172.7, 171.2, 133.8, 131.3, 129.0, 128.3, 127.8, 58.0, 52.8, 34.3, 20.7, 19.4; IR (υ, cm^{-1}, neat): 1713, 1711; HRMS (ESI): m/z calcd for $C_{13}H_{14}O_4$: 235.1010 $[M+H]^+$; found: 235.1022

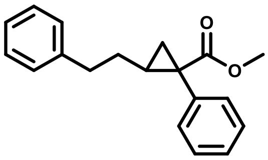

Synthesised according to general procedure G from 4-phenyl-1-butene (0.5288 g, 4 mmol) and HFIP as solvent to afford cyclopropane **3.46** (0.0740 g, 66%) as a colourless liquid. Cyclopropane **3.46**: ^{1}H NMR (400 MHz, $CDCl_3$): δ_H 7.32-7.21 (m, 7H), 7.16-7.13 (m, 1H), 7.07-7.05 (m, 2H), 3.61 (s, 3H), 2.70-2.64 (m, 2H), 1.91-1.86 (m, 1H), 1.71-1.69 (dd, J = 9.24, 4.55 Hz, 1H), 1.11-1.09 (dd, J = 6.90, 4.55 Hz, 1H), 0.90-0.83 (m, 2H); ^{13}C {^{1}H} NMR (101 MHz, $CDCl_3$): δ_C 175.2, 141.9, 136.2, 131.4, 128.5, 128.4, 128.2, 127.3, 126.0, 52.5, 35.5, 33.9, 32.5, 28.4, 21.6; IR (υ, cm^{-1}, neat): 1709; HRMS (ESI): m/z calcd for $C_{19}H_{20}O_2$: 281.1675 $[M+H]^+$; found: 281.1684

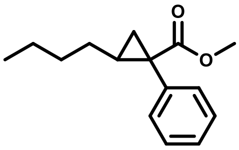

Synthesised according to general procedure G from 1-hexene (0.3366 g, 4 mmol) and HFIP as solvent to afford cyclopropane **3.47** (0.0269 g, 29%) as a colourless liquid. Cyclopropane **3.47**: ^{1}H NMR (400 MHz, $CDCl_3$): δ_H 7.56-7.54 (m, 2H), 7.34-7.30 (m, 3H), 3.79 (s, 3H), 2.46-2.28 (m, 2H), 1.72-1.66 (m, 1H), 1.28-1.25 (m, 6H), 0.90-0.86 (m, 3H); ^{13}C {^{1}H} NMR (101 MHz, $CDCl_3$): δ_C 164.2, 131.5, 128.3, 127.9, 126.7, 55.3, 51.2, 32.0, 29.5, 29.2, 28.9, 22.8, 14.2; IR (υ, cm^{-1}, neat): 1708; HRMS (ESI): m/z calcd for $C_{15}H_{20}O_2$: 233.1604 $[M+H]^+$; found: 233.1603

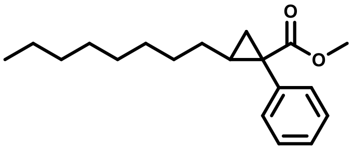

Synthesised according to general procedure G from 1-decene (0.5611 g, 4 mmol) and HFIP as solvent to afford cyclopropane **3.48** (0.0554 g, 48%) as a colourless liquid. Cyclopropane **3.48**: ^{1}H NMR (400 MHz, $CDCl_3$): δ_H 7.56-7.54 (m, 2H), 7.35-7.29 (m, 3H), 3.79 (s, 3H), 2.47-2.28 (m, 2H). 1.74-1.68 (m, 1H), 1.30-1.24 (m, 14H), 0.90-0.86 (m, 3H); ^{13}C {^{1}H} NMR (101 MHz, $CDCl_3$): δ_C 164.2, 131.5, 128.3, 127.9, 126.7, 55.3, 52.4, 51.2, 32.0, 29.5, 29.4, 29.2, 28.9, 22.8, 21.8, 14.2; IR (υ, cm^{-1}, neat): 1712; HRMS (ESI): m/z calcd for $C_{19}H_{28}O_2$: 289.2242 $[M+H]^+$; found: 289.2245

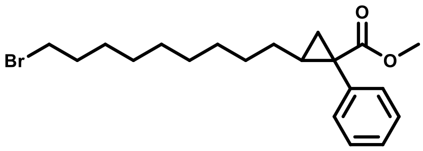

Synthesised according to general procedure G from 11-bromo-1-undecene (0.9328 g, 4 mmol) and HFIP as solvent to afford cyclopropane **3.49** (0.0564 g, 37%) as a colourless liquid. Cyclopropane **3.49**: ^{1}H NMR (400 MHz, $CDCl_3$): δ_H 7.56-7.54 (m, 1H), 7.34-7.30 (m, 4H), 3.79 (s, 3H), 3.42-3.39 (t, J = 6.79 Hz, 2H), 2.04-1.97 (m, 2H), 1.72-1.66 (m, 1H), 1.30-1.25 (m, 16H); ^{13}C {^{1}H} NMR (101 MHz, $CDCl_3$): δ_C 168.0, 131.5, 128.3, 127.9, 34.2, 33.0, 29.7, 28.9, 28.3; IR (υ, cm^{-1}, neat): 1708; HRMS (ESI): m/z calcd for $C_{20}H_{29}BrO_2$: 381.1564 $[M+H]^+$; found: 381.1569

General Procedure H for the electrosynthesis of stabilised diazo compounds

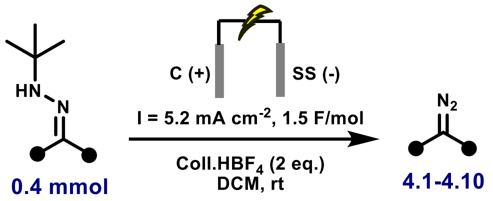

In a 5 mL ElectraSyn 2.0 vial, the hydrazone (0.4 mmol) and coll. HBF_4 (0.8 mmol, 0.1672 g, 2 eq.) were dissolved in DCM (5 mL). The ElectraSyn 2.0 cap was equipped with a C_{gr} anode (working electrode) and a stainless steel cathode (counter electrode). The reaction conditions were set to 5.2 mA cm^{-2} and 1.5 F/mol. The reaction was stirred vigorously and electrolysed at room temperature. The mixture was quenched with water and extracted with EtOAc (3 x 50 mL). The combined organics were dried over $MgSO_4$ and the solvent removed under reduced pressure. The crude residue was purified by silica gel flash chromatography (Hexane/EtOAc = 9:1 to 7:3) to afford the desired diazo compound. The procedure was repeated with different hydrazones of varying functionality.

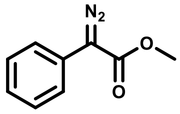

Synthesised according to general procedure H from hydrazone **2.1** (0.0937 g, 0.4 mmol) to afford cyclopropane **4.1** (0.0529 g, 75%) as a yellow oil. Diazo **4.1**: ^{1}H NMR (400 MHz, $CDCl_3$): δ_H 7.50-7.47 (m, 2H), 7.41-7.37 (m, 2H), 7.21-7.17 (m, 1H), 3.87 (s, 3H); ^{13}C {^{1}H} NMR (101 MHz, $CDCl_3$): δ_C 165.8, 129.1, 126.0, 125.6, 124.1, 52.1; IR (υ, cm^{-1}, neat): 2966, 2818, 2078, 1708; HRMS (ESI): m/z calcd for $C_9H_8N_2O_2$: 177.0659 $[M+H]^+$; found: 177.0656

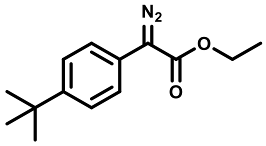

Synthesised according to general procedure H from hydrazone **2.13** (0.1162 g, 0.4 mmol) to afford diazo **4.2** (0.0502 g, 51%) as a yellow oil. Diazo **4.2**: ^{1}H NMR (400 MHz, $CDCl_3$): δ_H 7.42 (s, 4H), 4.36-4.31 (q, J = 7.21, 2H), 1.36-1.34 (t, J = 7.21 Hz, 3H), 1.33 (s, 9H); ^{13}C {^{1}H} NMR (101 MHz, $CDCl_3$): δ_C 165.6, 149.1, 126.1, 124.1, 122.5, 61.1, 34.6, 31.4, 14.6; IR (υ, cm^{-1}, neat): 2964, 2084, 1705; HRMS (ESI): m/z calcd for $C_{14}H_{18}N_2O_2$: 247.1447 $[M+H]^+$; found: 247.1445

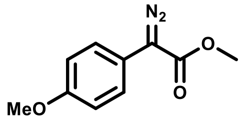

Synthesised according to general procedure H from hydrazone **2.5** (0.1057 g, 0.4 mmol) to afford diazo **4.3** (0.0256 g, 31%) as a yellow oil. Diazo **4.3**: ^{1}H NMR (400 MHz, $CDCl_3$): δ_H 7.40-7.36 (m, 2H), 6.96-6.92 (m, 2H), 4.35-4.29 (q, J = 6.94 Hz, 2H), 3.81 (s, 3H), 1.35-1.31 (t, J = 6.94 Hz, 3H); ^{13}C {^{1}H} NMR (101 MHz, $CDCl_3$): δ_C 165.9, 158.1, 126.1, 117.2, 114.7, 61.1, 55.5, 14.7; IR (υ, cm^{-1}, neat): 2923, 2854, 2254, 1735, 1676; HRMS (ESI): m/z calcd for $C_{11}H_{12}N_2O_3$: 243.0746 $[M+Na]^+$; found: 243.0739

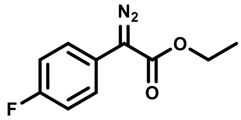

Synthesised according to general procedure H from hydrazone **2.3** (0.1009 g, 0.4 mmol) to afford diazo **4.4** (0.0350 g, 42%) as a yellow oil. Diazo **4.4**: ^{1}H NMR (400 MHz, $CDCl_3$): δ_H 7.46-7.43 (m, 2H), 7.12-7.07 (m, 2H), 4.36-4.30 (q, J = 7.26 Hz, 2H), 1.36-1.32 (t, J = 7.26 Hz, 3H); ^{13}C {^{1}H} NMR (101 MHz, $CDCl_3$): δ_C 165.4, 162.4, 126.1, 126.0, 121.6, 116.3, 116.0, 61.2, 14.6; ^{19}F NMR (471 MHz, $CDCl_3$): δ -116.3; IR (υ, cm^{-1}, neat): 3280, 3165, 2945, 2873, 2255, 1624; HRMS (ESI): m/z calcd for $C_{10}H_9FN_2O_2$: 209.0716 $[M+H]^+$; found: 209.0728

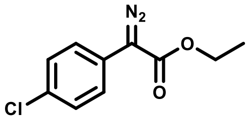

Synthesised according to general procedure H from hydrazone **2.2** (0.1075 g, 0.4 mmol) to afford diazo **4.5** (0.0458 g, 51%) as a yellow oil. Diazo **4.5**: ^{1}H NMR (400 MHz, $CDCl_3$): δ_H 7.43-7.40 (m, 2H), 7.35-7.32 (m, 2H), 4.35-4.30 (q, J = 7.31 Hz, 2H), 1.35-1.32 (t, J = 7.31 Hz, 3H); ^{13}C {^{1}H} NMR (101 MHz, $CDCl_3$): δ_C 165.0, 131.5, 129.2, 125.1, 124.4, 61.3, 14.6; IR (υ, cm^{-1}, neat): 2976, 2922, 2868, 2112, 1680; HRMS (ESI): m/z calcd for $C_{10}H_9ClN_2O_2$: 225.0561 [M+H]$^+$; found: 225.0568

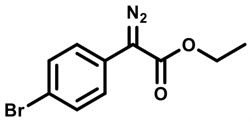

Synthesised according to general procedure H from hydrazone **2.4** (0.1253 g, 0.4 mmol) to afford diazo **4.6** (0.0431 g, 40%) as a yellow oil. Diazo **4.6**: ^{1}H NMR (400 MHz, $CDCl_3$): δ_H 7.50-7.47 (m, 2H), 7.38-7.34 (m, 2H), 4.36-4.30 (q, J = 7.14 Hz, 2H), 1.35-1.32 (t, J = 7.14 Hz, 3H); ^{13}C {^{1}H} NMR (101 MHz, $CDCl_3$): δ_C 165.0, 132.1, 125.4, 125.0, 119.4, 61.3, 14.6; IR (υ, cm^{-1}, neat): 3333, 3263, 2982, 2089, 1737; HRMS (ESI): m/z calcd for $C_{10}H_9BrN_2O_2$: 268.9920 [M+H]$^+$; found: 268.9915

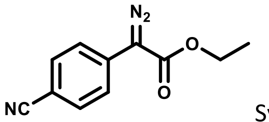

Synthesised according to general procedure H from hydrazone **2.22** (0.1037 g, 0.4 mmol) to afford diazo **4.7** (0.0482 g, 56%) as a yellow oil. Diazo **4.7**: ^{1}H NMR (400 MHz, $CDCl_3$): δ_H 7.65-7.59 (m, 4H), 4.38-4.33 (q, J = 7.04 Hz, 2H), 1.37-1.33 (t, J = 7.04 Hz, 3H); ^{13}C {^{1}H} NMR (101 MHz, $CDCl_3$): δ_C 164.0, 132.7, 131.9, 123.5, 118.9, 108.7, 61.6, 14.6; IR (υ, cm^{-1}, neat): 2925, 2231, 2094, 1704; HRMS (ESI): m/z calcd for $C_{11}H_9N_3O_2$: 216.0633 [M+H]$^+$; found: 216.0625

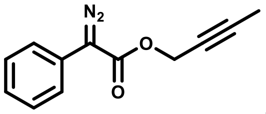

Synthesised according to general procedure H from hydrazone **2.18** (0.1089 g, 0.4 mmol) to afford diazo **4.8** (0.0334 g, 39%) as a yellow oil. Diazo **4.8**: ^{1}H NMR (400 MHz, $CDCl_3$): δ_H 7.50-7.47 (m, 2H), 7.41-7.37 (m, 2H), 7.21-7.17 (m, 1H), 4.85-4.83 (q, J = 2.50 Hz, 2H), 1.88-1.87 (t, J = 7.04 Hz, 3H); ^{13}C {^{1}H} NMR (101 MHz, $CDCl_3$): δ_C 164.7, 129.1, 126.1, 125.4, 124.1, 83.6, 73.3, 53.2, 3.8; IR (υ, cm^{-1}, neat): 2945, 2857, 2065, 1713; HRMS (ESI): m/z calcd for $C_{12}H_{10}N_2O_2$: 215.0821 [M+H]$^+$; found: 215.0824

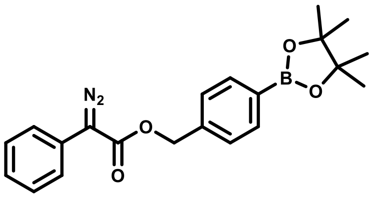

Synthesised according to general procedure H from hydrazone **2.24** (0.1745 g, 0.4 mmol) to afford diazo **4.9** (0.0393 g, 26%) as a yellow oil. Diazo **4.9**: ^{1}H NMR (400 MHz, $CDCl_3$): δ_H 7.84-7.82 (m, 2H), 7.50-7.48 (m, 2H), 7.41-7.36 (m, 4H), 7.21-7.17 (m, 1H), 5.53 (s, 2H), 1.35 (s, 12H); ^{13}C {^{1}H} NMR (101 MHz, $CDCl_3$): δ_C 165.1, 139.0, 135.2, 129.1, 127.4, 126.0, 125.5, 124.2, 84.0, 66.5, 25.0; IR (υ, cm^{-1}, neat): 3201, 3176, 3047, 2983, 2942, 1754; HRMS (ESI): m/z calcd for $C_{21}H_{23}BN_2O_4$: 379.1922 [M+H]$^+$; found: 379.1928

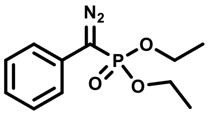

Synthesised according to general procedure H from hydrazone **2.14** (0.1249 g, 0.4 mmol) to afford diazo **4.10** (0.0569 g, 56%) as a yellow oil. Diazo **4.10**: ^{1}H NMR (400 MHz, $CDCl_3$): δ_H 7.38-7.33 (m, 2H), 7.19-7.12 (m, 3H), 4.27-4.09 (m, 4H), 1.36-1.32 (t, J = 7.07 Hz, 6H); ^{13}C {^{1}H} NMR (101 MHz, $CDCl_3$): δ_C 129.4, 125.5, 122.8, 122.8, 63.1, 63.0, 16.3, 16.3; ^{31}P NMR (202 MHz, $CDCl_3$): δ 17.9; IR (υ, cm^{-1}, neat): 2946, 2850, 2082, 1794, 1602; HRMS (ESI): m/z calcd for $C_{11}H_{15}N_2O_3P$: 255.0916 [M+H]$^+$; found: 255.0917

General Procedure I for the electrochemical cyclopropanation of activated hydrazones with decreased olefin concentration

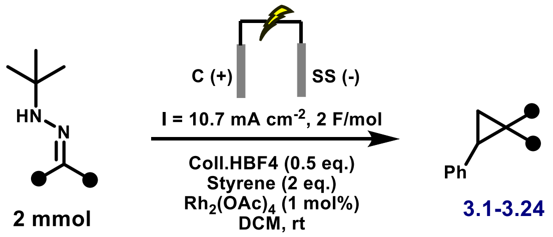

In a 5 mL ElectraSyn 2.0 vial, the hydrazone (2 mmol, 1 eq., 0.4 M), Coll HBF_4 (1 mmol, 0.5 eq., 0.2 M), styrene (4 mmol, 2 eq., 0.8 M), and rhodium (II) acetate (1 mol%, 0.004 M) were dissolved in DCM (5 mL). The ElectraSyn 2.0 cap was equipped with a C_{gr} anode (working electrode) and a stainless steel cathode (counter electrode). The reaction conditions were set to 10.7 mA cm^{-2} and 2 F/mol. The reaction was stirred vigorously and electrolysed at room temperature. The mixture was quenched with water and extracted with EtOAc (3 x 50 mL). The combined organics were dried over $MgSO_4$ and the solvent removed under reduced pressure. The crude residue was purified by silica gel flash chromatography (Hexane/EtOAc = 9:1 to 7:3) to afford the desired cyclopropane. The procedure was repeated with different hydrazones of varying functionality.

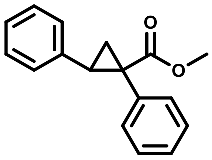

Synthesised according to general procedure I from hydrazone **2.1** (0.4686 g, 2 mmol) to afford cyclopropane **3.1** (0.3532 g, 70%) as a colourless oil. Cyclopropane **3.1**: ^{1}H NMR (400 MHz, $CDCl_3$): δ_H 7.15-7.13 (m, 3H), 7.07-7.03 (m, 5H), 6.79-6.77 (m, 2H), 3.67 (s, 3H), 3.14-3.11 (dd, J = 9.35, 7.20 Hz, 1H), 2.17-2.14 (dd, J = 9.29, 4.74 Hz, 1H), 1.91-1.88 (dd, J = 7.34, 4.97 Hz, 1H); ^{13}C {^{1}H} NMR (101 MHz, $CDCl_3$): δ_C 174.5, 136.5, 134.8, 132.1, 128.2, 127.8, 127.2, 126.4, 52.8, 37.5, 33.3, 20.6; IR (υ, cm^{-1}, neat): 1716; HRMS (ESI): m/z calcd for $C_{17}H_{16}O_2$: 253.1229 $[M+H]^+$; found: 253.1219

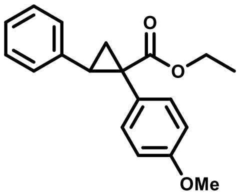

Synthesised according to general procedure I from hydrazone **2.5** (0.5567 g, 2 mmol) to afford cyclopropane **3.4** (0.3557 g, 60%) as a yellow oil. Cyclopropane **3.4**: ^{1}H NMR (400 MHz, $CDCl_3$): δ_H 7.07-7.06 (m, 3H), 6.94-6.93 (m, 2H), 6.79-6.77 (m, 2H), 6.67-6.65 (m, 2H), 4.18-4.08 (m, 2H), 3.72 (s, 3H), 3.07-3.04 (dd, J = 9.09, 7.18 Hz, 1H), 2.13-2.10 (dd, J = 9.33, 5.02 Hz, 1H), 1.83-1.80 (dd, J = 7.18, 4.79 Hz, 1H), 1.20-1.17 (t, J = 7.18 Hz, 3H); ^{13}C {^{1}H} NMR (101 MHz, $CDCl_3$): δ_C 174.2, 158.5, 136.8, 133.0, 128.2, 127.8, 127.1, 126.3, 113.2, 61.3, 55.2, 37.0, 33.1, 20.6, 14.3; IR (υ, cm^{-1}, neat): 1717; HRMS (ESI): m/z calcd for $C_{19}H_{20}O_3$: 297.1491 $[M+H]^+$; found: 297.1492

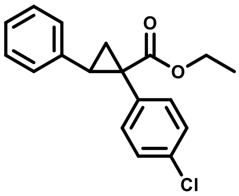

Synthesised according to general procedure I from hydrazone **2.2** (0.5655 g, 2 mmol) to afford cyclopropane **3.6** (0.4030 g, 67%) as a colourless liquid. Cyclopropane **3.6**: ^{1}H NMR (400 MHz, $CDCl_3$): δ_H 7.10-7.07 (m, 5H), 6.96-6.95 (m, 2H), 6.79-6.77 (m, 2H), 4.18-4.09 (m, 2H), 3.12-3.09 (dd, J = 9.68, 7.37 Hz, 1H), 2.15-2.12 (dd, J = 9.22, 4.95 Hz, 1H), 1.85-1.83 (dd, J = 7.37, 5.07 Hz, 1H), 1.20-1.17 (t, J = 7.37 Hz, 3H); ^{13}C {^{1}H} NMR (101 MHz, $CDCl_3$): δ_C 173.4, 136.1, 133.7, 133.3, 132.9, 128.1, 128.1, 128.0, 126.6, 61.5, 37.0, 33.1, 20.2, 14.3; IR (υ, cm^{-1}, neat): 1717; HRMS (ESI): m/z calcd for $C_{18}H_{17}ClO_2$: 301.0995 $[M+H]^+$; found: 301.0984

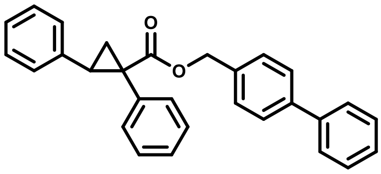

Synthesised according to general procedure I from hydrazone **2.31** (0.7730 g, 2 mmol) to afford cyclopropane **3.16** (0.3399 g, 42%) as a colourless liquid. Cyclopropane **3.16**: ^{1}H NMR (400 MHz, $CDCl_3$): δ_H 7.60-7.58 (m, 2H), 7.55-7.54 (m, 2H), 7.46-7.43 (m, 2H), 7.38-7.34 (m, 1H), 7.28-7.26 (m, 2H), 7.16-7.15 (m, 3H), 7.09-7.06 (m, 5H), 6.80-6.78 (m, 2H), 5.25-5.13 (m, 2H), 3.18-3.15 (dd, J = 9.42, 7.19 Hz, 1H), 2.21-2.19 (dd, J = 9.42, 4.86 Hz, 1H), 1.95-1.92 (dd, J = 7.48, 4.86 Hz, 1H); ^{13}C {^{1}H} NMR (400 MHz, $CDCl_3$): δ_C 173.7, 140.9, 140.8, 136.4, 135.3, 134.8, 132.1, 128.9, 128.2, 127.9, 127.8, 127.5, 127.3, 127.2, 127.2, 126.5, 66.5, 37.7, 33.2, 20.5; IR (υ, cm^{-1}, neat): 1713; HRMS (ESI): m/z calcd for $C_{29}H_{24}O_2$: 405.1855 [M+H]$^+$; found: 405.1856

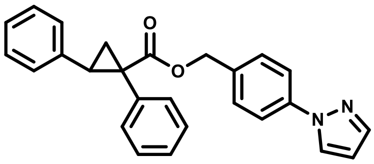

Synthesised according to general procedure I from hydrazone **2.38** (0.7529 g, 2 mmol) to afford cyclopropane **3.24** (0.3157 g, 40%) as a colourless liquid. Cyclopropane **3.24**: ^{1}H NMR (400 MHz, $CDCl_3$): δ_H 7.89-7.88 (d, J = 2.50 Hz, 1H), 7.71-7.70 (d, J = 1.65 Hz, 1H), 7.63-7.60 (m, 2H), 7.27-7.23 (m, 2H), 7.14-7.11 (m, 3H), 7.06-7.03 (m, 5H), 6.79-6.75 (m, 2H), 6.45-6.44 (t, J = 2.19 Hz, 1H), 5.20-5.07 (m, 2H), 3.15-3.11 (dd, J = 9.25, 7.24 Hz, 1H), 2.18-2.14 (dd, J = 9.25, 5.00 Hz, 1H), 1.92-1.89 (dd, J = 7.24, 5.00 Hz, 1H); ^{13}C {^{1}H} NMR (101 MHz, $CDCl_3$): δ_C 173.6, 141.3, 139.8, 136.3, 134.7, 134.5, 132.0, 128.6, 128.2, 127.8, 127.8, 127.2, 126.8, 126.5, 119.2, 107.8, 66.1, 37.7, 33.3, 20.5; IR (υ, cm^{-1}, neat): 3129, 1714; HRMS (ESI): m/z calcd for $C_{26}H_{22}N_2O_2$: 395.1760 [M+H]$^+$; found: 395.1765

General Procedure J for the electrochemical cyclopropanation of activated hydrazones in flow

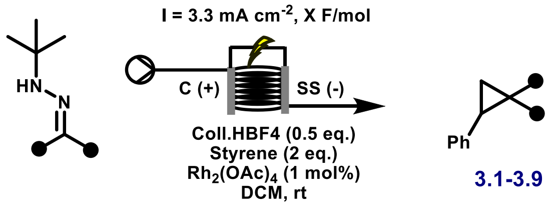

A solution of substrate (1 eq., 0.4 M), styrene (2 eq., 0.8 M), $Rh_2(OAc)_4$ (1% mol, 0.004 M) and Coll HBF_4 (0.5 eq. 0.2M) was pumped at 0.1 mL min^{-1} through an Asia® FLUX reactor (225 µL, 3.3 mA cm^{-2}) followed by an Asia® Pressure Controller set at 2.5 bar. The reaction mixture was left recirculating until full conversion was achieved. The mixture was extracted with EtOAc (3 x 50 mL). The combined organics were dried over $MgSO_4$ and the solvent removed under reduced pressure. The crude residue was purified by silica gel flash chromatography (Hexane/EtOAc = 9:1 to 7:3) to afford the desired cyclopropane. The procedure was repeated with different hydrazones of varying functionality.

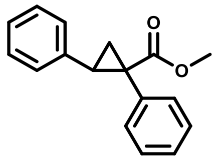

Synthesised according to general procedure J from hydrazone **2.1** (0.4686 g, 2 mmol) to afford cyclopropane **3.1** (0.3078 g, 61%) as a colourless oil. Cyclopropane **3.1**: ^1H NMR (400 MHz, $CDCl_3$): δ_H 7.15-7.13 (m, 3H), 7.07-7.03 (m, 5H), 6.79-6.77 (m, 2H), 3.67 (s, 3H), 3.14-3.11 (dd, J = 9.35, 7.20 Hz, 1H), 2.17-2.14 (dd, J = 9.29, 4.74 Hz, 1H), 1.91-1.88 (dd, J = 7.34, 4.97 Hz, 1H); ^{13}C {^1H} NMR (101 MHz, $CDCl_3$): δ_C 174.5, 136.5, 134.8, 132.1, 128.2, 127.8, 127.2, 126.4, 52.8, 37.5, 33.3, 20.6; IR (υ, cm^{-1}, neat): 1716; HRMS (ESI): m/z calcd for $C_{17}H_{16}O_2$: 253.1229 $[M+H]^+$; found: 253.1219

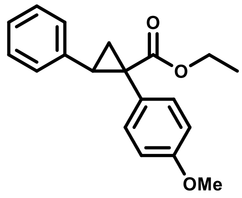

Synthesised according to general procedure J from hydrazone **2.5** (0.5567 g, 2 mmol) to afford cyclopropane **3.4** (0.2017 g, 34%) as a yellow oil. Cyclopropane **3.4**: ^1H NMR (400 MHz, $CDCl_3$): δ_H 7.07-7.06 (m, 3H), 6.94-6.93 (m, 2H), 6.79-6.77 (m, 2H), 6.67-6.65 (m, 2H), 4.18-4.08 (m, 2H), 3.72 (s, 3H), 3.07-3.04 (dd, J = 9.09, 7.18 Hz, 1H), 2.13-2.10 (dd, J = 9.33, 5.02 Hz, 1H), 1.83-1.80 (dd, J = 7.18, 4.79 Hz, 1H), 1.20-1.17 (t, J = 7.18 Hz, 3H); ^{13}C {^1H} NMR (101 MHz, $CDCl_3$): δ_C 174.2, 158.5, 136.8, 133.0, 128.2, 127.8, 127.1, 126.3, 113.2, 61.3, 55.2, 37.0, 33.1, 20.6, 14.3; IR (υ, cm^{-1}, neat): 1717; HRMS (ESI): m/z calcd for $C_{19}H_{20}O_3$: 297.1491 $[M+H]^+$; found: 297.1492

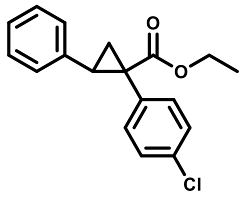

Synthesised according to general procedure J from hydrazone **2.2** (0.5655 g, 2 mmol) to afford cyclopropane **3.6** (0.4091 g, 68%) as a colourless liquid. Cyclopropane **3.6**: ^1H NMR (400 MHz, $CDCl_3$): δ_H 7.10-7.07 (m, 5H), 6.96-6.95 (m, 2H), 6.79-6.77 (m, 2H), 4.18-4.09 (m, 2H), 3.12-3.09 (dd, J = 9.68, 7.37 Hz, 1H), 2.15-2.12 (dd, J = 9.22, 4.95 Hz, 1H), 1.85-1.83 (dd, J = 7.37, 5.07 Hz, 1H), 1.20-1.17 (t, J = 7.37 Hz, 3H); ^{13}C {^1H} NMR (101 MHz, $CDCl_3$): δ_C 173.4, 136.1, 133.7, 133.3, 132.9, 128.1, 128.1, 128.0, 126.6, 61.5, 37.0, 33.1, 20.2, 14.3; IR (υ, cm^{-1}, neat): 1717; HRMS (ESI): m/z calcd for $C_{18}H_{17}ClO_2$: 301.0995 $[M+H]^+$; found: 301.0984

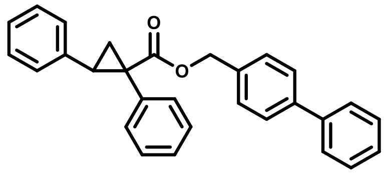Synthesised according to general procedure J from hydrazone **2.31** (0.7730 g, 2 mmol) to afford cyclopropane **3.16** (0.2104 g, 26%) as a colourless liquid. Cyclopropane **3.16**: ^{1}H NMR (400 MHz, $CDCl_3$): δ_H 7.60-7.58 (m, 2H), 7.55-7.54 (m, 2H), 7.46-7.43 (m, 2H), 7.38-7.34 (m, 1H), 7.28-7.26 (m, 2H), 7.16-7.15 (m, 3H), 7.09-7.06 (m, 5H), 6.80-6.78 (m, 2H), 5.25-5.13 (m, 2H), 3.18-3.15 (dd, J = 9.42, 7.19 Hz, 1H), 2.21-2.19 (dd, J = 9.42, 4.86 Hz, 1H), 1.95-1.92 (dd, J = 7.48, 4.86 Hz, 1H); ^{13}C {^{1}H} NMR (400 MHz, $CDCl_3$): δ_C 173.7, 140.9, 140.8, 136.4, 135.3, 134.8, 132.1, 128.9, 128.2, 127.9, 127.8, 127.5, 127.3, 127.2, 127.2, 126.5, 66.5, 37.7, 33.2, 20.5; IR (υ, cm^{-1}, neat): 1713; HRMS (ESI): m/z calcd for $C_{29}H_{24}O_2$: 405.1855 [M+H]$^+$; found: 405.1856

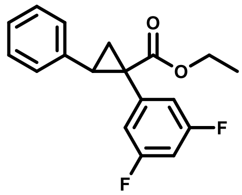Synthesised according to general procedure J from hydrazone **2.6** (0.5686 g, 2 mmol) to afford cyclopropane **3.9** (0.2117 g, 35%) as a colourless liquid. Cyclopropane **3.9**: ^{1}H NMR (400 MHz, $CDCl_3$): δ_H 7.12-7.09 (m, 3H), 6.84-6.81 (m, 2H), 6.59-6.55 (m, 3H), 4.21-4.11 (m, 2H), 3.16-3.12 (dd, J = 9.49, 7.38 Hz, 1H), 2.14-2.11 (dd, J = 9.24, 5.19 Hz, 1H), 1.88-1.85 (dd, J = 7.38, 5.19 Hz, 1H), 1.20-1.16 (t, J = 7.05 Hz, 3H); ^{13}C {^{1}H} NMR (101 MHz, $CDCl_3$): δ_C 172.7, 163.4, 163.3, 161.4, 161.3, 139.0, 139.0, 135.5, 128.5, 128.1, 128.0, 126.9, 126.1, 115.0, 115.0, 114.8, 114.8, 103.0, 102.8, 102.6, 61.7, 37.1, 37.1, 33.3, 20.0, 14.2; ^{19}F NMR (471 MHz, $CDCl_3$): δ -111.1; IR (υ, cm^{-1}, neat): 1716; HRMS (ESI): m/z calcd for $C_{18}H_{16}F_2O_2$: 303.1197 [M+H]$^+$; found: 303.1177

Faradaic Efficiency

$$FE\ (\%) = \frac{n \times F \times \eta_{produit}}{Q_{total}} \times 100 = \frac{2 \times 96485 \times 0.97}{3 \times 96485} \times 100 = 0.647$$

Assuming a two-electron process, passage of 3 F mol^{-1} corresponds to a theoretical maximum Faradaic efficiency of 67%. Based on the isolated yield (97%), a Faradaic efficiency of 65% was obtained for the formation of **3.1**, using the method presented in Figure 3.

Table S8. Faradaic efficiency (FE) of electrochemical cyclopropanation, calculated for the formation of compounds 3.1 to 3.27.

Compound	Yield (%)	FE (%)
**3.1**	97	64.7
**3.2**	77	51.3
**3.3**	69	46.0
**3.4**	84	56.0
**3.5**	71	47.3
**3.6**	83	55.3
**3.7**	58	38.7
**3.8**	81	54.0
**3.9**	81	54.0
**3.10**	58	38.7
**3.11**	84	56.0
**3.12**	77	51.3
**3.13**	17	11.3
**3.14**	99	66.0
**3.15**	78	52.0
**3.16**	49	32.7
**3.17**	36	24.0
**3.18**	95	63.3
**3.19**	56	37.3
**3.20**	63	42.0
**3.21**	76	50.7
**3.22**	36	24.0
**3.23**	72	48.0
**3.24**	54	36.0
**3.25**	47	31.3
**3.26**	73	48.7
**3.27**	63	42.0

References

[1] Y. Liang, R. Kleinmans, C. G. Daniliuc, F. Glorius, J. Am. Chem. Soc. 2022, ***144***, 20207–20213.

[2] Y. Mizutani, H. Tanimoto, T. Morimoto, Y. Nishiyama, K. Kakiuchi, Tetrahedron Lett. 2012, ***53***, 5903–5906.

[3] S. Meena, R. Singh, R. A. Vishwakarma, M. A. Aga, S. K. Jain, Tetrahedron Lett. 2016, ***57***, 3715–3717.

[4] B. E. Howard, K. A. Woerpel, Tetrahedron 2009, ***65***, 6447–6453.

[5] A. Jordan, K. D. Whymark, J. Sydenham, H. F. Sneddon, Green Chem. 2021, ***23***, 6405–6413.

[6] A. B. Shabade, R. K. Singh, R. G. Gonnade, B. Punji, Adv. Synth. Catal. 2024, ***366***, 3338–3345.

[7] H. Maeda, K. Takahashi, H. Ohmori, Tetrahedron 1998, ***54***, 12233–12242.

[8] S. M. Nicolle, C. J. Moody, Chem. Eur. J. 2014, ***20***, 4420–4425.

[9] J. R. Denton, D. Sukumaran, H. M. L. Davies, Org. Lett. 2007, ***9***, 2625–2628.

NMR spectra of previously described compounds

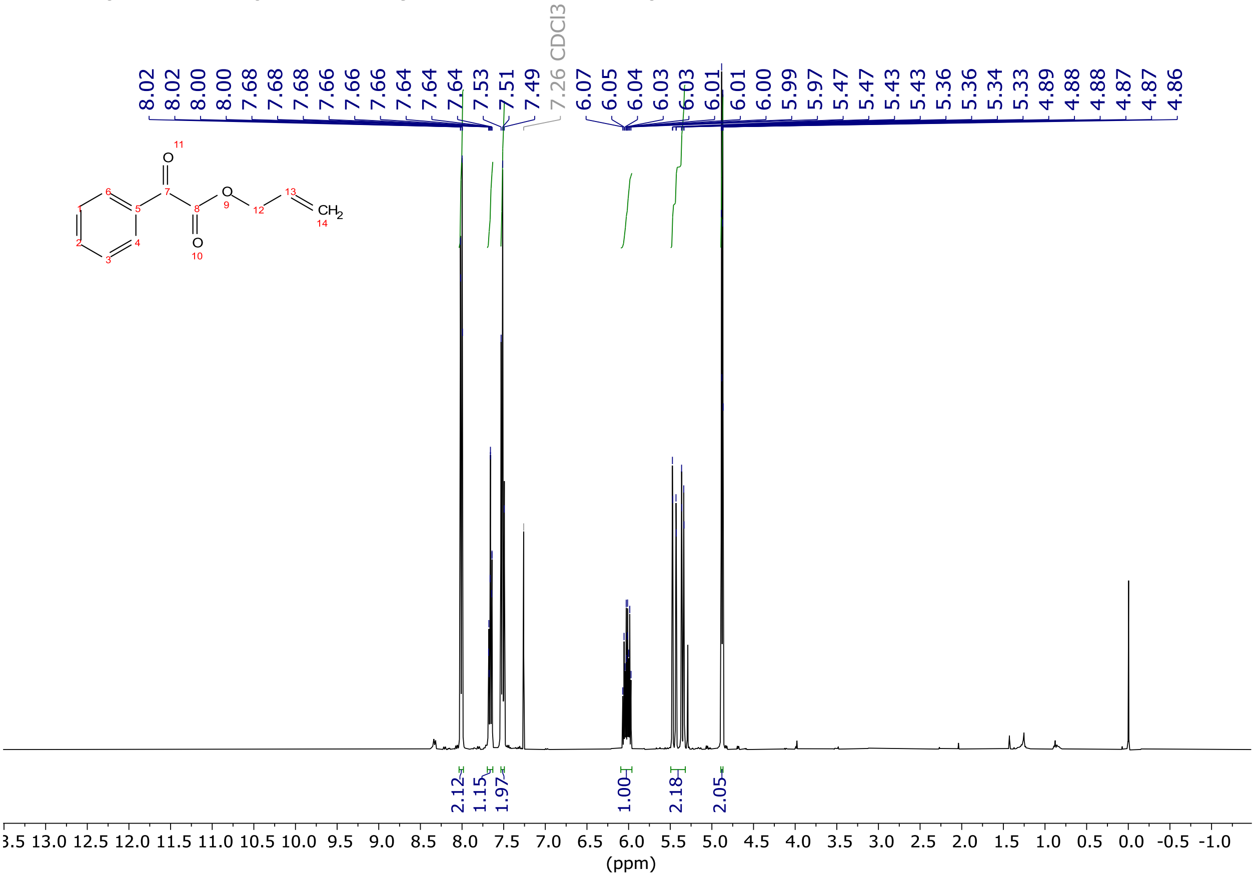

Figure S 4: ^{1}H NMR spectrum of 1.1

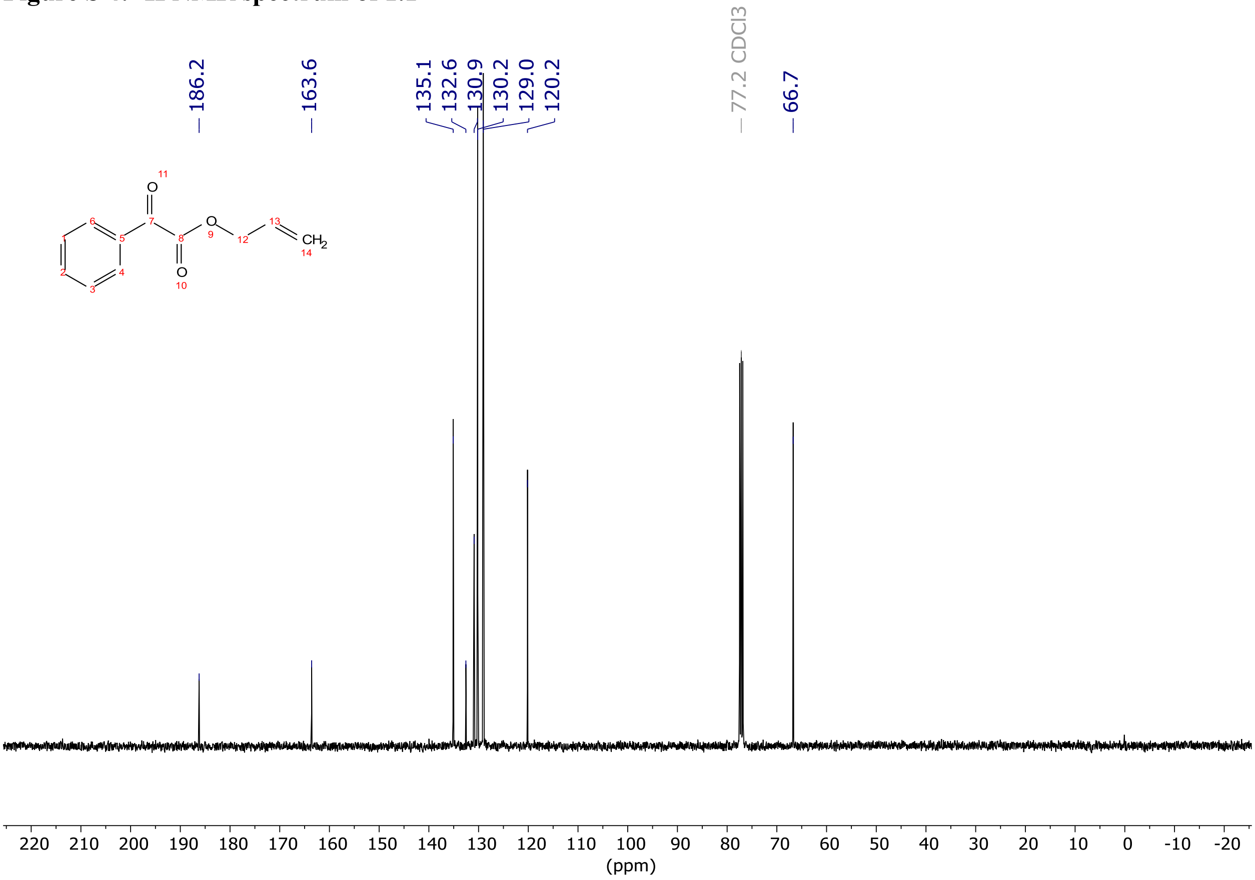

Figure S 5 ^{13}C NMR spectrum of 1.1

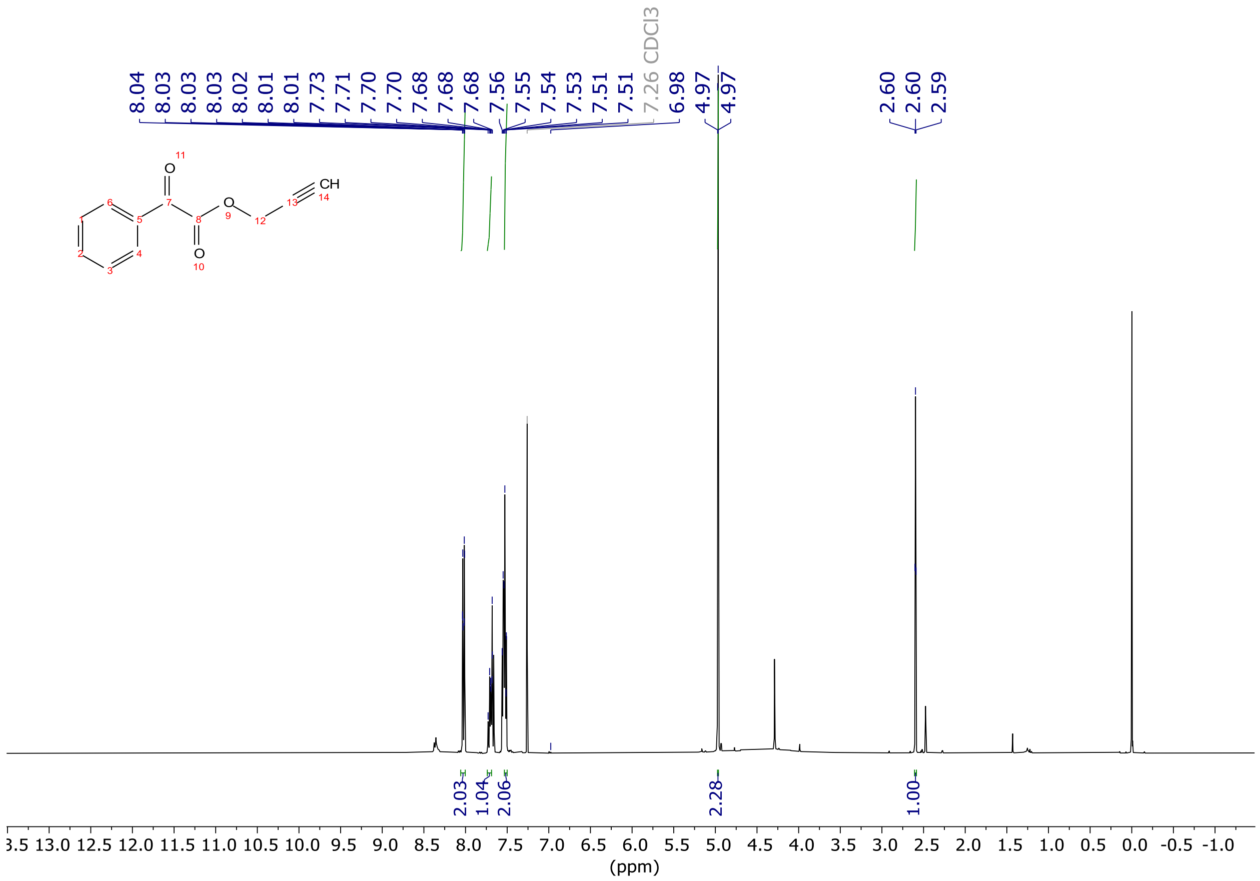

Figure S 6: ^{1}H NMR spectrum of 1.2

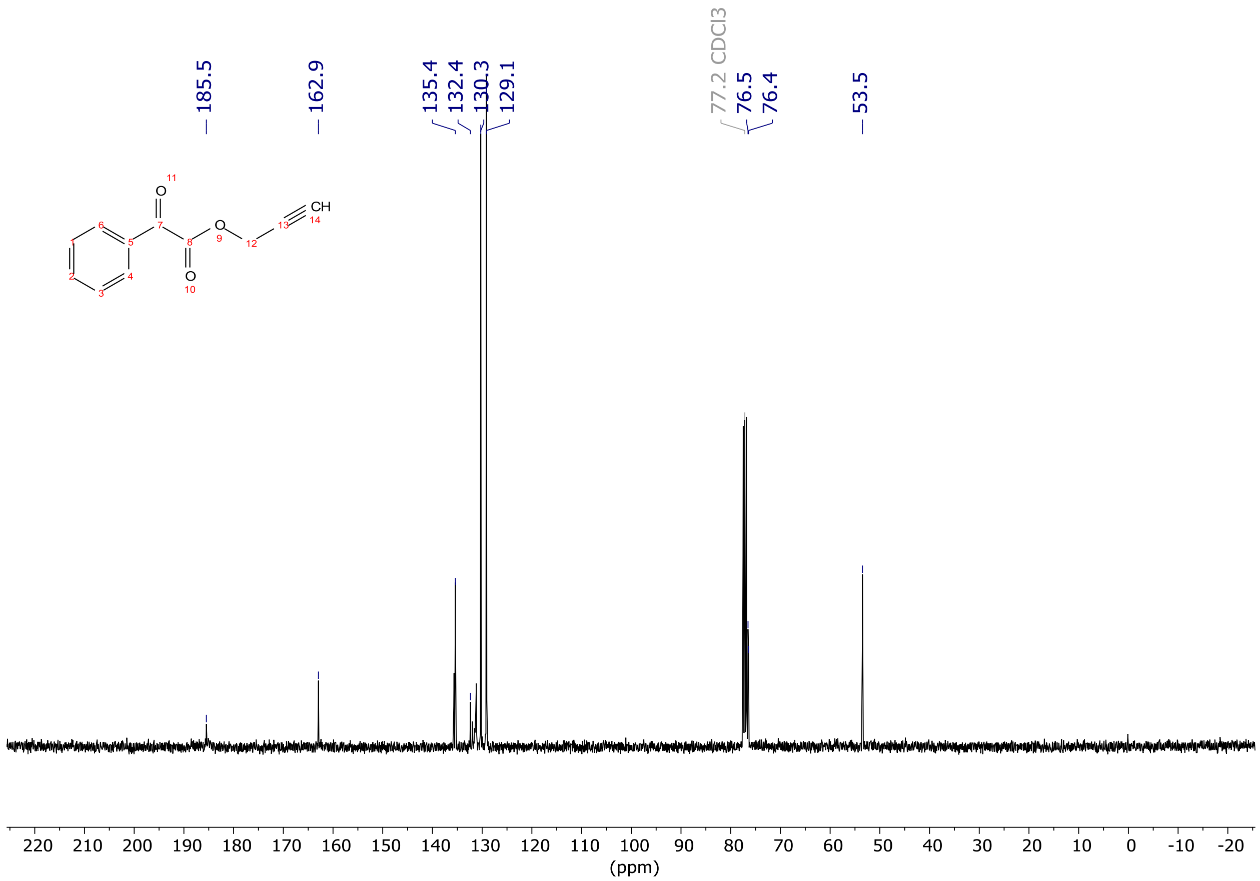

Figure S 7 ^{13}C NMR spectrum of 1.2

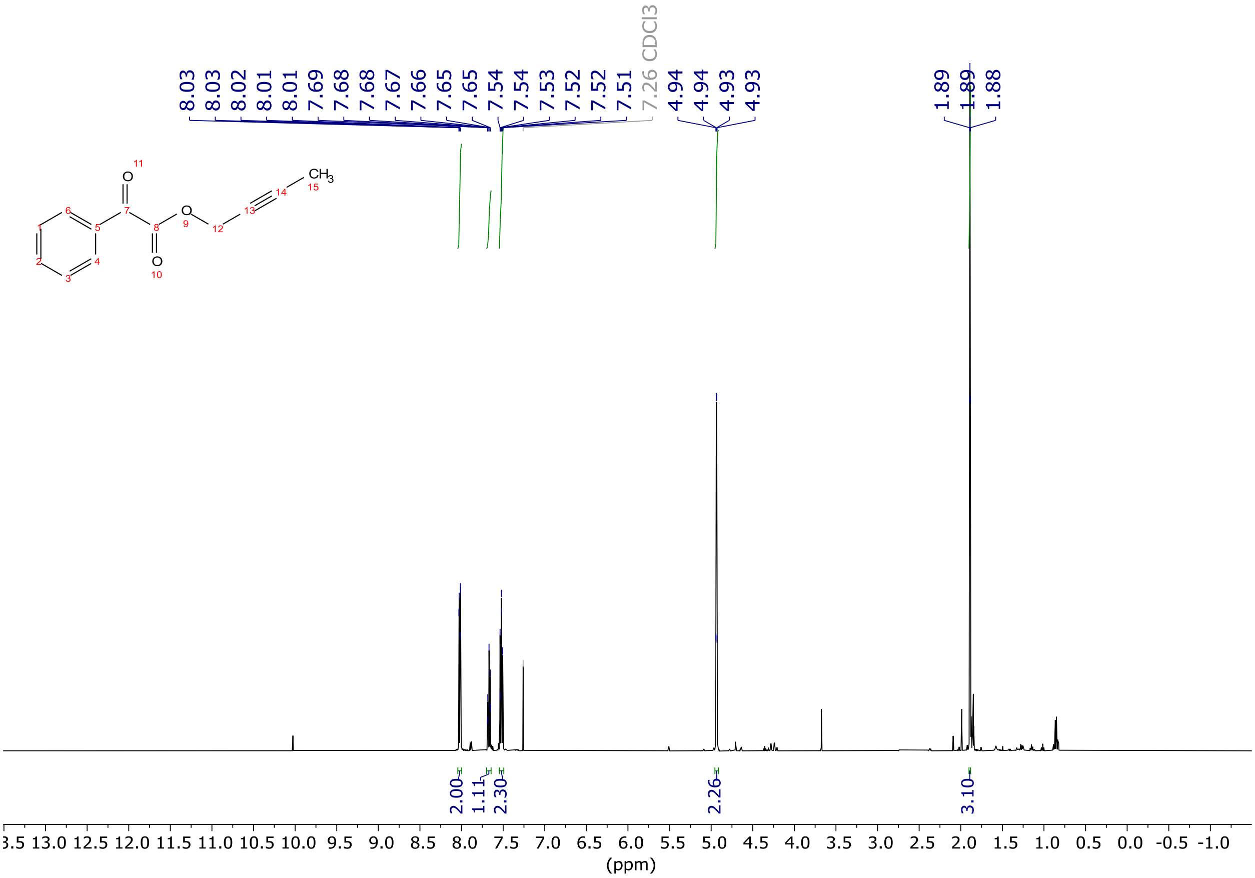

Figure S 8: ^{1}H NMR spectrum of 1.3

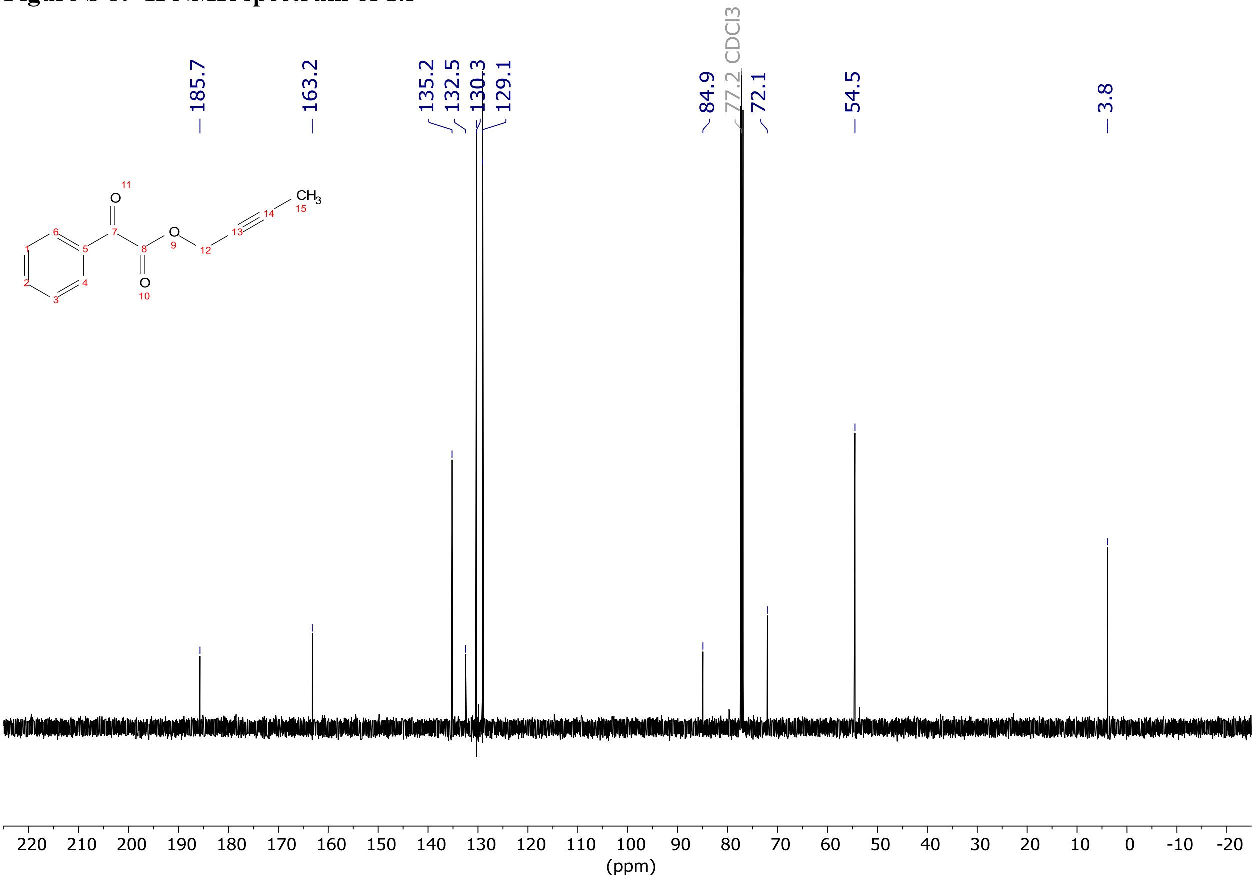

Figure S 9 ^{13}C NMR spectrum of 1.3

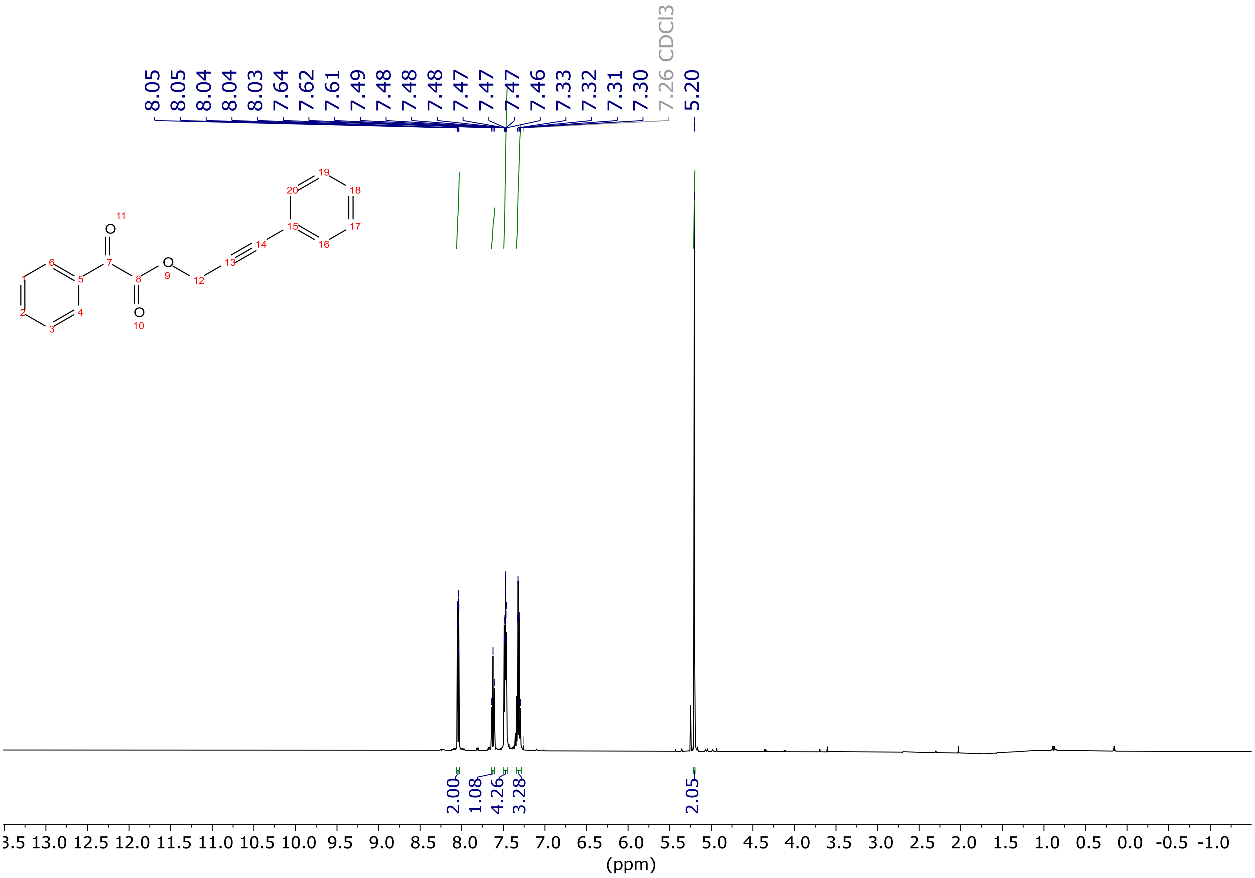

Figure S 10 ^{1}H NMR spectrum of 1.4

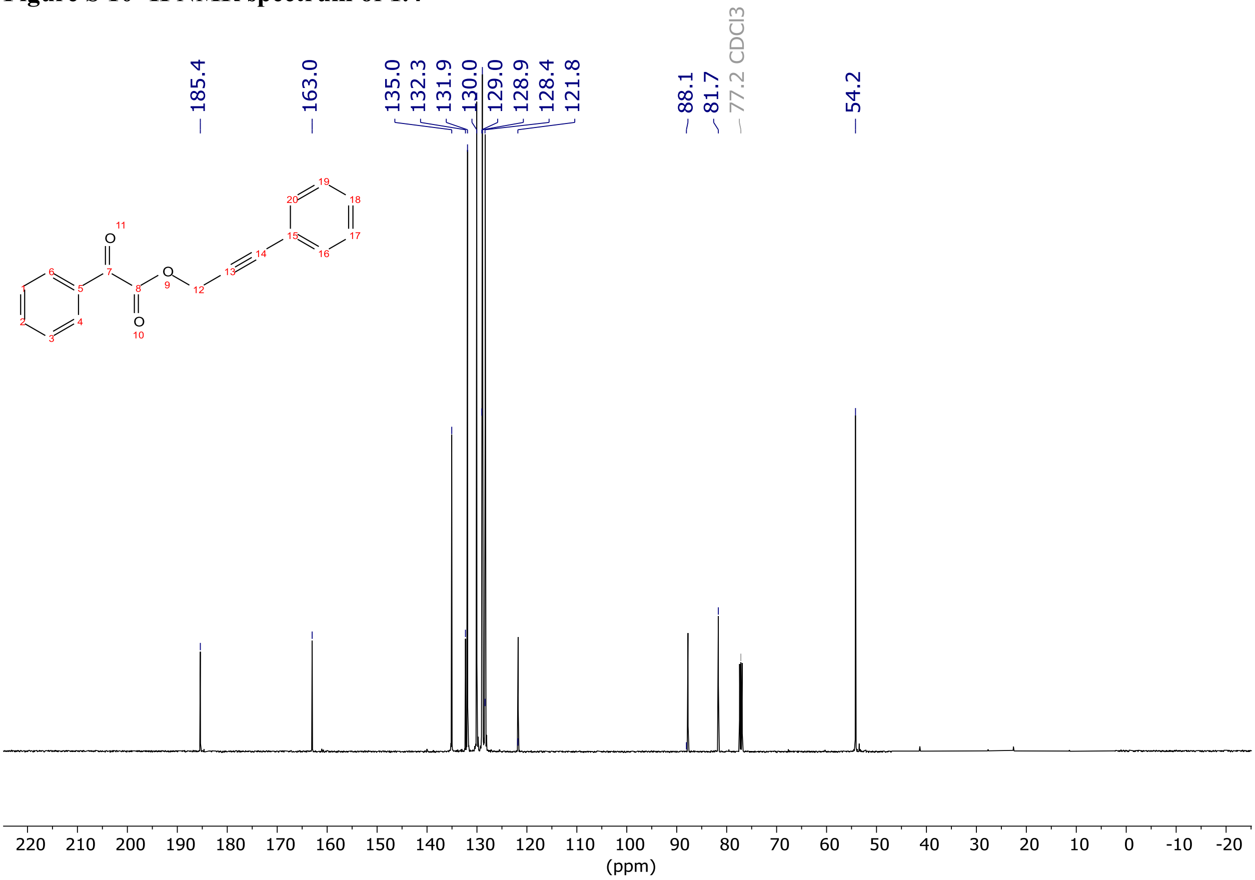

Figure S 11 ^{13}C NMR spectrum of 1.4

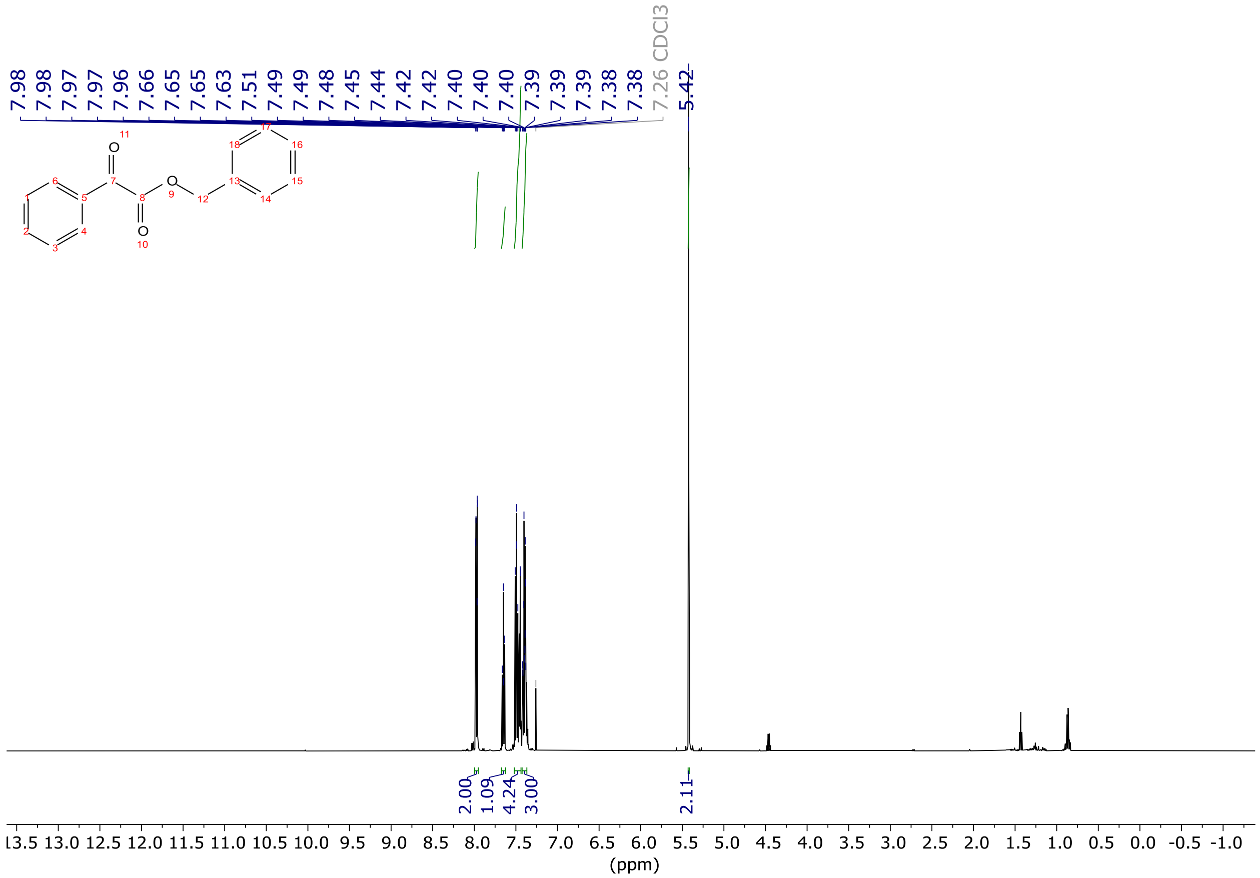

Figure S 12 ^{1}H NMR spectrum of 1.5

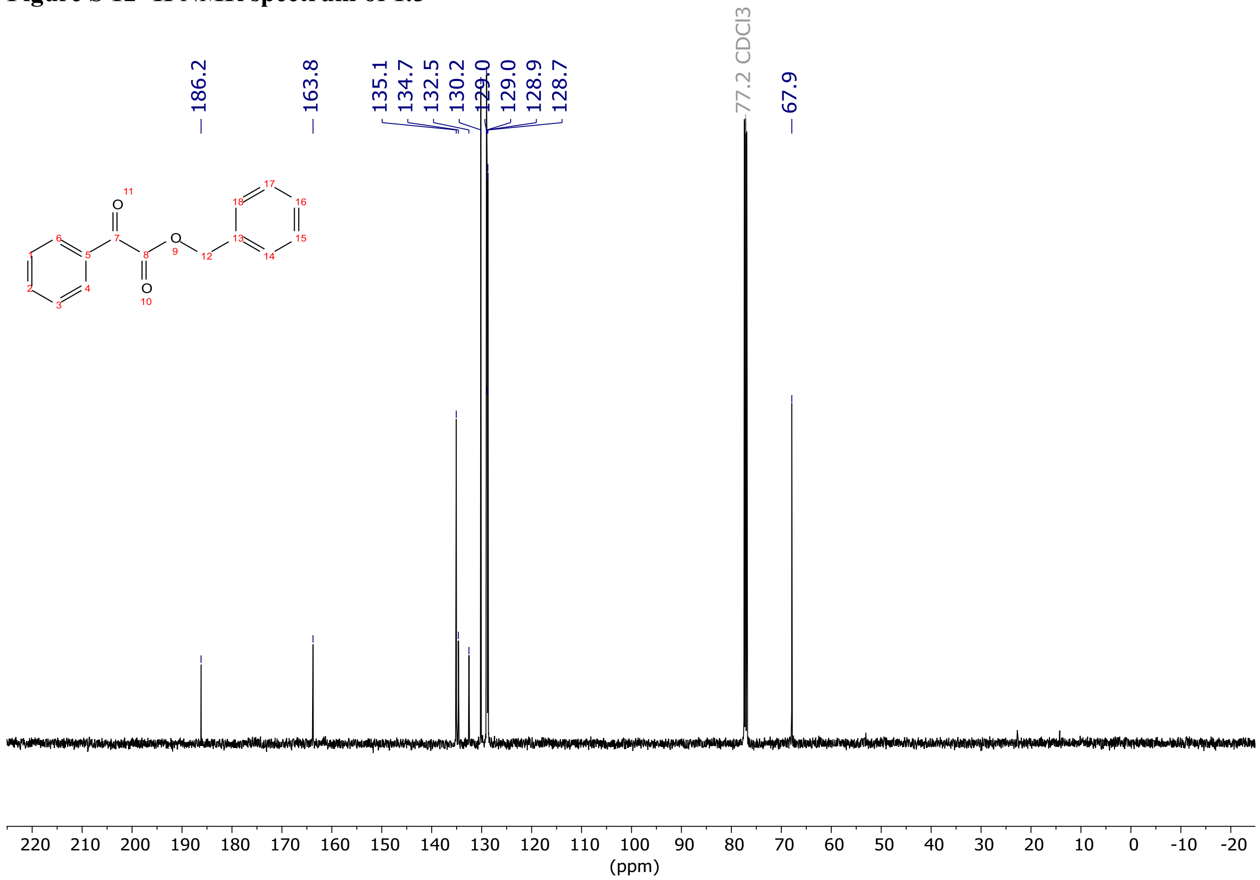

Figure S 13 ^{13}C NMR spectrum of 1.5

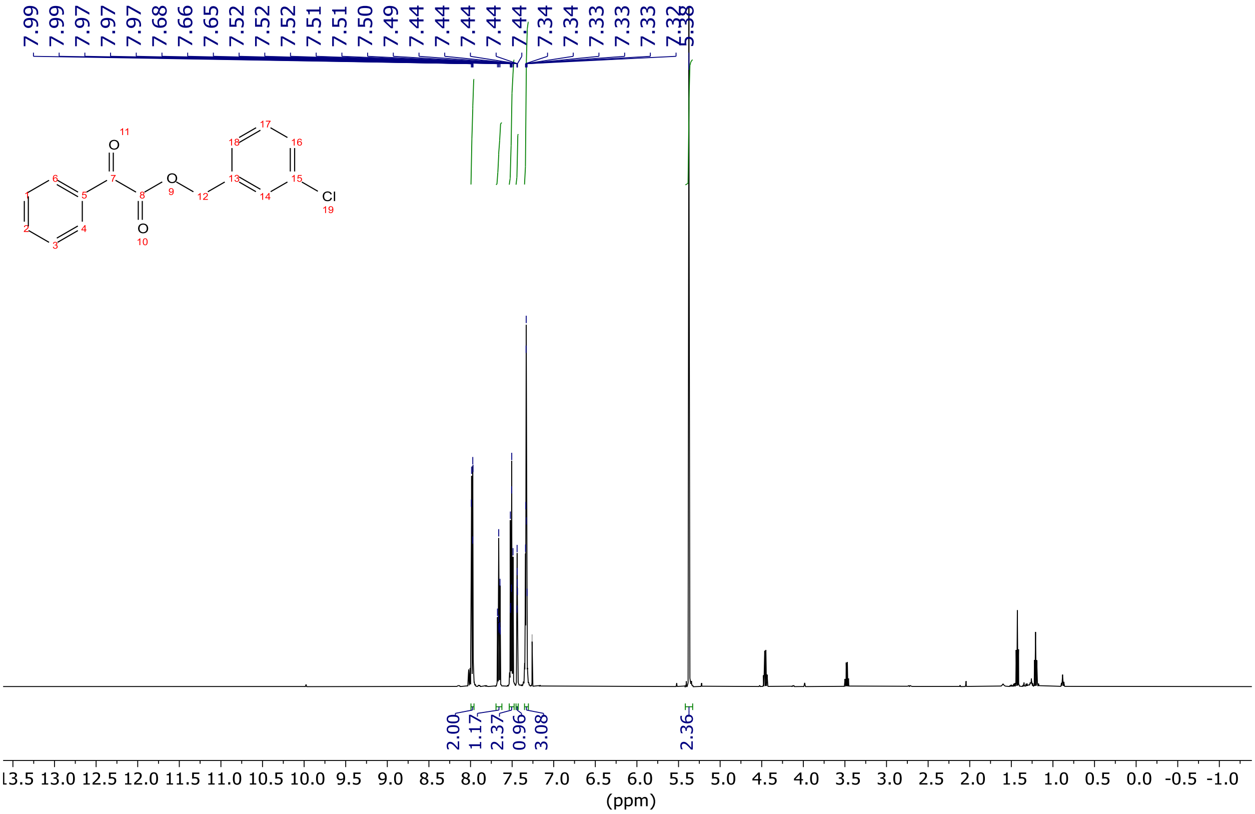

Figure S 14 ^{1}H NMR spectrum of 1.6

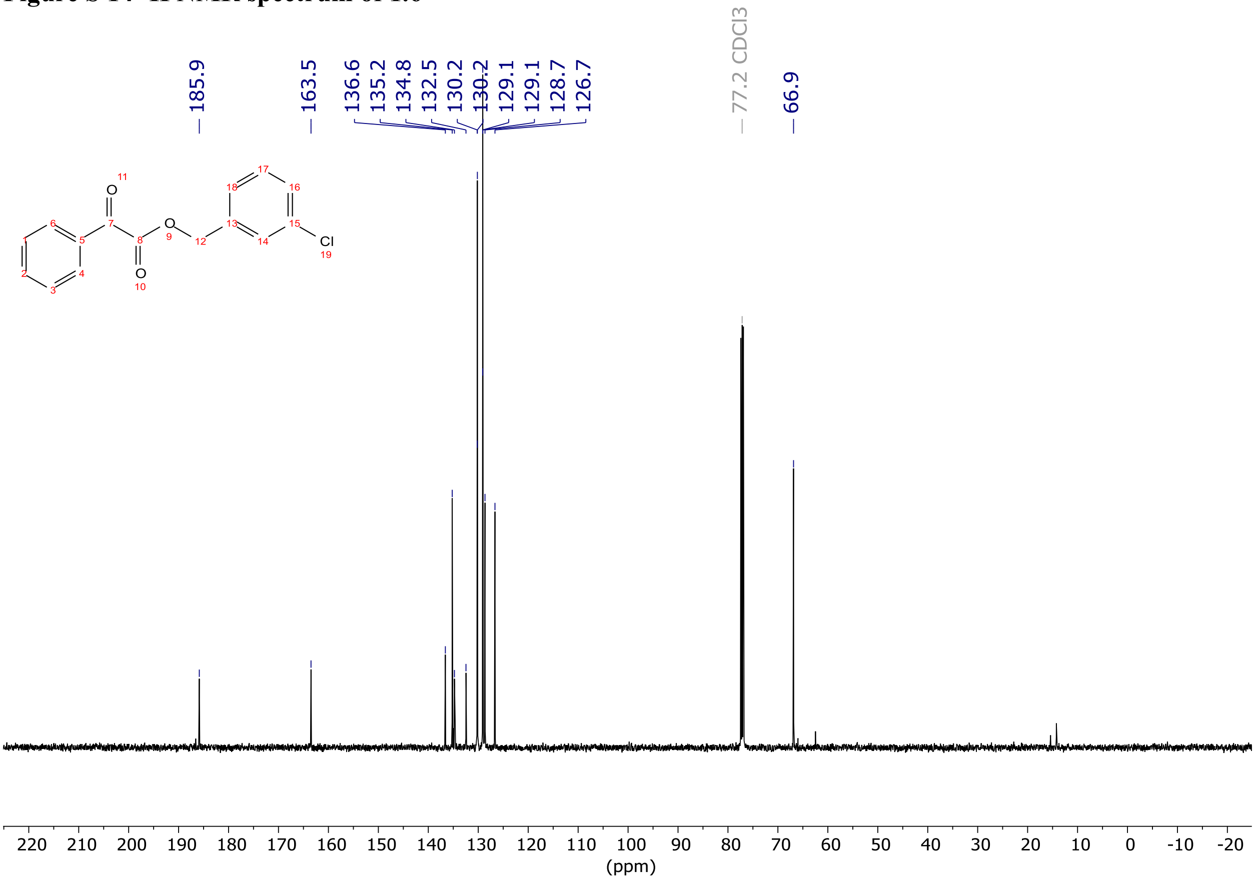

Figure S 15 ^{13}C NMR spectrum of 1.6

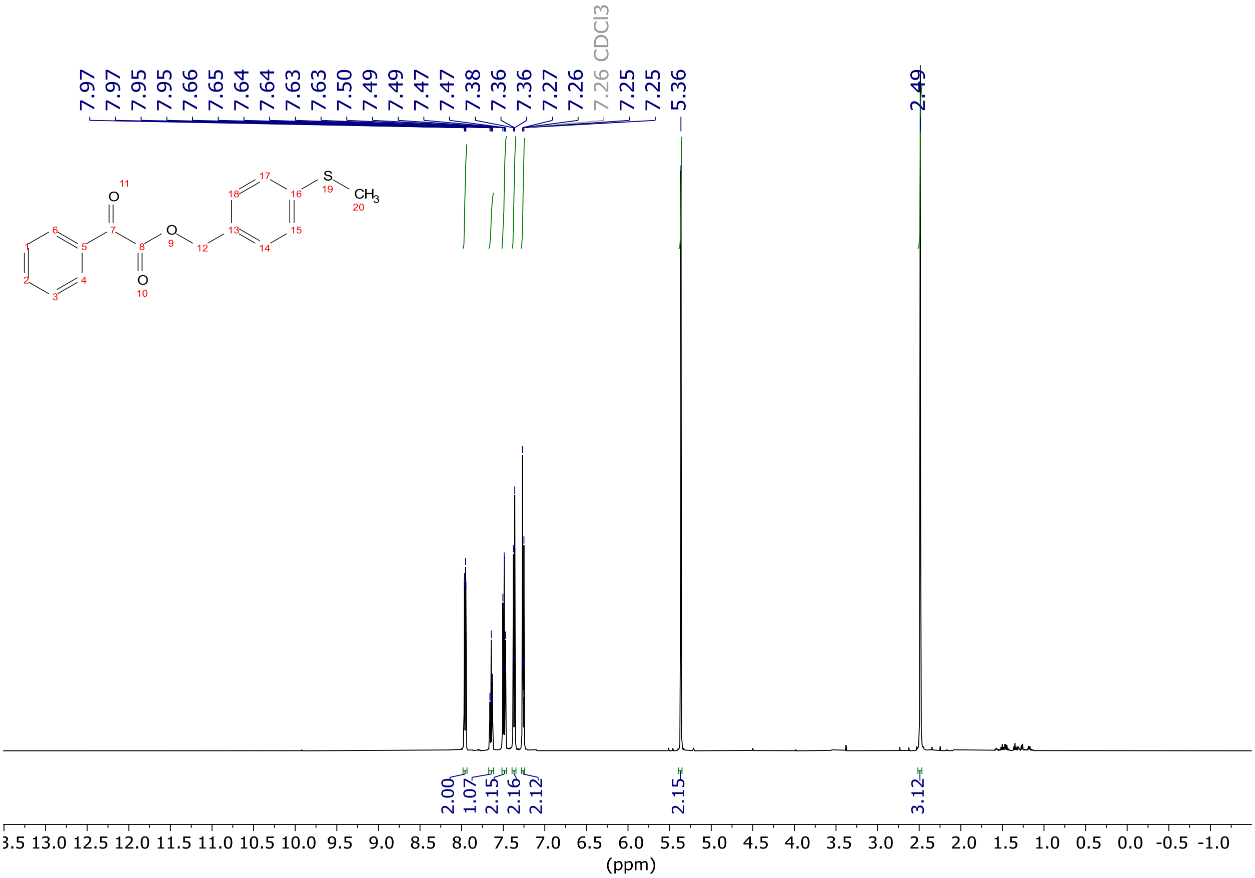

Figure S 16 ^{1}H NMR spectrum of 1.7

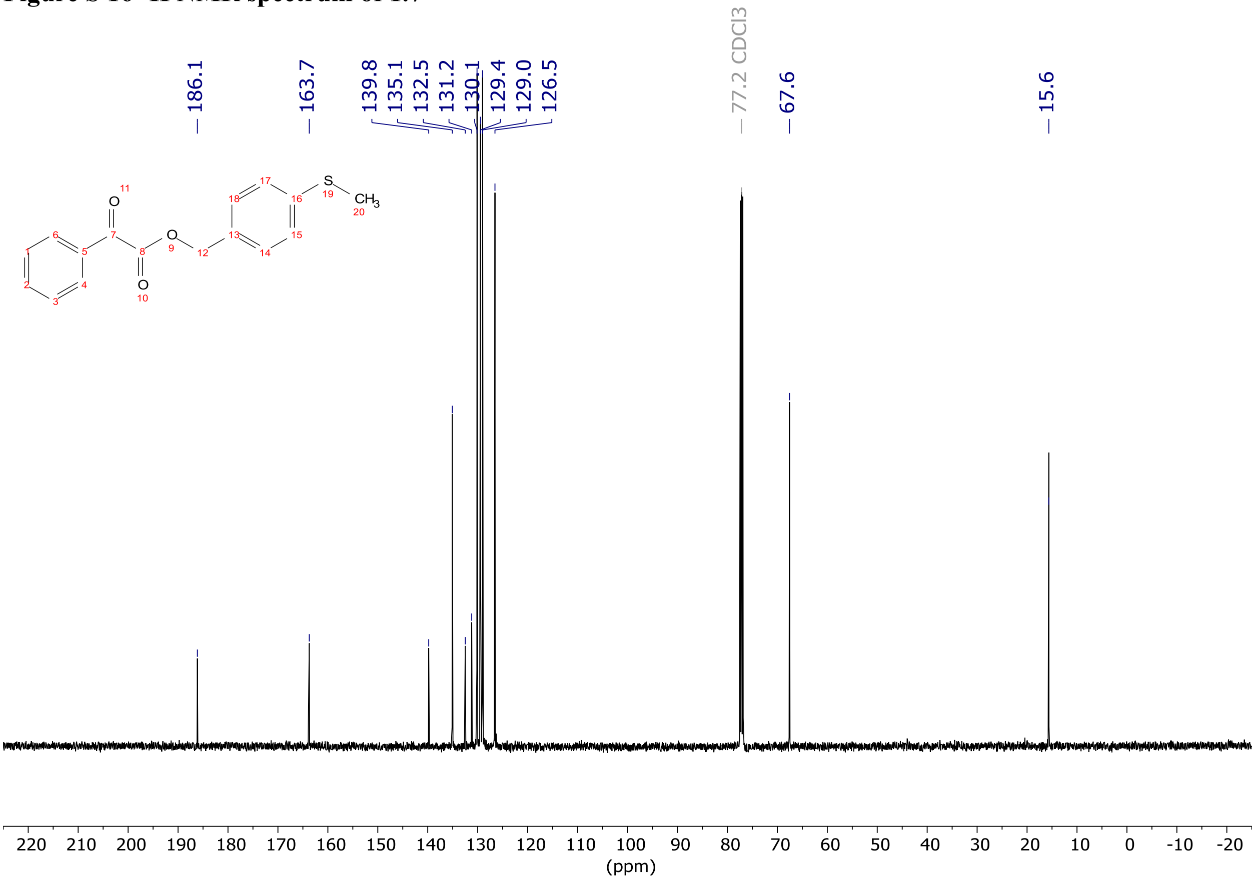

Figure S 17 ^{13}C NMR spectrum of 1.7

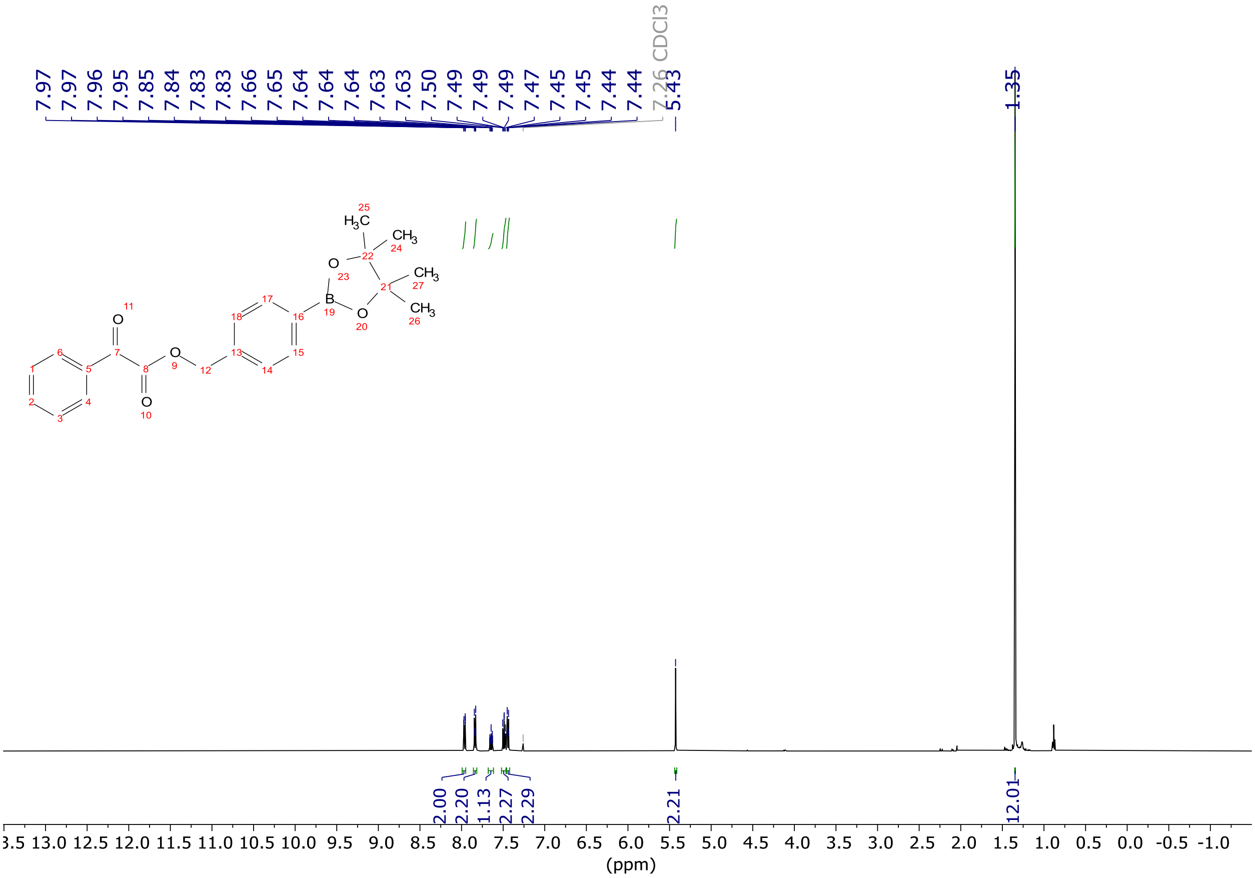

Figure S 18 ^{1}H NMR spectrum of 1.8

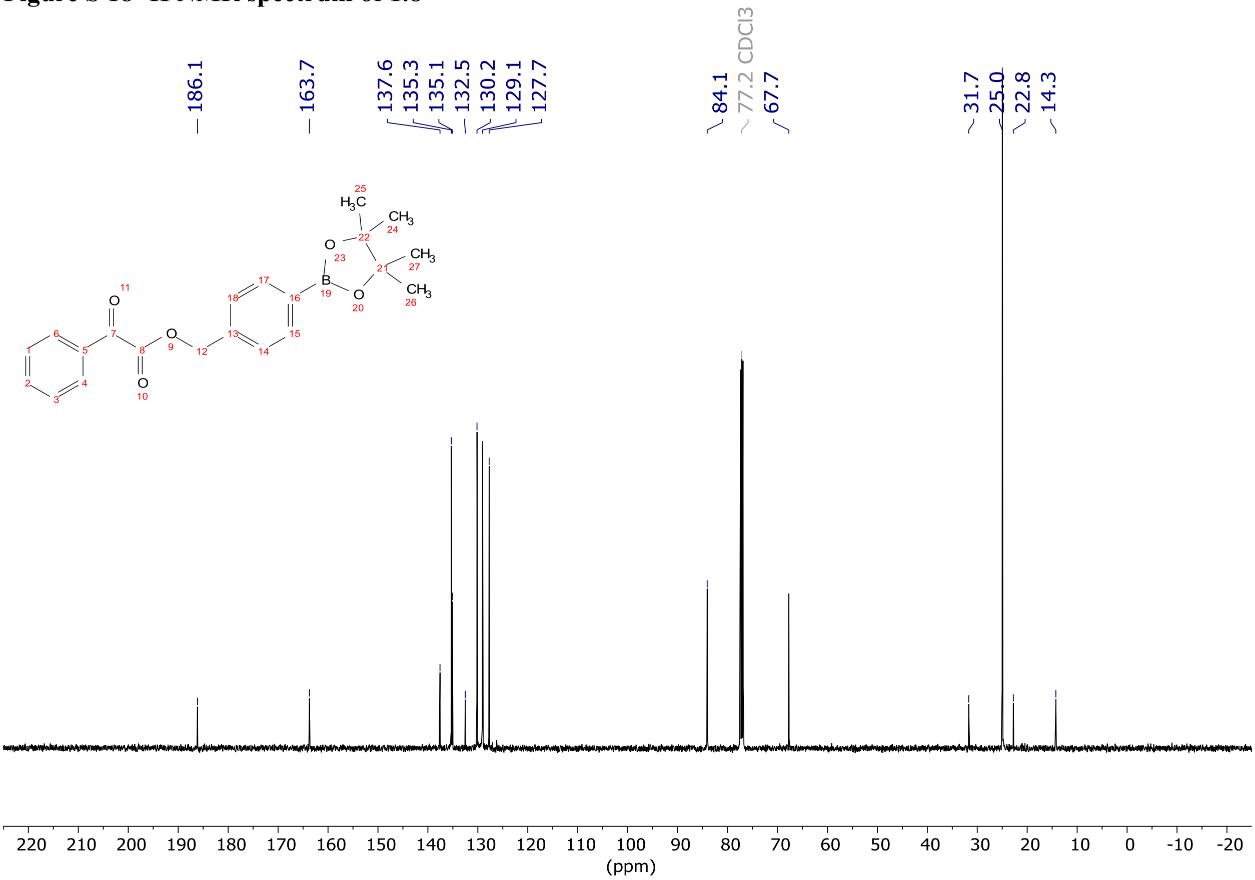

Figure S 19 ^{13}C NMR spectrum of 1.8

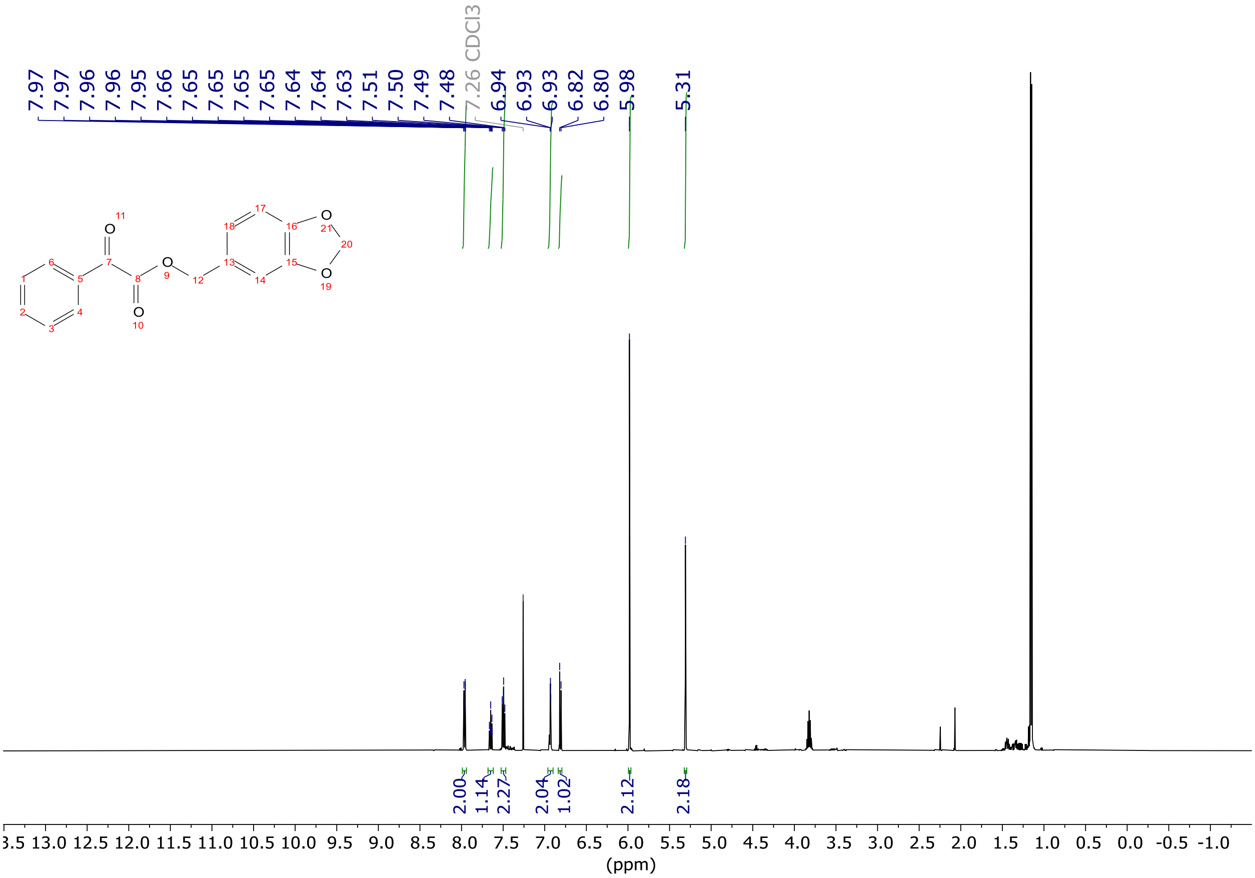

Figure S 20 ^{1}H NMR spectrum of 1.9

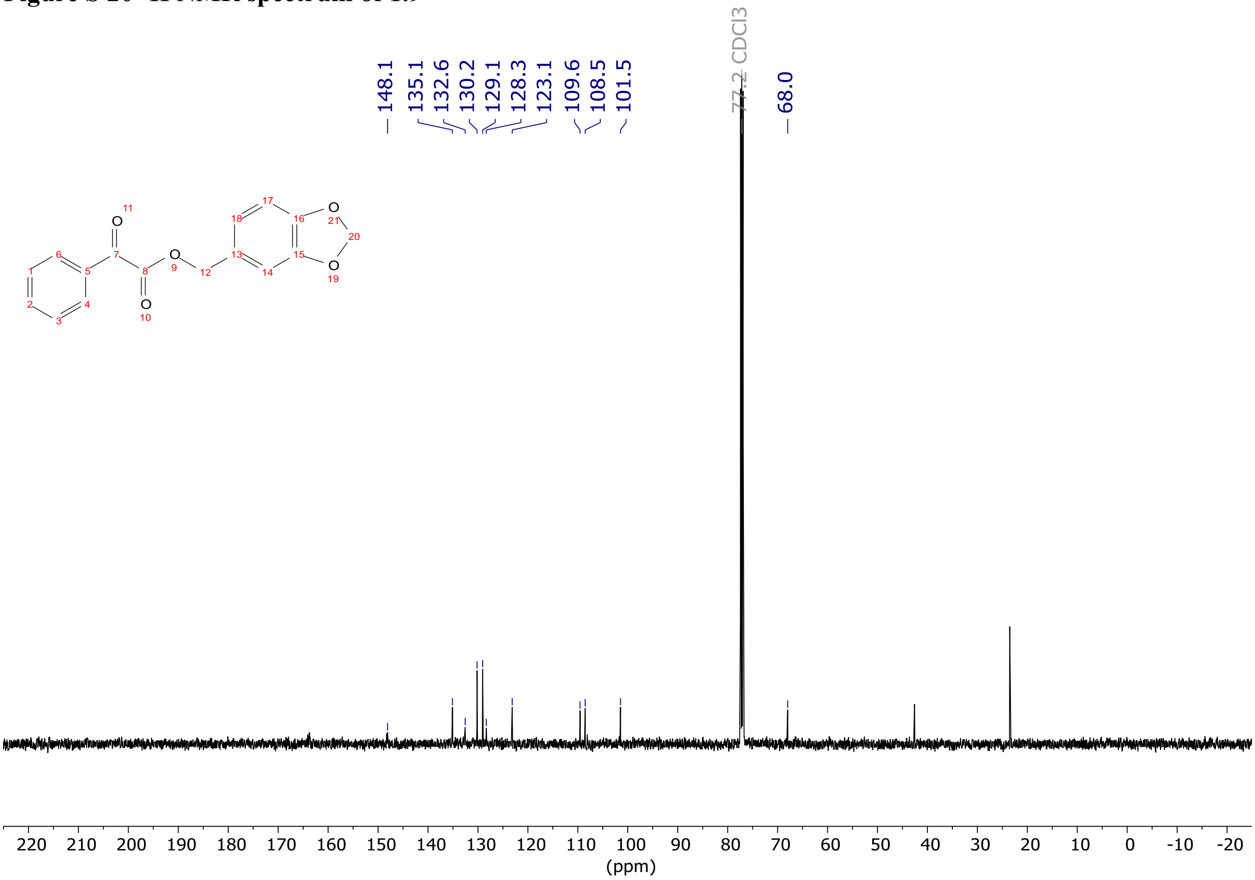

Figure S 21 ^{13}C NMR spectrum of 1.9

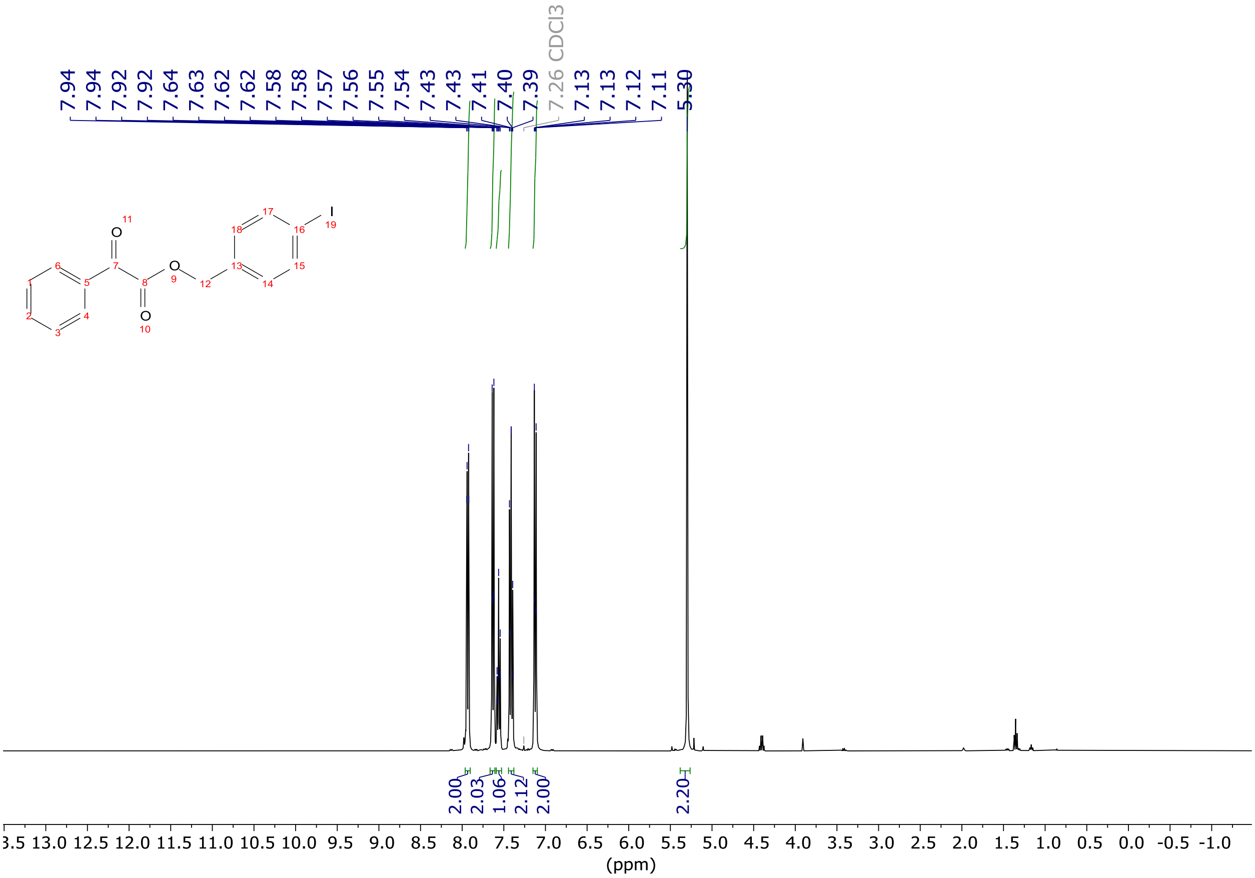

Figure S 22 ^{1}H NMR spectrum of 1.10

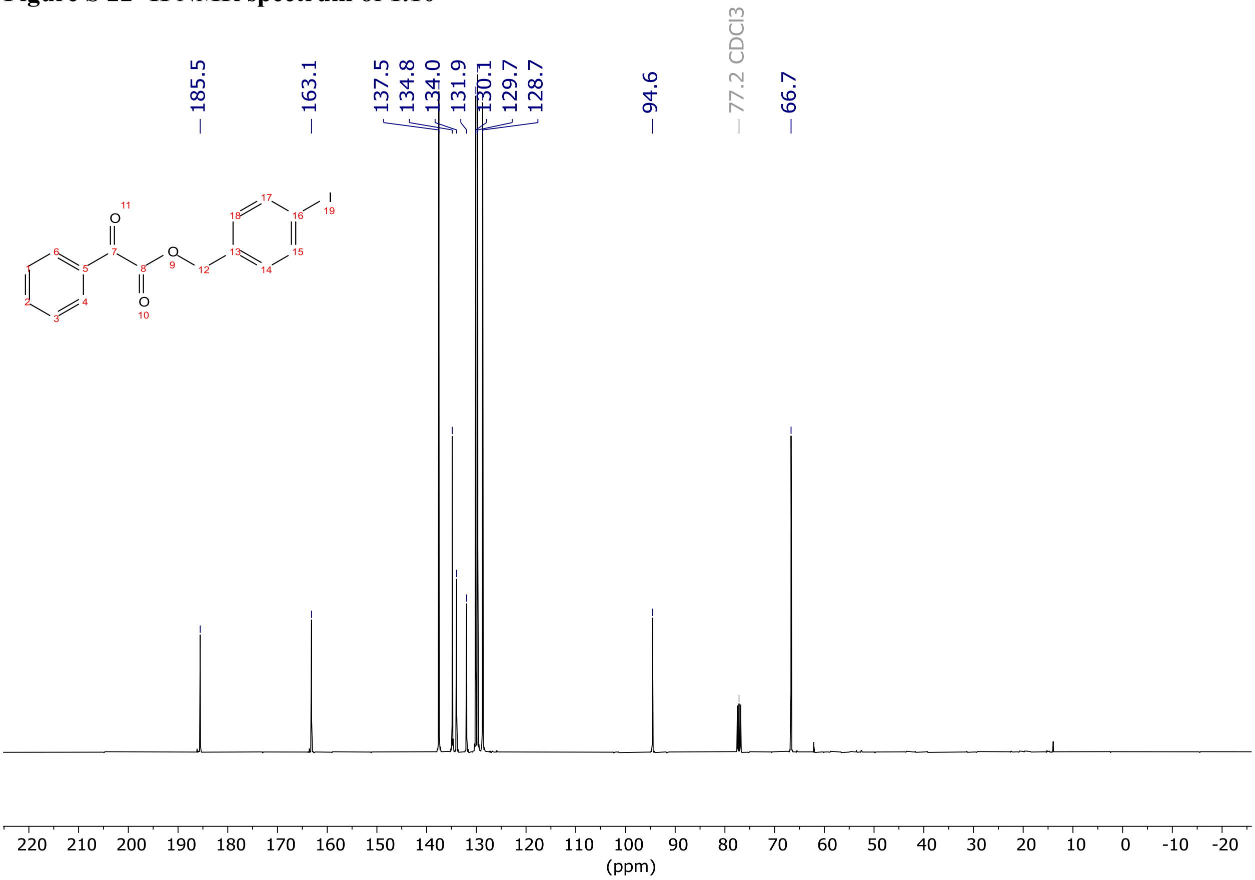

Figure S 23 ^{13}C NMR spectrum of 1.10

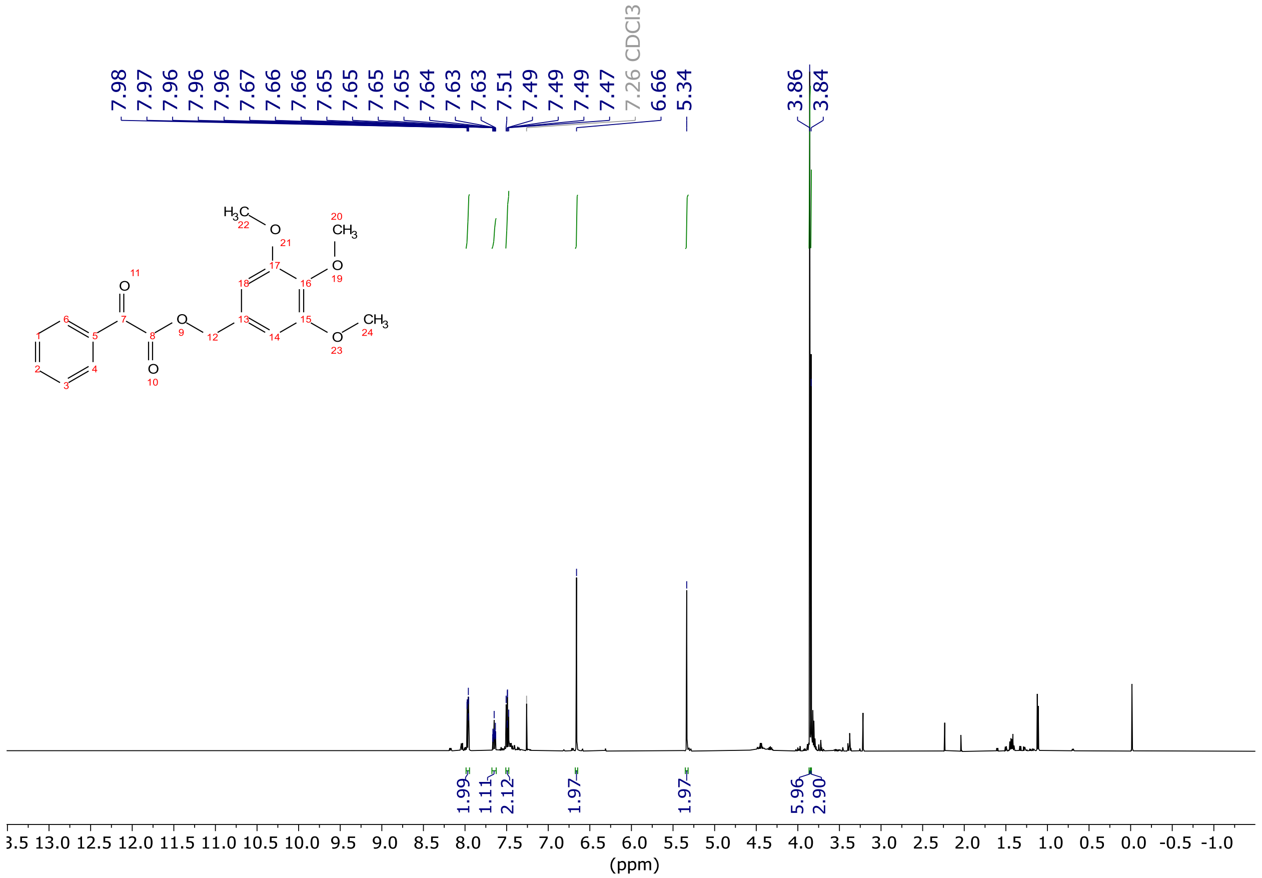

Figure S 24 ^{1}H NMR spectrum of 1.11

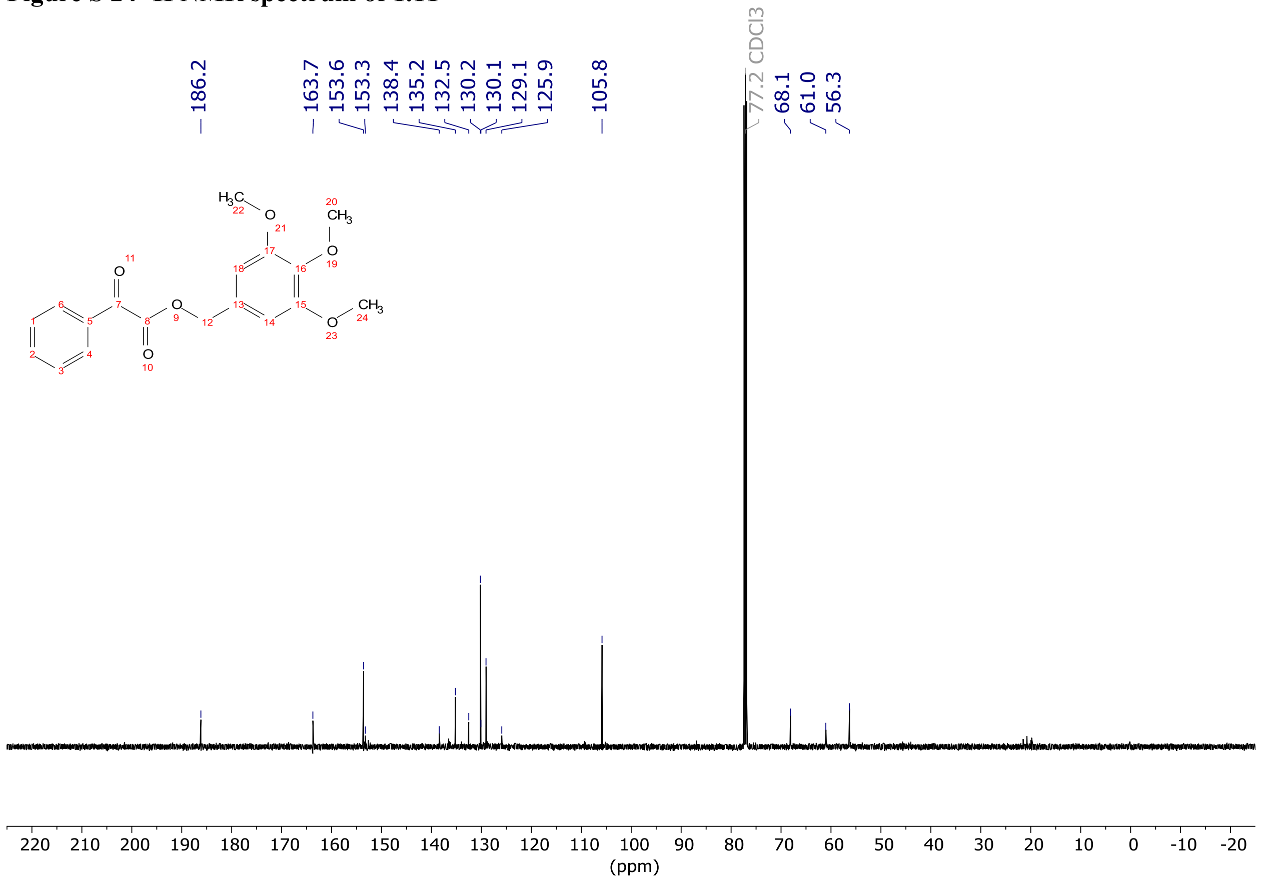

Figure S 25 ^{13}C NMR spectrum of 1.11

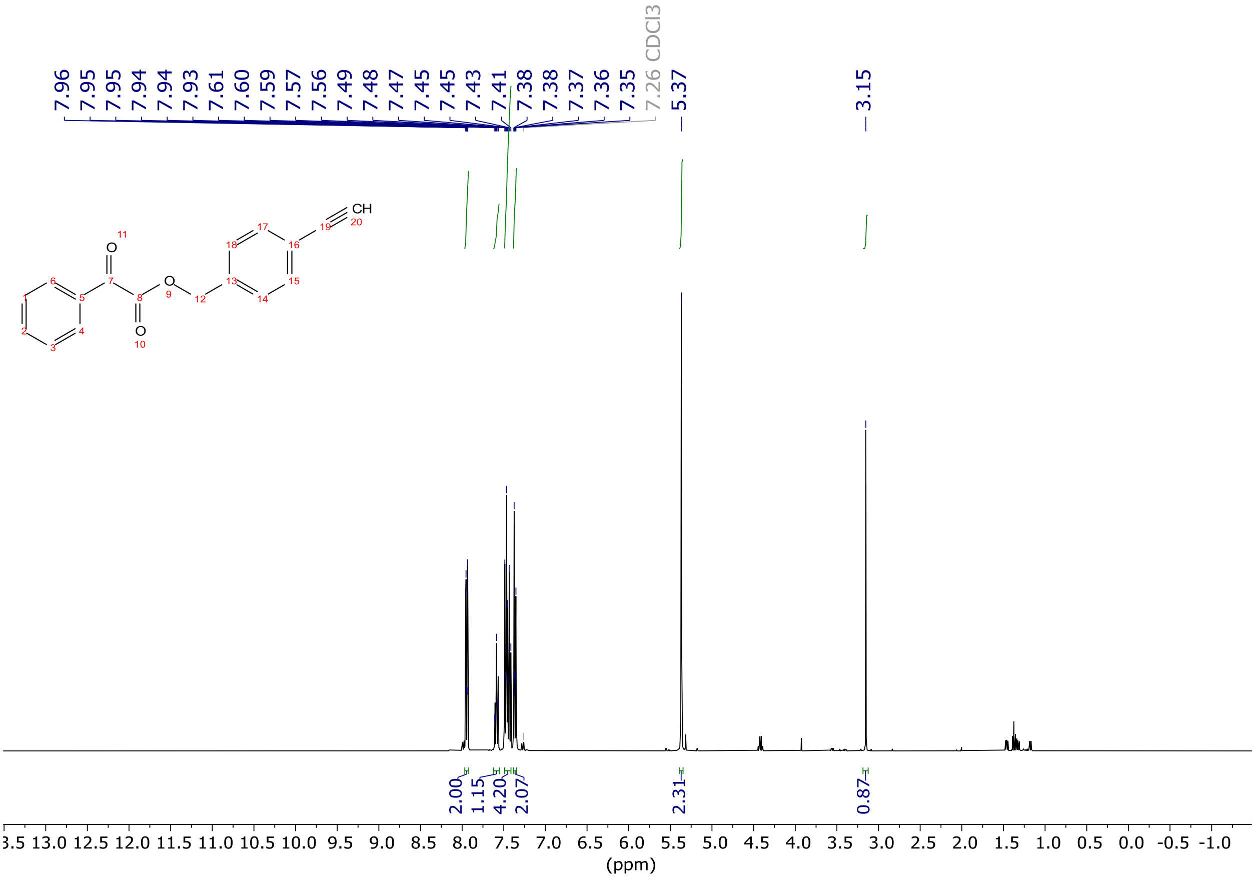

Figure S 26 ^{1}H NMR spectrum of 1.12

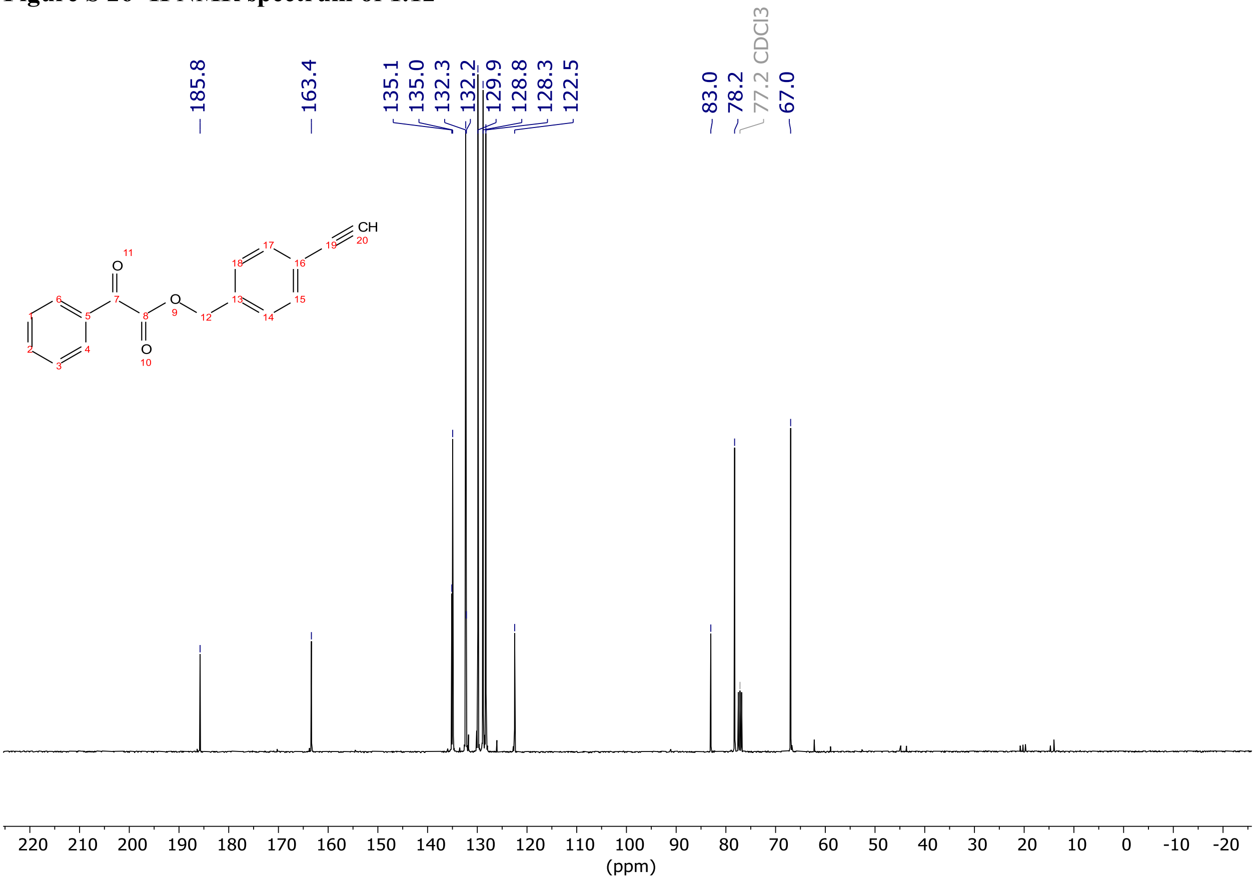

Figure S 27 ^{13}C NMR spectrum of 1.12

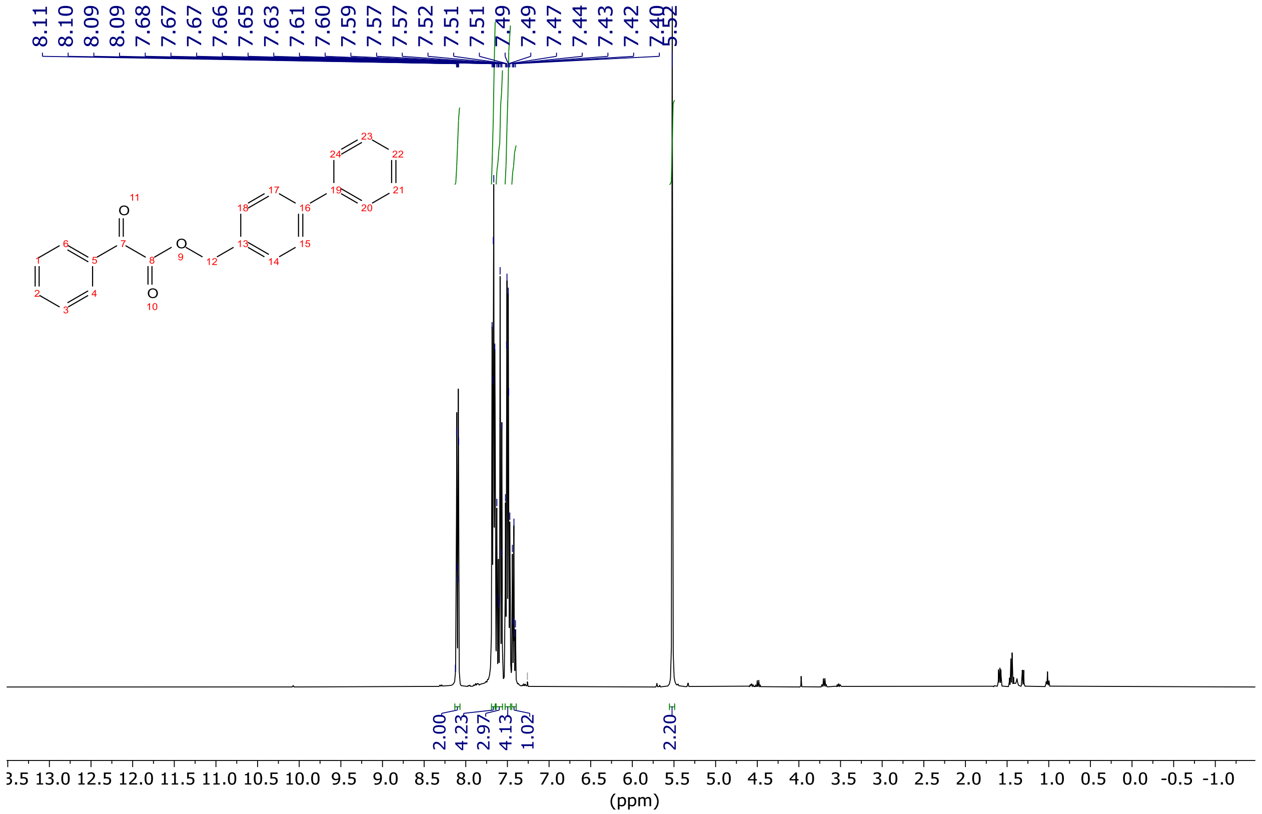

Figure S 28 ^{1}H NMR spectrum of 1.13

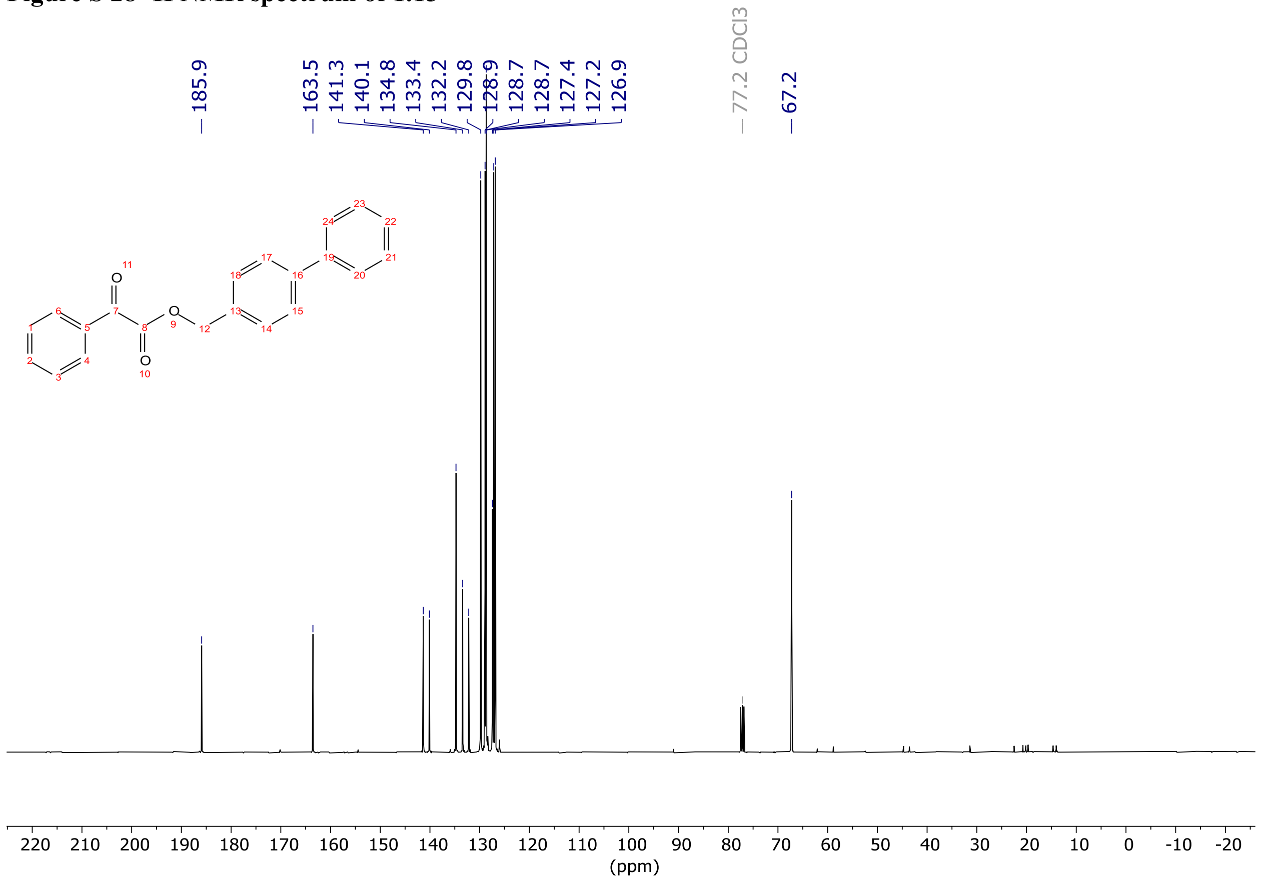

Figure S 29 ^{13}C NMR spectrum of 1.13

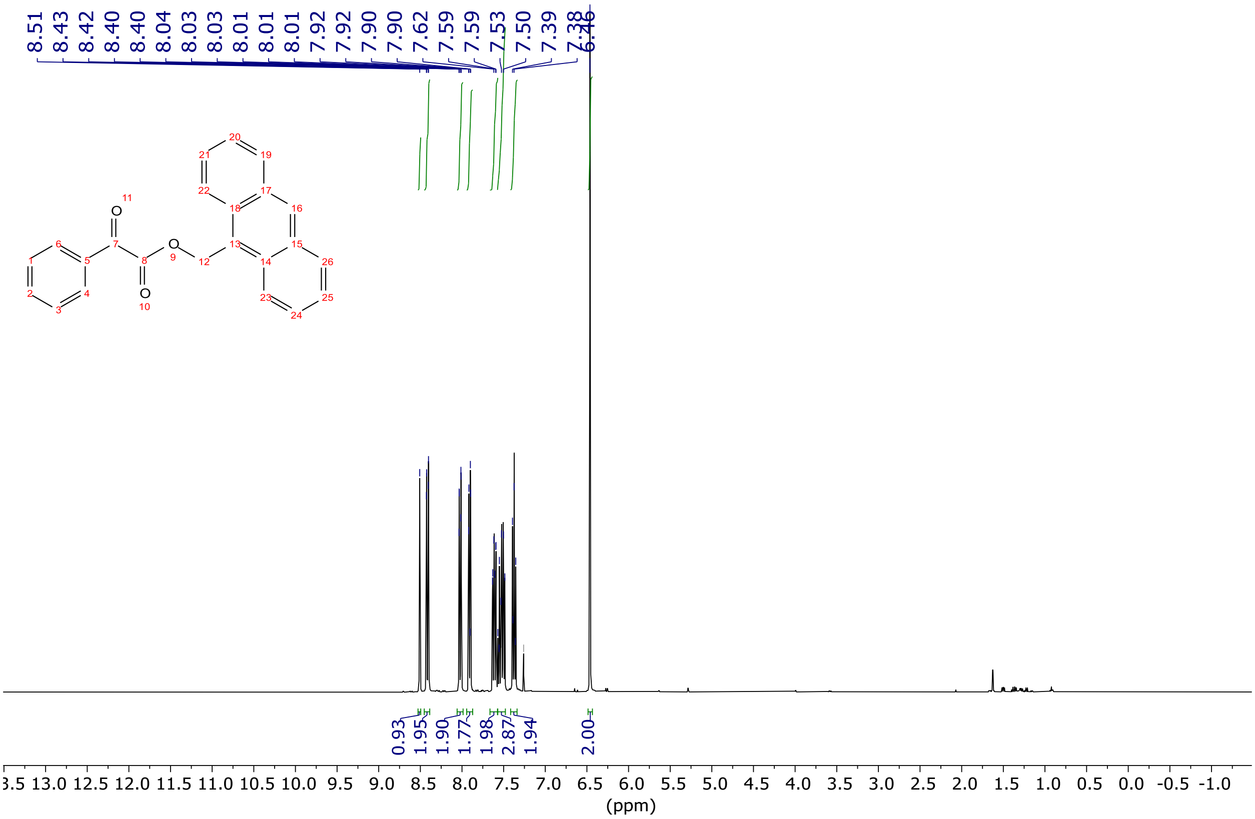

Figure S 30 ^{1}H NMR spectrum of 1.14

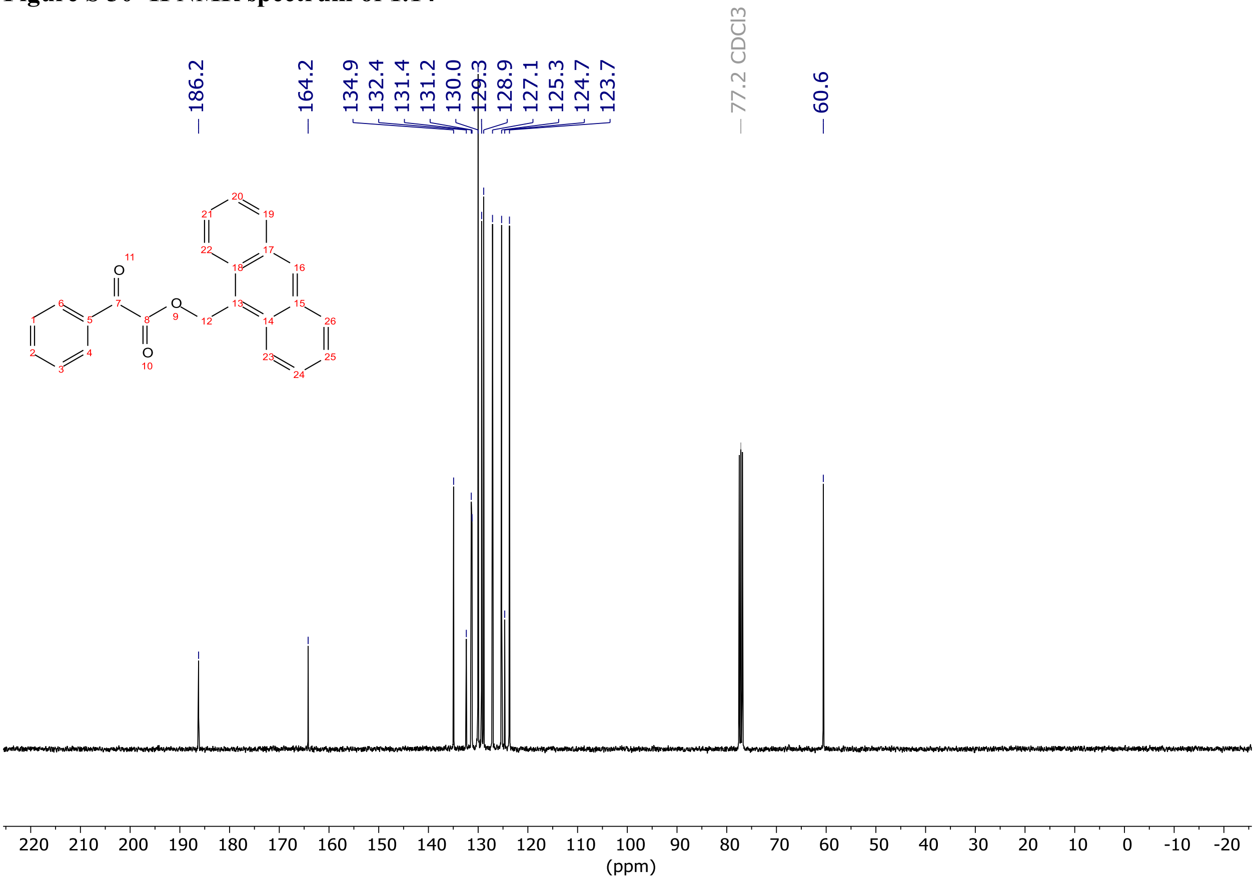

Figure S 31 ^{13}C NMR spectrum of 1.14

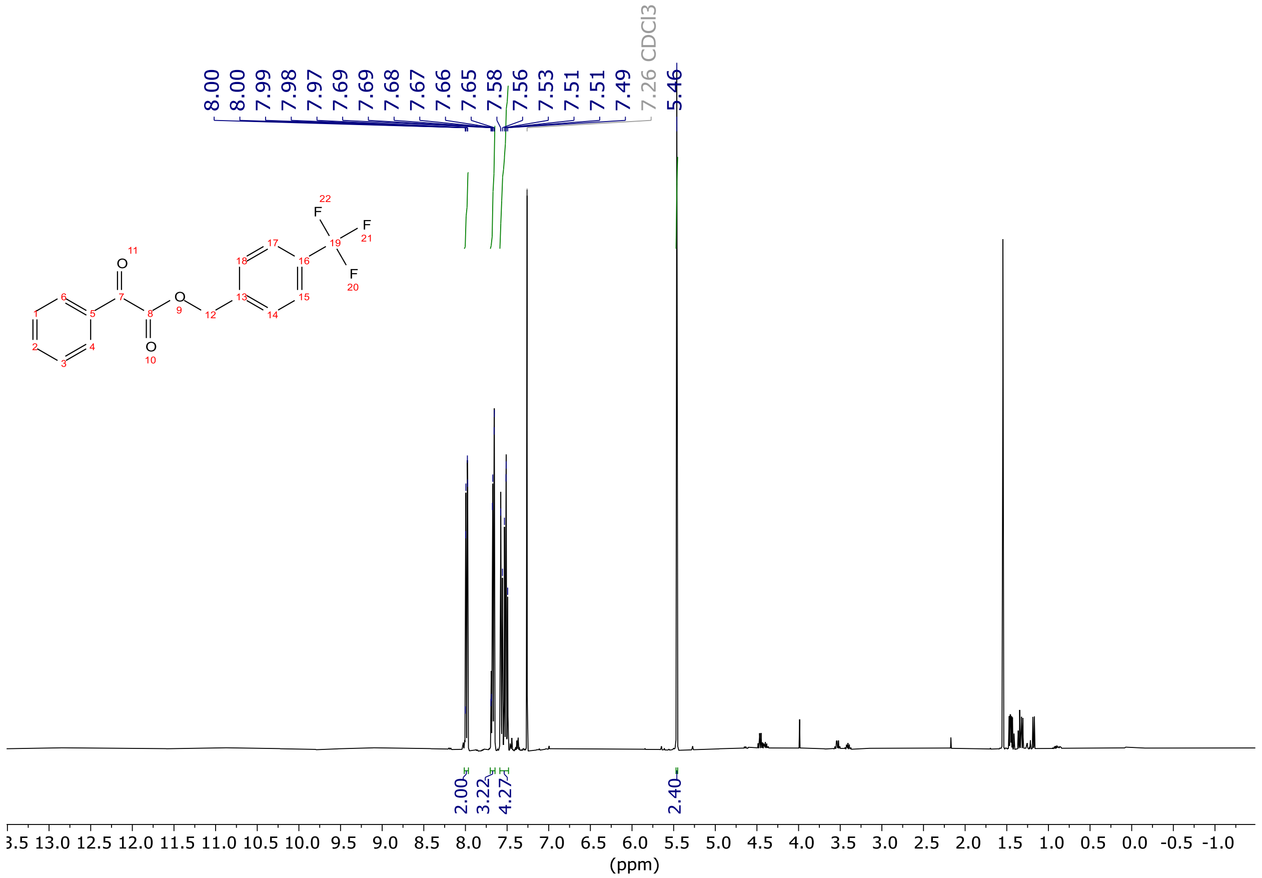

Figure S 32 ^{1}H NMR spectrum of 1.15

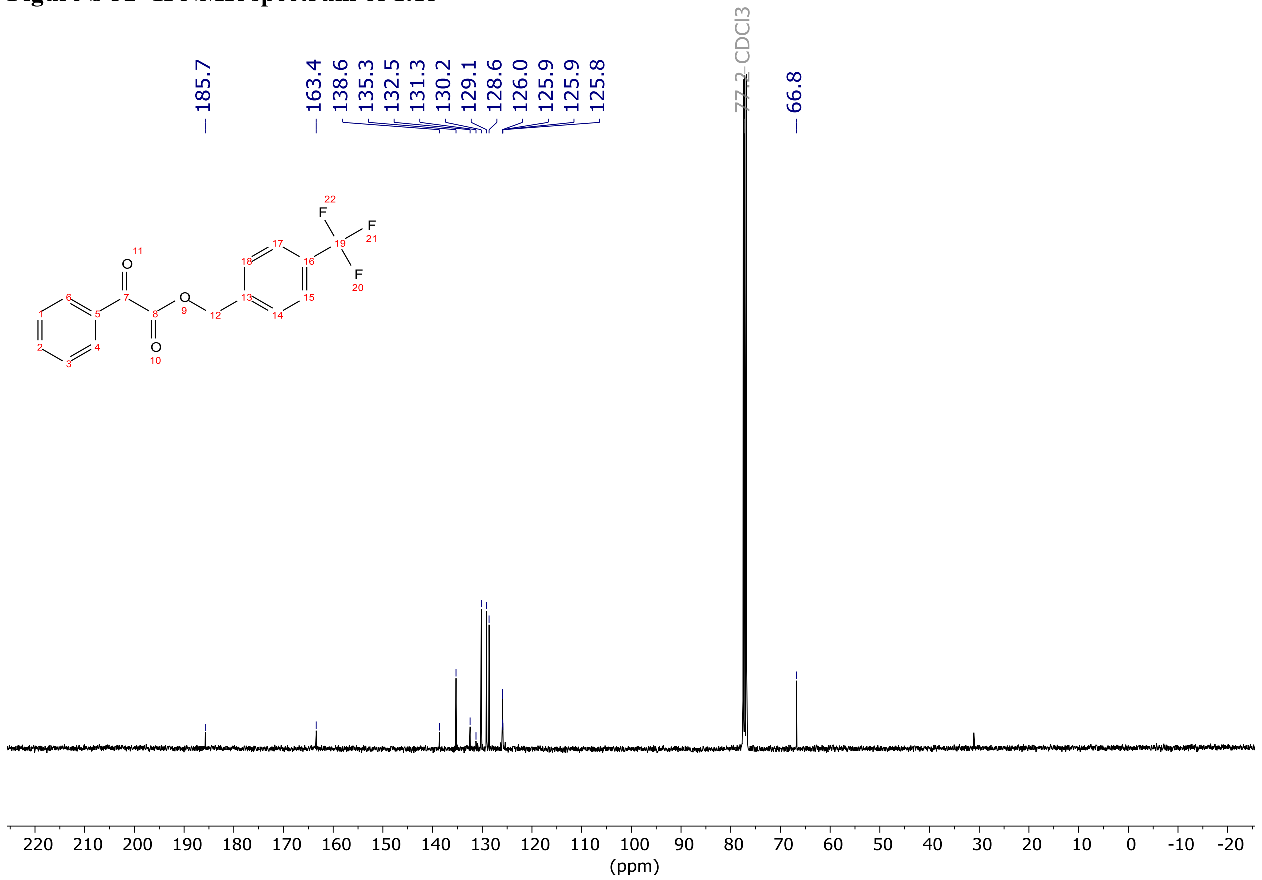

Figure S 33 ^{13}C NMR spectrum of 1.15

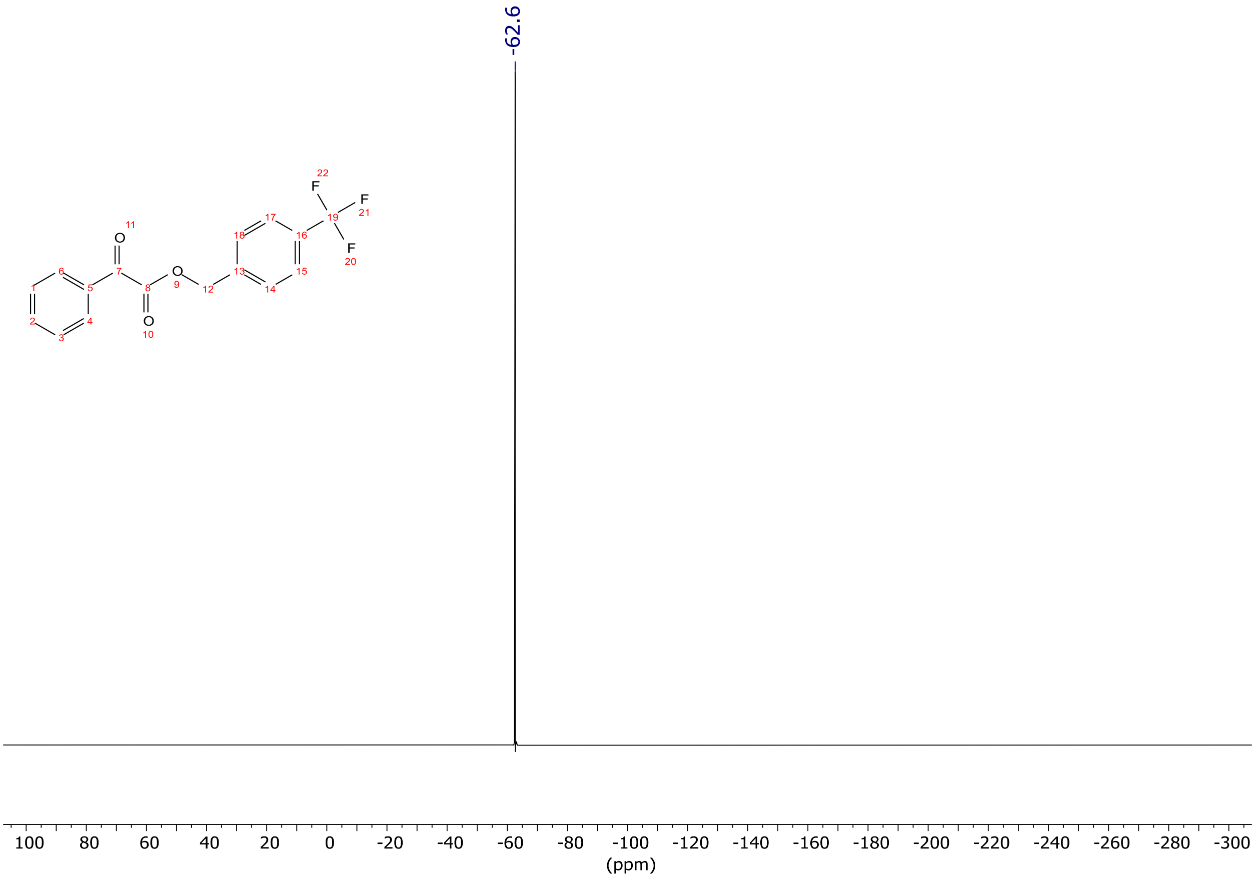

Figure S 34 ^{19}F NMR spectrum of 1.15

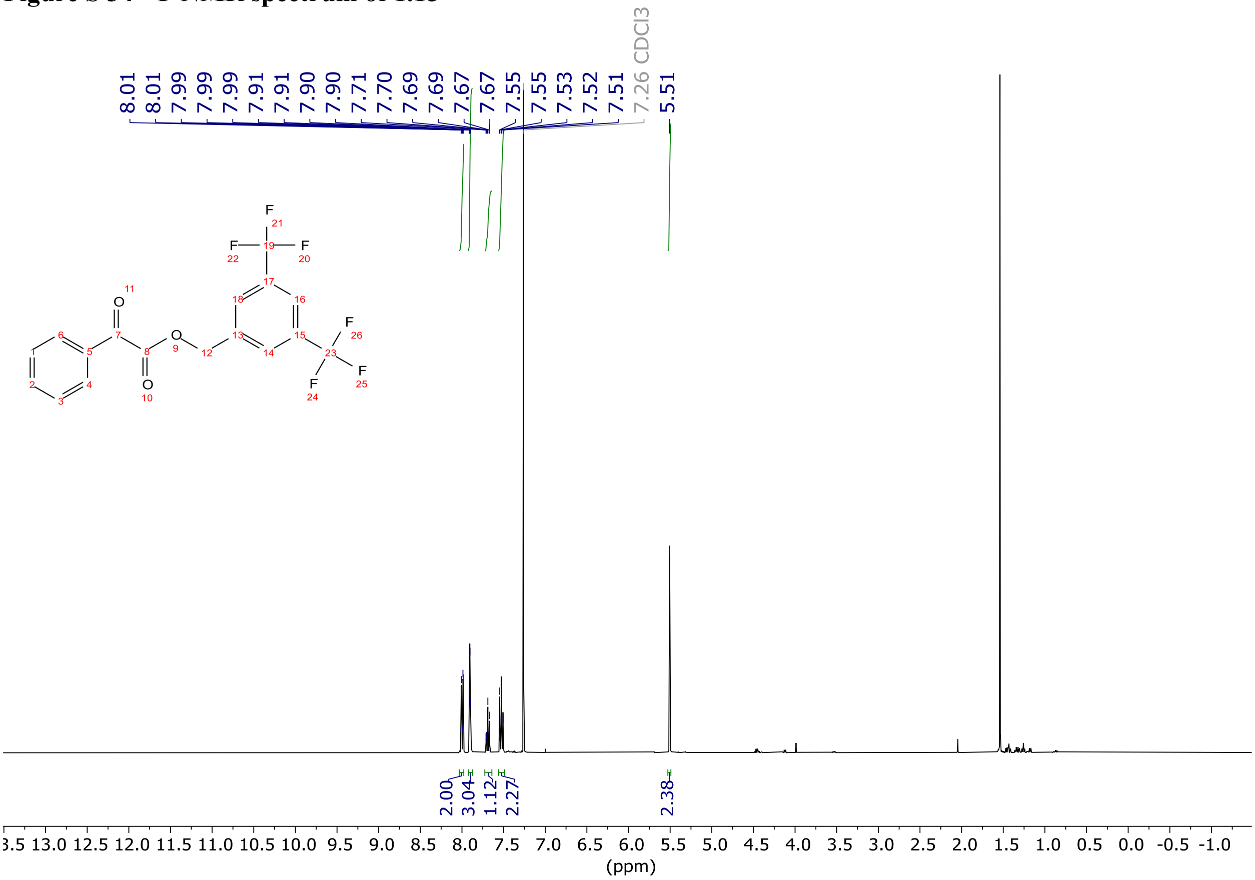

Figure S 35 ^{1}H NMR spectrum of 1.16

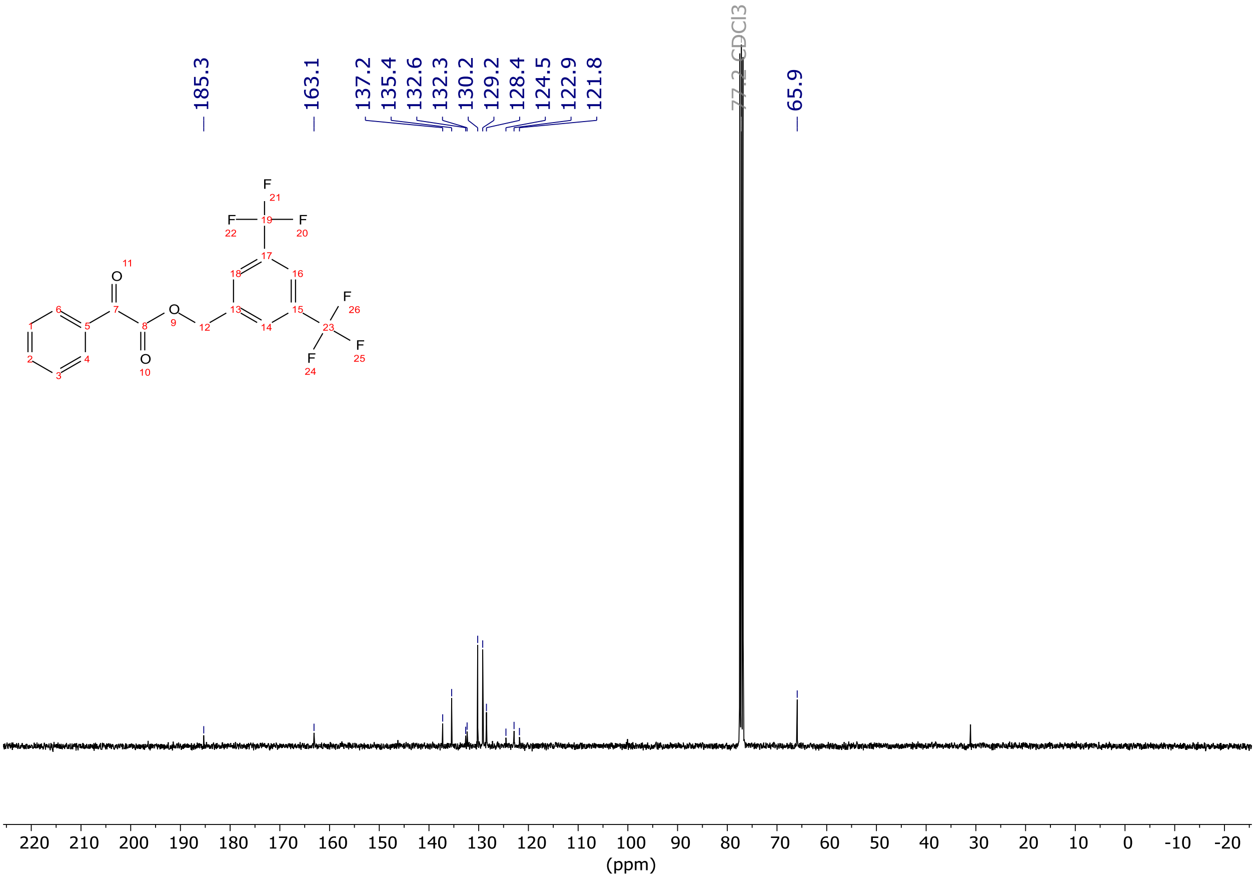

Figure S 36 ^{13}C NMR spectrum of 1.16

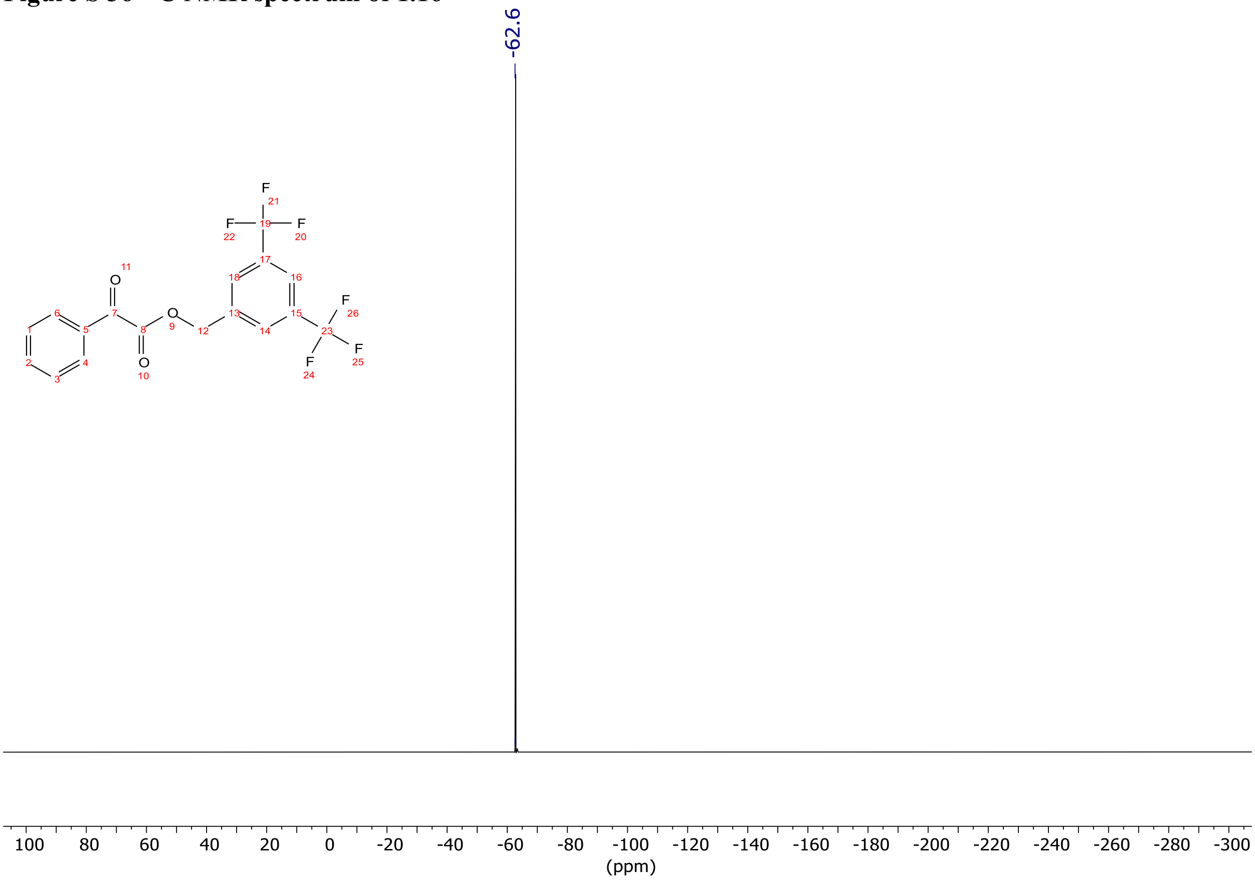

Figure S 37 ^{19}F NMR spectrum of 1.16

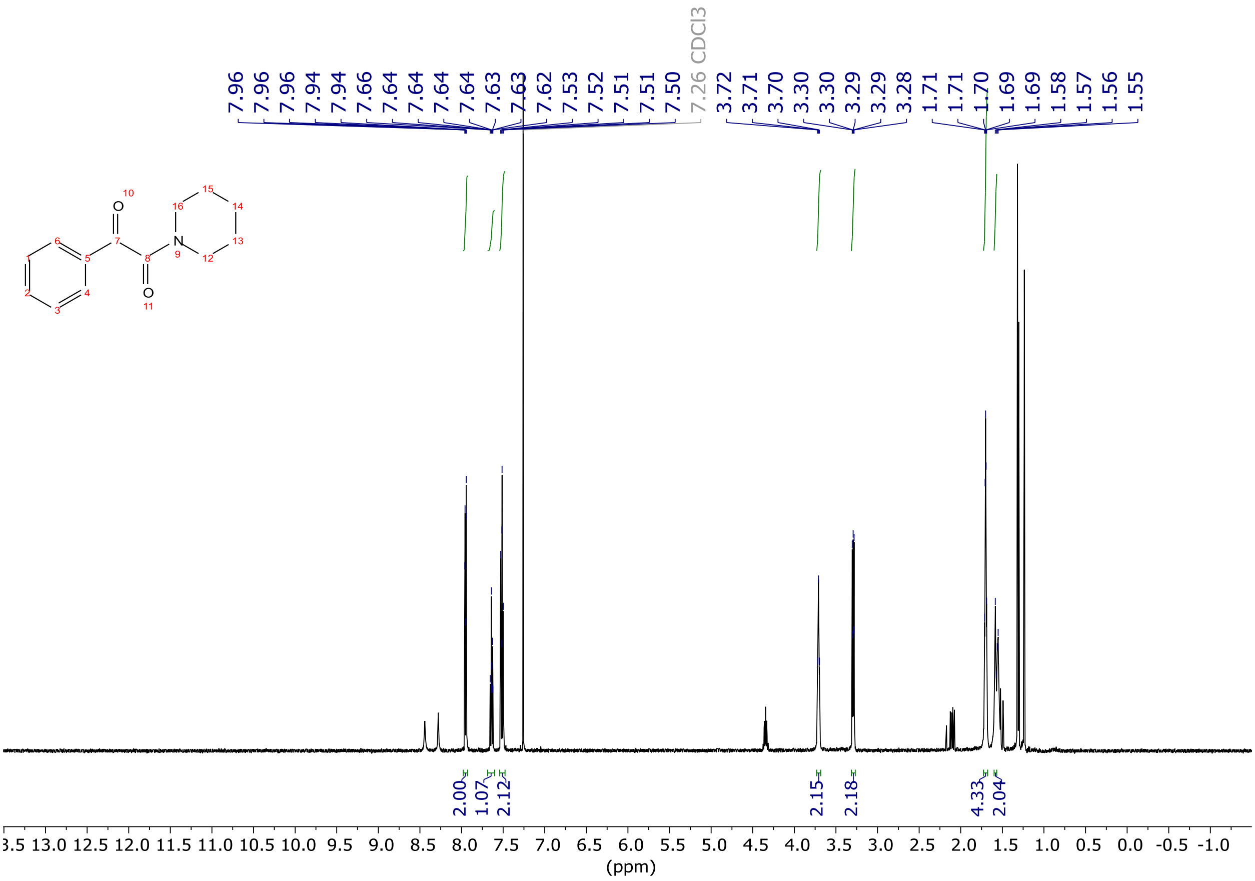

Figure S 38 ^{1}H NMR spectrum of 1.17

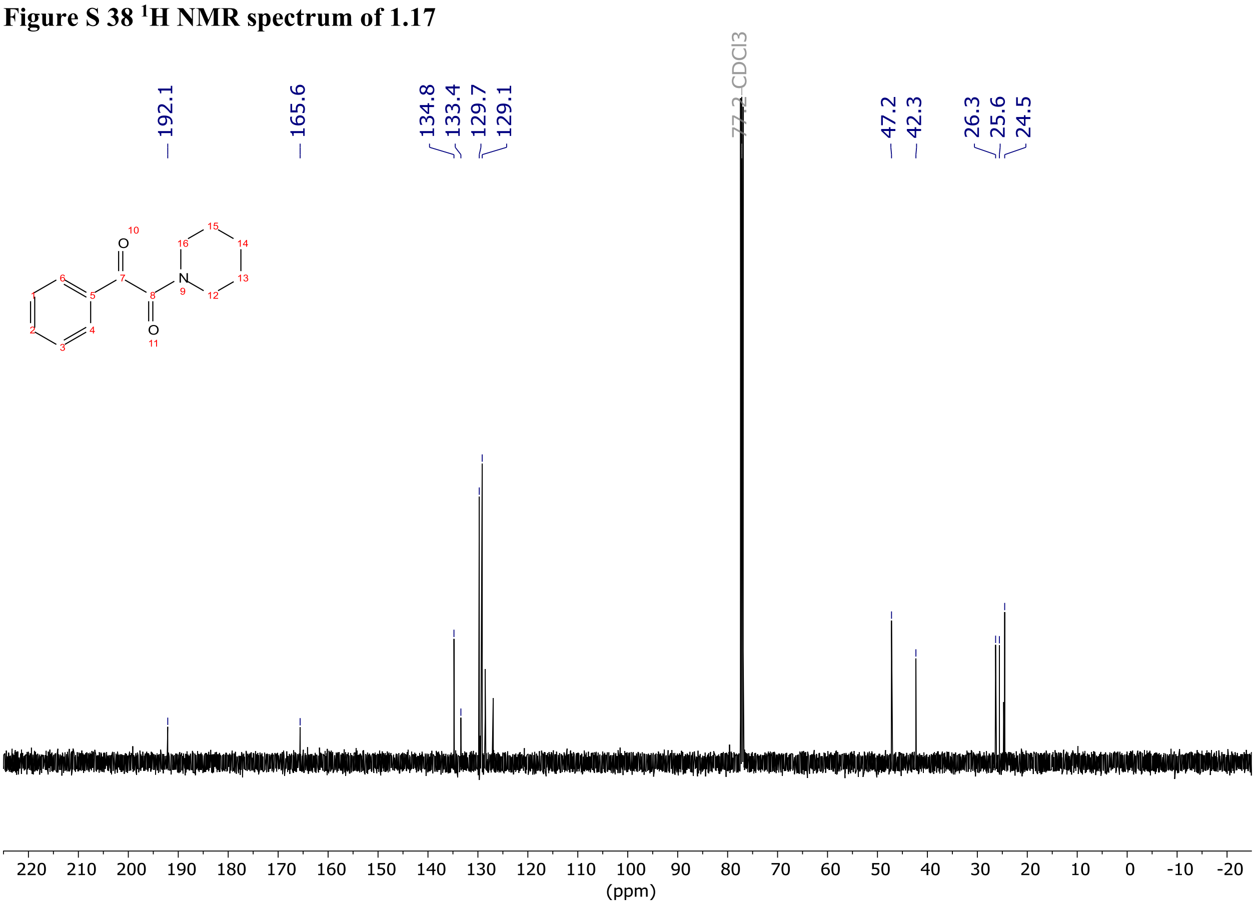

Figure S 39 ^{13}C NMR spectrum of 1.17

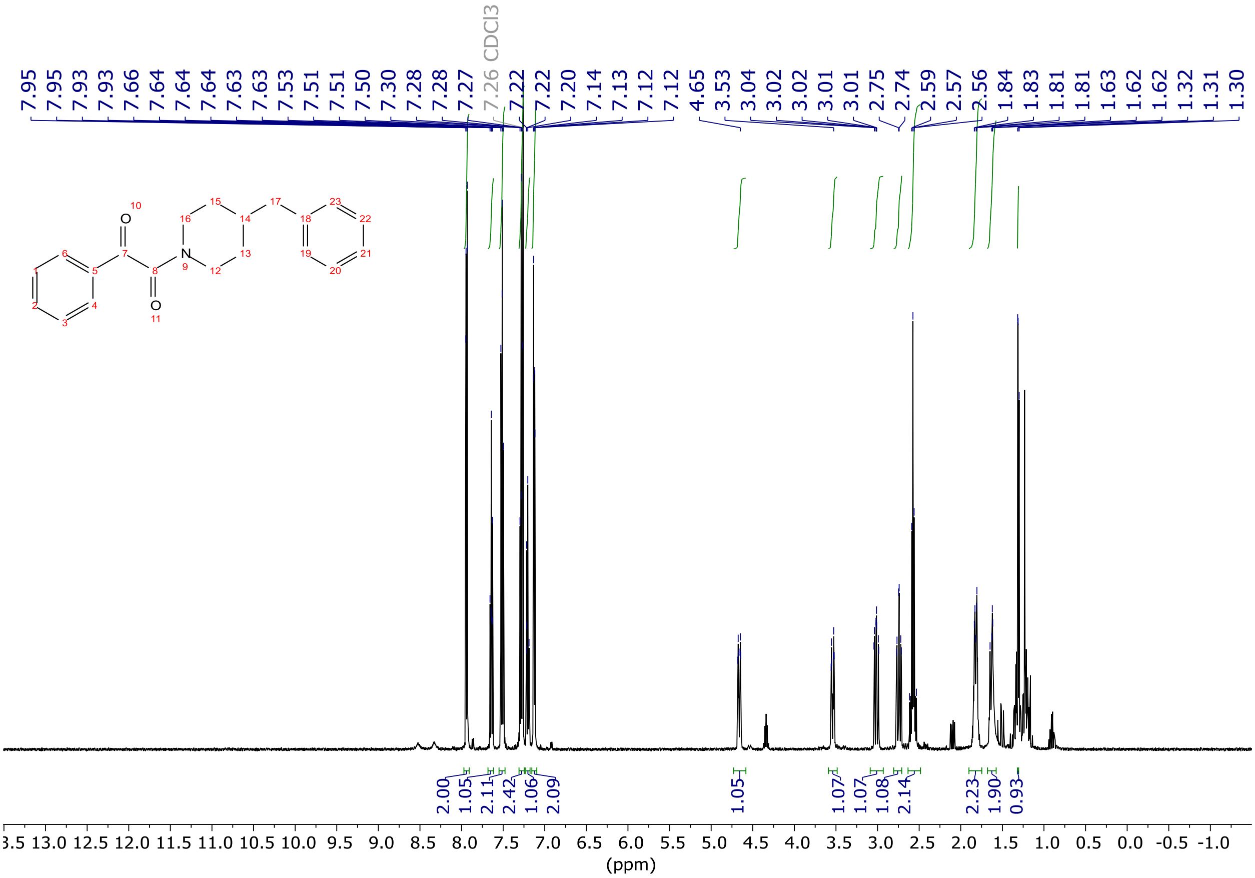

Figure S 40: ^{1}H NMR spectrum of 1.18

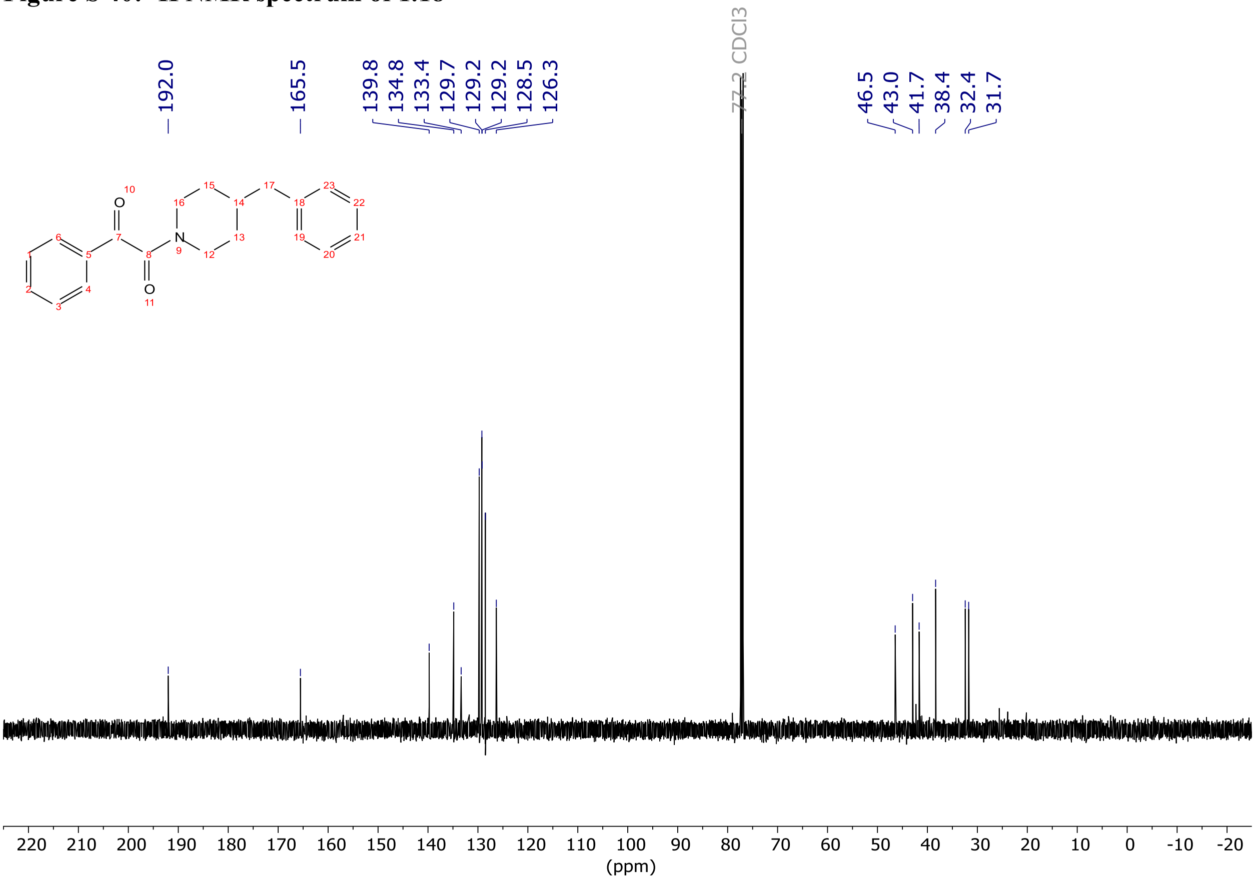

Figure S 41 ^{13}C NMR spectrum of 1.18

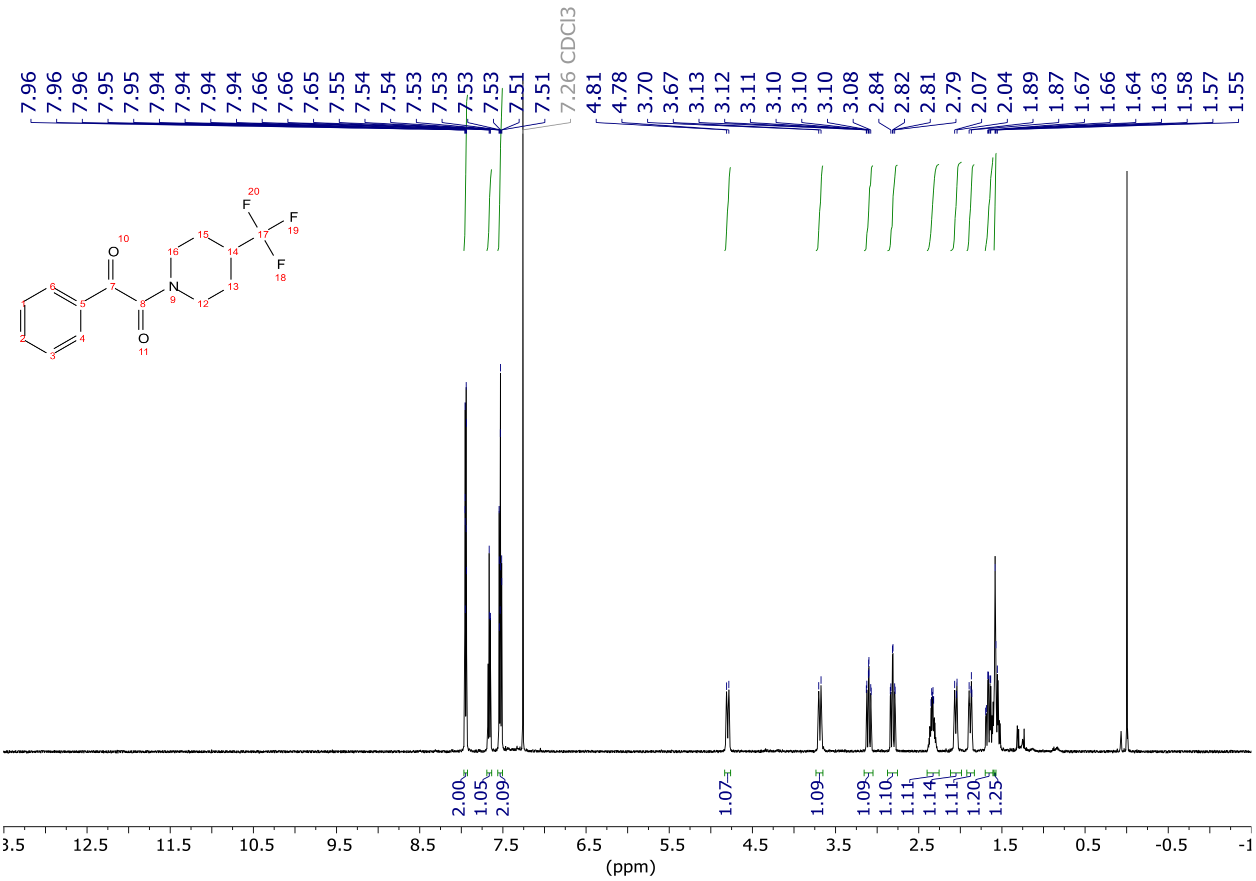

Figure S 42 ^{1}H NMR spectrum of 1.19

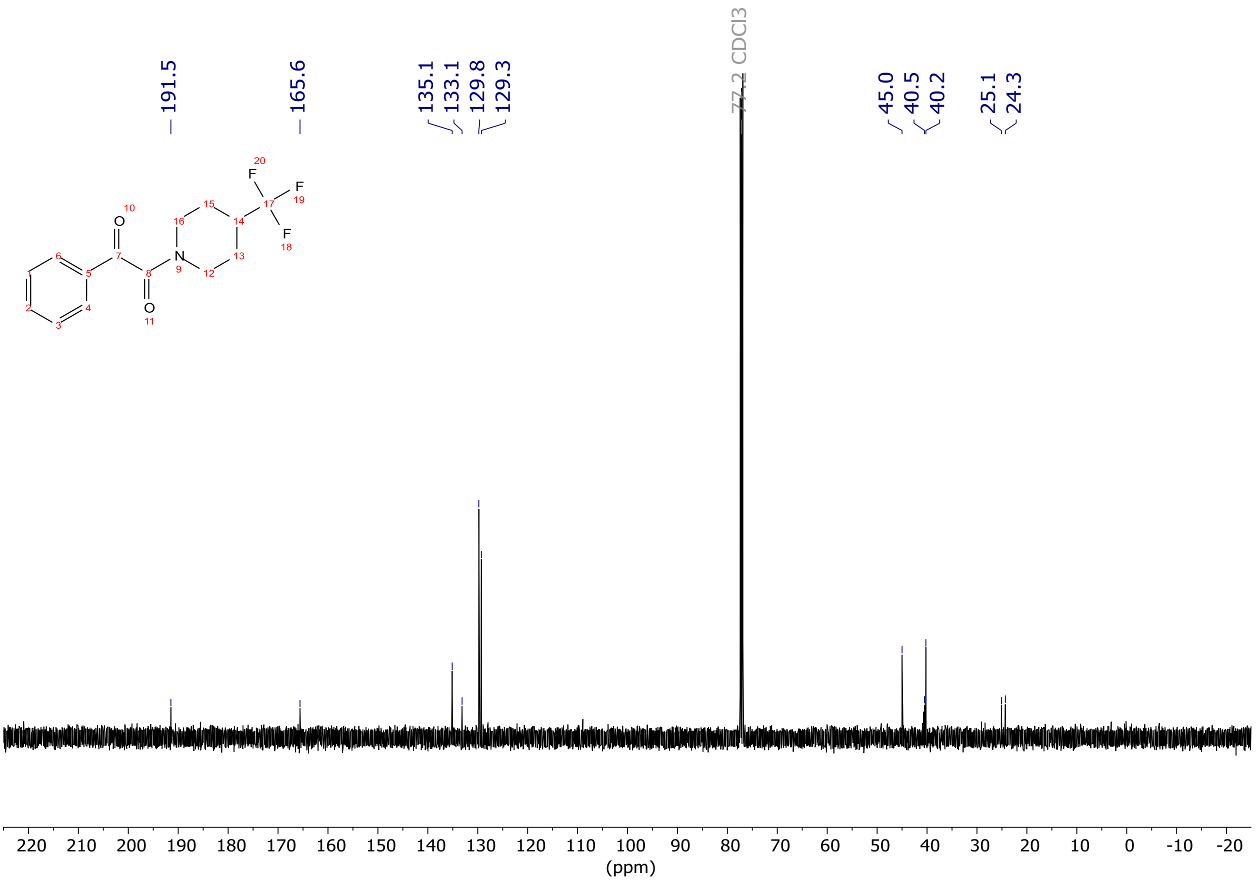

Figure S 43 ^{13}C NMR spectrum of 1.19

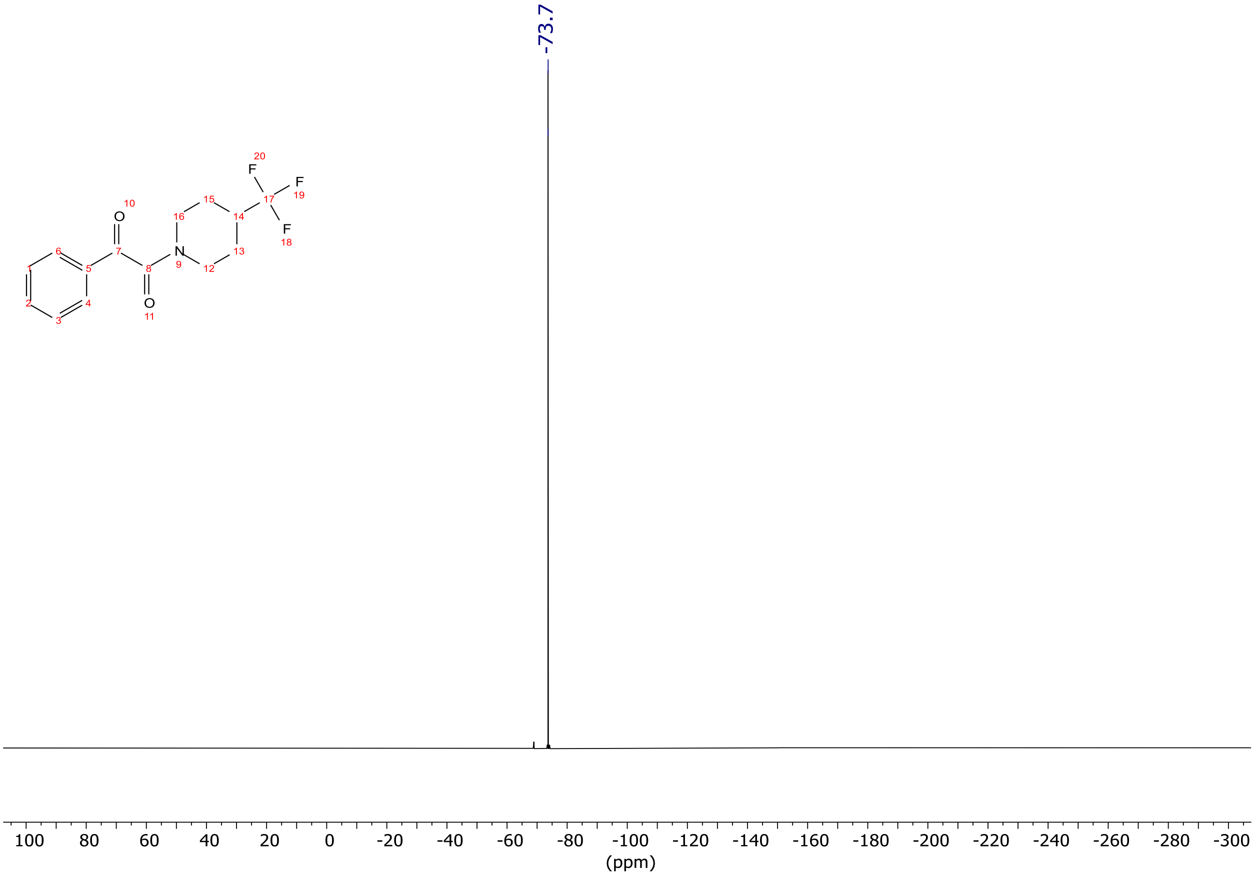

Figure S 44 ^{19}F NMR spectrum of 1.19

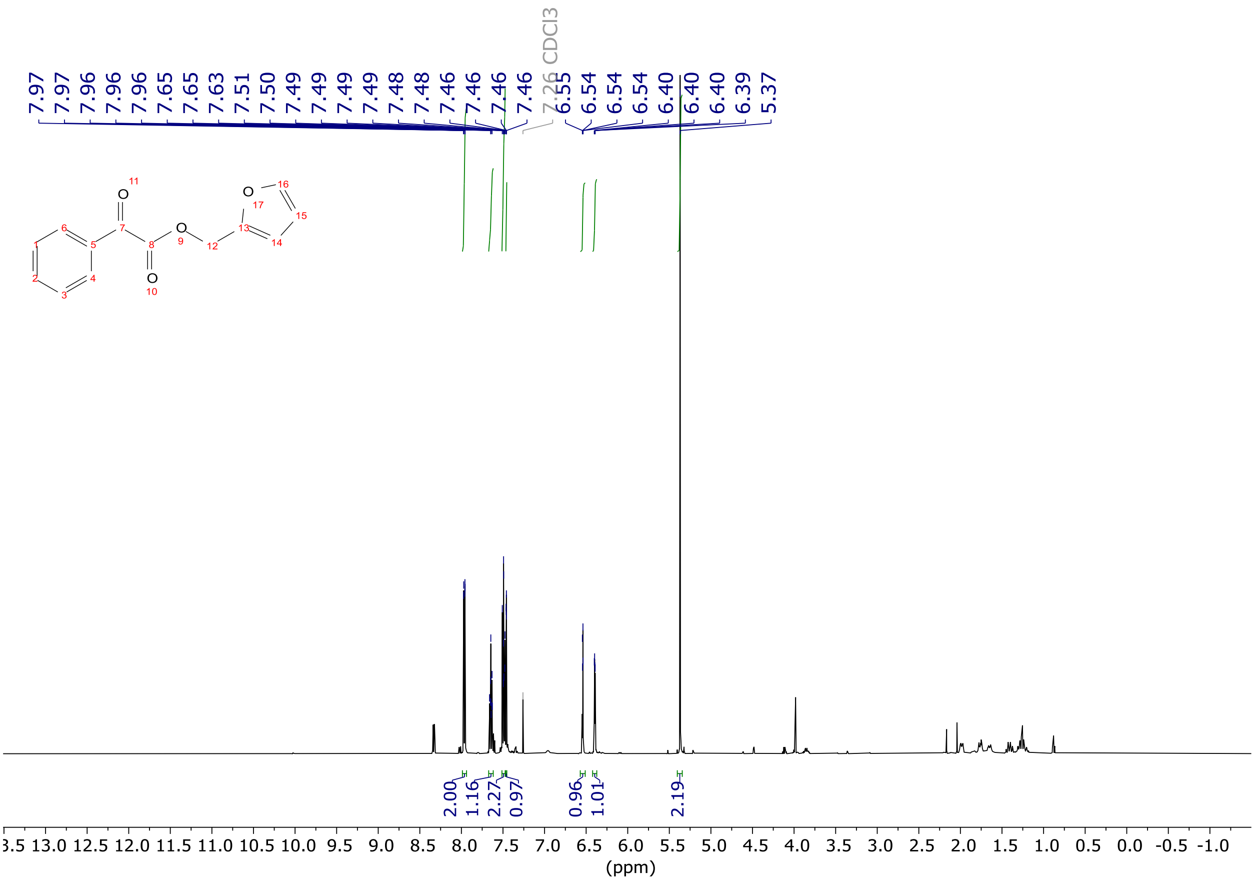

Figure S 45 ^{1}H NMR spectrum of 1.20

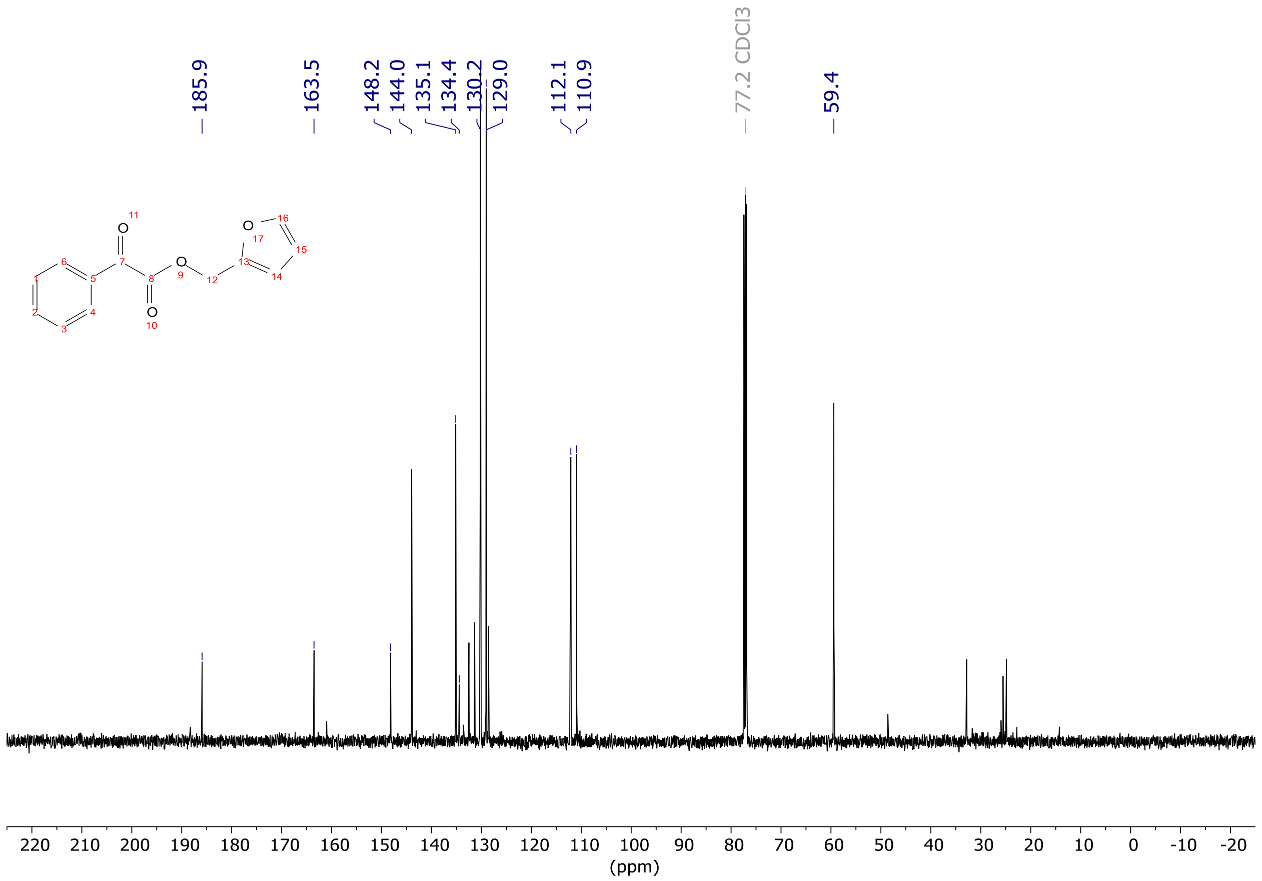

Figure S 46 ^{13}C NMR spectrum of 1.20

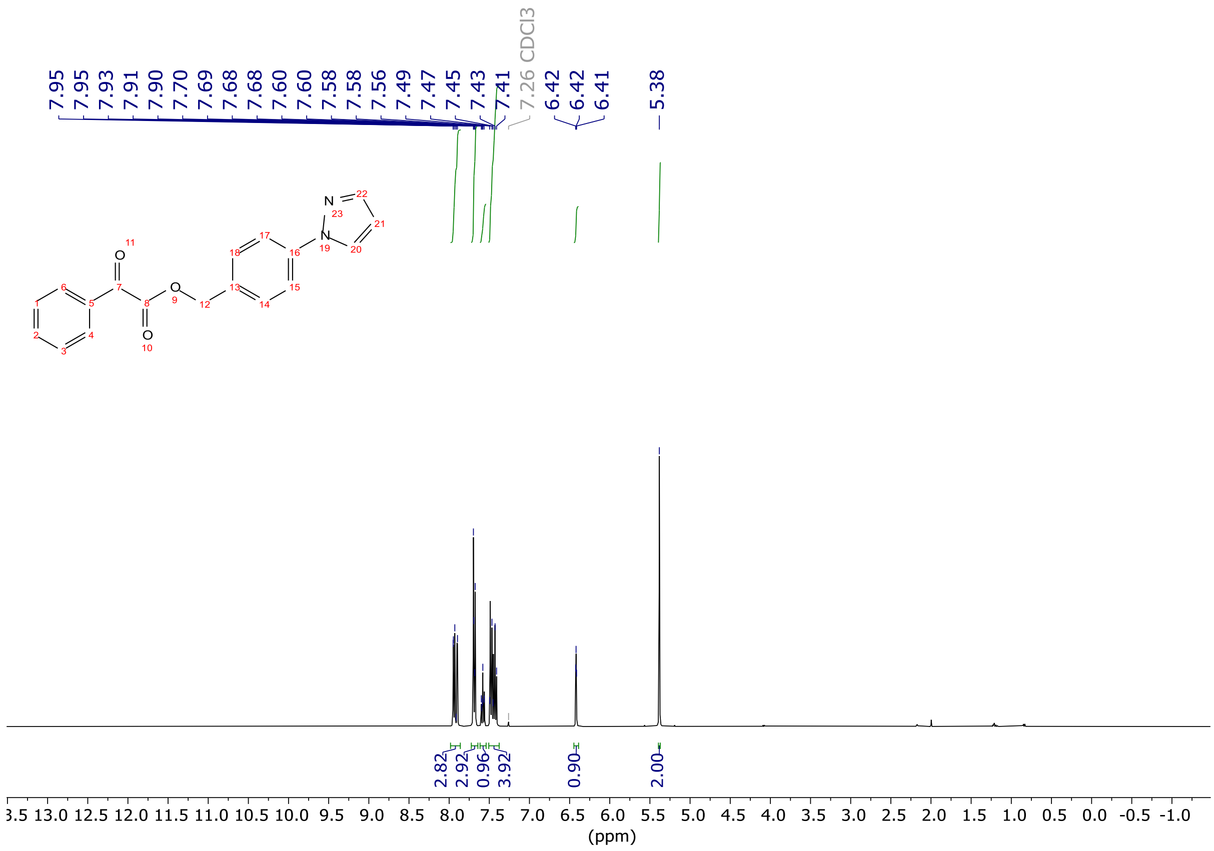

Figure S 47 ^{1}H NMR spectrum of 1.21

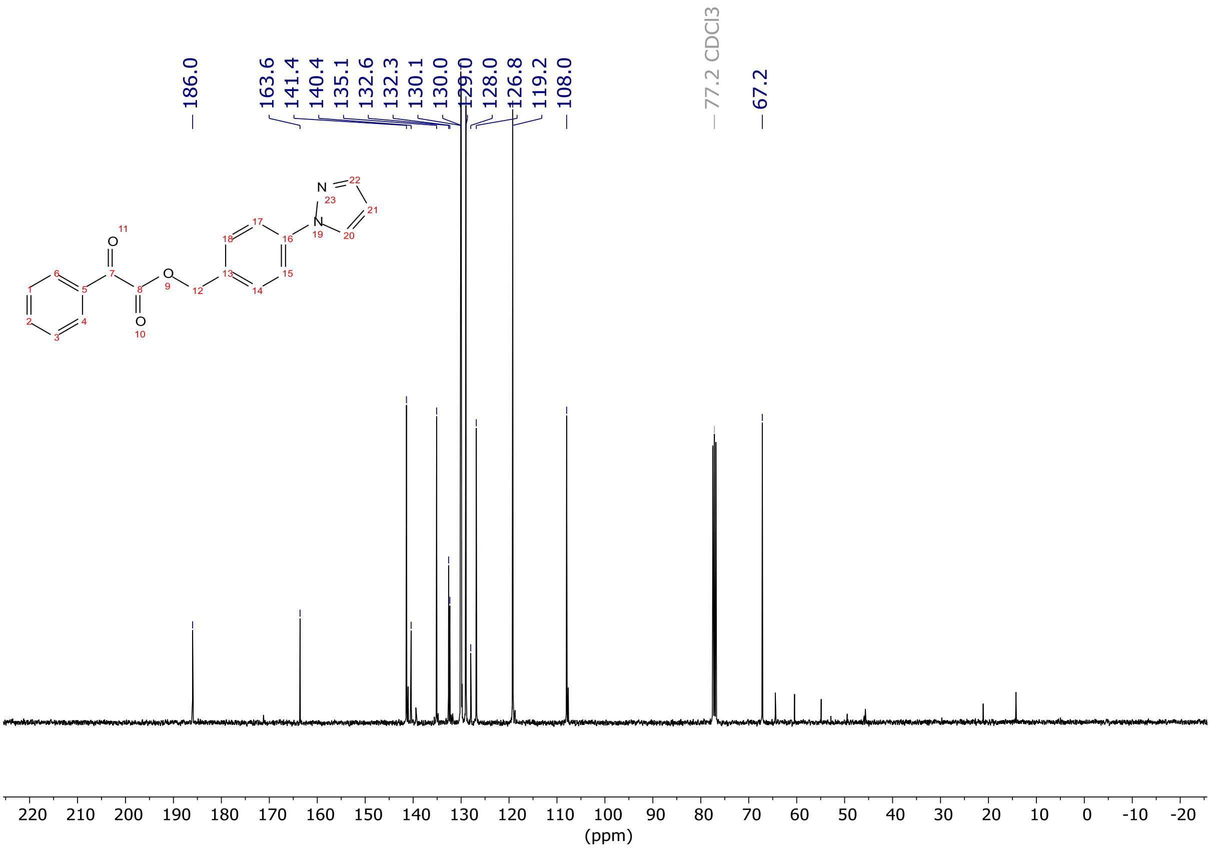

Figure S 48 ^{13}C NMR spectrum of 1.21

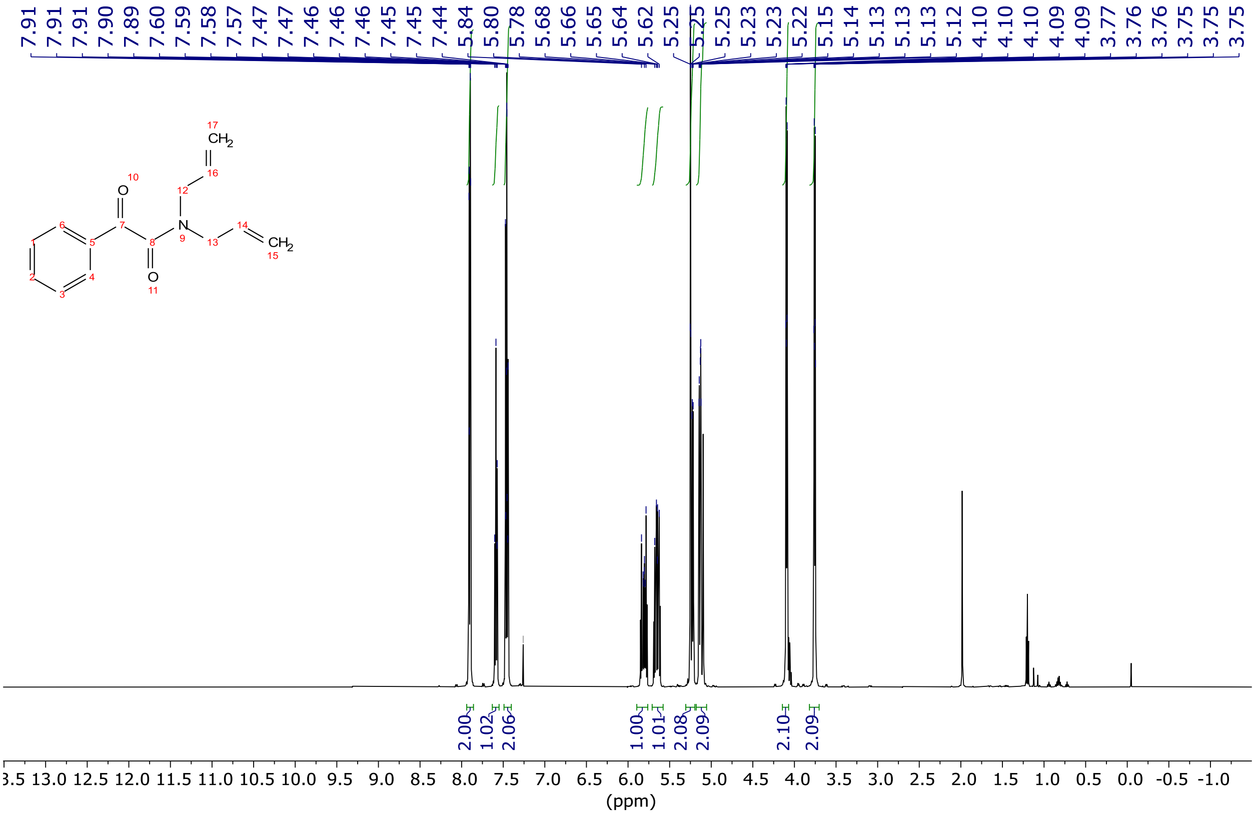

Figure S 49 ^{1}H NMR spectrum of 1.22

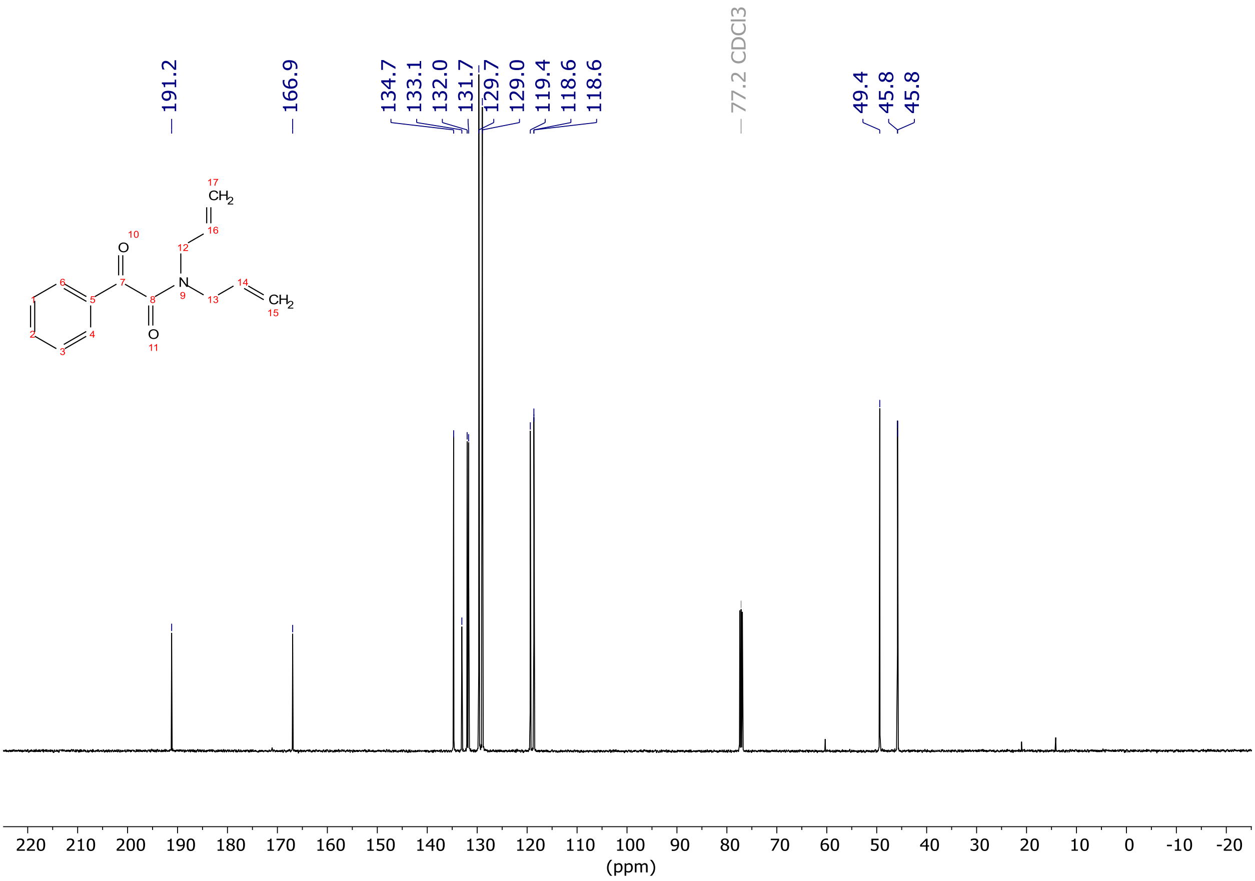

Figure S 50 ^{13}C NMR spectrum of 1.22

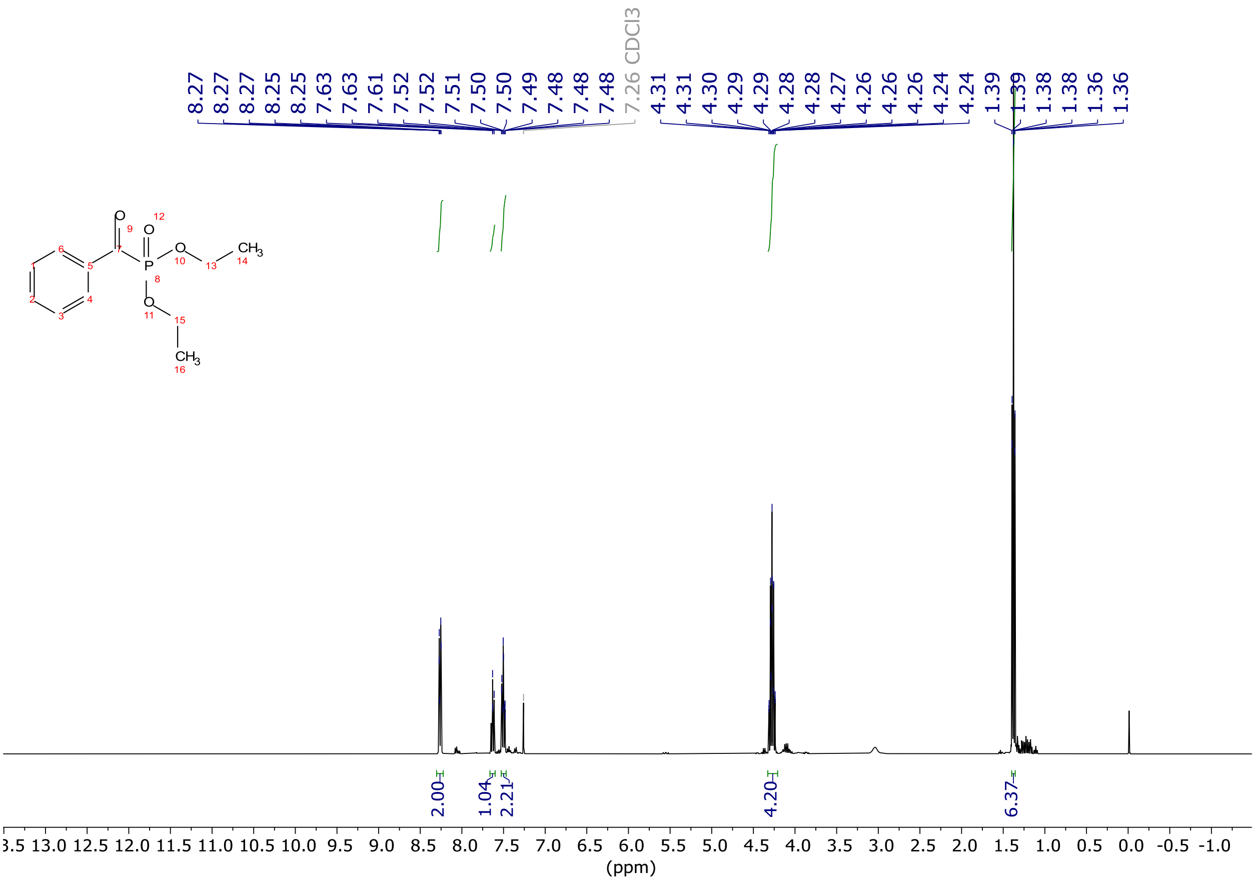

Figure S 51 ^{1}H NMR spectrum of 1.23

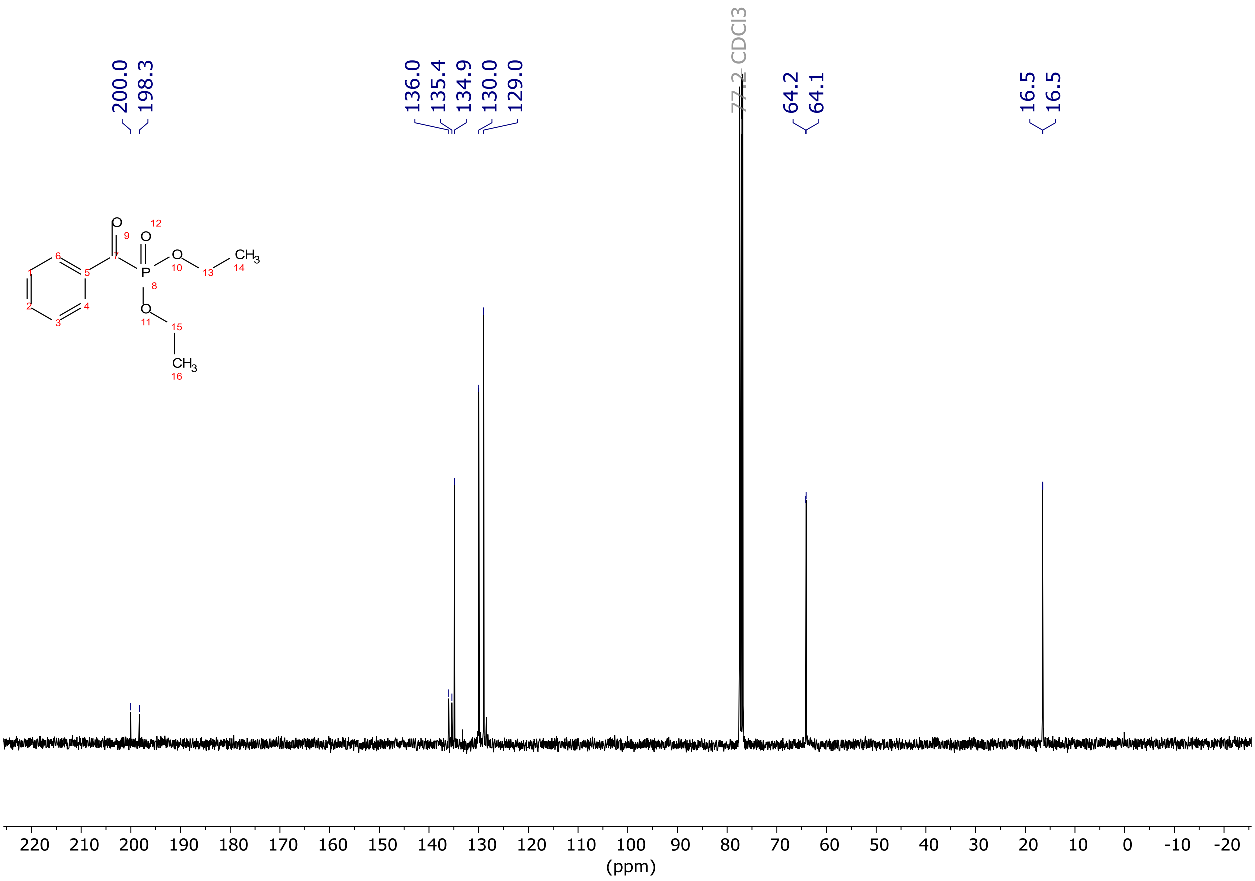

Figure S 52 ^{13}C NMR spectrum of 1.23

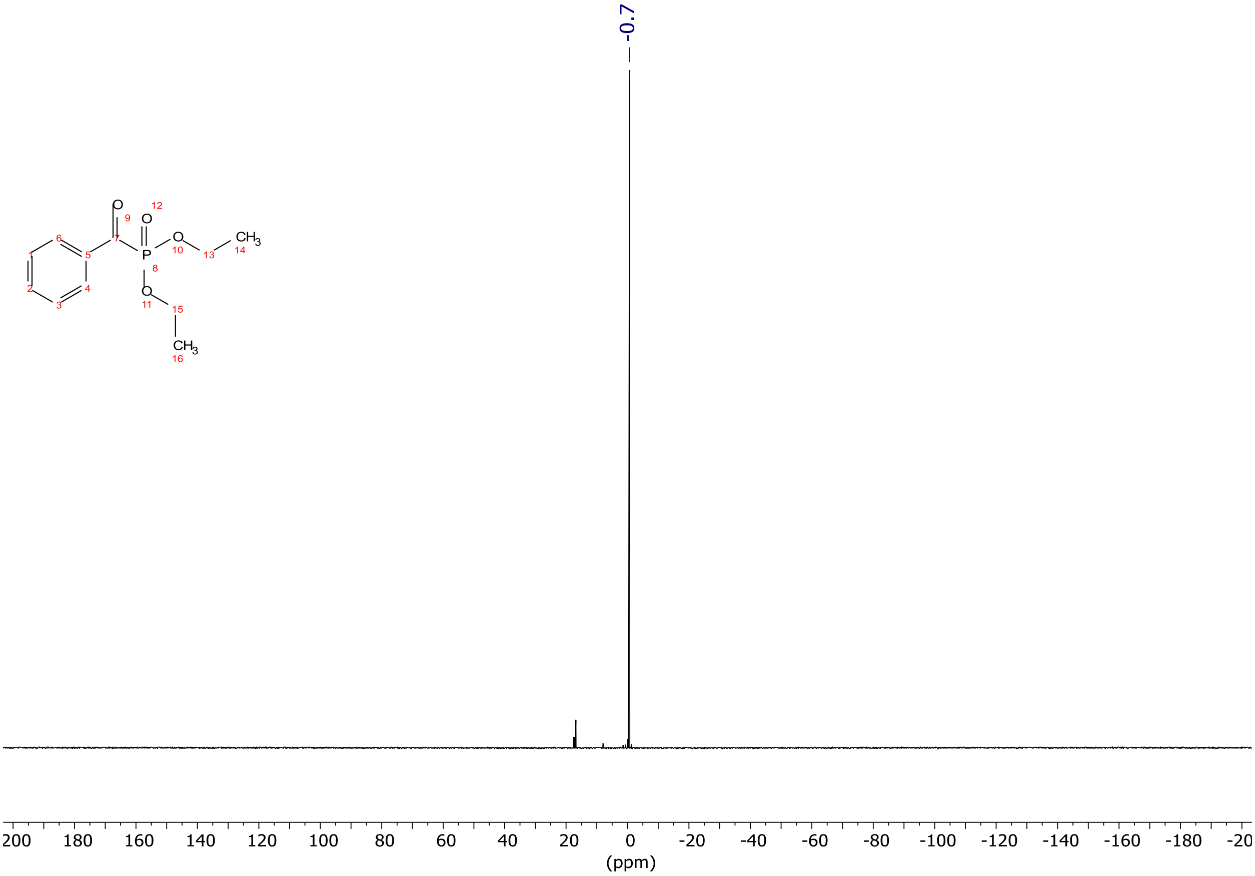

Figure S 53 ^{31}P NMR spectrum of 1.23

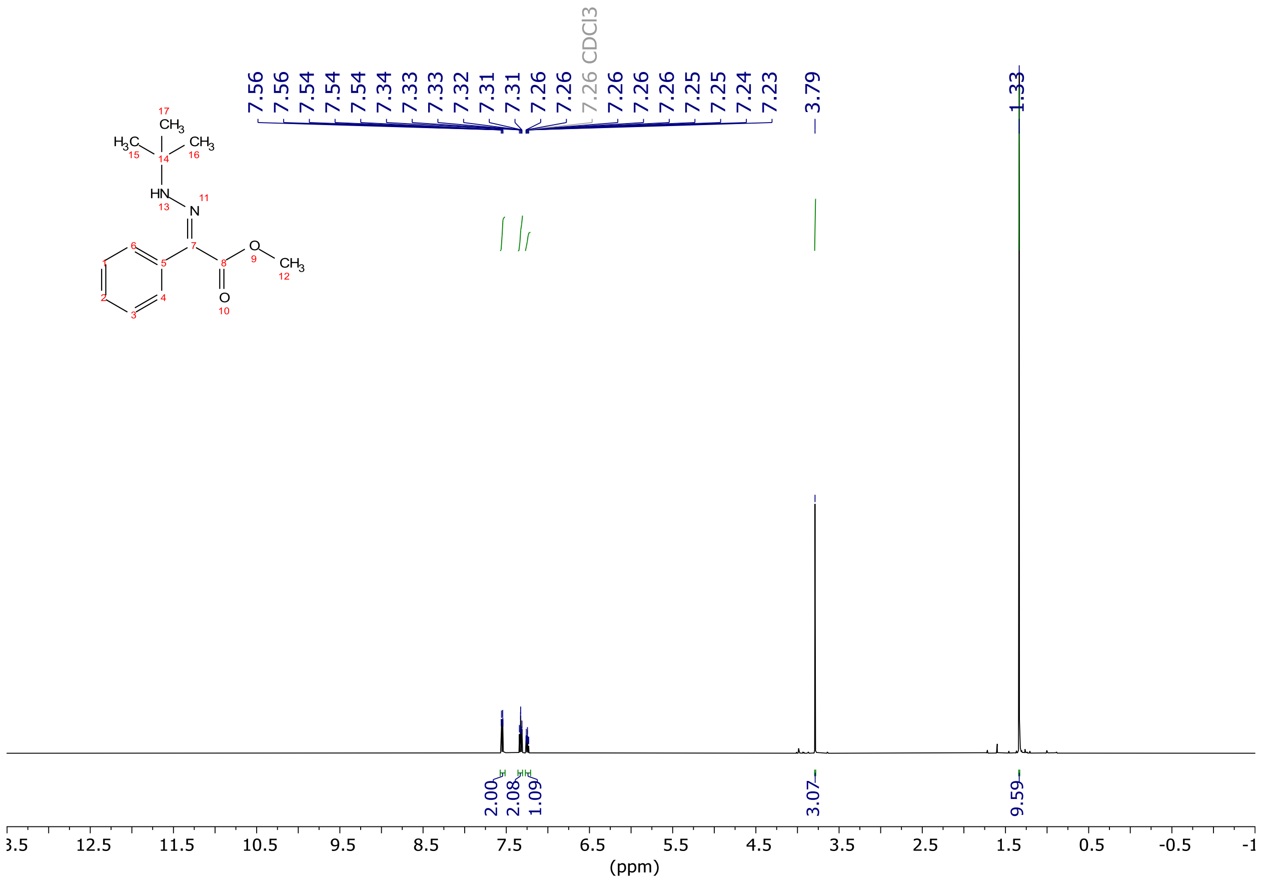

Figure S 54 ^{1}H NMR spectrum of 2.1

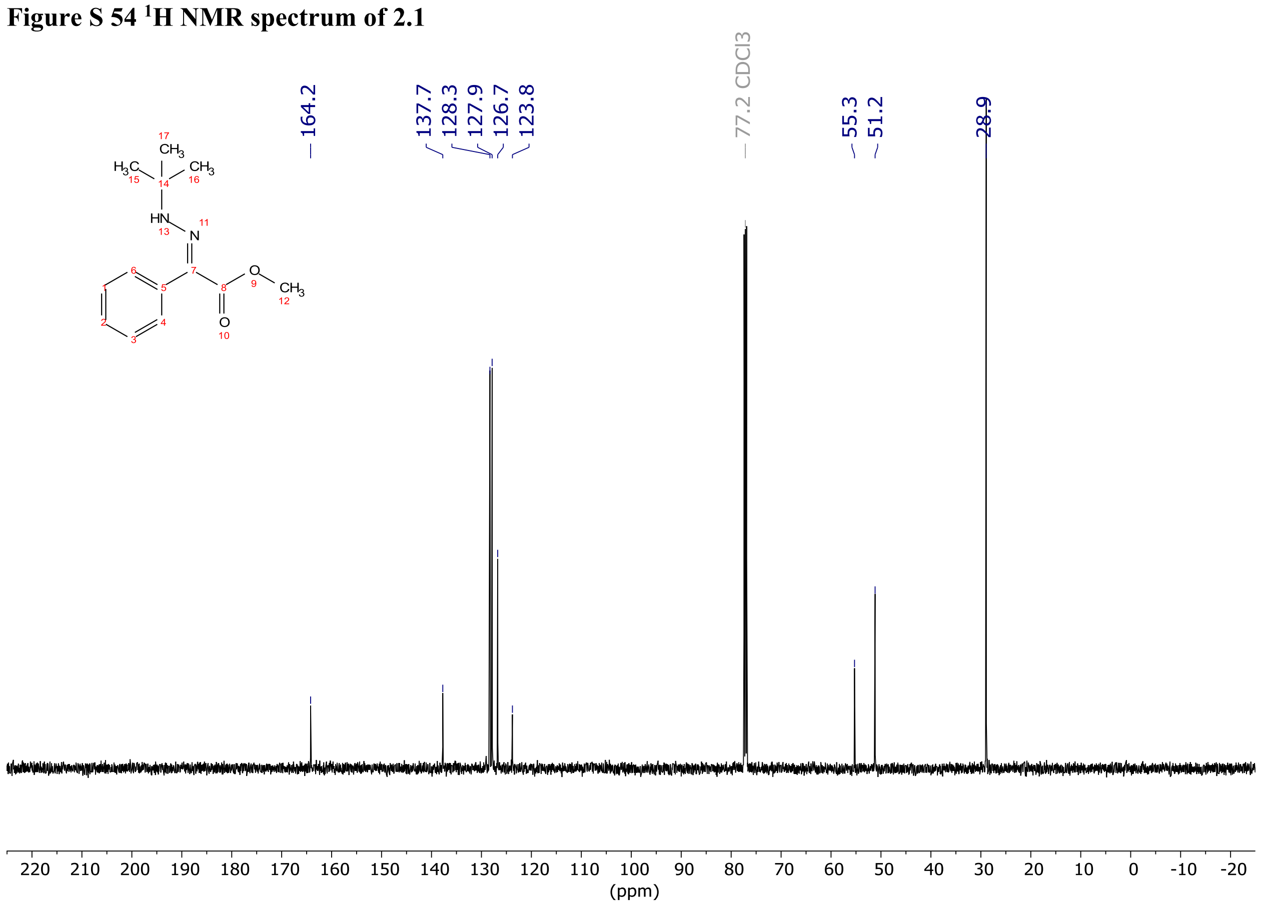

Figure S 55 ^{13}C NMR spectrum of 2.1

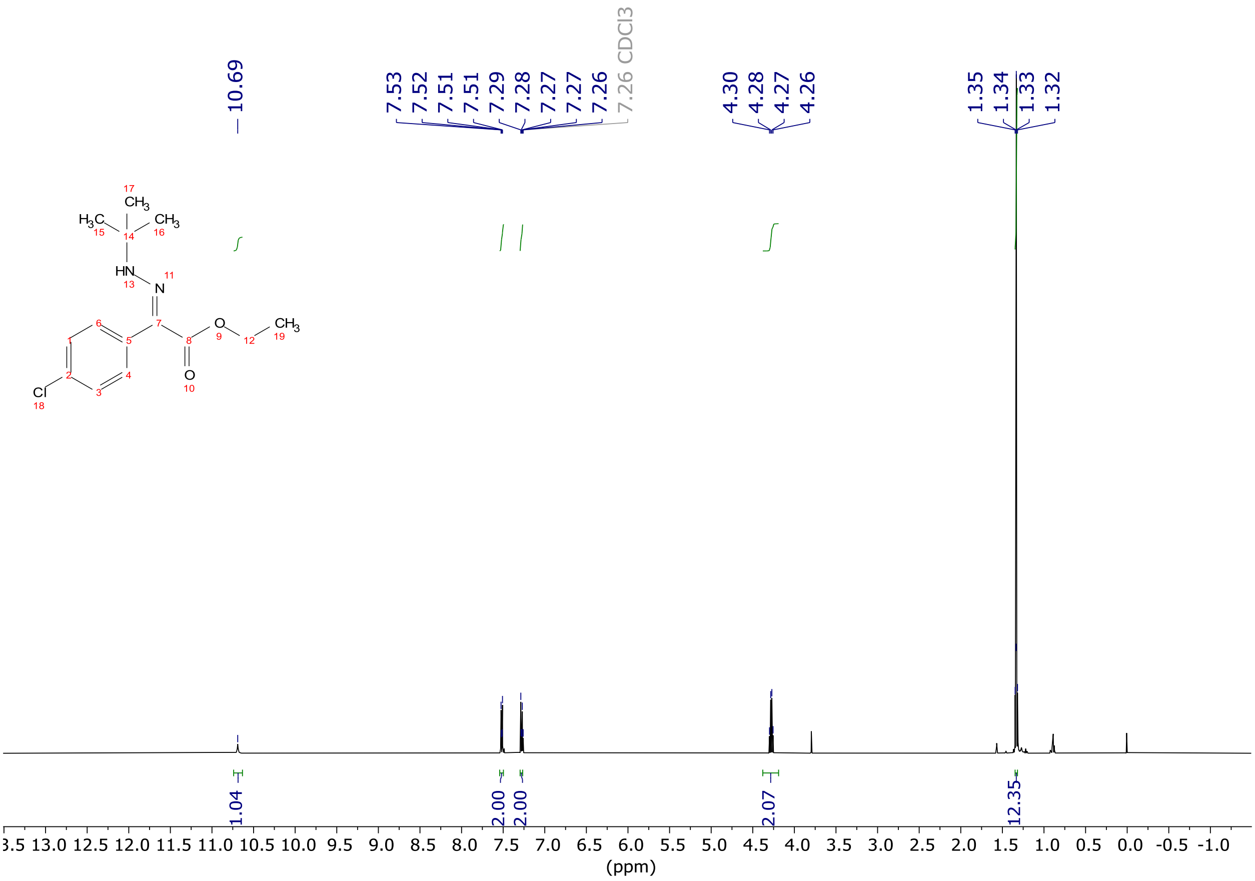

Figure S 56 ^{1}H NMR spectrum of 2.2

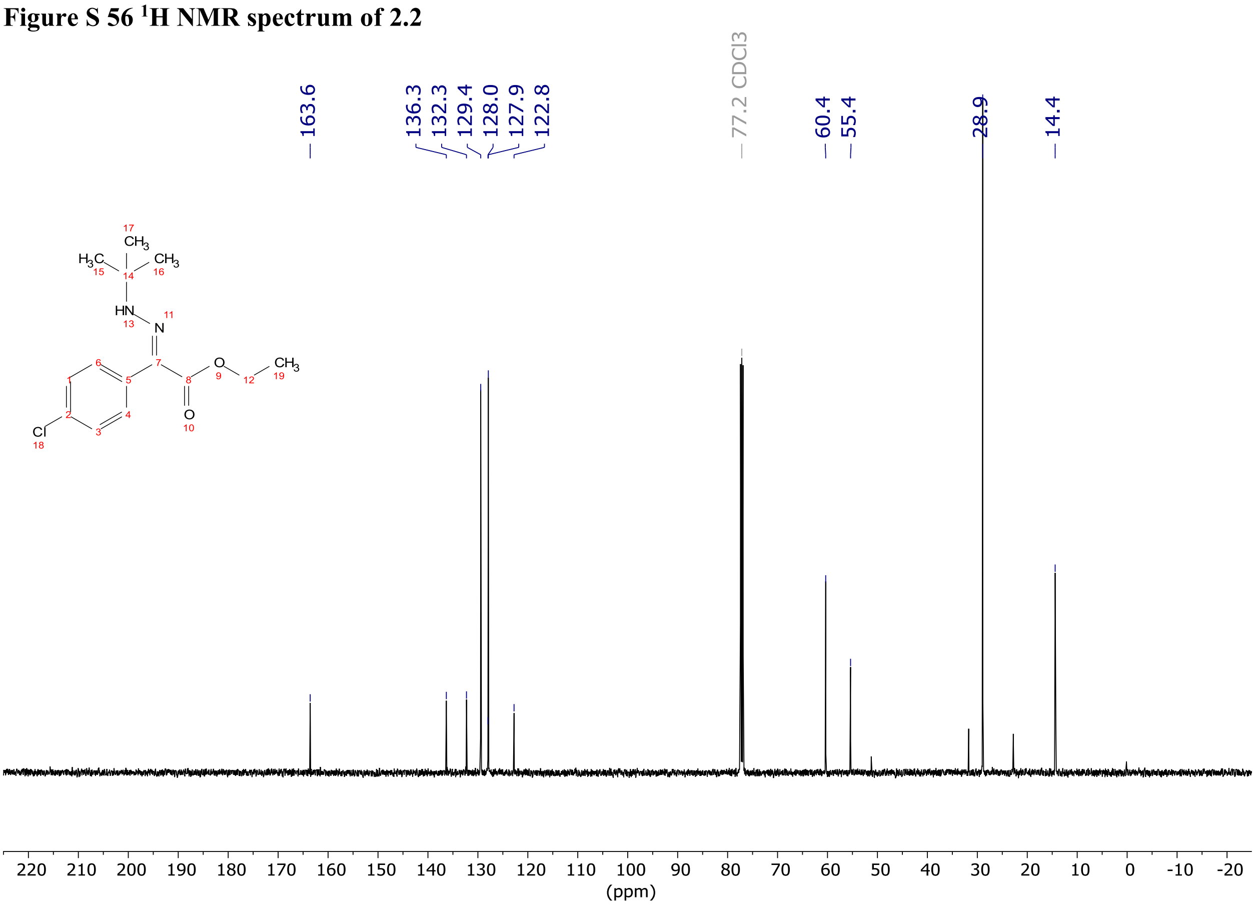

Figure S 57 ^{13}C NMR spectrum of 2.2

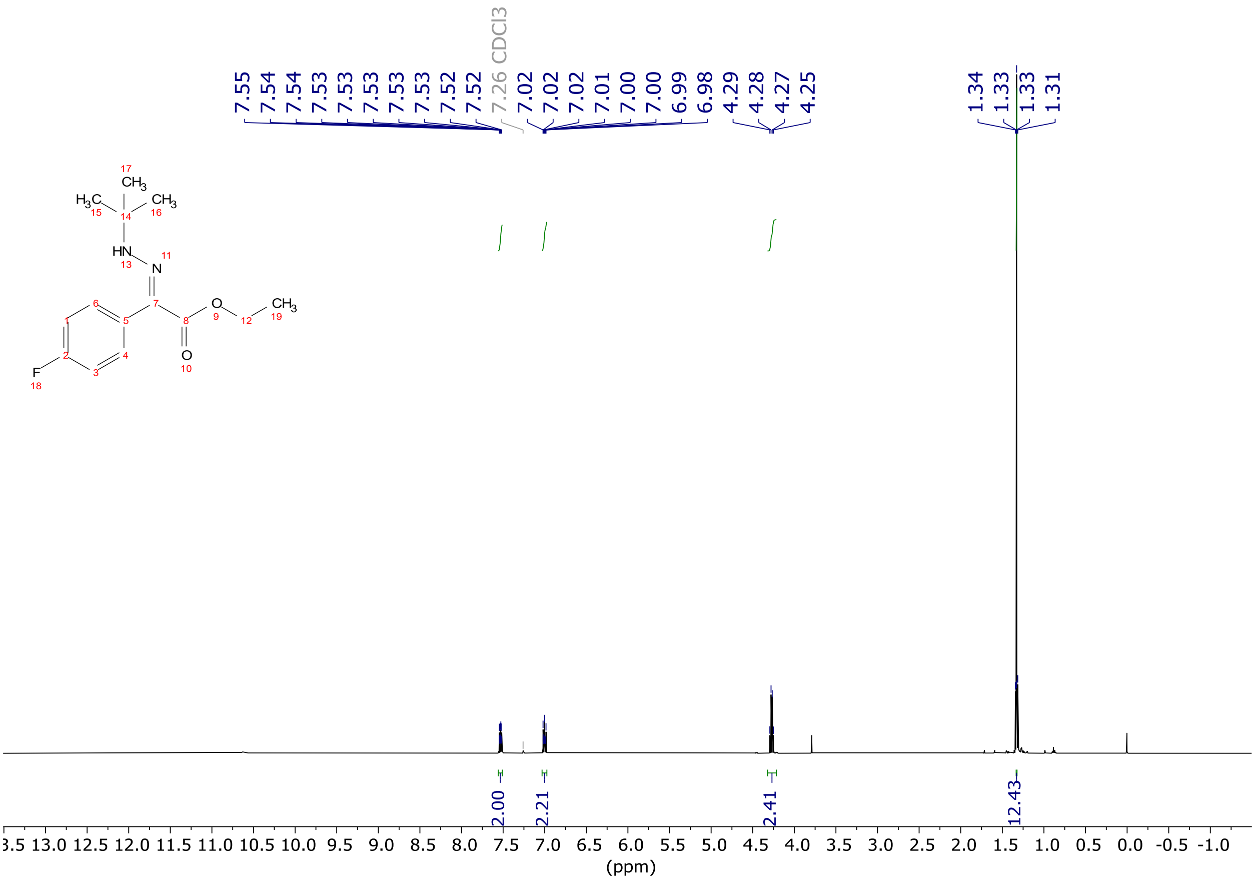

Figure S 58 ^{1}H NMR spectrum of 2.3

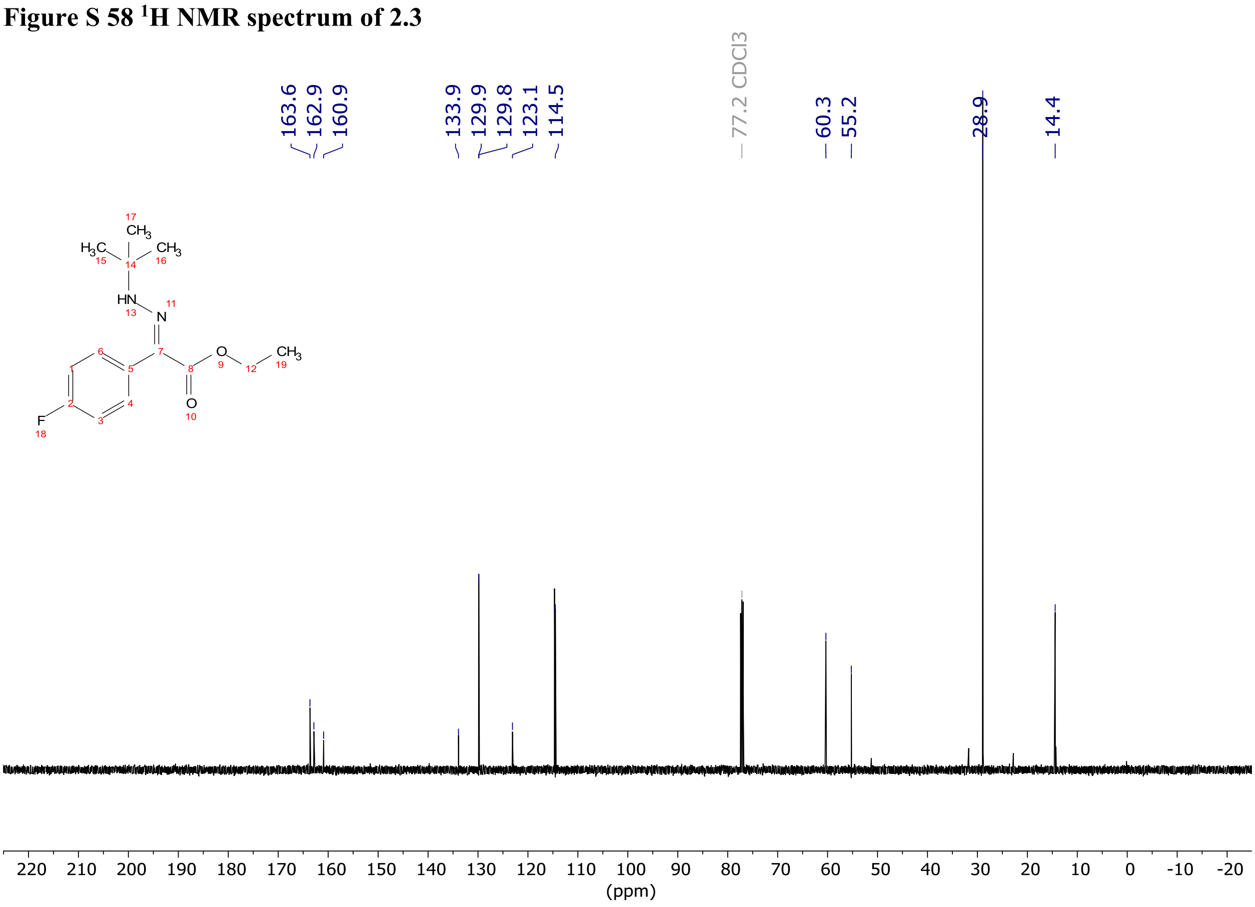

Figure S 59 ^{13}C NMR spectrum of 2.3

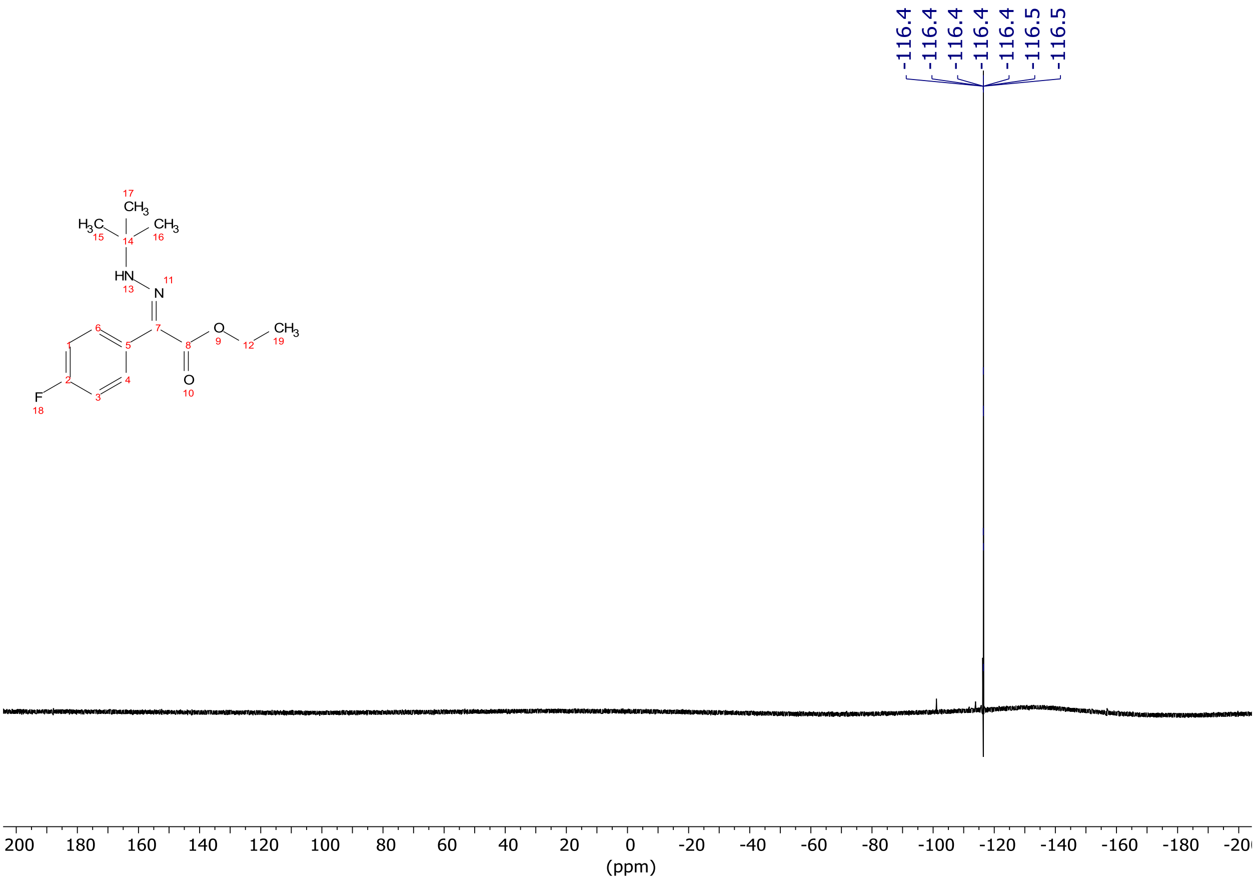

Figure S 60 ^{19}F NMR spectrum of 2.3

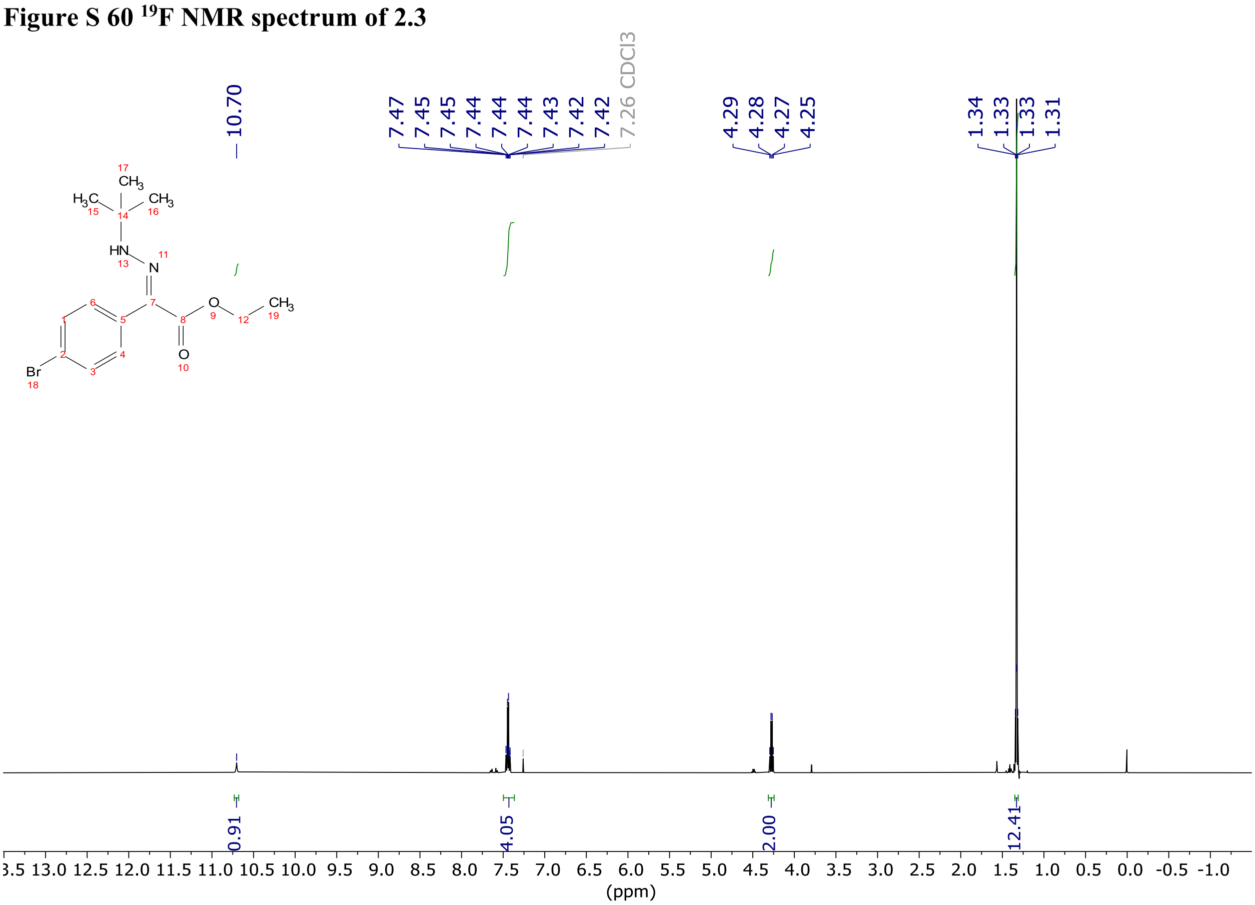

Figure S 61 ^{1}H NMR spectrum of 2.4

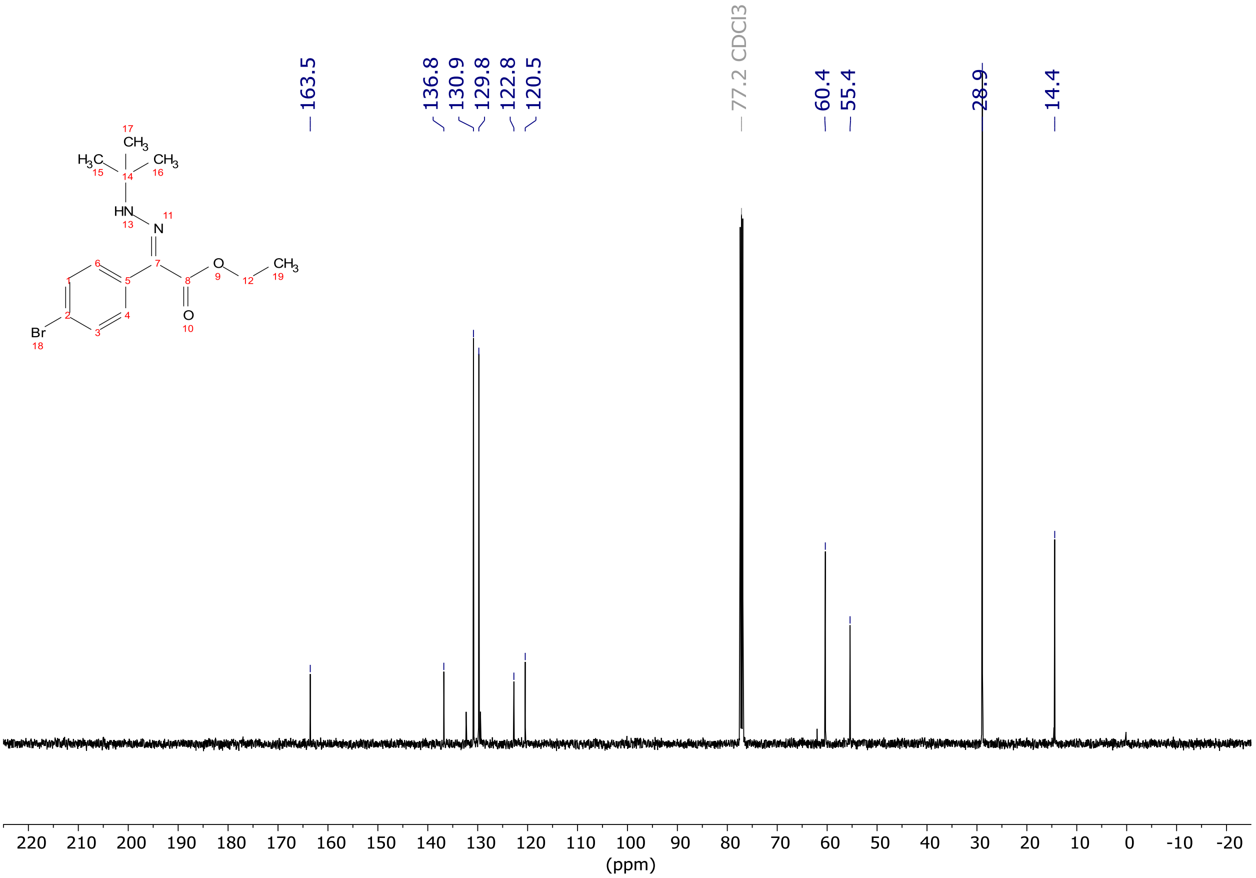

Figure S 62 ^{13}C NMR spectrum of 2.4

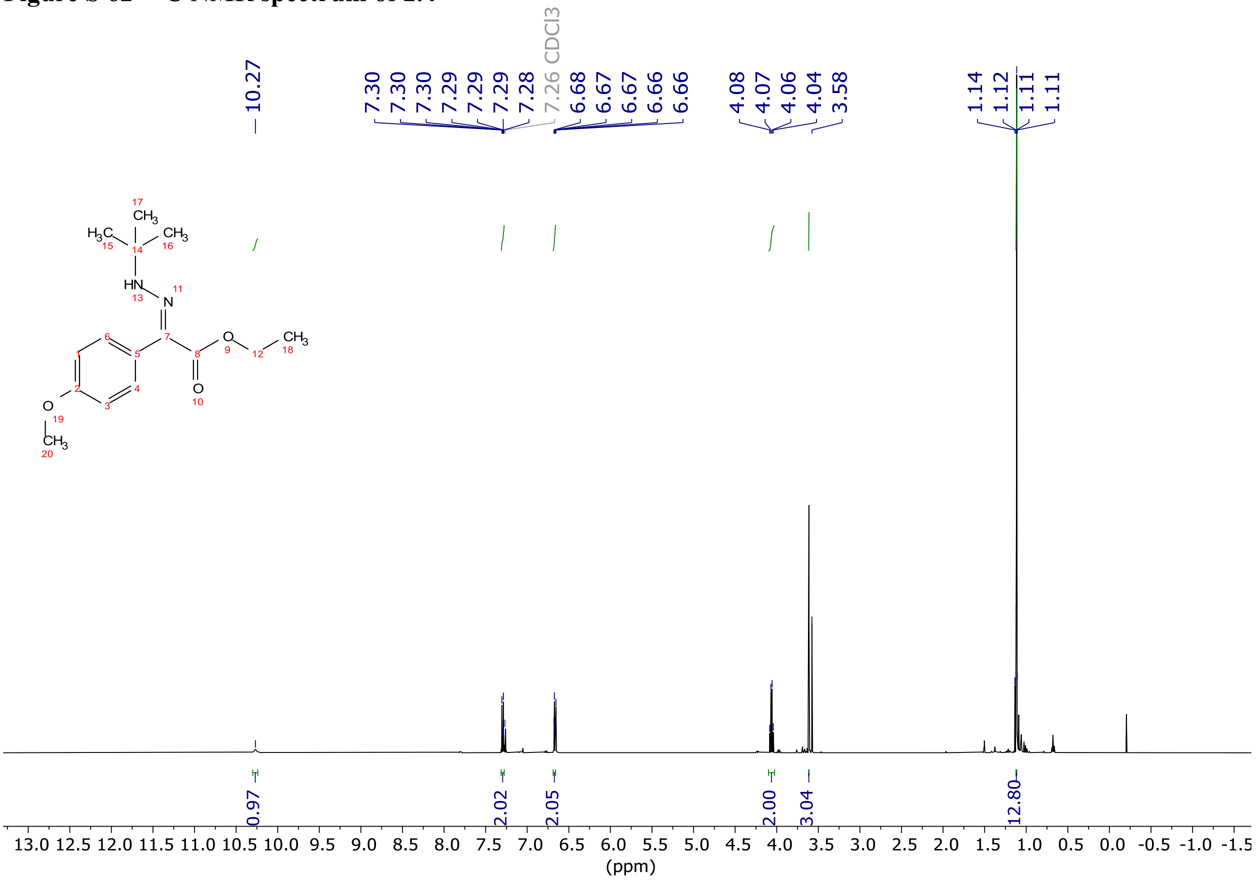

Figure S 63 ^{1}H NMR spectrum of 2.5

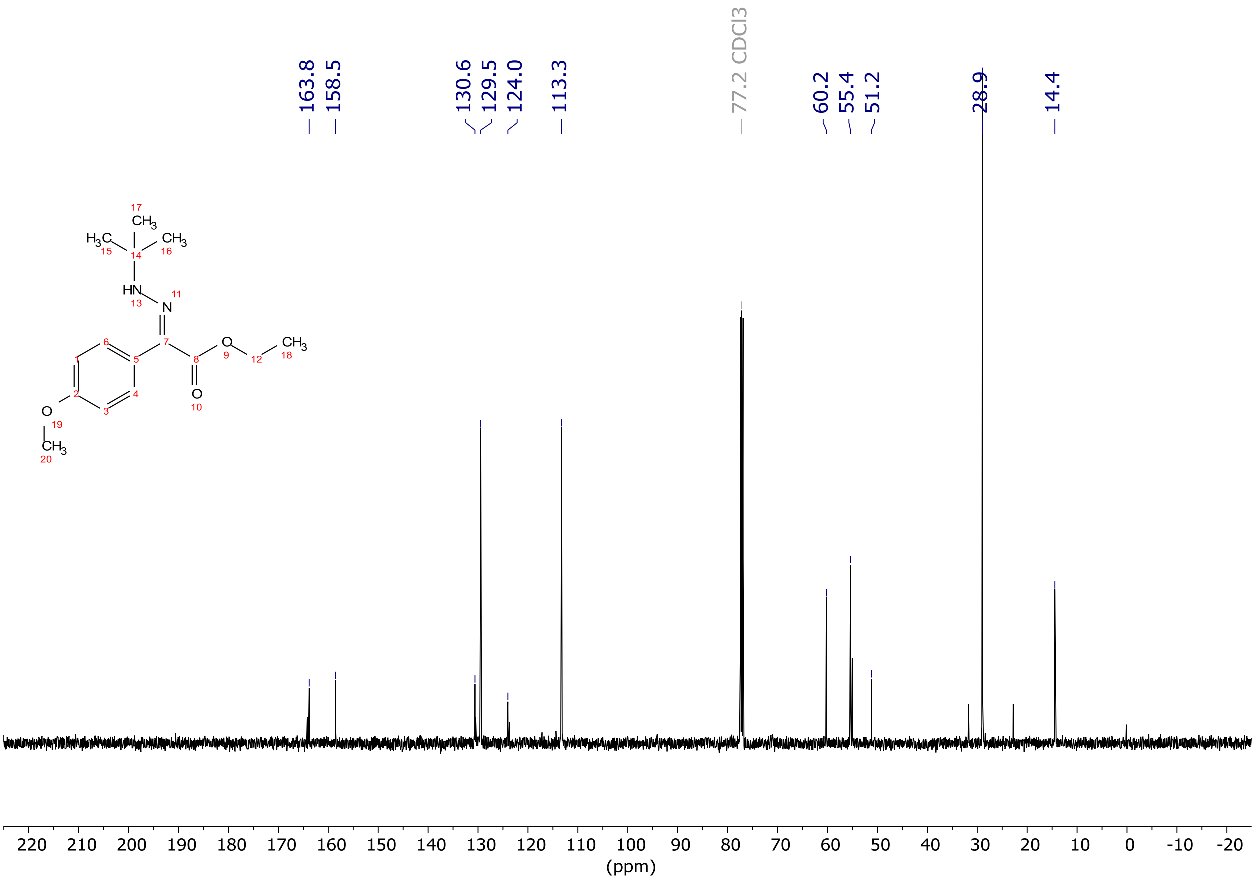

Figure S 64 ^{13}C NMR spectrum of 2.5

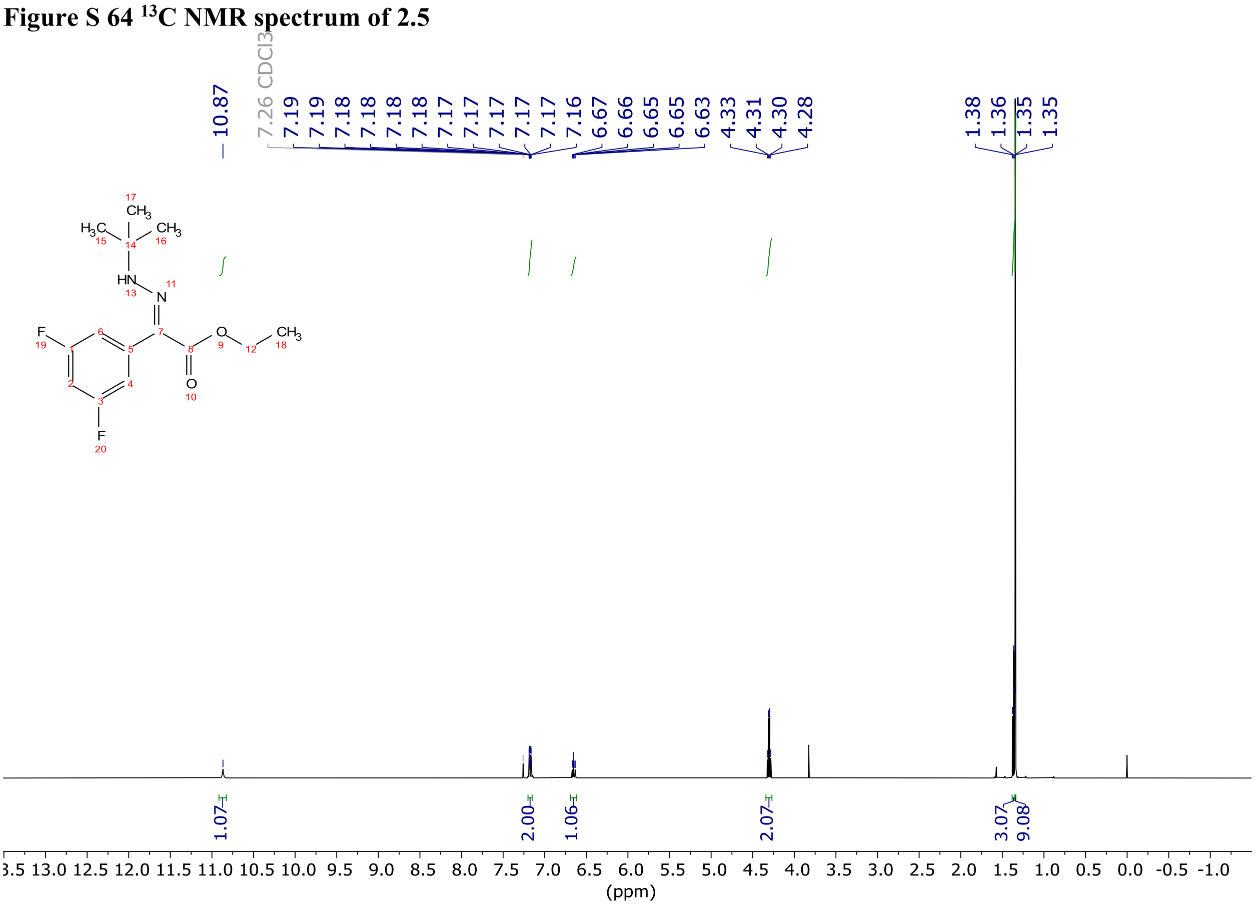

Figure S 65 ^{1}H NMR spectrum of 2.6

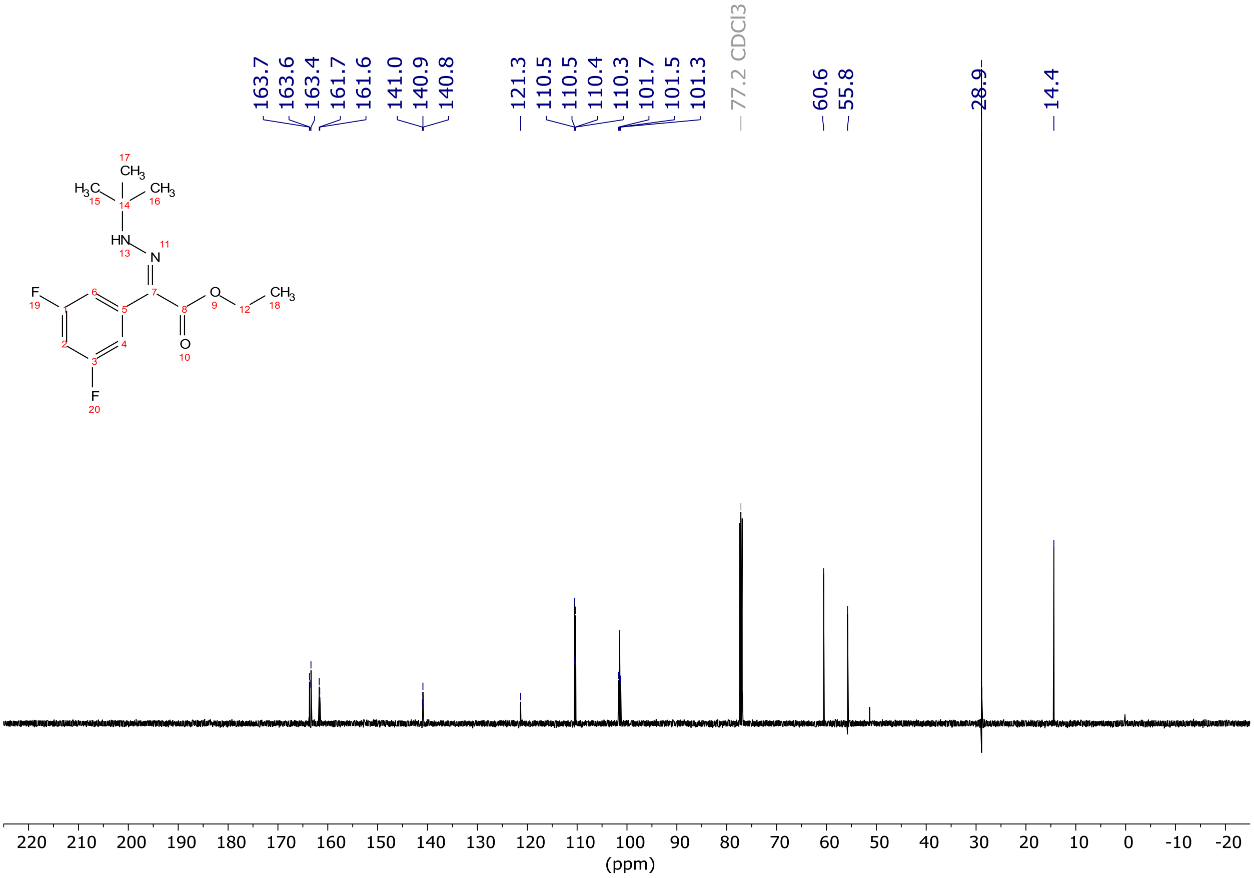

Figure S 66 ^{13}C NMR spectrum of 2.6

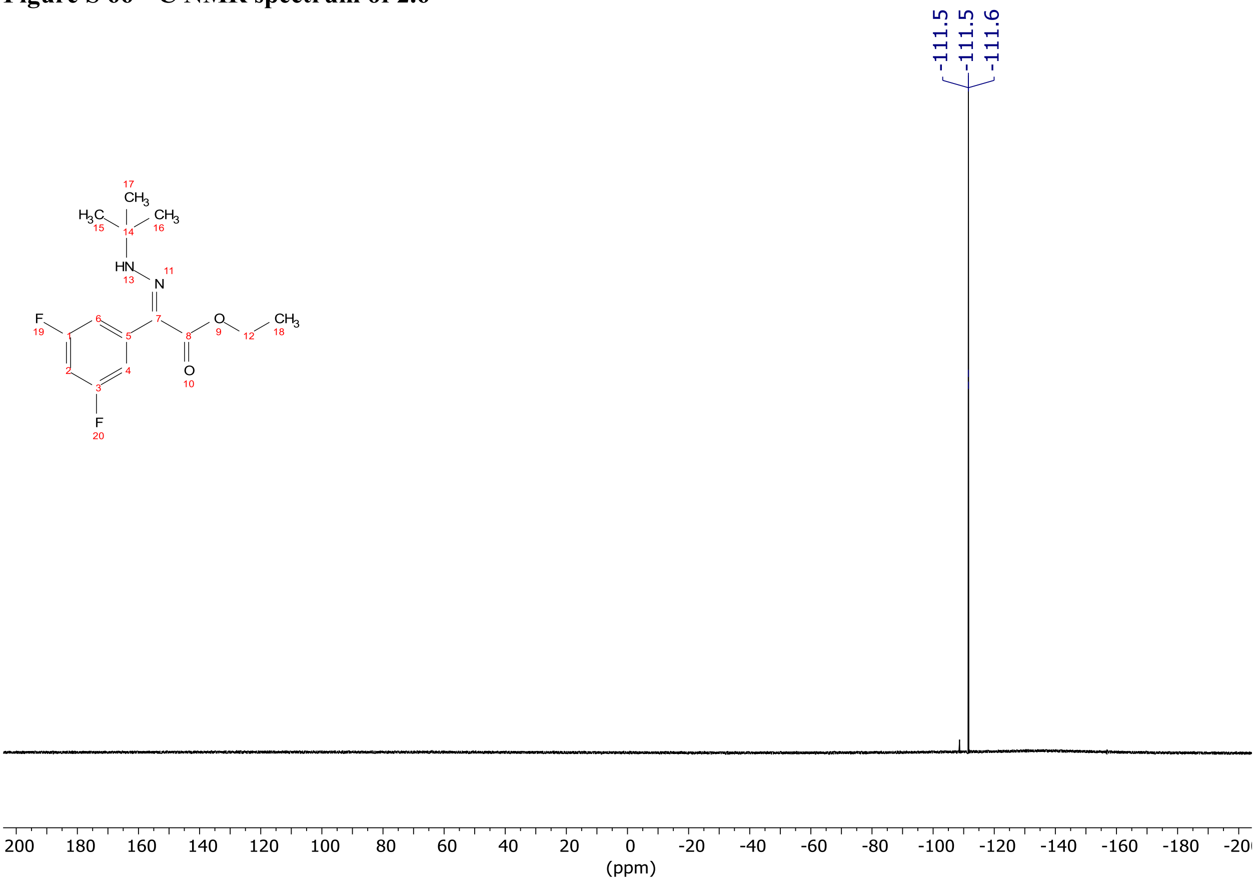

Figure S 67 ^{19}F NMR spectrum of 2.6

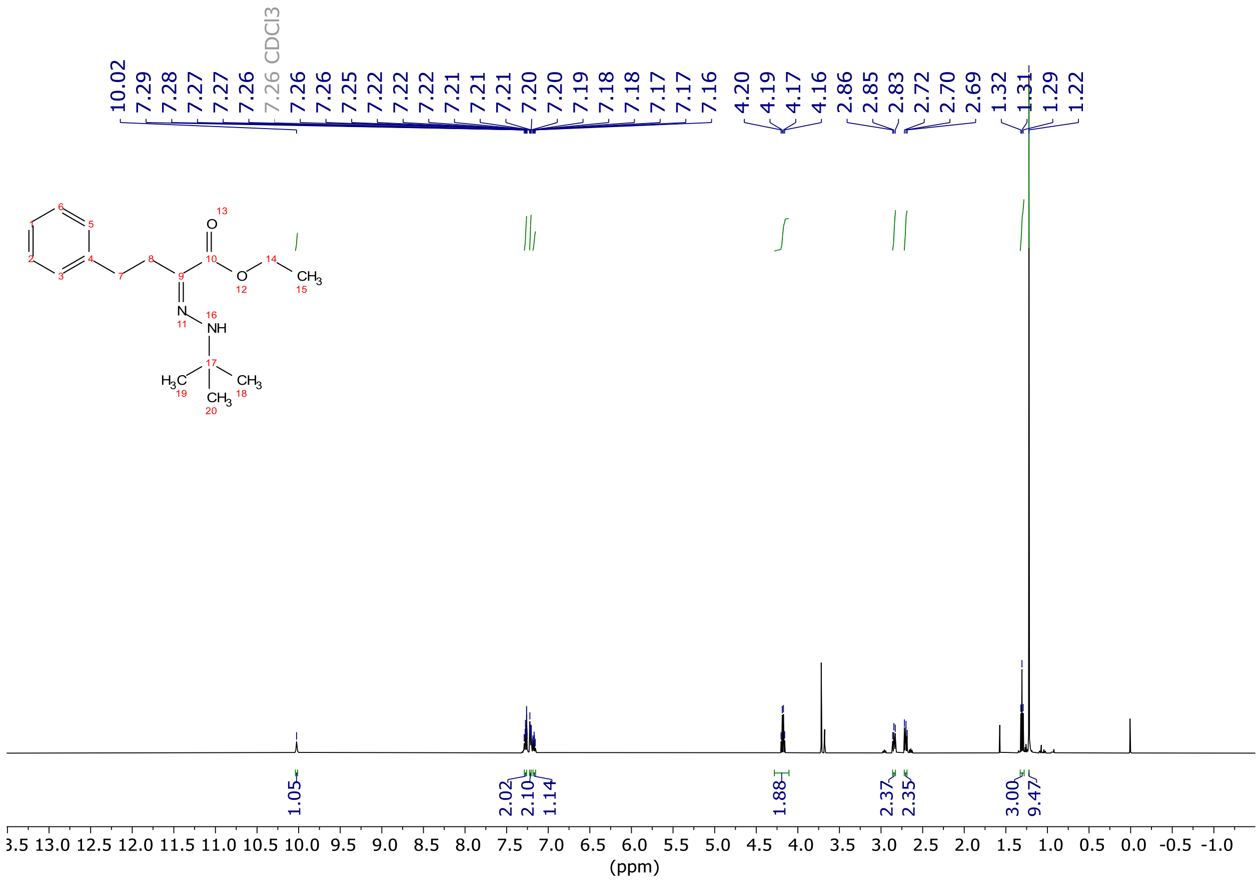

Figure S 68 ^{1}H NMR spectrum of 2.7

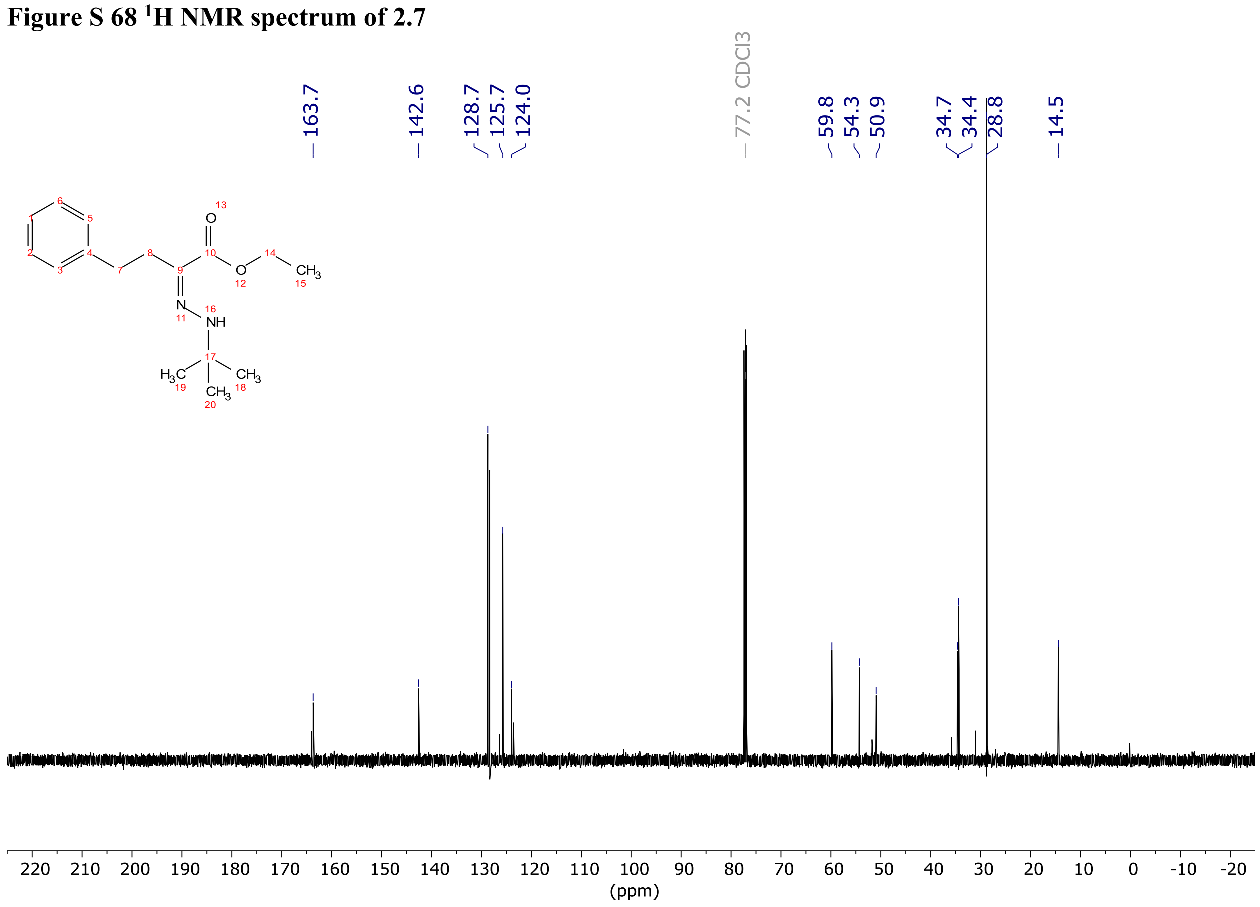

Figure S 69 ^{13}C NMR spectrum of 2.7

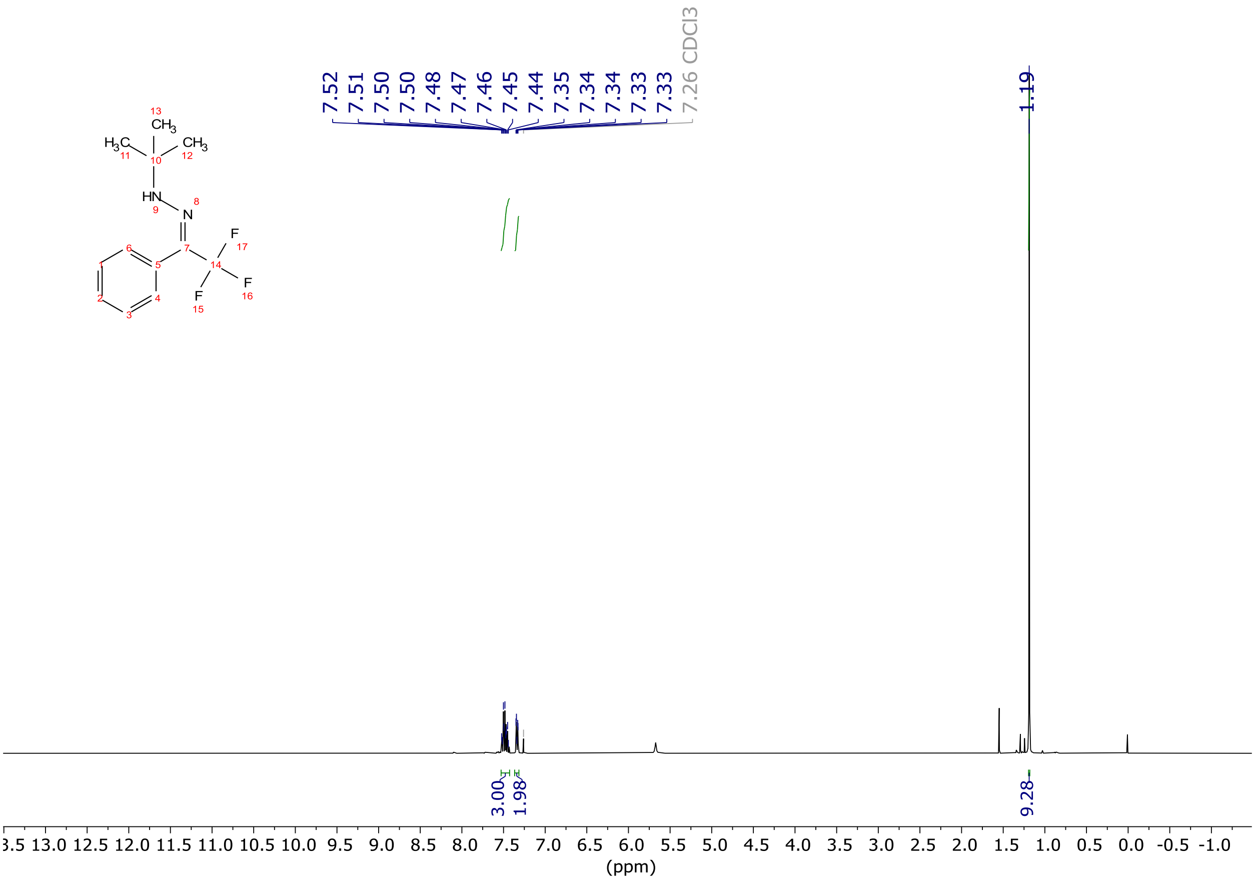

Figure S 70 ^{1}H NMR spectrum of 2.8

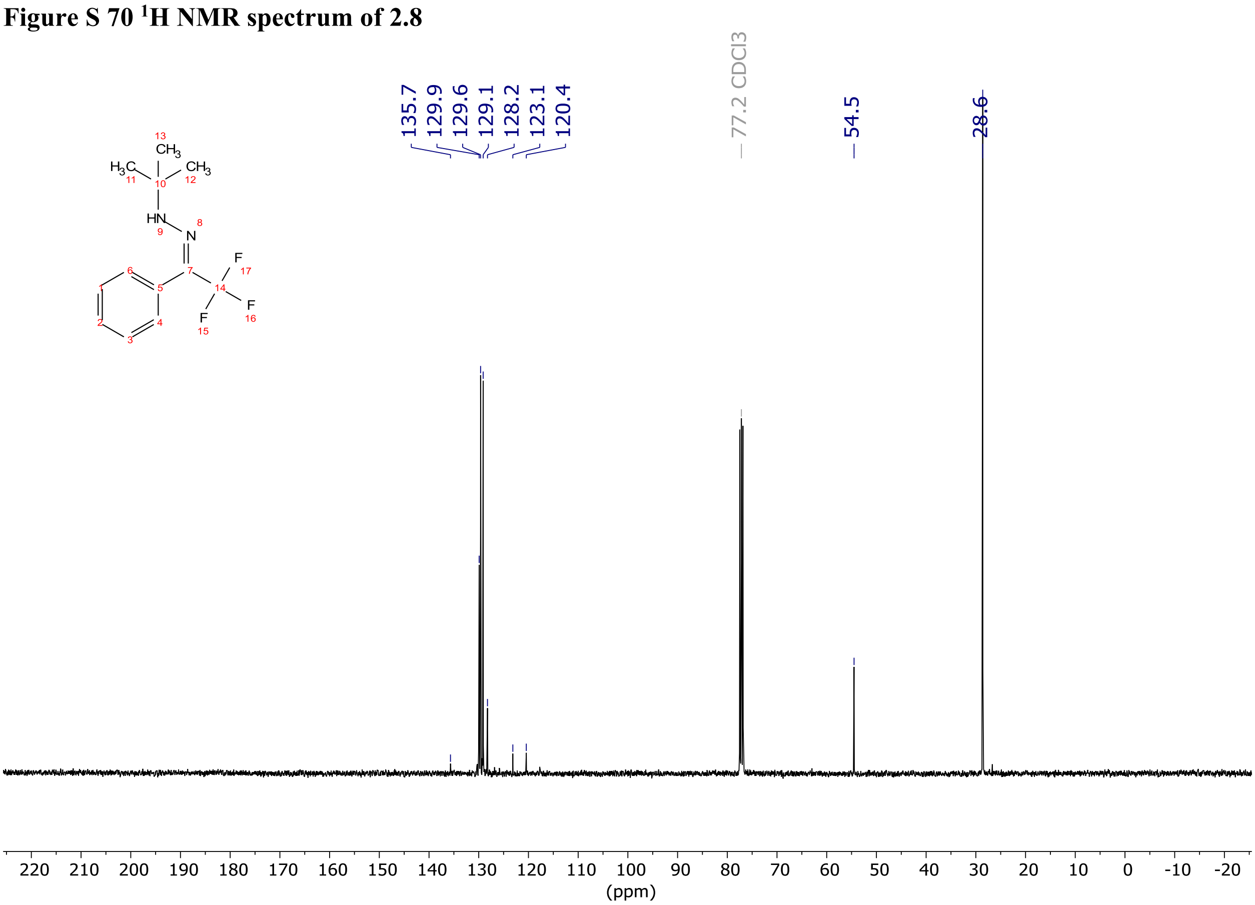

Figure S 71 ^{13}C NMR spectrum of 2.8

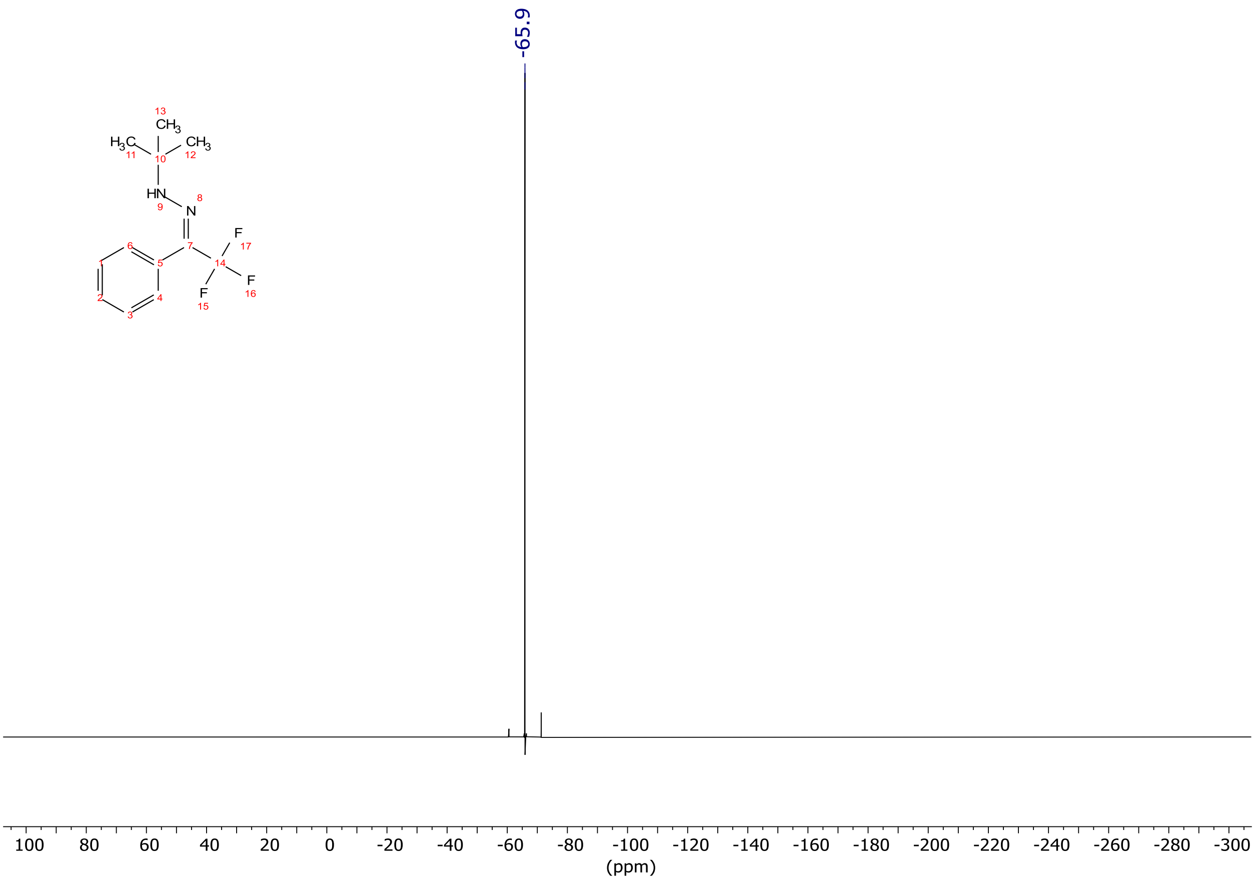

Figure S 72 ^{19}F NMR spectrum of 2.8

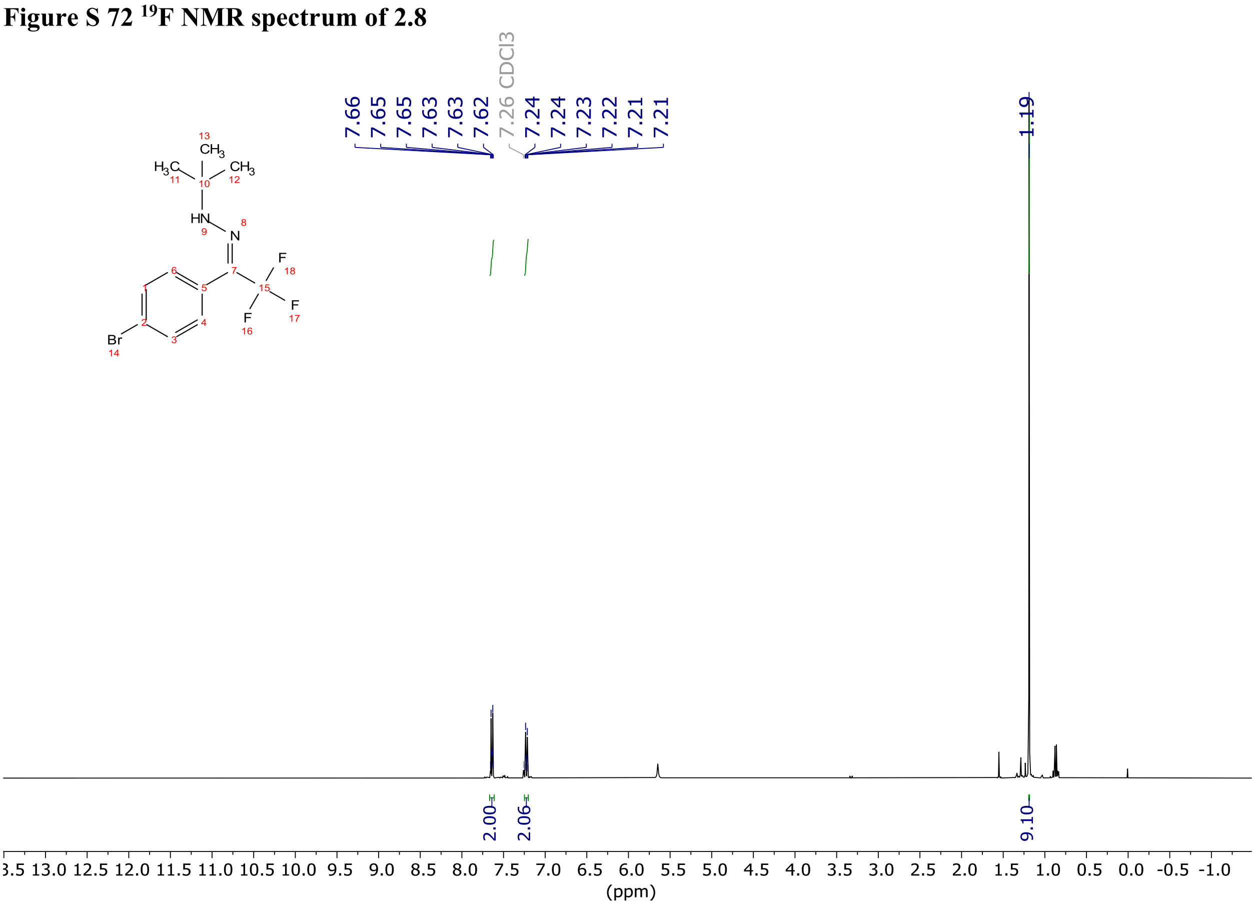

Figure S 73 1H NMR spectrum of 2.9

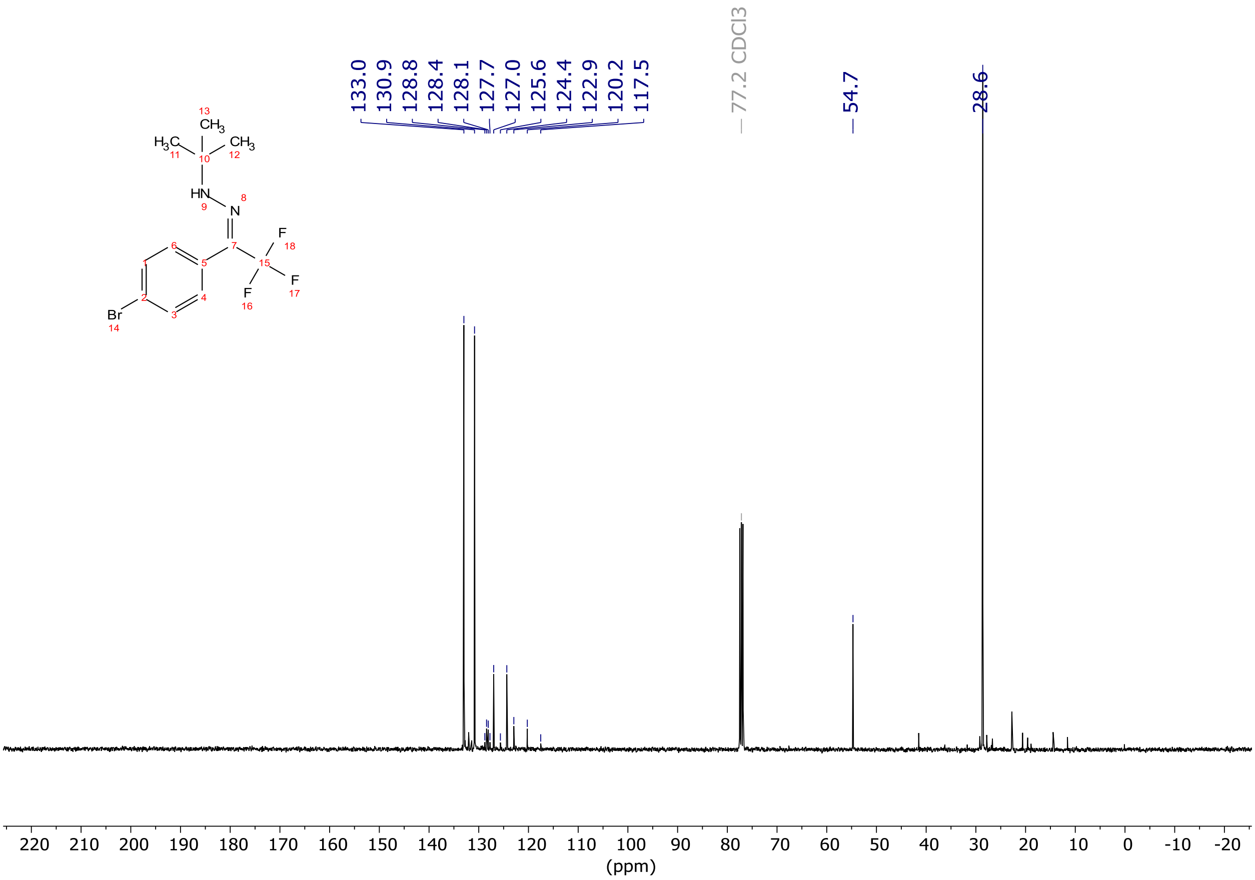

Figure S 74 ^{13}**C NMR spectrum of 2.9**

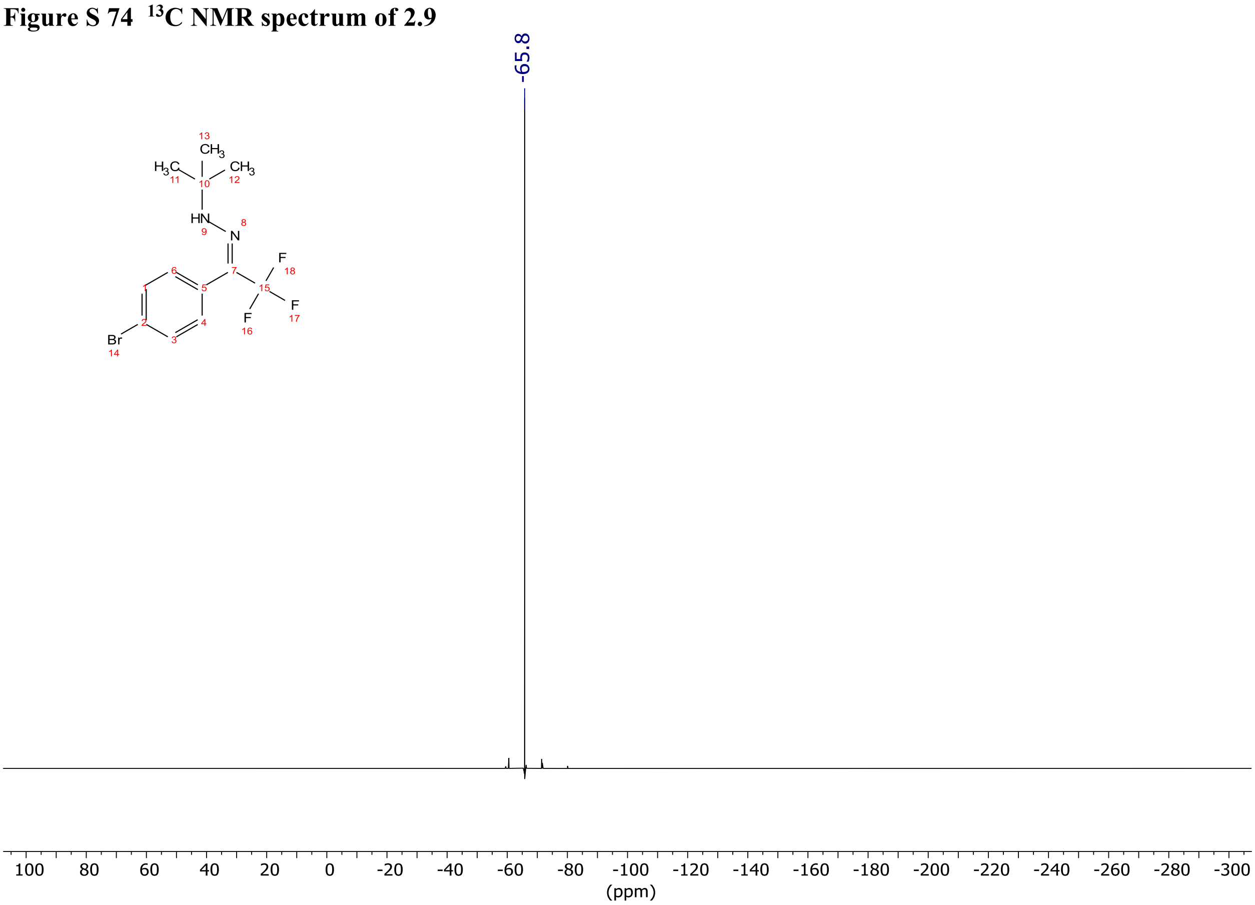

Figure S 75 ^{19}**F NMR spectrum of 2.9**

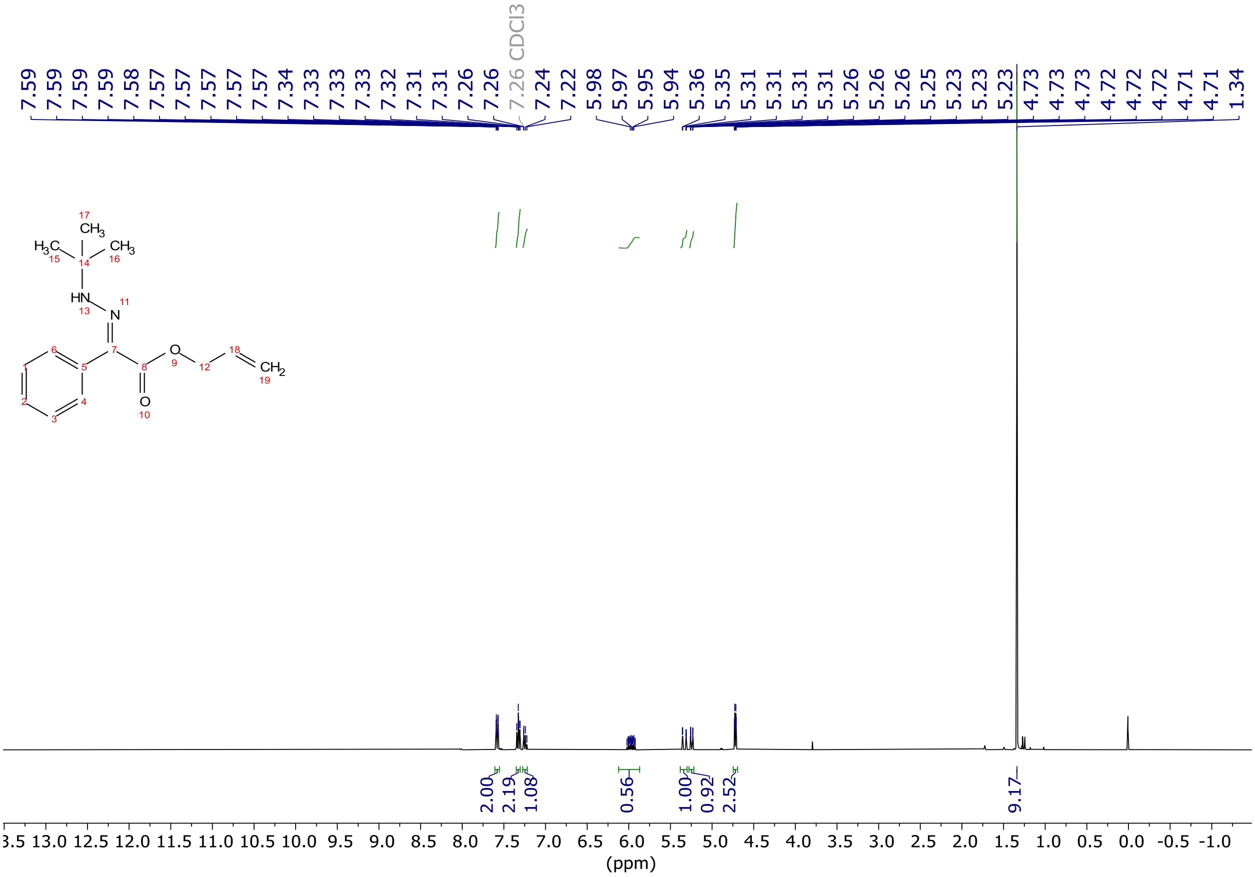

Figure S 76 ^{1}H NMR spectrum of 2.10

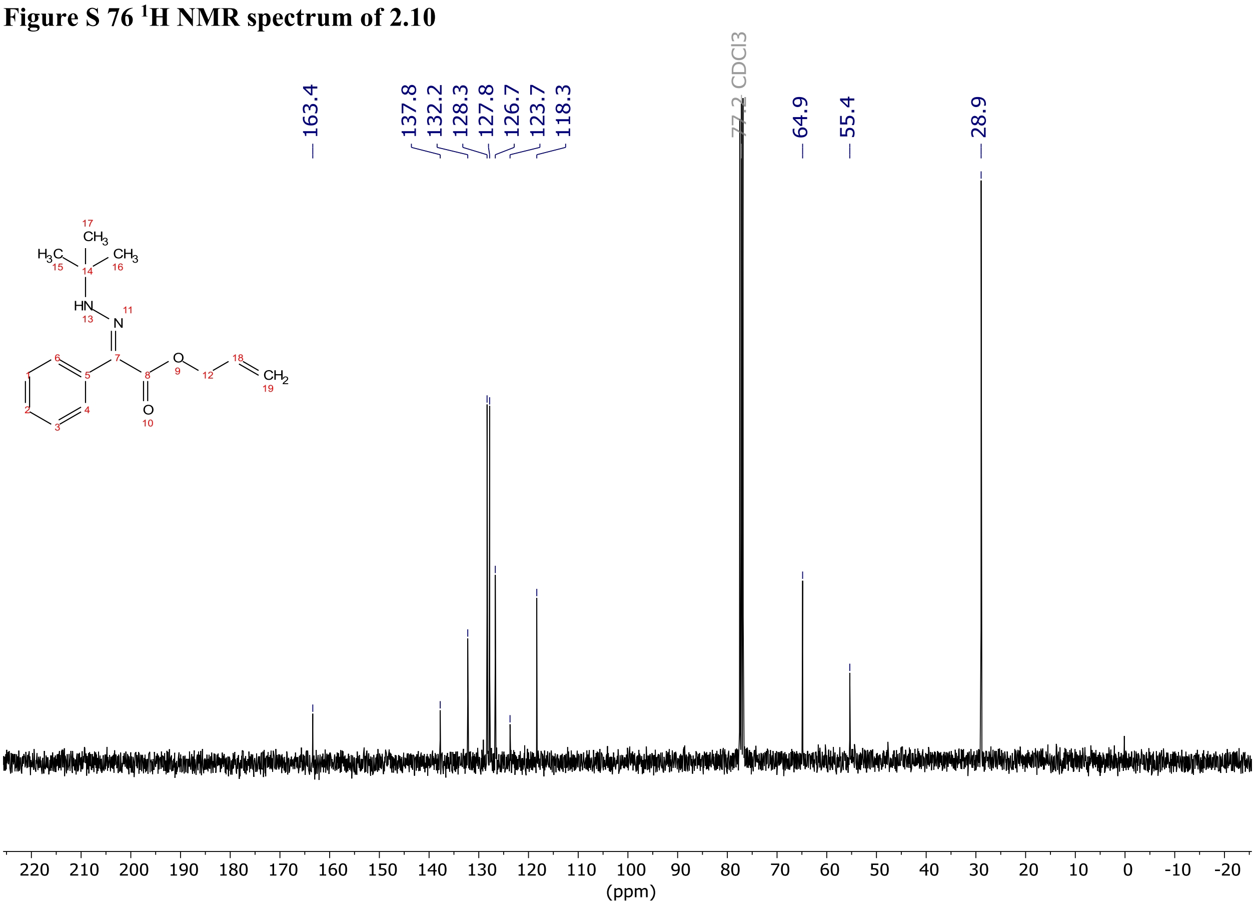

Figure S 77 ^{13}C NMR spectrum of 2.10

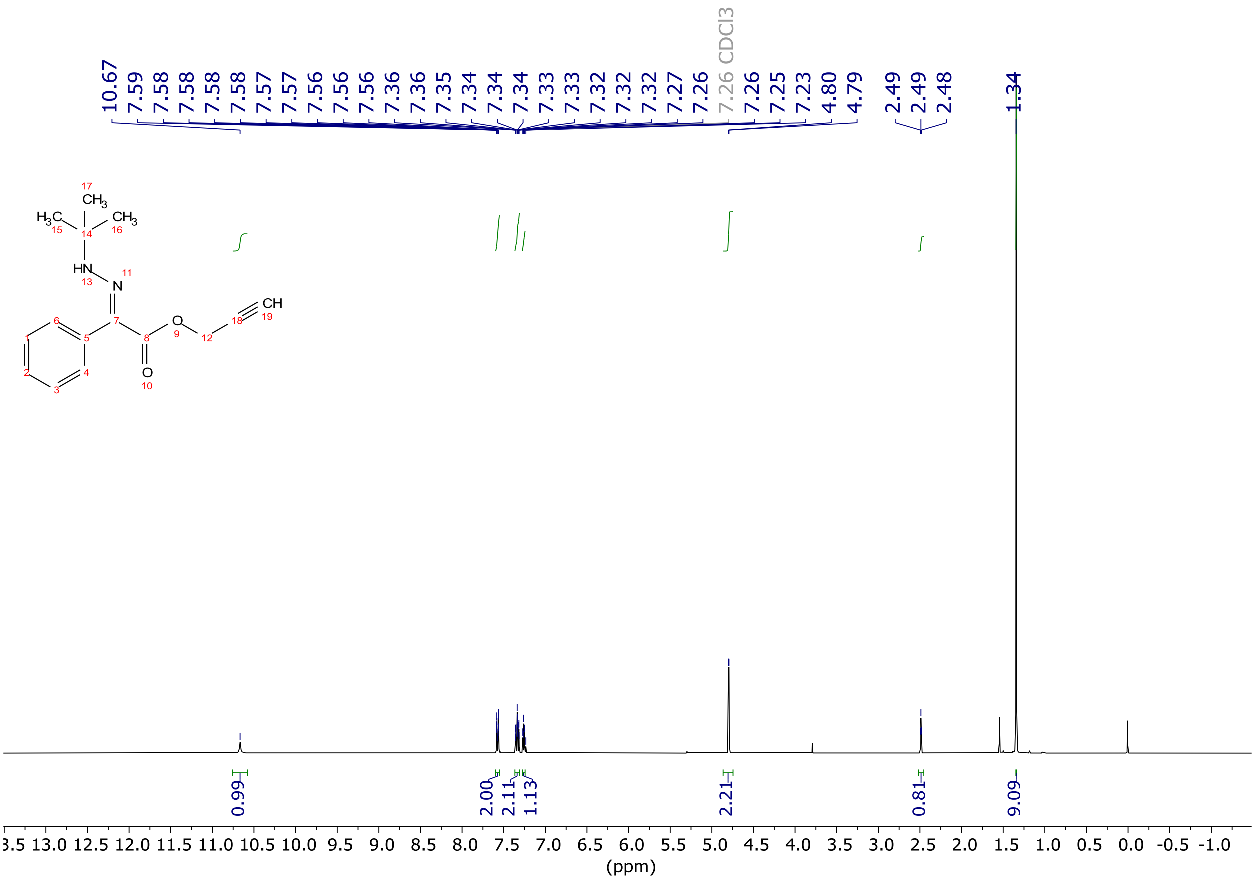

Figure S 78 ^{1}H NMR spectrum of 2.11

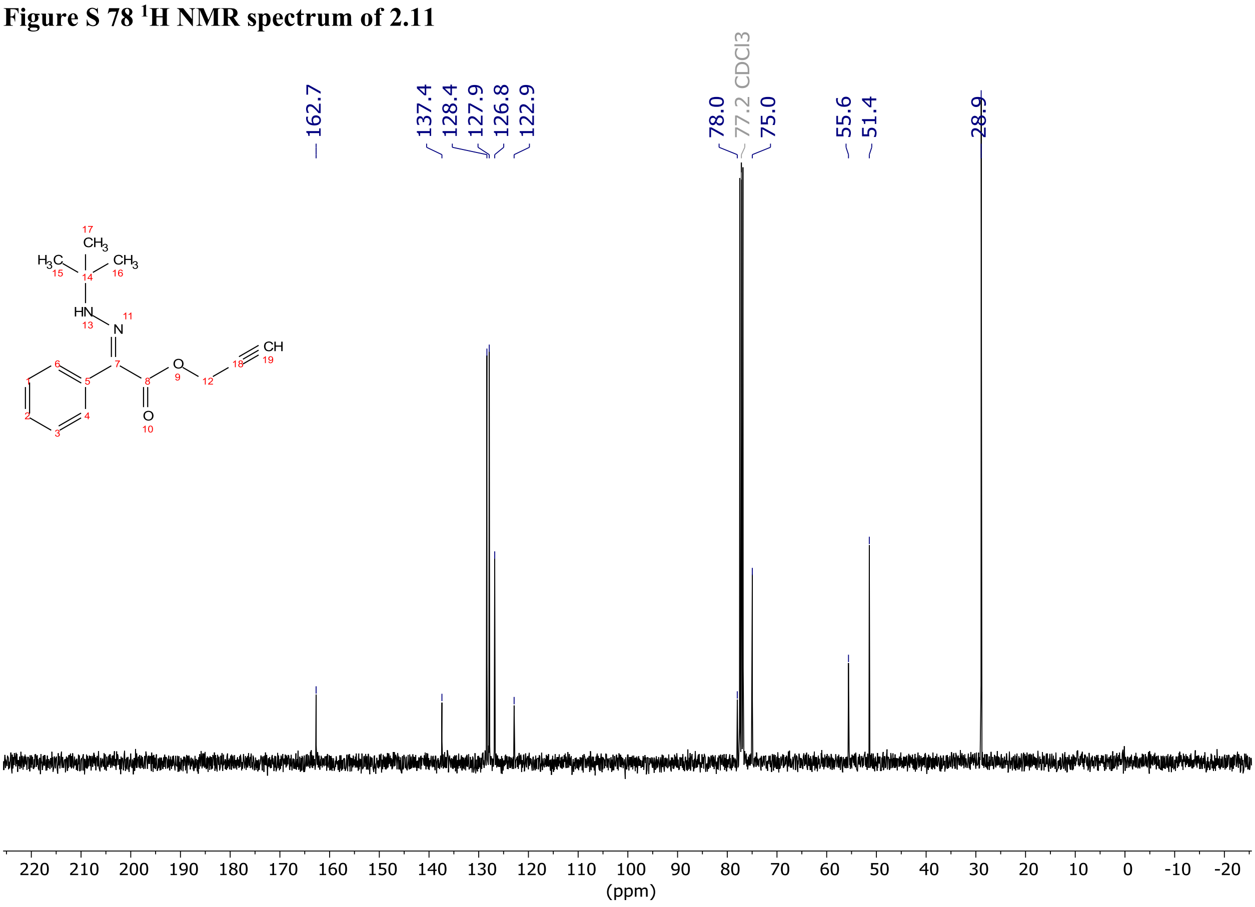

Figure S 79 ^{13}C NMR spectrum of 2.11

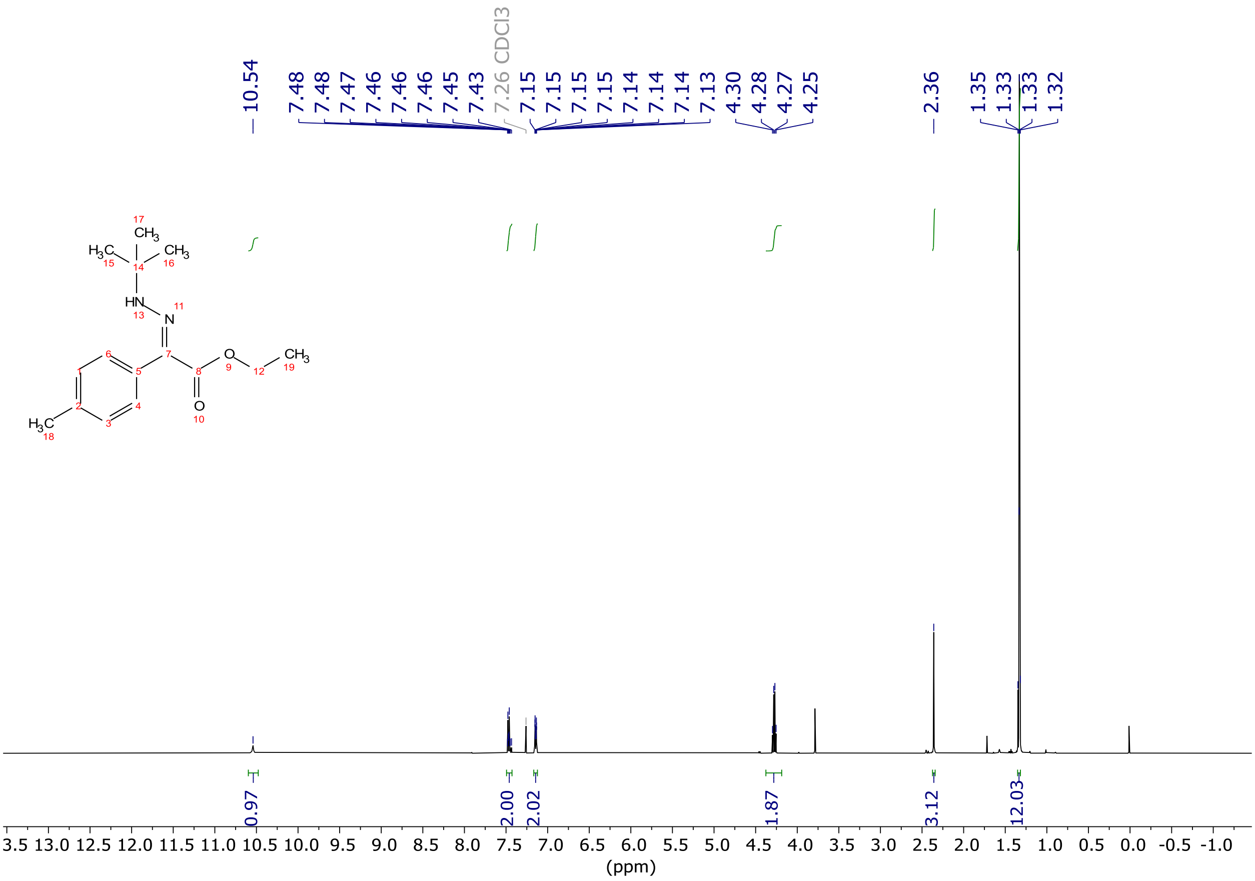

Figure S 80 ^{1}H NMR spectrum of 2.12

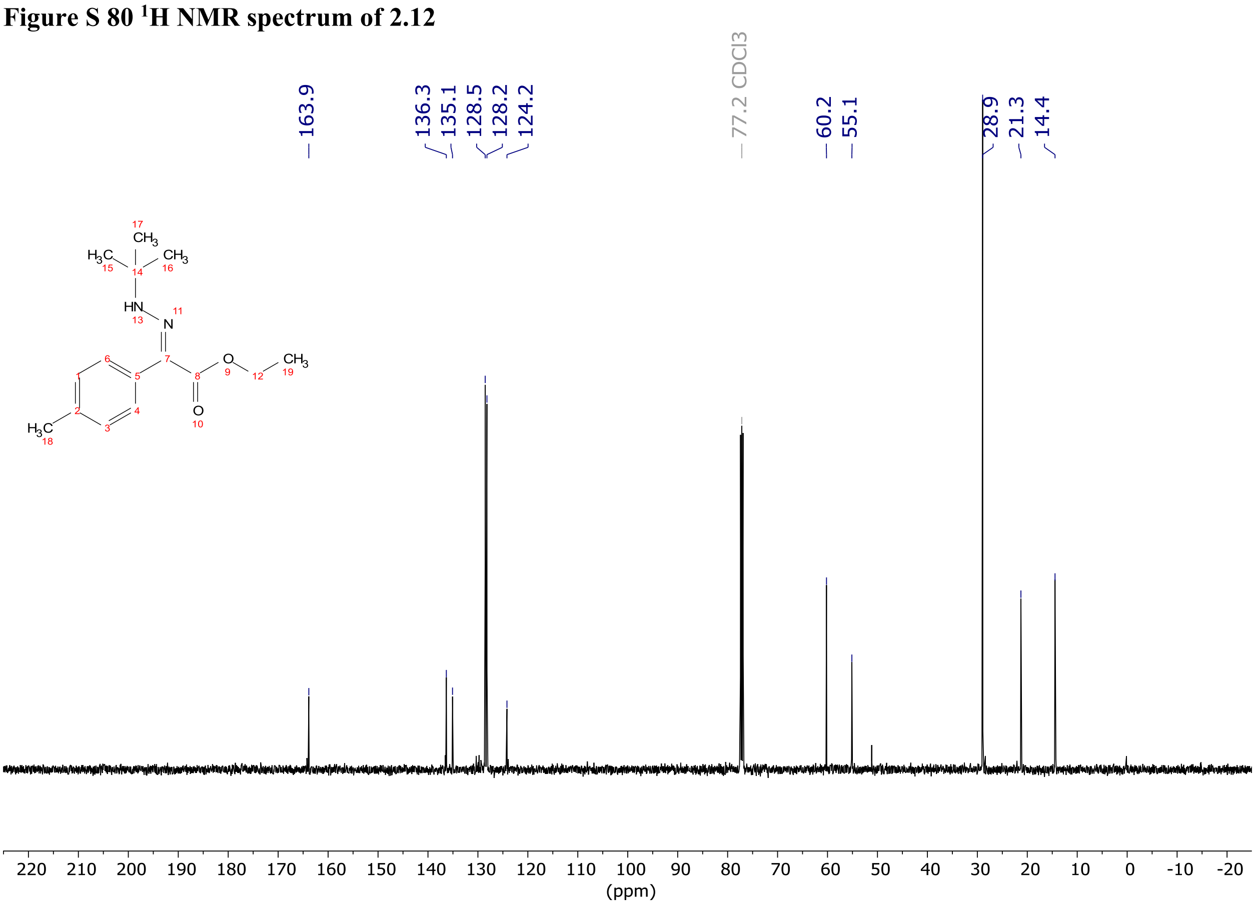

Figure S 81 ^{13}C NMR spectrum of 2.12

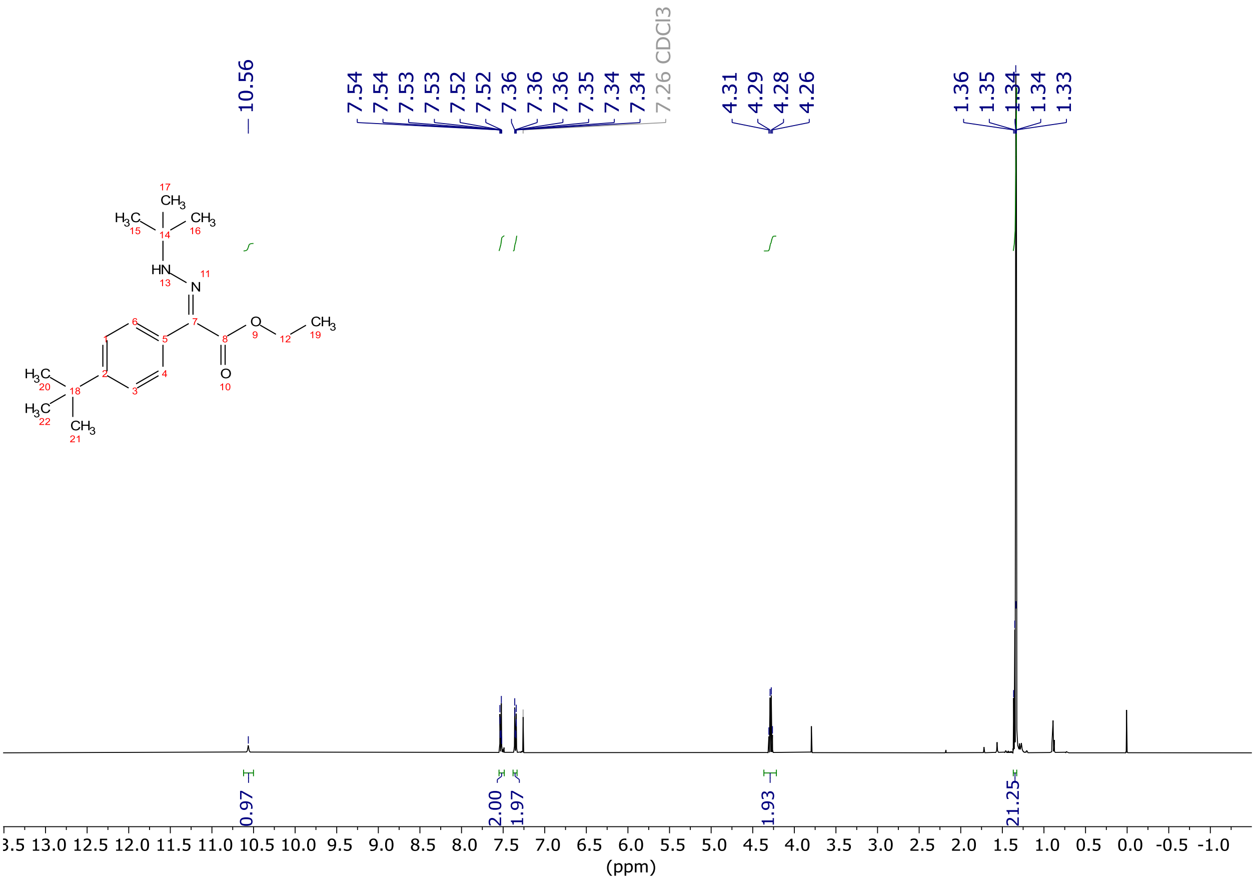

Figure S 82 ^{1}H NMR spectrum of 2.13

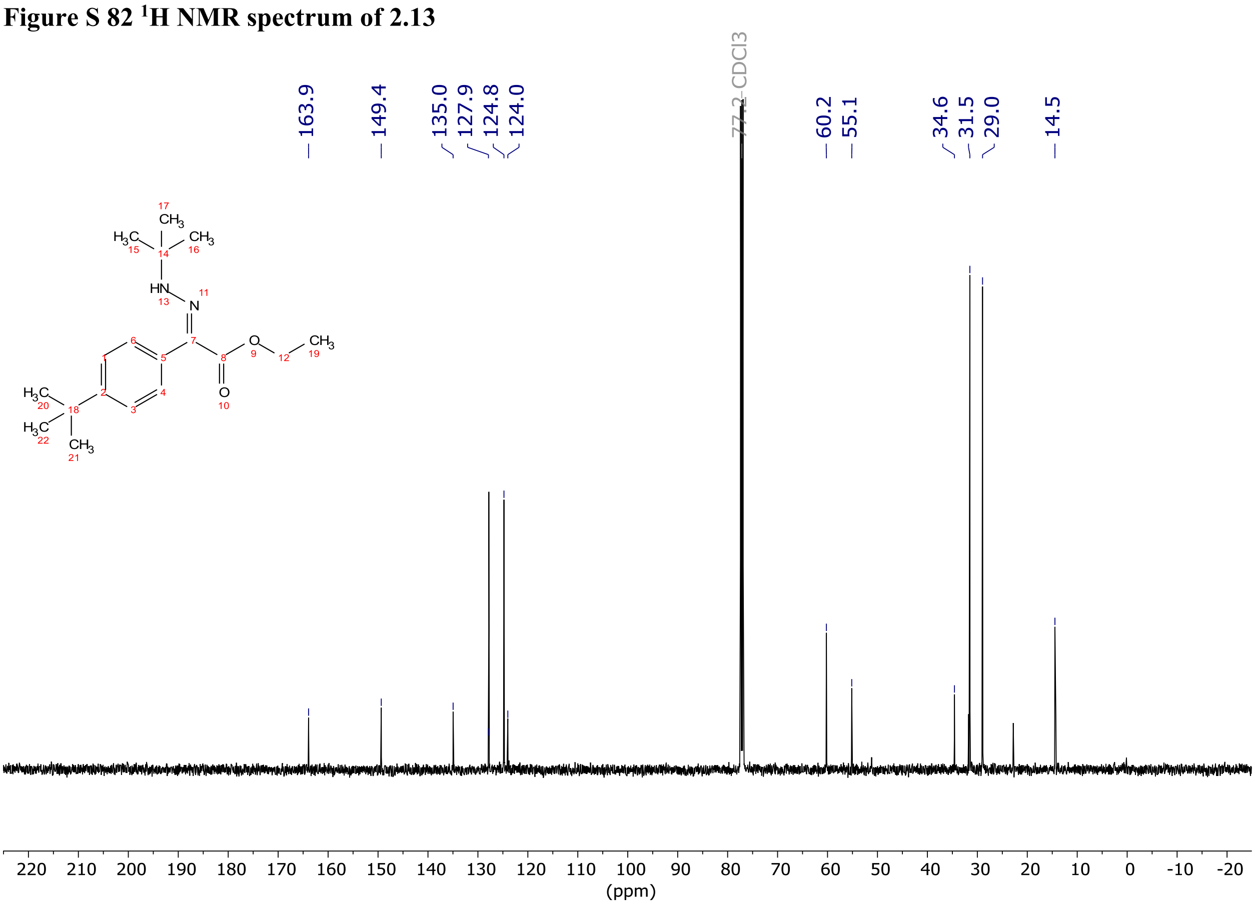

Figure S 83 ^{13}C NMR spectrum of 2.13

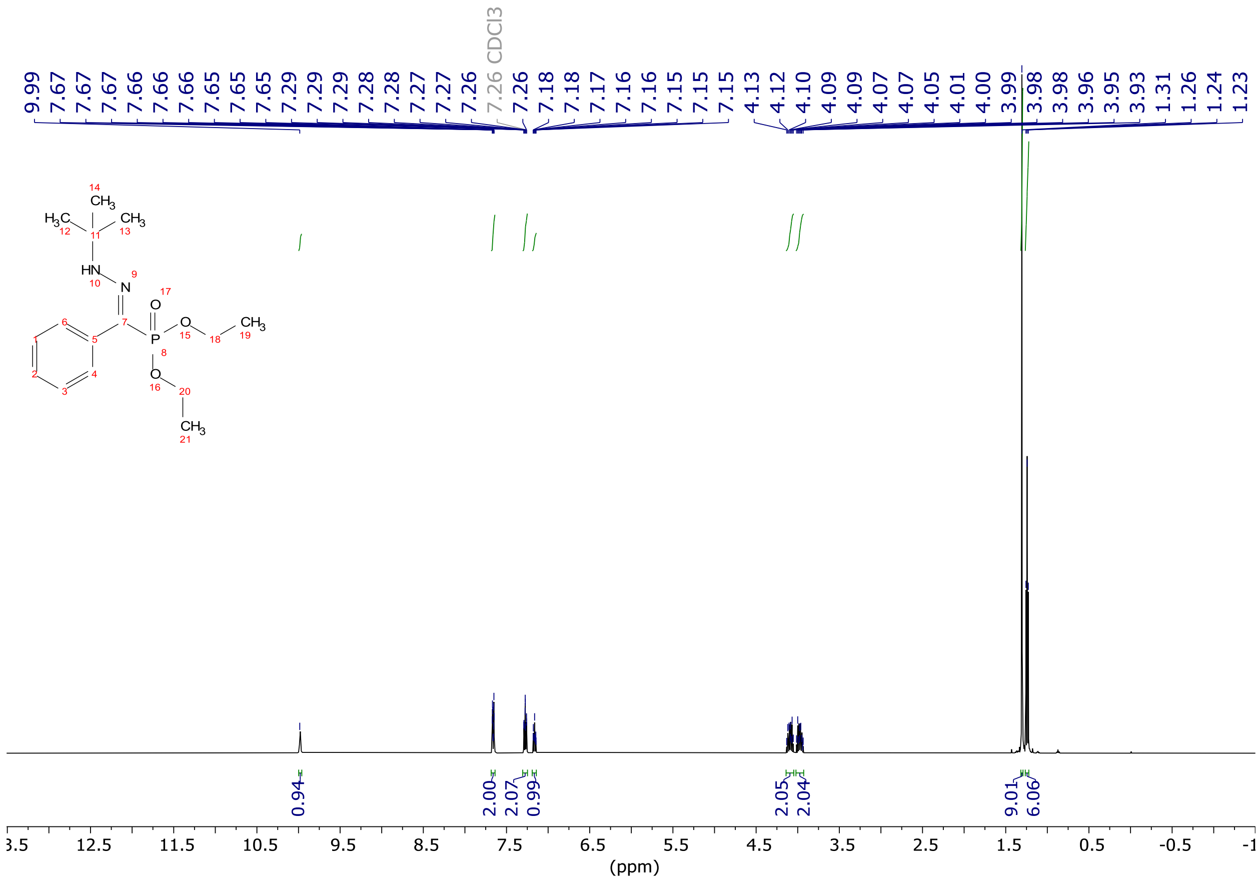

Figure S 84 ^{1}H NMR spectrum of 2.14

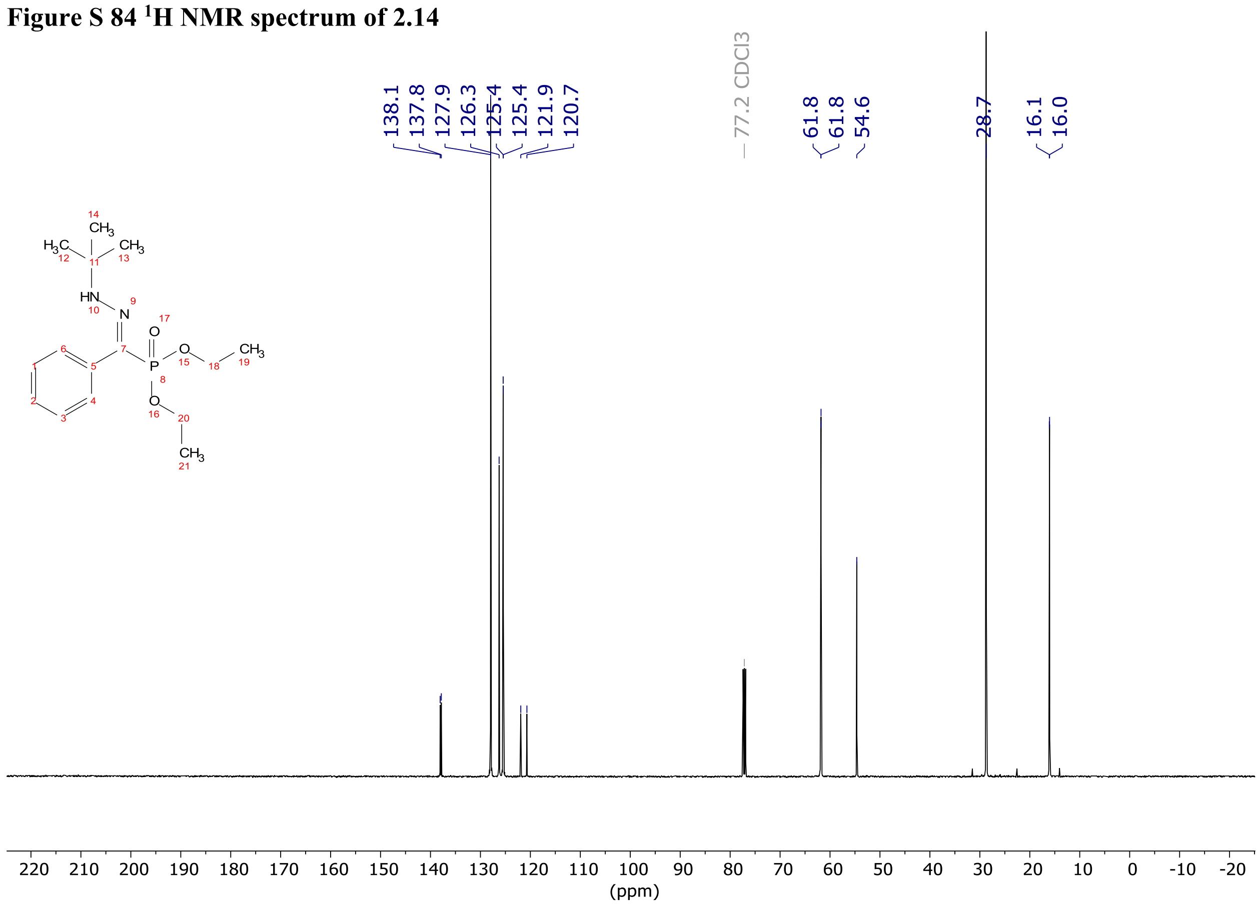

Figure S 85 ^{13}C NMR spectrum of 2.14

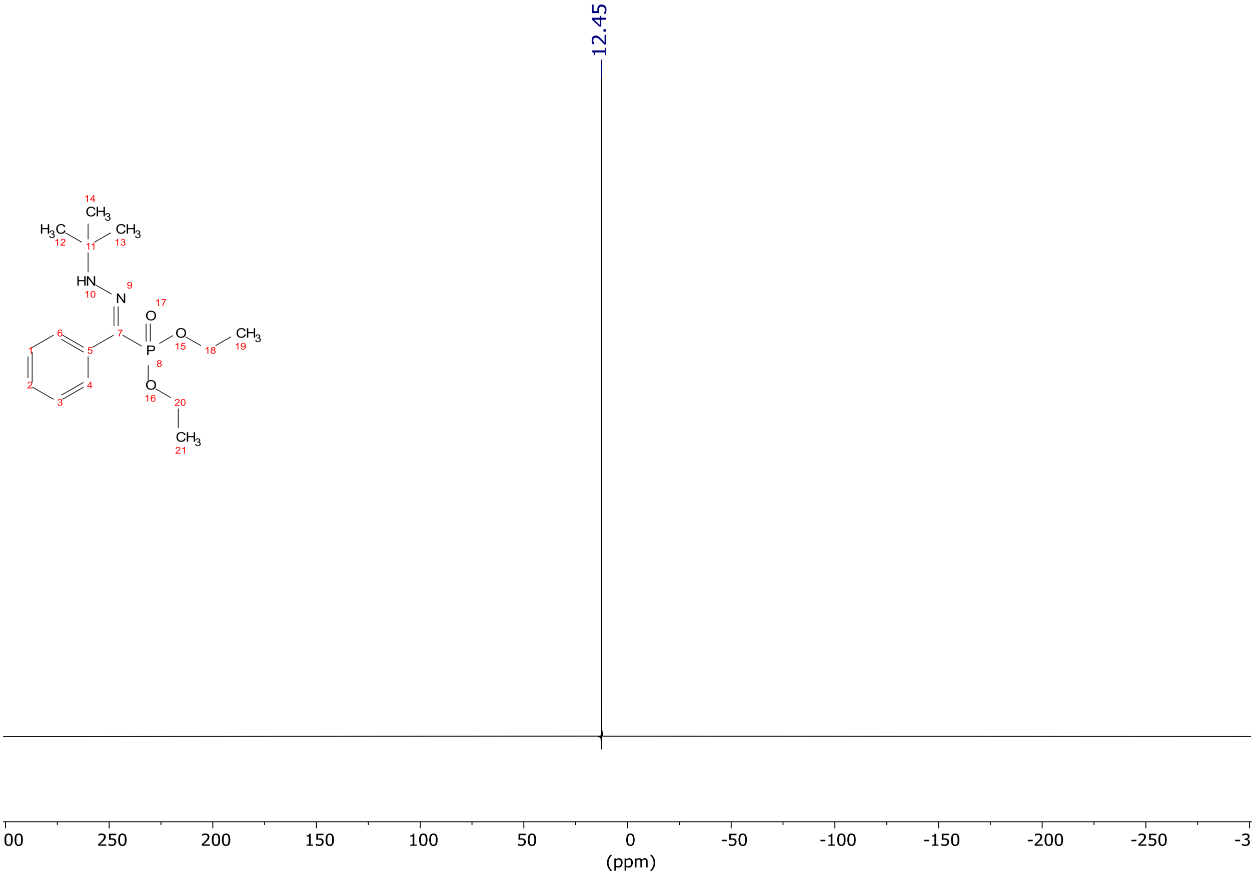

Figure S 86 ^{31}P NMR spectrum of 2.14

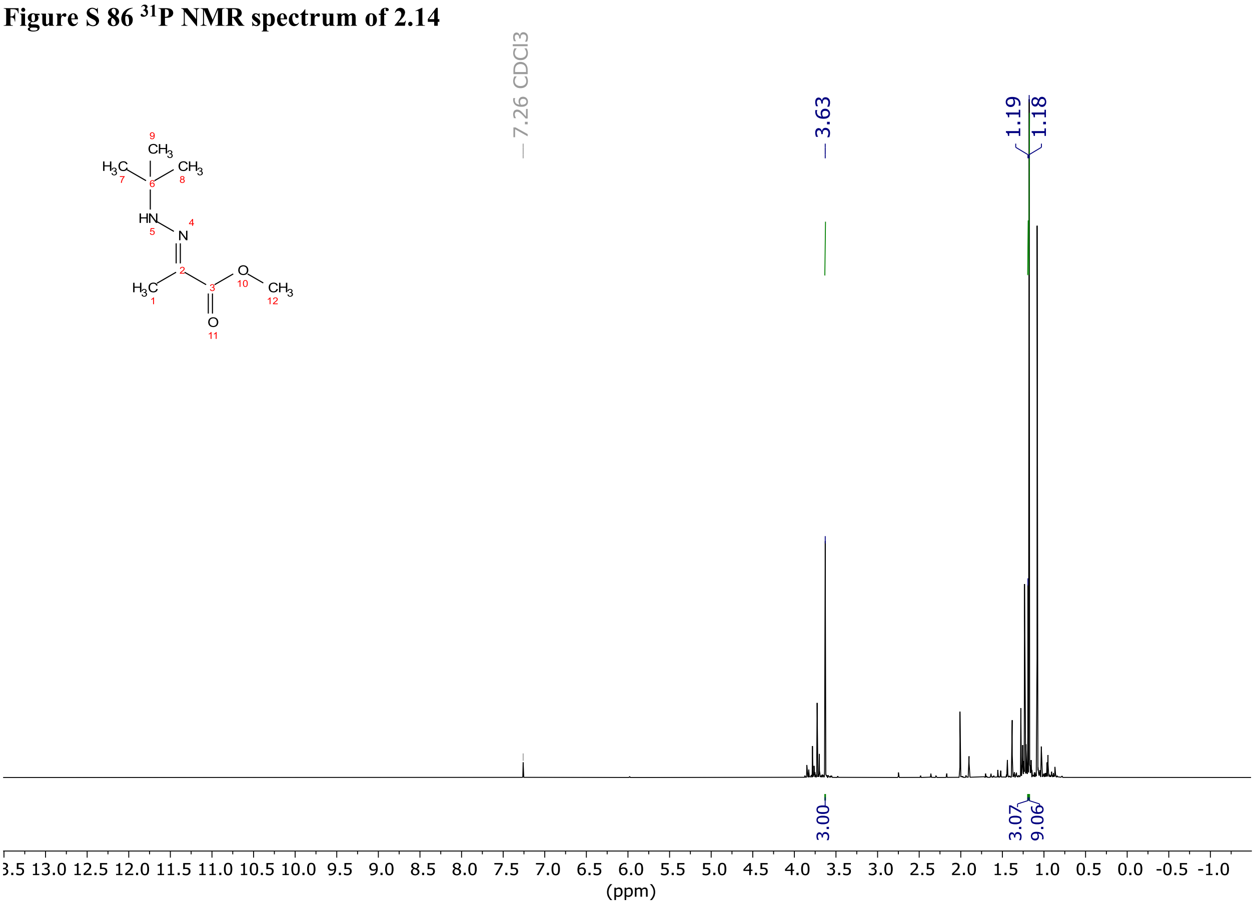

Figure S 87 ^{1}H NMR spectrum of 2.15

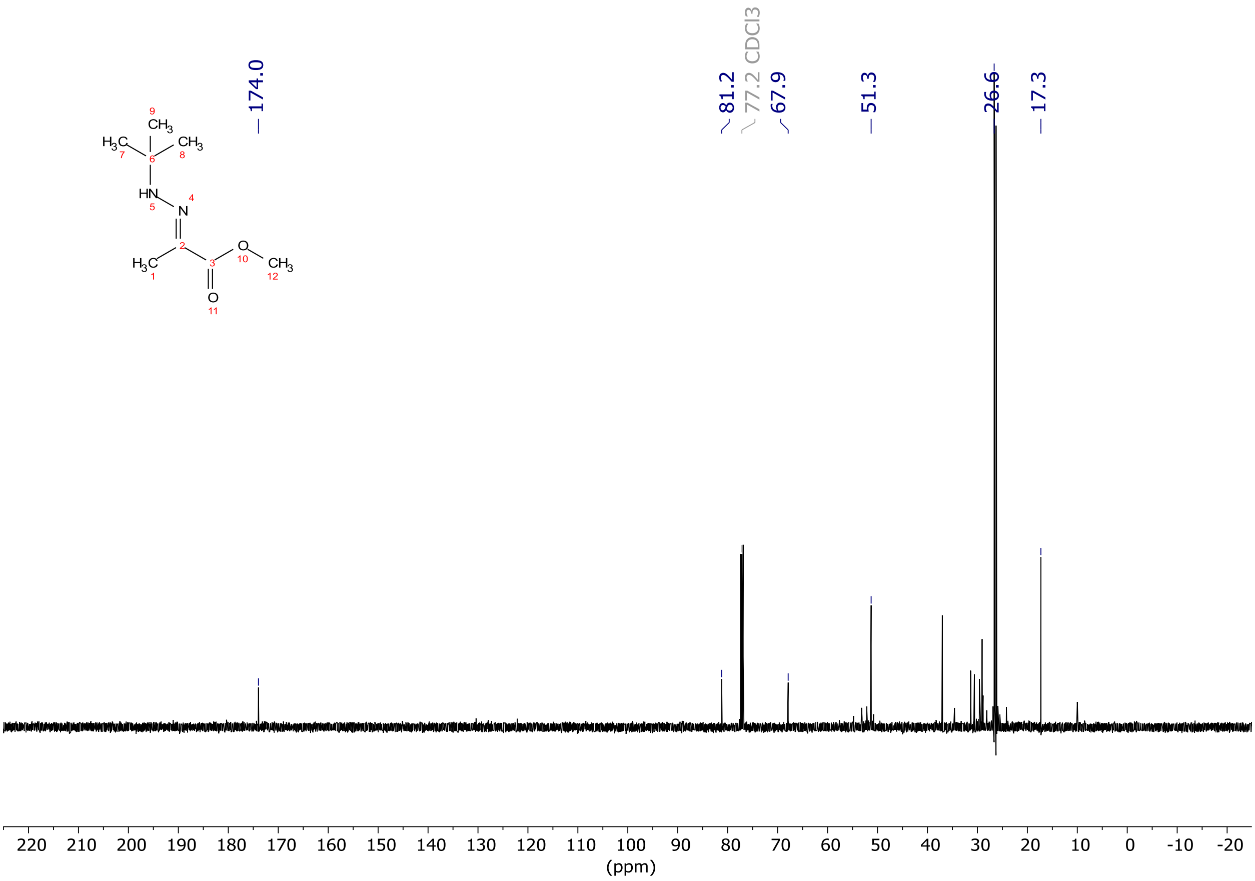

Figure S 88 ^{13}C NMR spectrum of 2.15

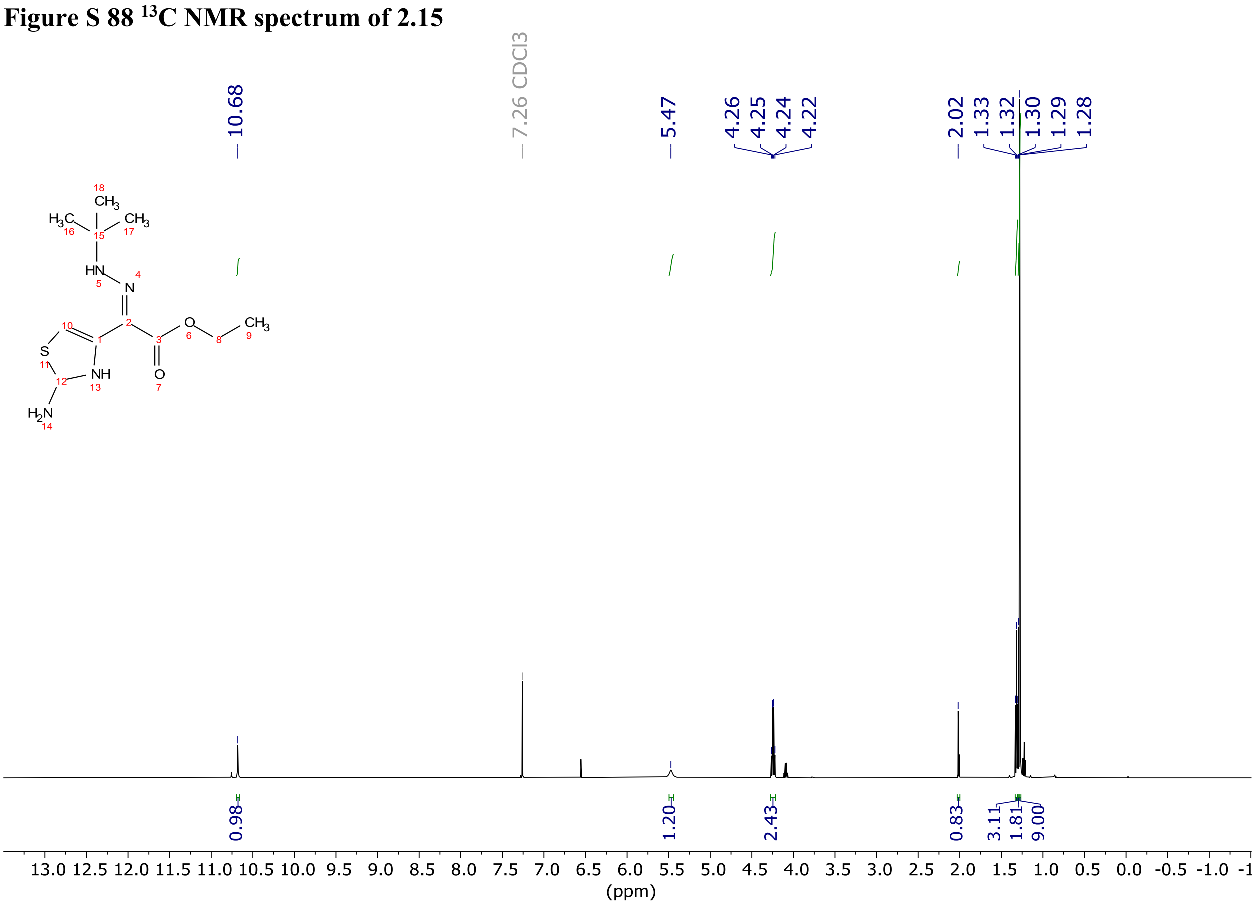

Figure S 89 ^{1}H NMR spectrum of 2.16

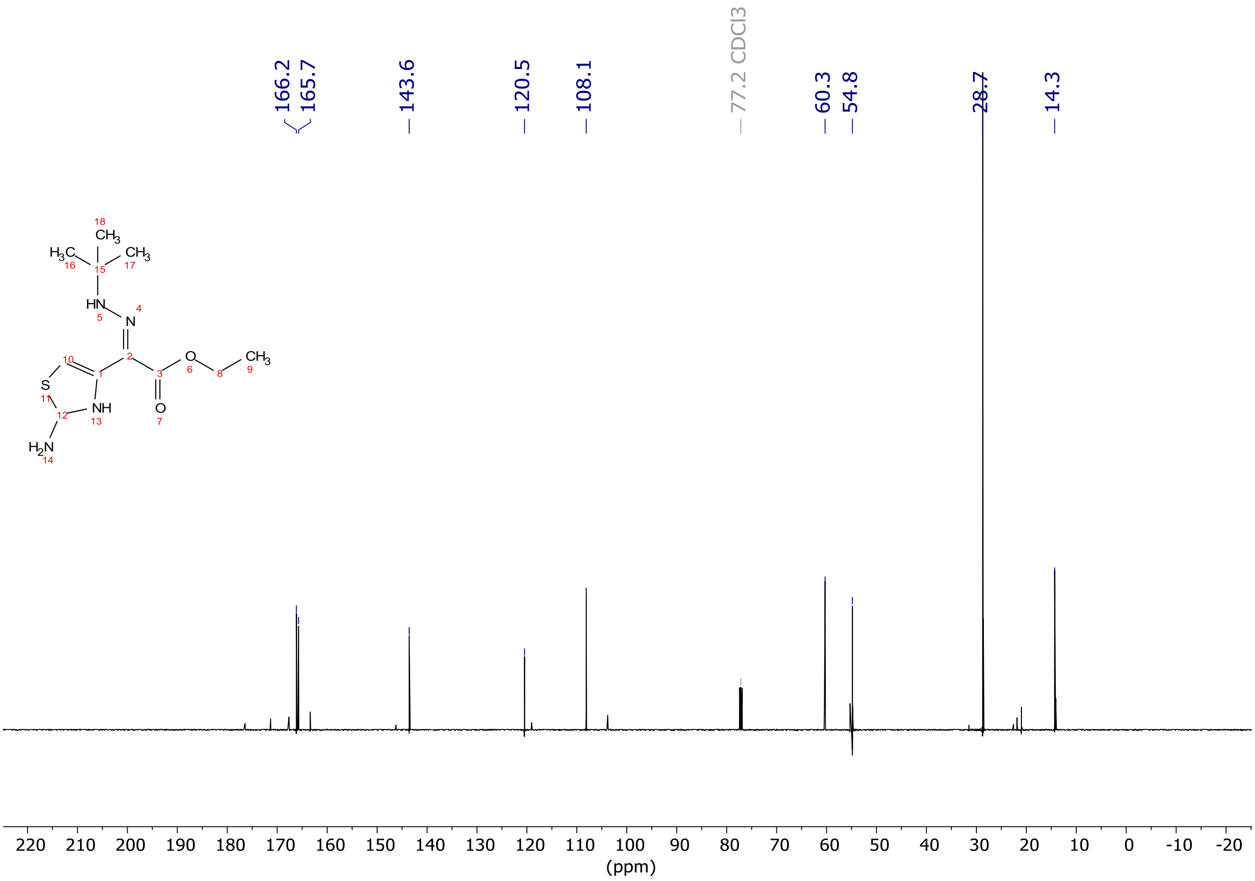

Figure S 90 ^{13}C NMR spectrum of 2.16

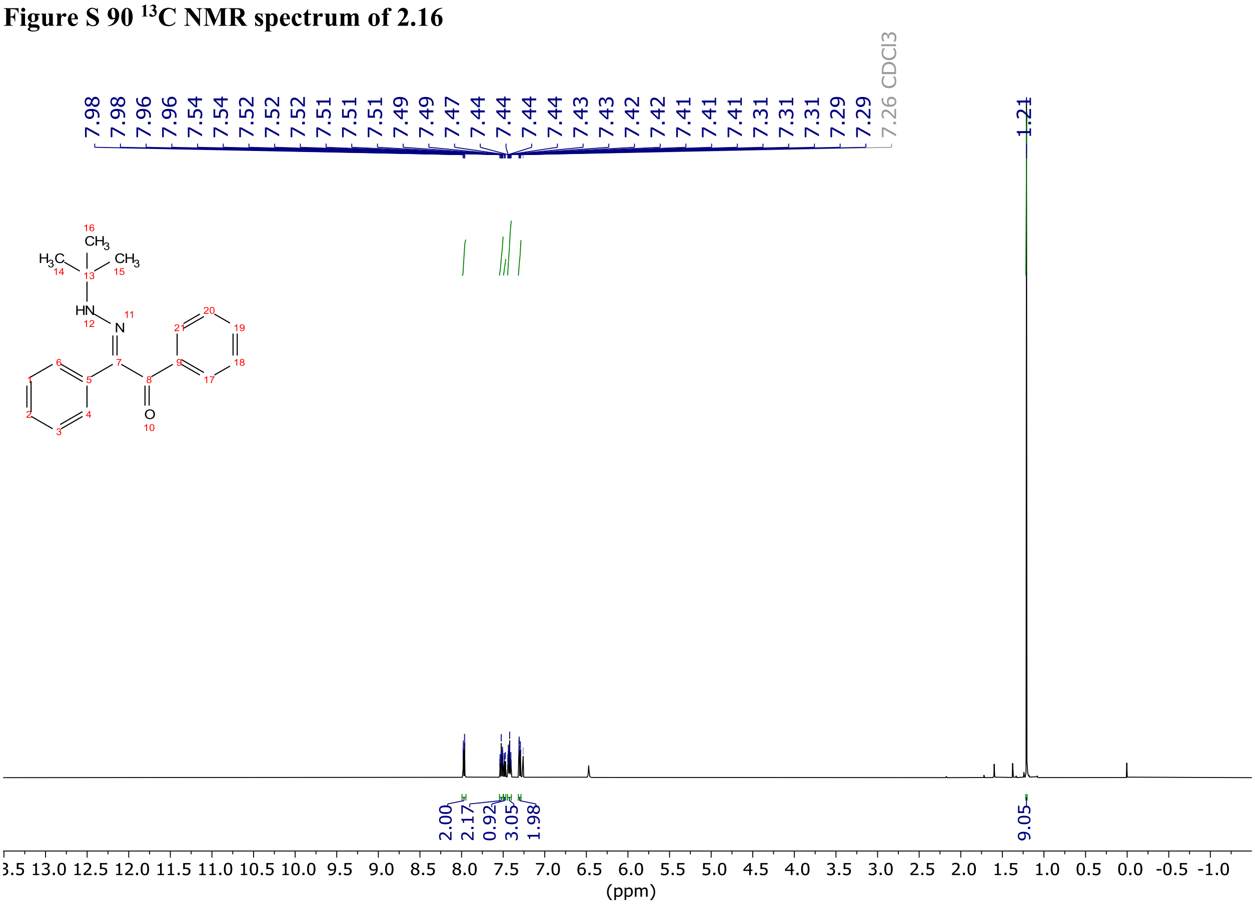

Figure S 91 ^{1}H NMR spectrum of 2.17

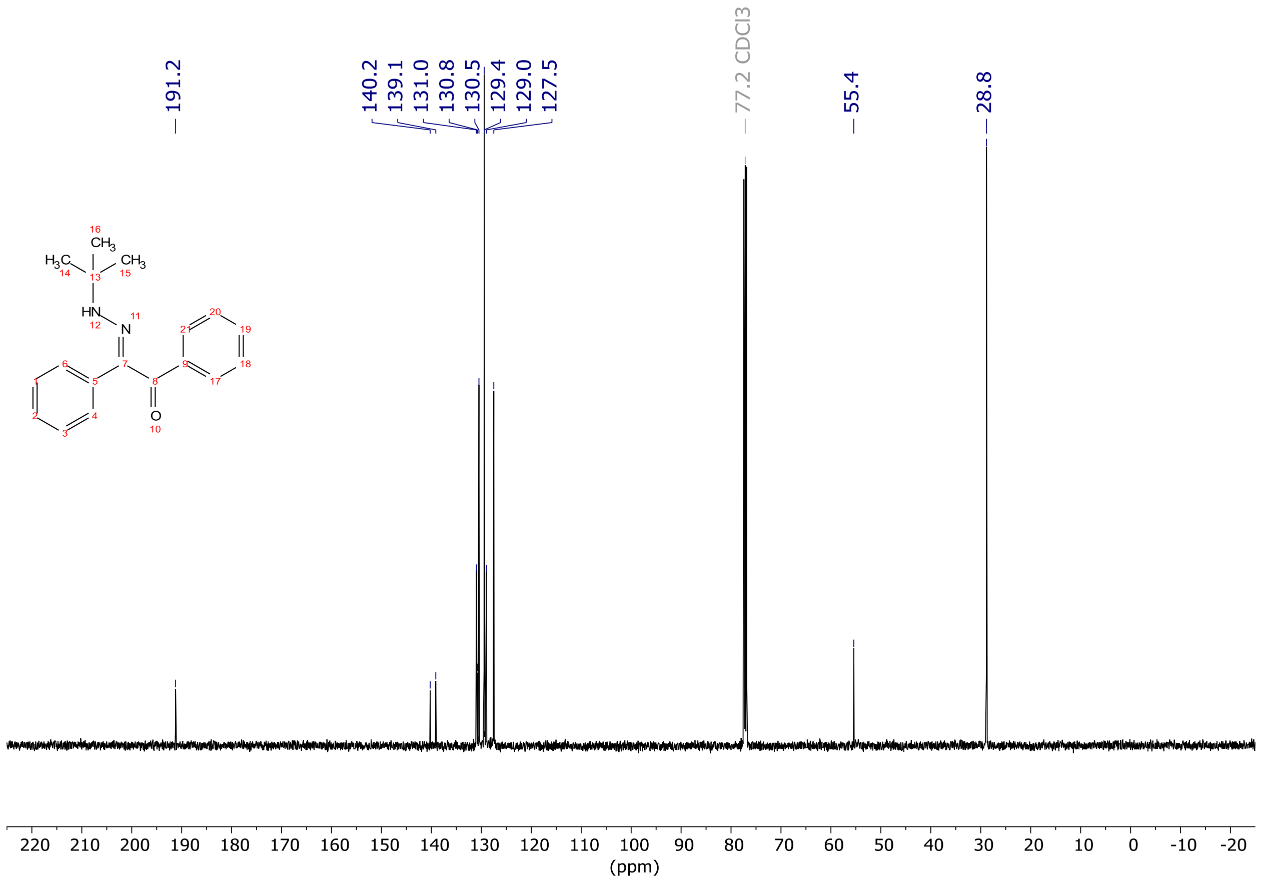

Figure S 92 ^{13}C NMR spectrum of 2.17

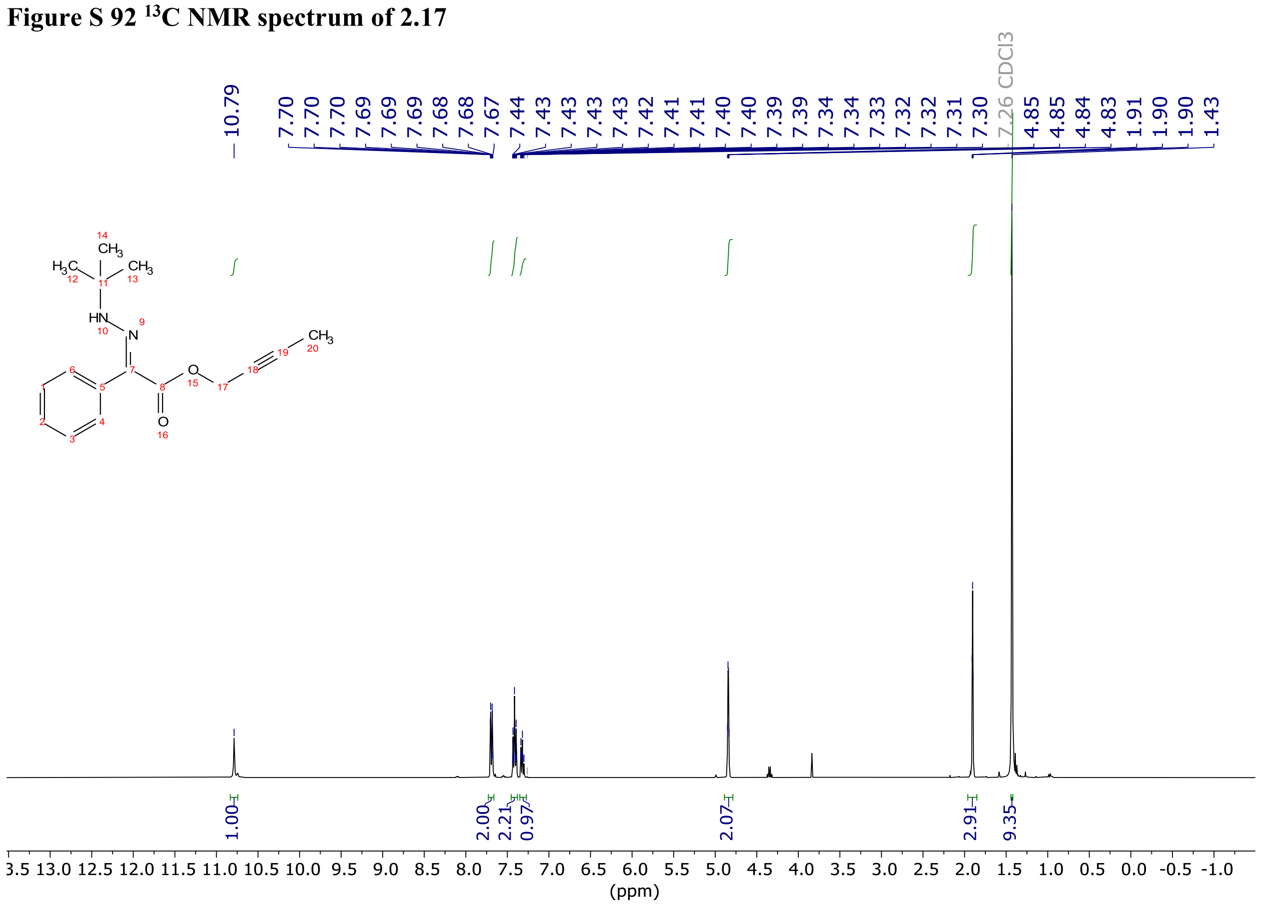

Figure S 93 ^{1}H NMR spectrum of 2.18

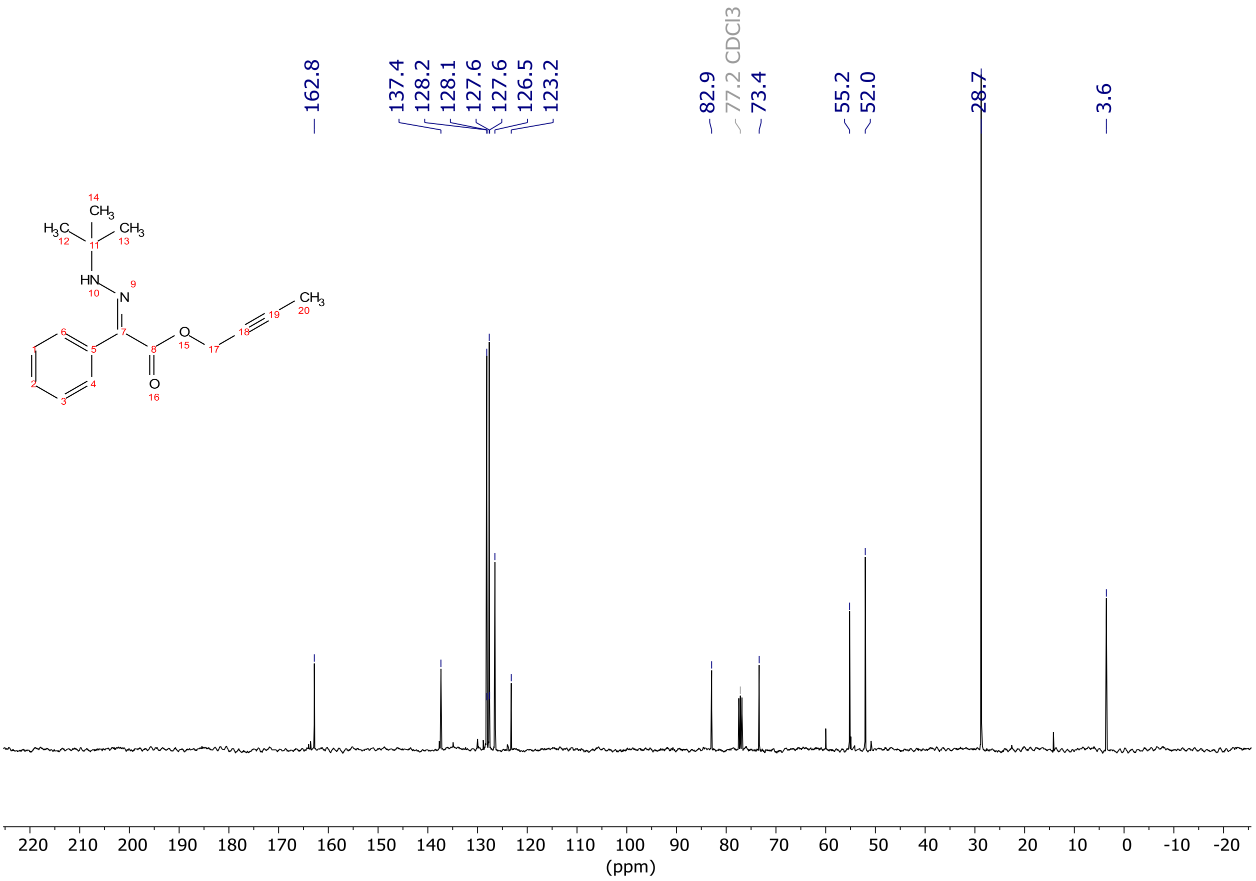

Figure S 94 ^{13}C NMR spectrum of 2.18

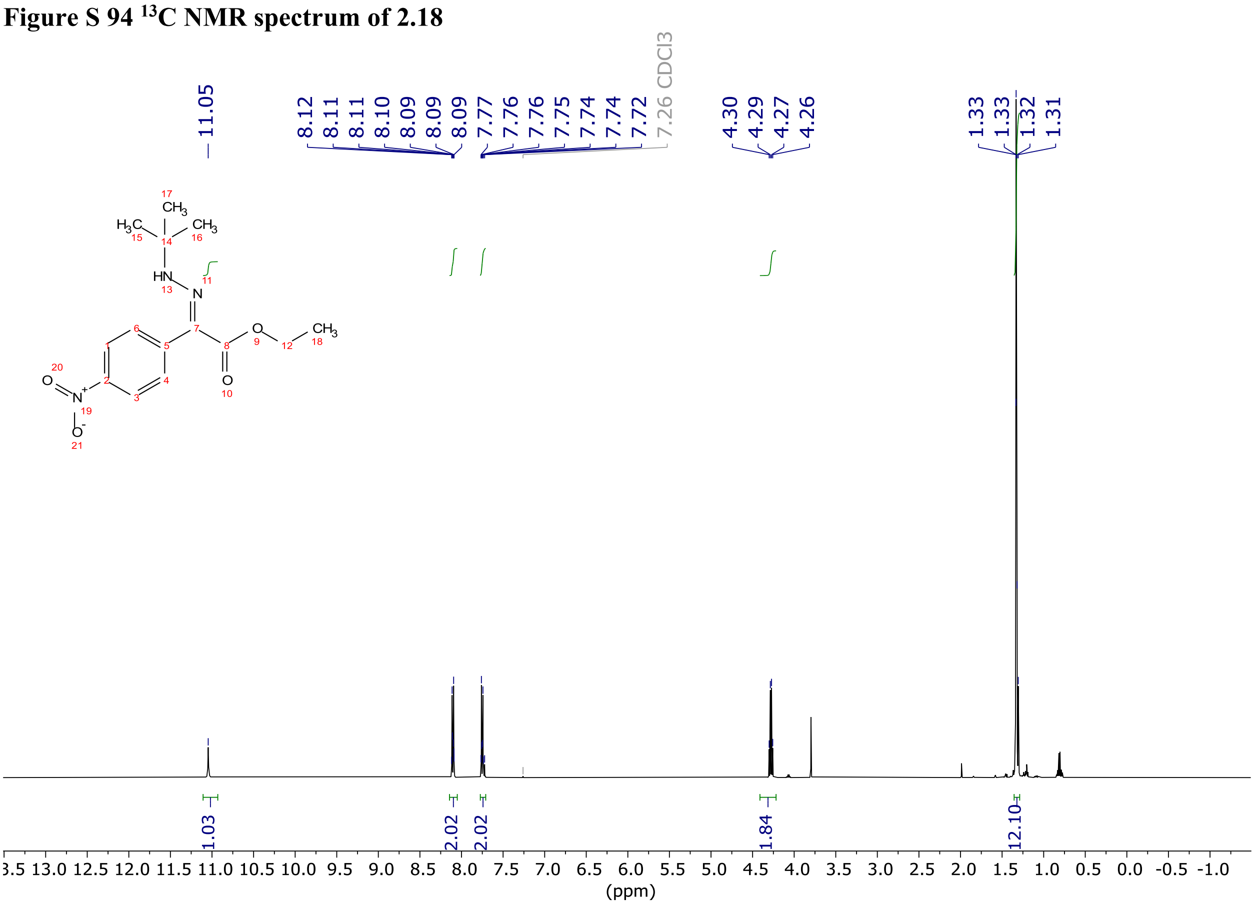

Figure S 95 ^{1}H NMR spectrum of 2.19

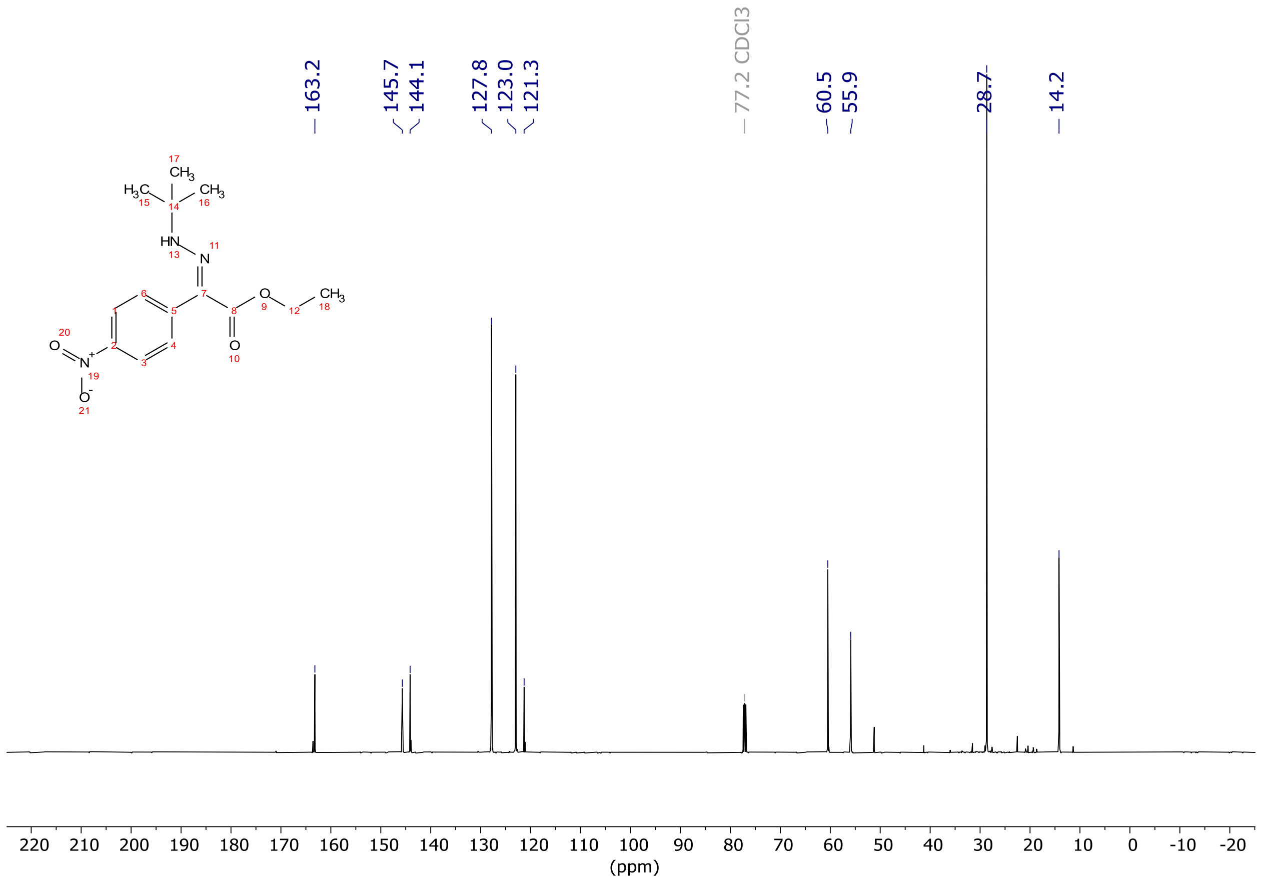

Figure S 96 ^{13}C NMR spectrum of 2.19

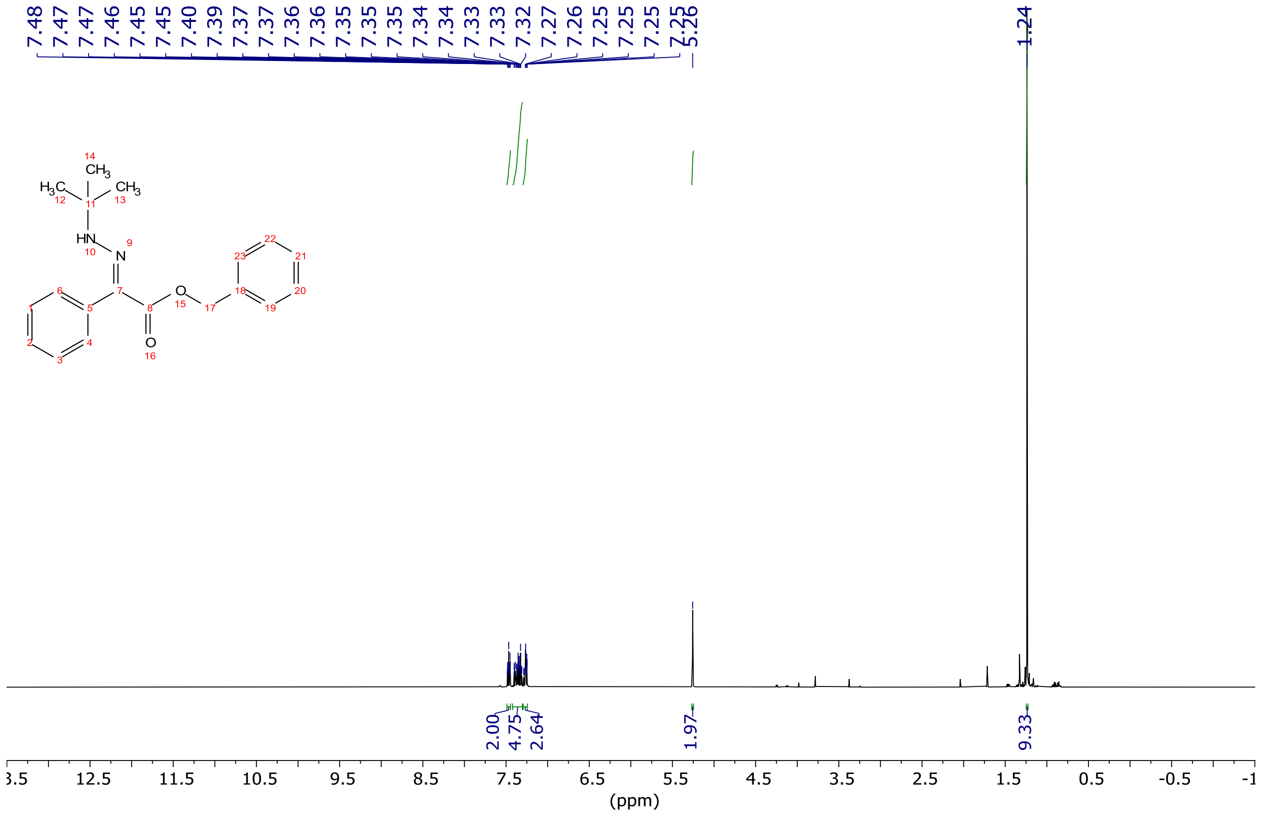

Figure S 97 ^{1}H NMR spectrum of 2.20

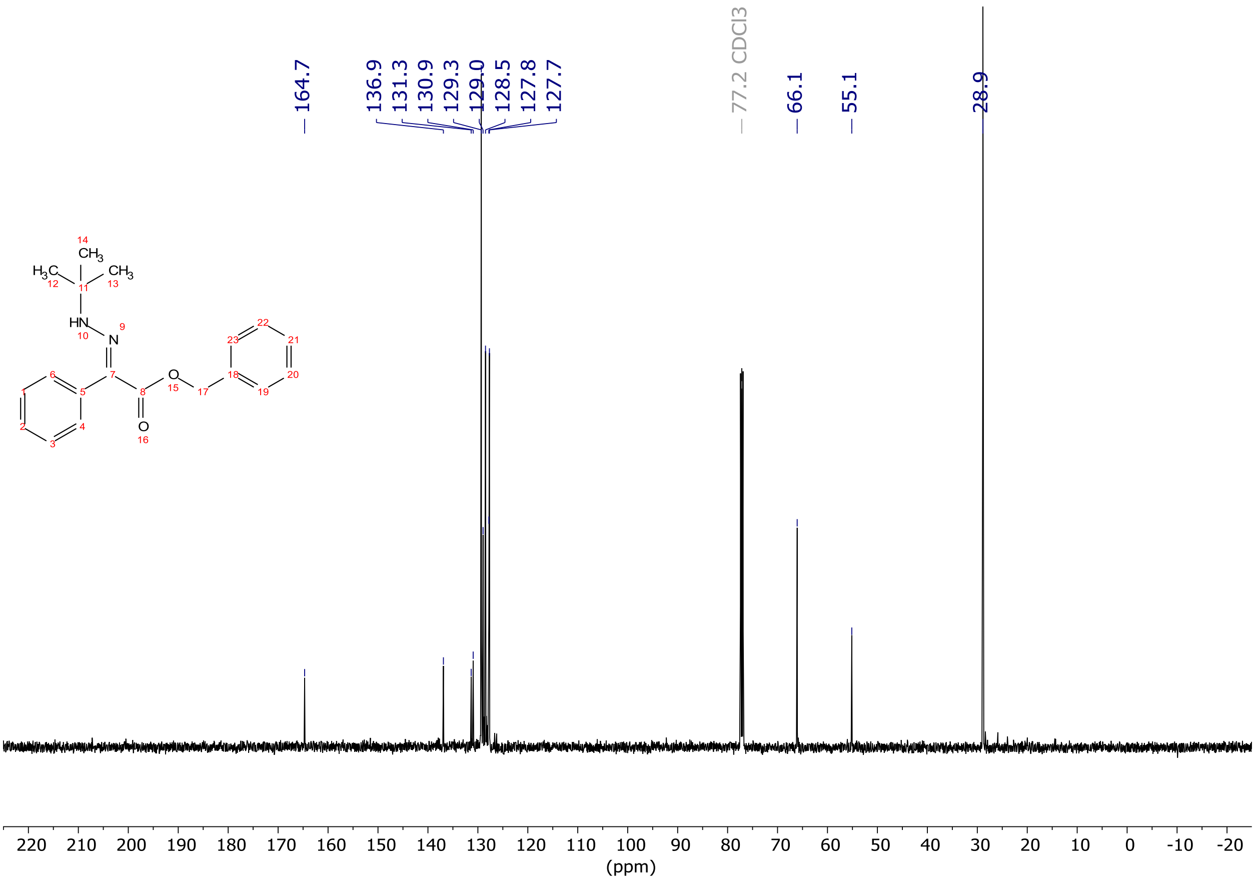

Figure S 98 ^{13}C NMR spectrum of 2.20

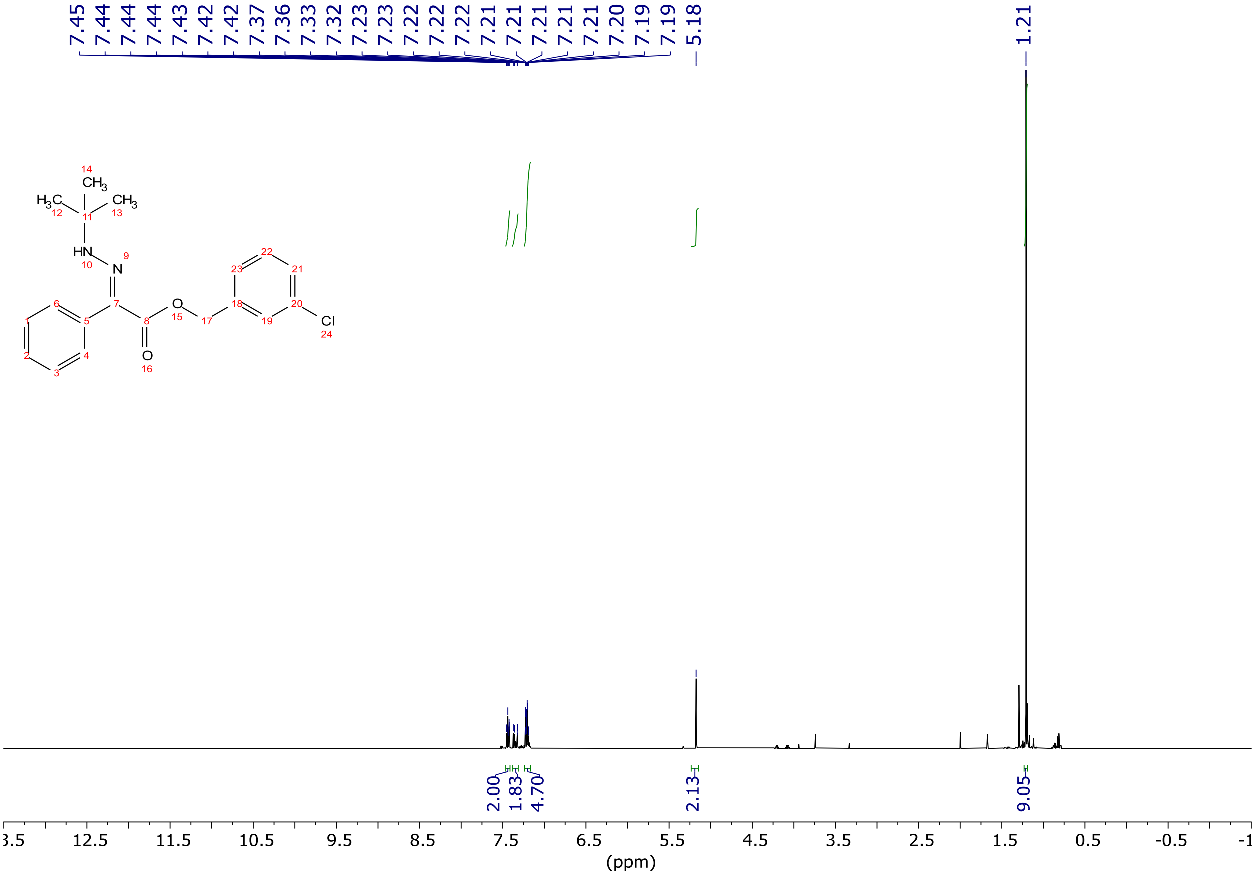

Figure S 99 ^{1}H NMR spectrum of 2.21

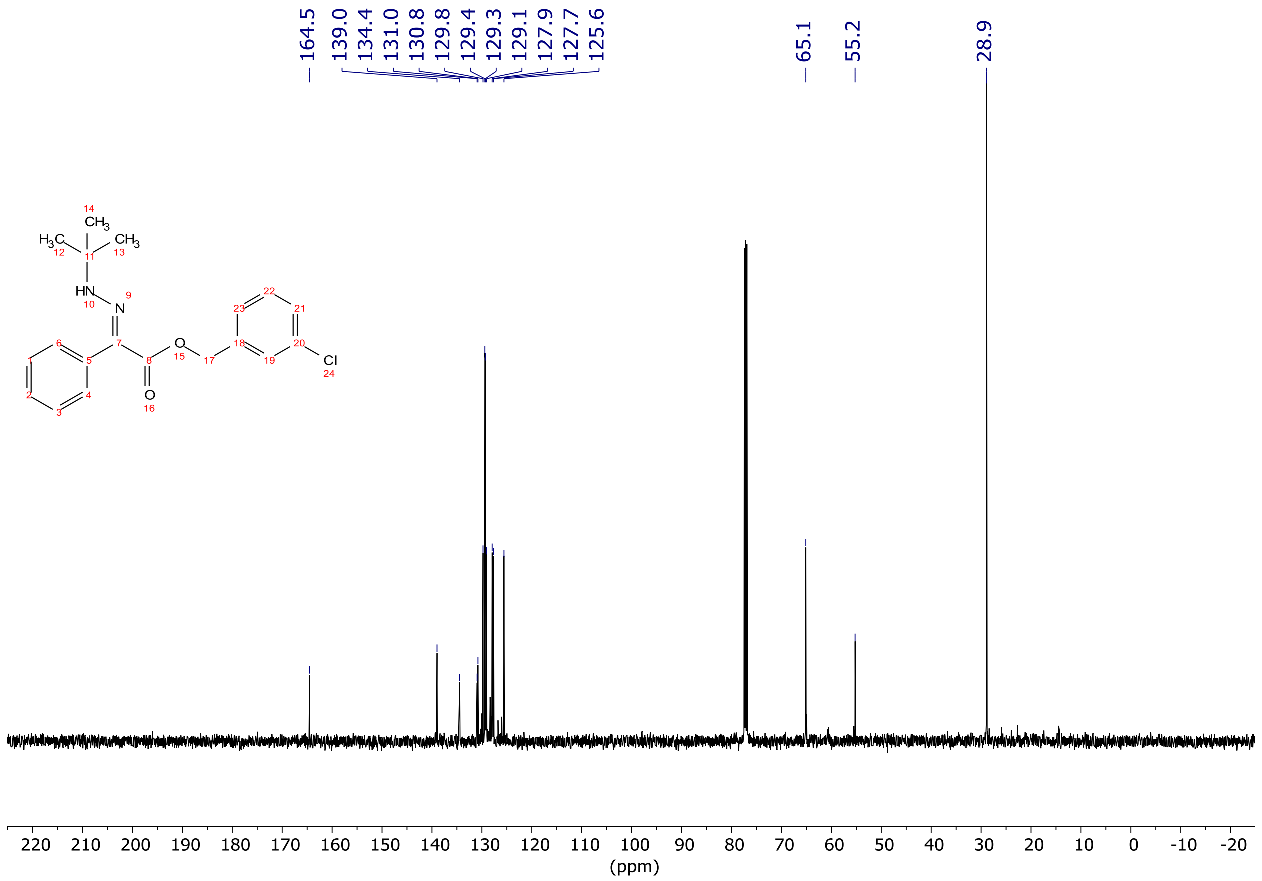

Figure S 100 ^{13}C NMR spectrum of 2.21

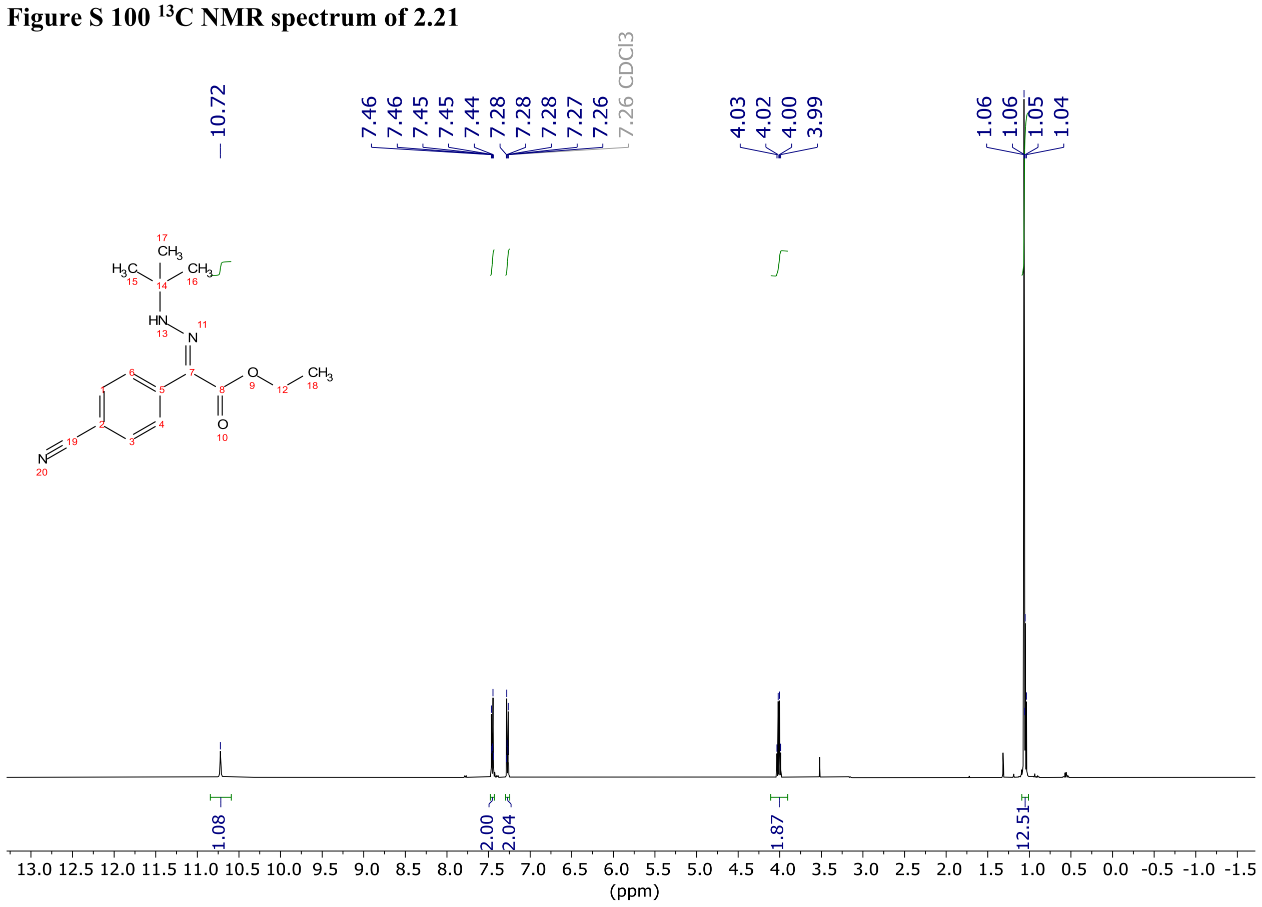

Figure S 101 ^{1}H NMR spectrum of 2.22

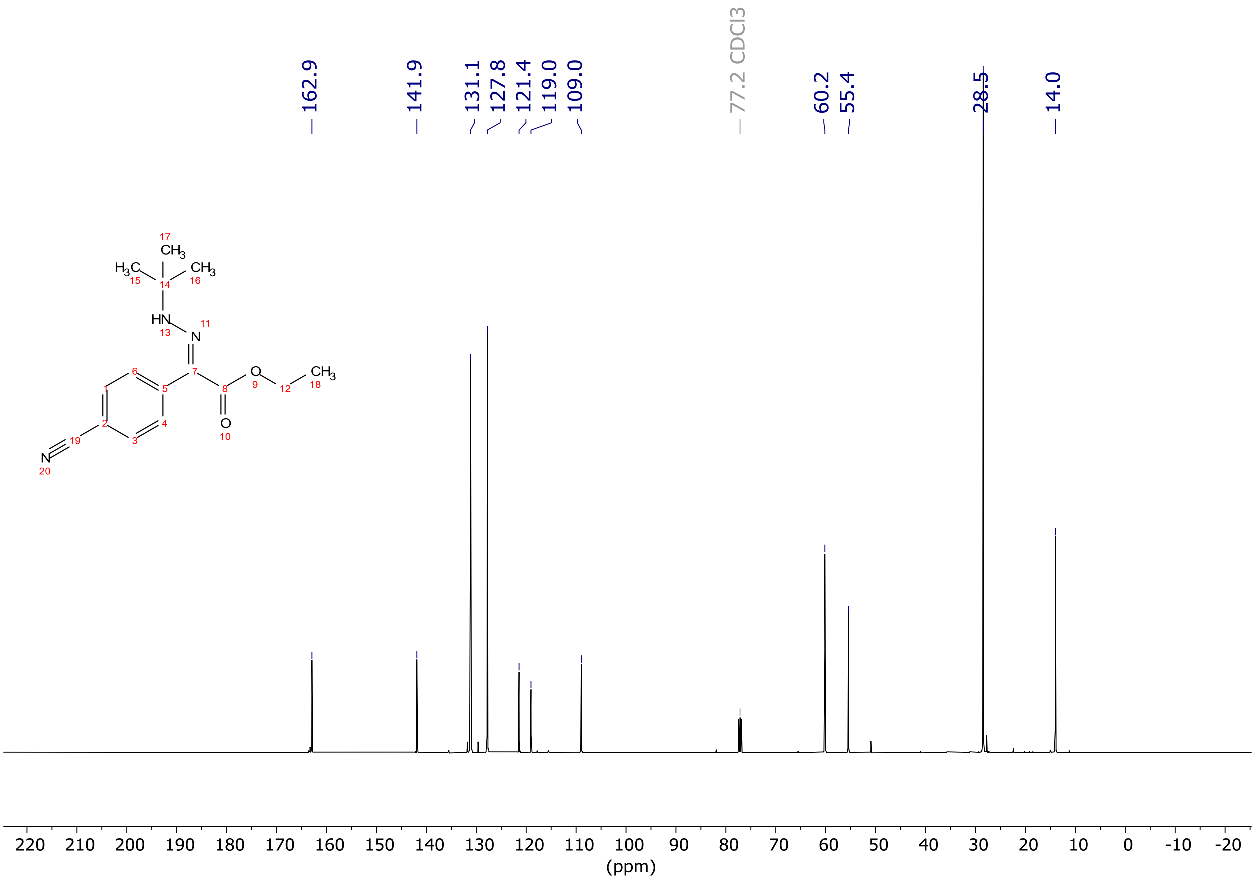

Figure S 102 ^{13}C NMR spectrum of 2.22

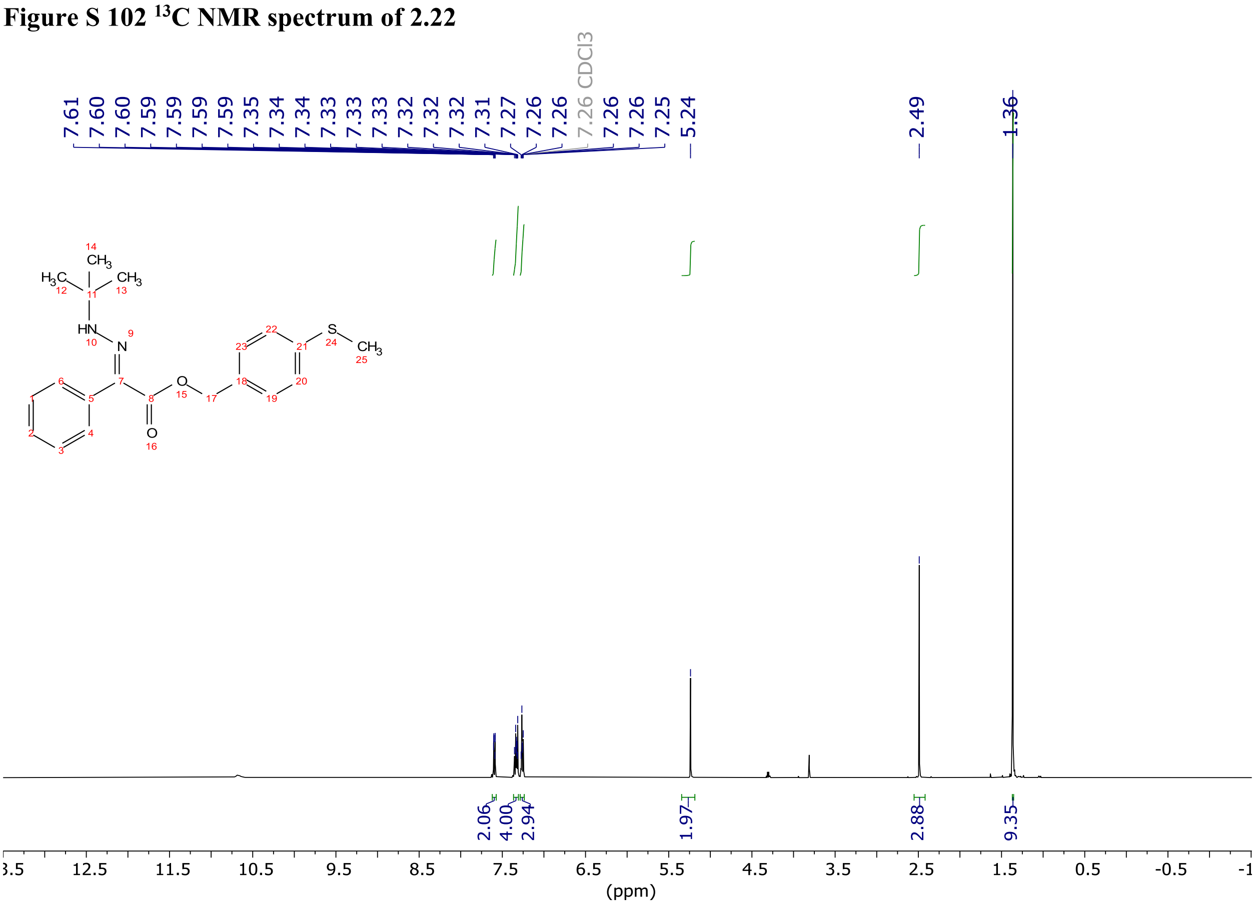

Figure S 103 ^{1}H NMR spectrum of 2.23

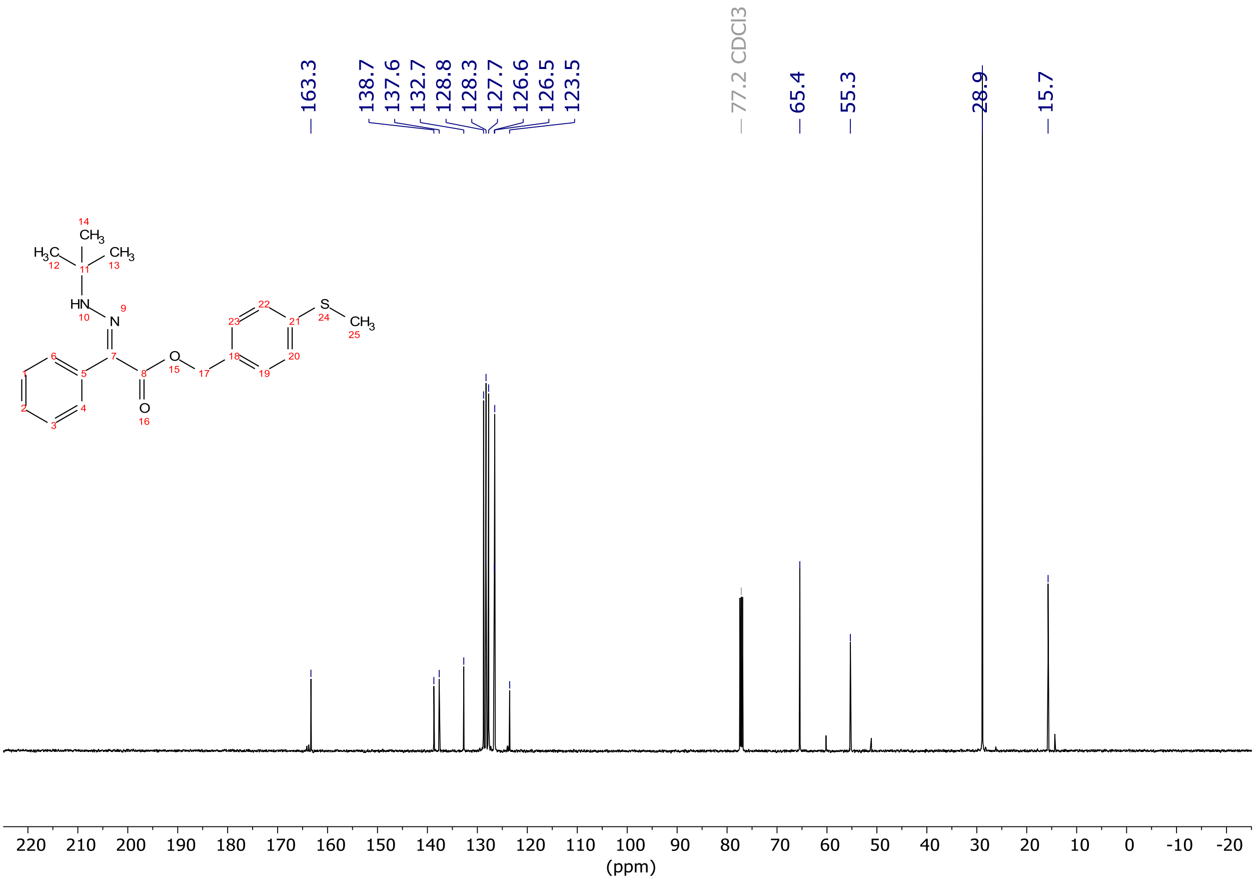

Figure S 104 ^{13}C NMR spectrum of 2.23

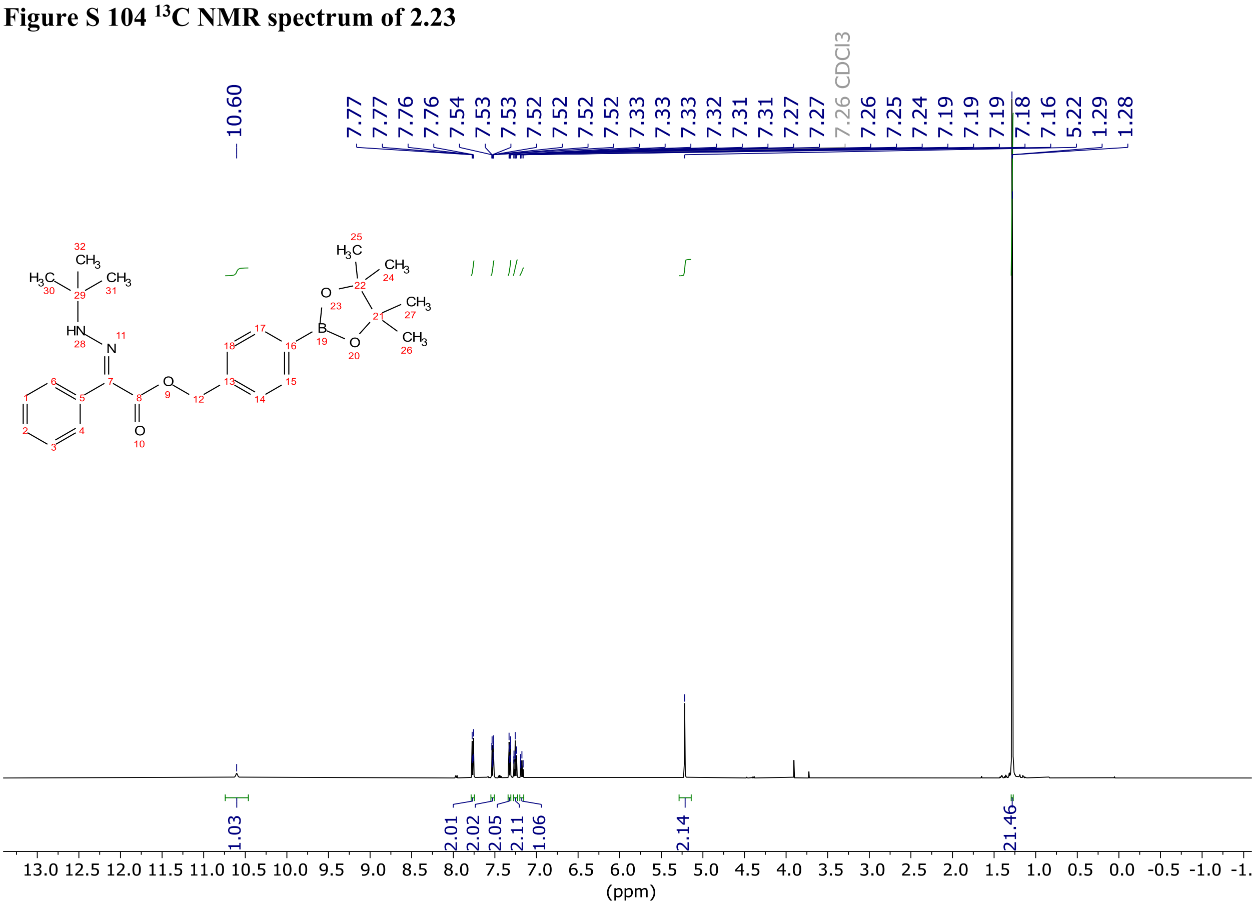

Figure S 105 ^{1}H NMR spectrum of 2.24

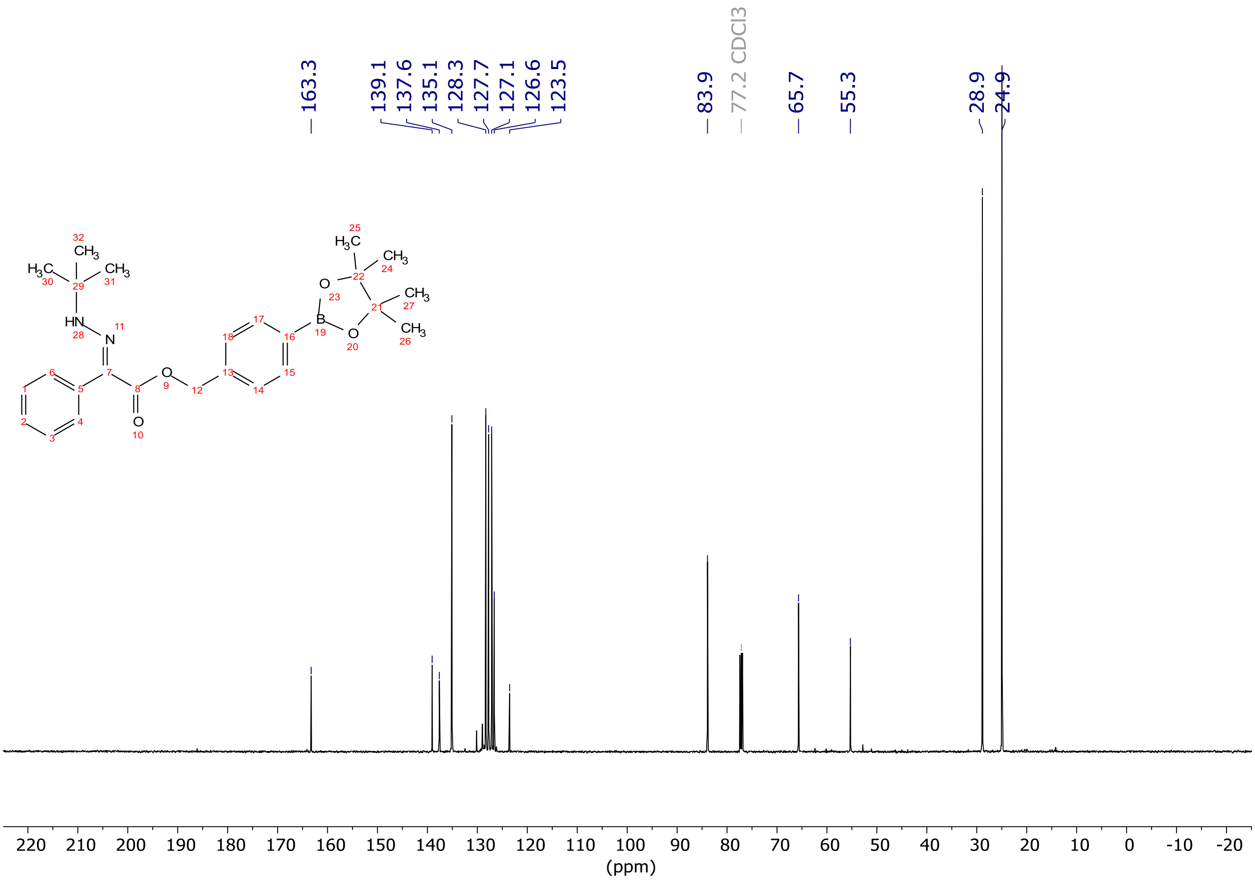

Figure S 106 ^{13}C NMR spectrum of 2.24

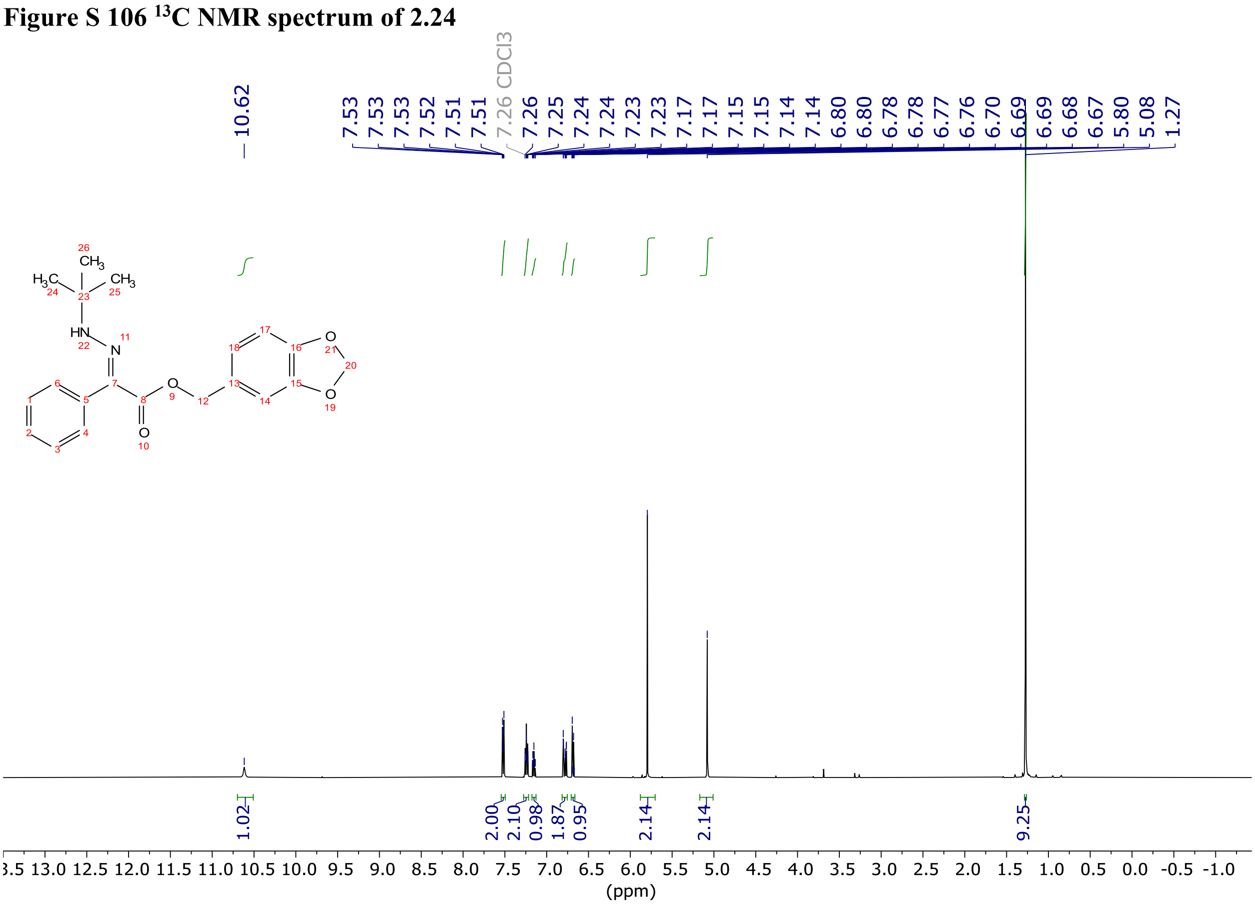

Figure S 107 ^{1}H NMR spectrum of 2.25

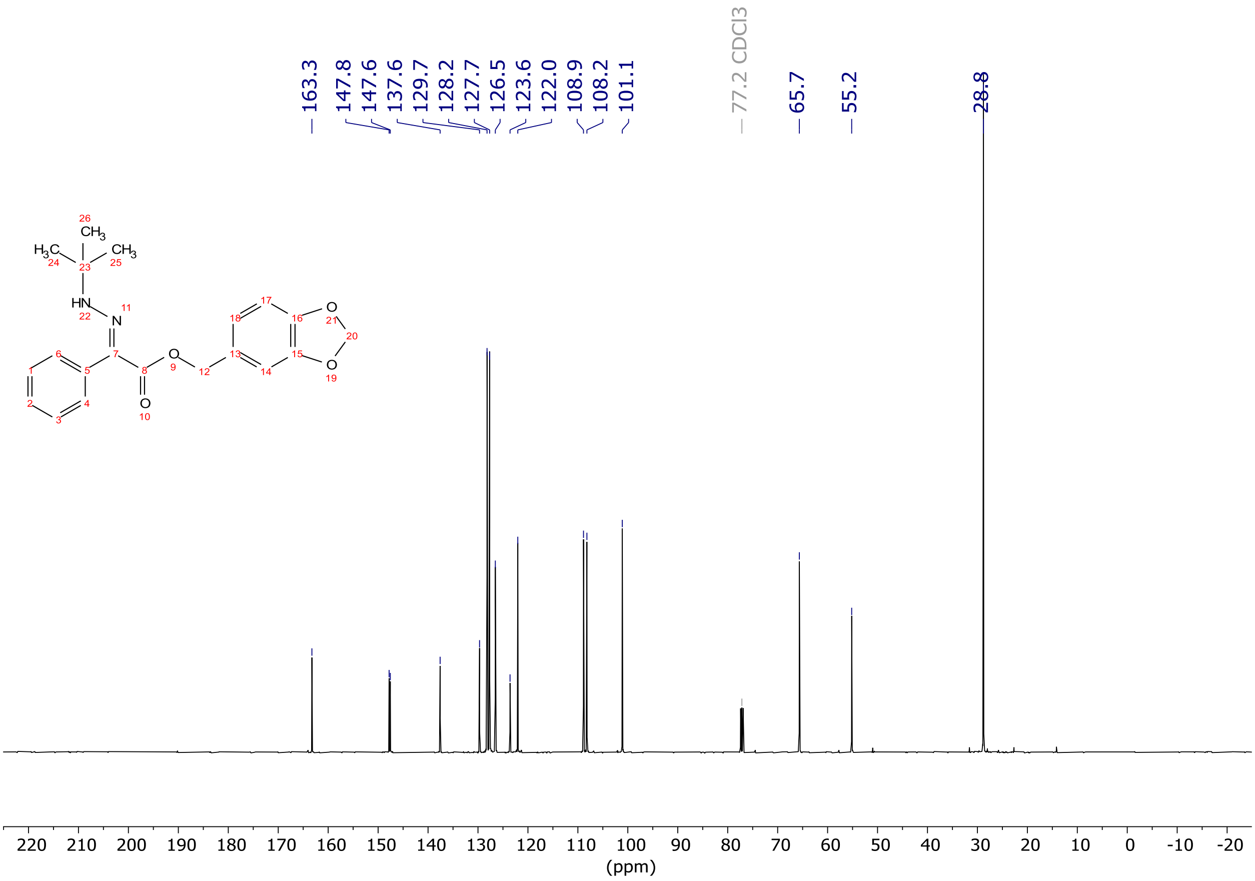

Figure S 108 ^{13}C NMR spectrum of 2.25

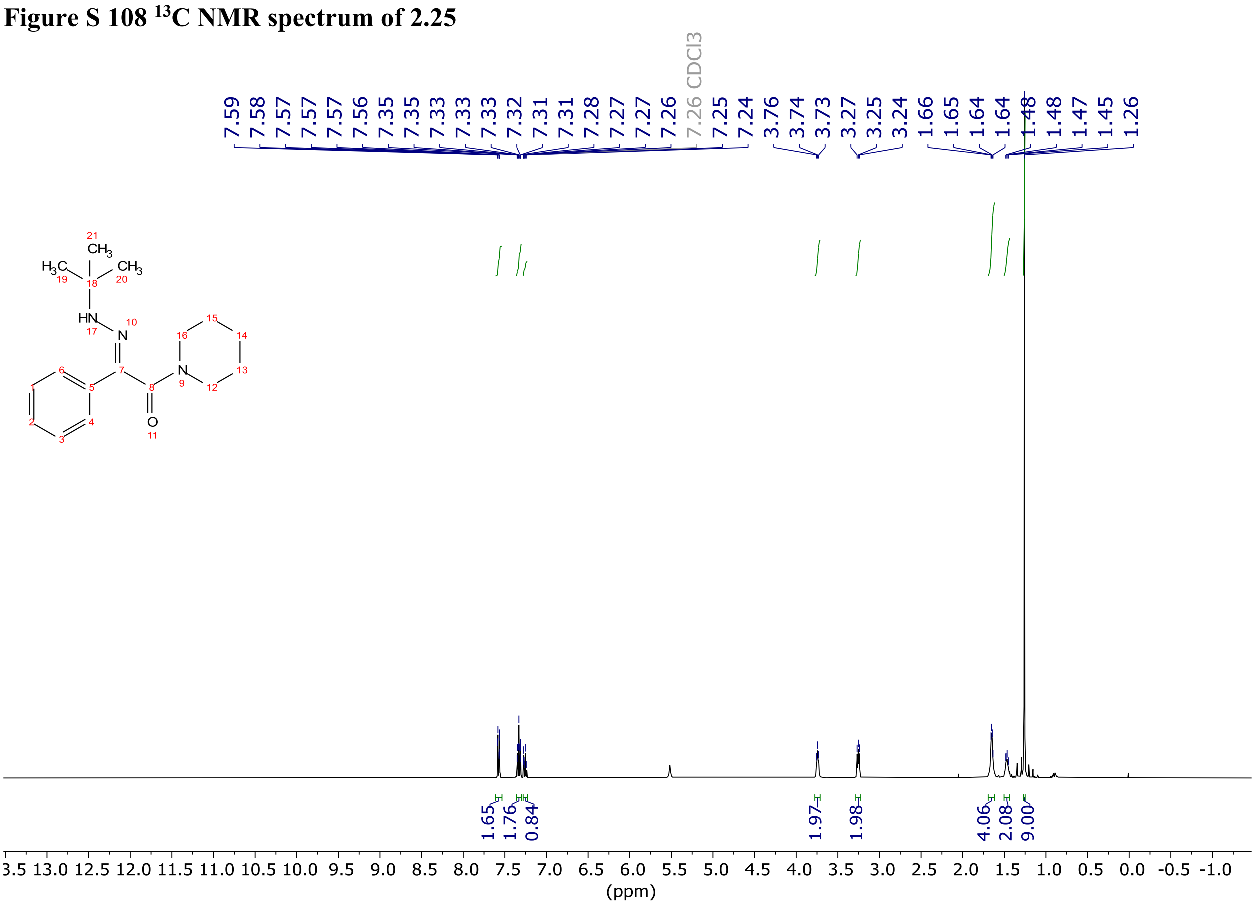

Figure S 109 ^{1}H NMR spectrum of 2.26

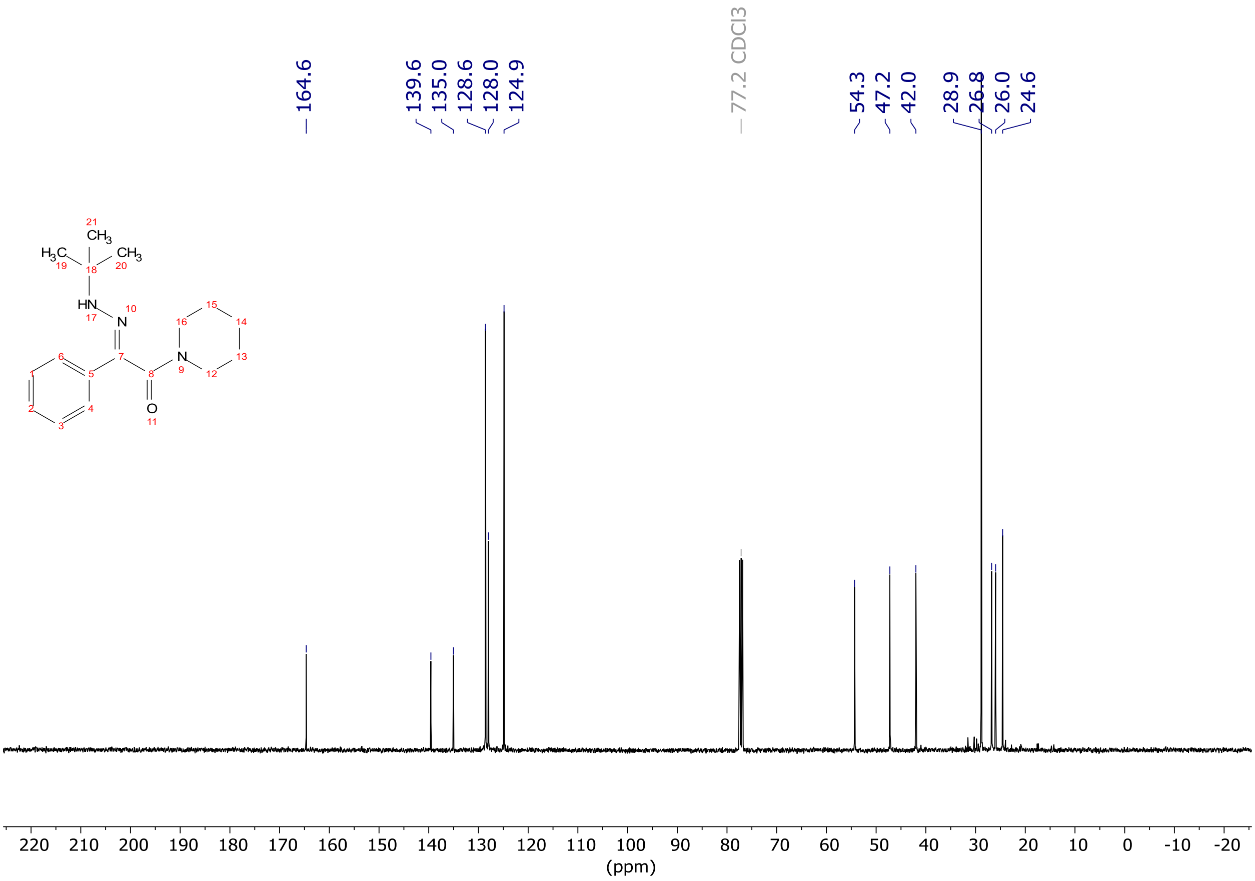

Figure S 110 ^{13}C NMR spectrum of 2.26

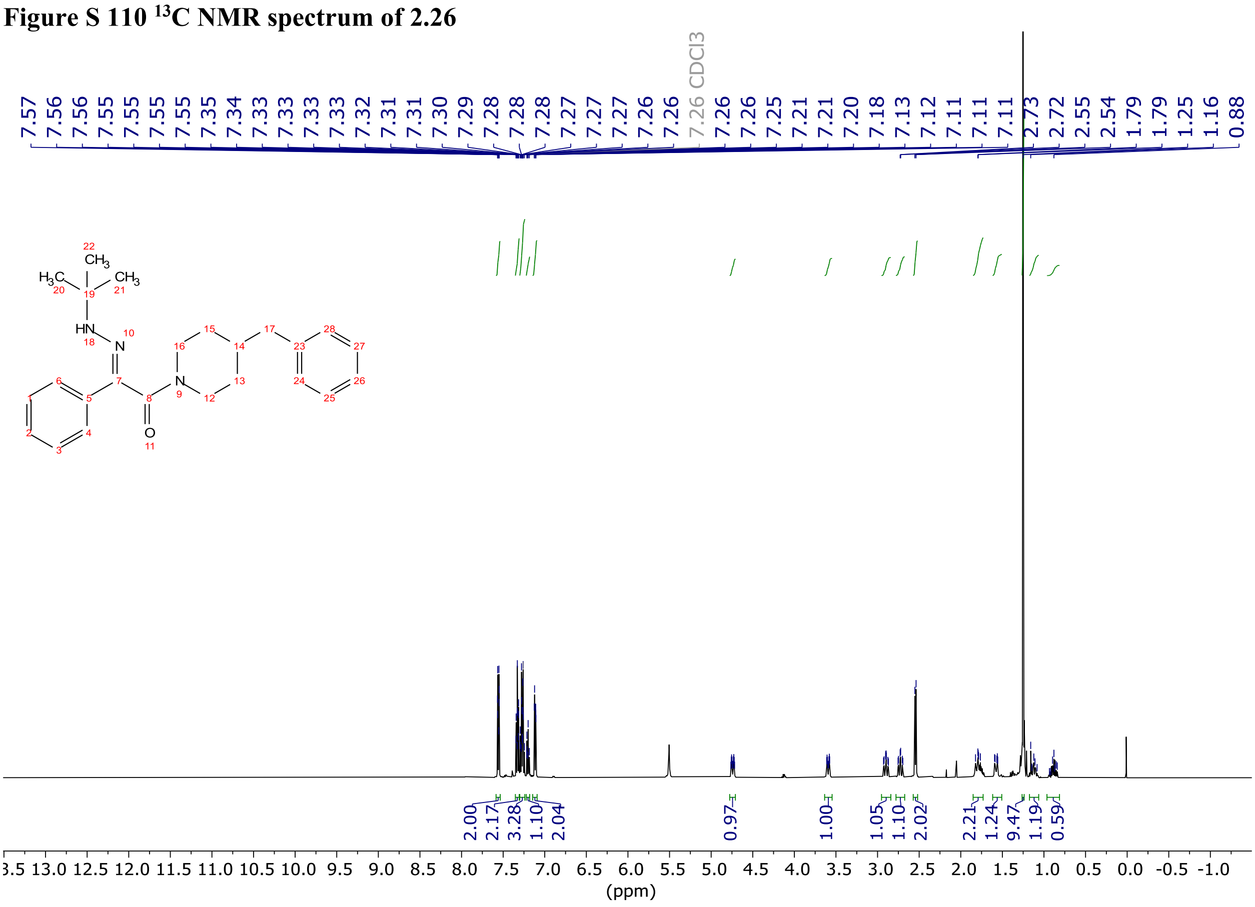

Figure S 111 ^{1}H NMR spectrum of 2.27

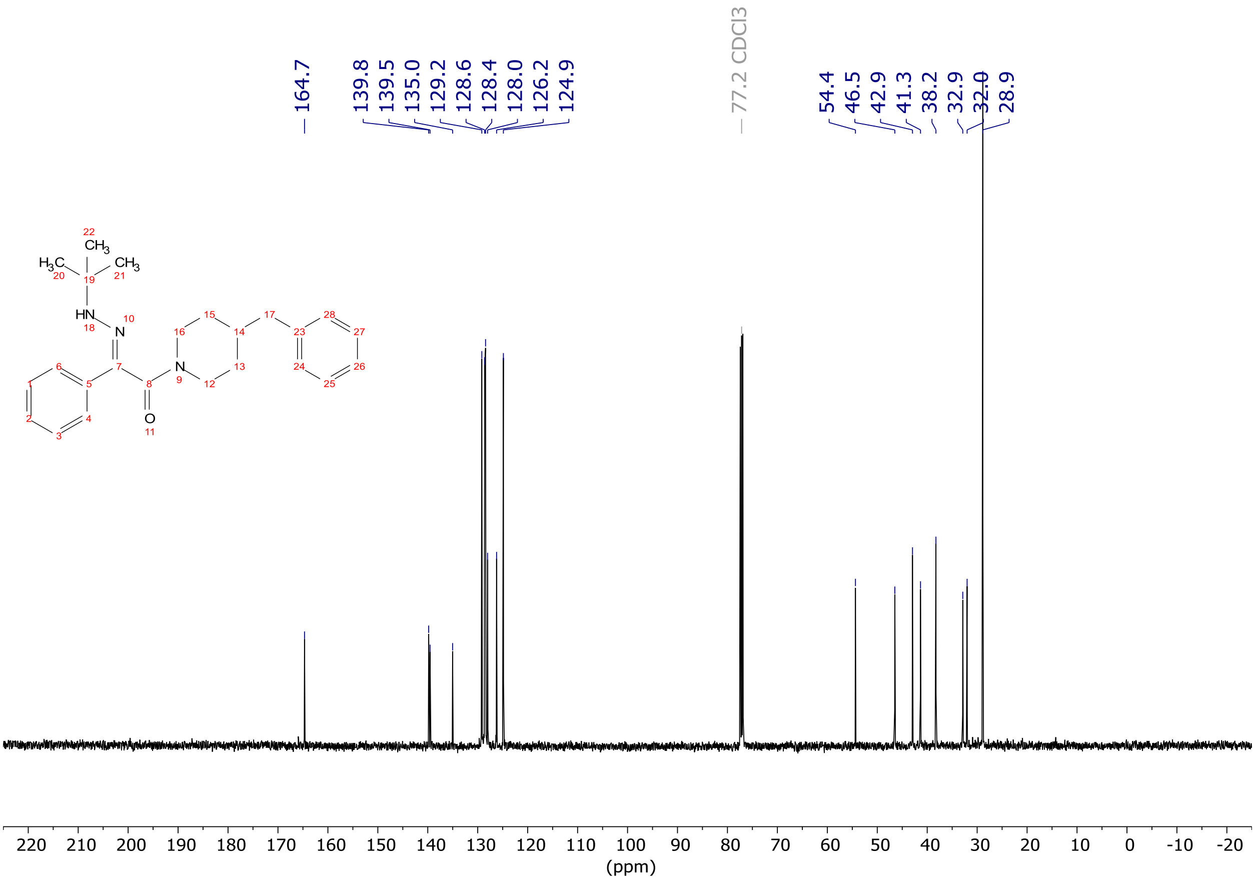

Figure S 112 ^{13}C NMR spectrum of 2.27

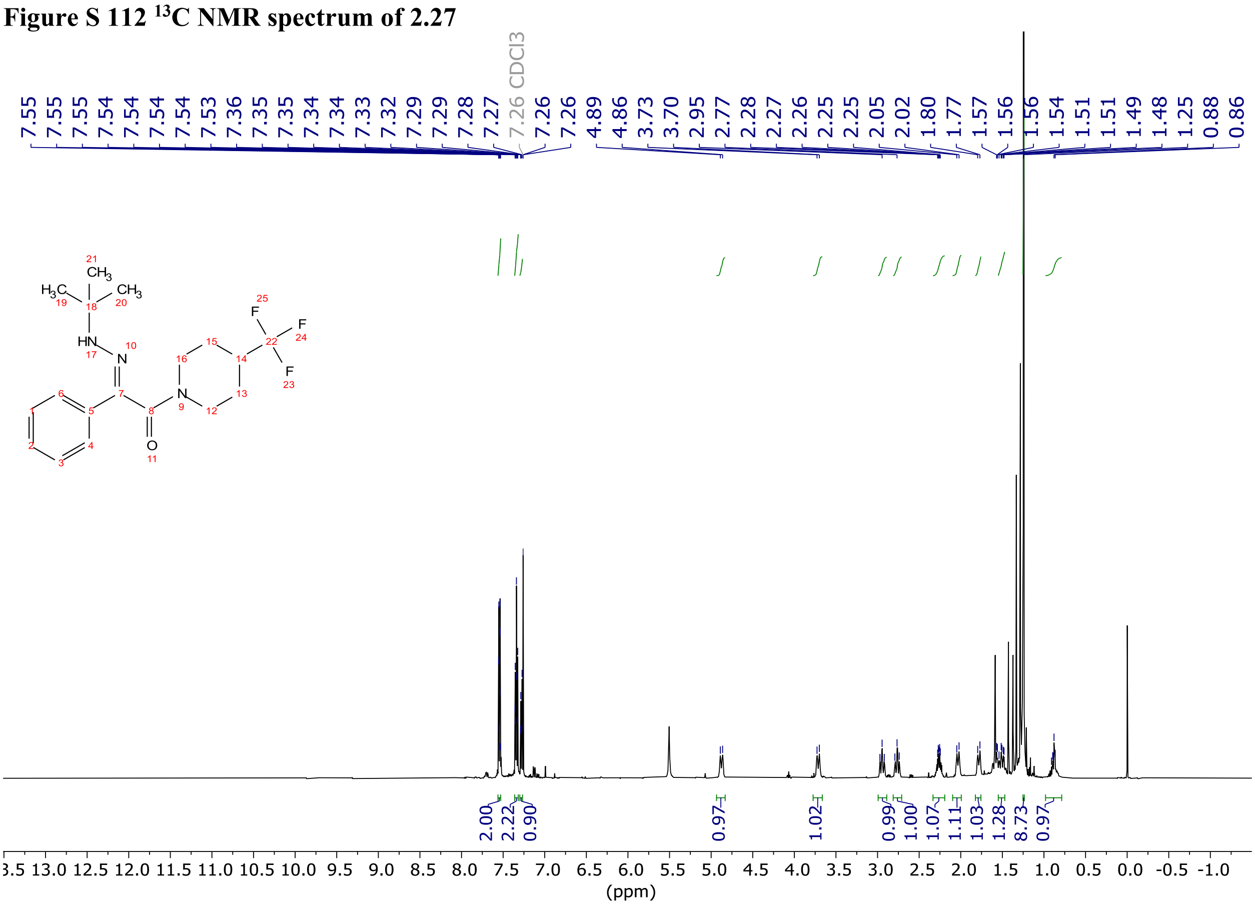

Figure S 113 ^{1}H NMR spectrum of 2.28

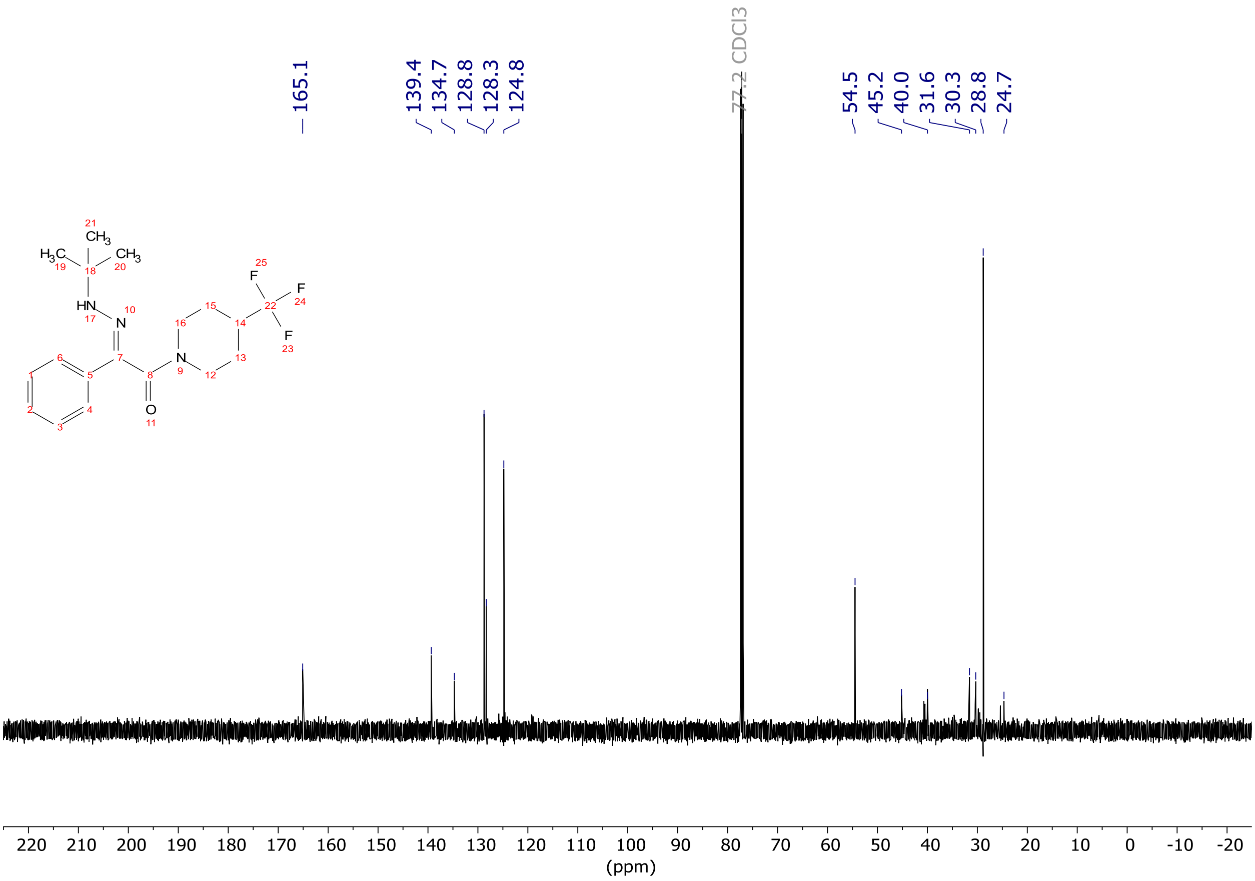

Figure S 114 ^{13}C NMR spectrum of 2.28

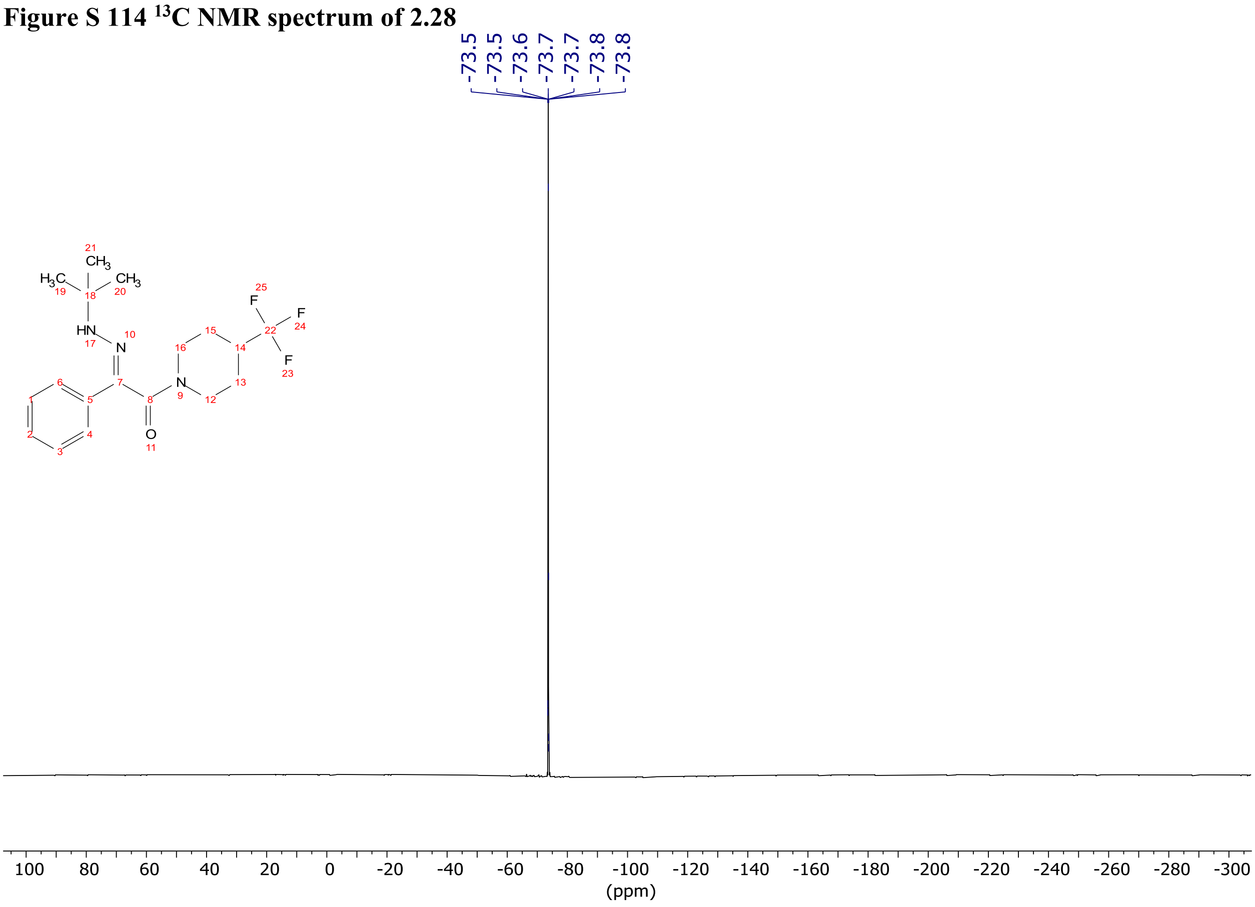

Figure S 115 ^{19}F NMR spectrum of 2.28

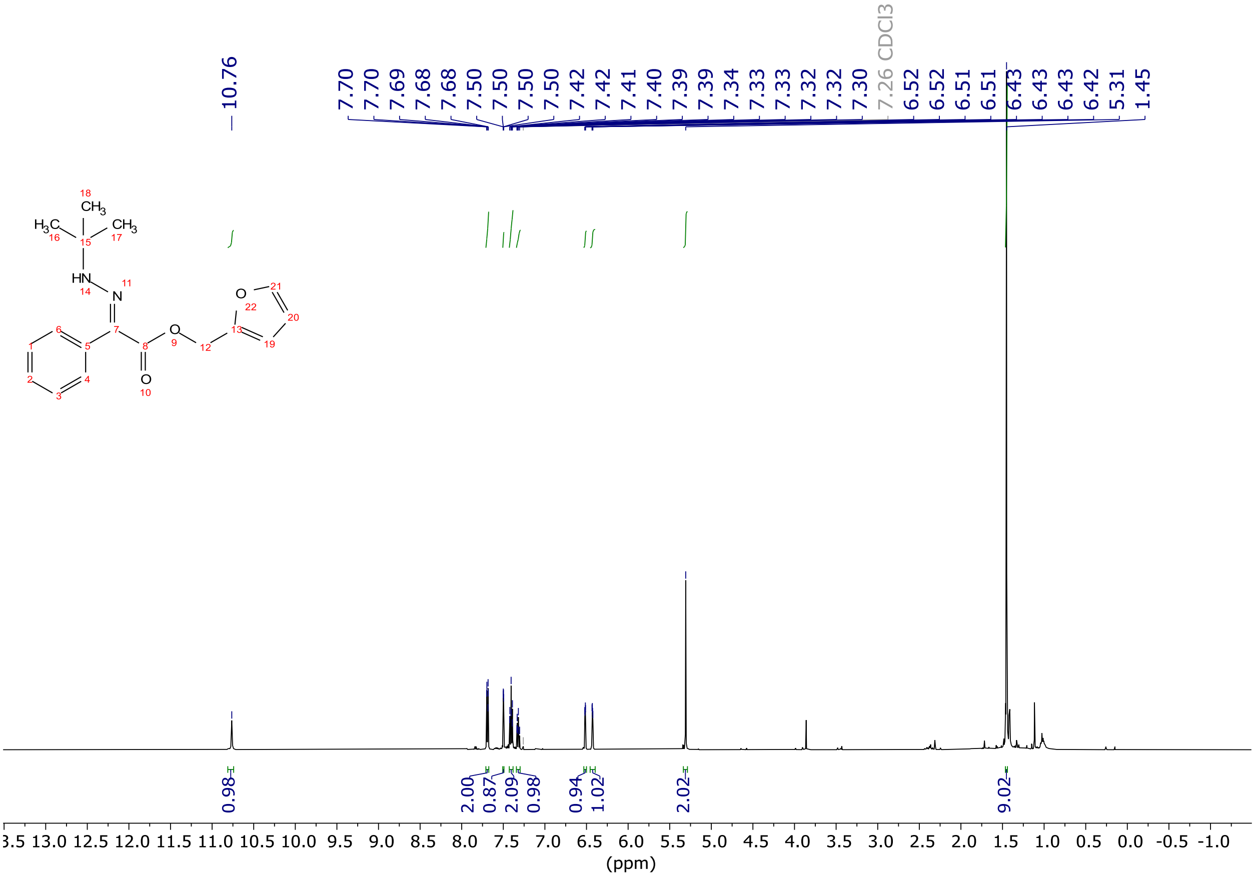

Figure S 116 ^{1}H NMR spectrum of 2.29

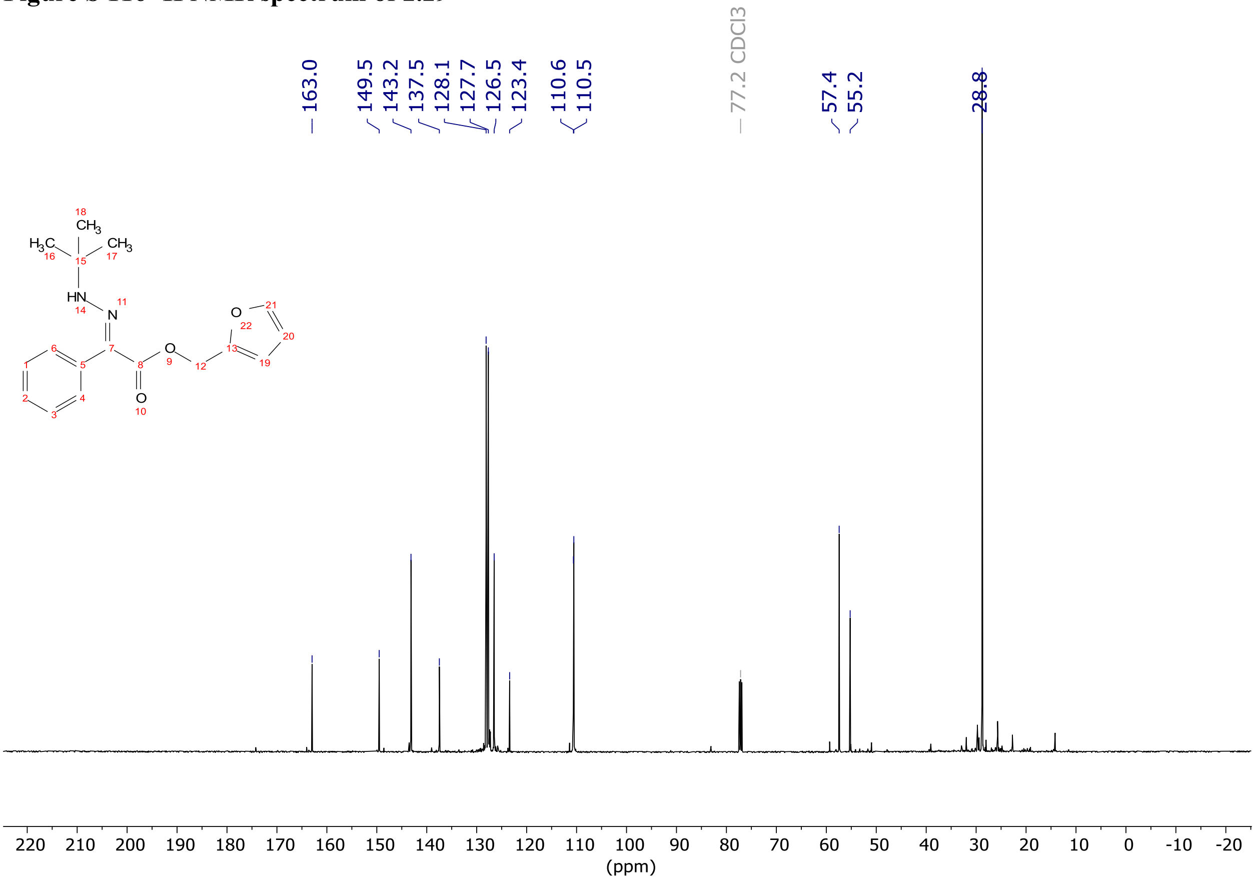

Figure S 117 ^{13}C NMR spectrum of 2.29

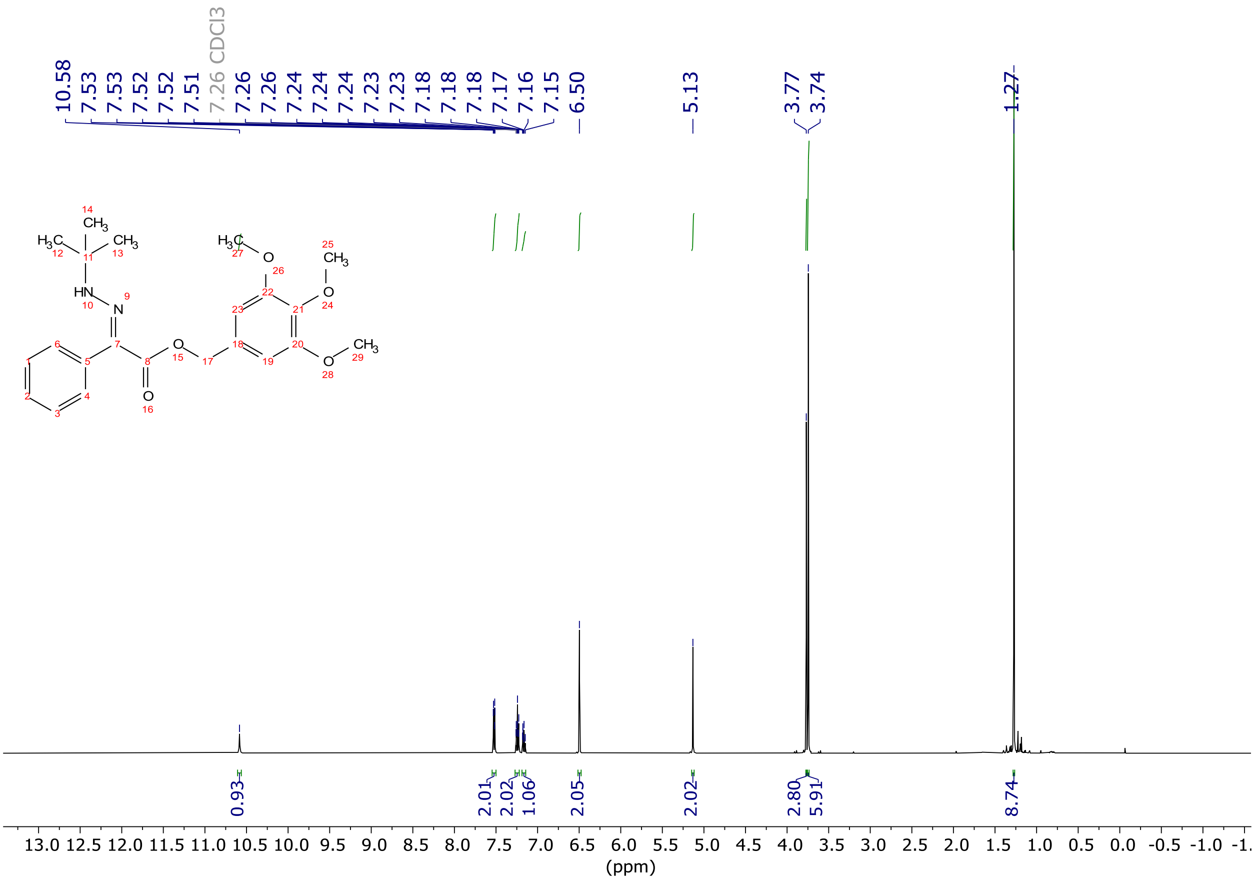

Figure S 118 ^{1}H NMR spectrum of 2.30

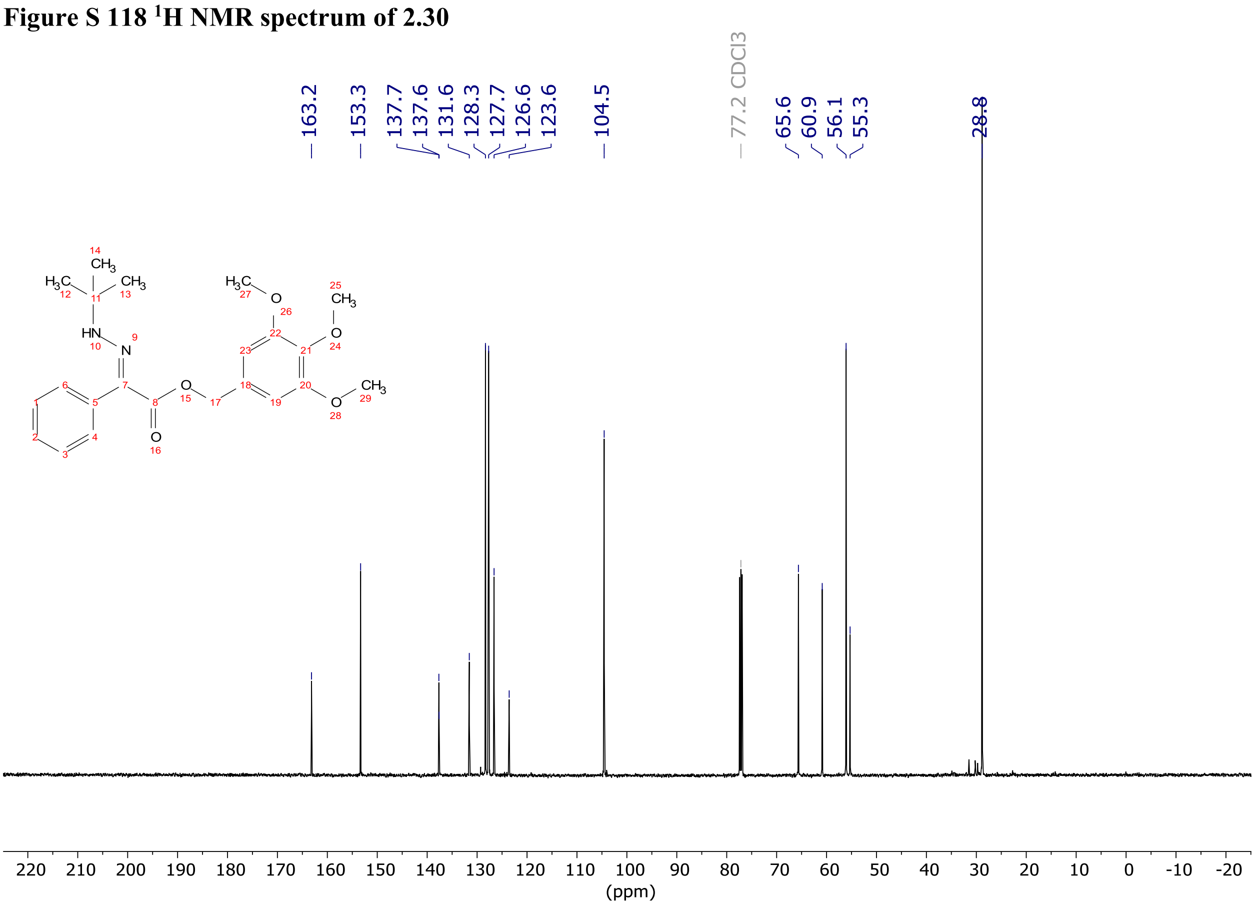

Figure S 119 ^{13}C NMR spectrum of 2.30

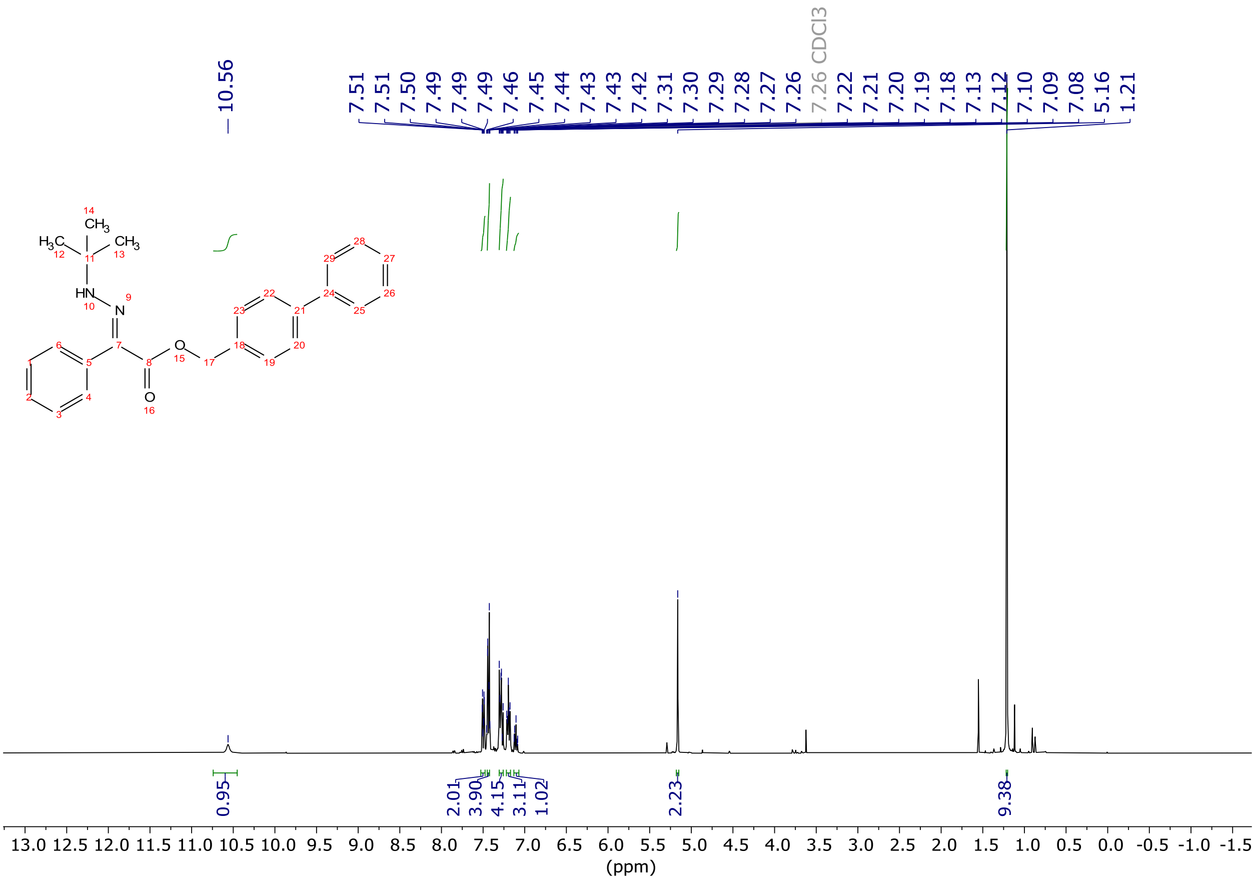

Figure S 120 ^{1}H NMR spectrum of 2.31

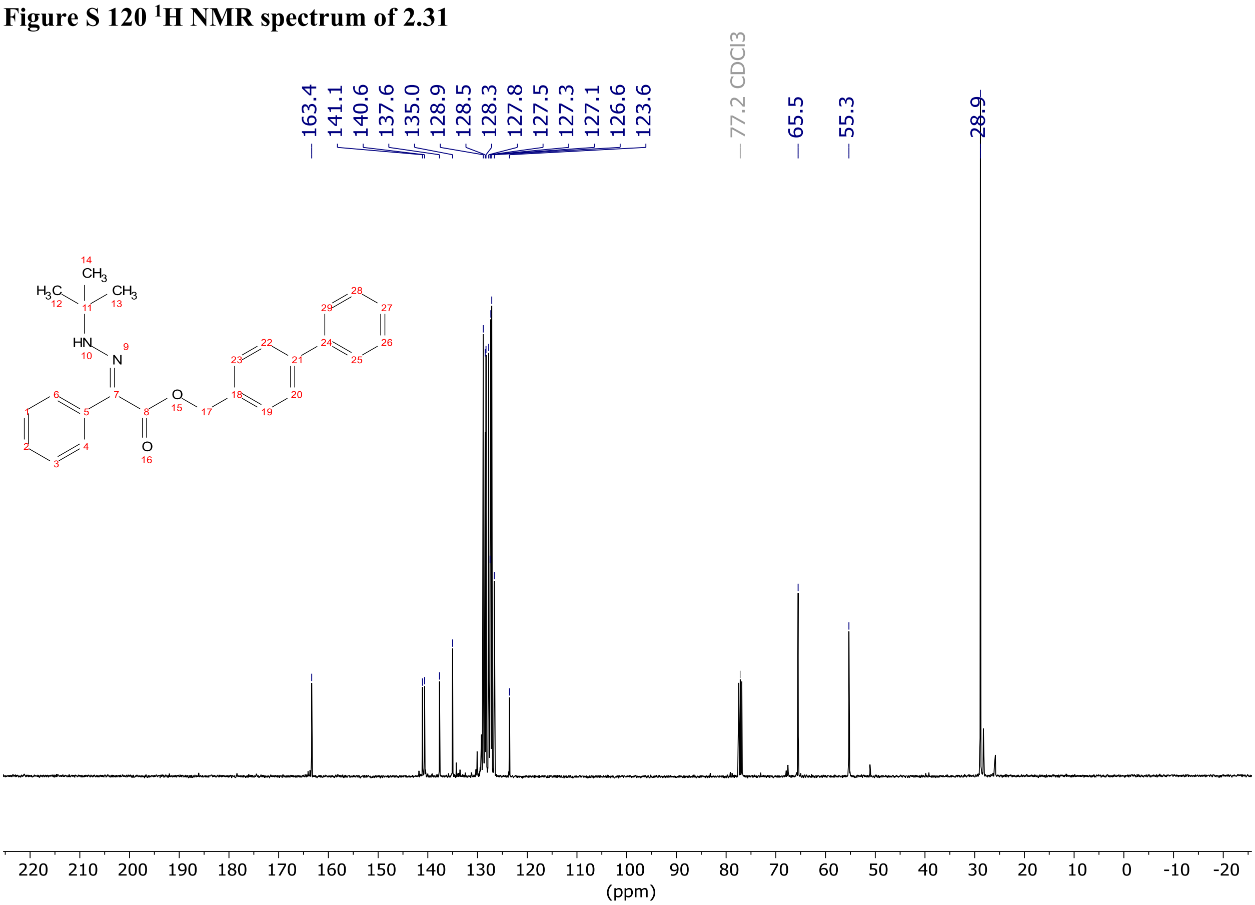

Figure S 121 ^{13}C NMR spectrum of 2.31

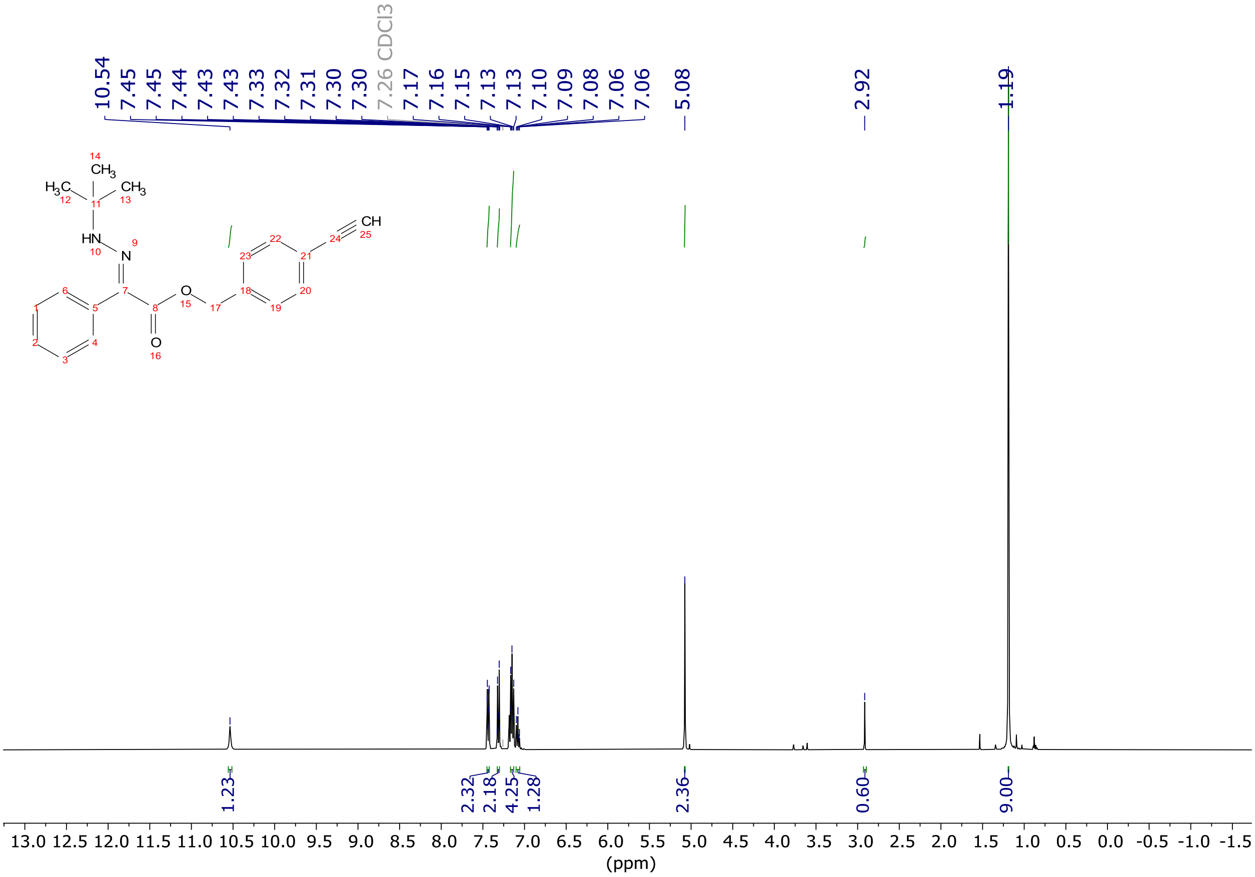

Figure S 122 ^{1}H NMR spectrum of 2.32

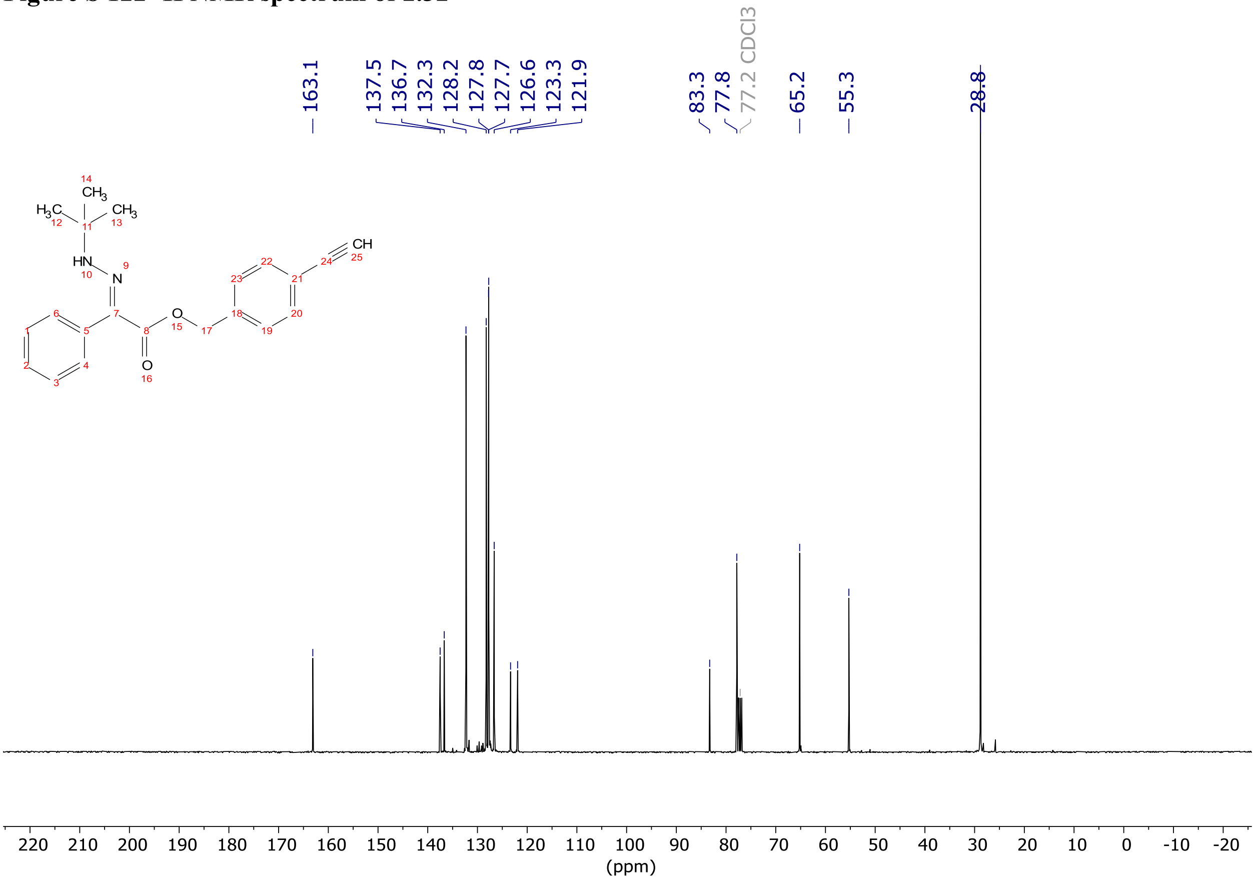

Figure S 123 ^{13}C NMR spectrum of 2.32

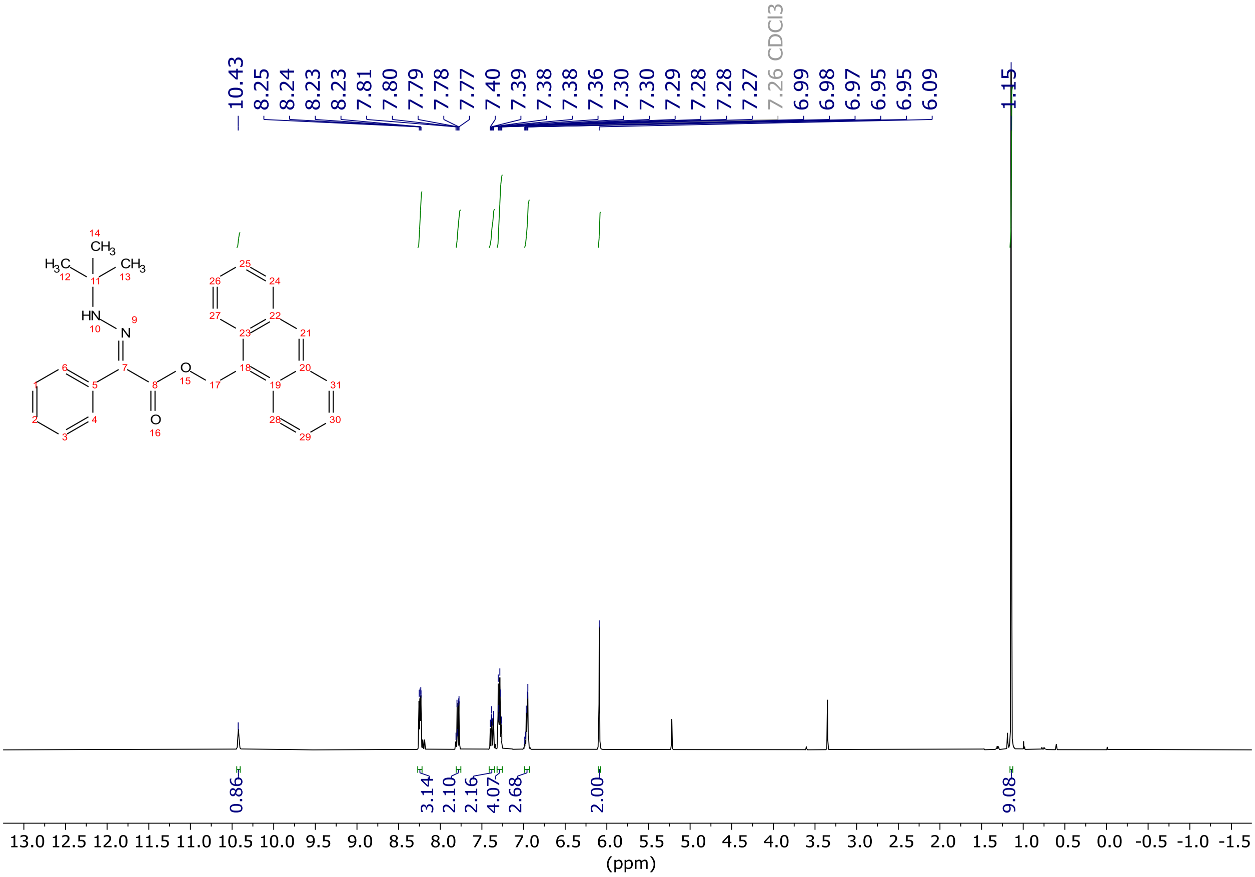

Figure S 124 ^{1}H NMR spectrum of 2.33

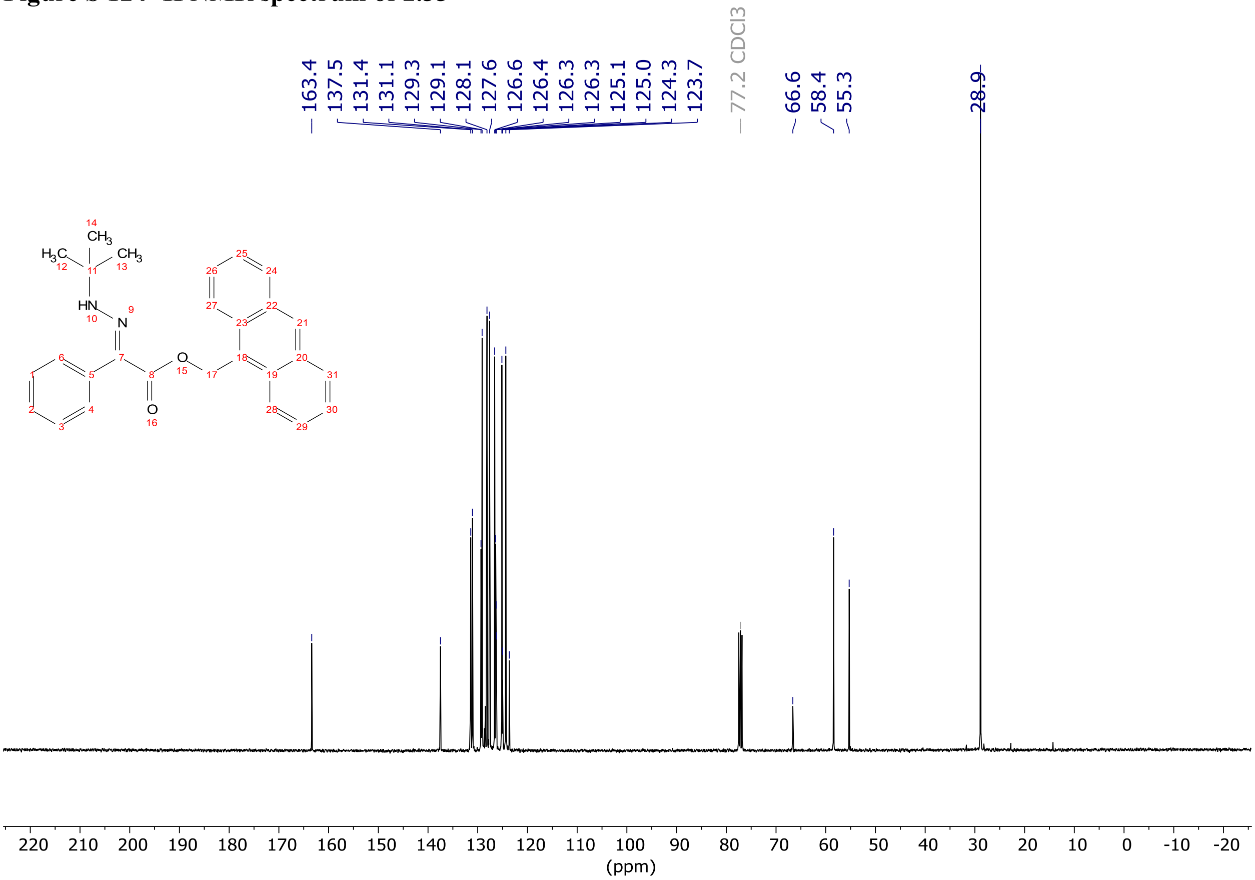

Figure S 125 ^{13}C NMR spectrum of 2.33

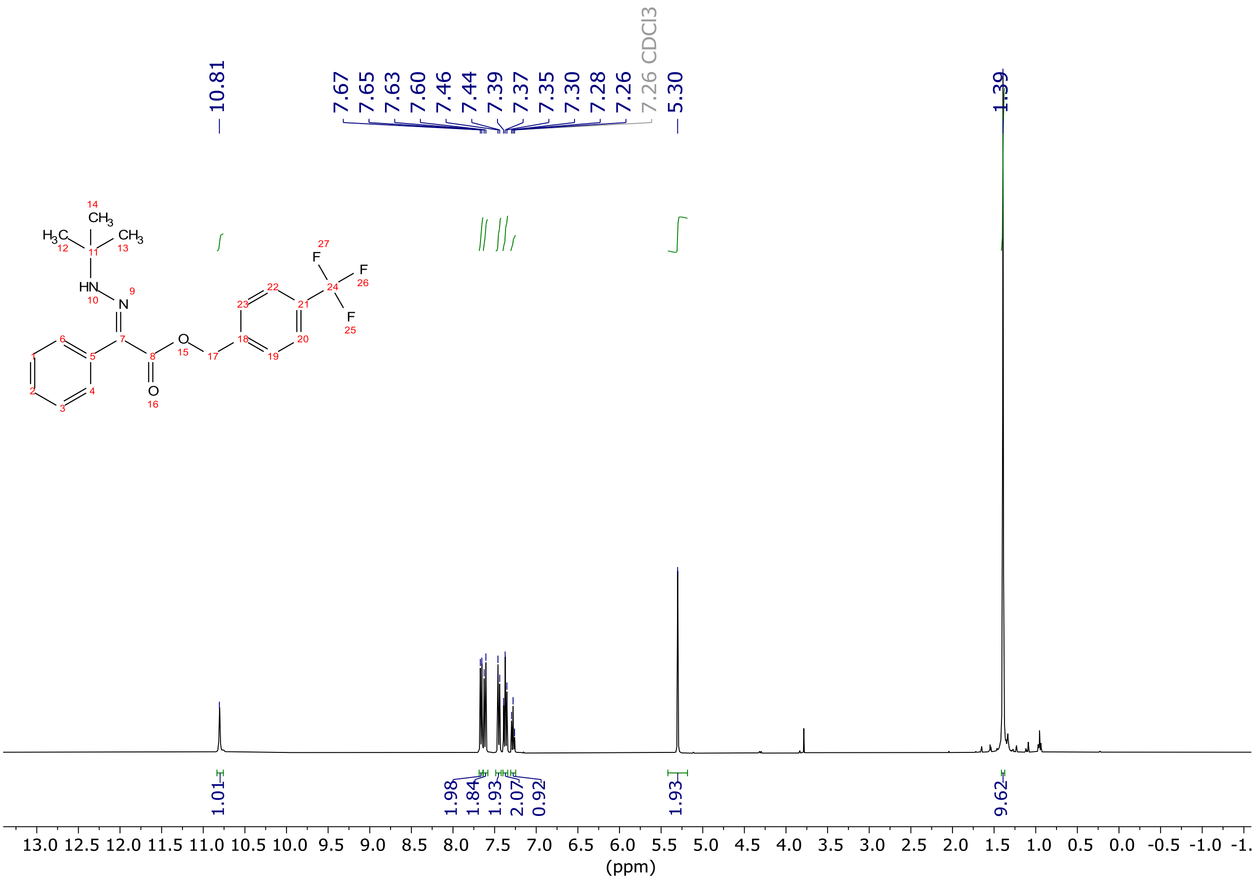

Figure S 126 ^{1}H NMR spectrum of 2.34

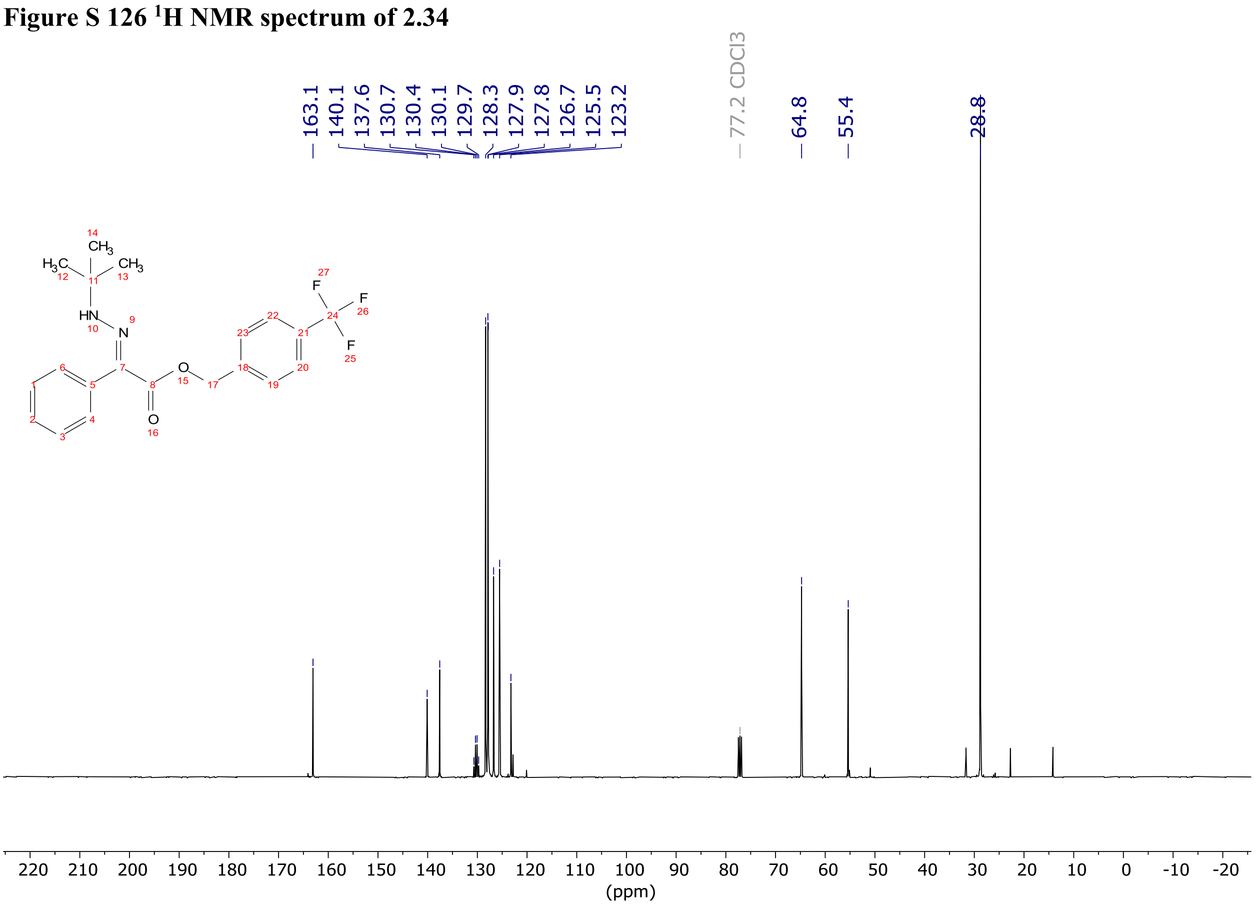

Figure S 127 ^{13}C NMR spectrum of 2.34

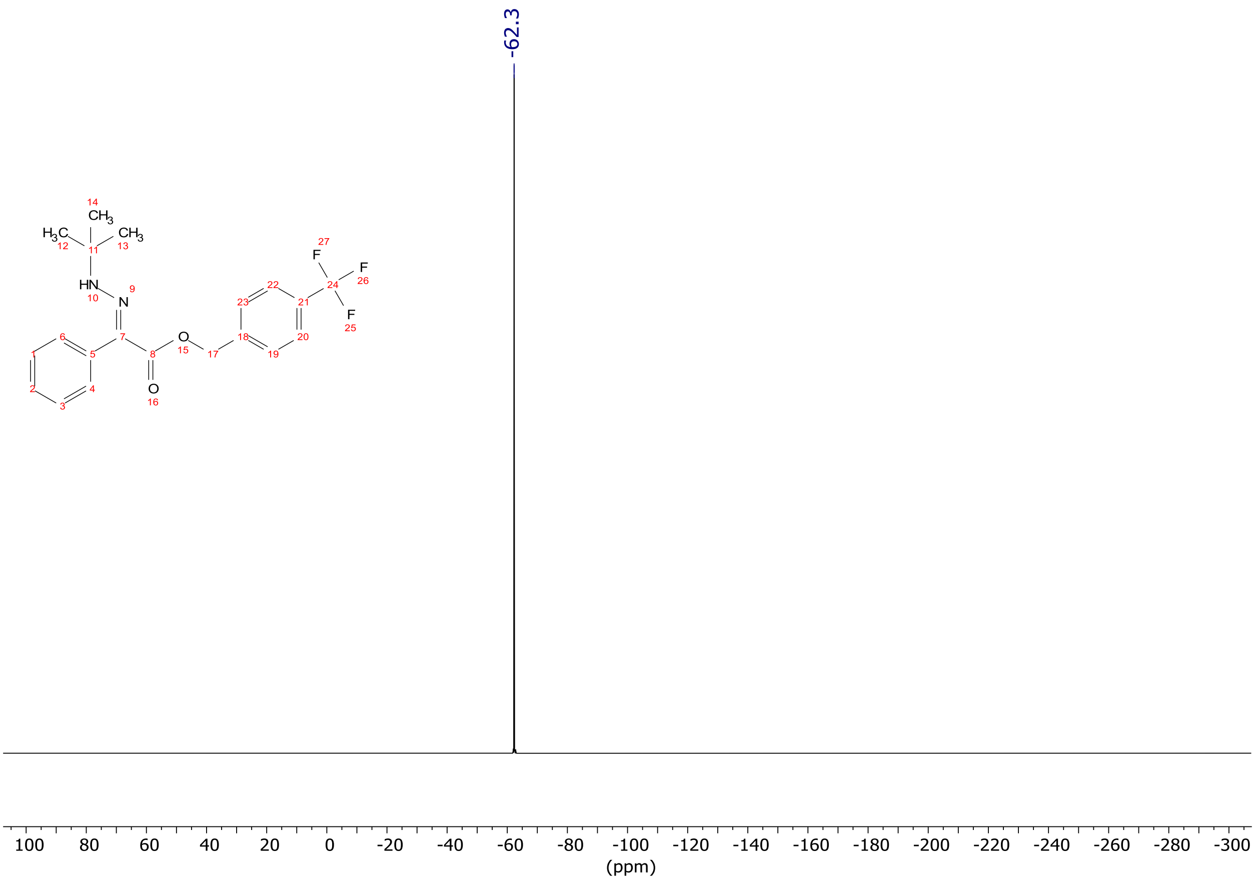

Figure S 128 ^{19}F NMR spectrum of 2.34

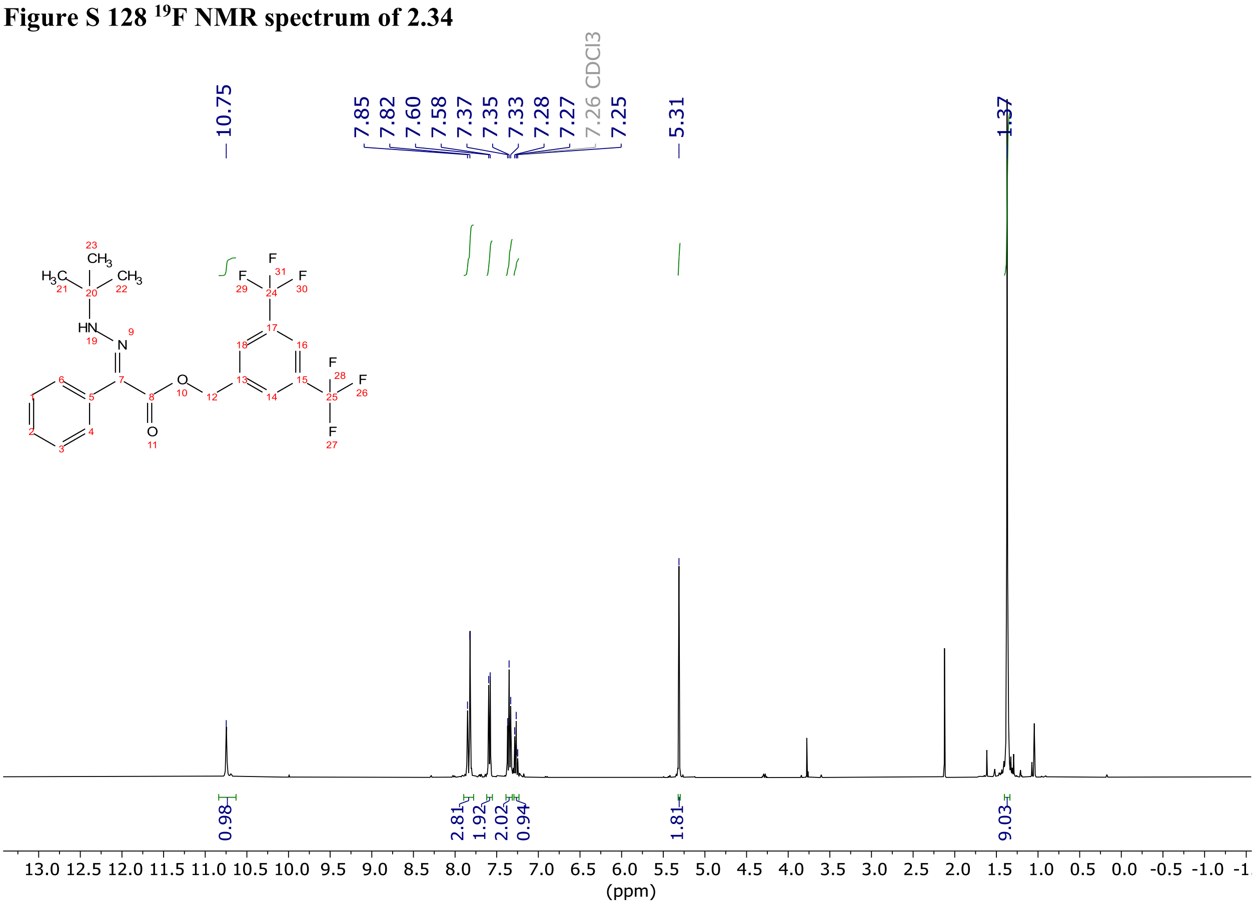

Figure S 129 ^{1}H NMR spectrum of 2.35

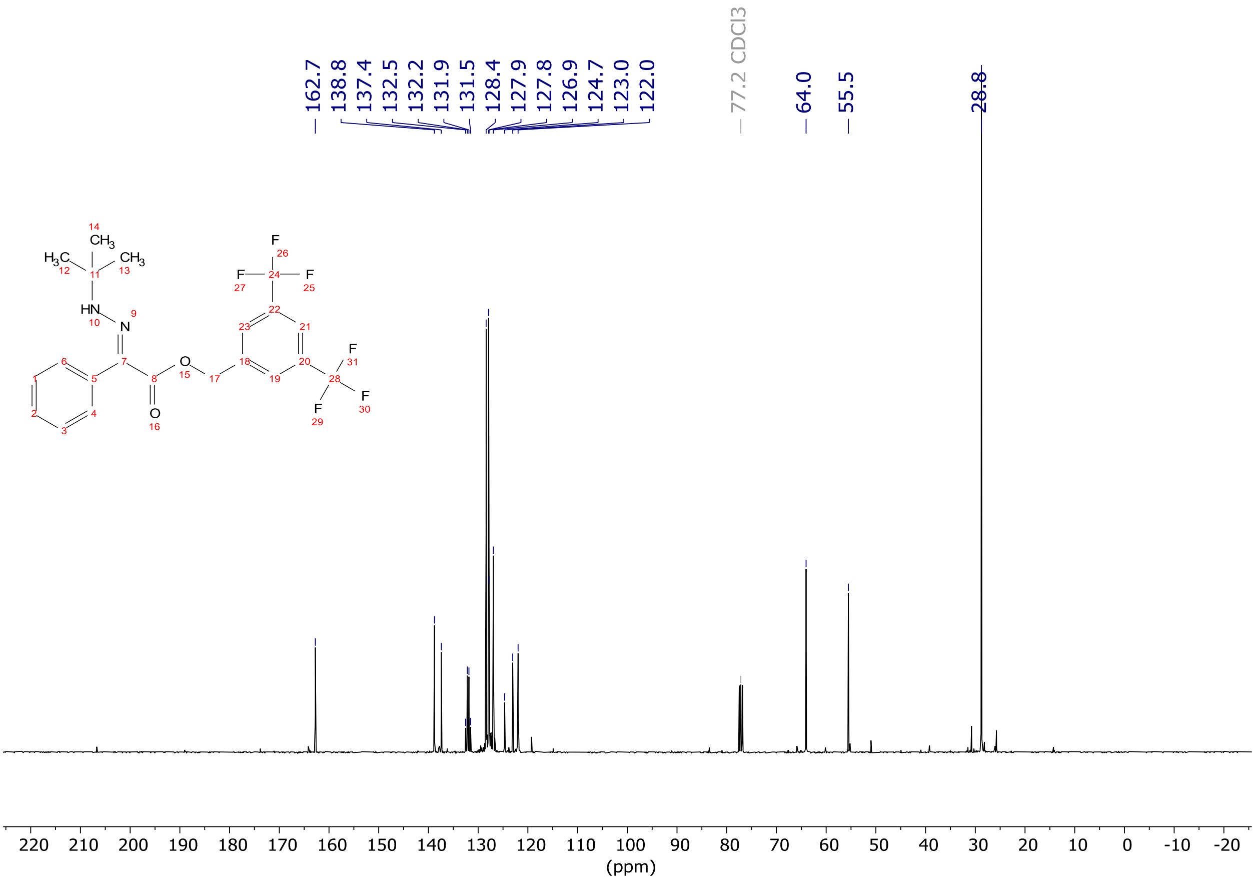

Figure S 130 ^{13}C NMR spectrum of 2.35

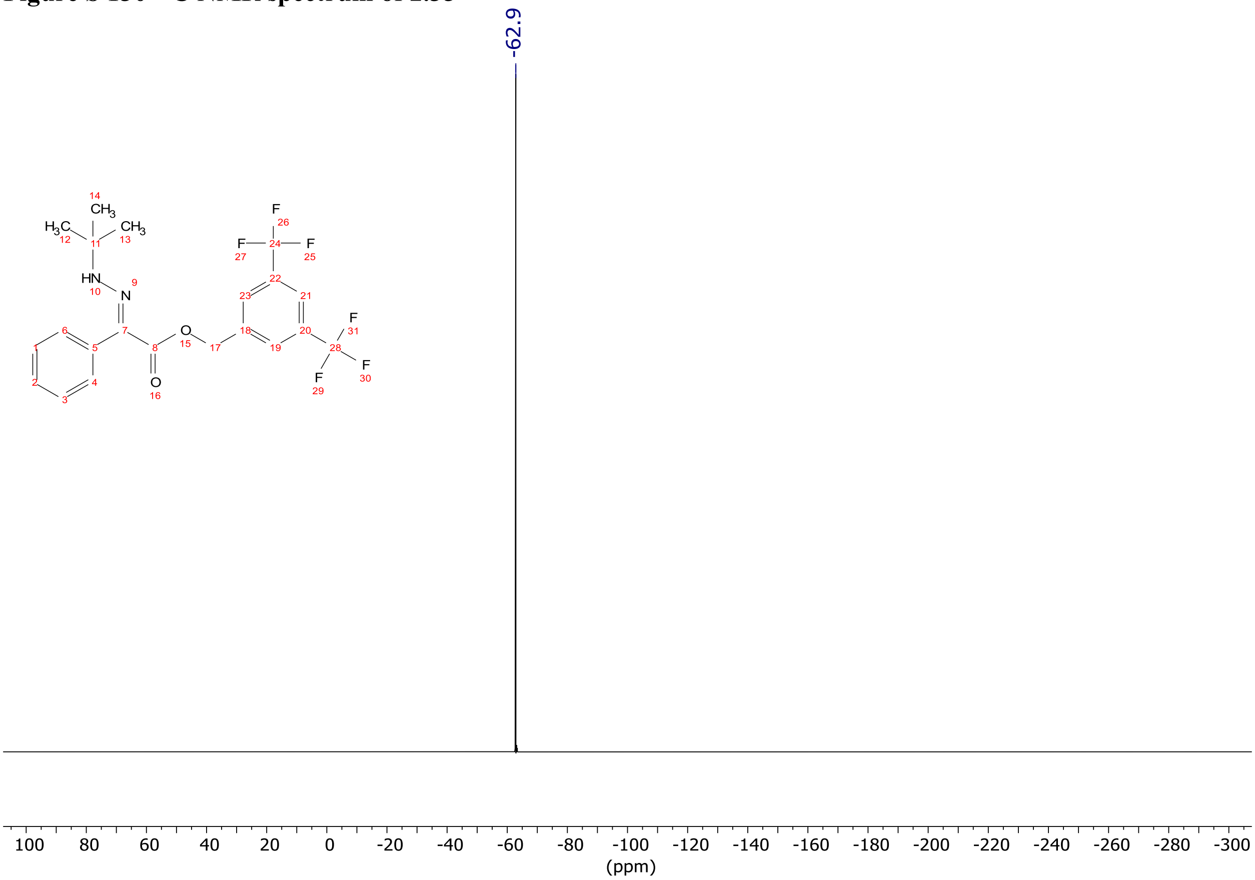

Figure S 131 ^{19}F NMR spectrum of 2.35

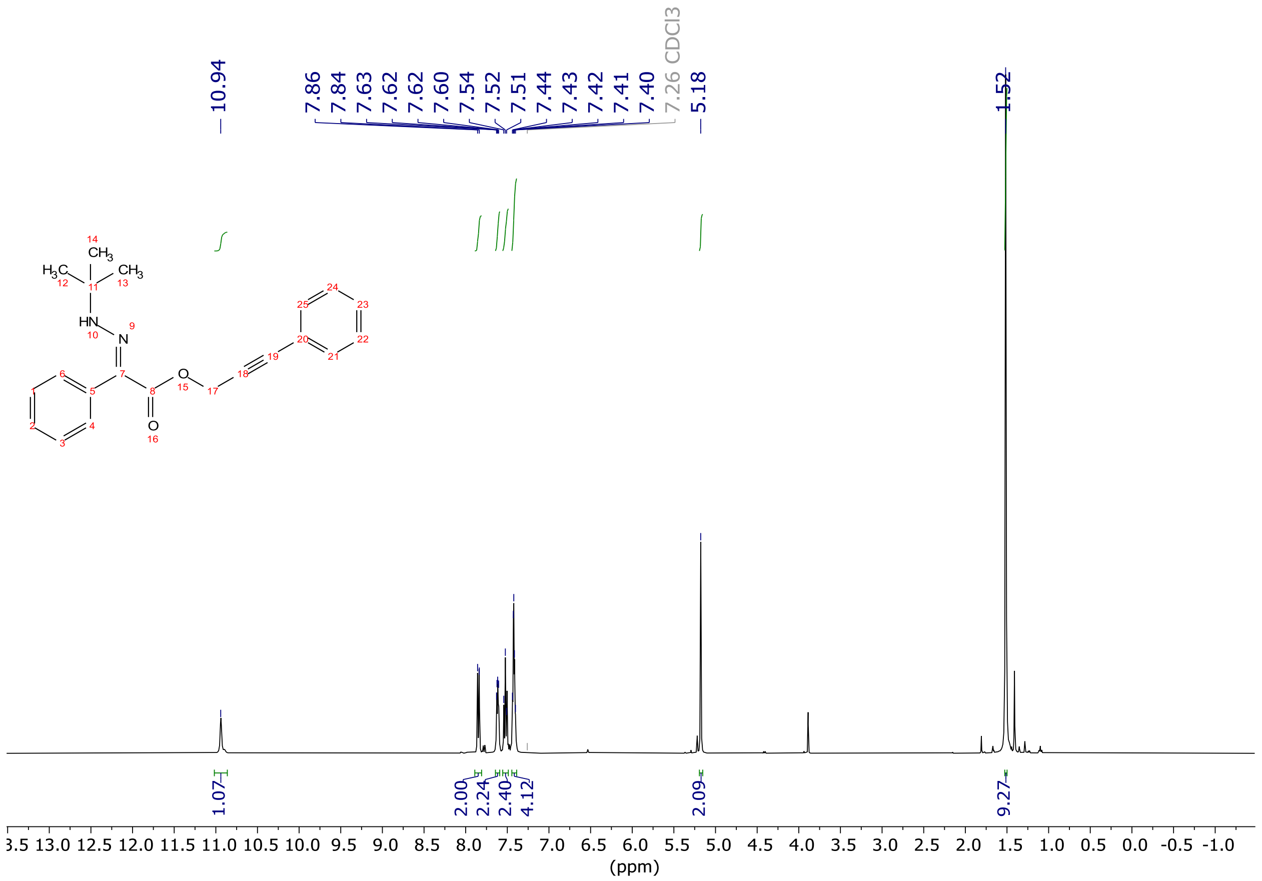

Figure S 132 ^{1}H NMR spectrum of 2.36

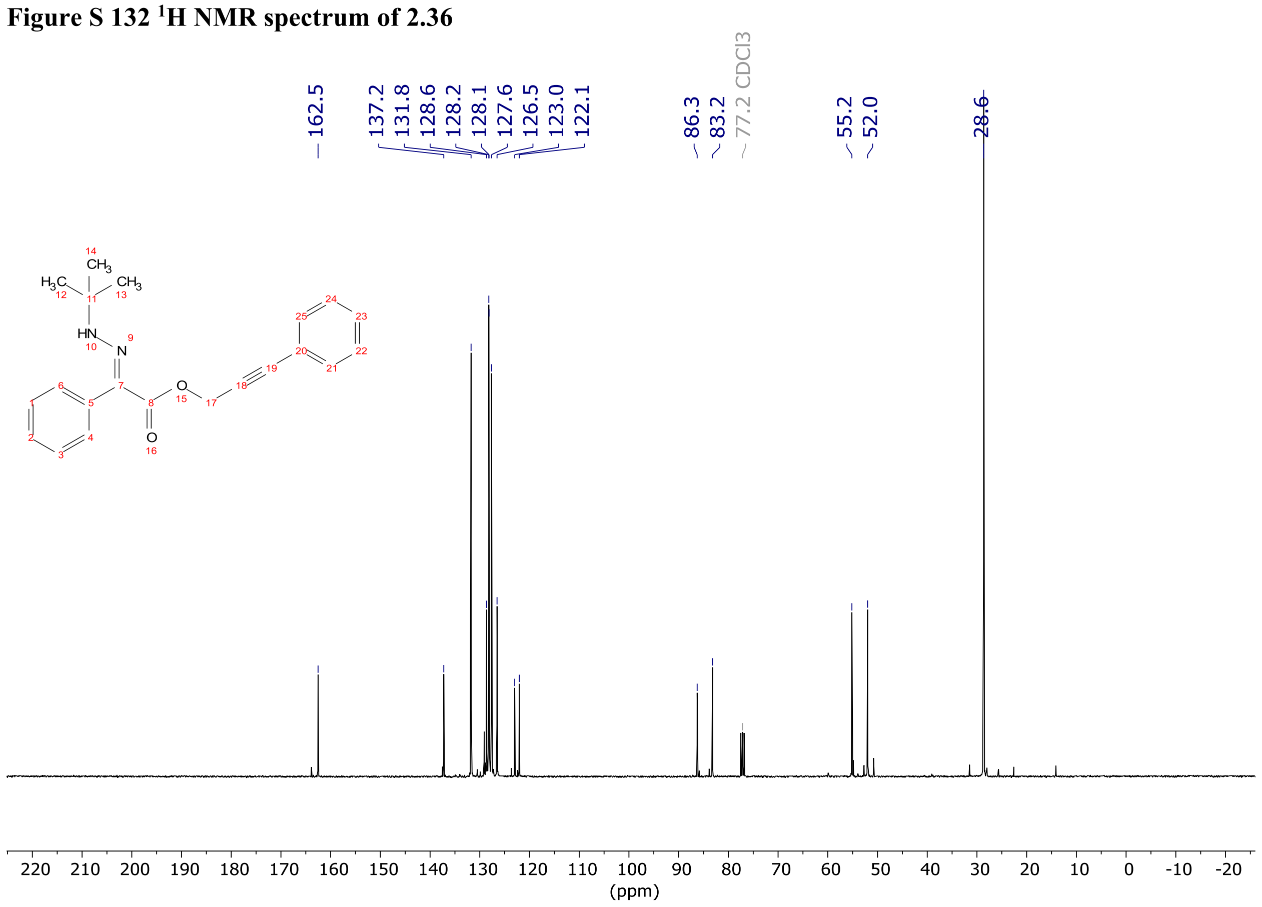

Figure S 133 ^{13}C NMR spectrum of 2.36

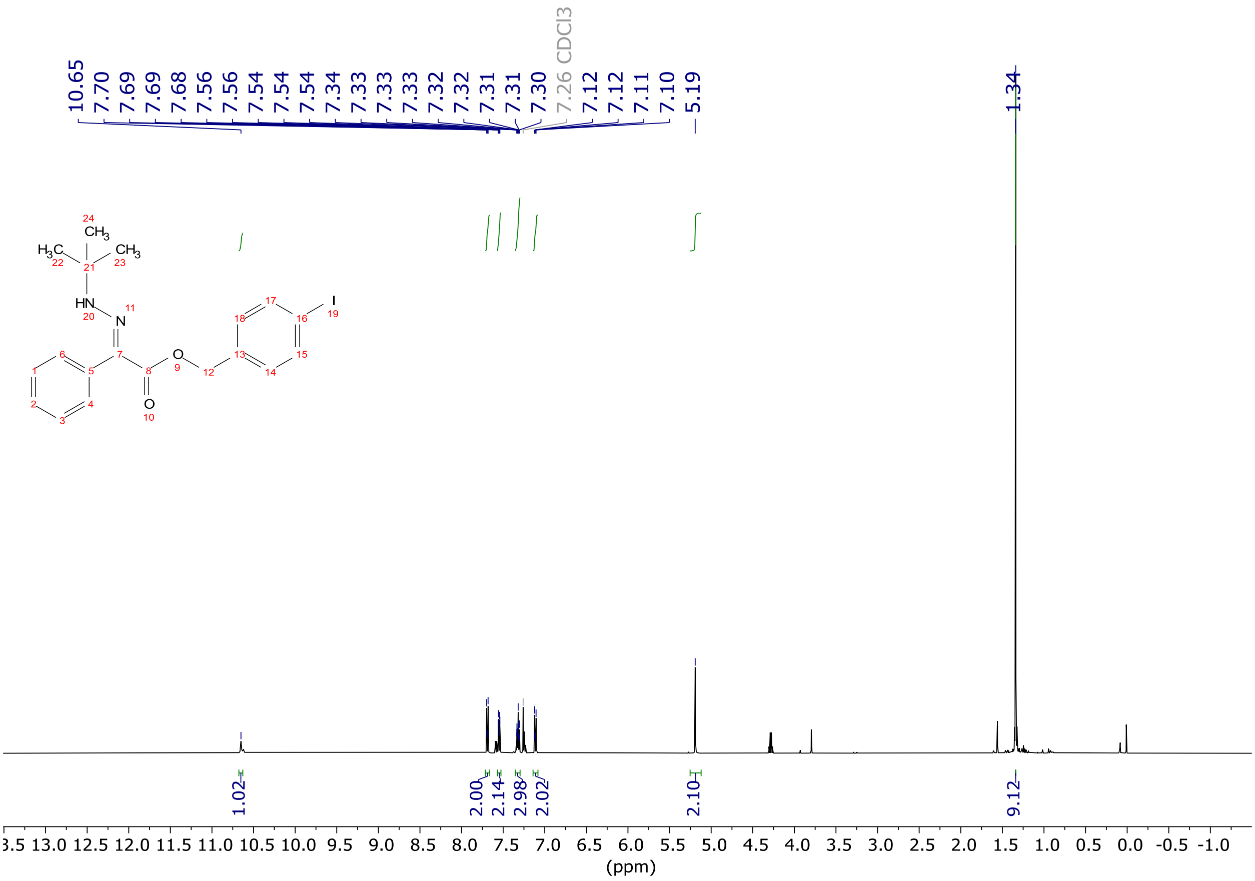

Figure S 134 ^{1}H NMR spectrum of 2.37

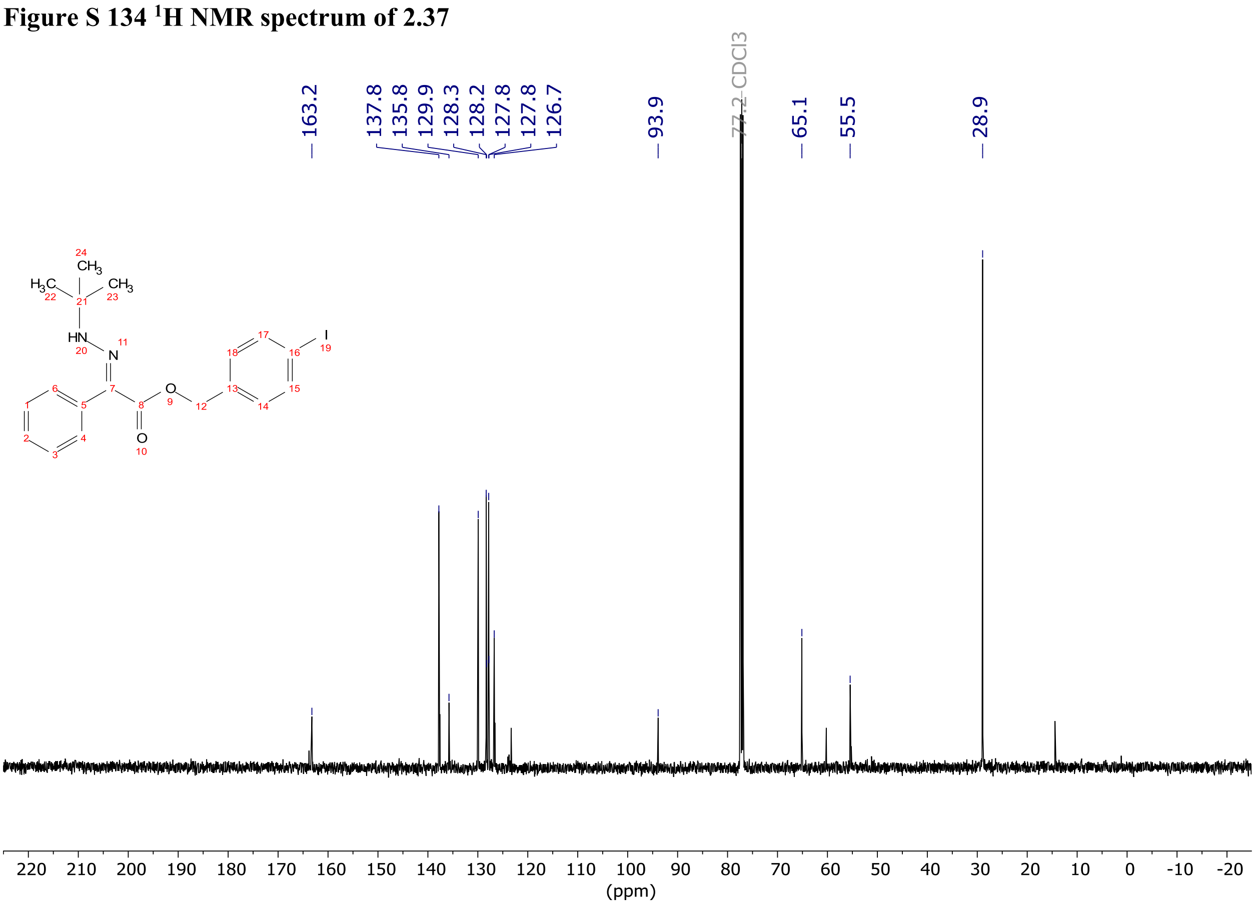

Figure S 135 ^{13}C NMR spectrum of 2.37

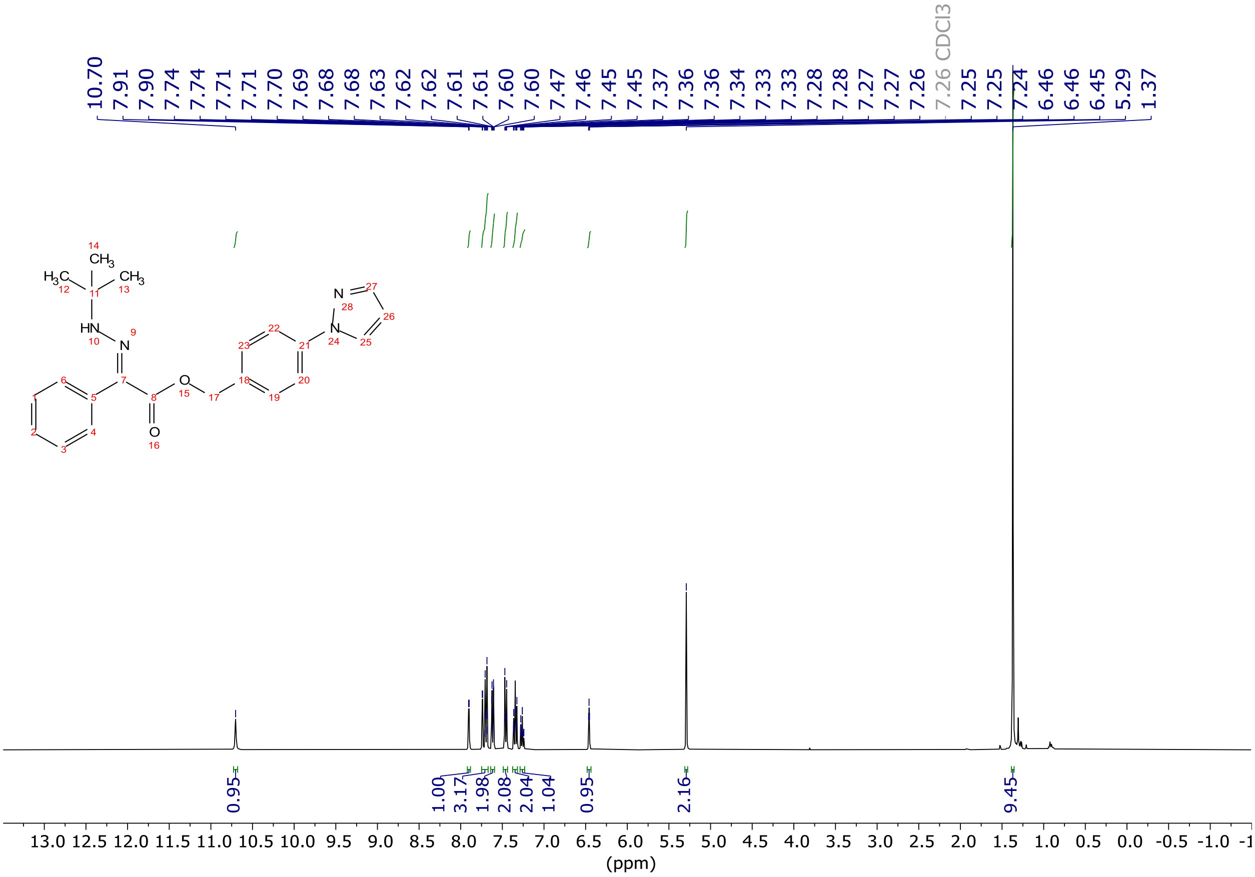

Figure S 136 ^{1}H NMR spectrum of 2.38

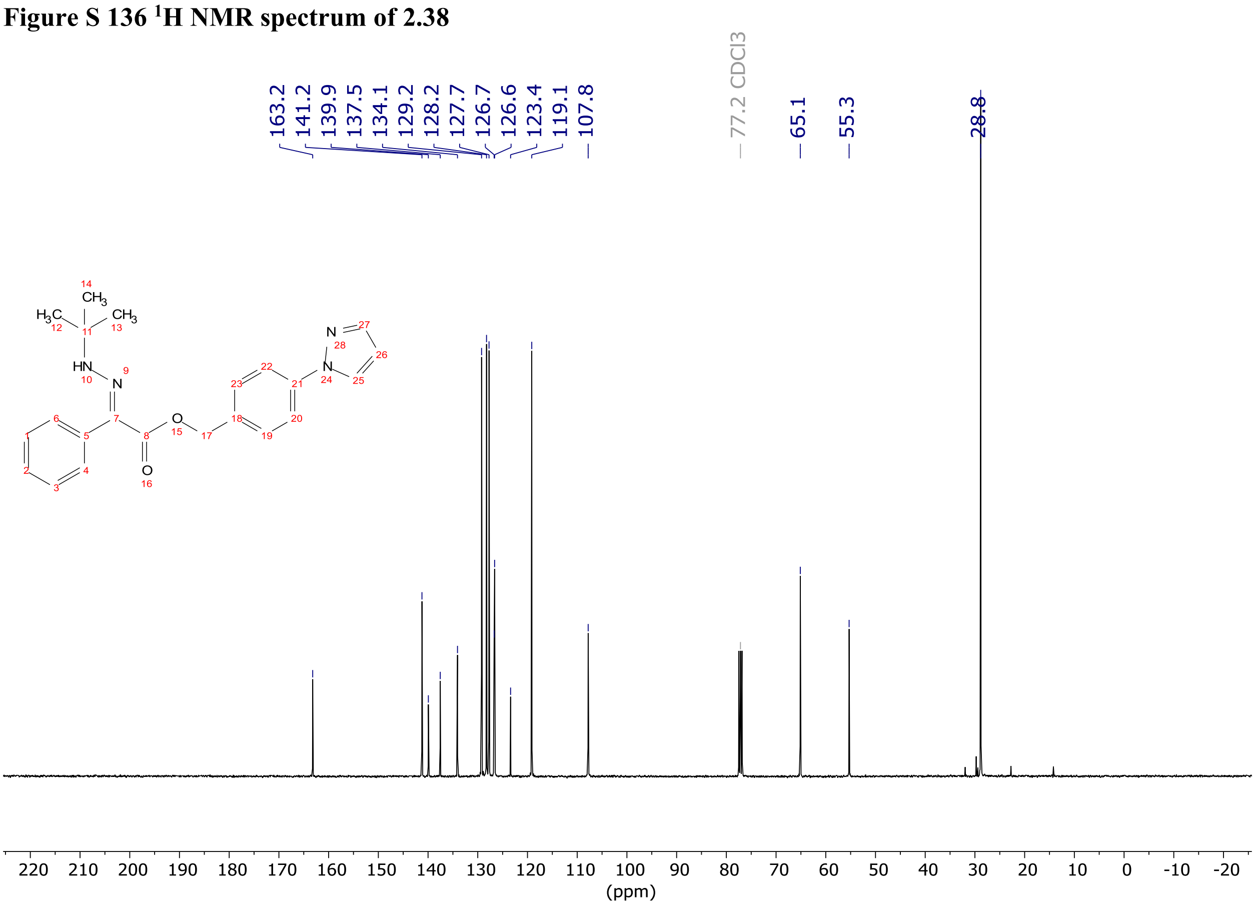

Figure S 137 ^{13}C NMR spectrum of 2.38

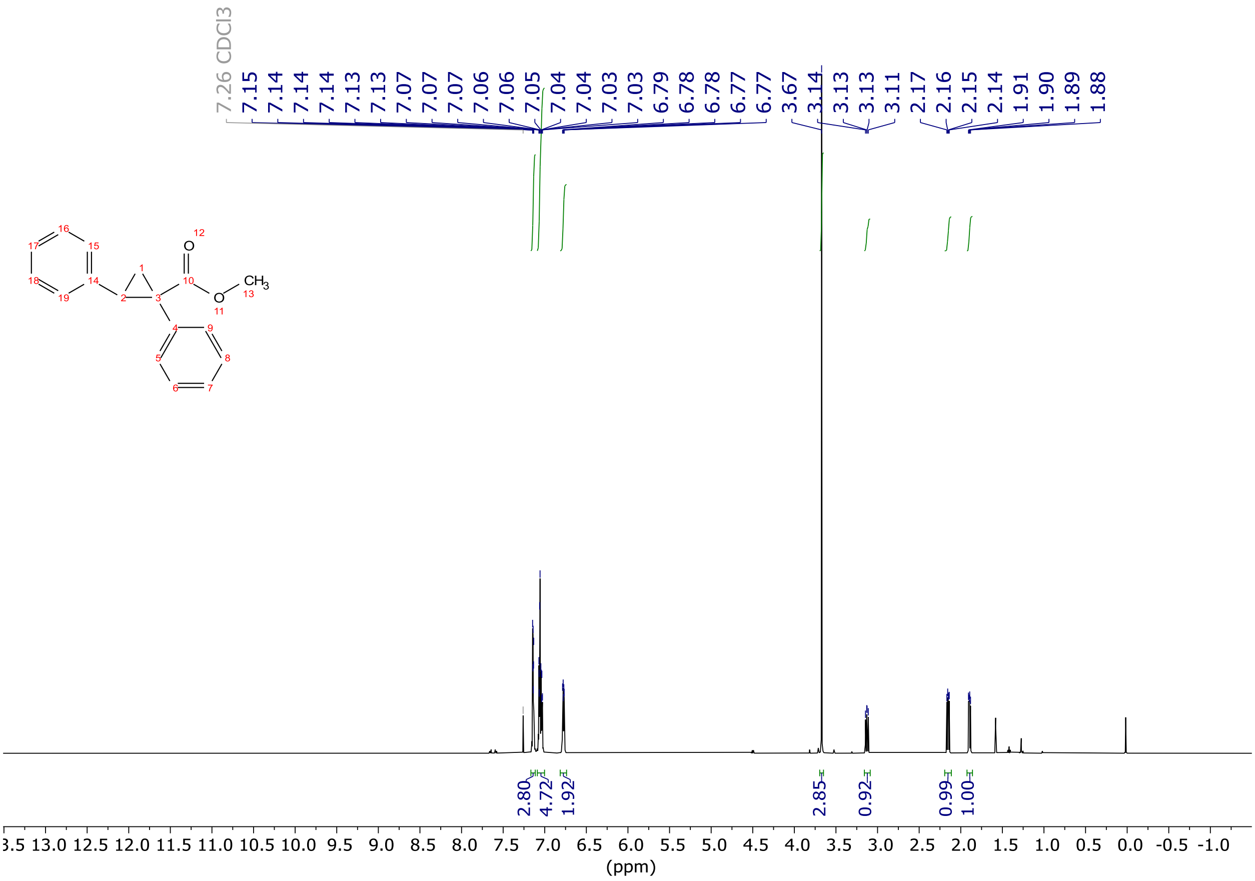

Figure S 138 ^{1}H NMR spectrum of 3.1

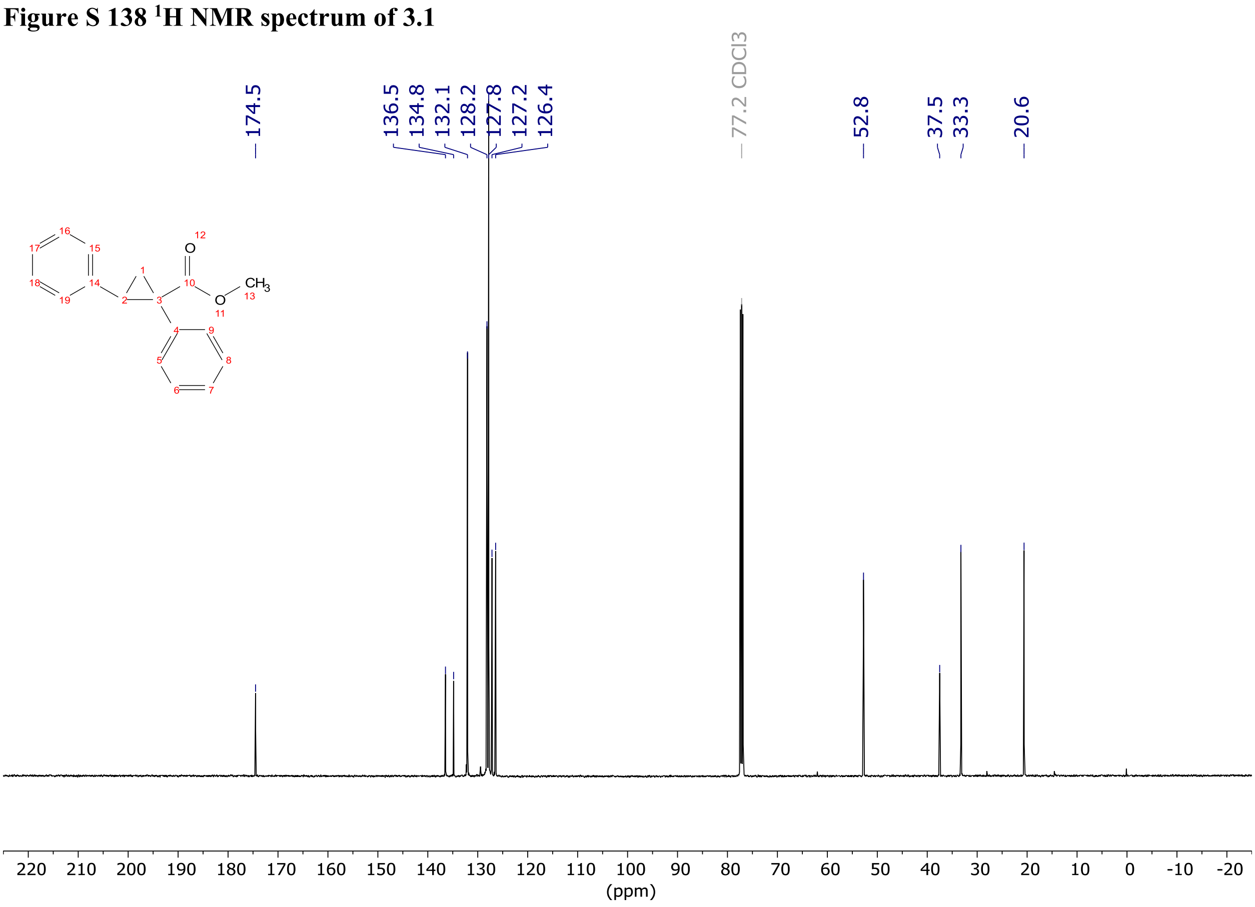

Figure S 139 ^{13}C NMR spectrum of 3.1

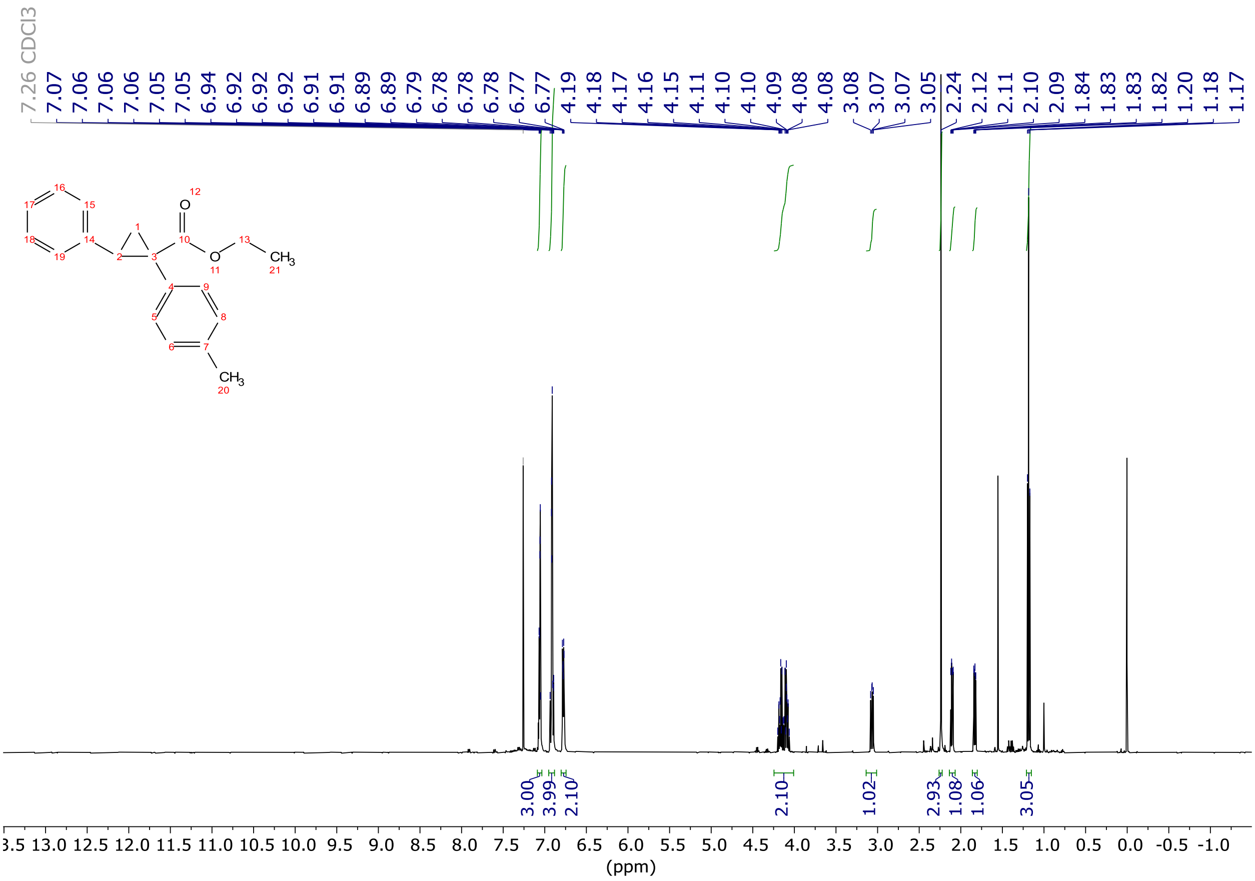

Figure S 140 ^{1}H NMR spectrum of 3.2

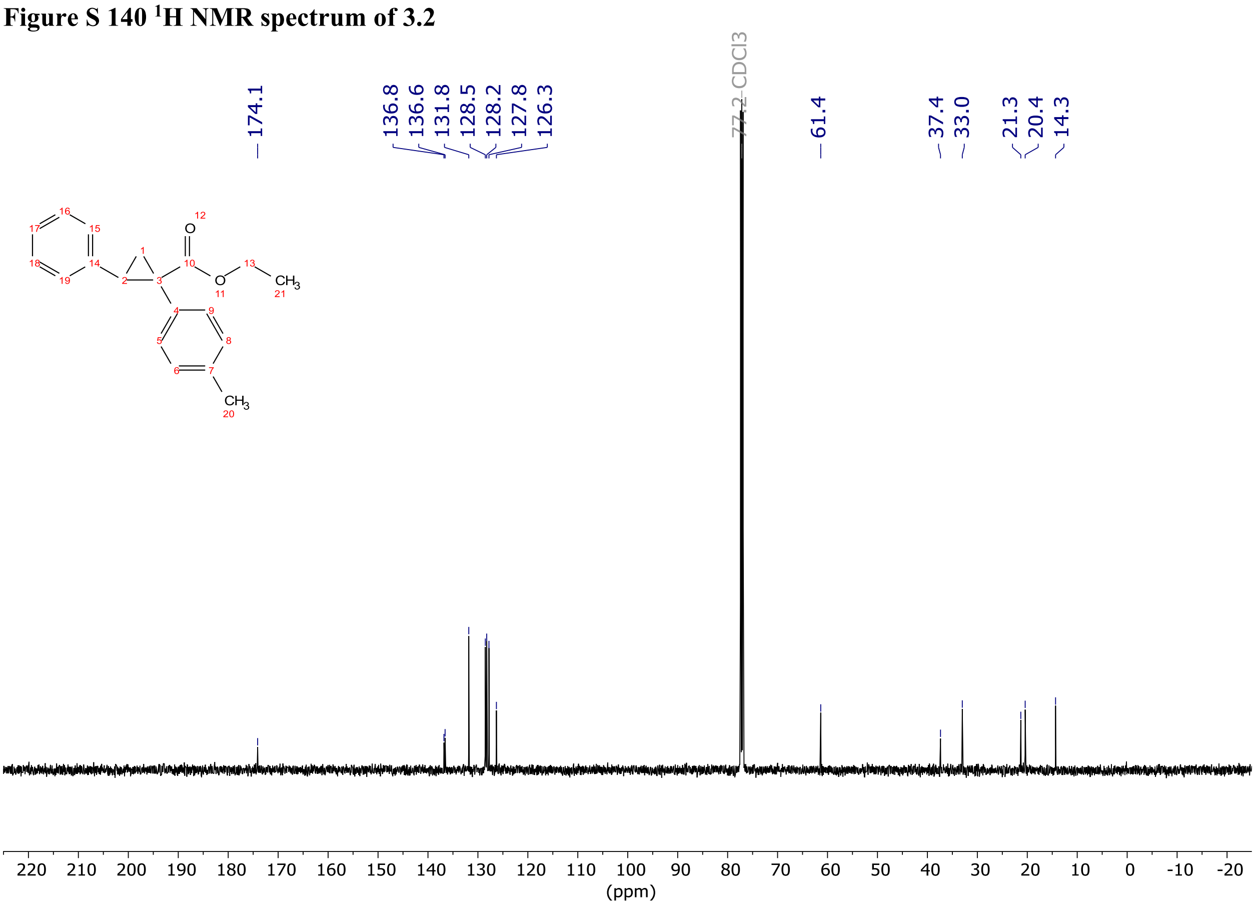

Figure S 141 ^{13}C NMR spectrum of 3.2

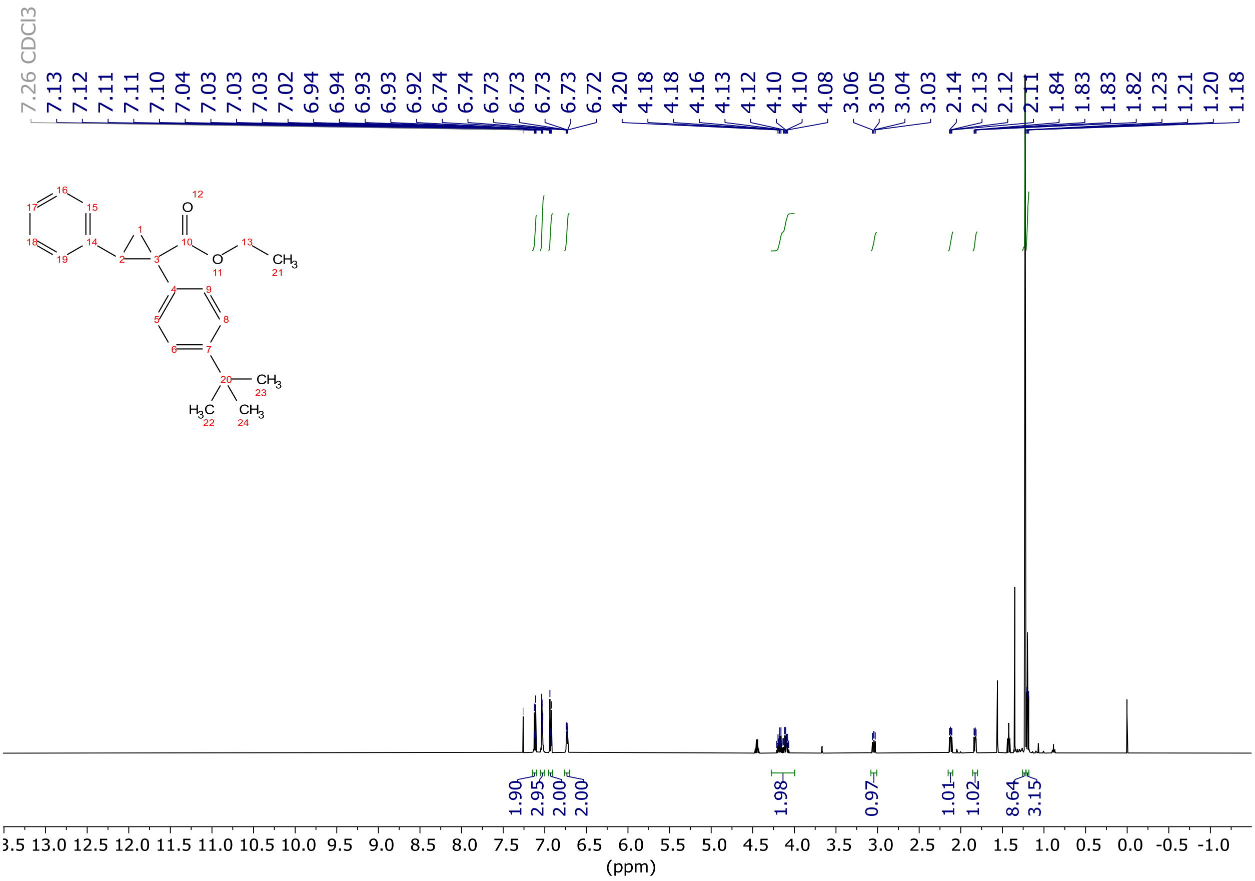

Figure S 142 ^{1}H NMR spectrum of 3.3

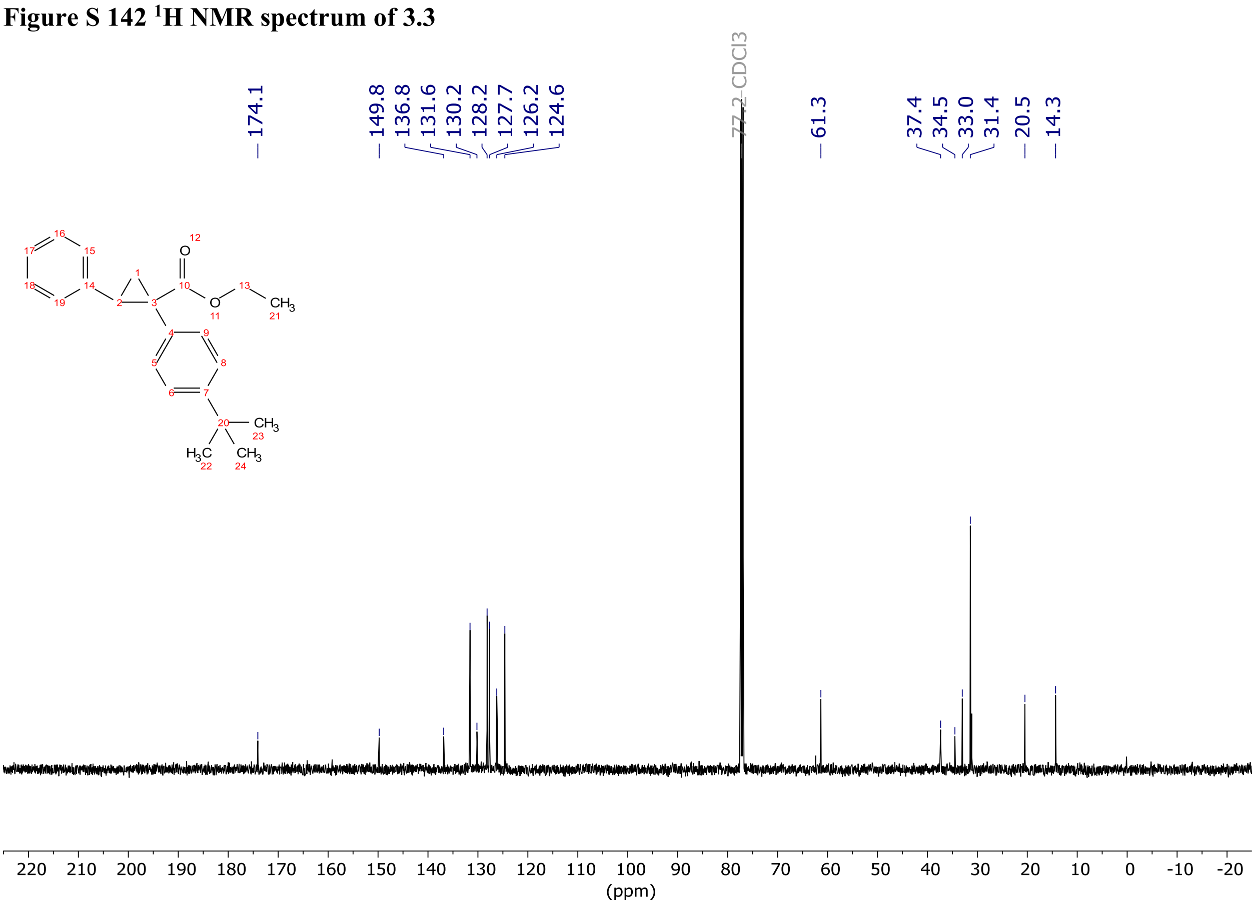

Figure S 143 ^{13}C NMR spectrum of 3.3

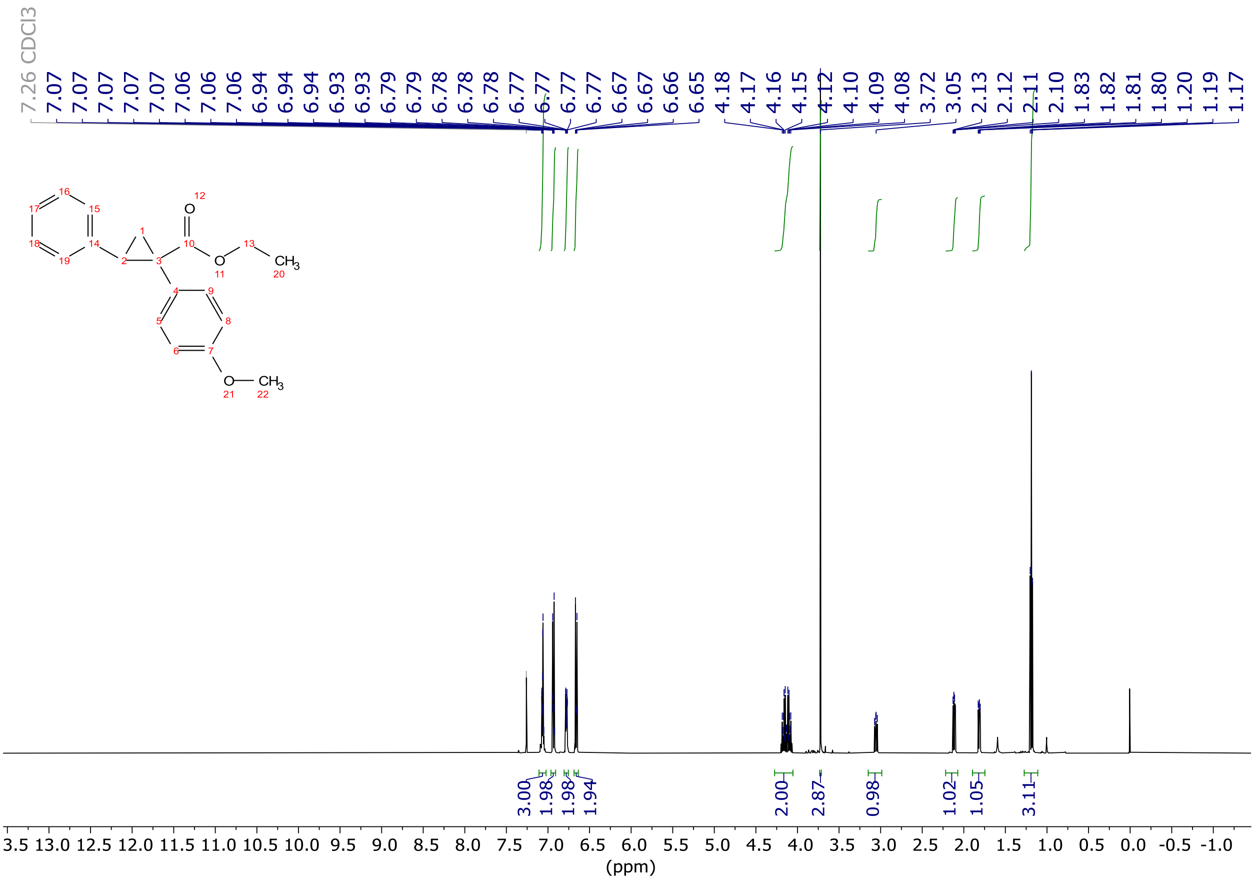

Figure S 144 ^{1}H NMR spectrum of 3.4

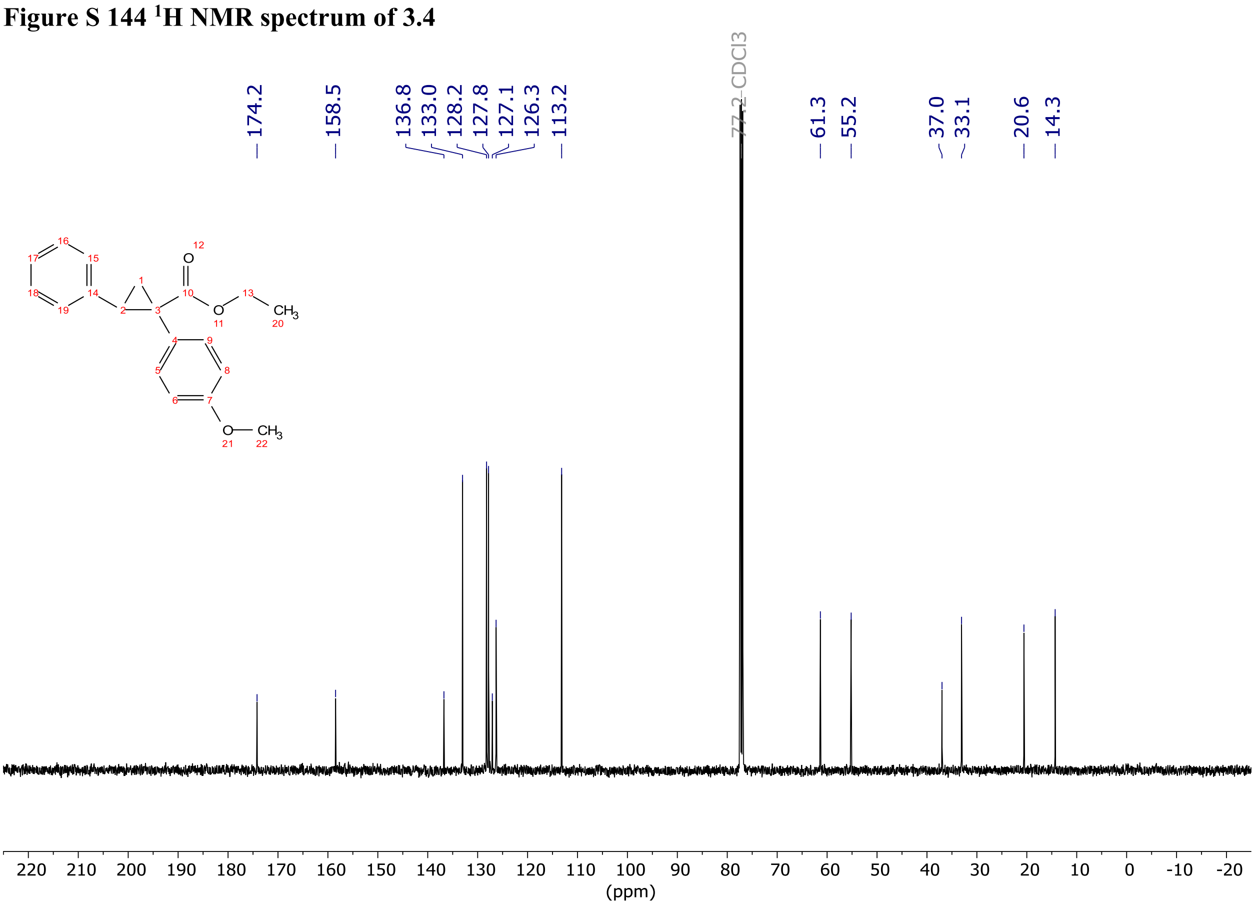

Figure S 145 ^{13}C NMR spectrum of 3.4

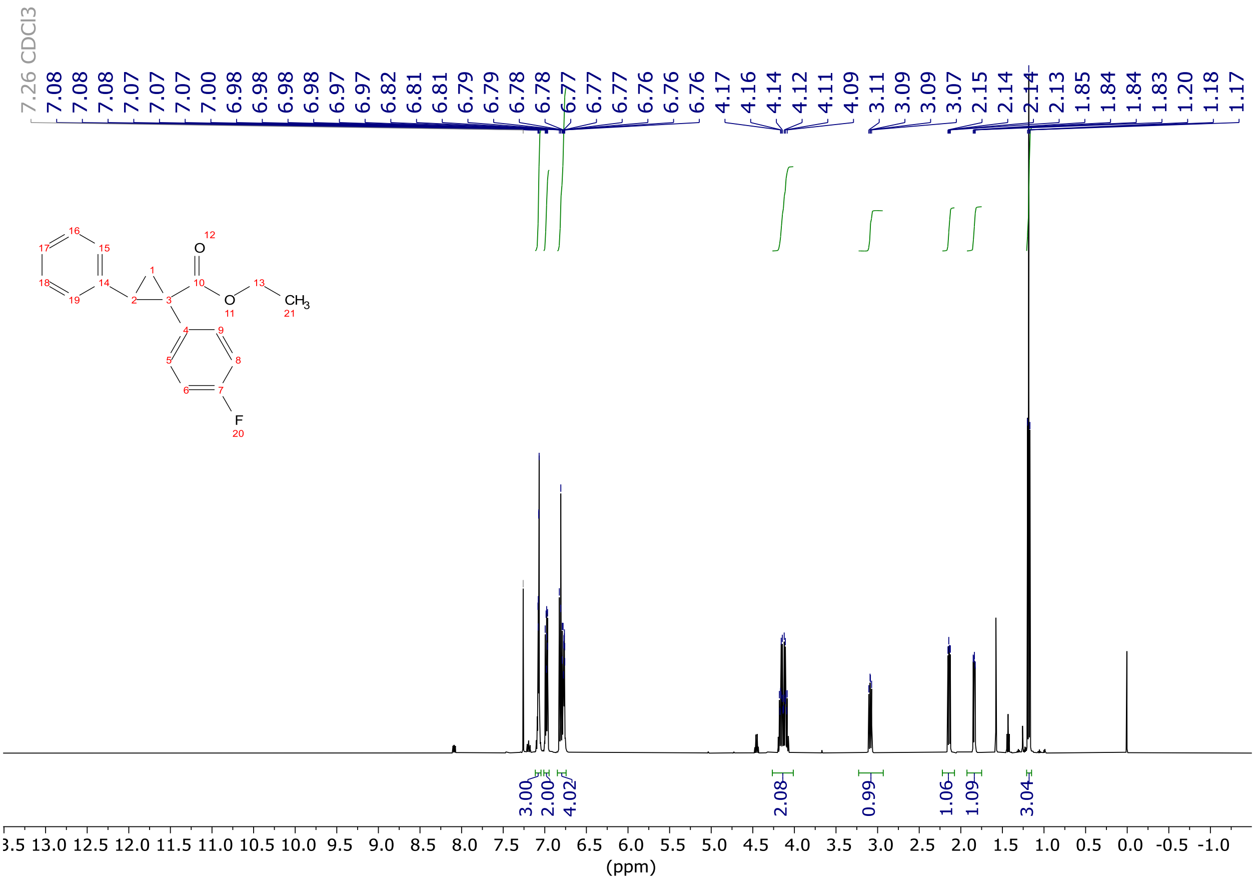

Figure S 146 ^{1}H NMR spectrum of 3.5

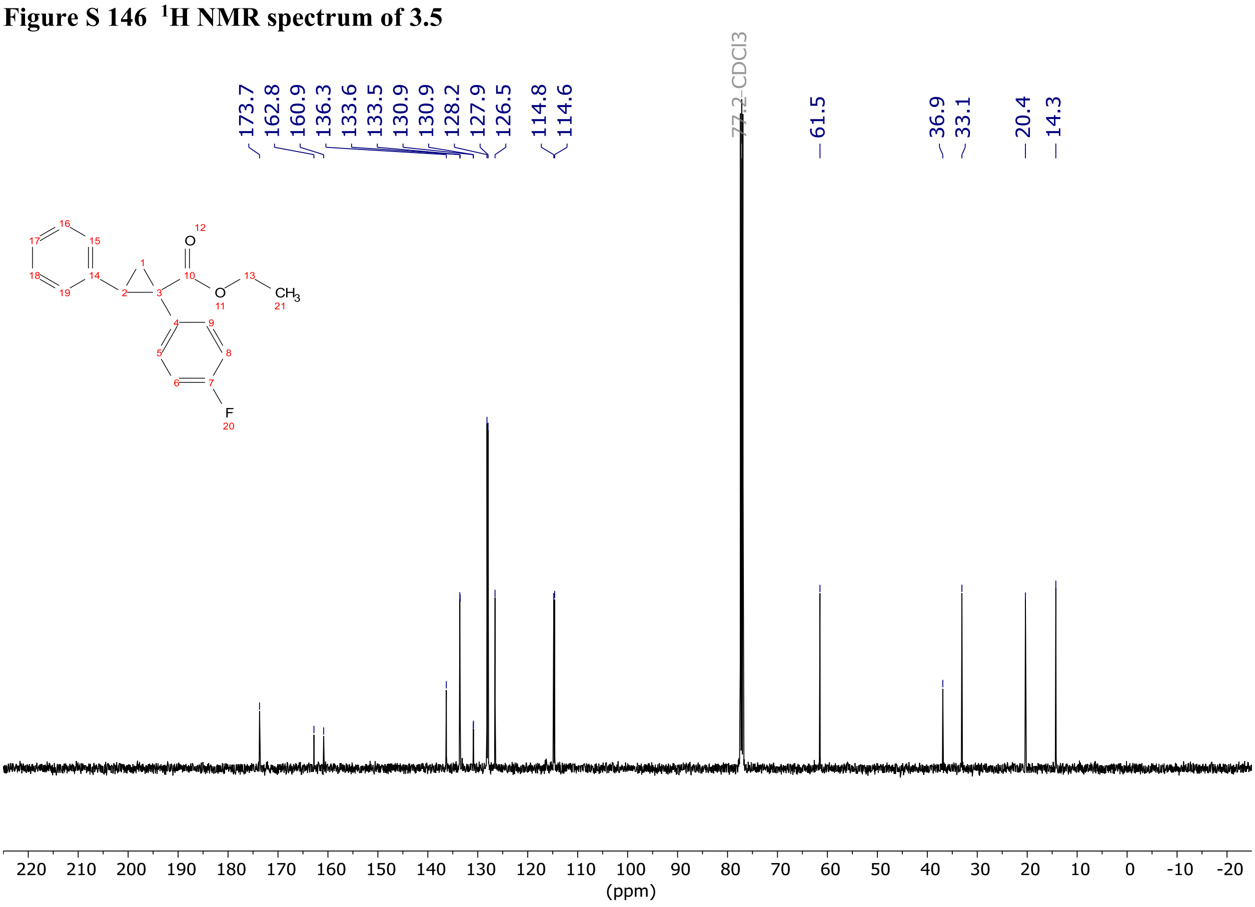

Figure S 147 ^{13}C NMR spectrum of 3.5

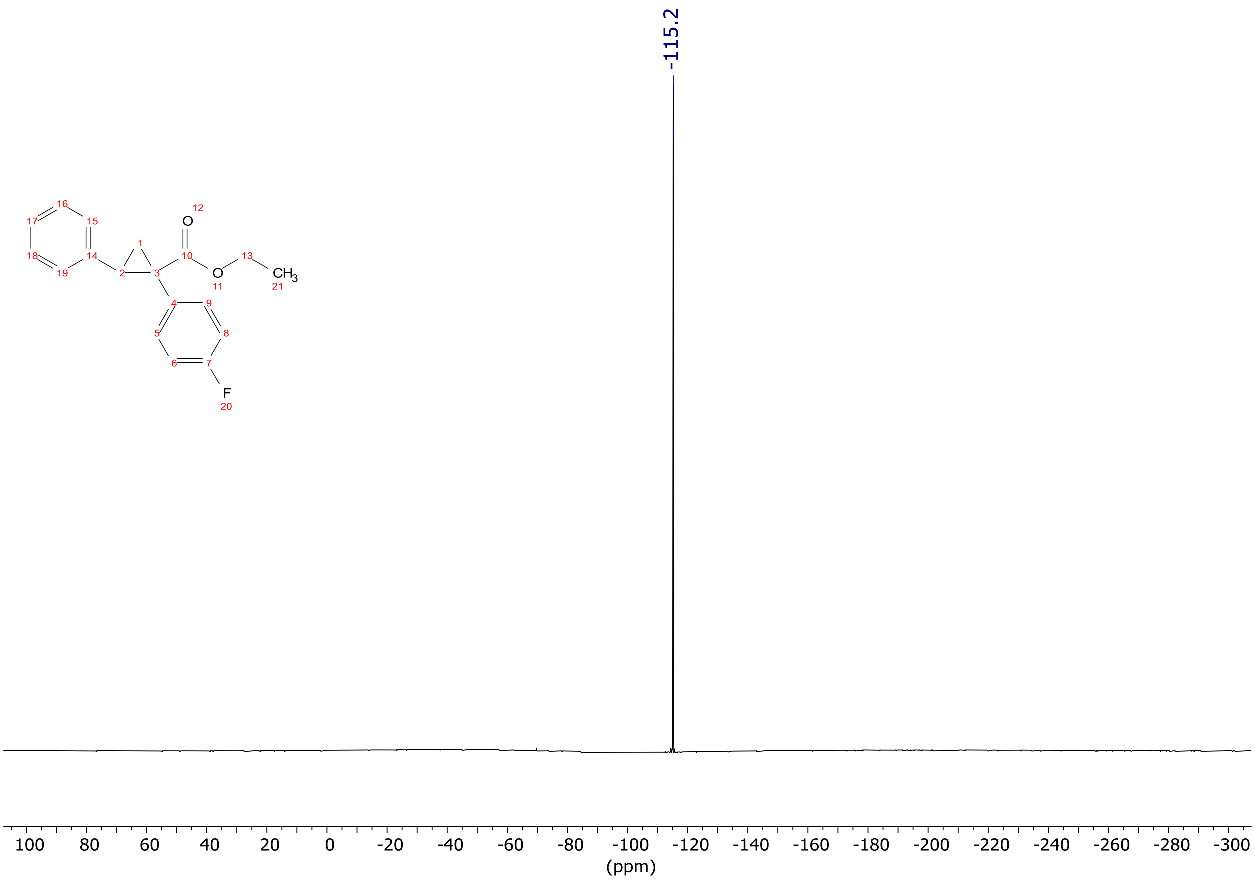

Figure S 148 ^{19}F NMR spectrum of 3.5

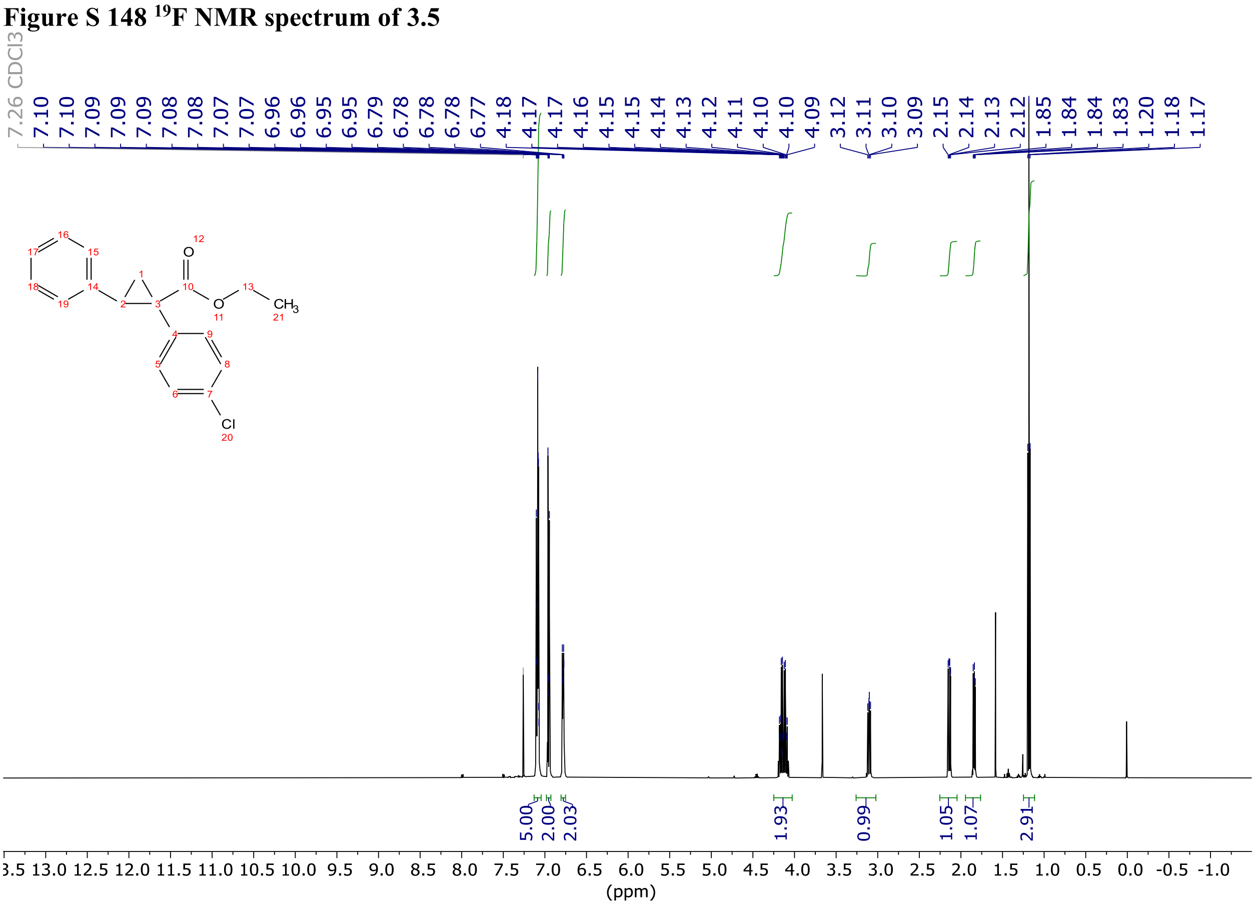

Figure S 149 ^{1}H NMR spectrum of 3.6

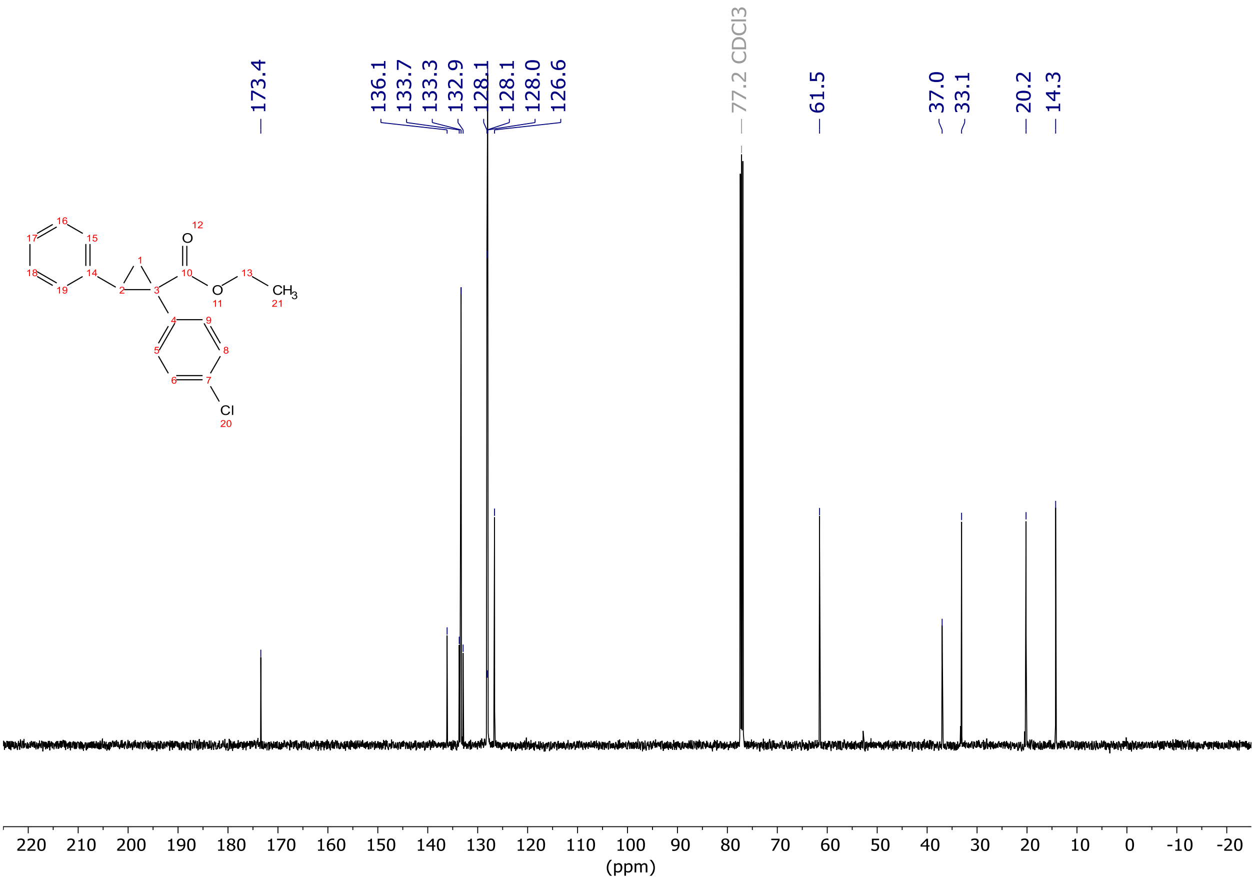

Figure S 150 ^{13}C NMR spectrum of 3.6

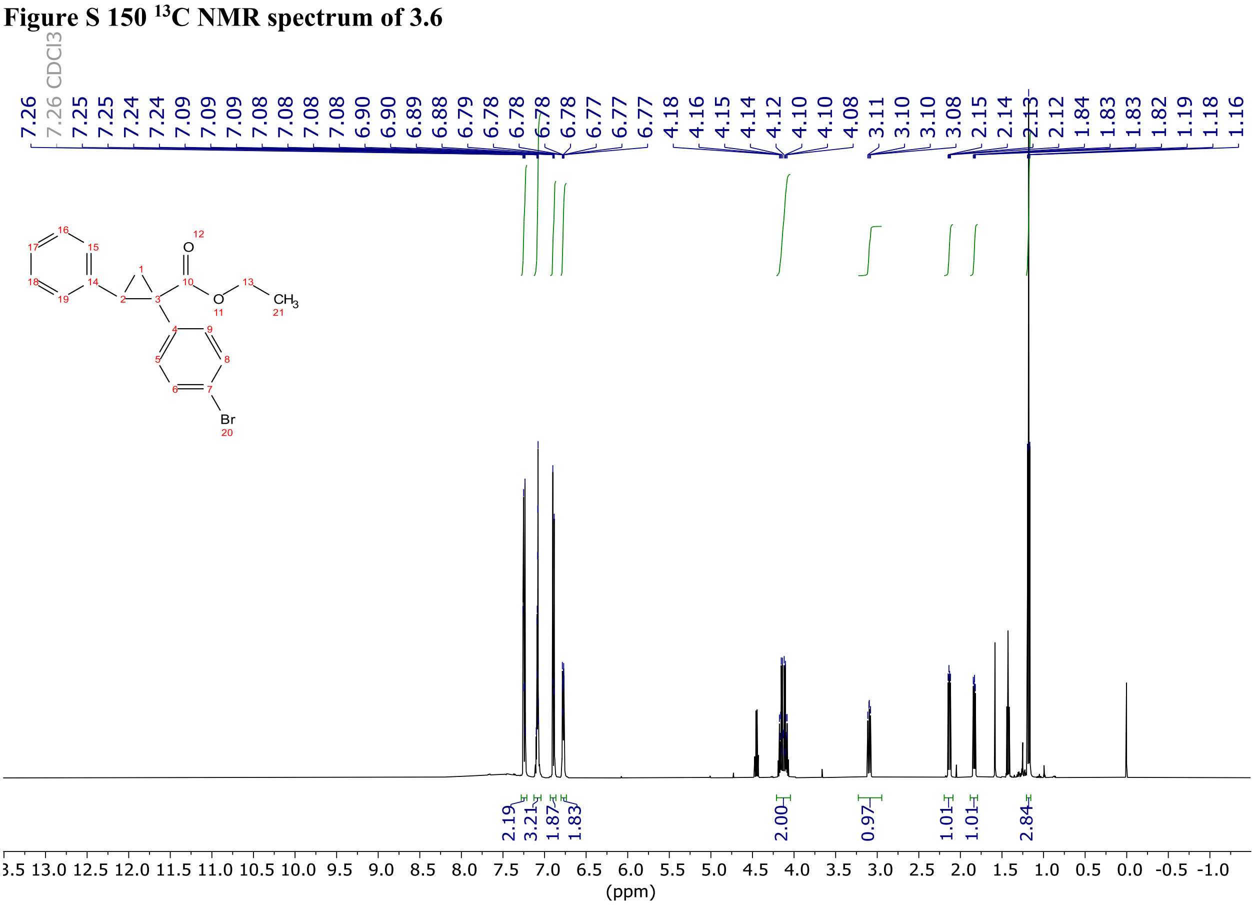

Figure S 151 ^{1}H NMR spectrum of 3.7

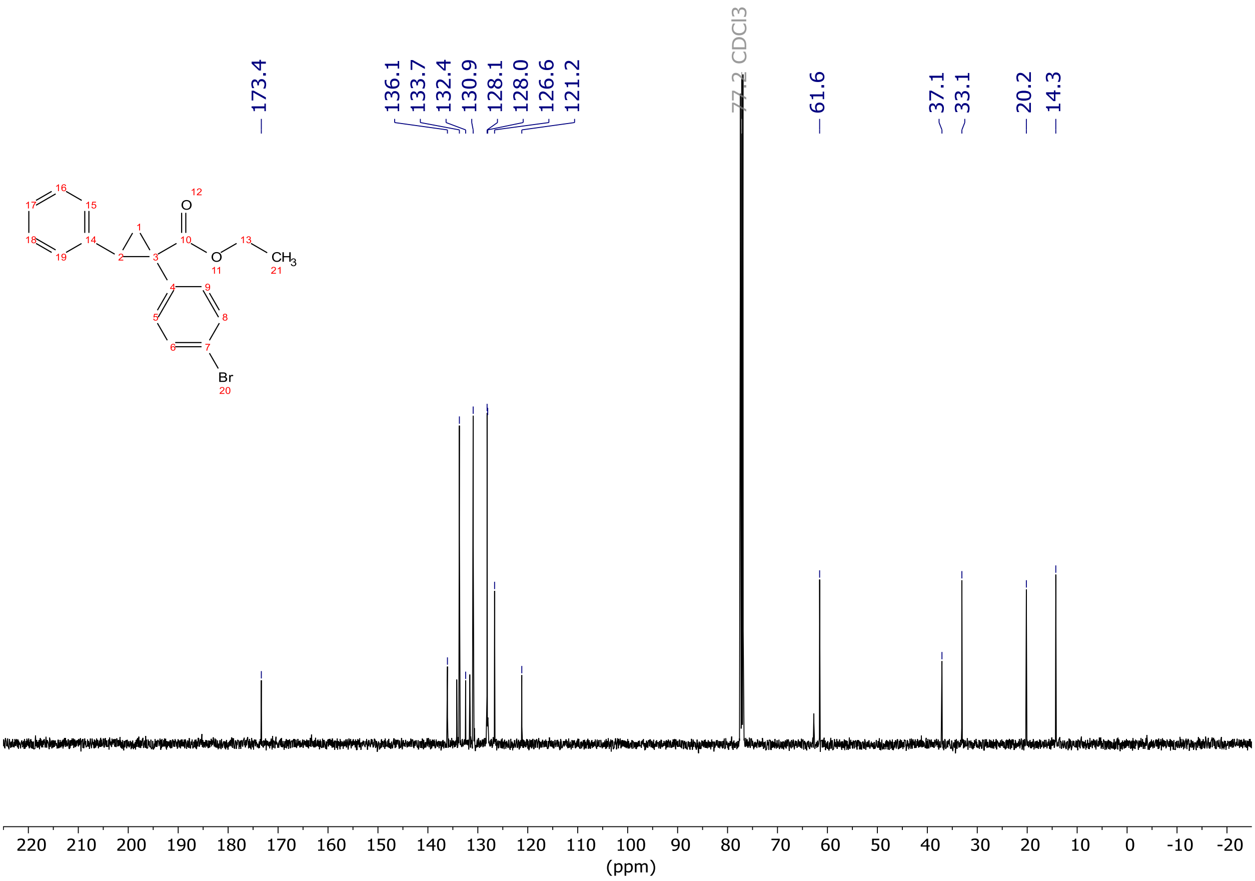

Figure S 152 ^{13}C NMR spectrum of 3.7

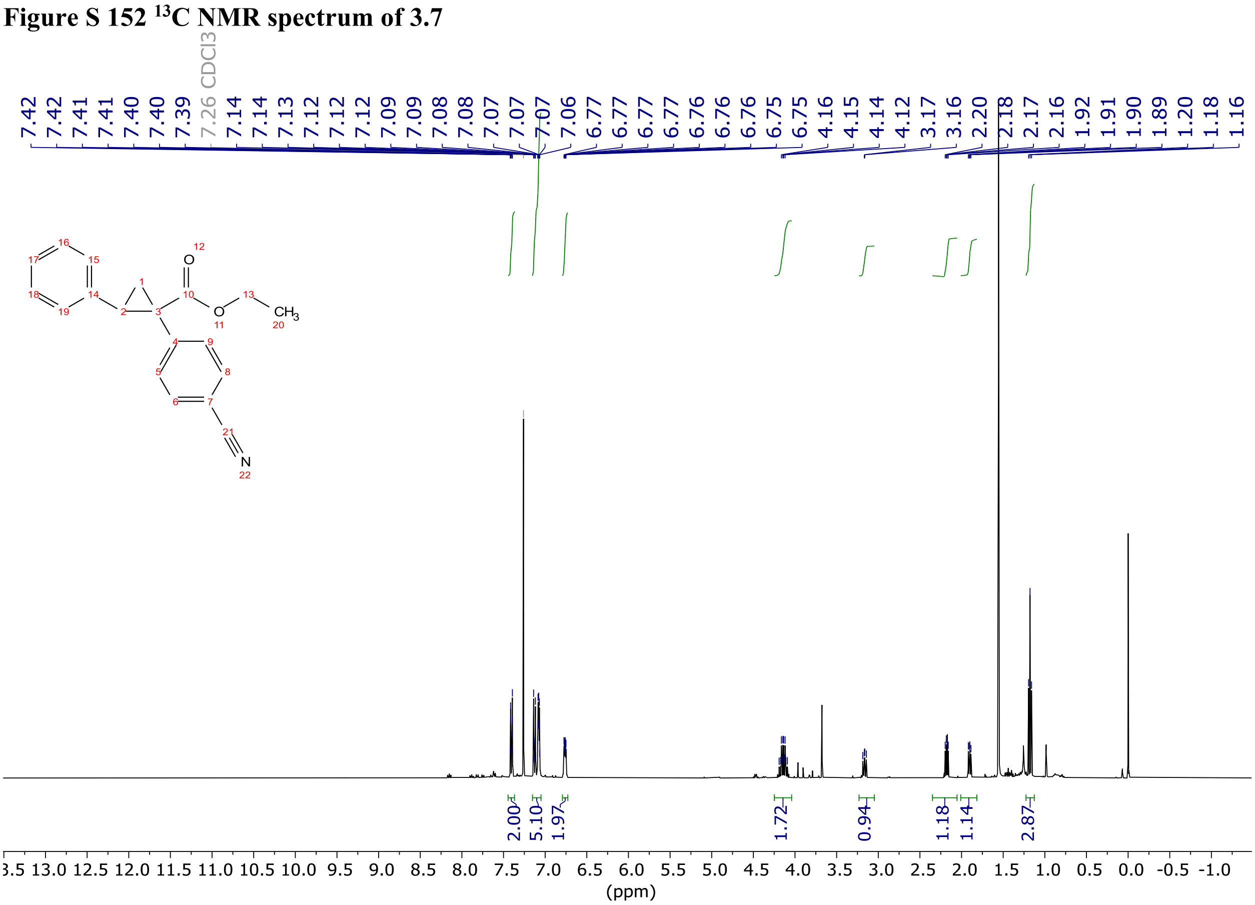

Figure S 153 ^{1}H NMR spectrum of 3.8

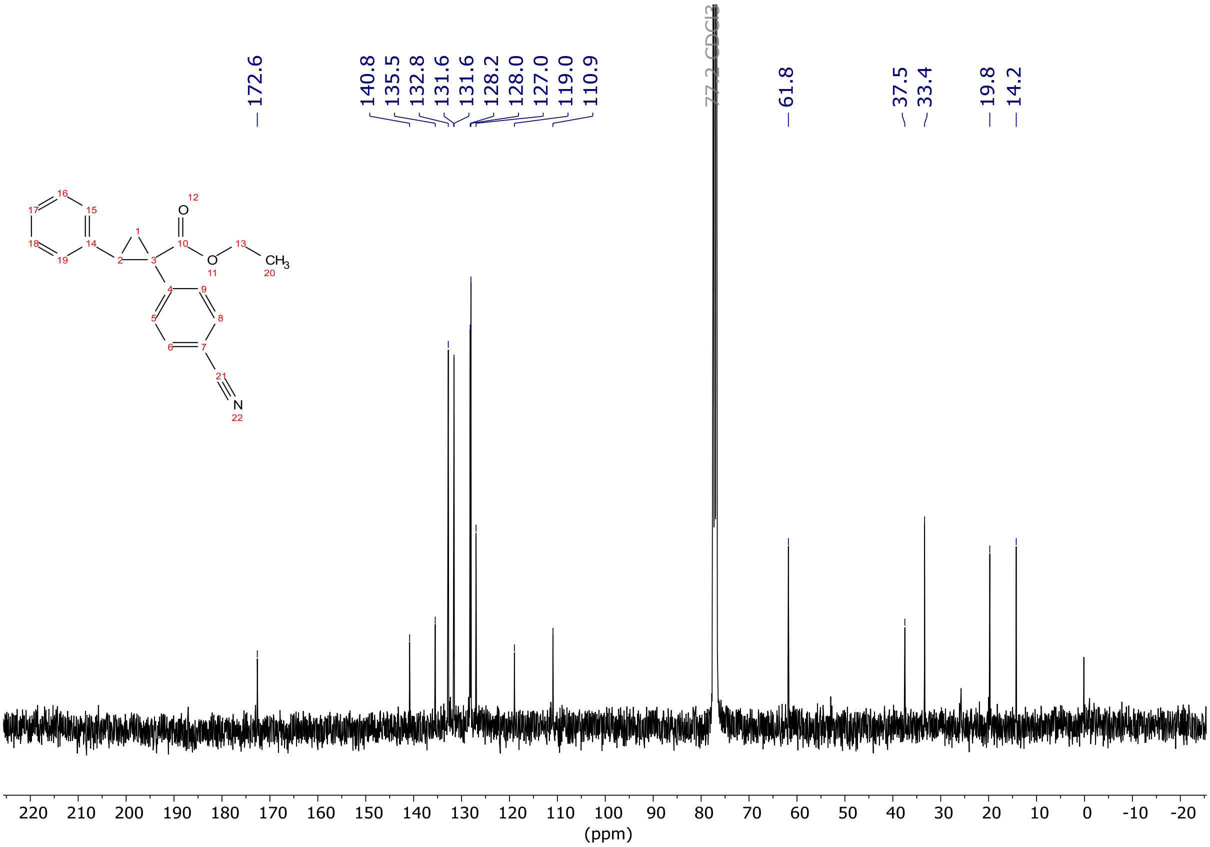

Figure S 154 13C NMR spectrum of 3.8

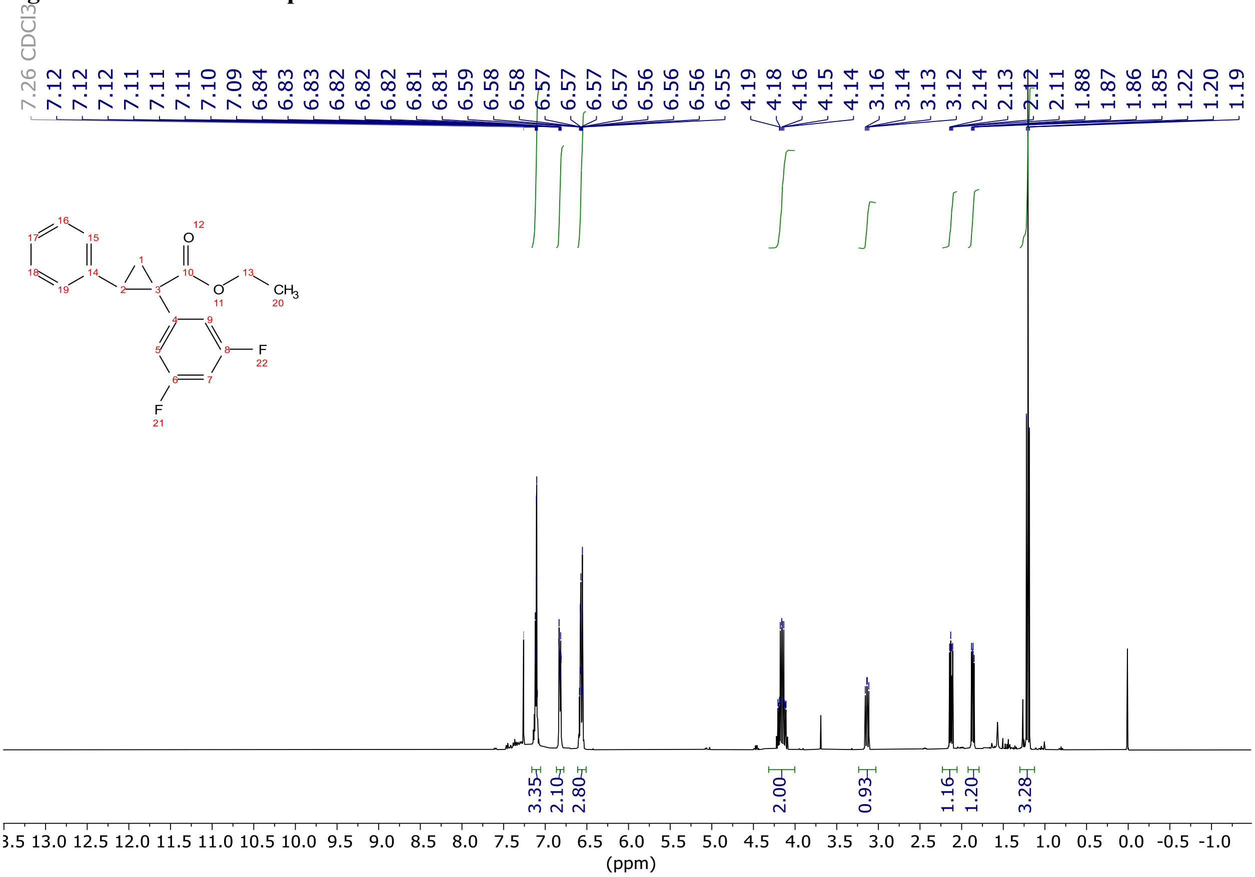

Figure S 155 ^{1}H NMR spectrum of 3.9

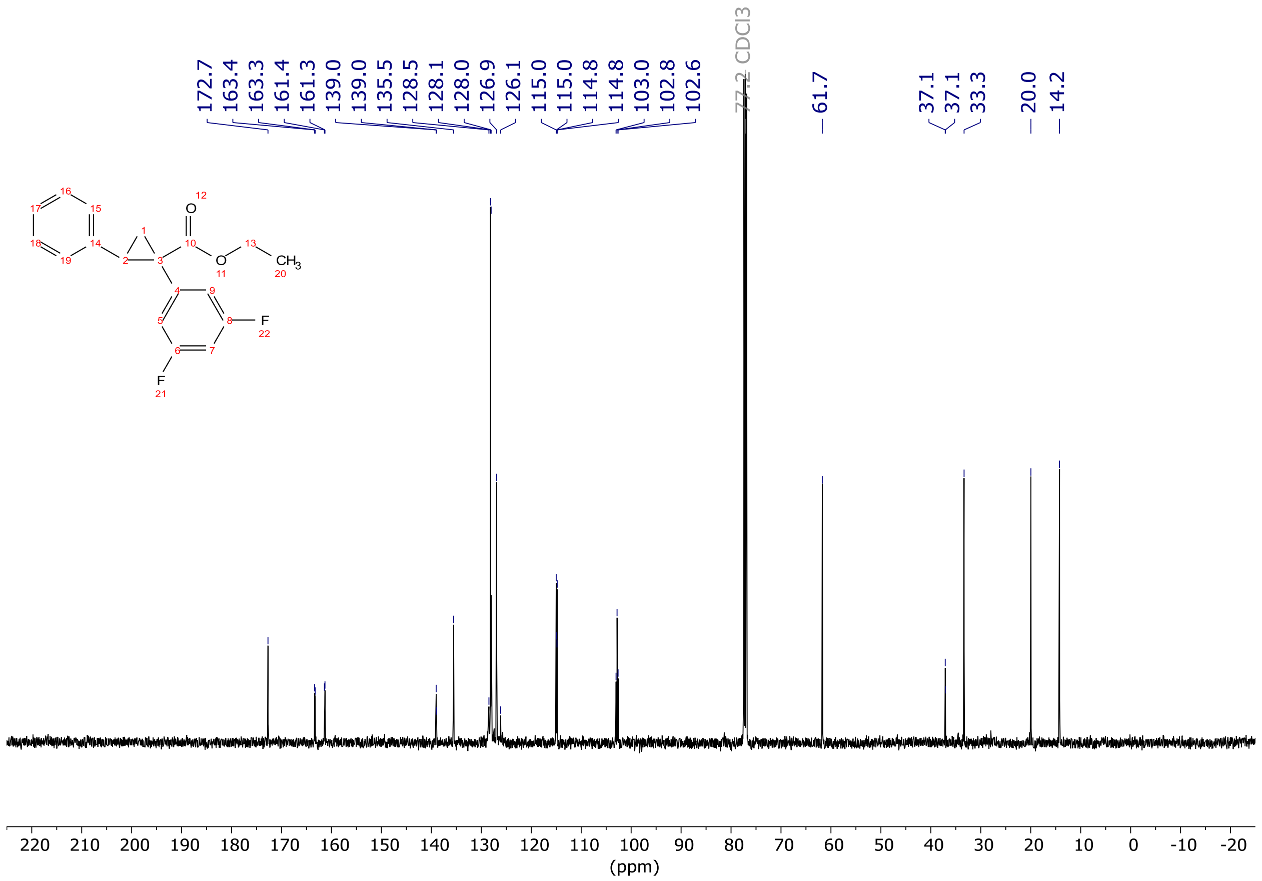

Figure S 156 ^{13}C NMR spectrum of 3.9

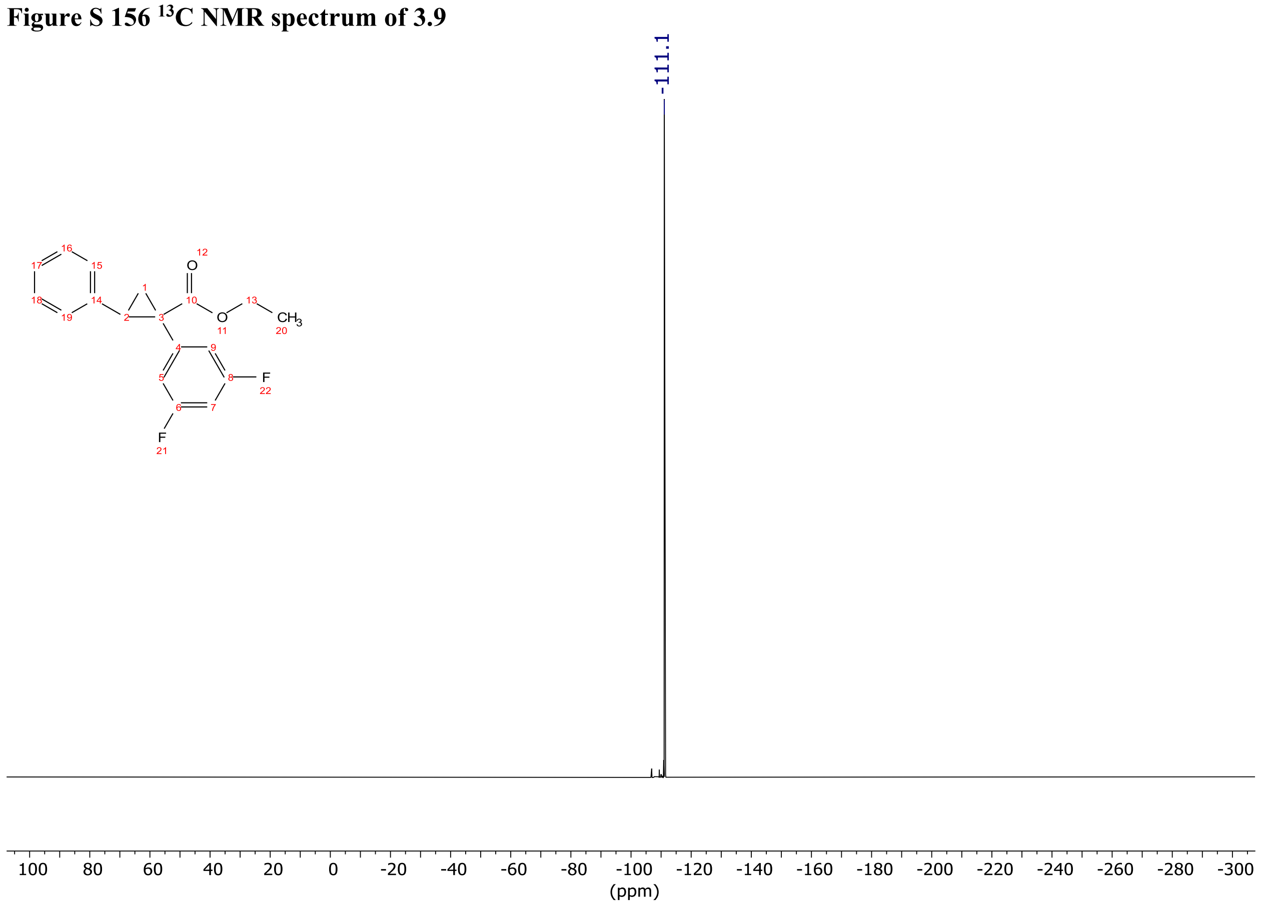

Figure S 157 ^{19}F NMR spectrum of 3.9

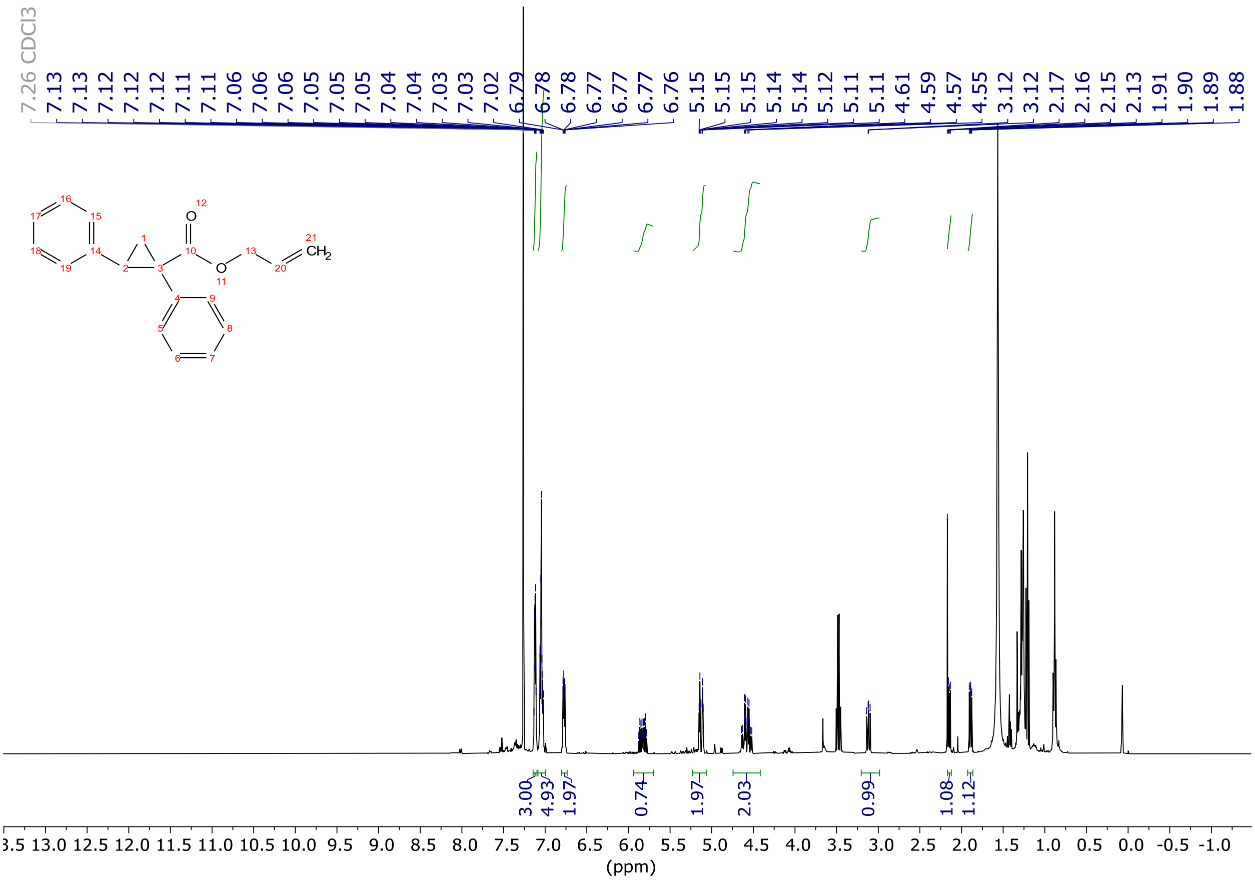

Figure S 158 ^{1}H NMR spectrum of 3.10

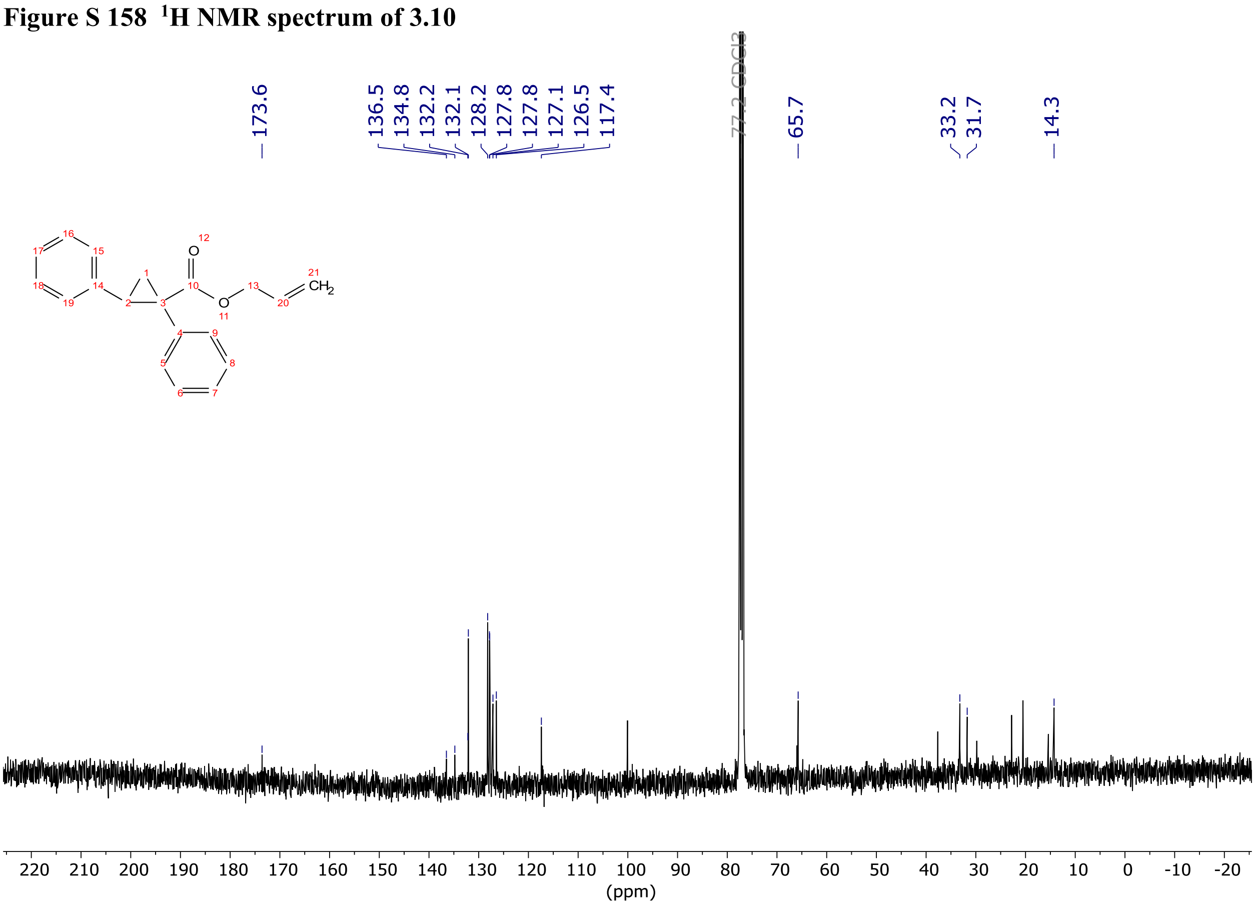

Figure S 159 ^{13}C NMR spectrum of 3.10

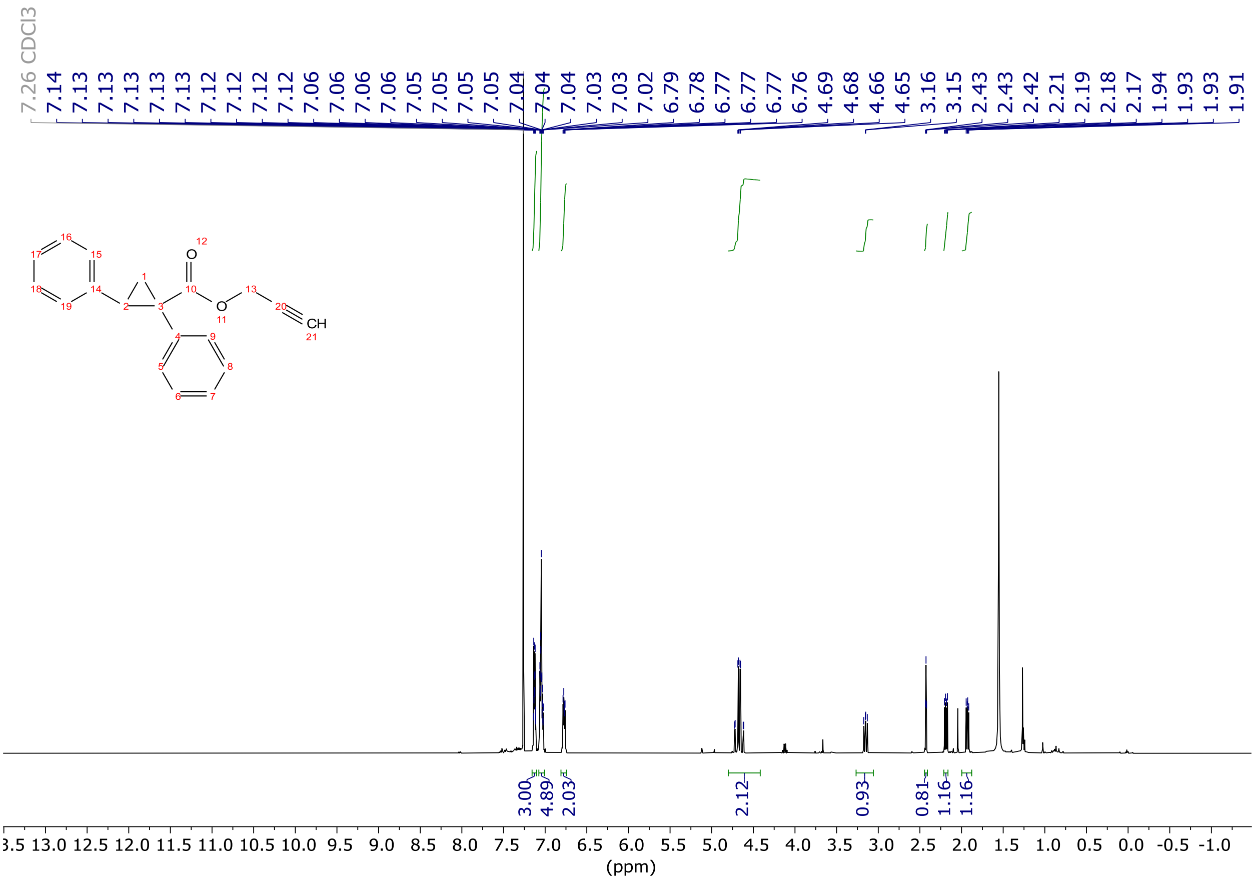

Figure S 160 ^{1}H NMR spectrum of 3.11

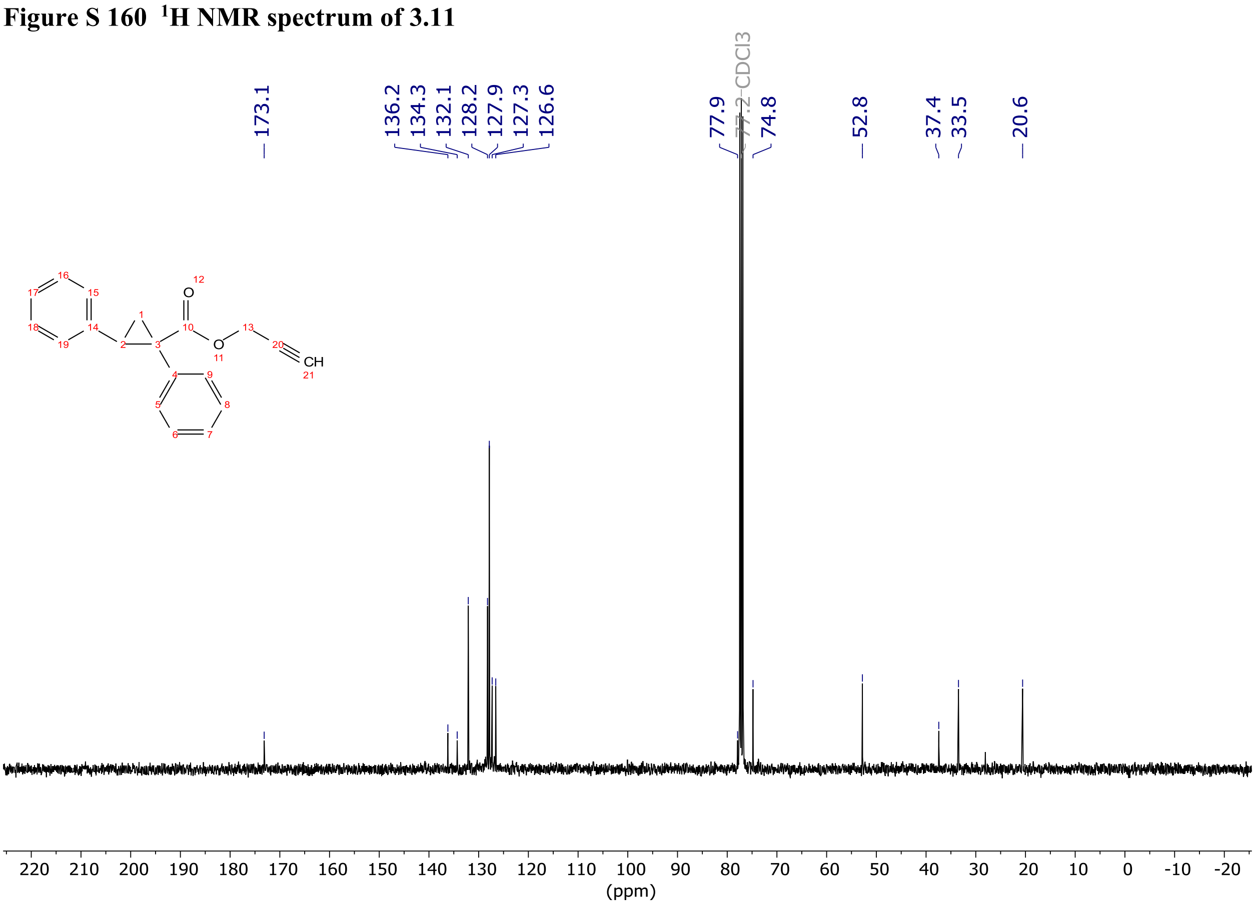

Figure S 161 ^{13}C NMR spectrum of 3.11

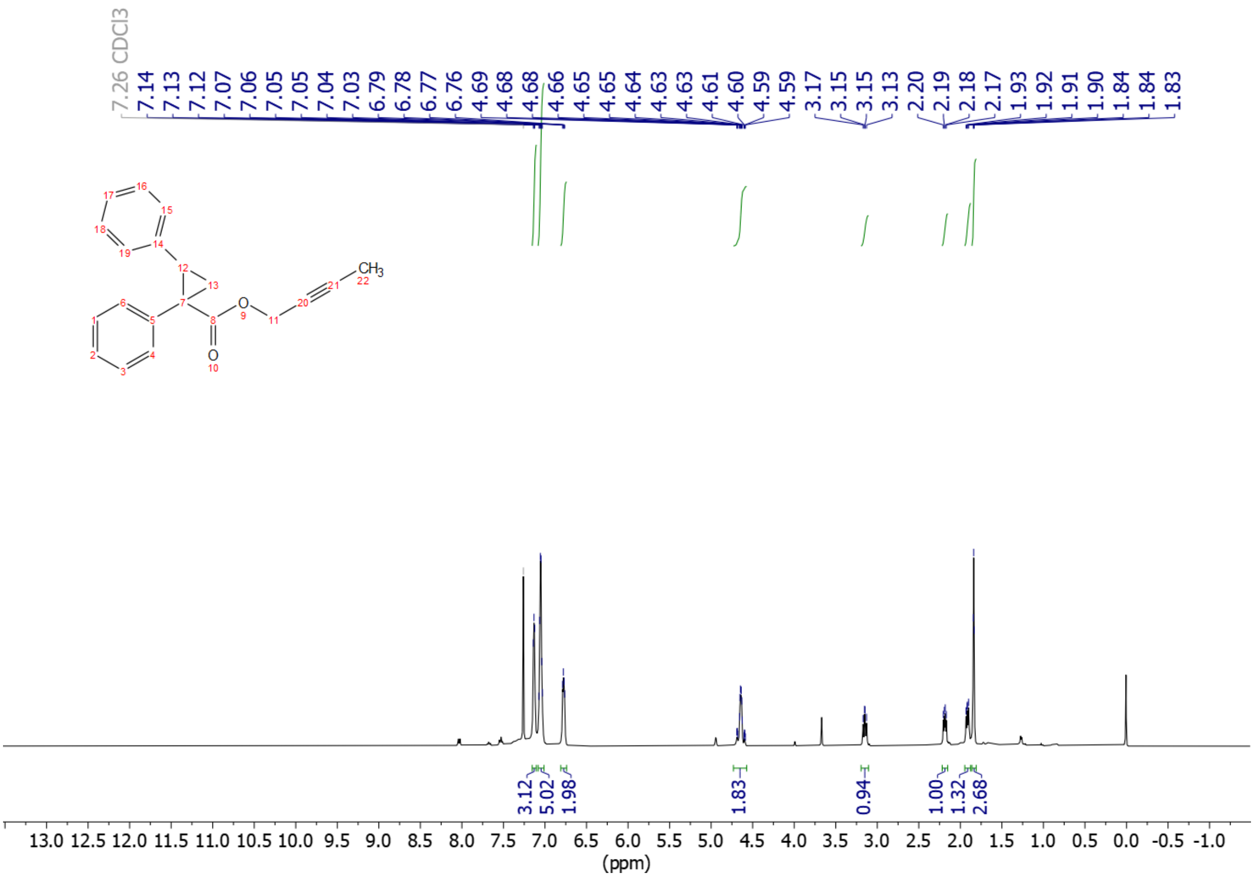

Figure S 162 **¹H NMR spectrum of 3.12**

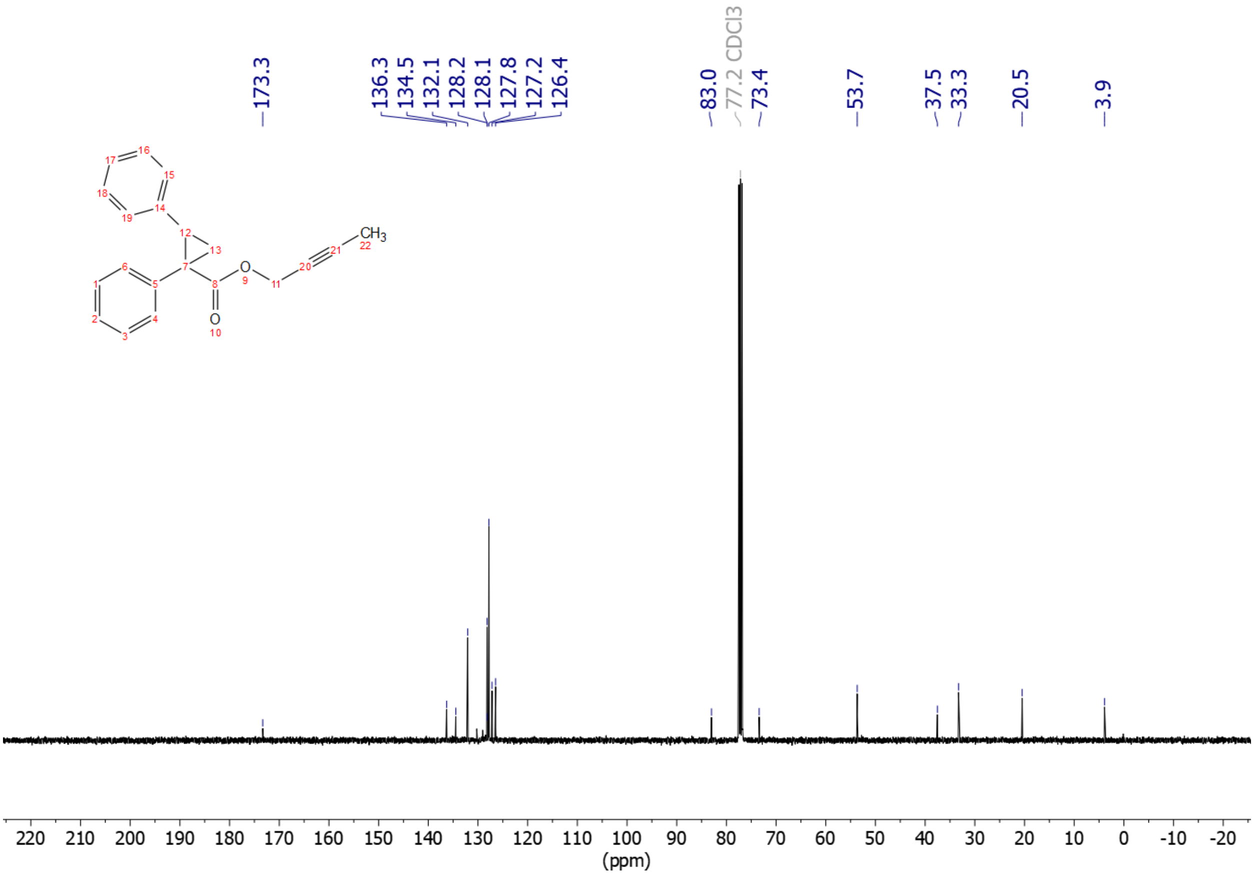

Figure S 163 **¹³C NMR spectrum of 3.12**

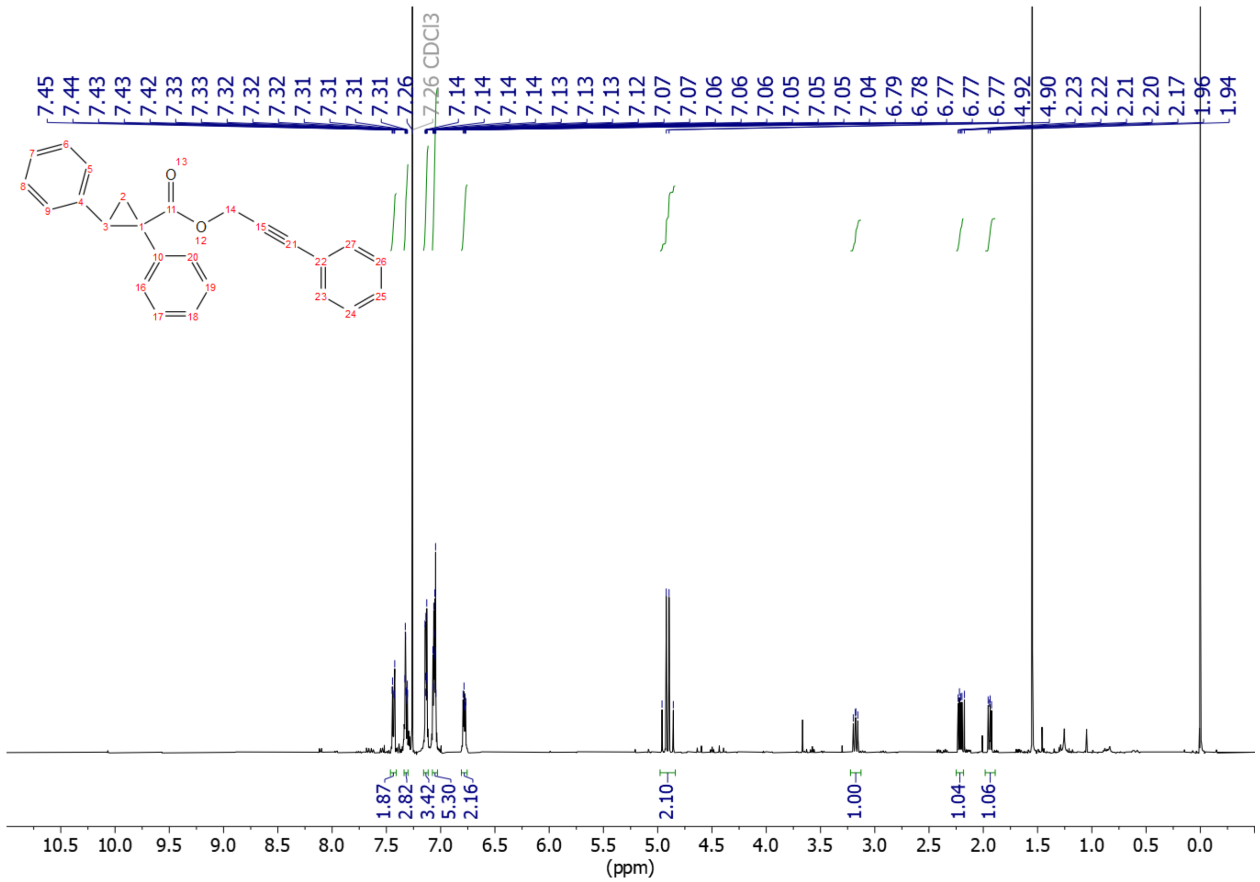

Figure S 164 ^{1}H NMR spectrum of 3.13

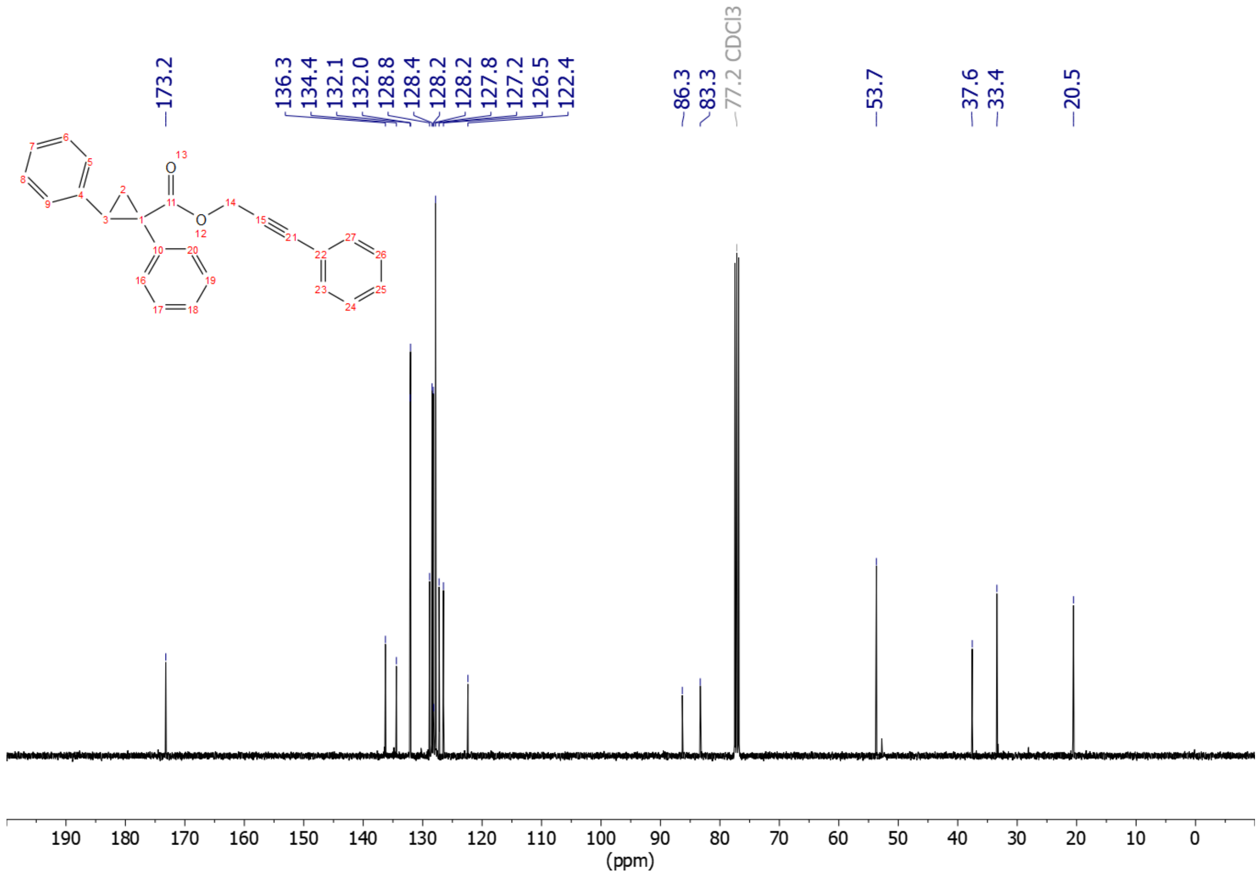

Figure S 165 ^{13}C NMR spectrum of 3.13

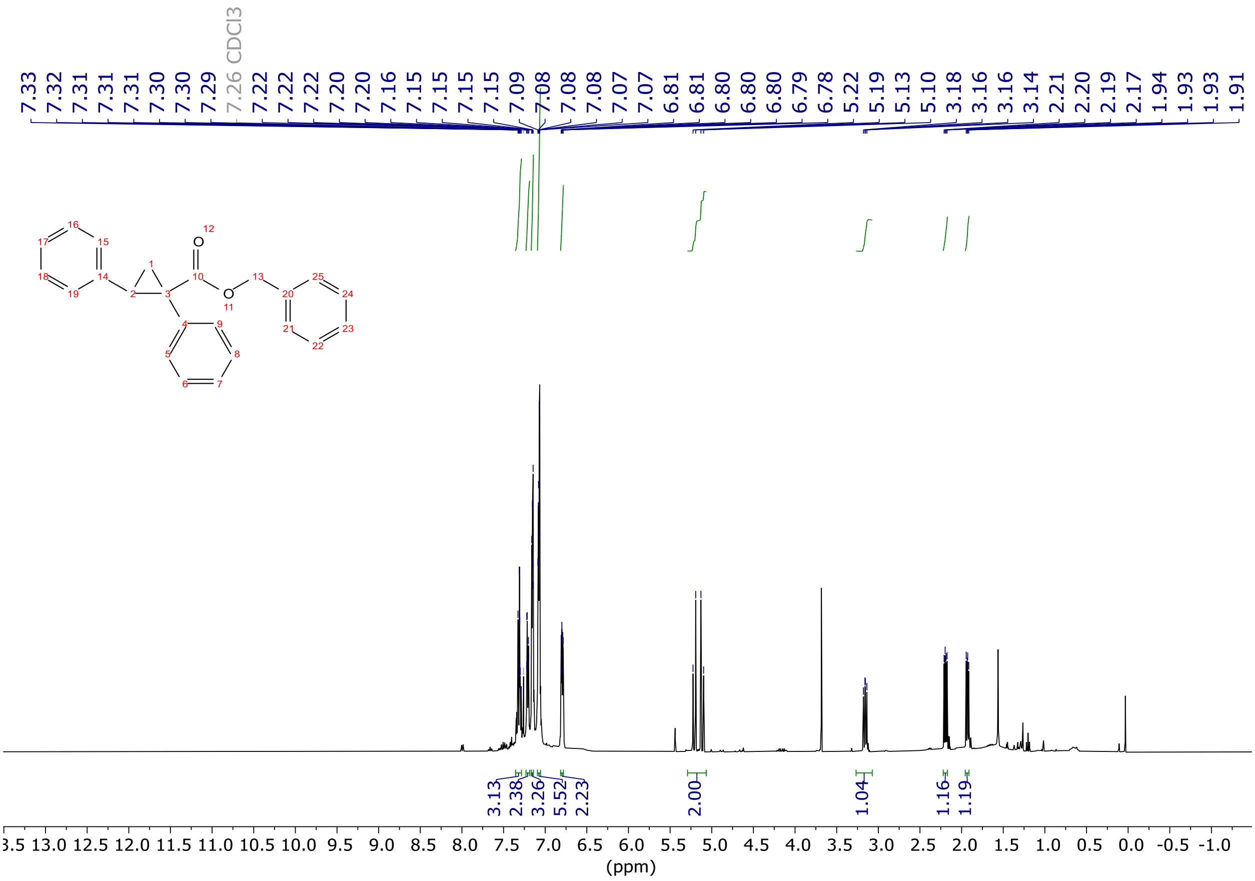

Figure S 166 **¹H NMR spectrum of 3.14**

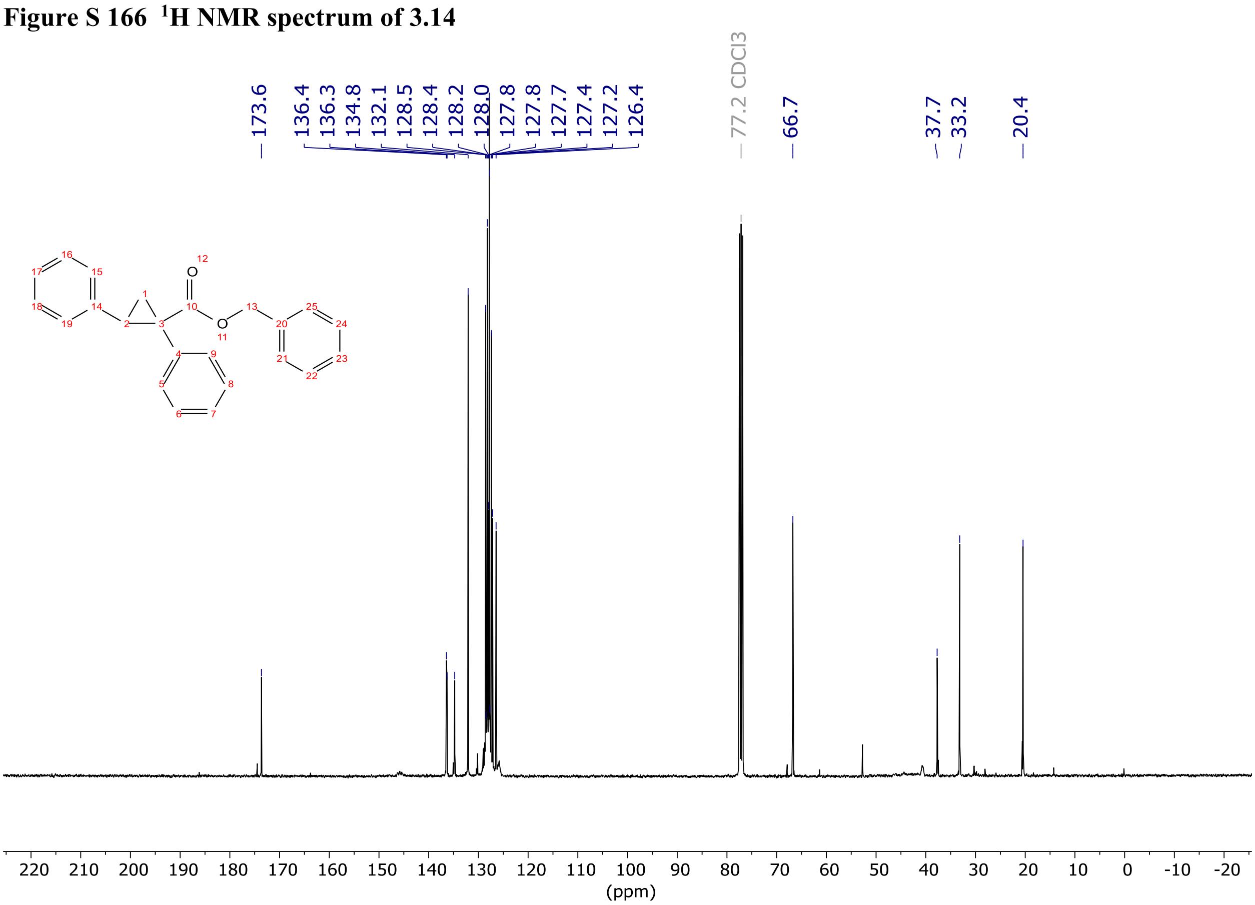

Figure S 167 **¹³C NMR spectrum of 3.14**

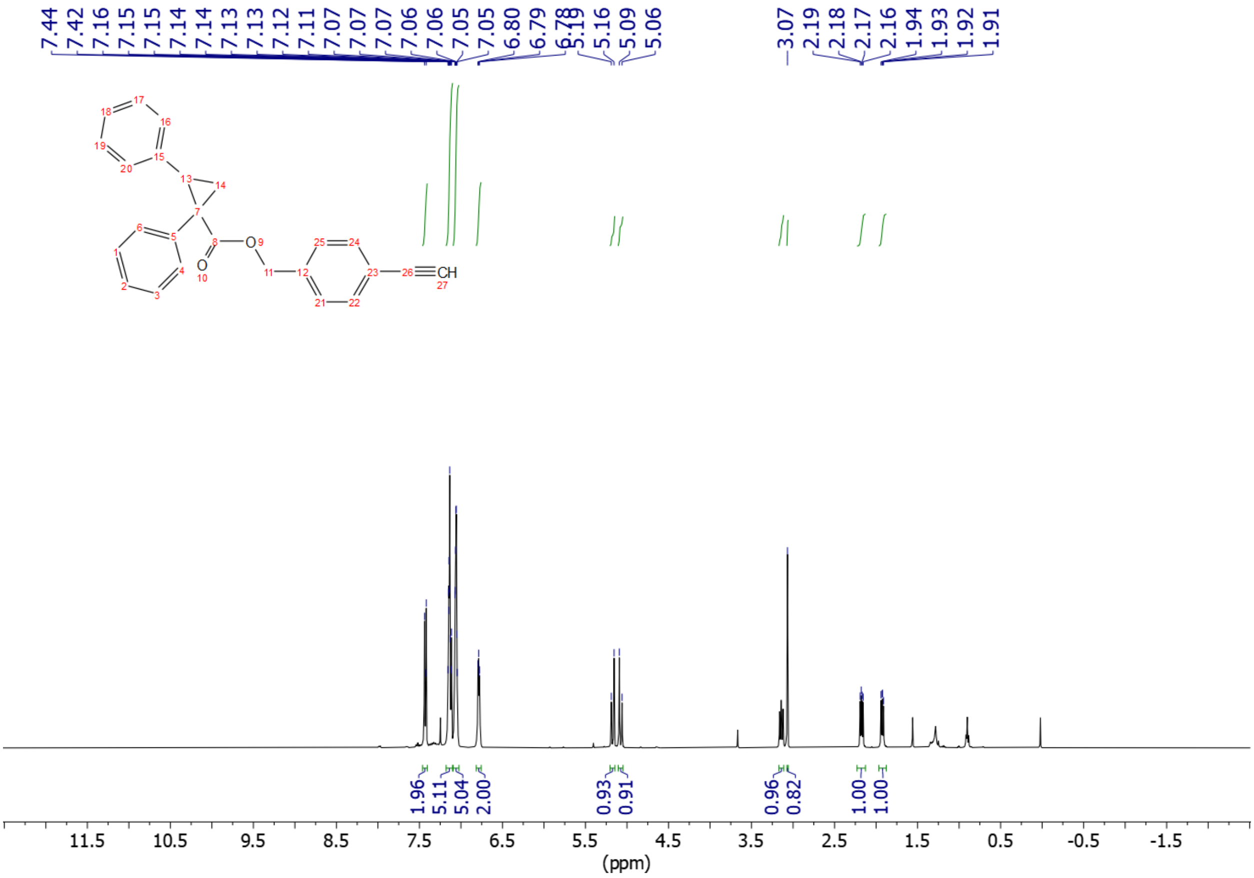

Figure S 168 ^{1}H NMR spectrum of 3.15

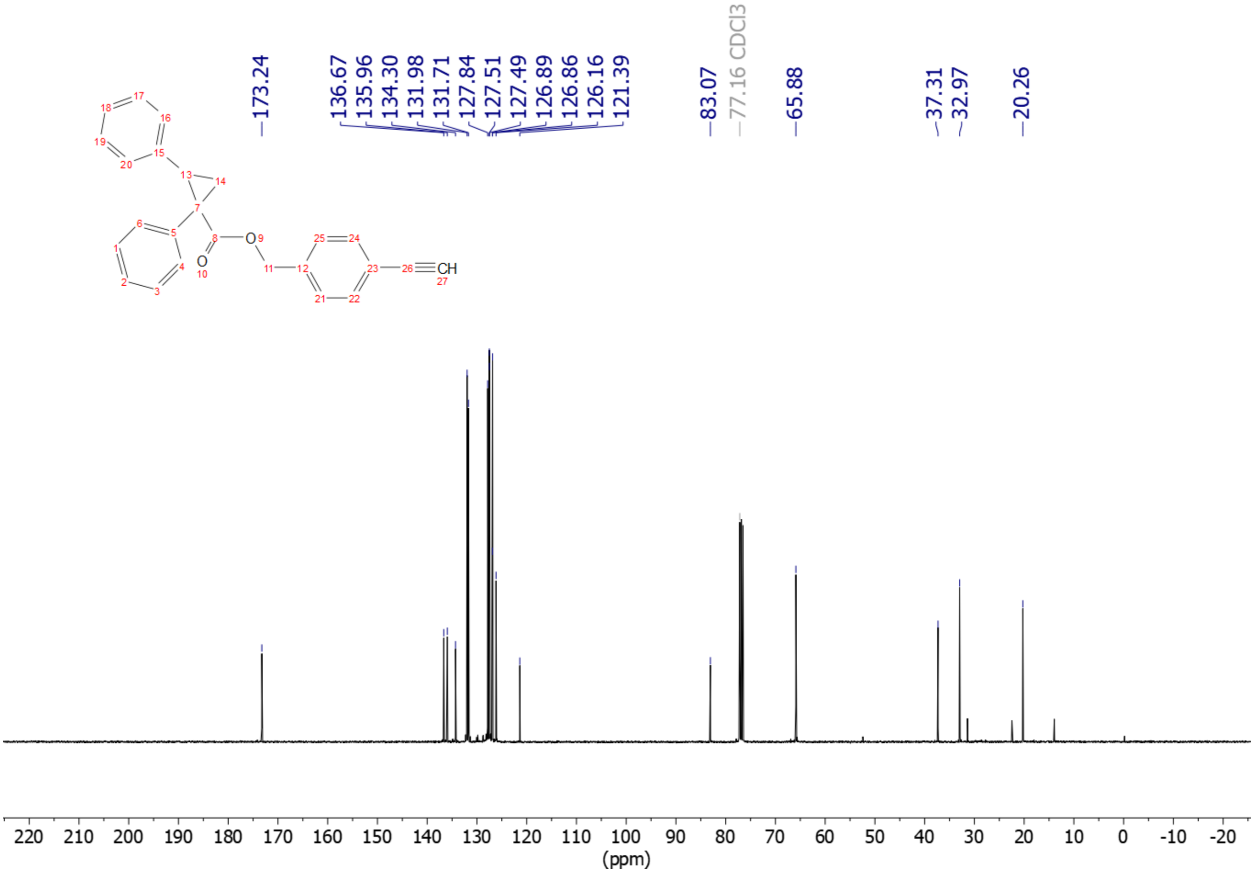

Figure S 169 ^{13}C NMR spectrum of 3.15

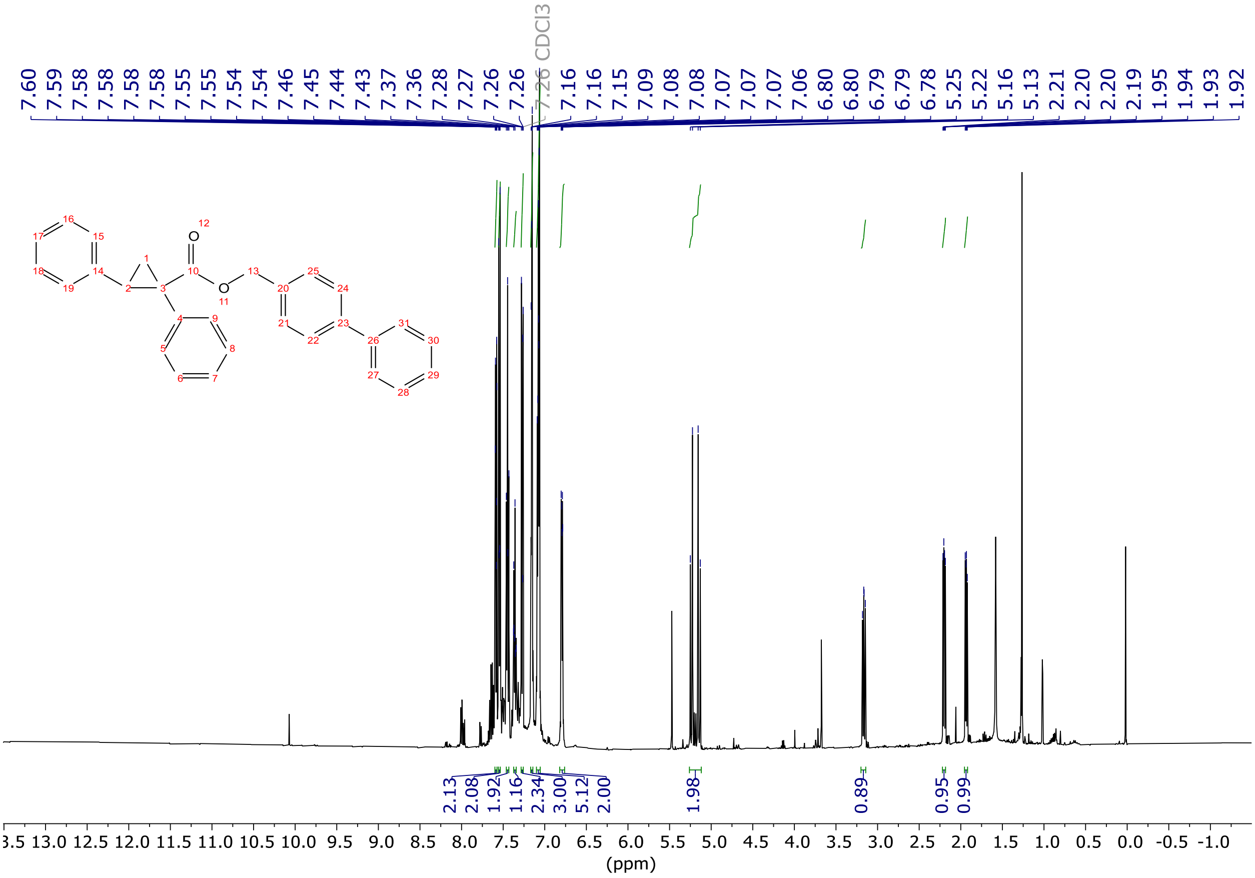

Figure S 170 ^{1}H NMR spectrum of 3.16

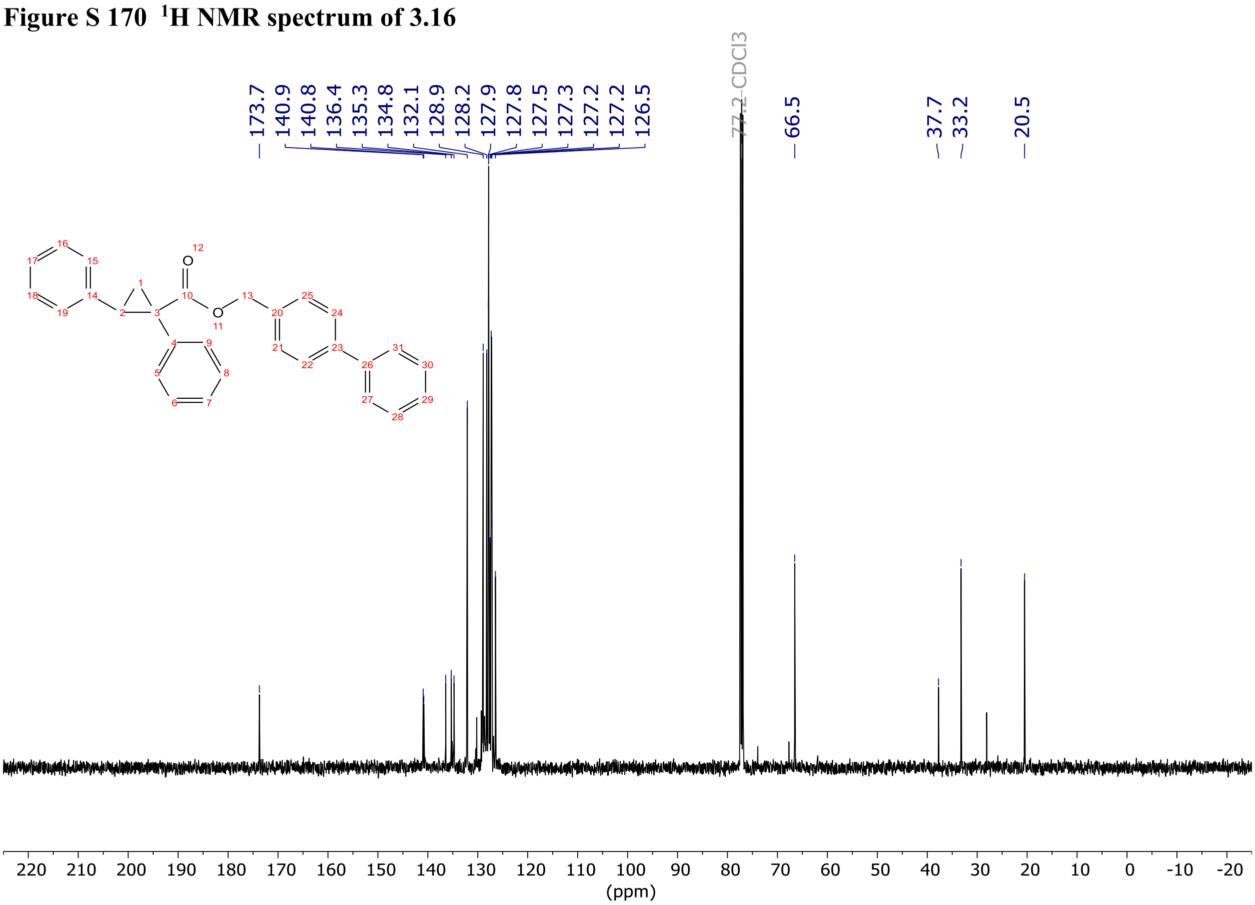

Figure S 171 ^{13}C NMR spectrum of 3.16

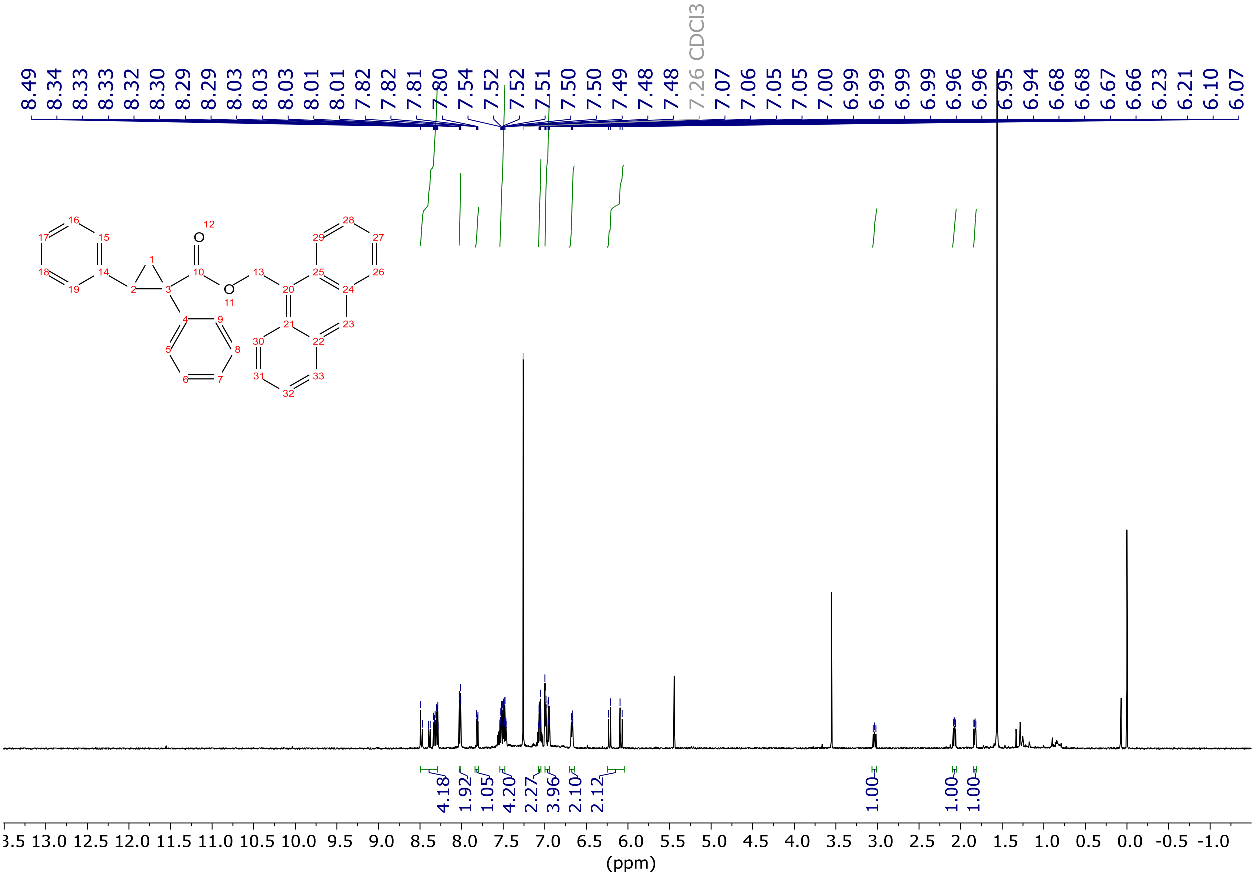

Figure S 172 ^{1}H NMR spectrum of 3.17

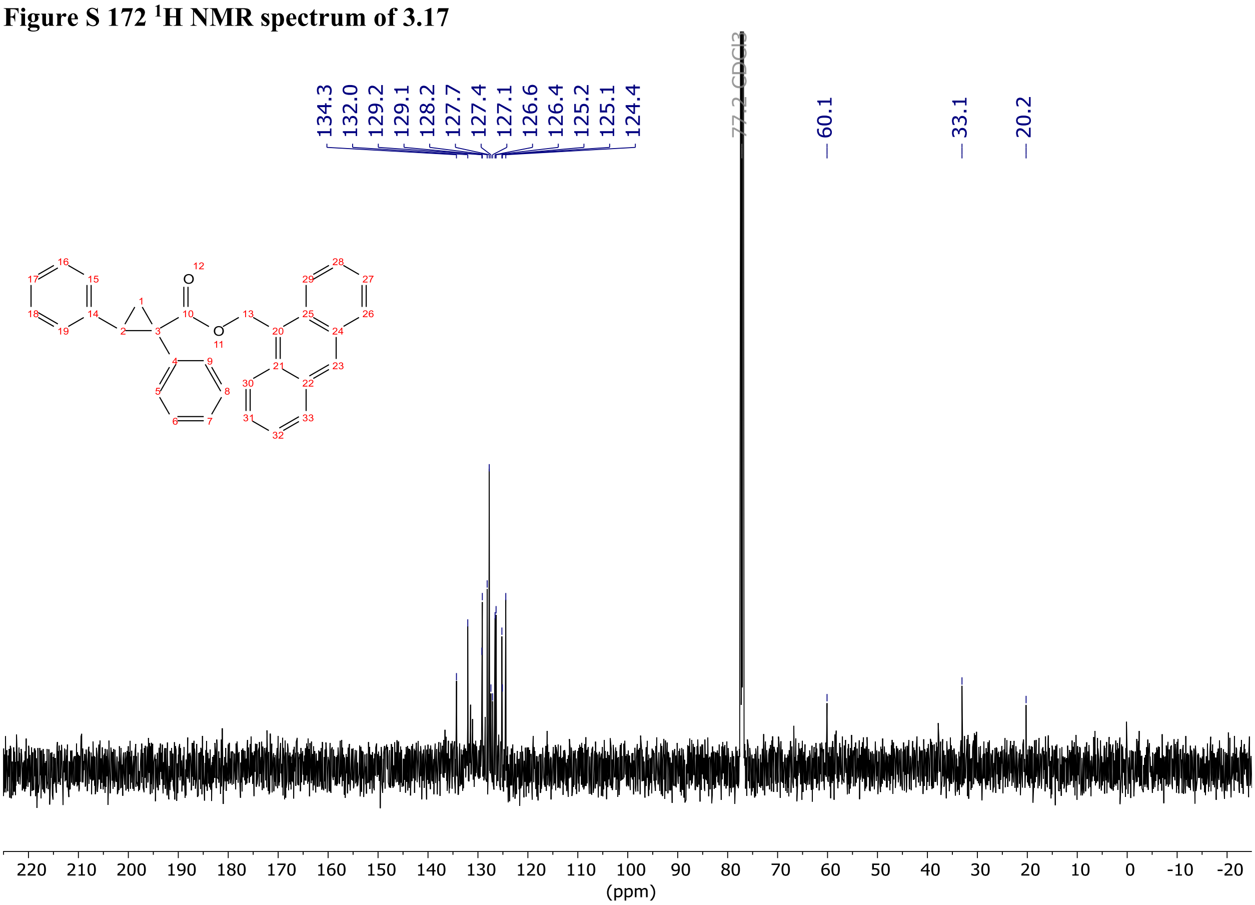

Figure S 173 ^{13}C NMR spectrum of 3.17

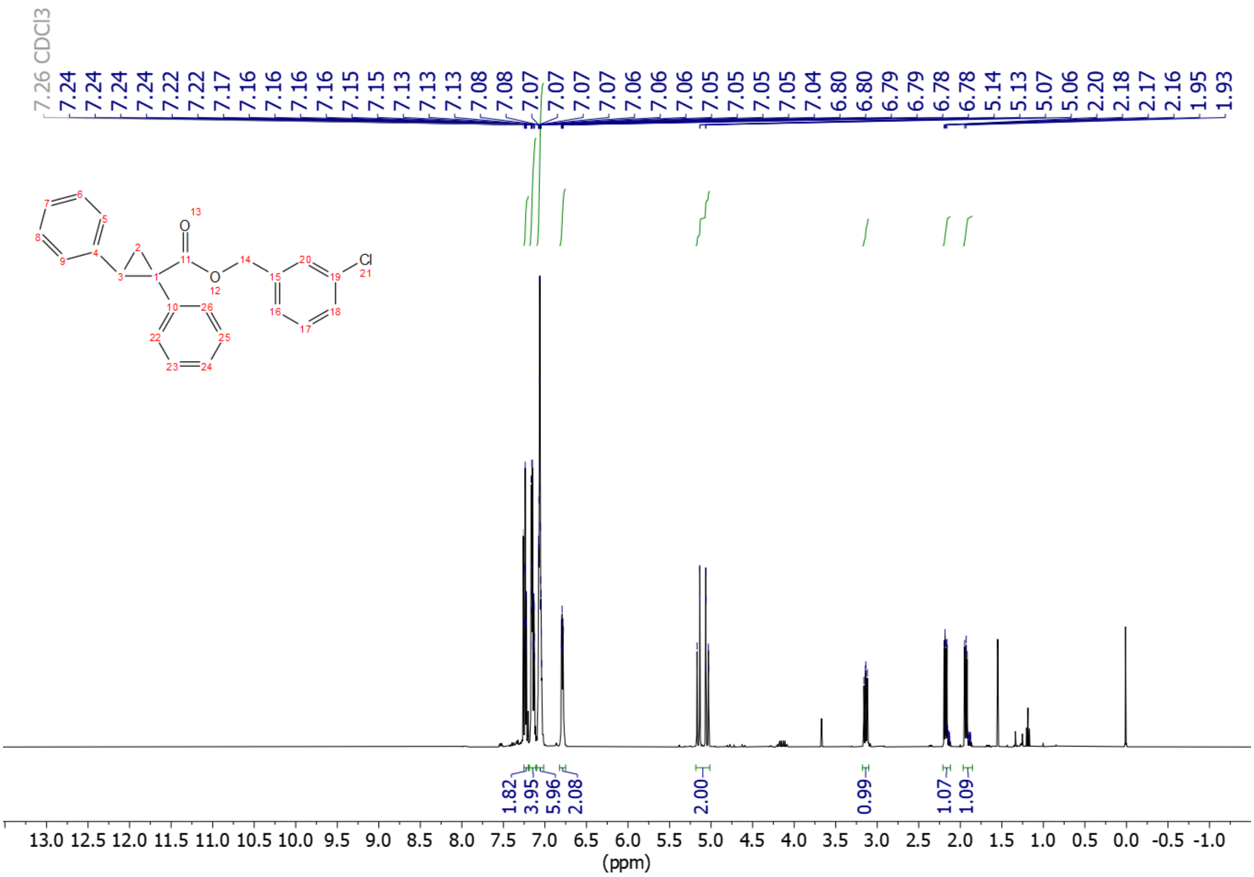

Figure S 174 ^{1}H NMR spectrum of 3.18

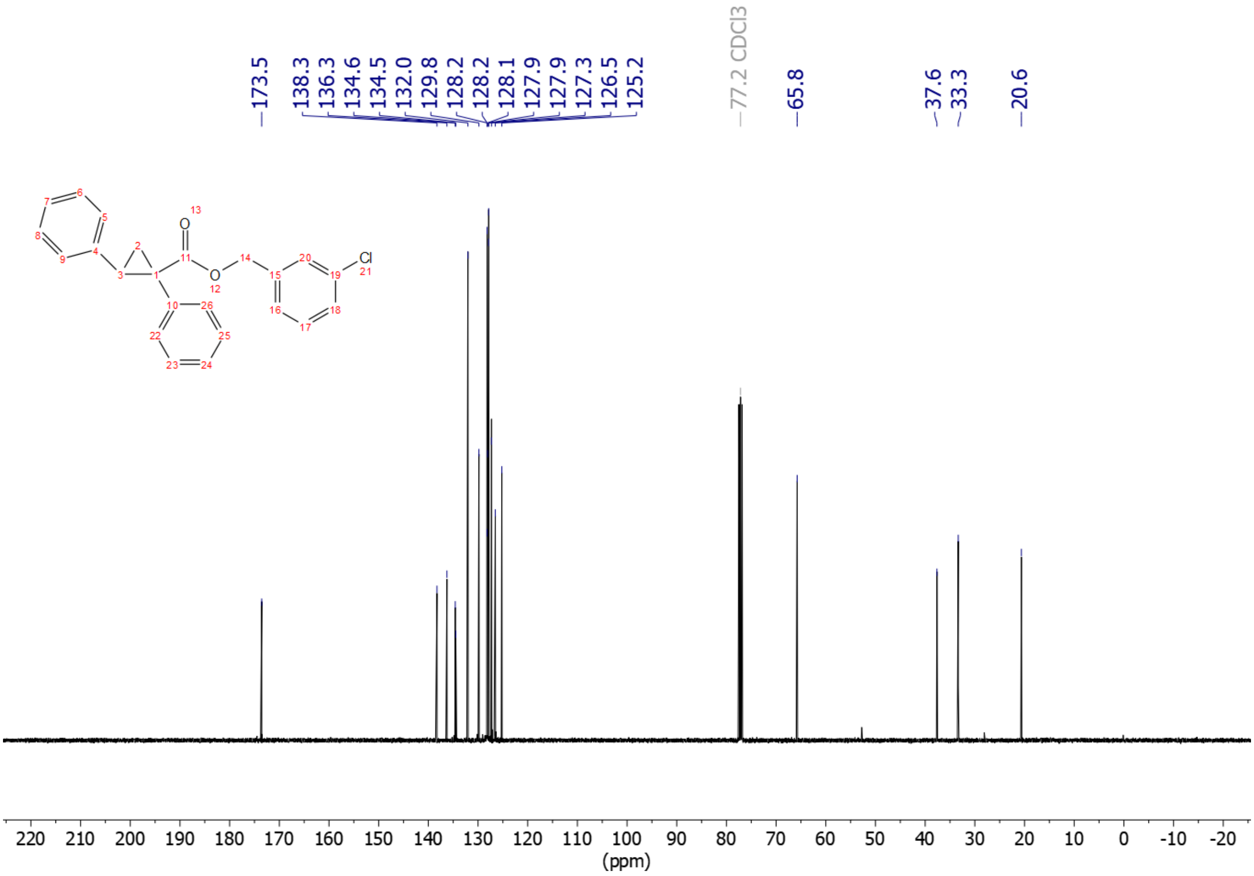

Figure S 175 ^{13}C NMR spectrum of 3.18

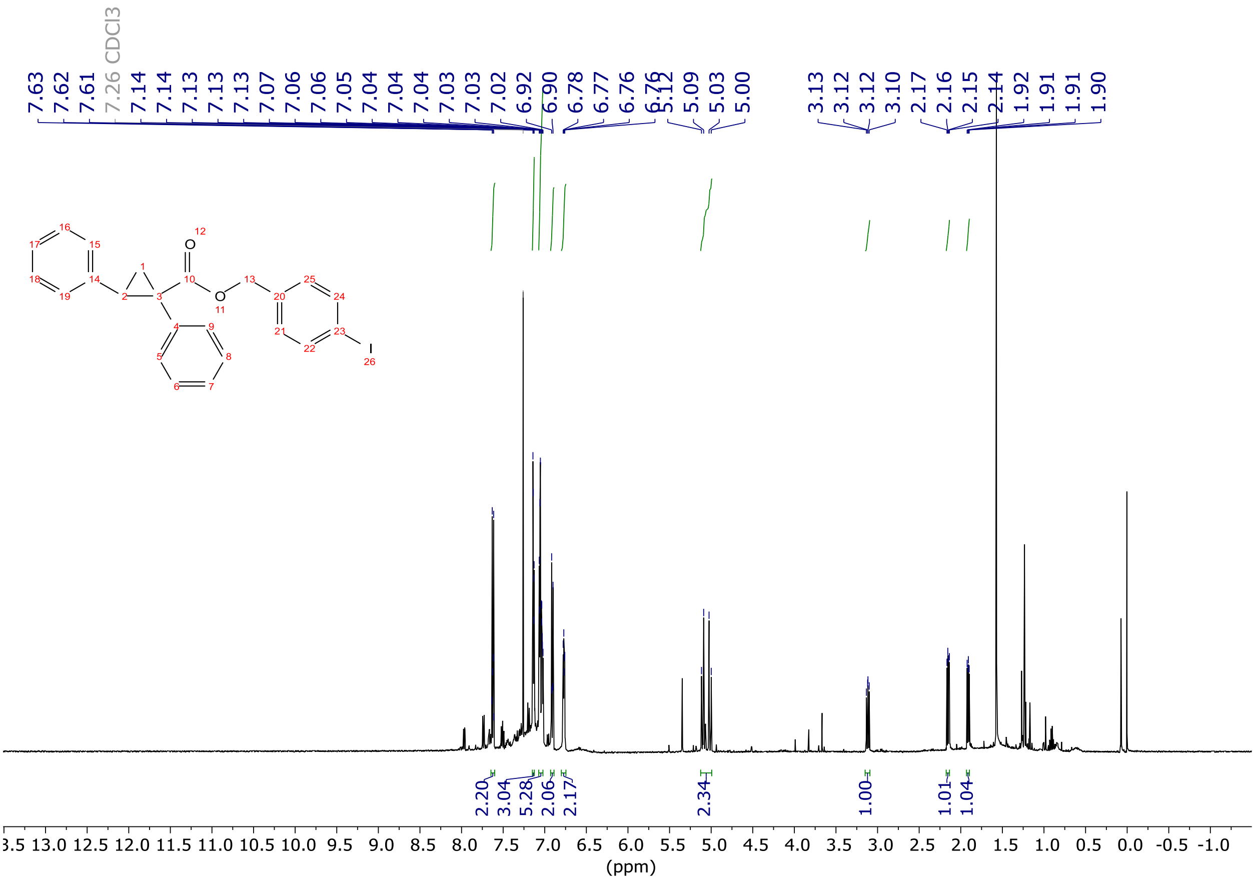

Figure S 176 ^{1}H NMR spectrum of 3.19

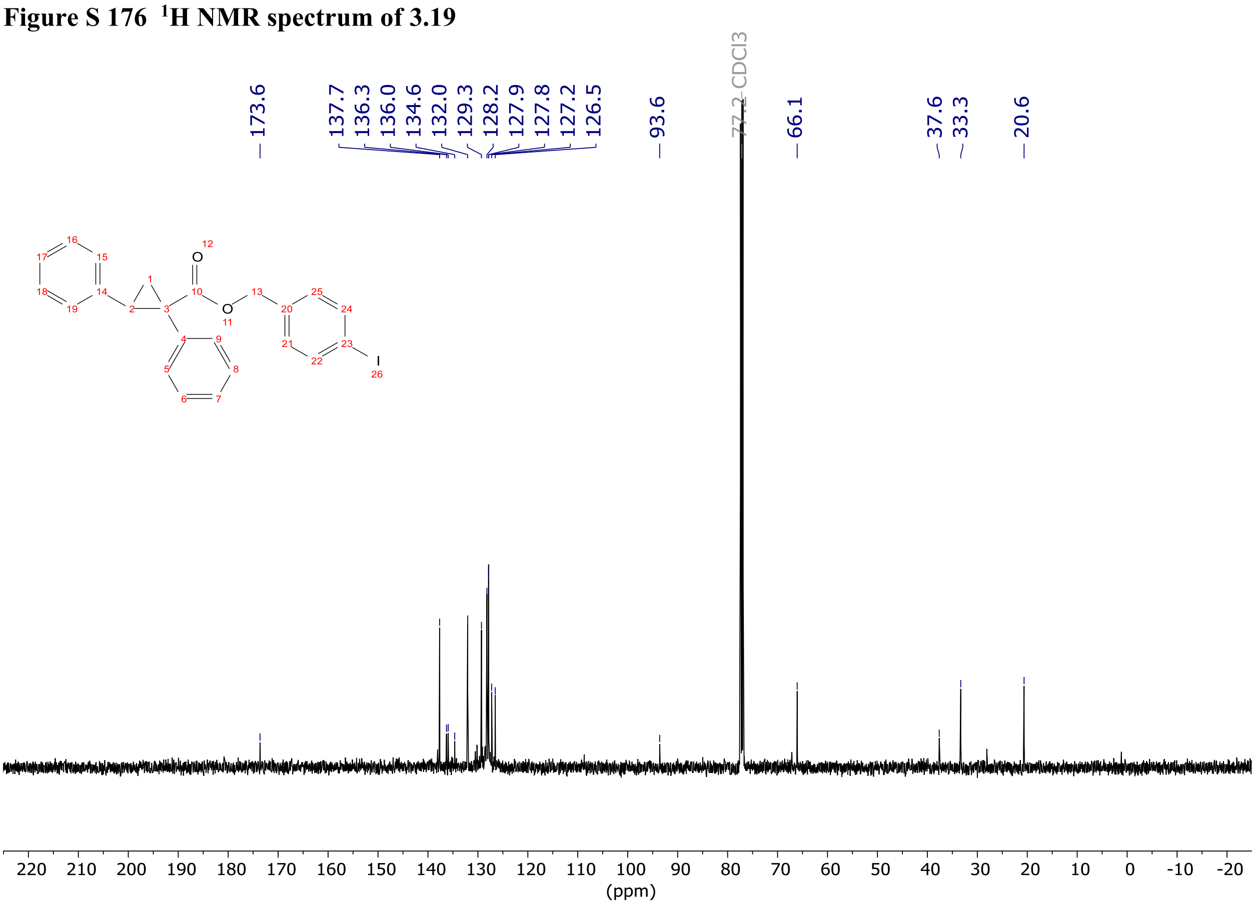

Figure S 177 ^{13}C NMR spectrum of 3.19

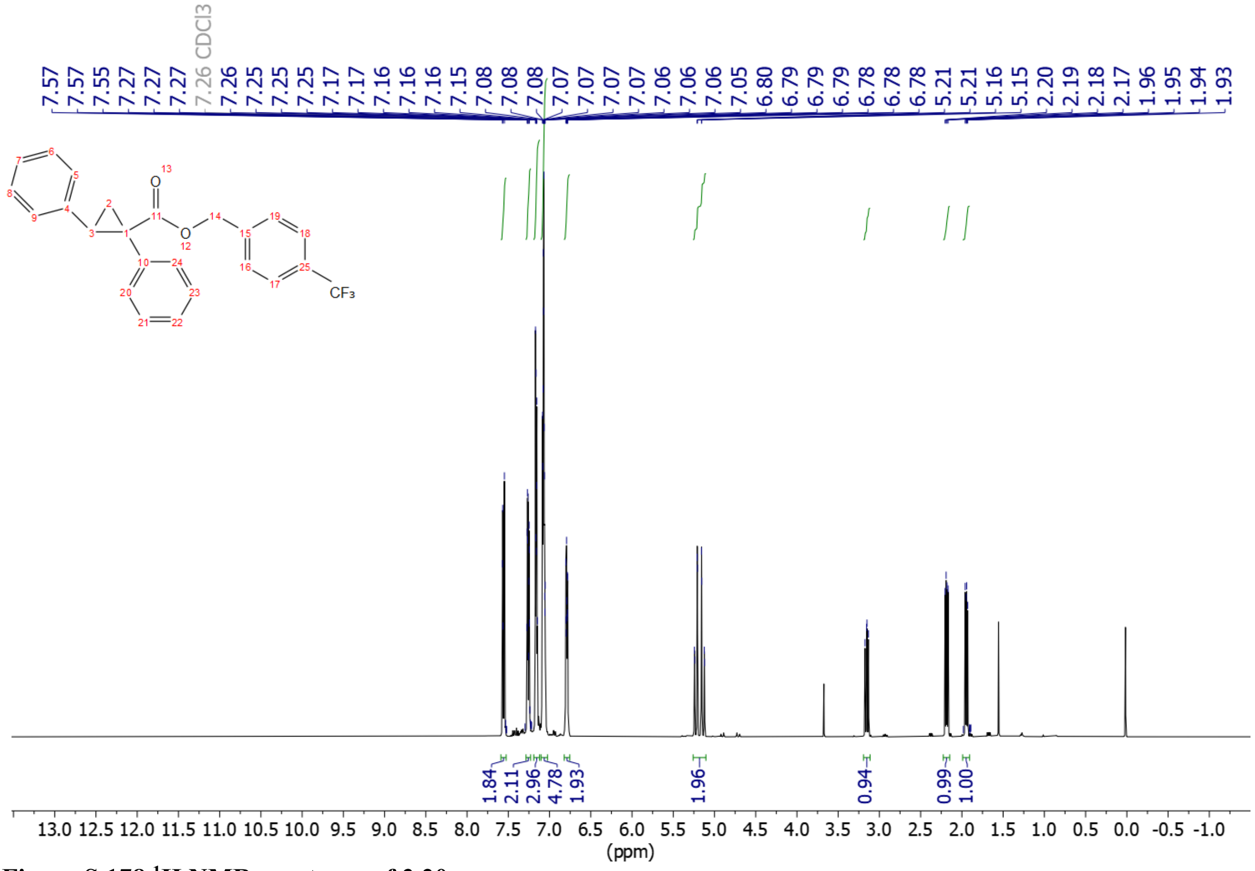

Figure S 178 ^{1}H NMR spectrum of 3.20

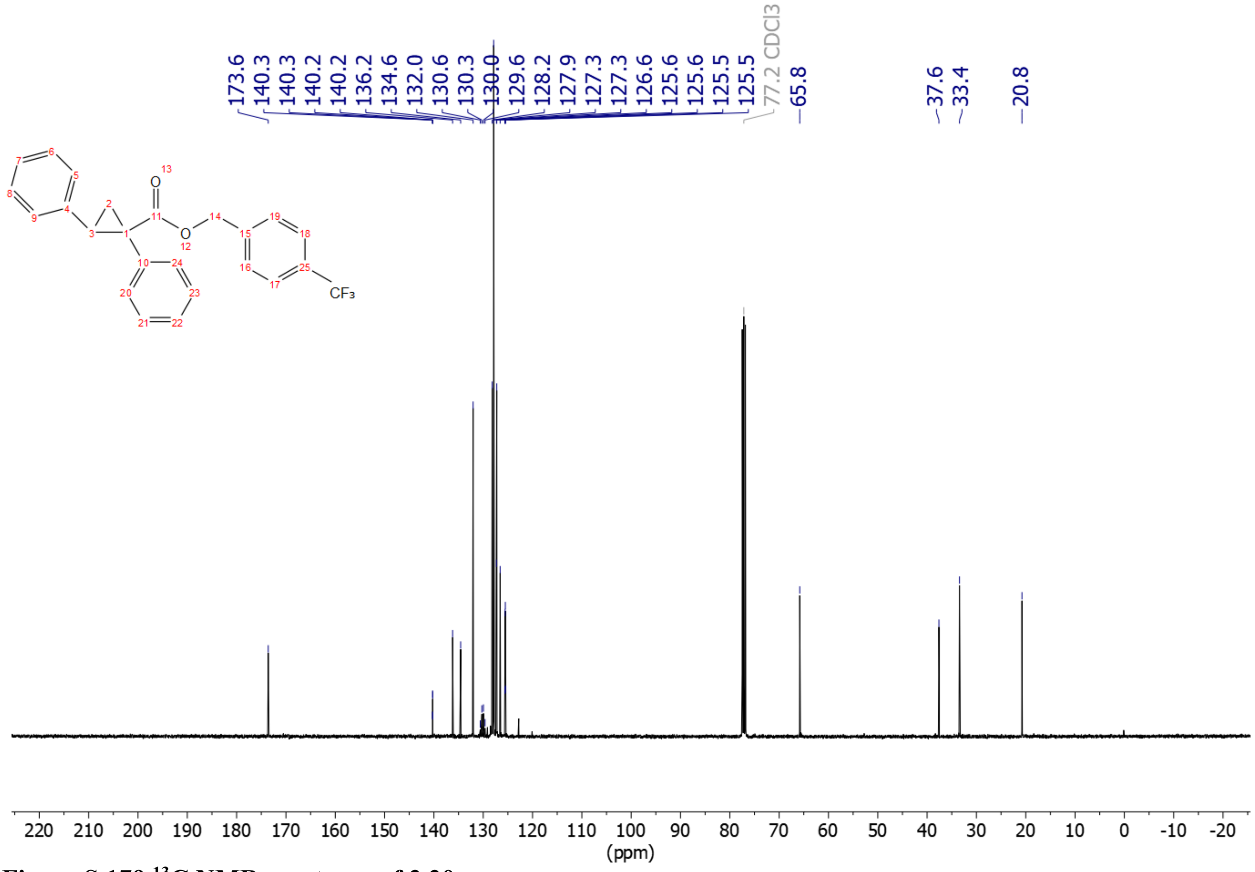

Figure S 179 ^{13}C NMR spectrum of 3.20

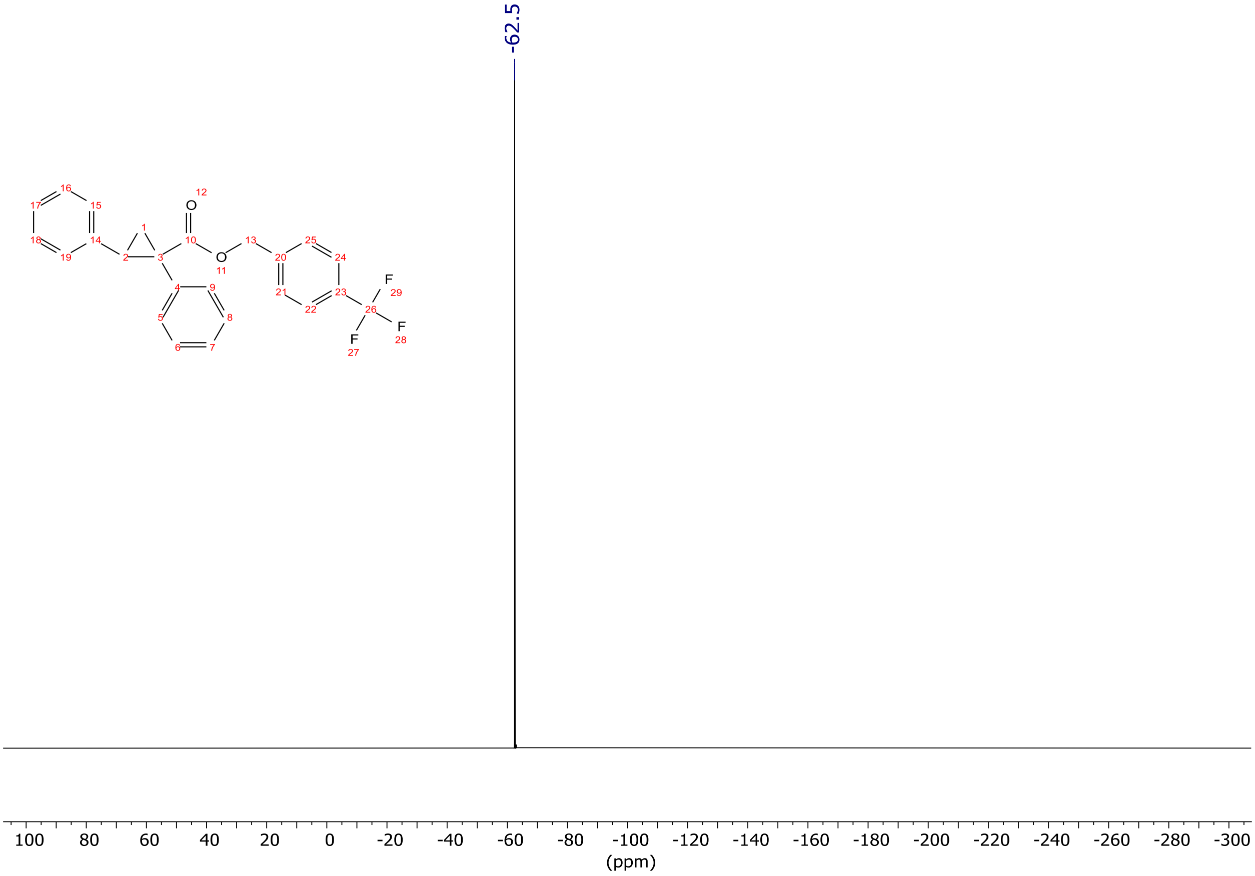

Figure S 180 ^{19}F NMR spectrum of 3.20

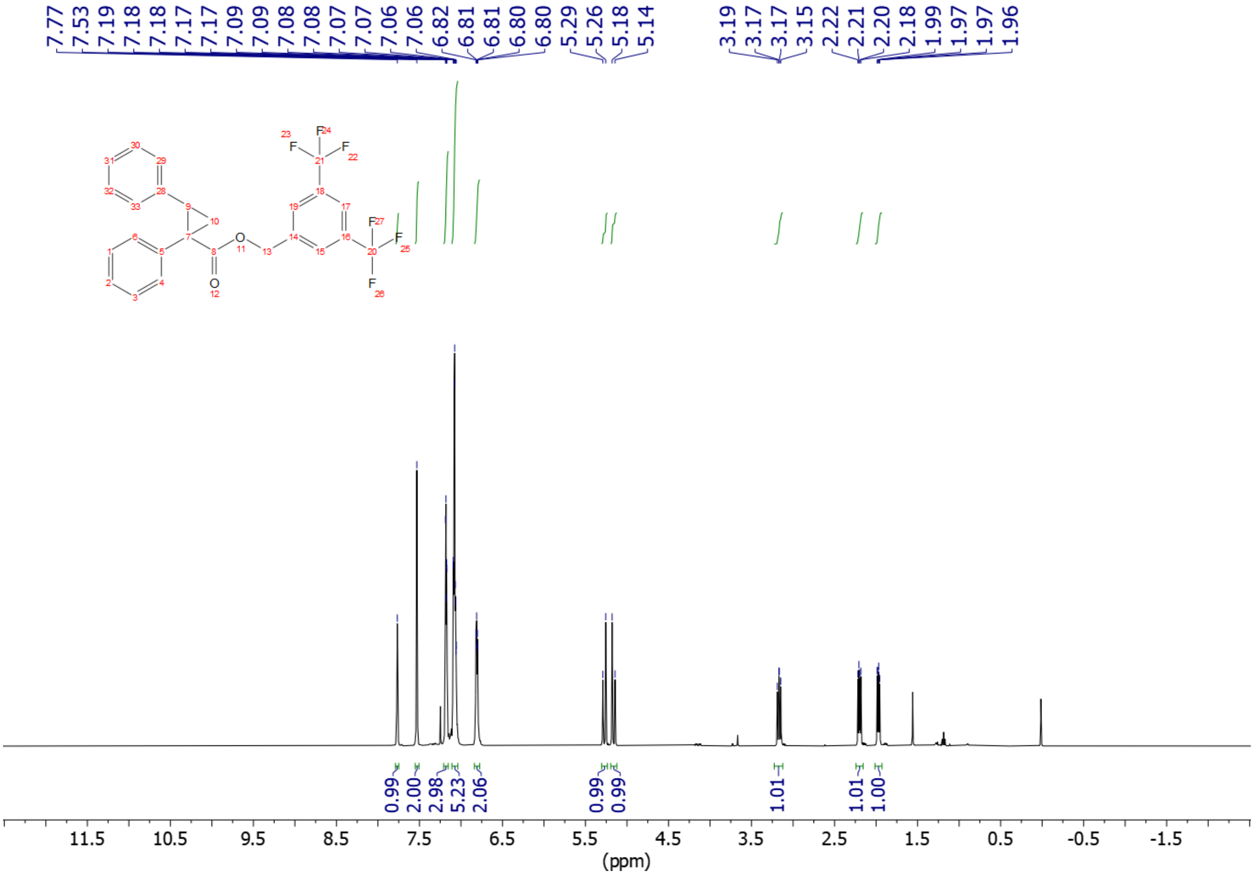

Figure S 181 ^{1}H NMR spectrum of 3.21

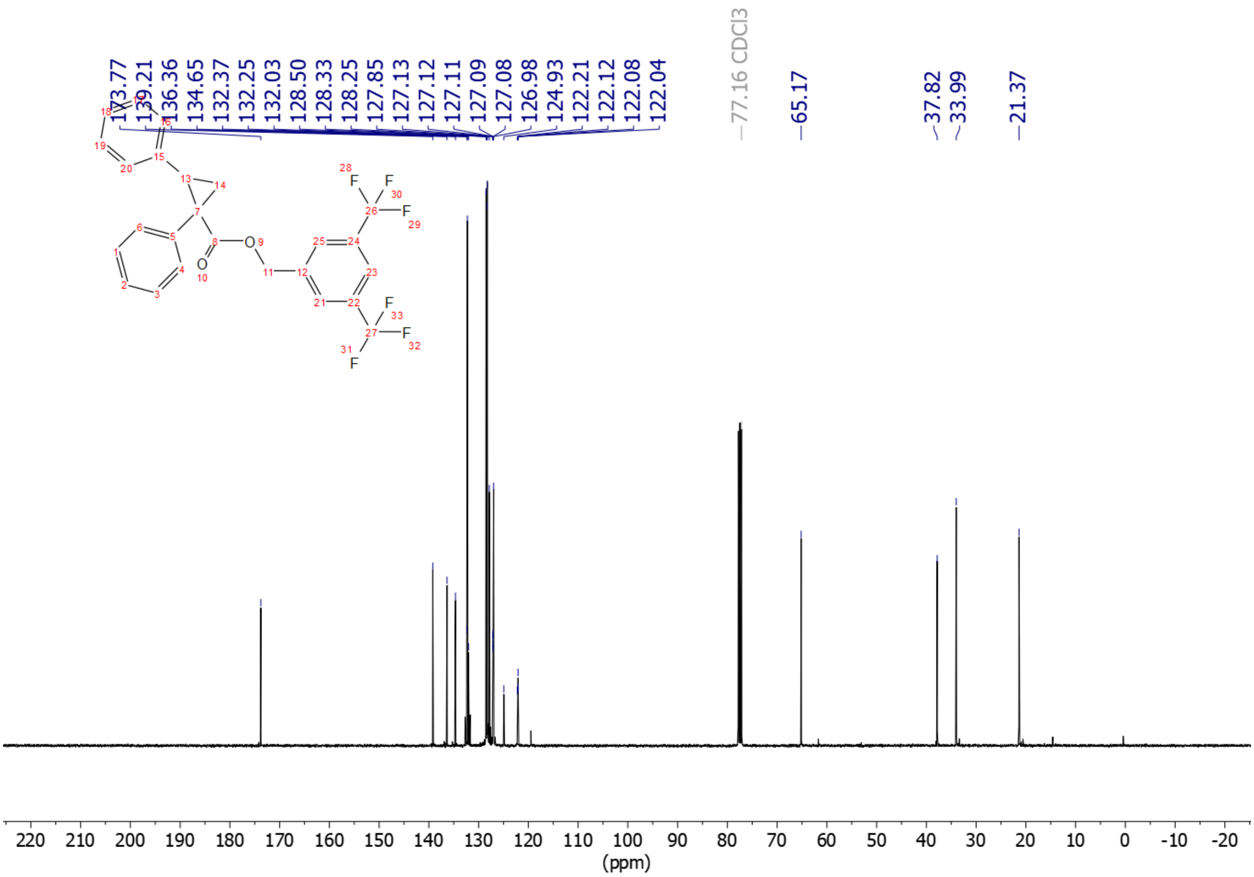

Figure S 182 ^{13}C NMR spectrum of 3.21

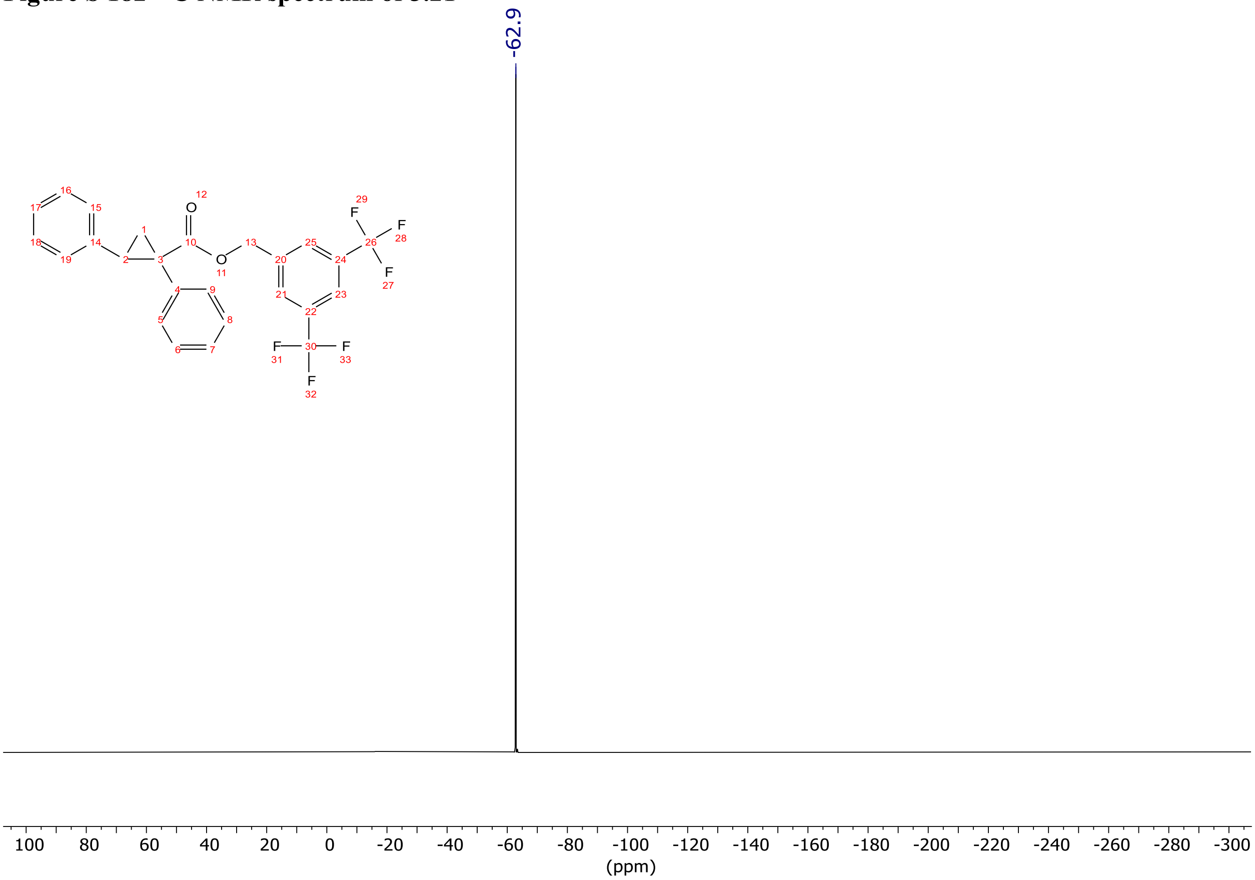

Figure S 183 ^{19}F NMR spectrum of 3.21

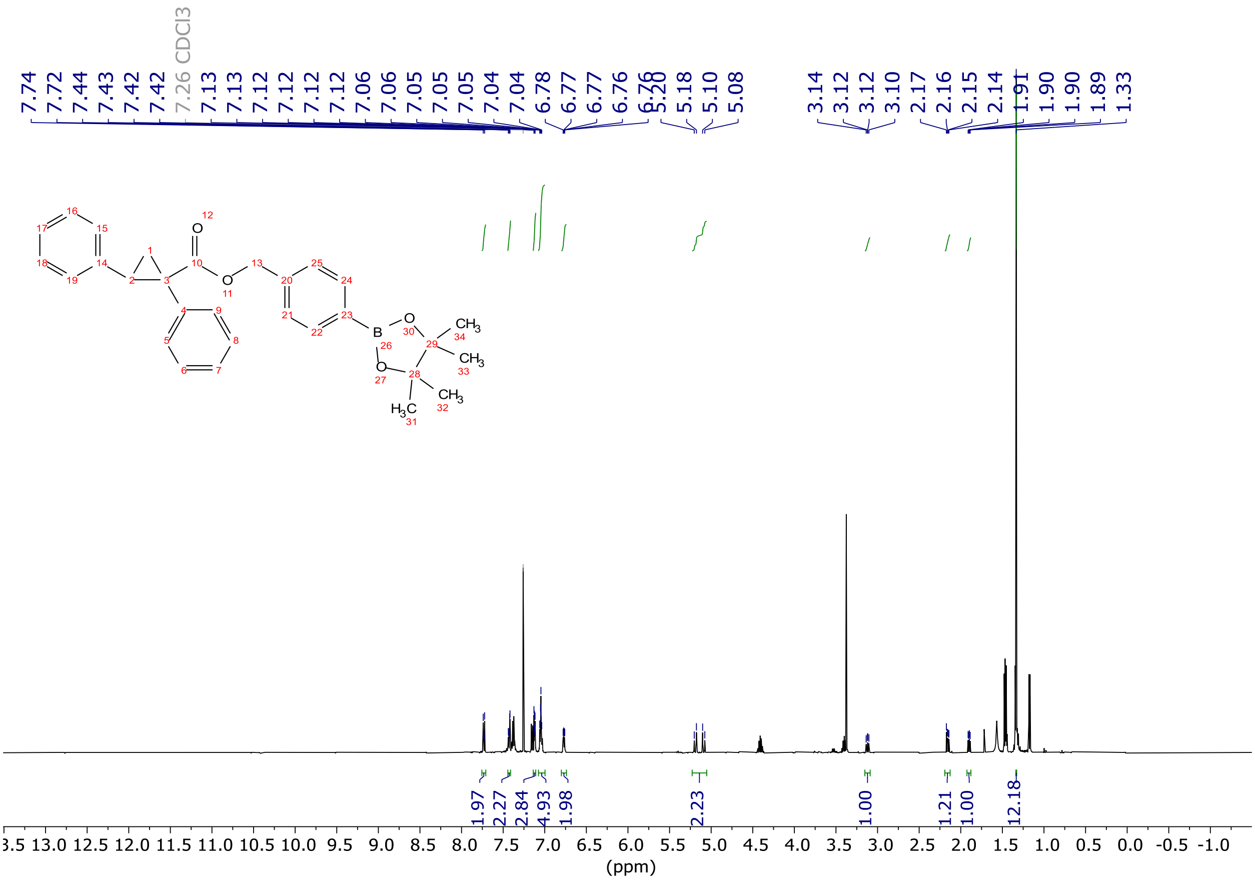

Figure S 184 ^{1}H NMR spectrum of 3.22

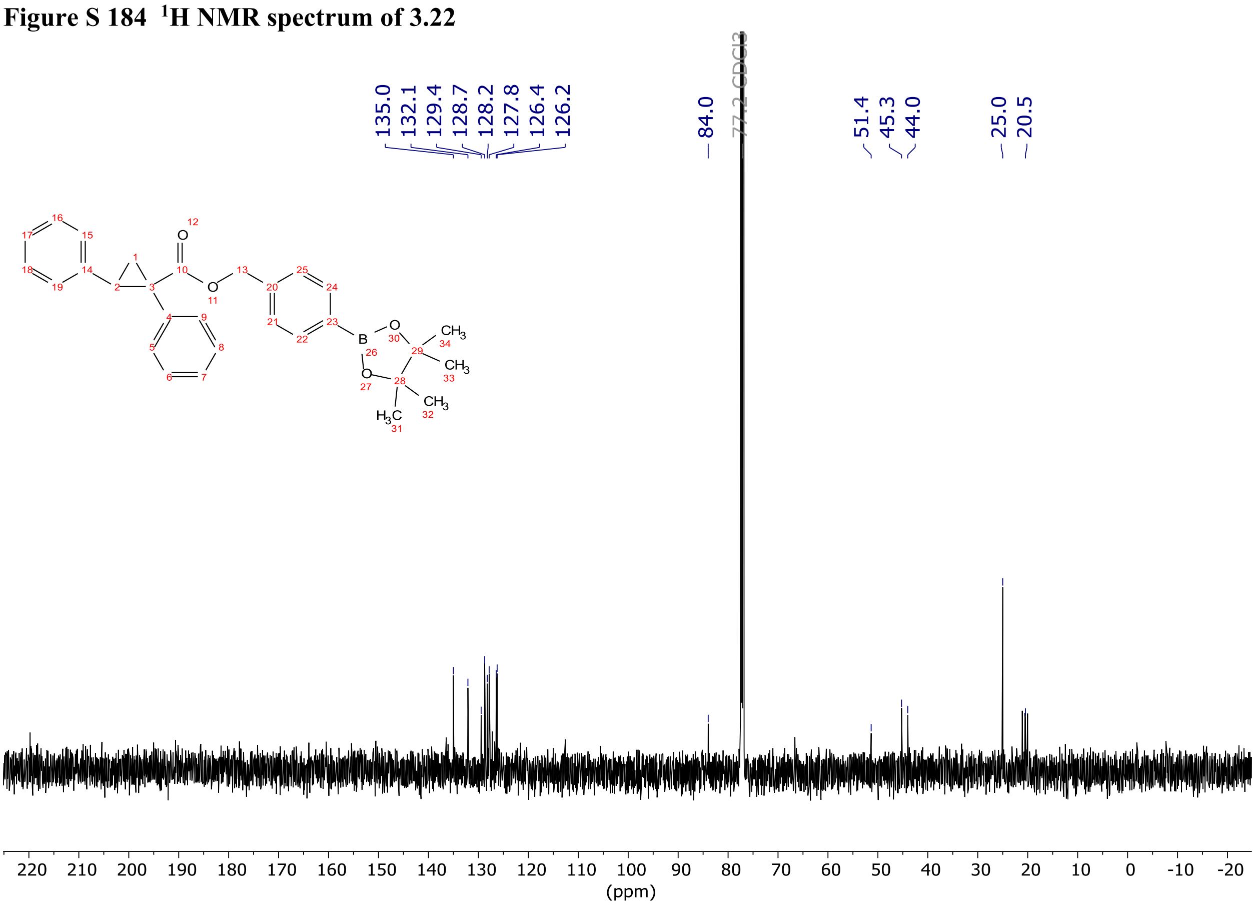

Figure S 185 ^{13}C NMR spectrum of 3.22

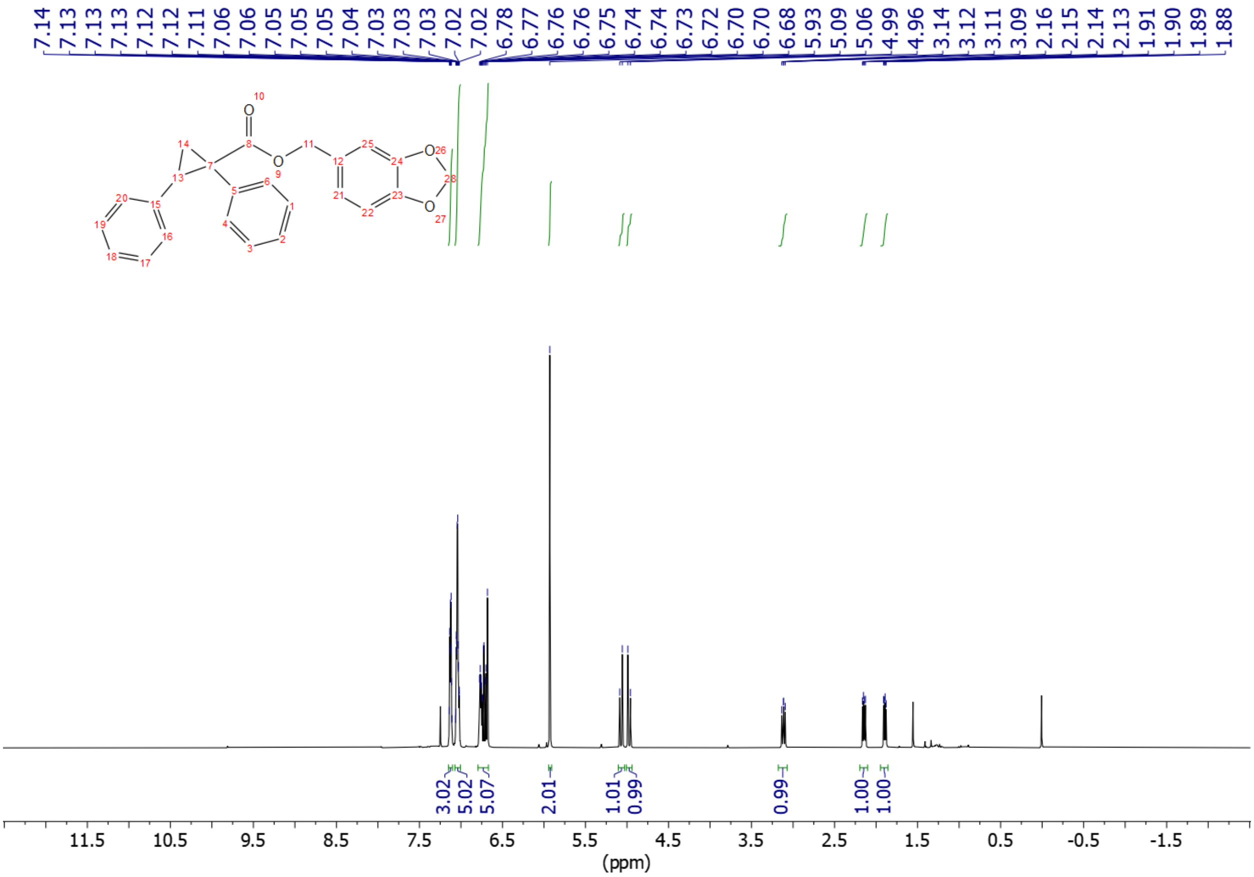

Figure S 186 ^{1}H NMR spectrum of 3.23

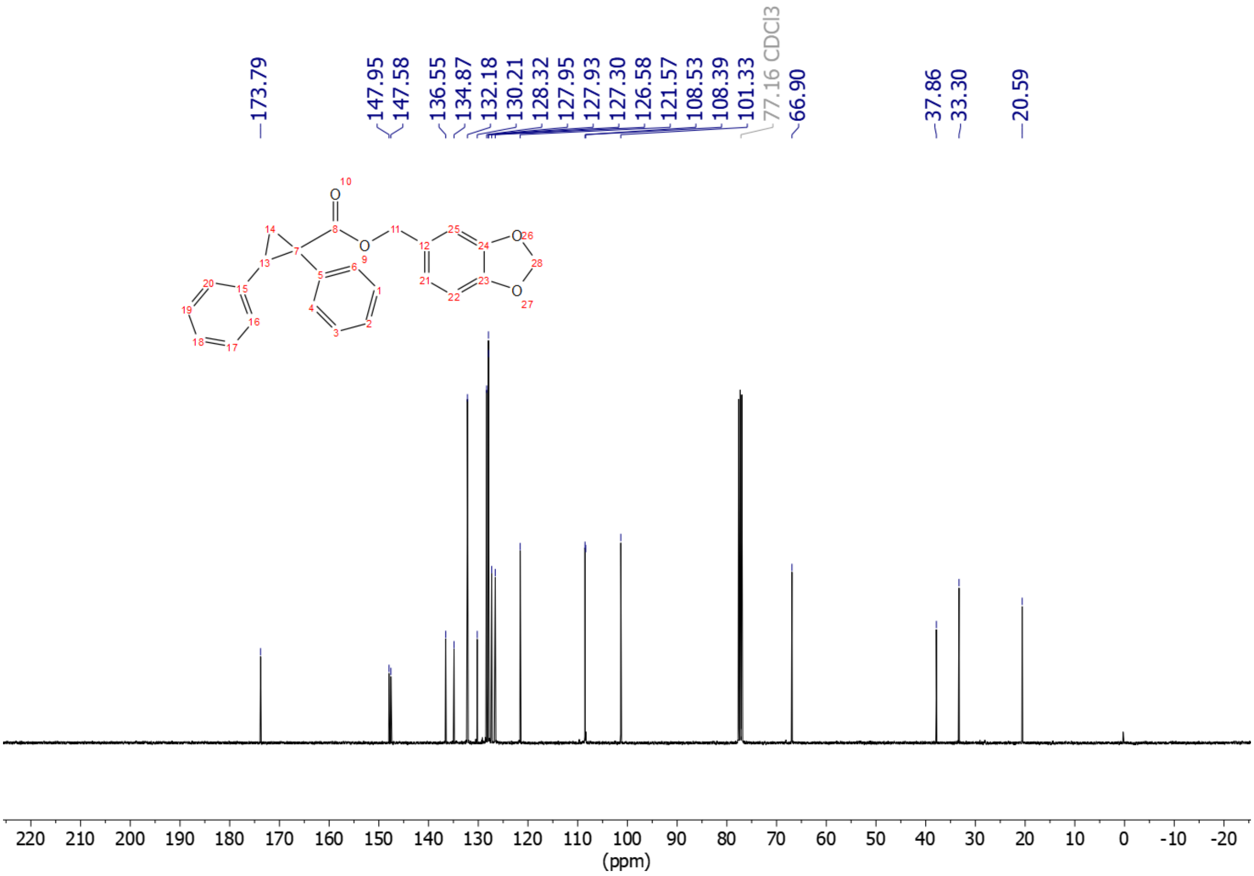

Figure S 187 ^{13}C NMR spectrum of 3.23

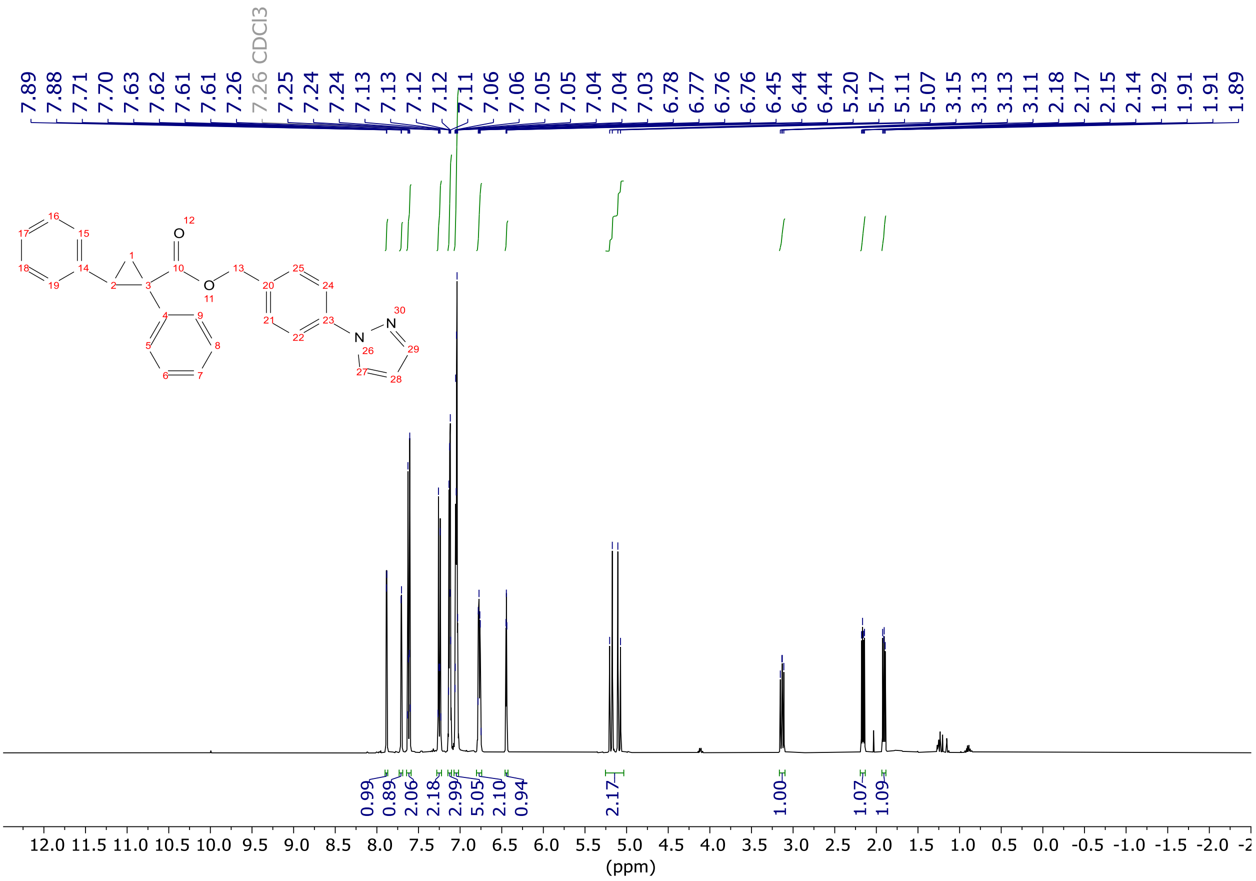

Figure S 188 ^{1}H NMR spectrum of 3.24

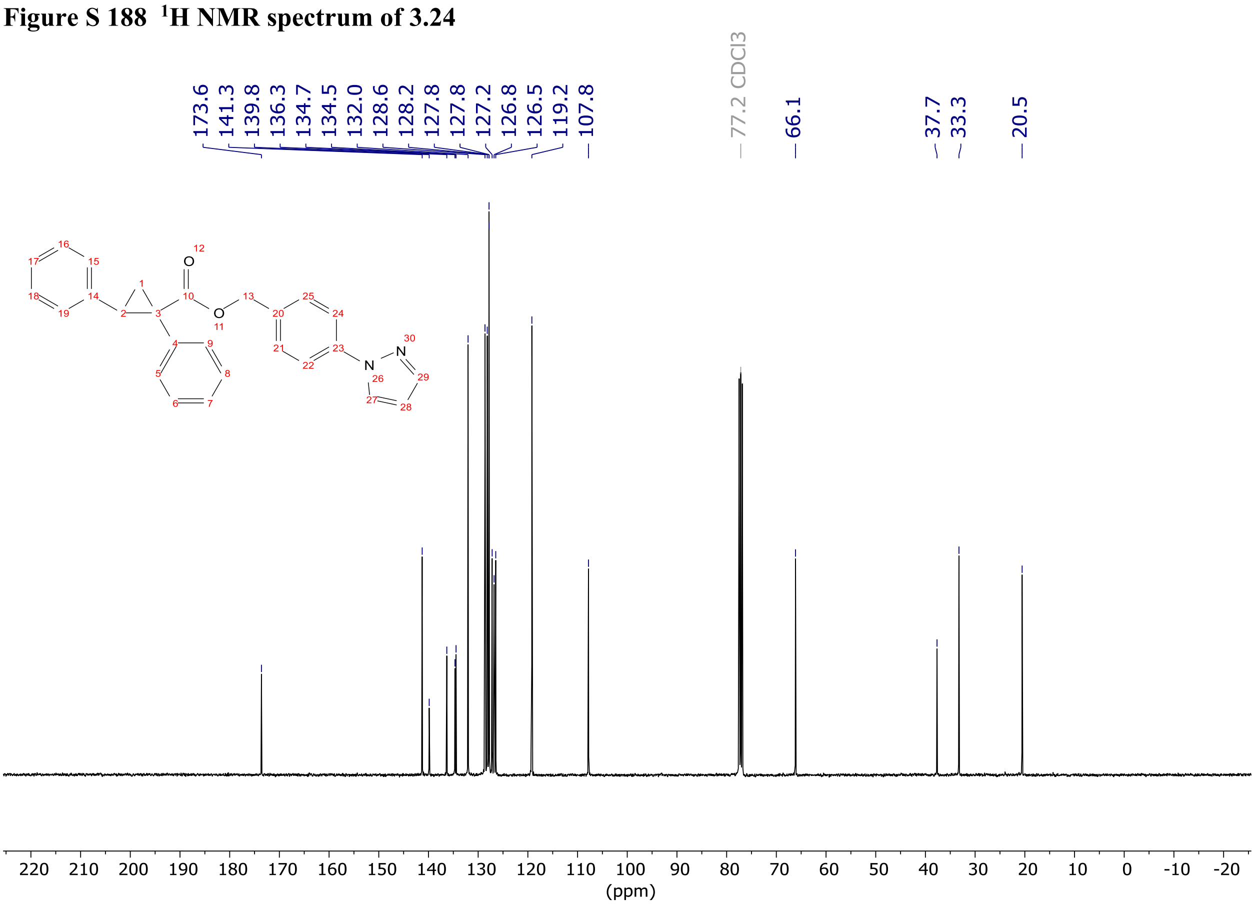

Figure S 189 ^{13}C NMR spectrum of 3.24

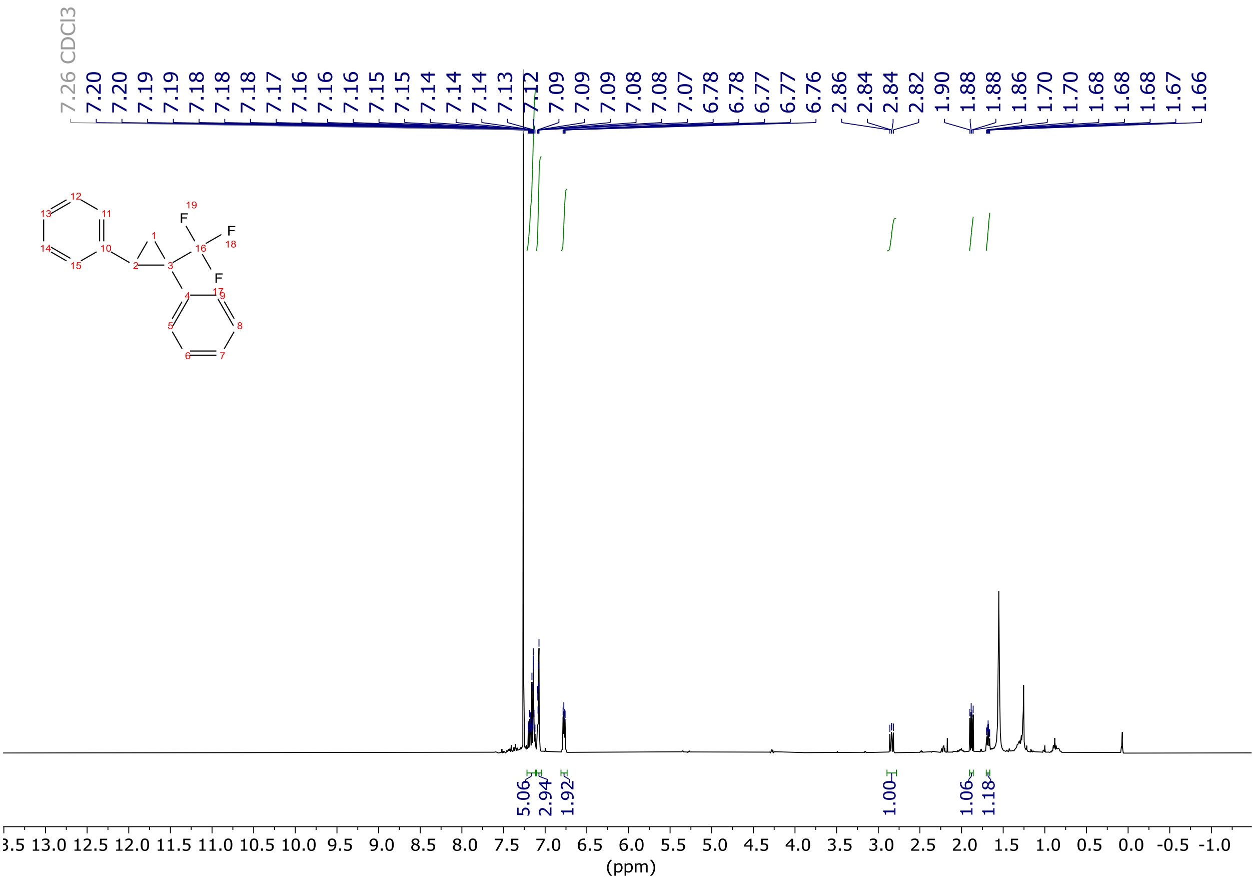

Figure S 190 ¹H NMR spectrum of 3.25

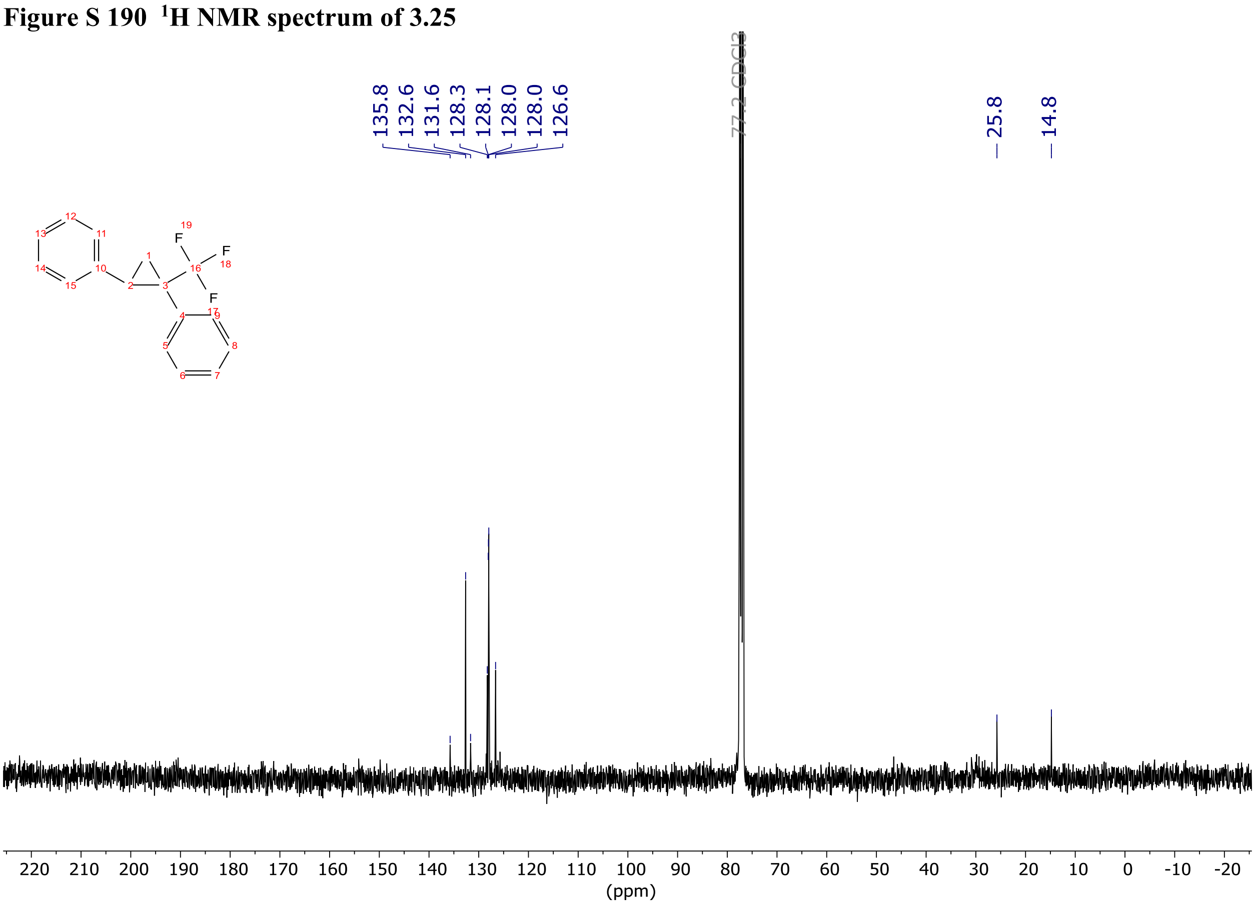

Figure S 191 ¹³C NMR spectrum of 3.25

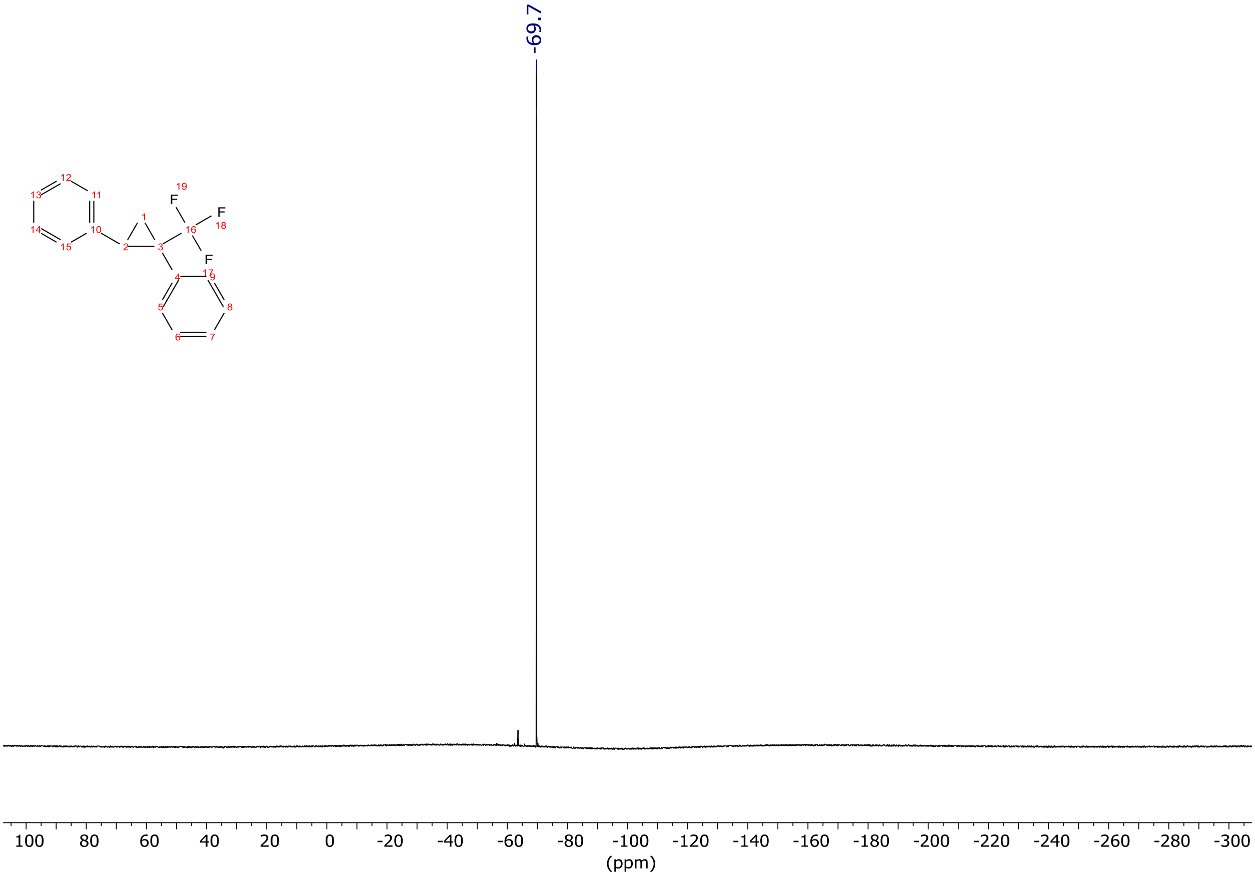

Figure S 192 ^{19}**F NMR spectrum of 3.25**

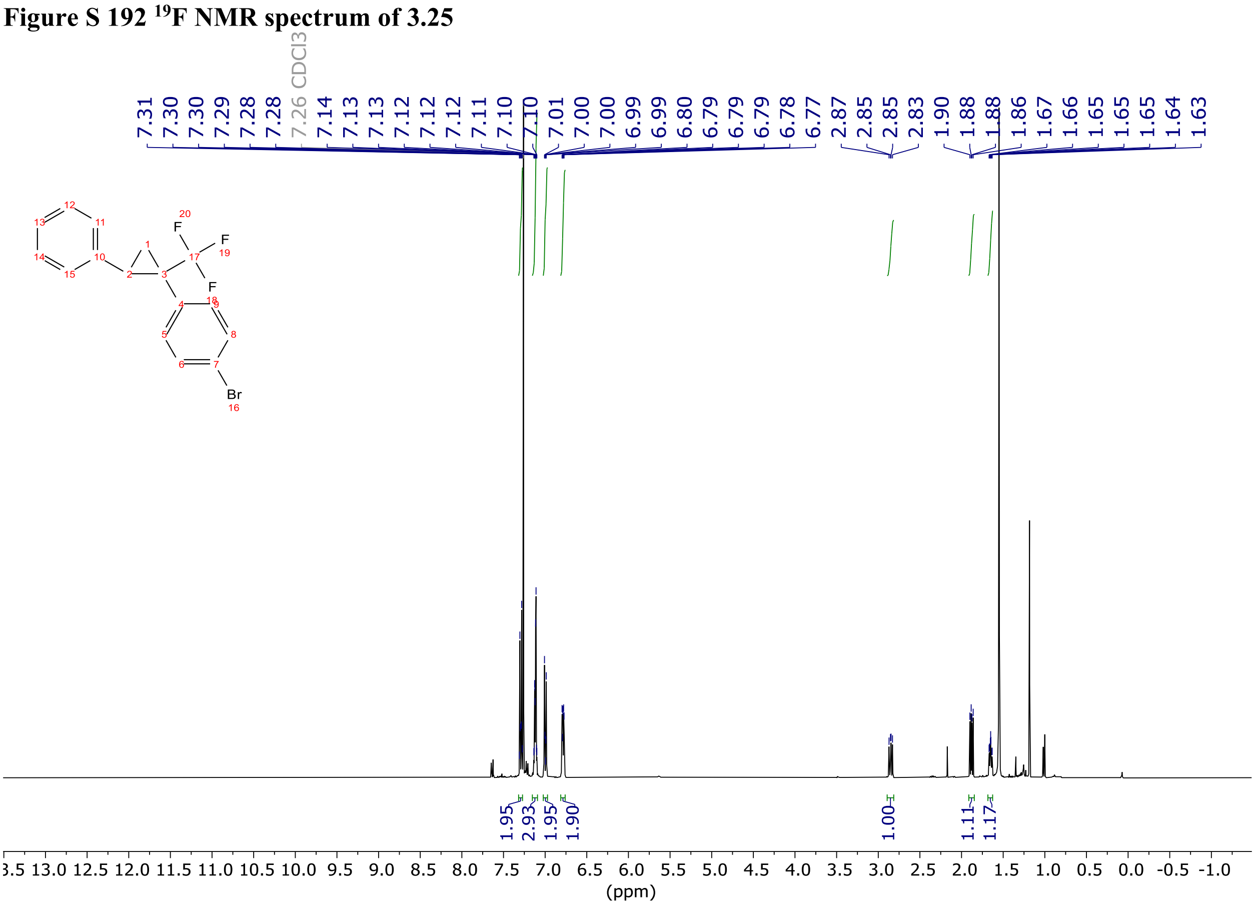

Figure S 193 ^{1}**H NMR spectrum of 3.26**

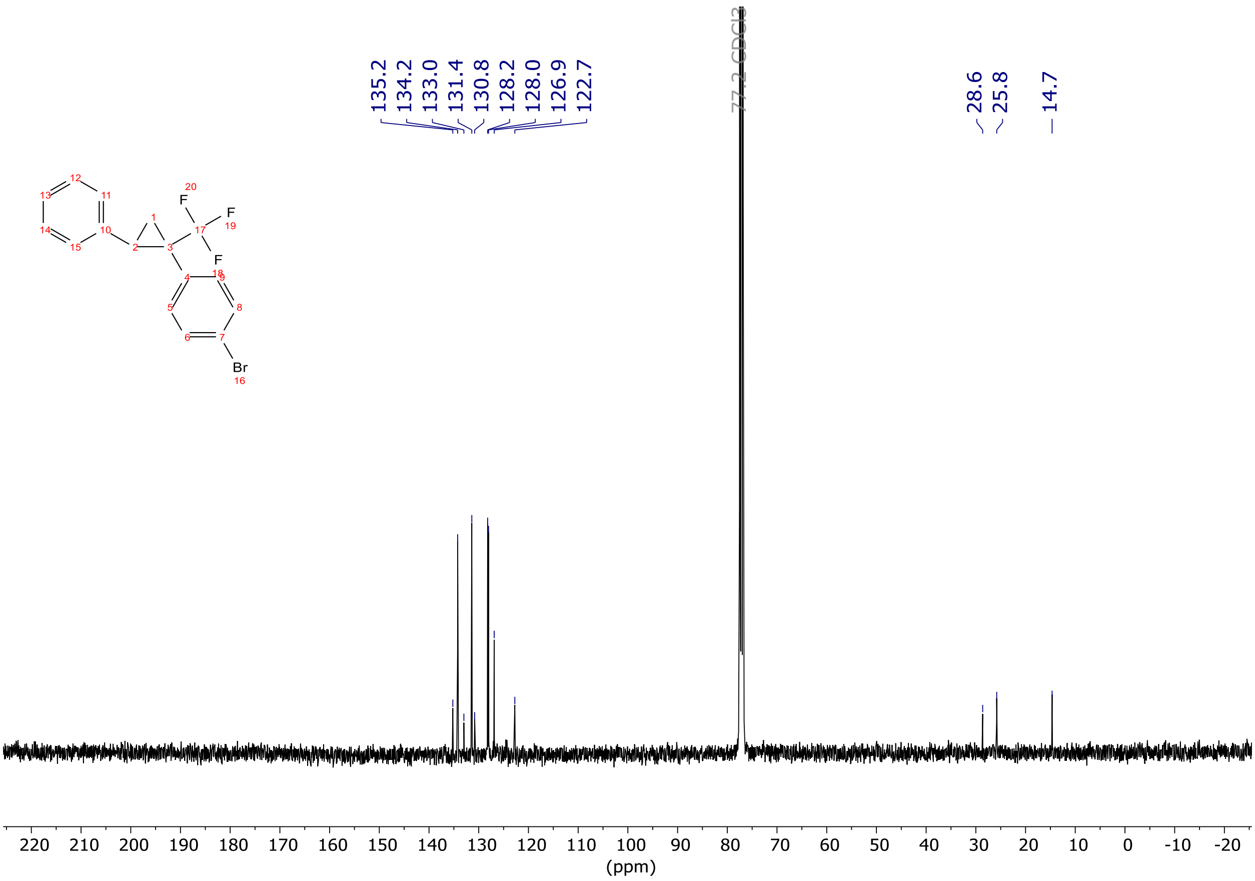

Figure S 194 ^{13}C NMR spectrum of 3.26

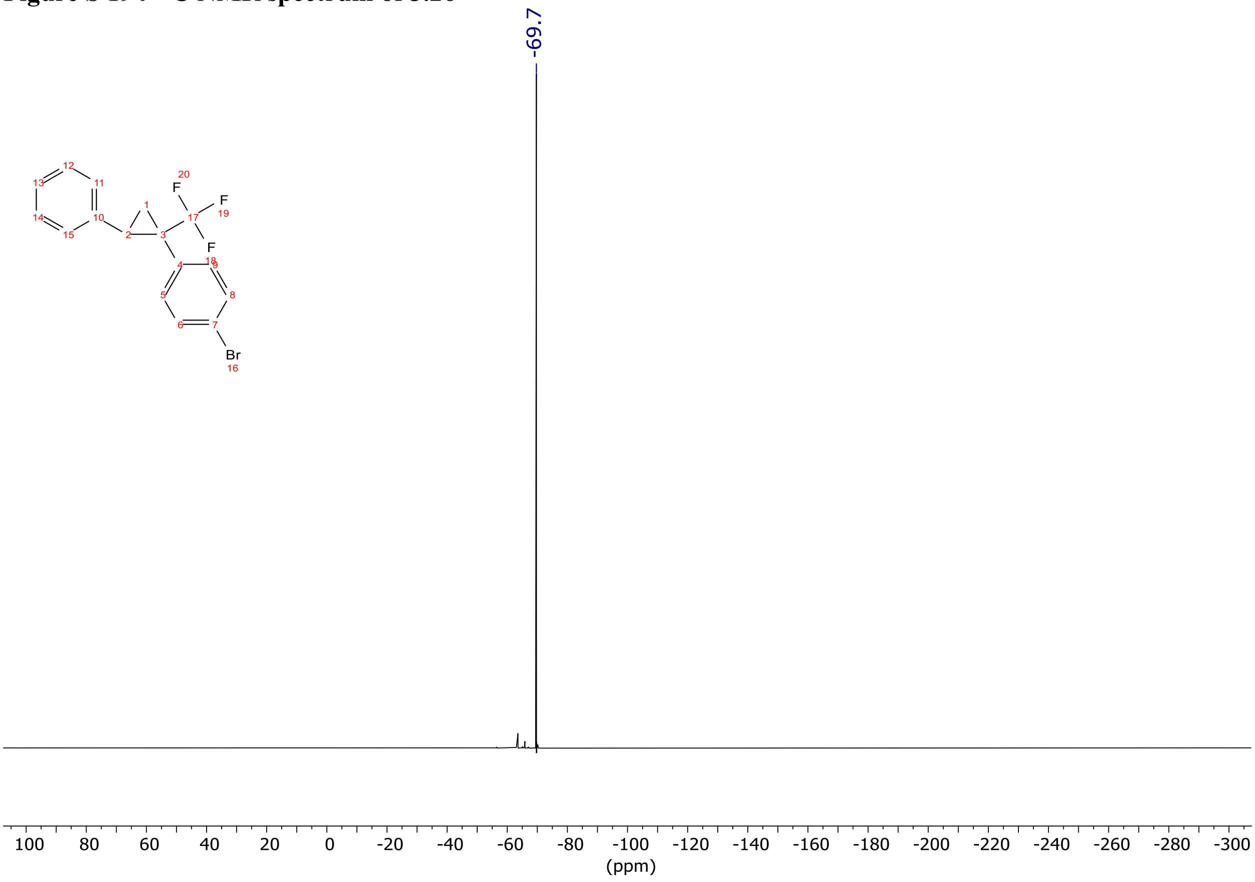

Figure S 195 ^{19}F NMR spectrum of 3.26

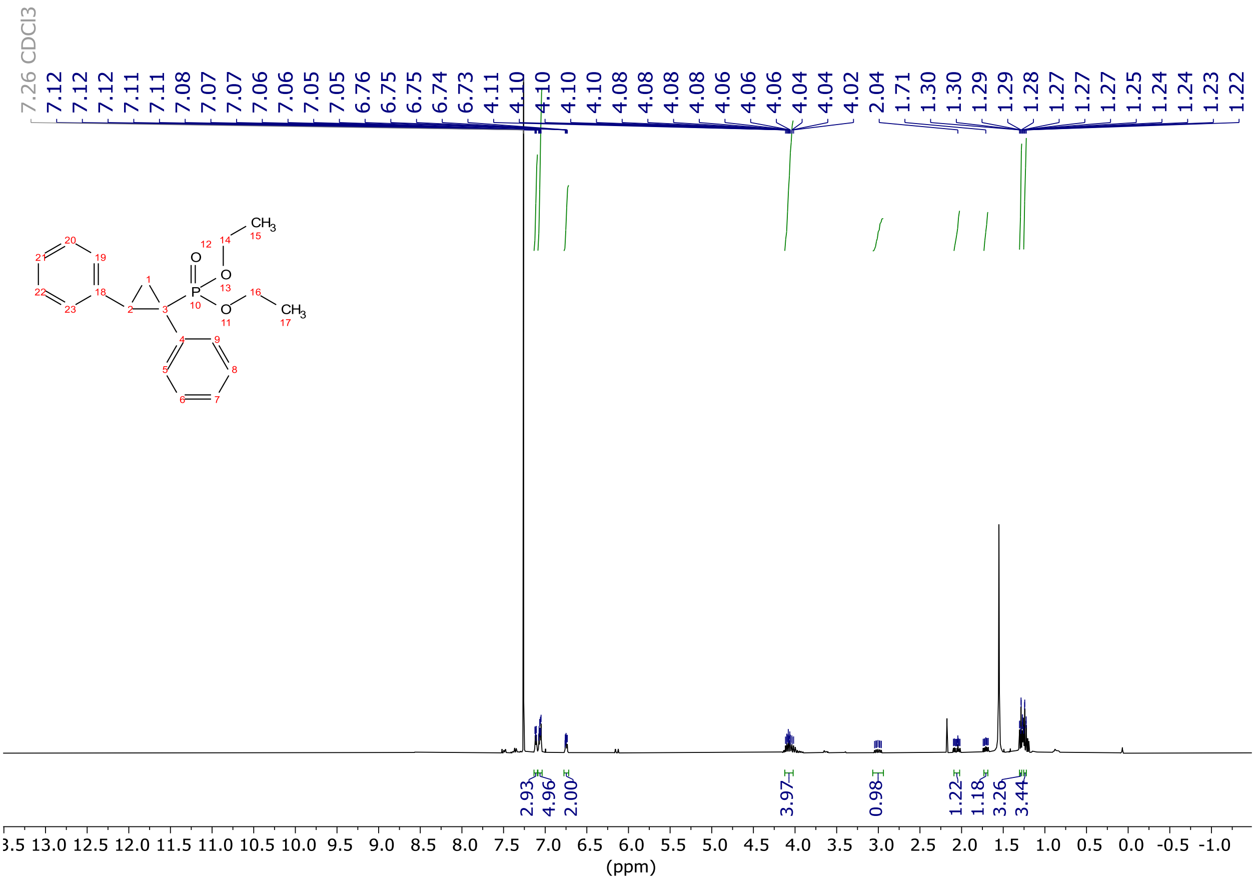

Figure S 196 ^{1}H NMR spectrum of 3.27

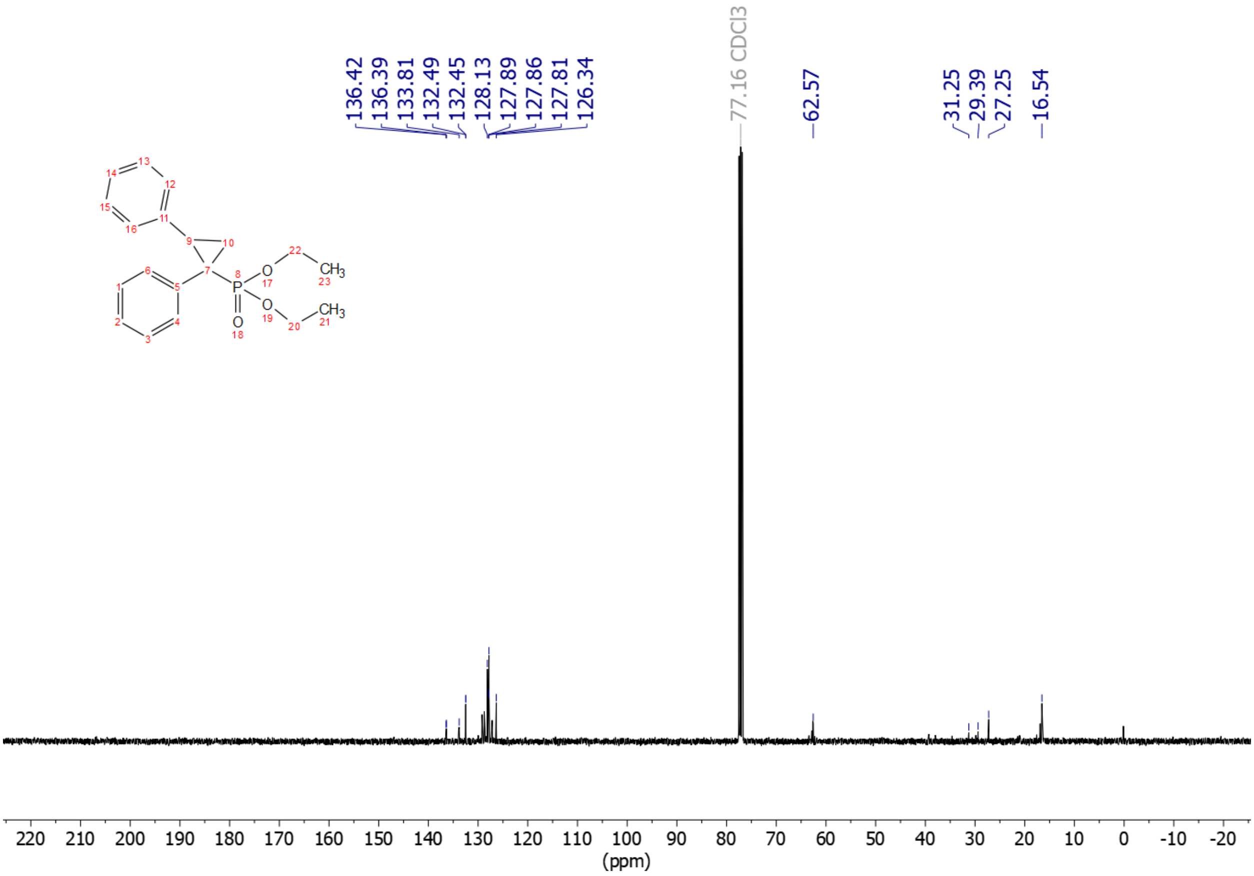

Figure S 197 ^{13}C NMR spectrum of 3.27

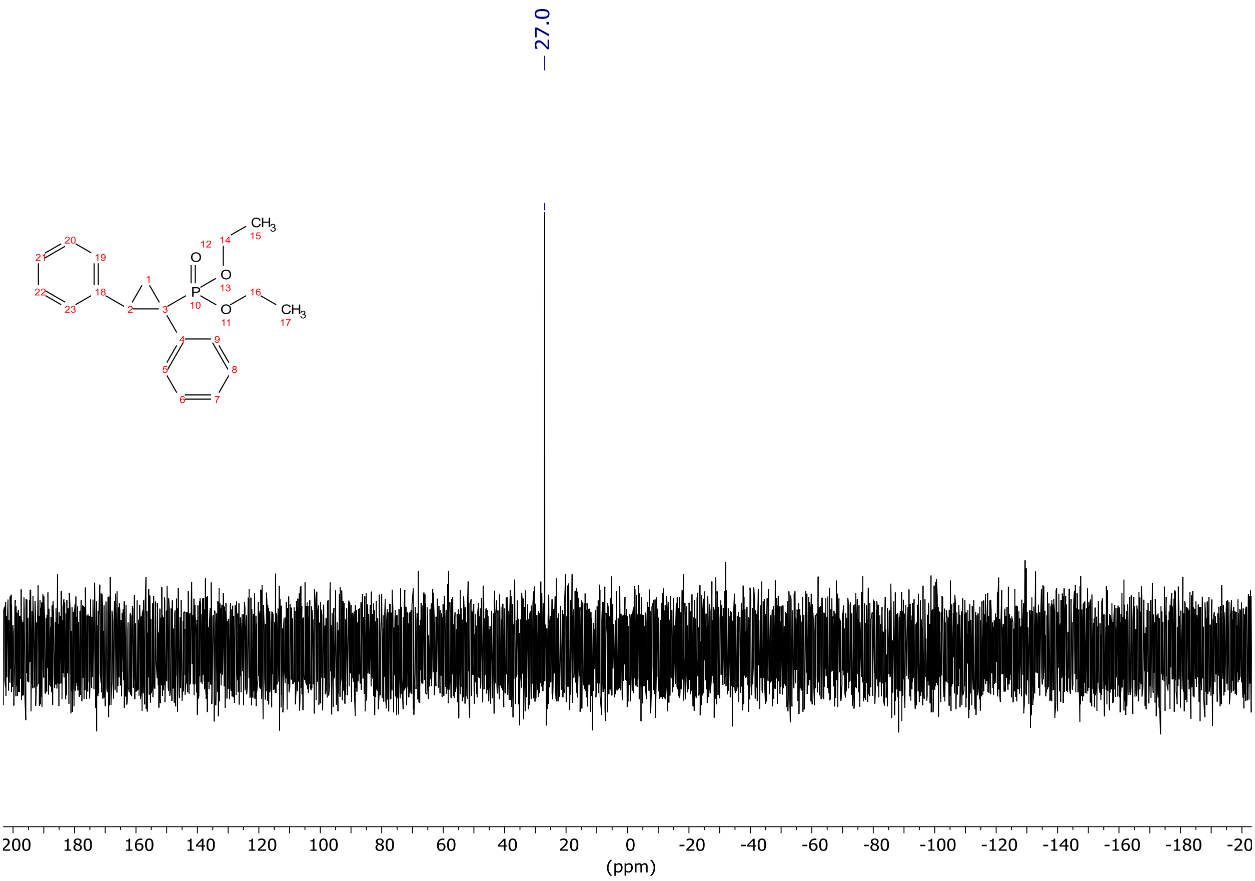

Figure S 198 ^{31}P NMR spectrum of 3.27

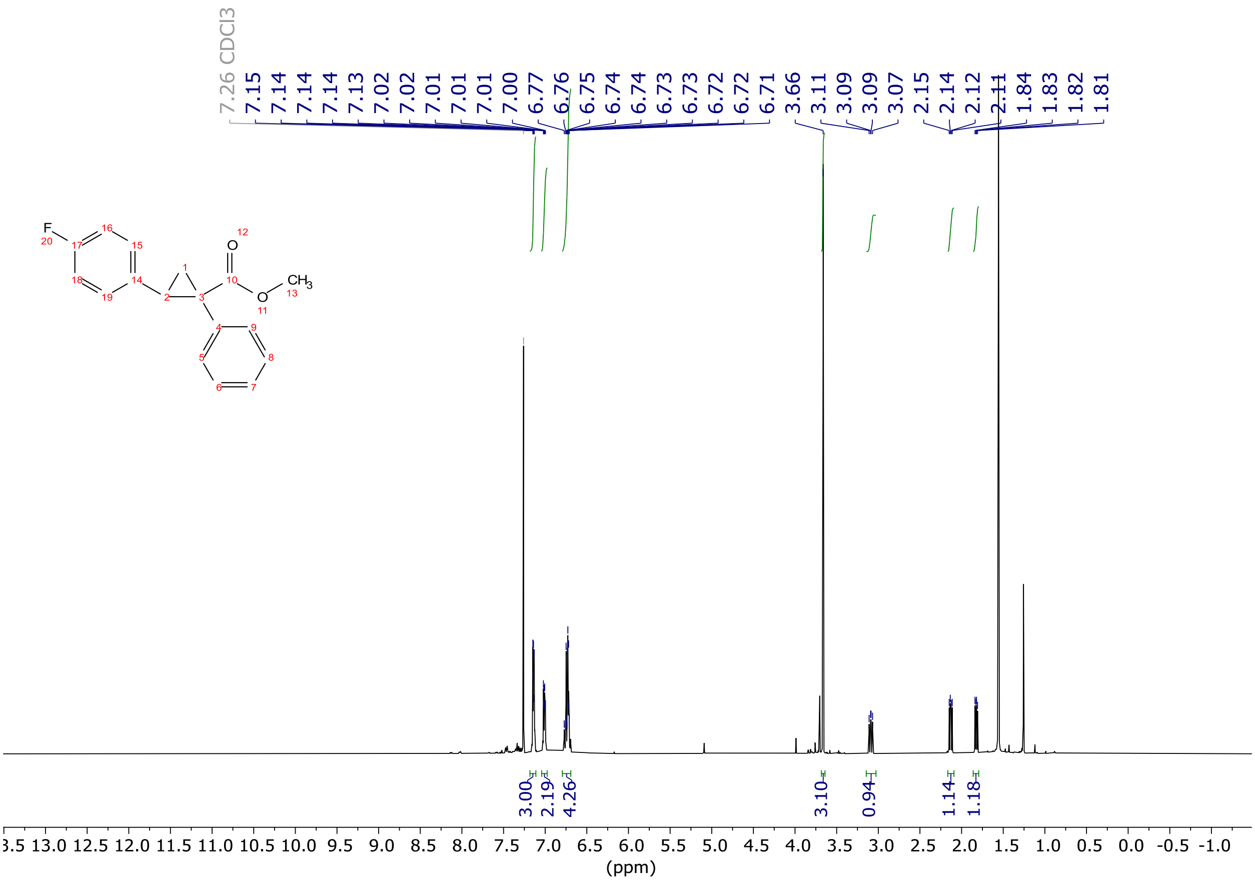

Figure S 199 ^{1}H NMR spectrum of 3.39

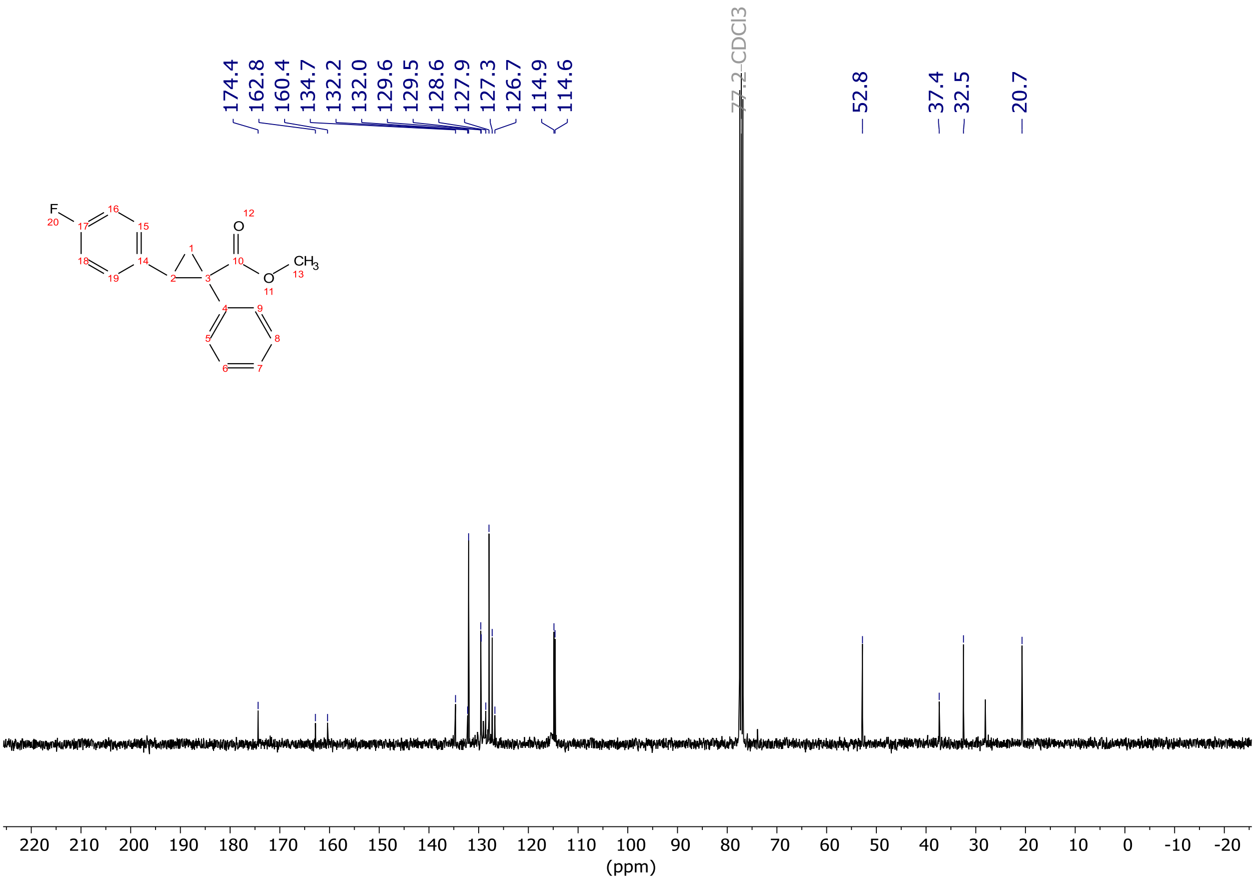

Figure S 200 ^{13}C NMR spectrum of 3.39

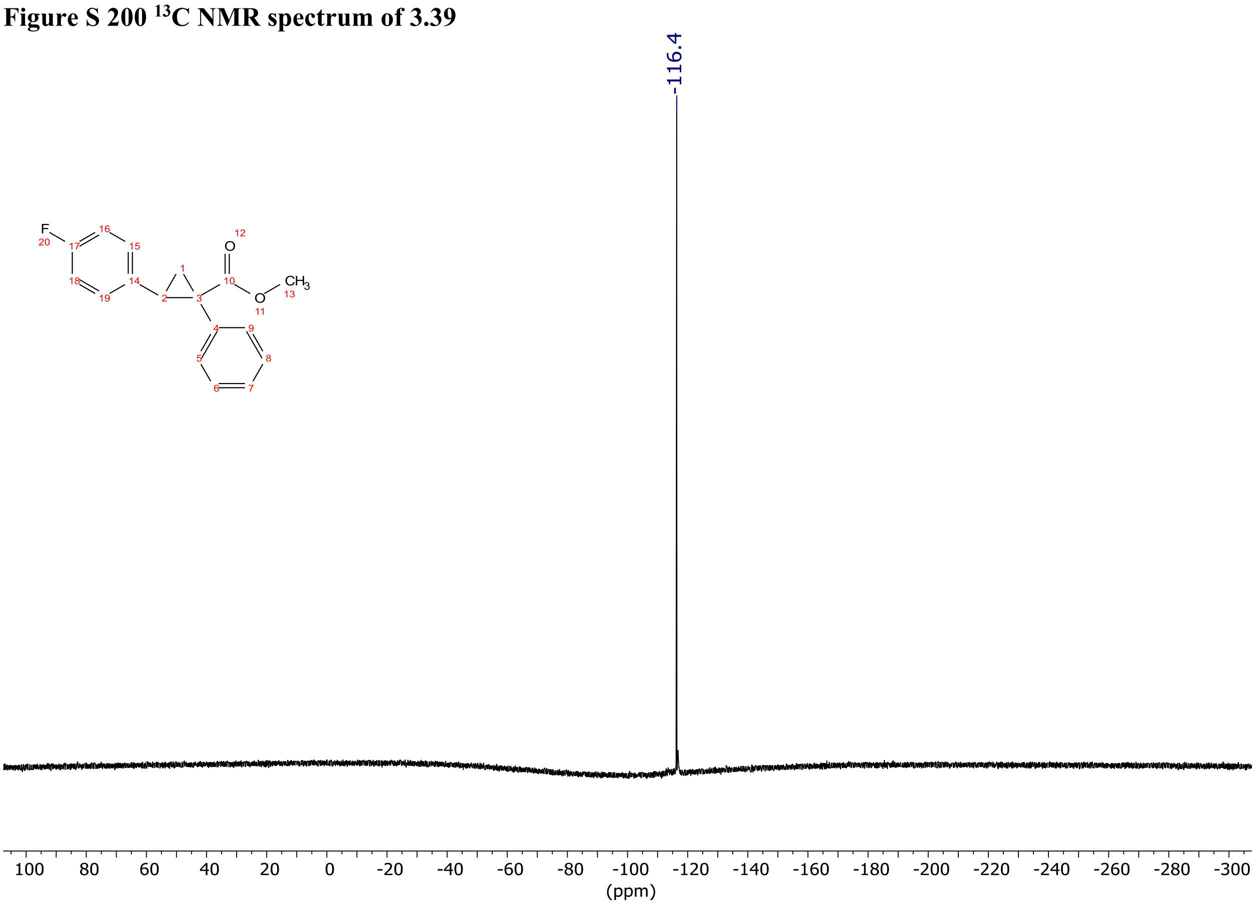

Figure S 201 ^{19}F NMR spectrum of 3.39

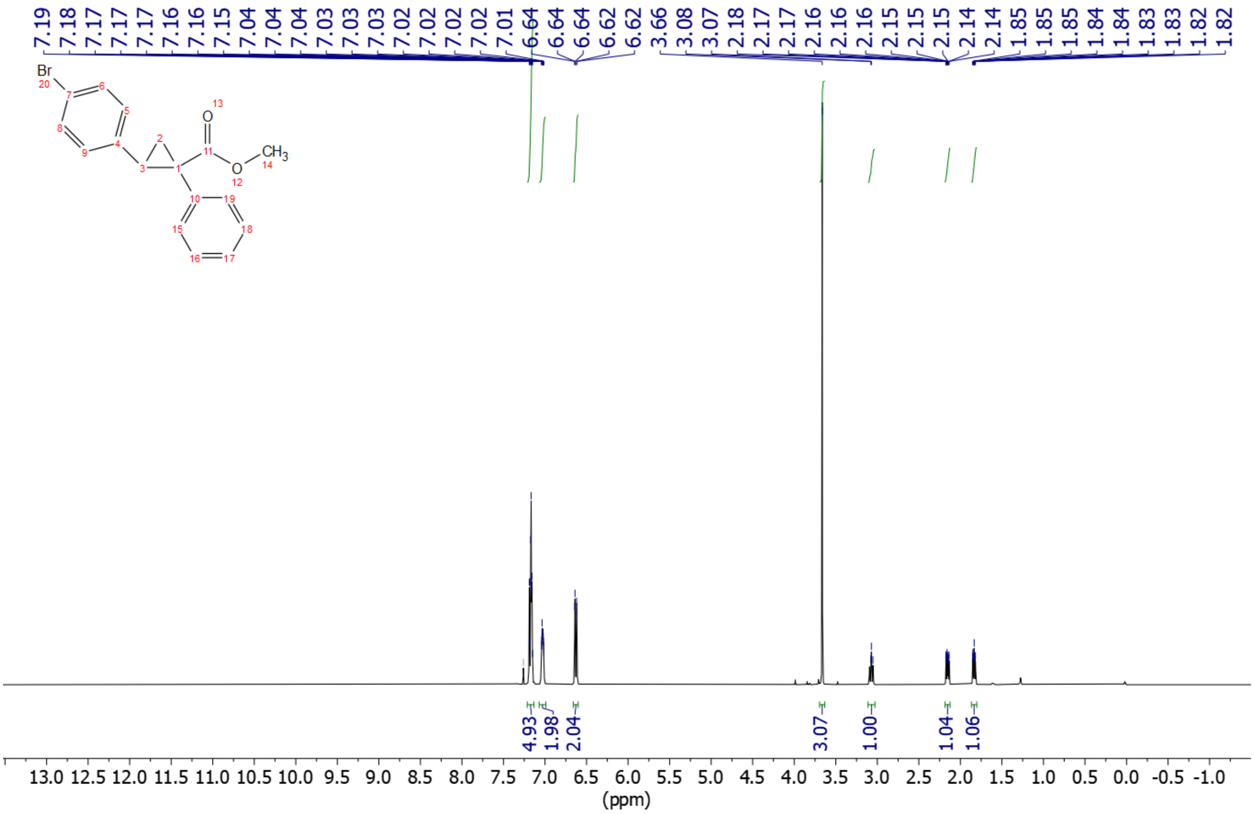

Figure S 202 ^{1}H NMR spectrum of 3.40

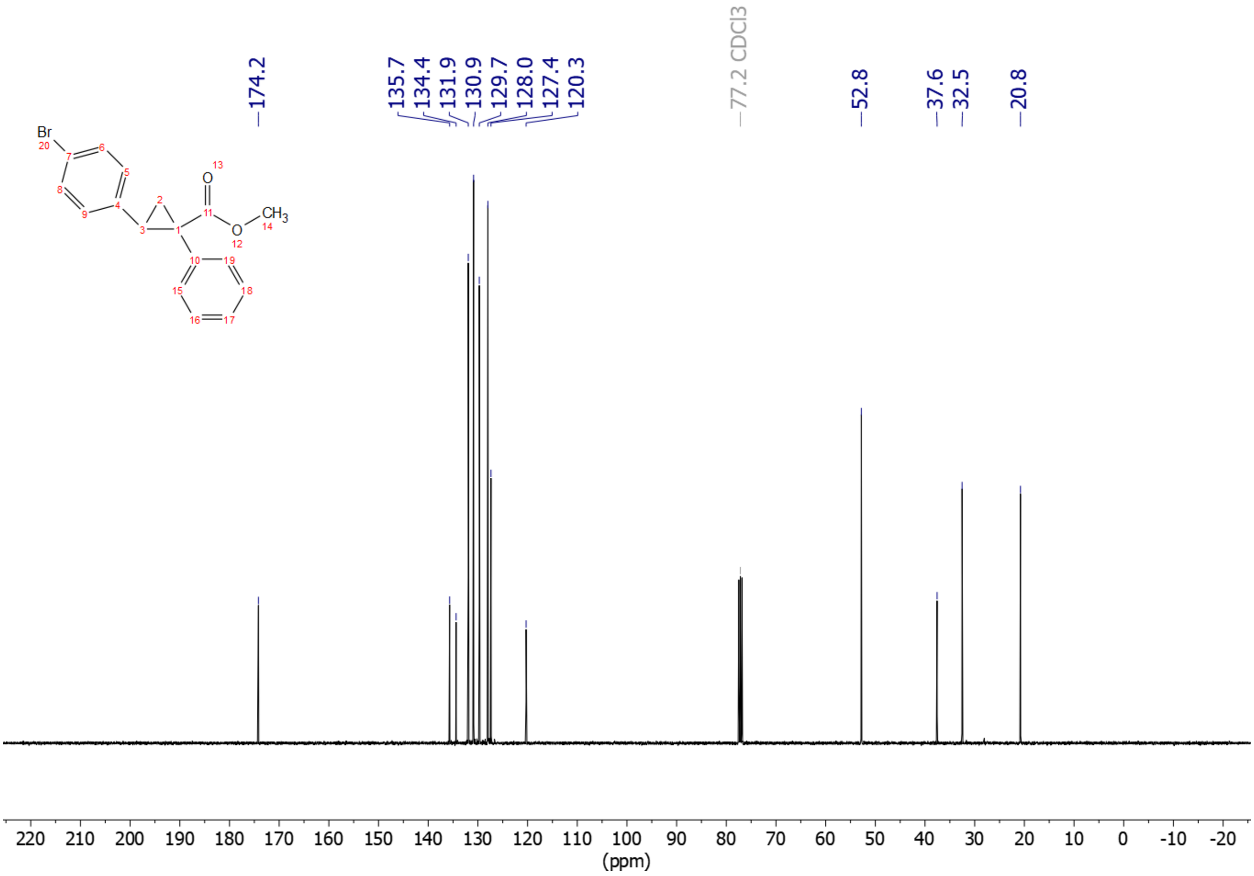

Figure S 203 ^{13}C NMR spectrum of 3.40

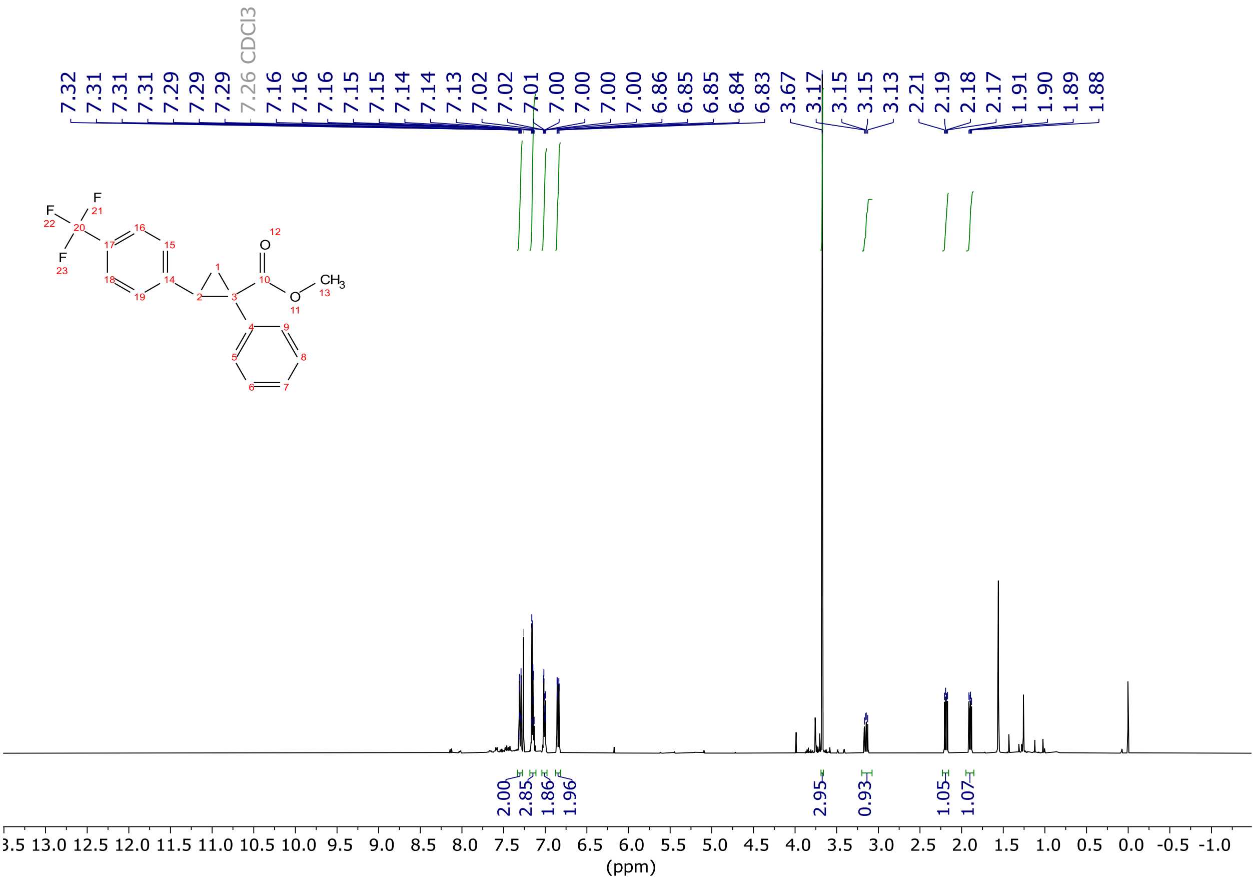

Figure S 204 ^{1}H NMR spectrum of 3.41

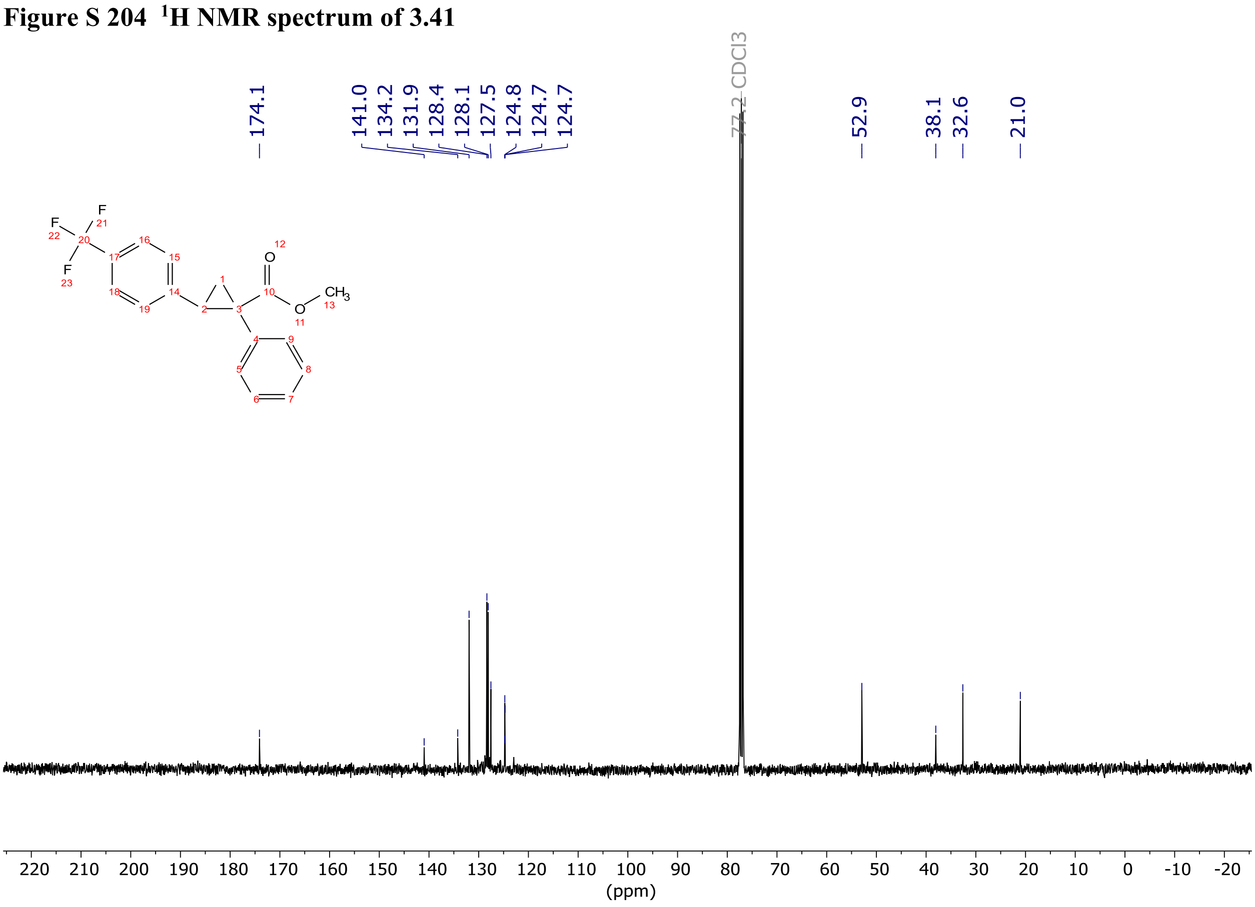

Figure S 205 ^{13}C NMR spectrum of 3.41

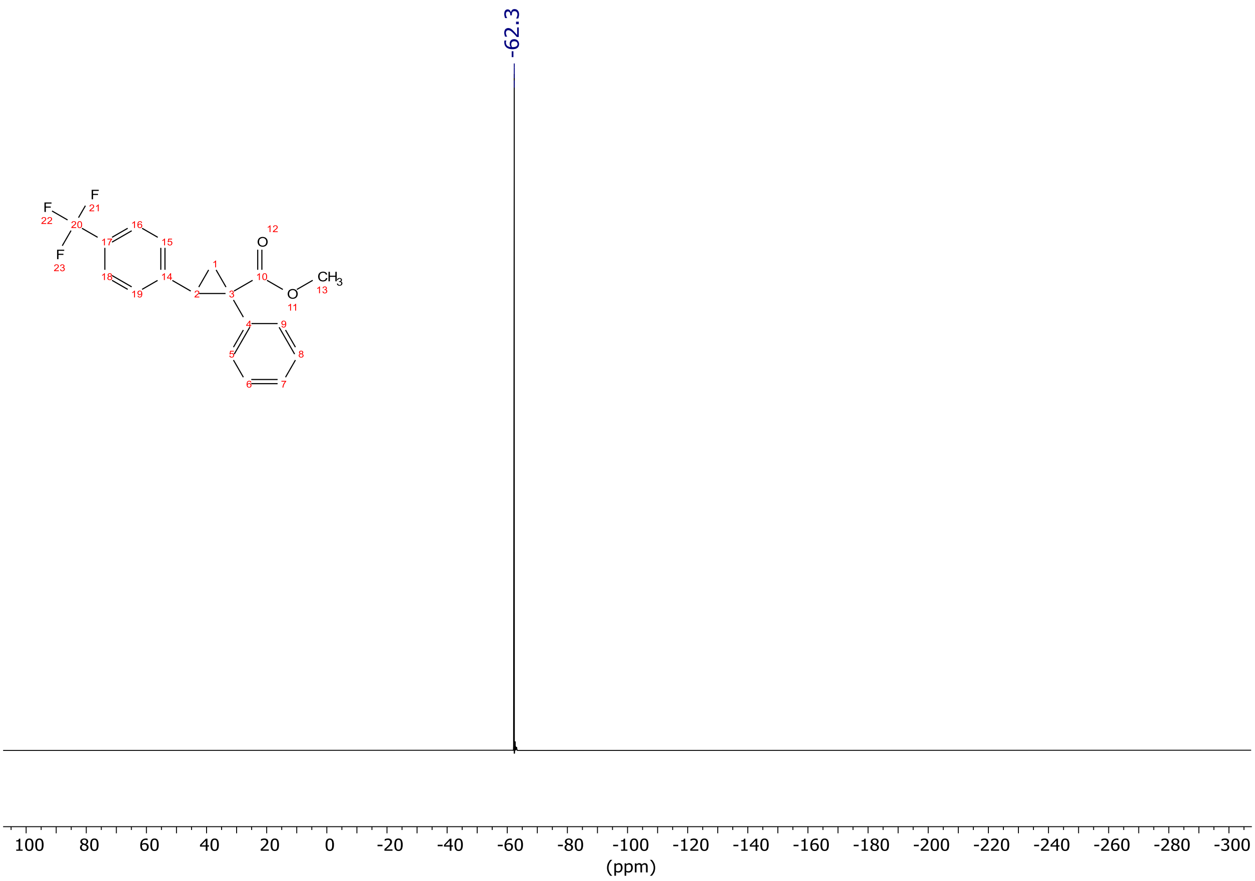

Figure S 206 ^{19}F NMR spectrum of 3.41

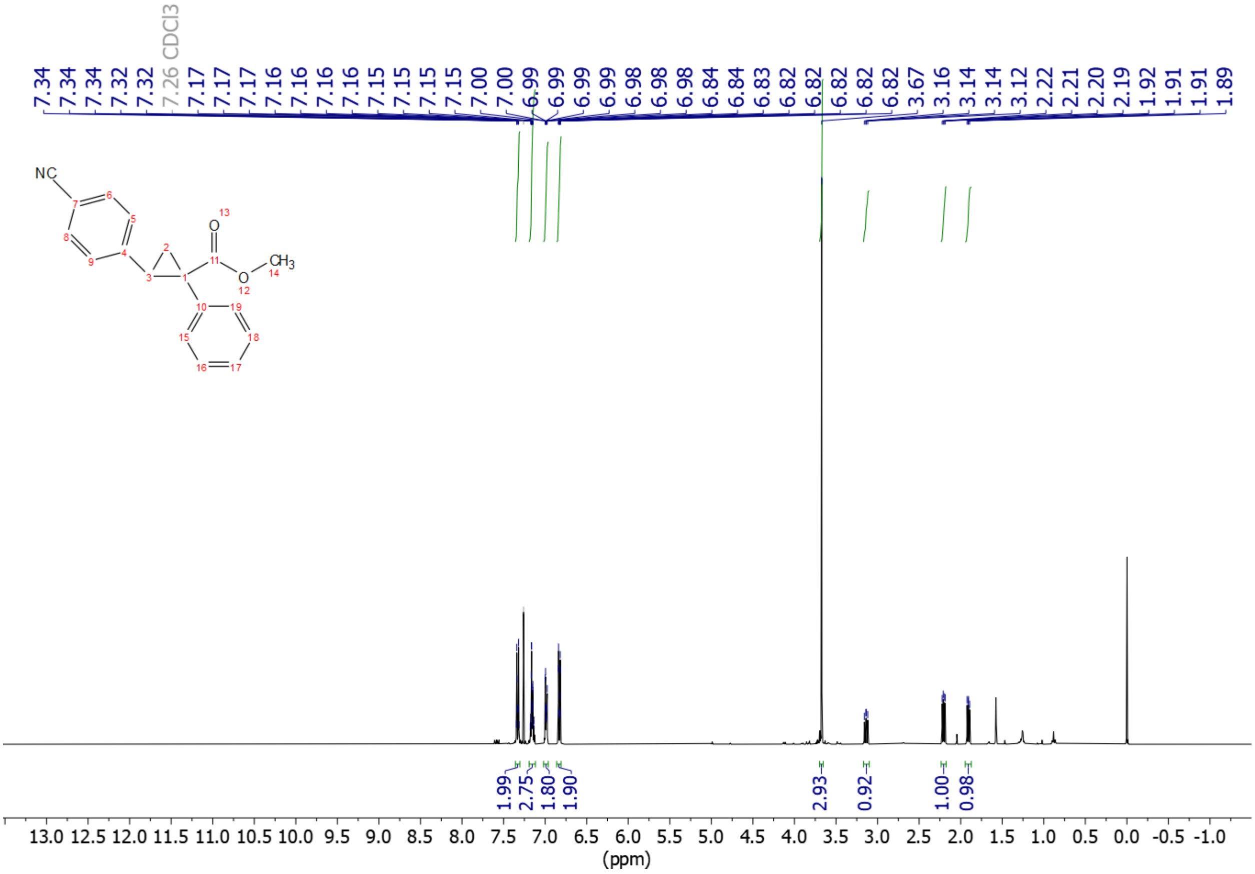

Figure S 207 ^{1}H NMR spectrum of 3.42

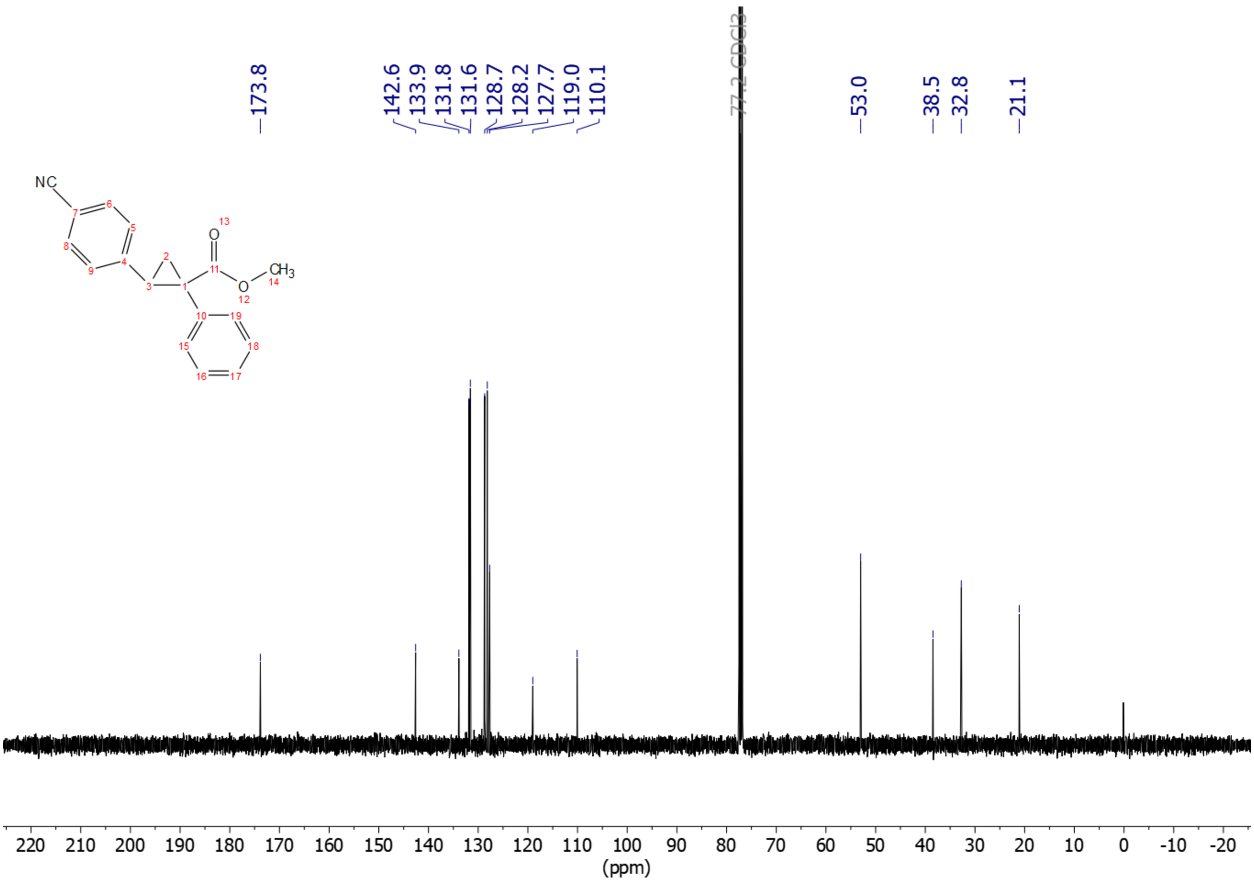

Figure S 208 ^{13}C NMR spectrum of 3.42

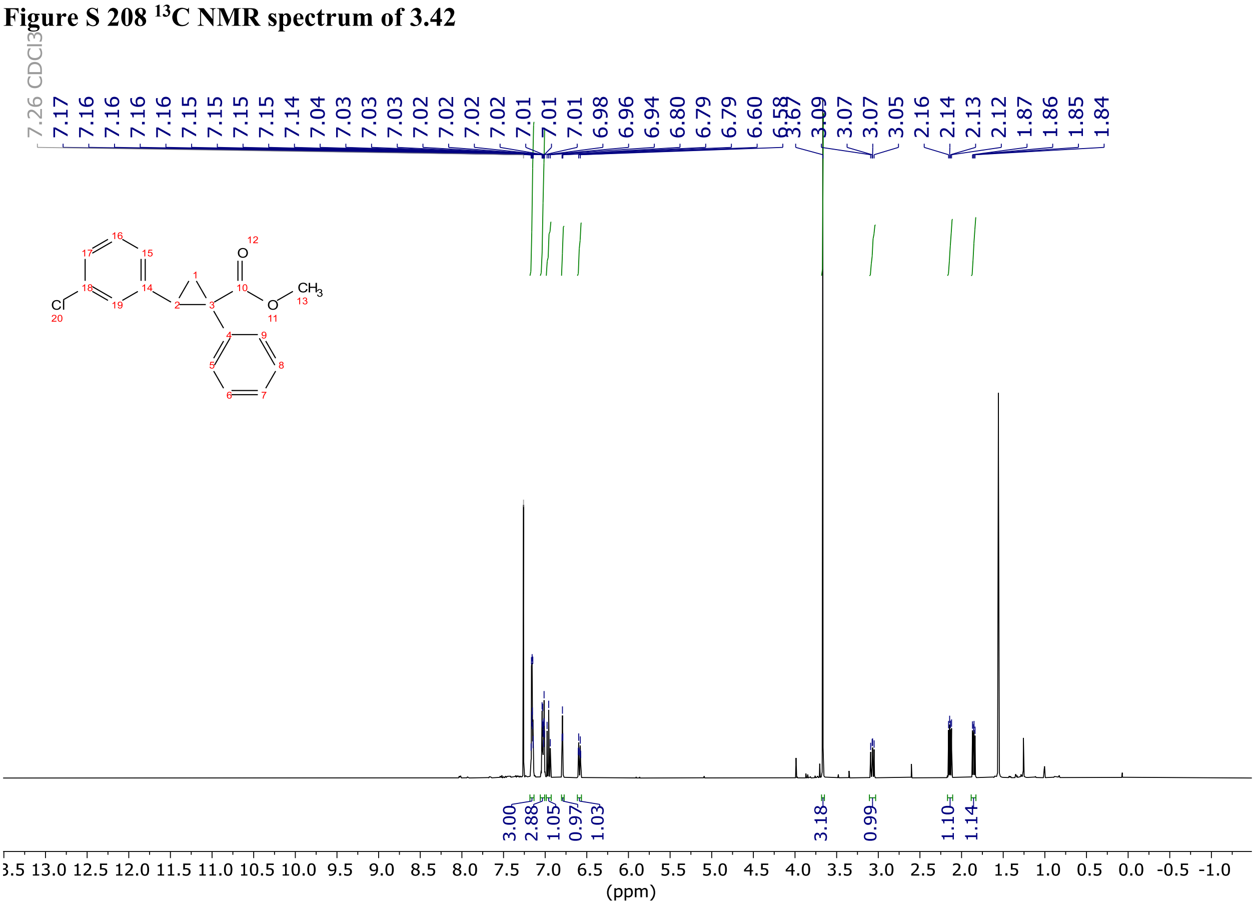

Figure S 209 ^{1}H NMR spectrum of 3.43

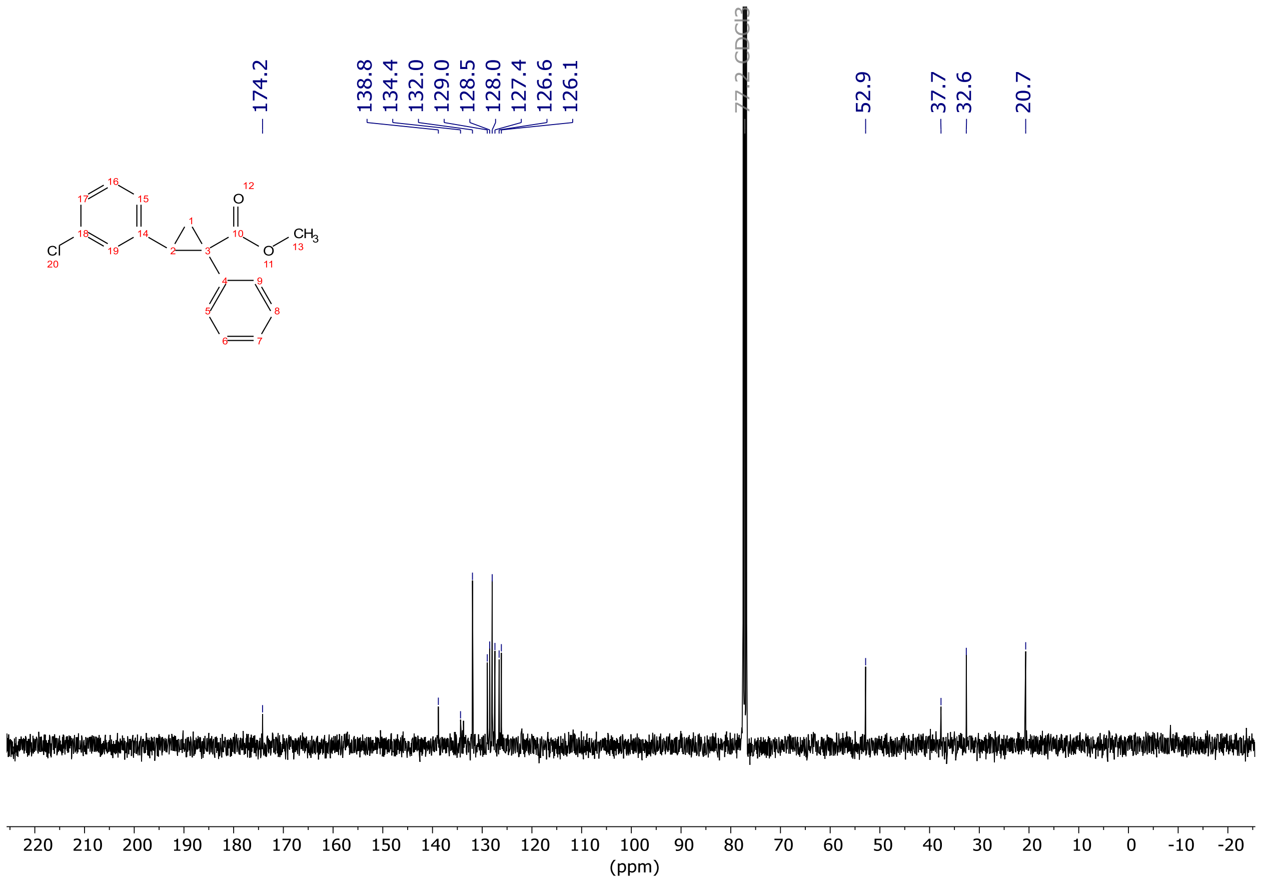

Figure S 210 ^{13}C NMR spectrum of 3.43

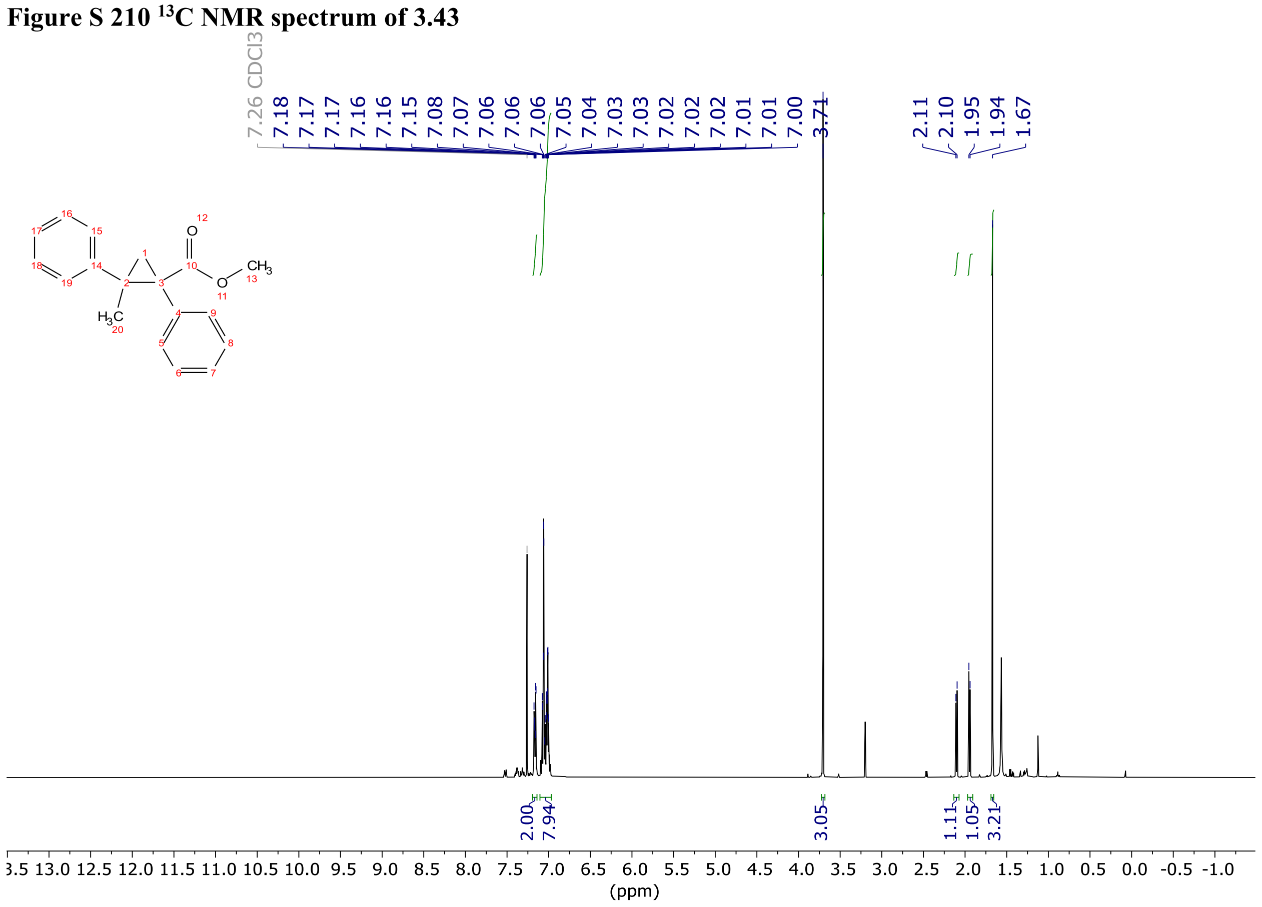

Figure S 211 ^{1}H NMR spectrum of 3.44

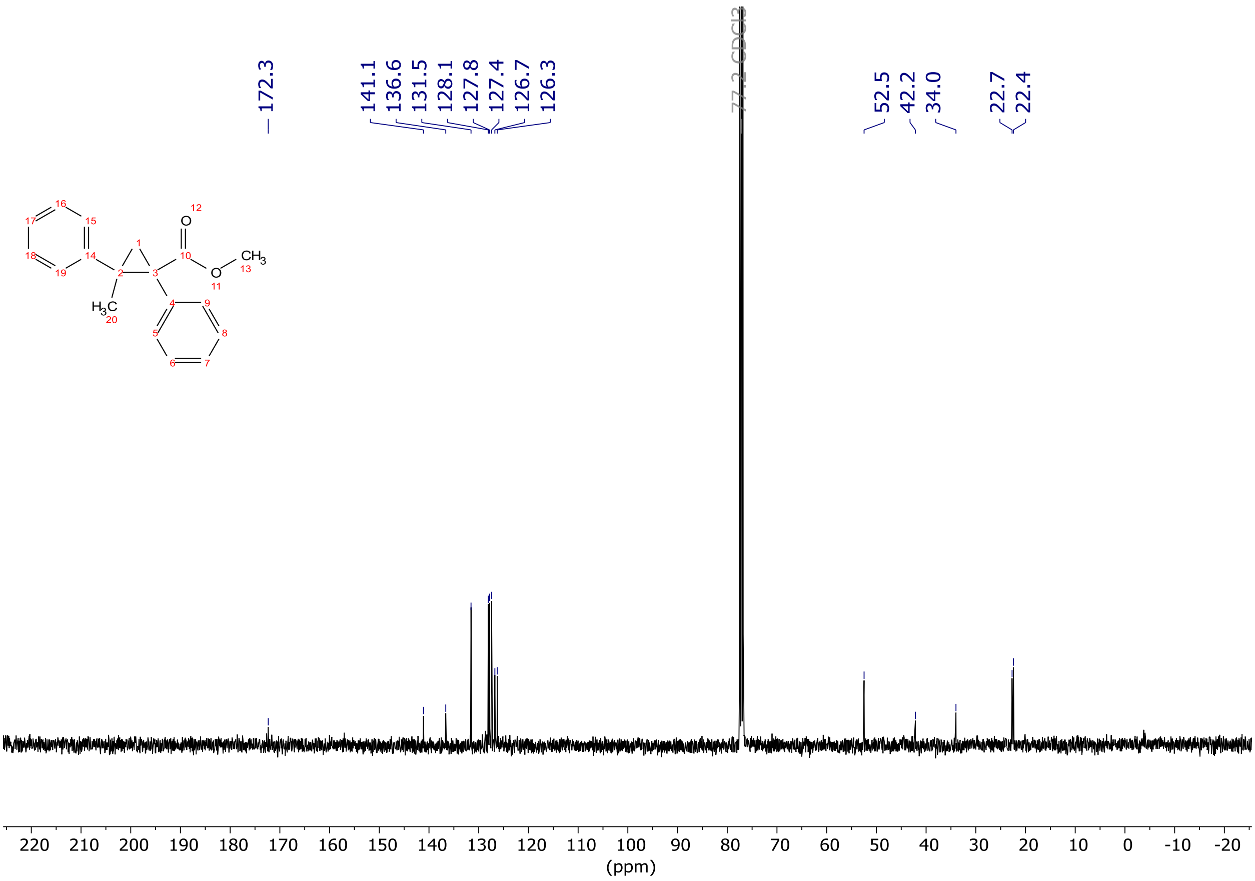

Figure S 212 ^{13}C NMR spectrum of 3.44

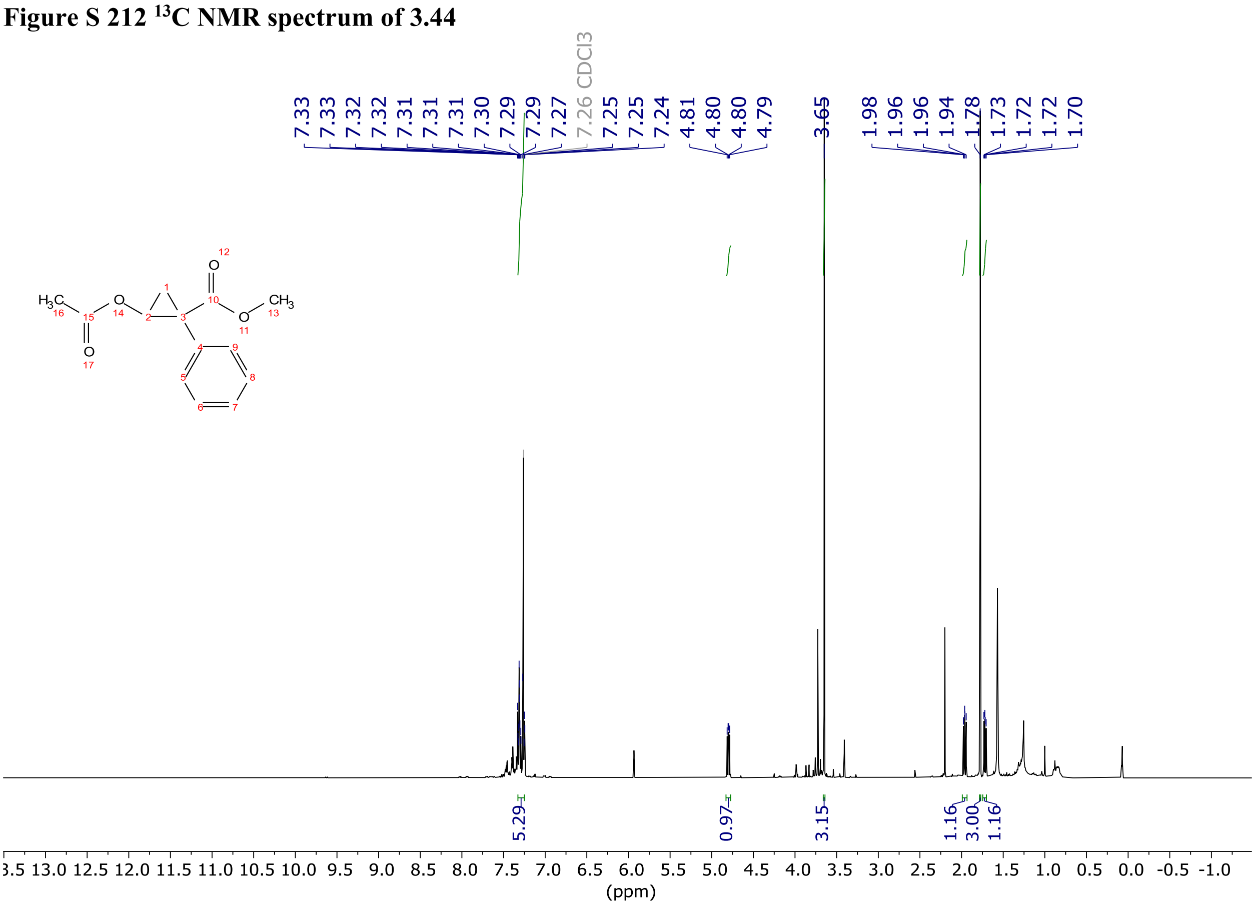

Figure S 213 ^{1}H NMR spectrum of 3.45

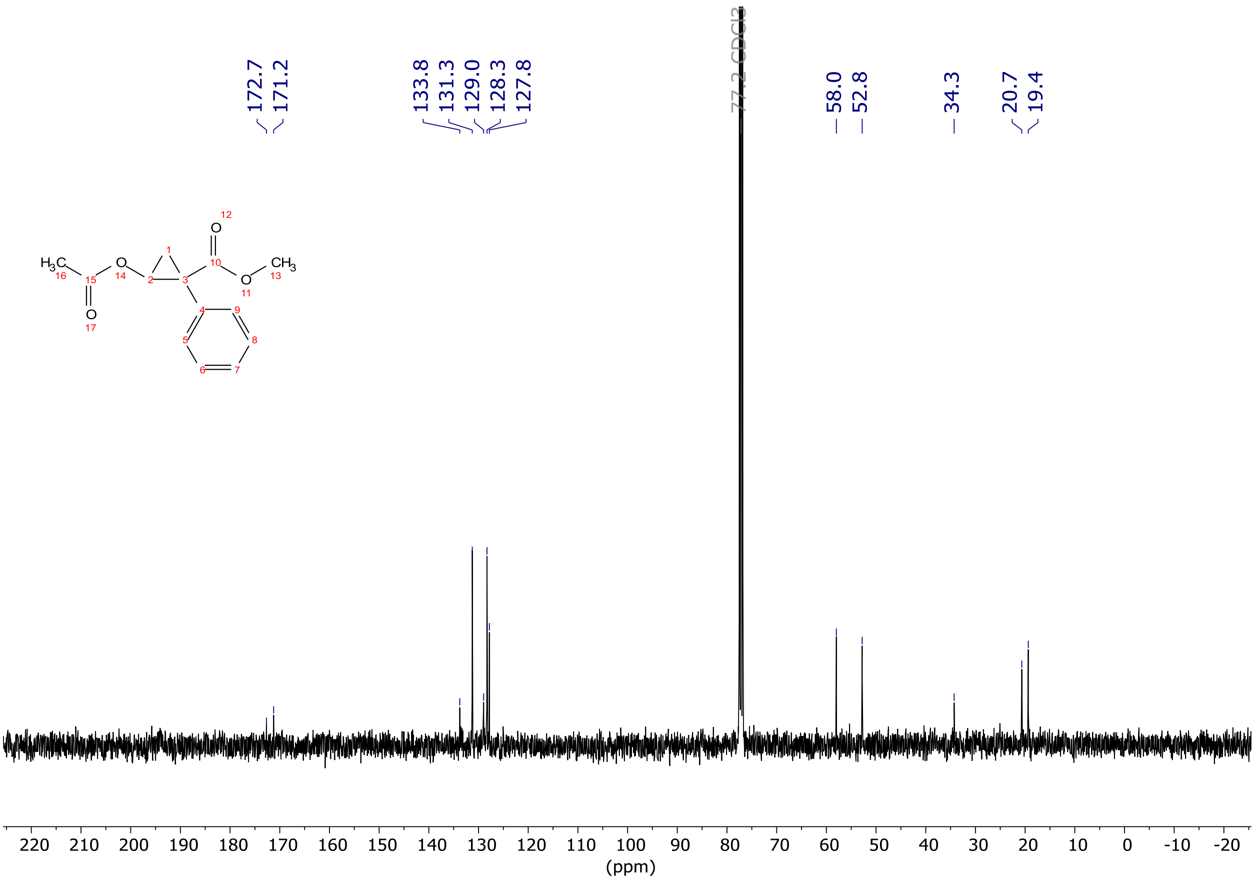

Figure S 214 ^{13}C NMR spectrum of 3.45

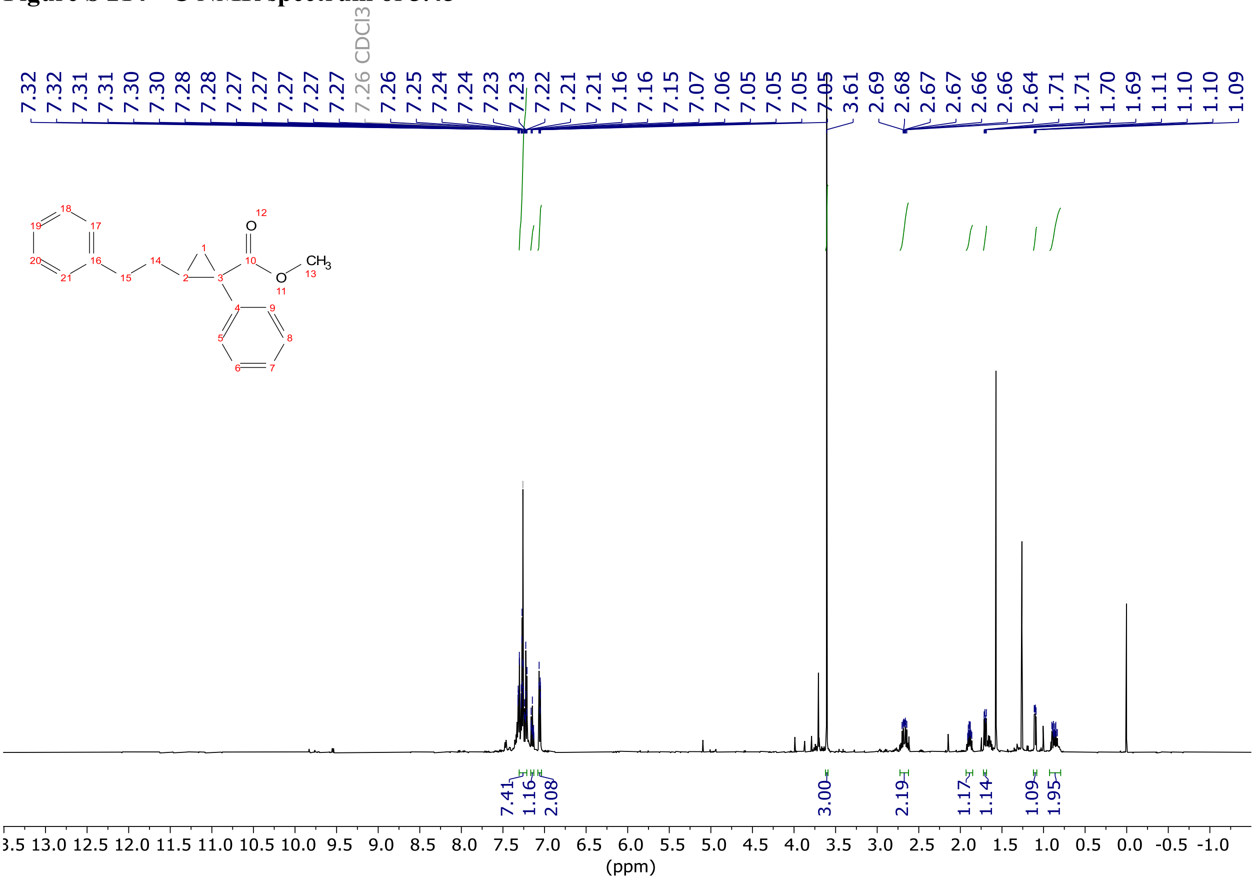

Figure S 215 ^{1}H NMR spectrum of 3.46

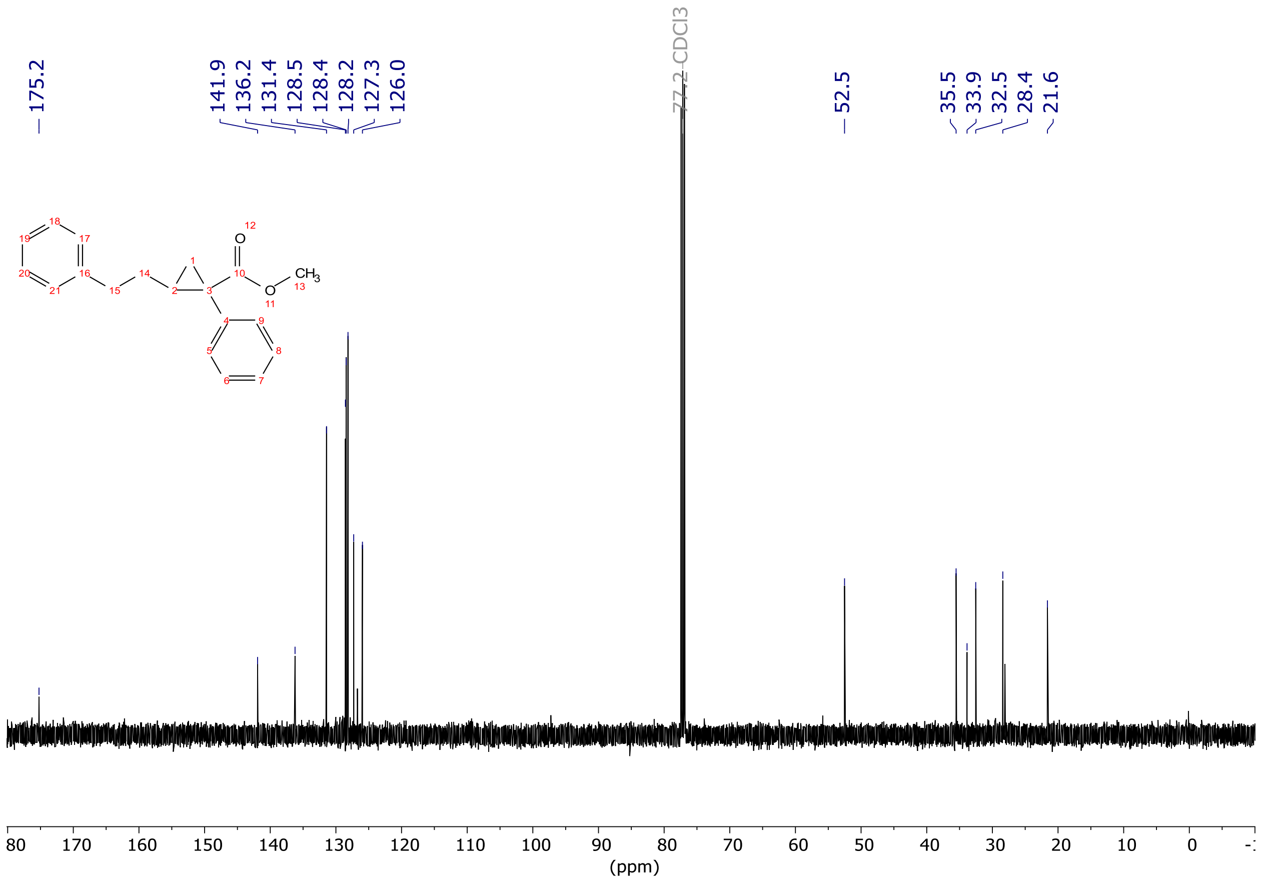

Figure S 216 ^{13}C NMR spectrum of 3.46

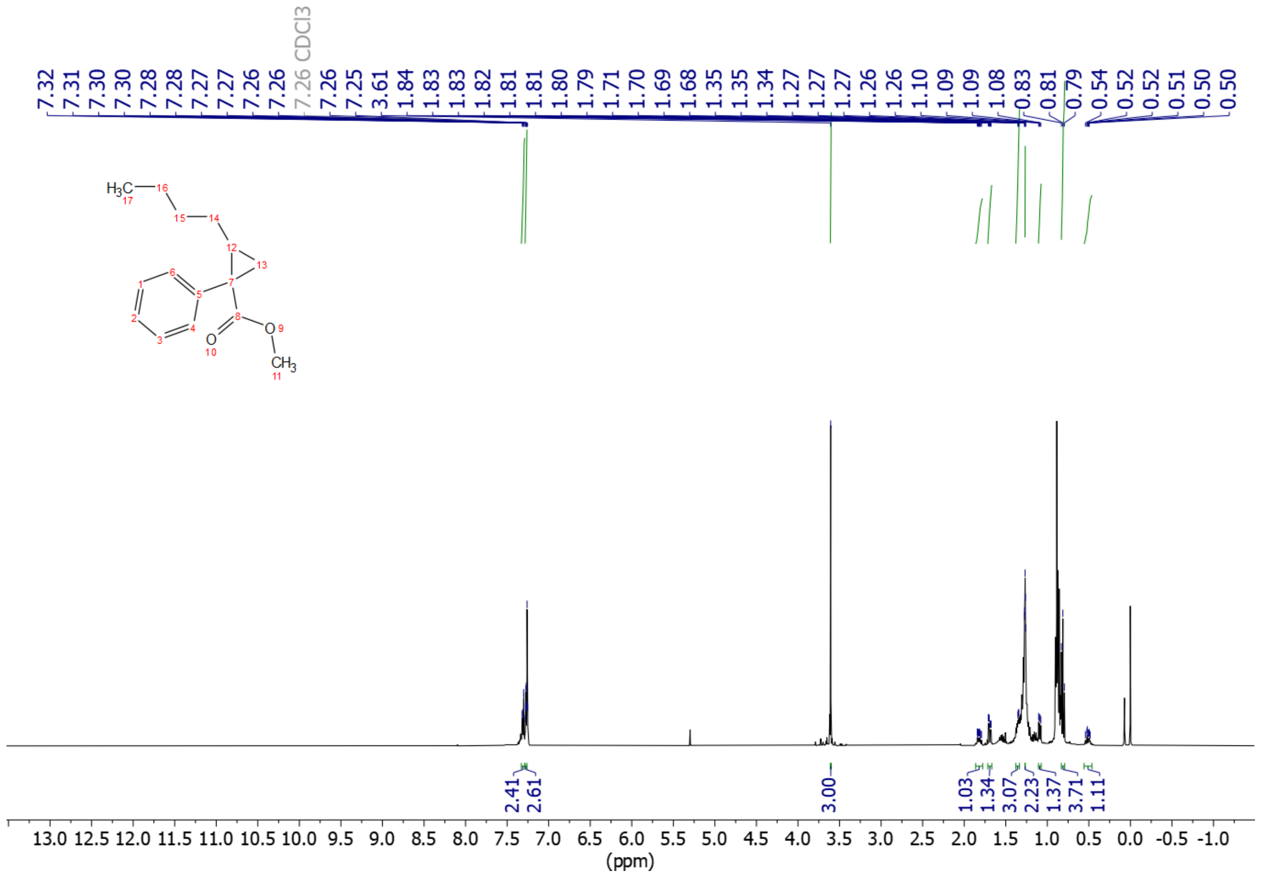

Figure S 217 ^{1}H NMR spectrum of 3.47

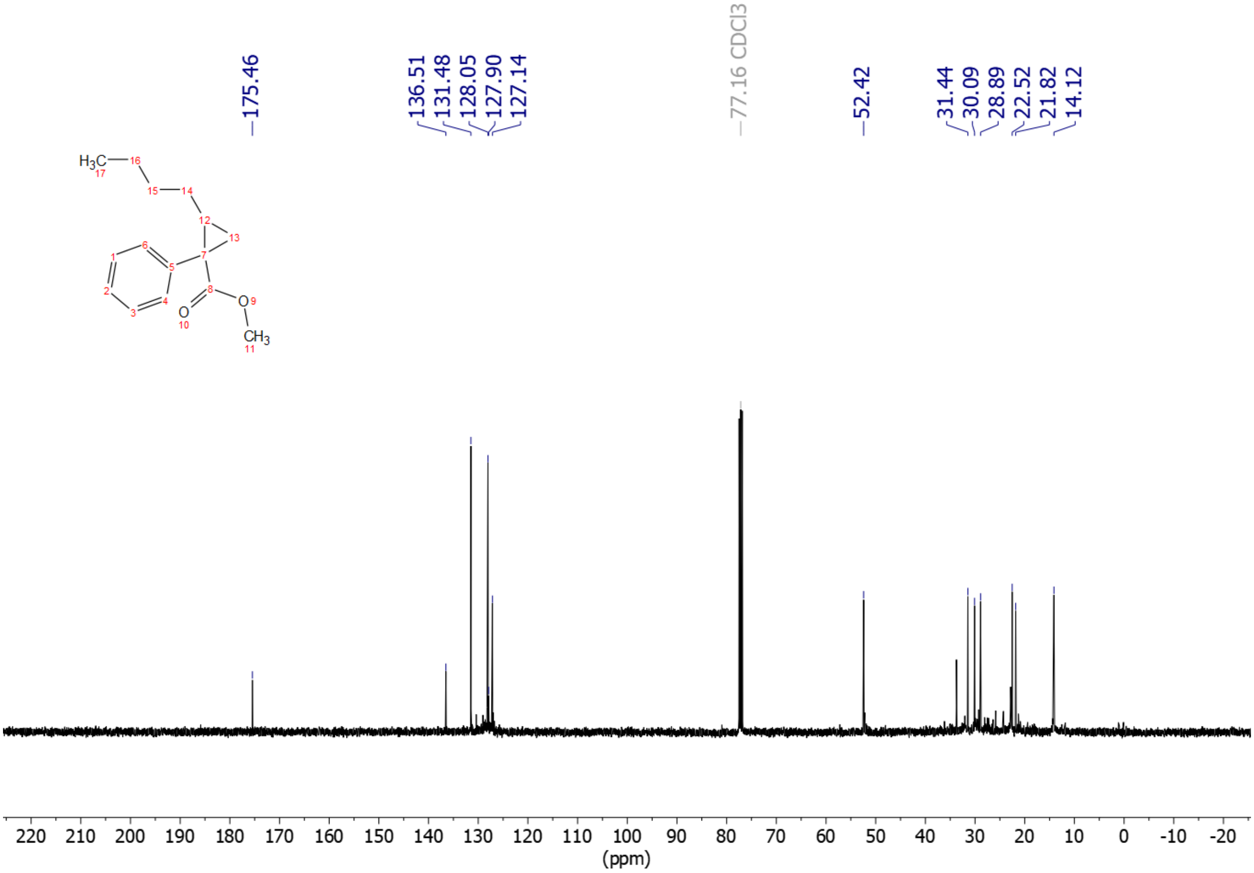

Figure S 218 ^{13}C NMR spectrum of 3.47

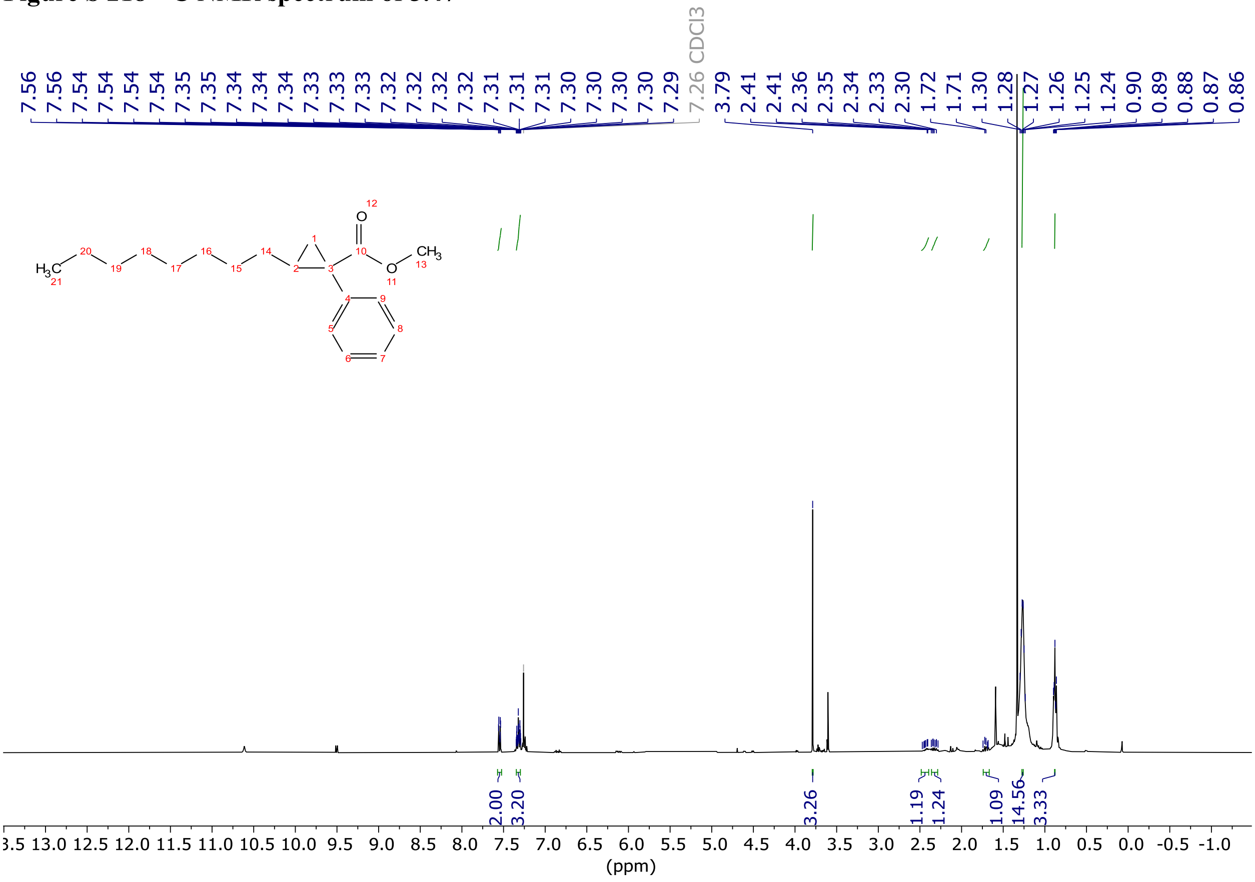

Figure S 219 ^{1}H NMR spectrum of 3.48

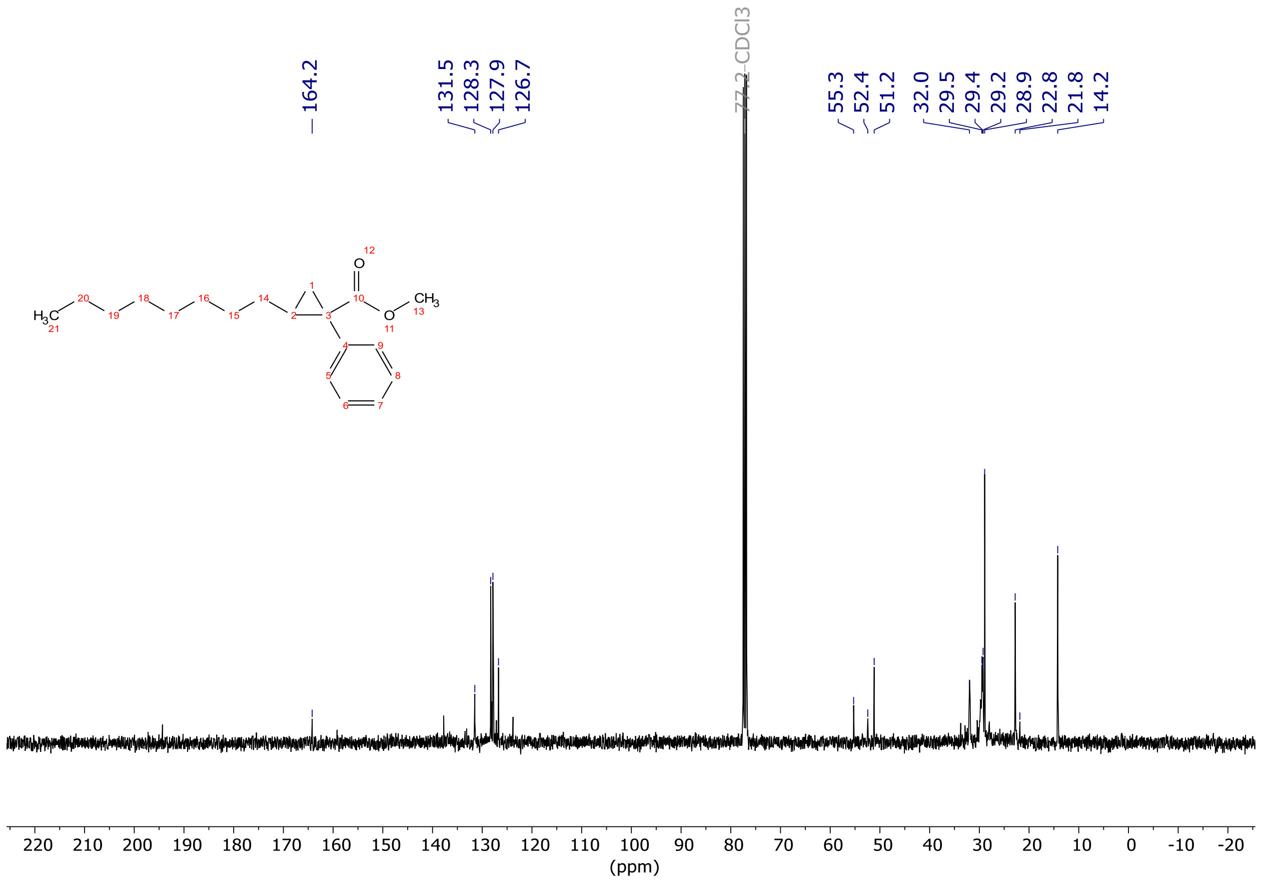

Figure S 220 ^{13}C NMR spectrum of 3.48

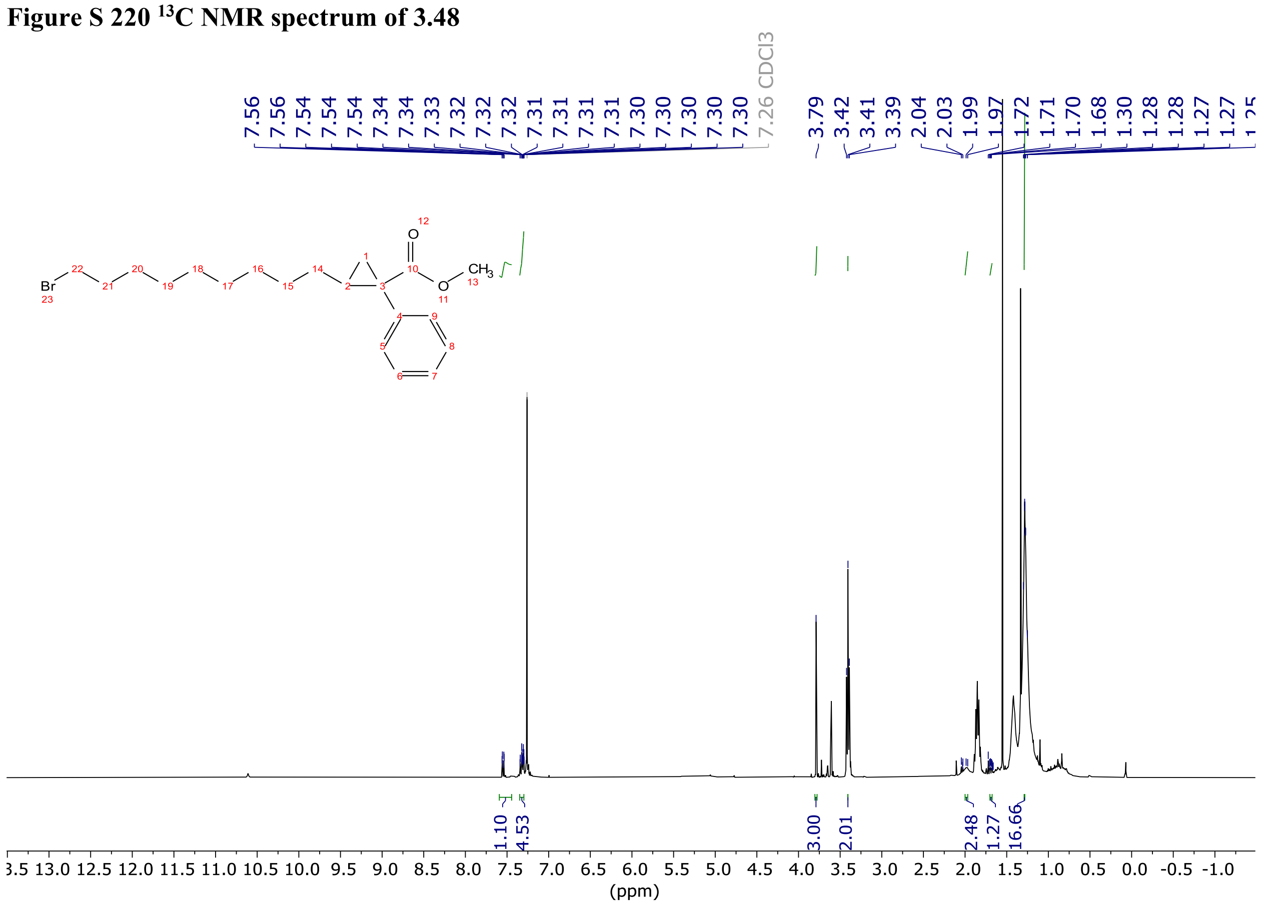

Figure S 221 ^{1}H NMR spectrum of 3.49

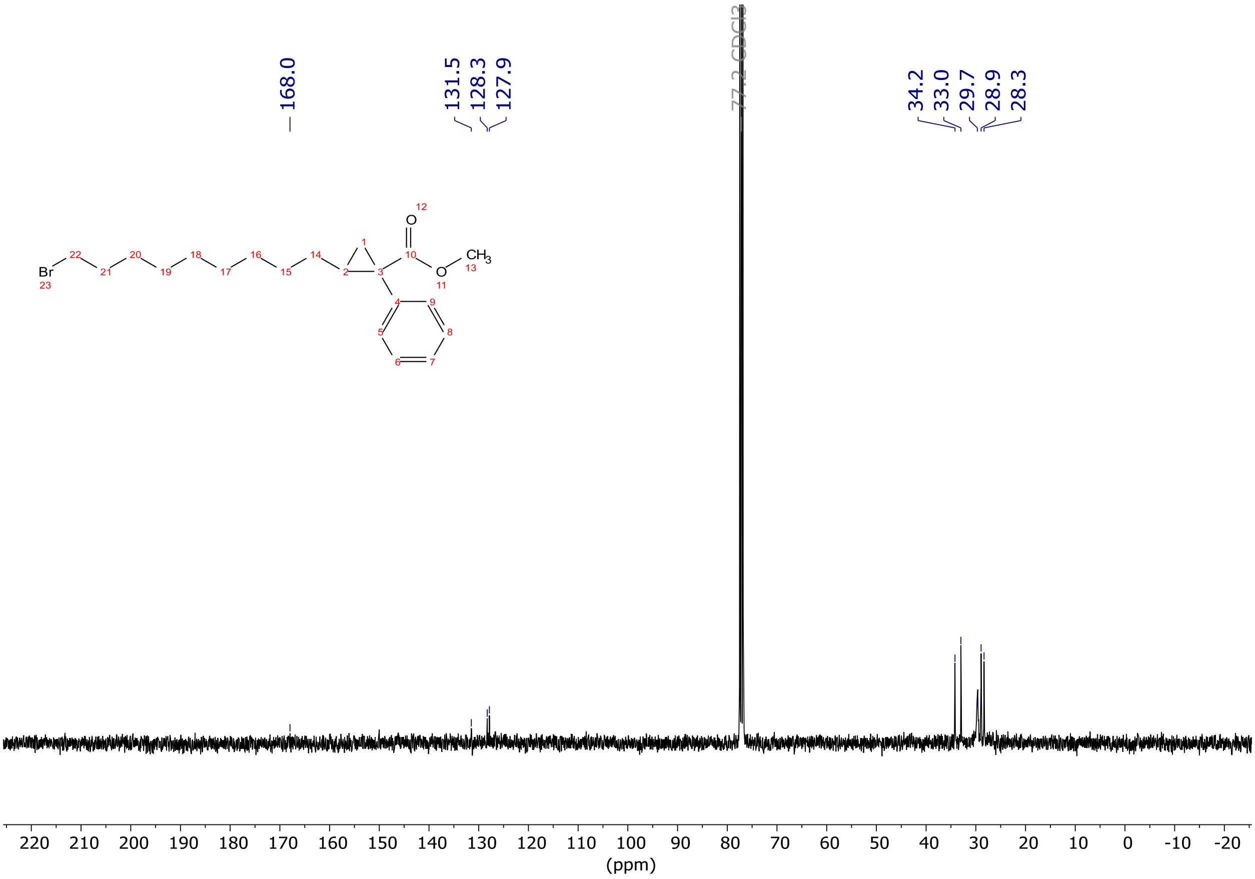

Figure S 222 ^{13}C NMR spectrum of 3.49

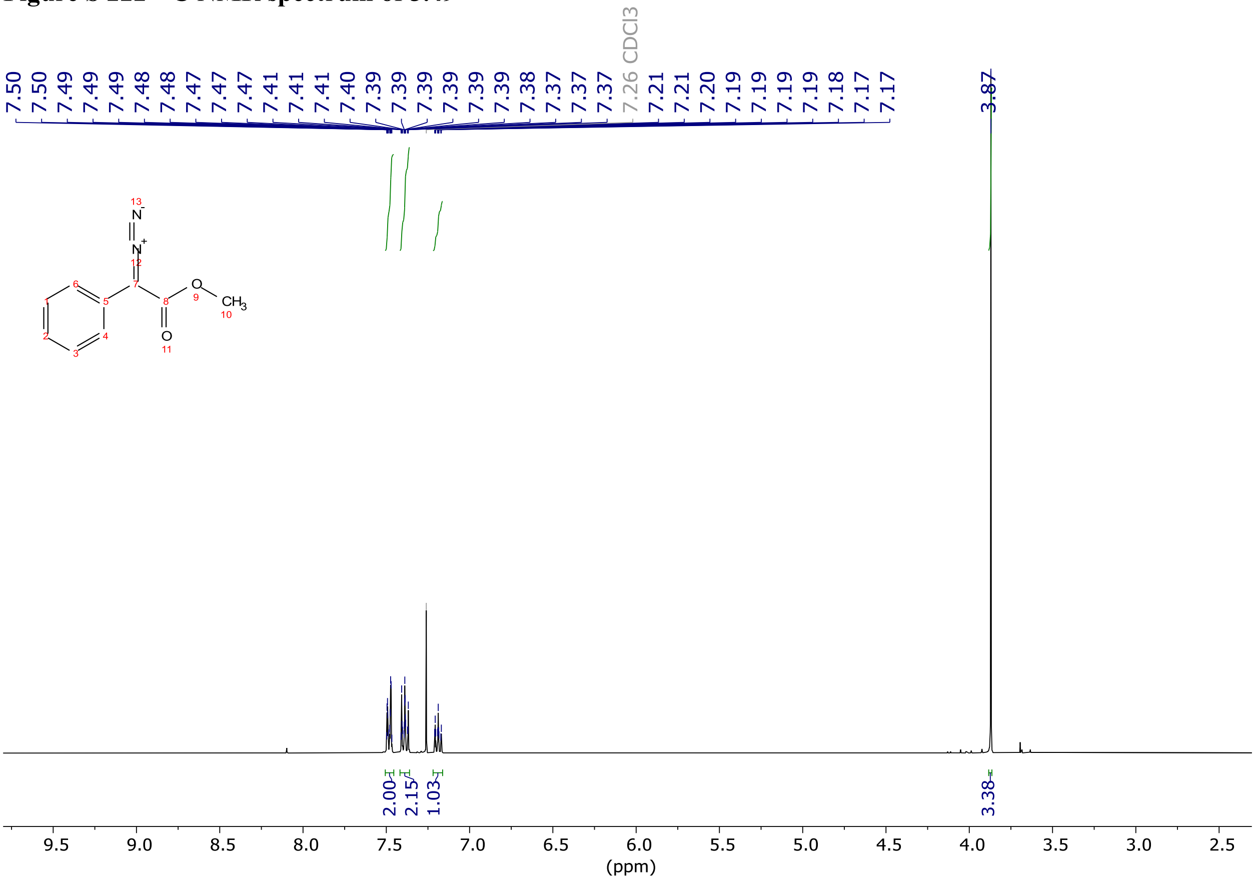

Figure S 223 ^{1}H NMR spectrum of 4.1

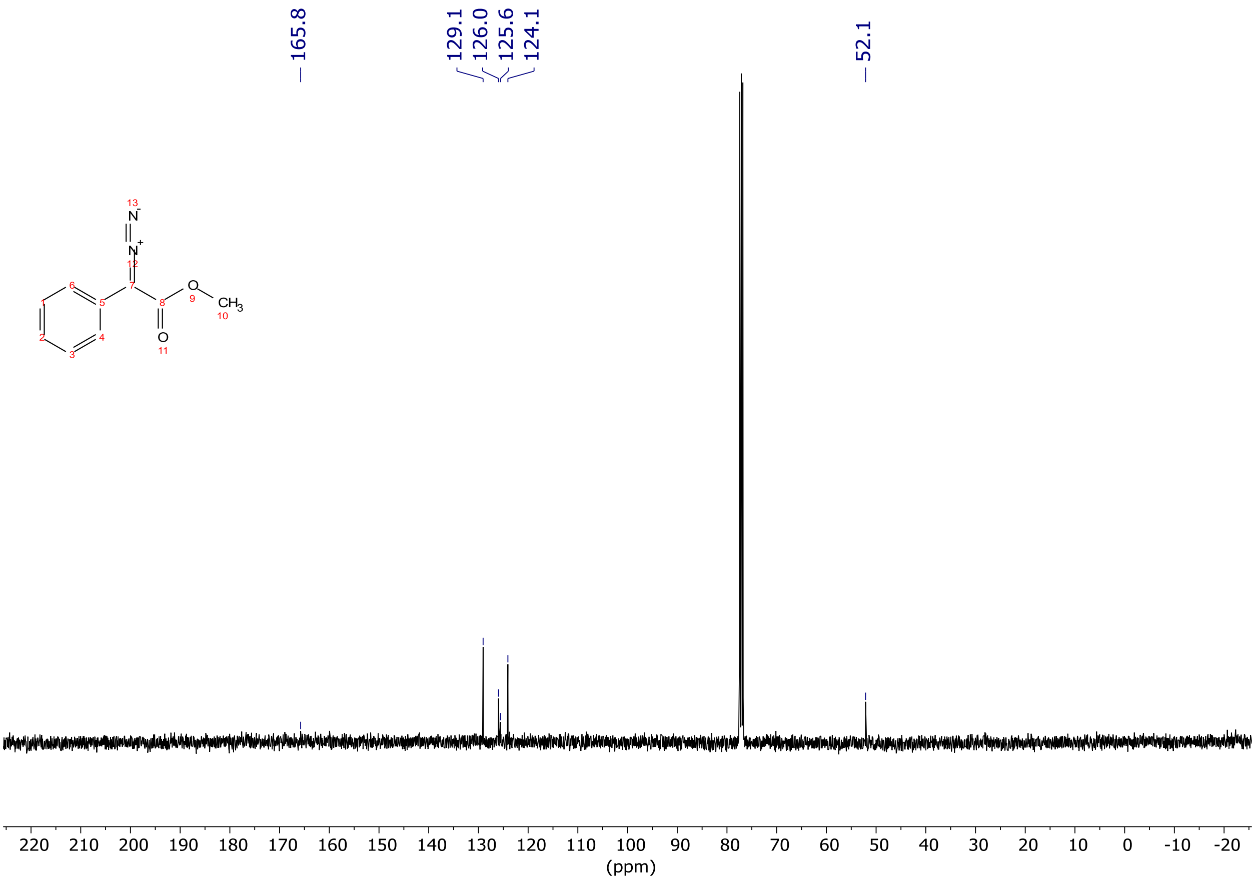

Figure S 224 ^{13}C NMR spectrum of 4.1

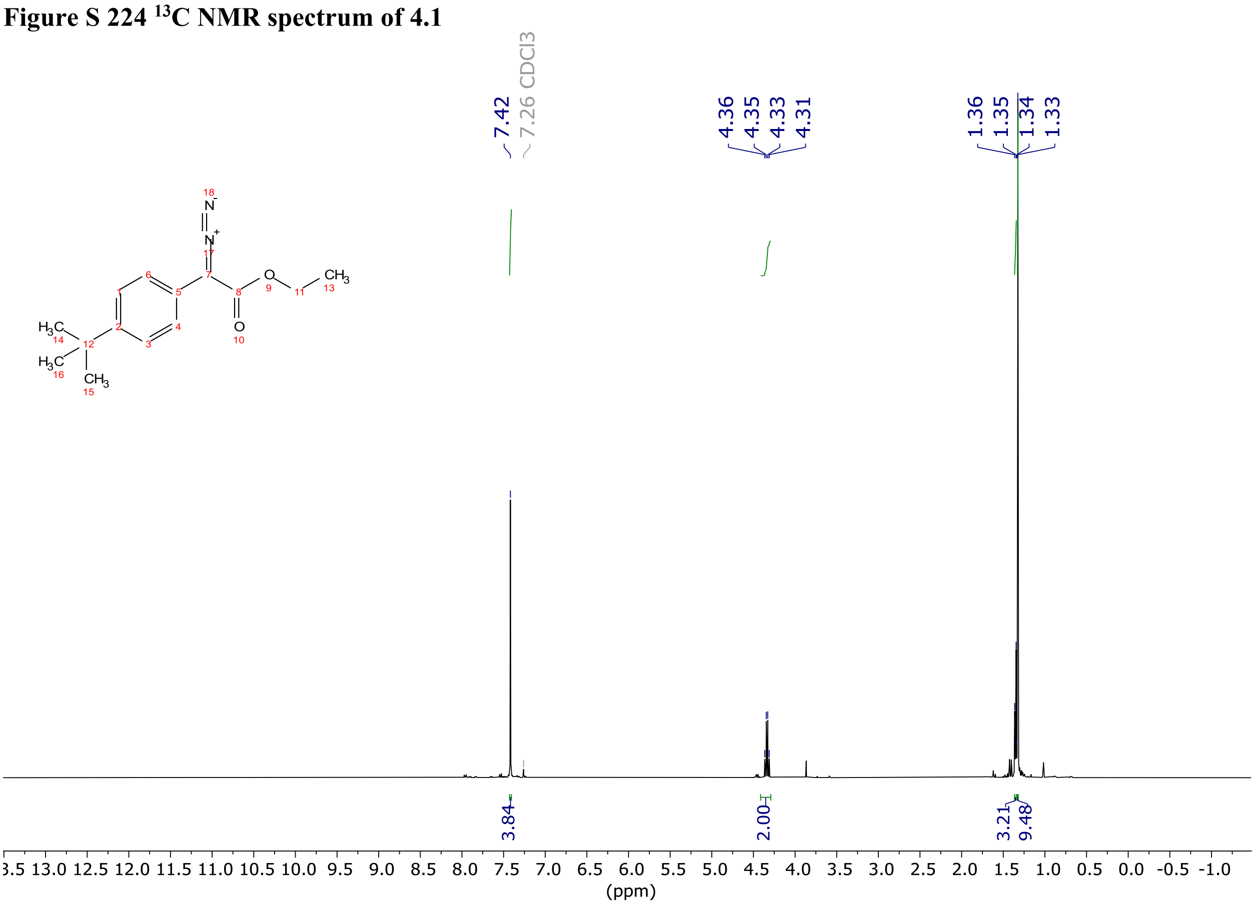

Figure S 225 ^{1}H NMR spectrum of 4.2

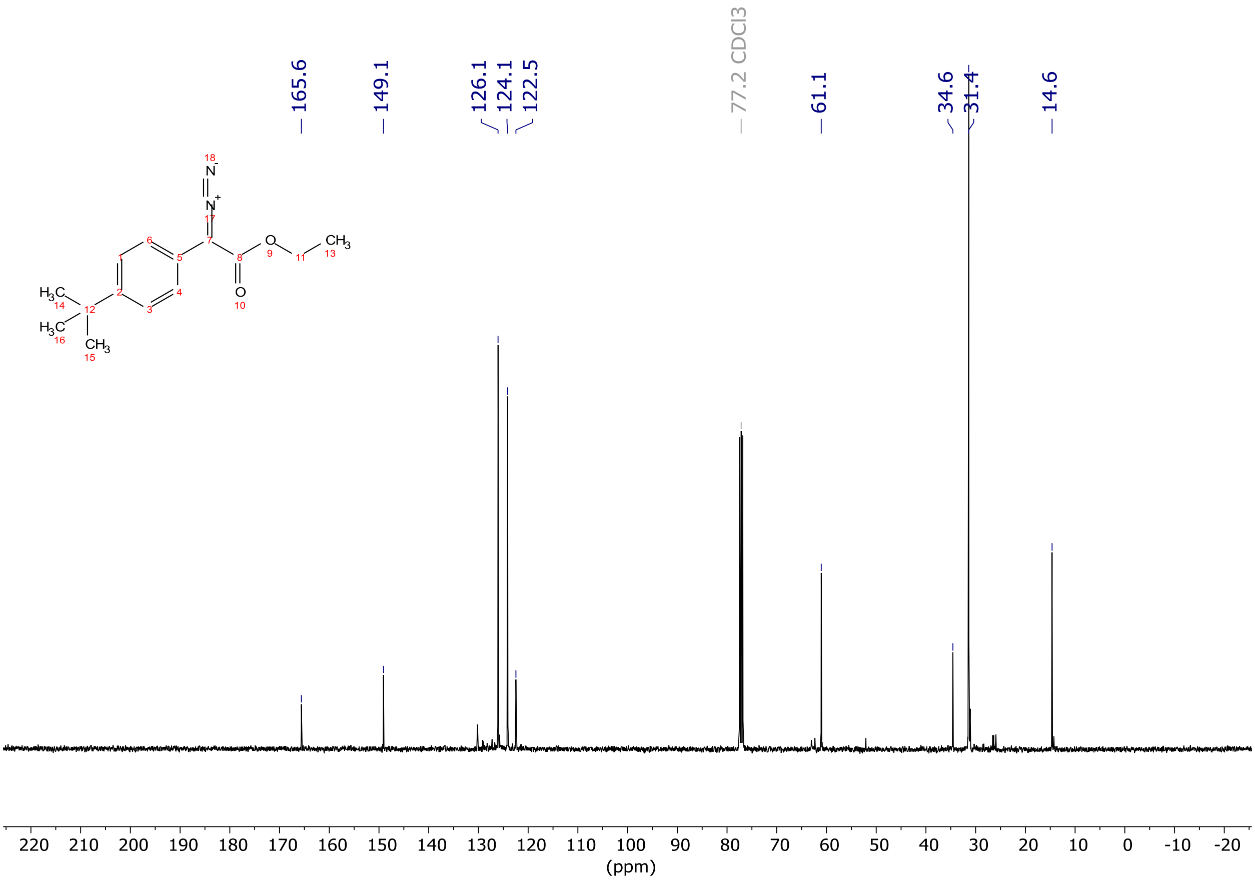

Figure S 226 ^{13}C NMR spectrum of 4.2

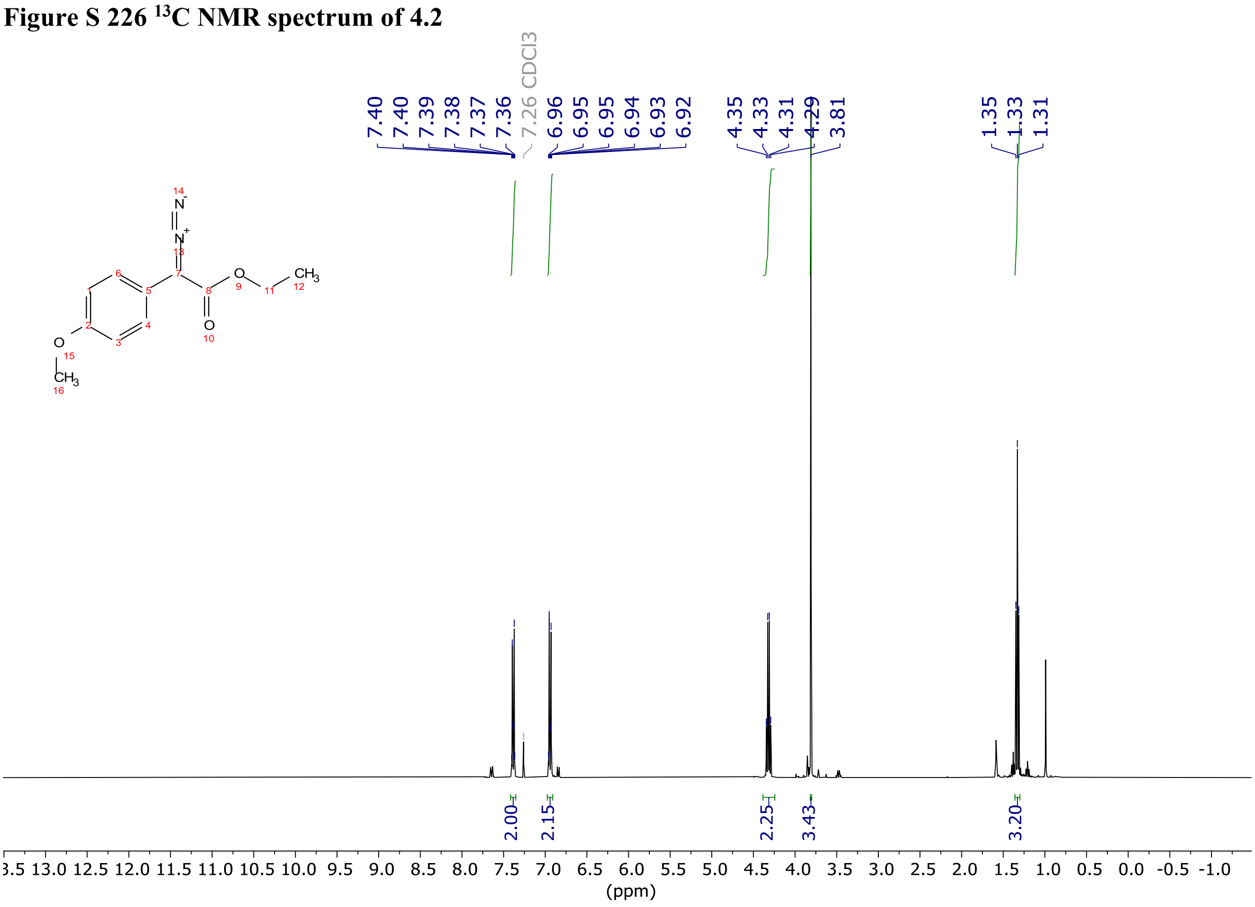

Figure S 227 ^{1}H NMR spectrum of 4.3

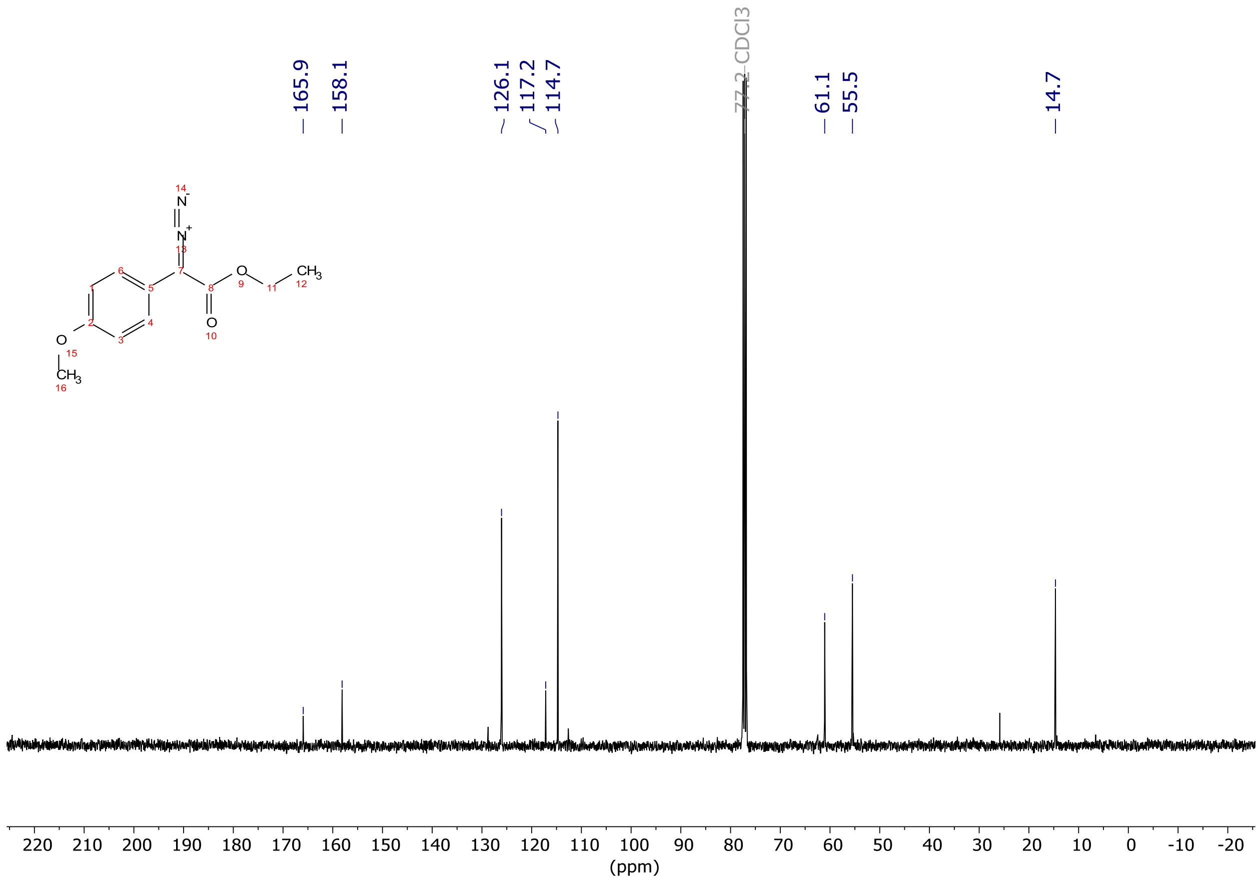

Figure S 228 ^{13}C NMR spectrum of 4.3

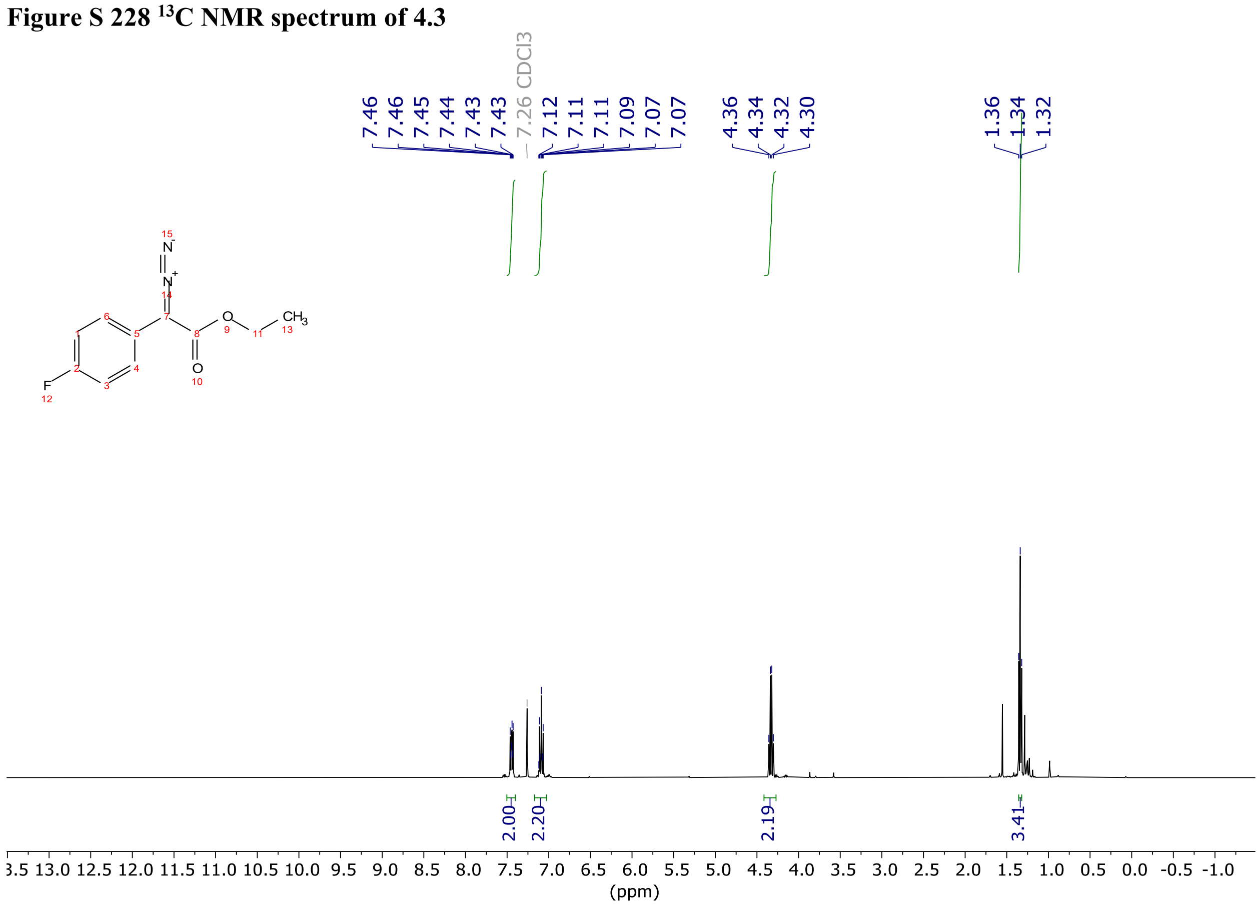

Figure S 229 ^{1}H NMR spectrum of 4.4

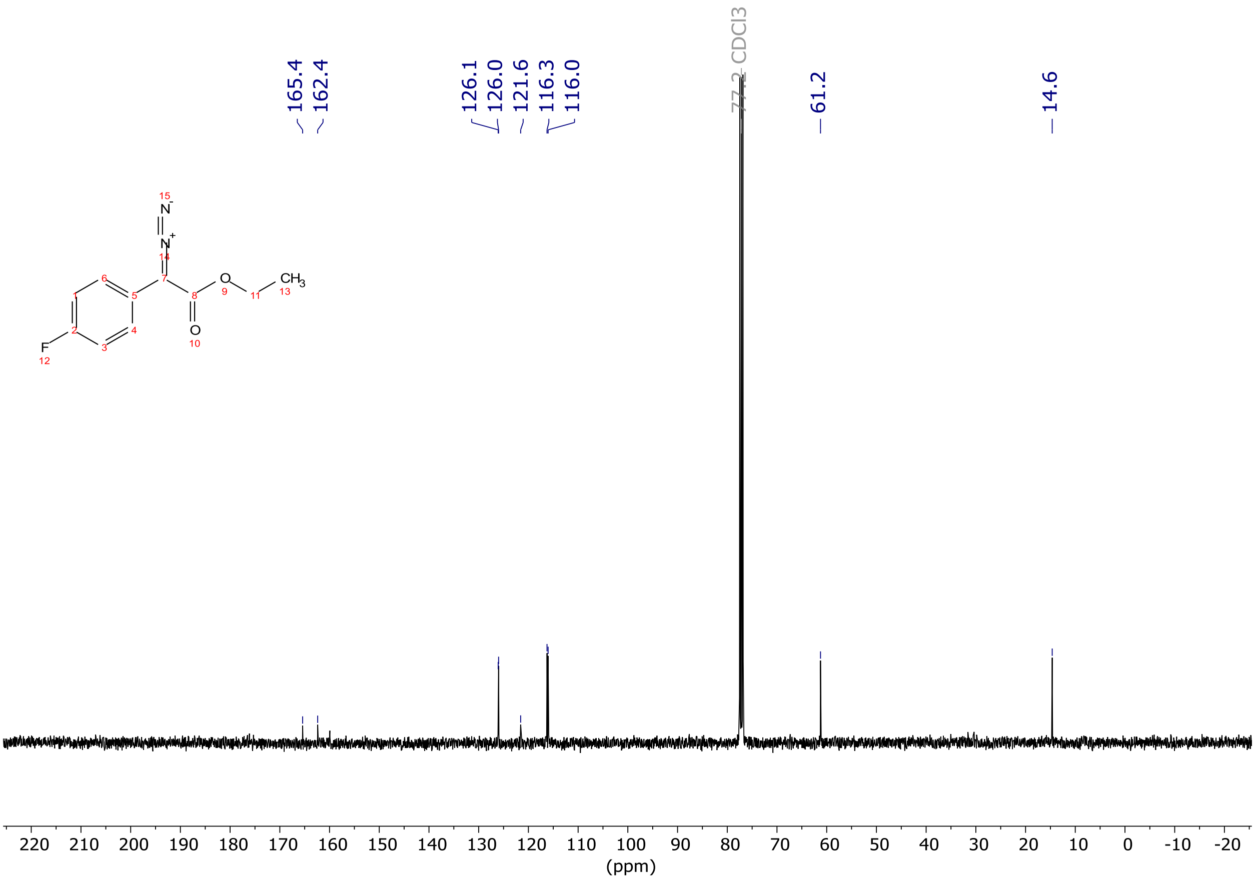

Figure S 230 ^{13}C NMR spectrum of 4.4

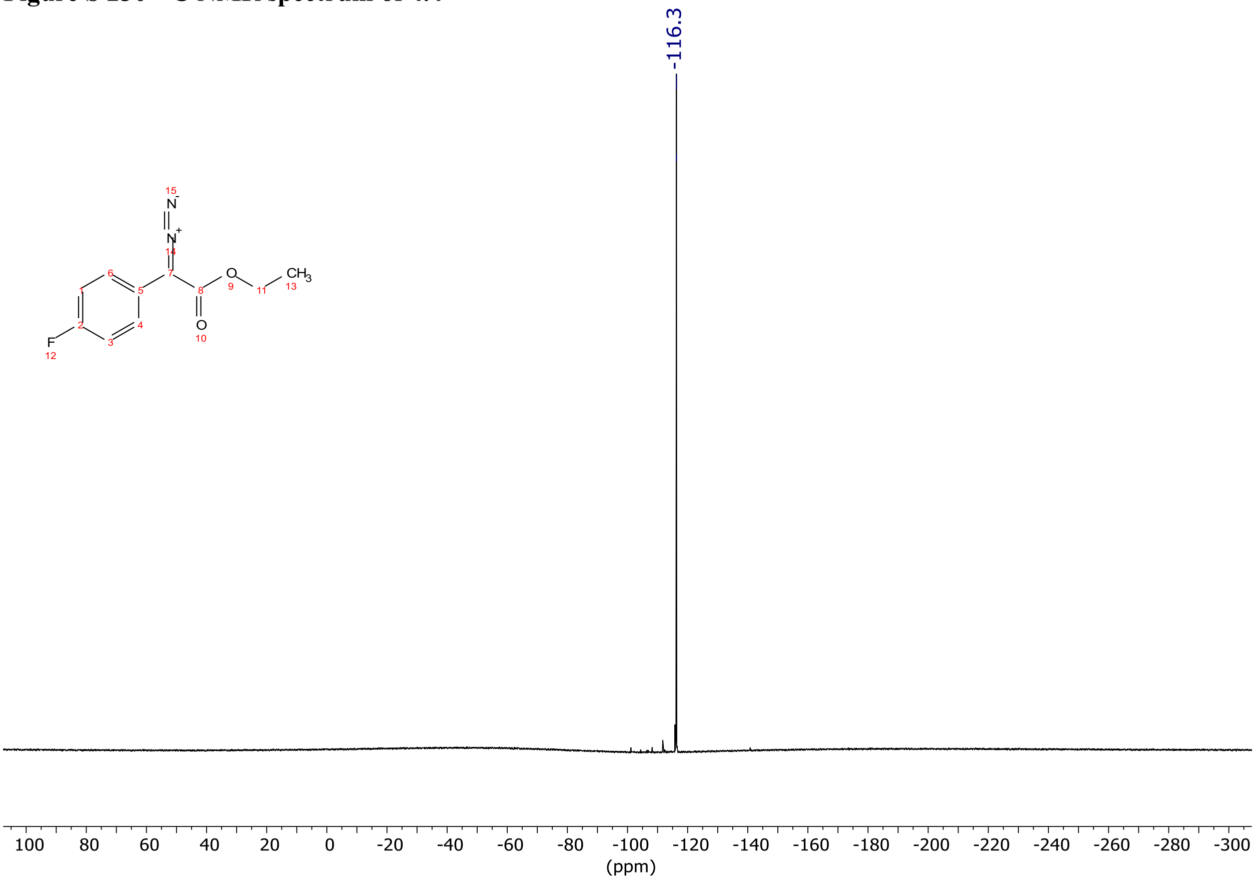

Figure S 231 ^{19}F NMR spectrum of 4.4

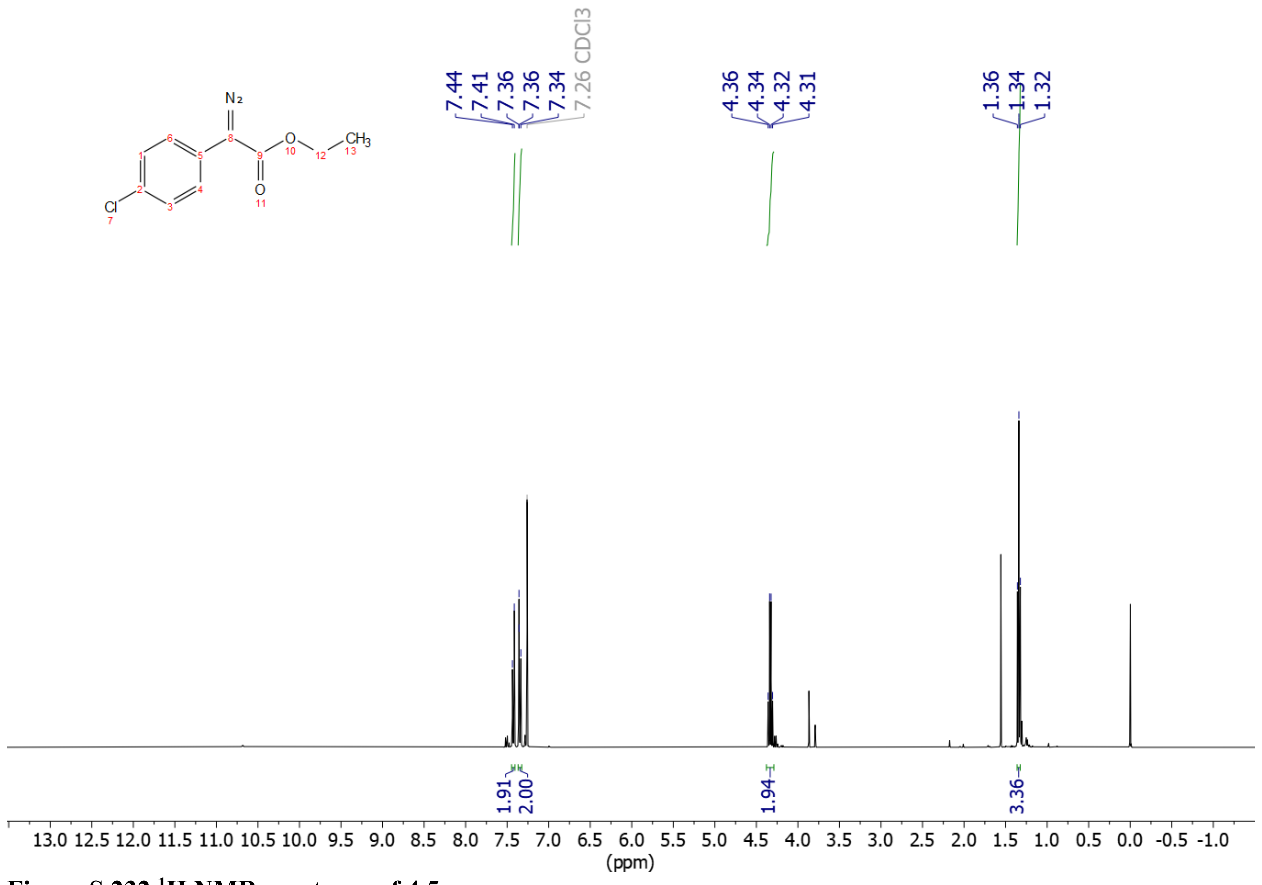

Figure S 232 ^{1}H NMR spectrum of 4.5

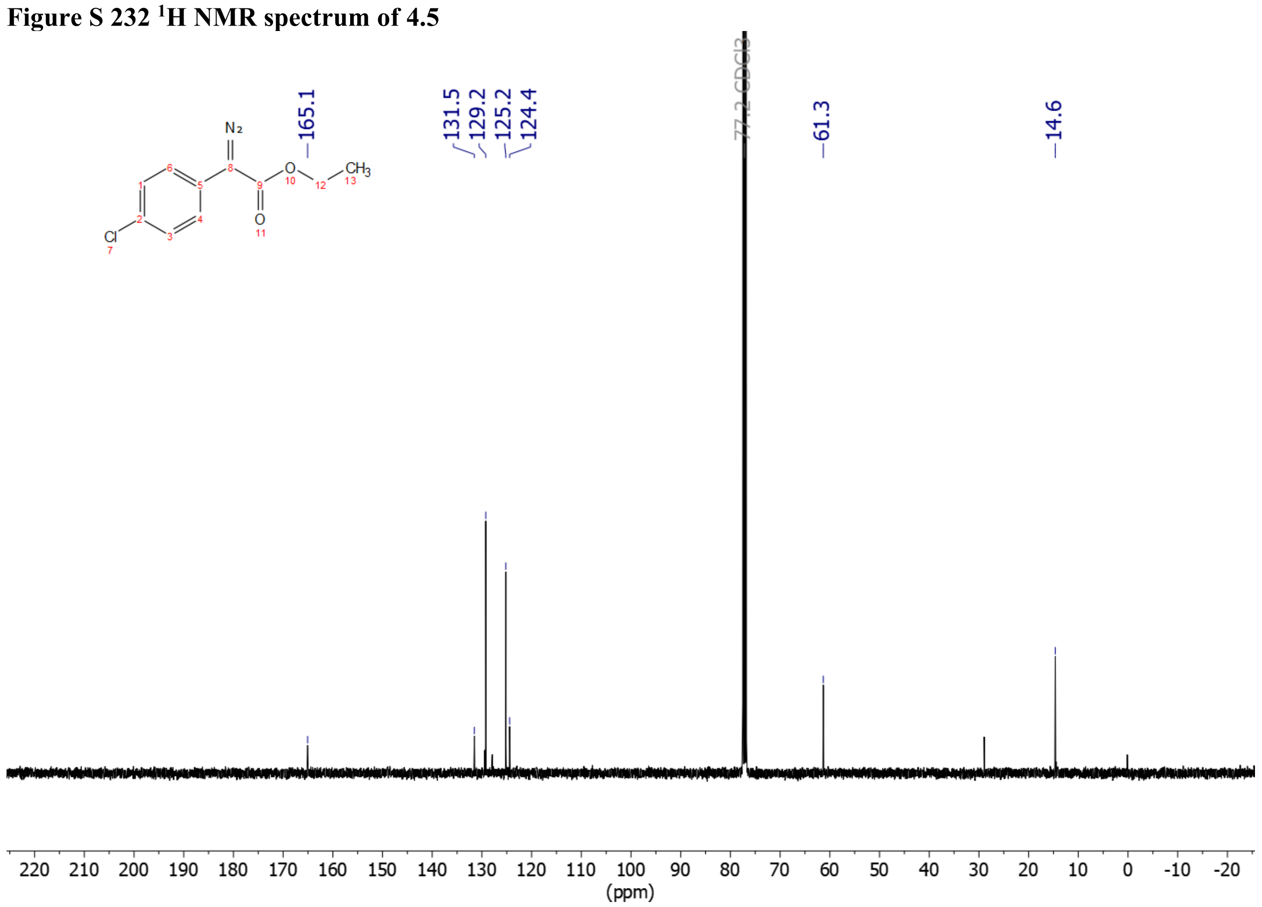

Figure S 233 ^{13}C NMR spectrum of 4.5

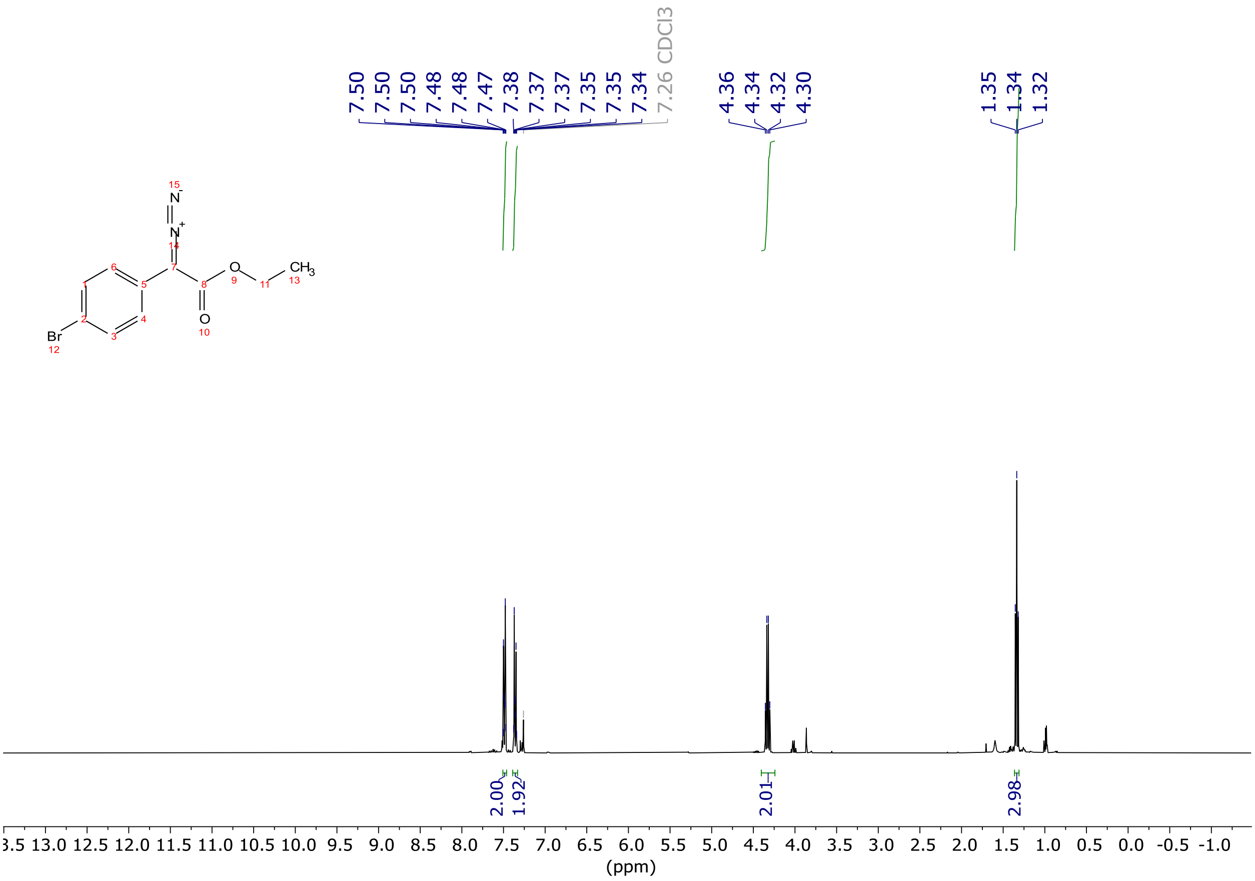

Figure S 234 ^{1}H NMR spectrum of 4.6

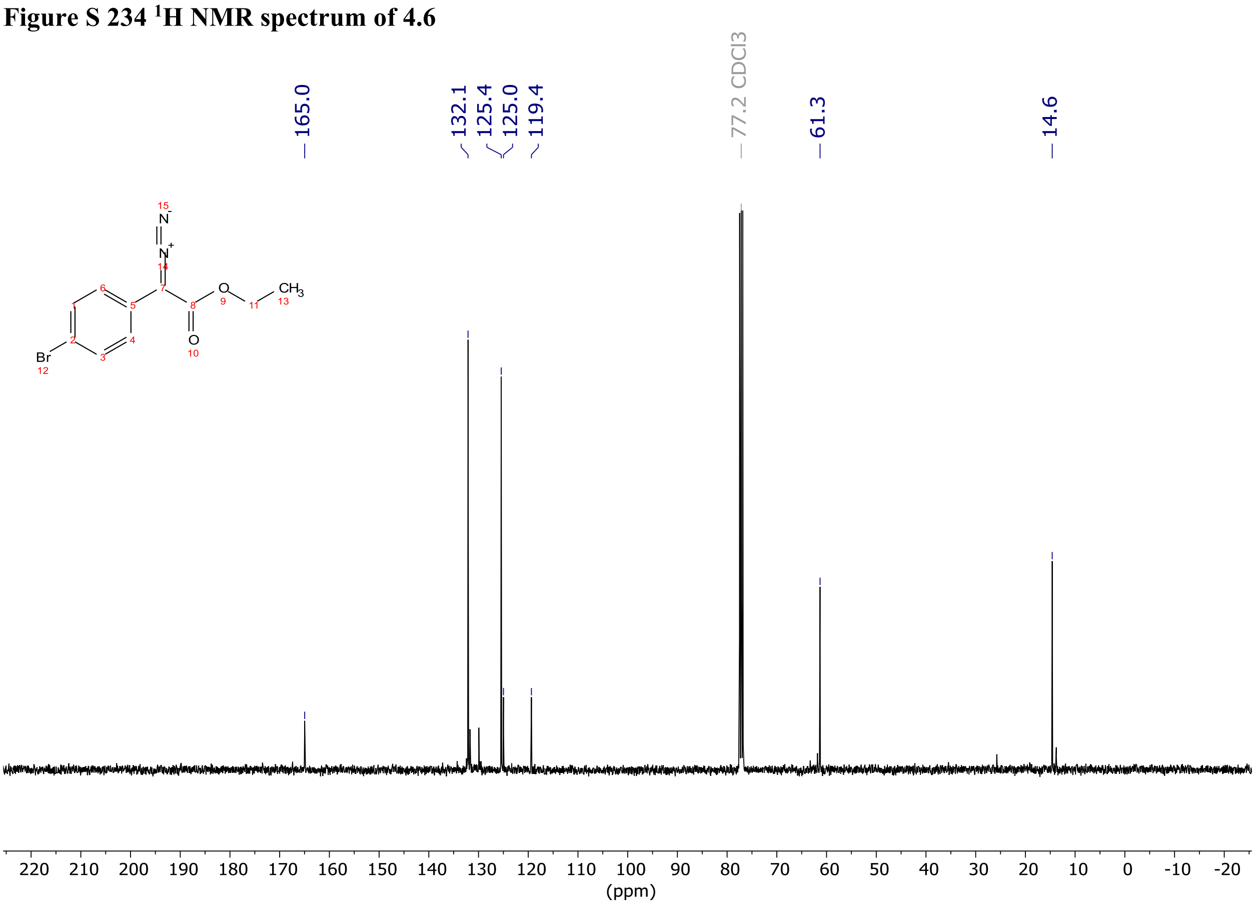

Figure S 235 ^{13}C NMR spectrum of 4.6

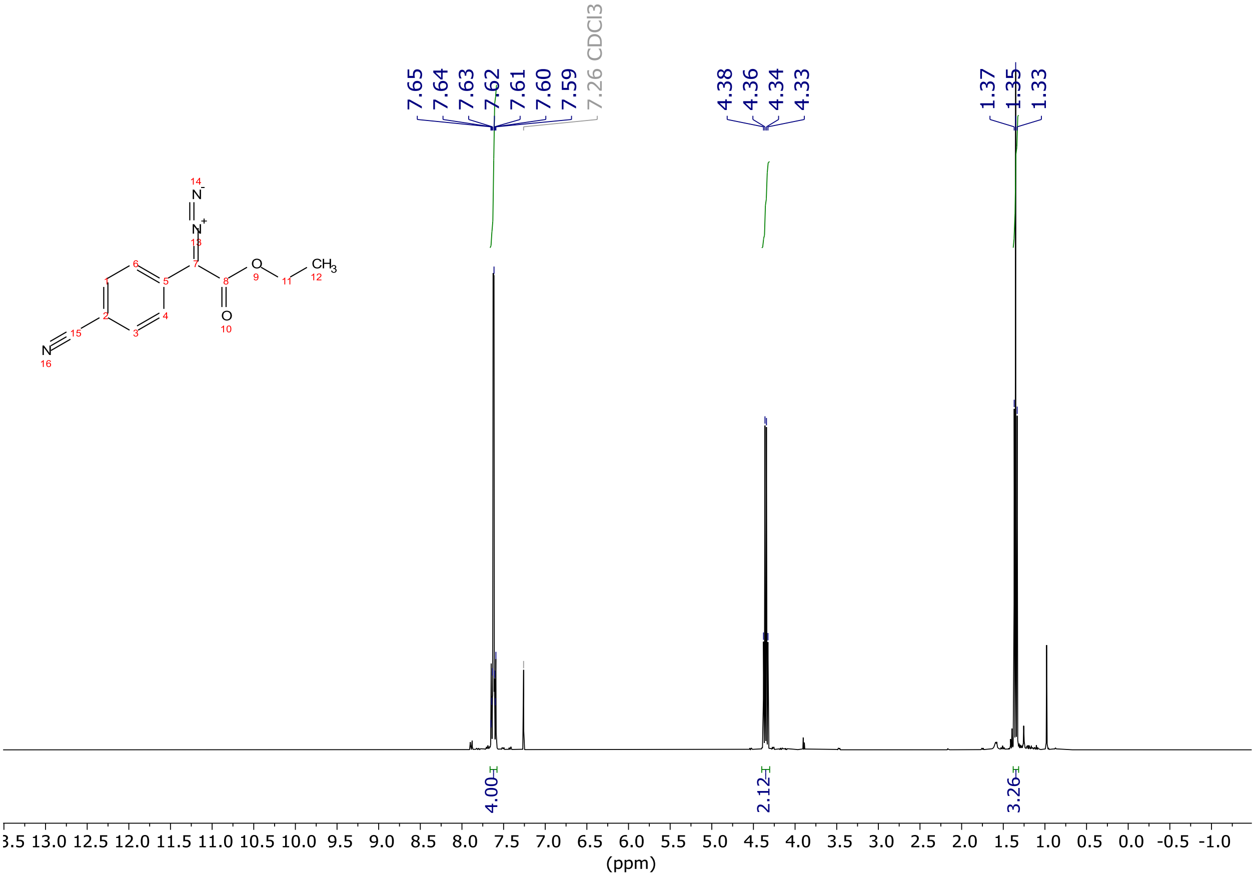

Figure S 236 ^{1}H NMR spectrum of 4.7

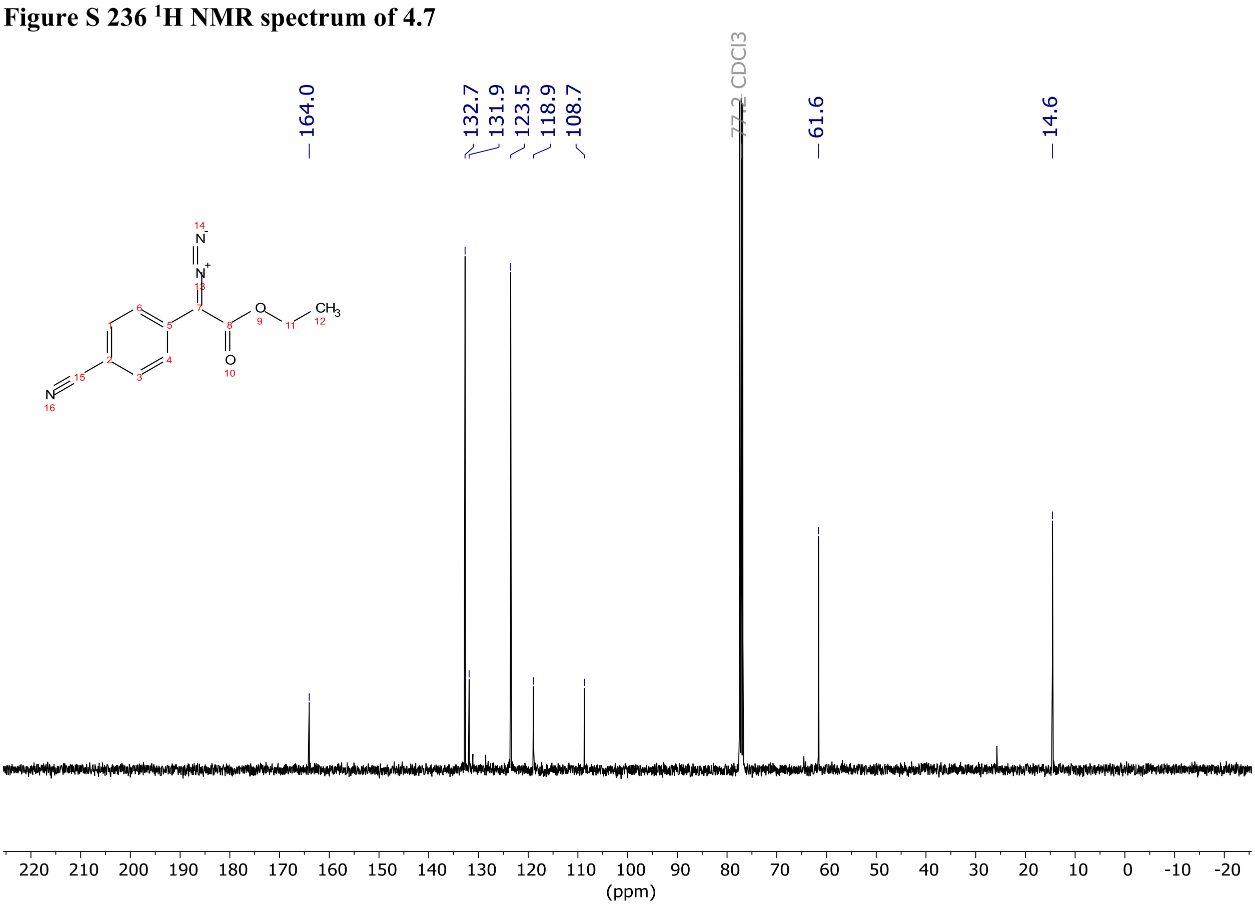

Figure S 237 ^{13}C NMR spectrum of 4.7

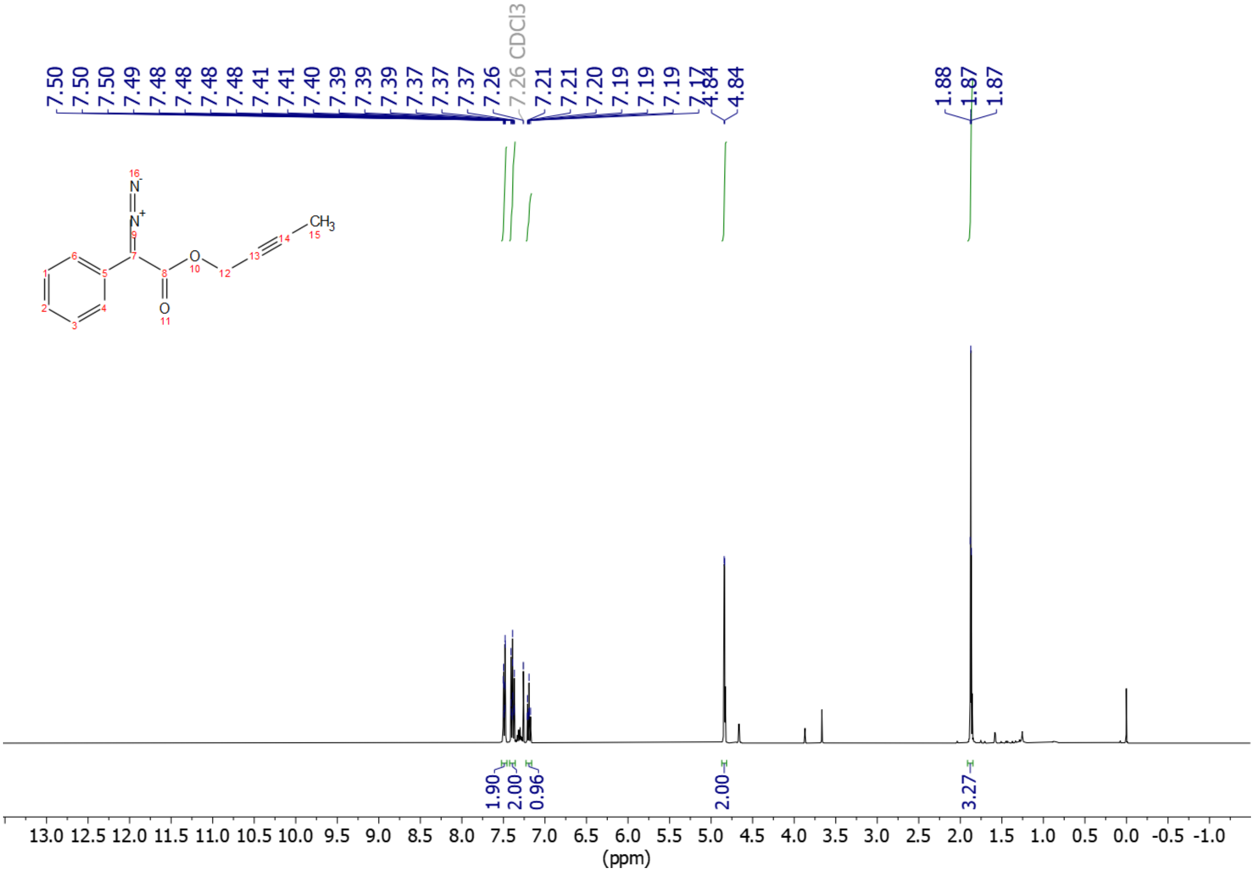

Figure S 238 ^{1}H NMR spectrum of 4.8

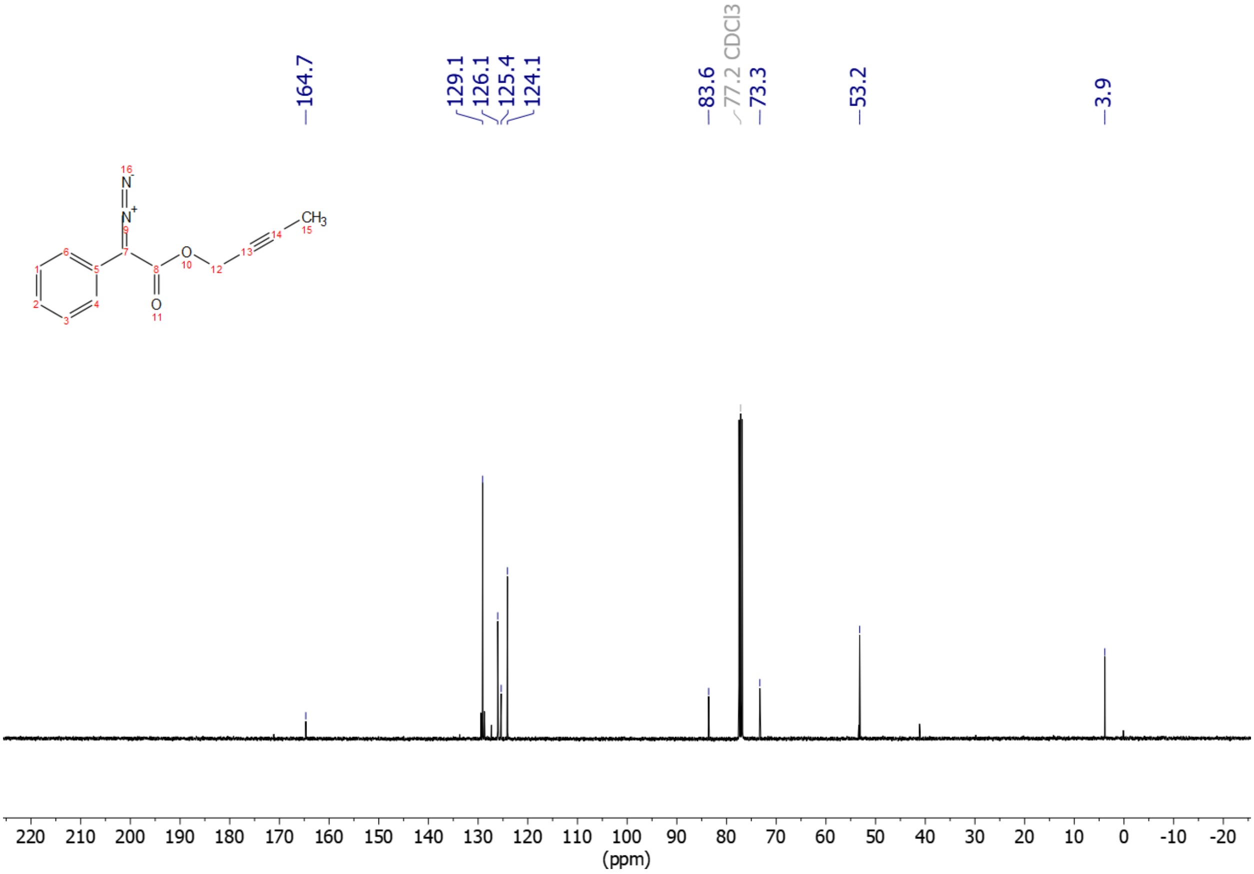

Figure S 239 ^{13}C NMR spectrum of 4.8

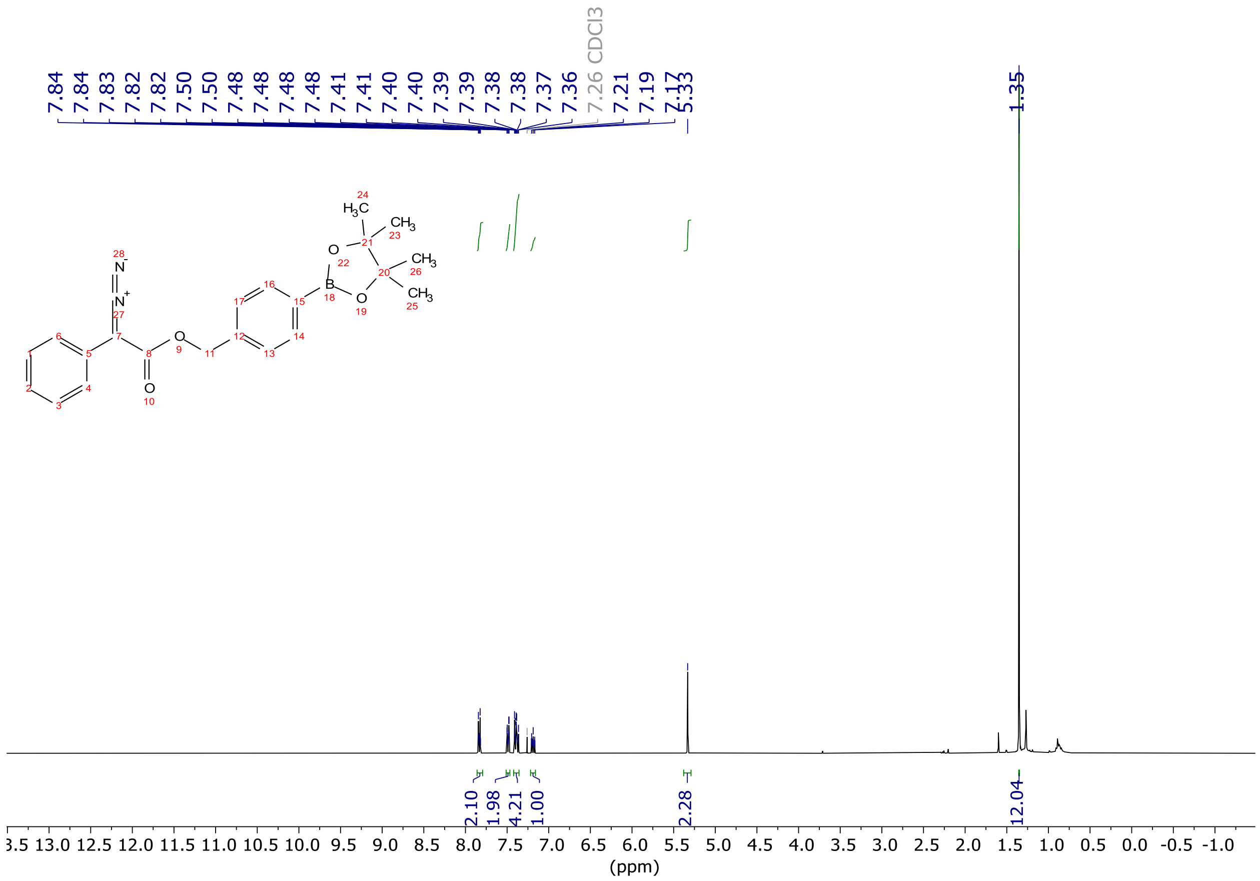

Figure S 240 ^{1}H NMR spectrum of 4.9

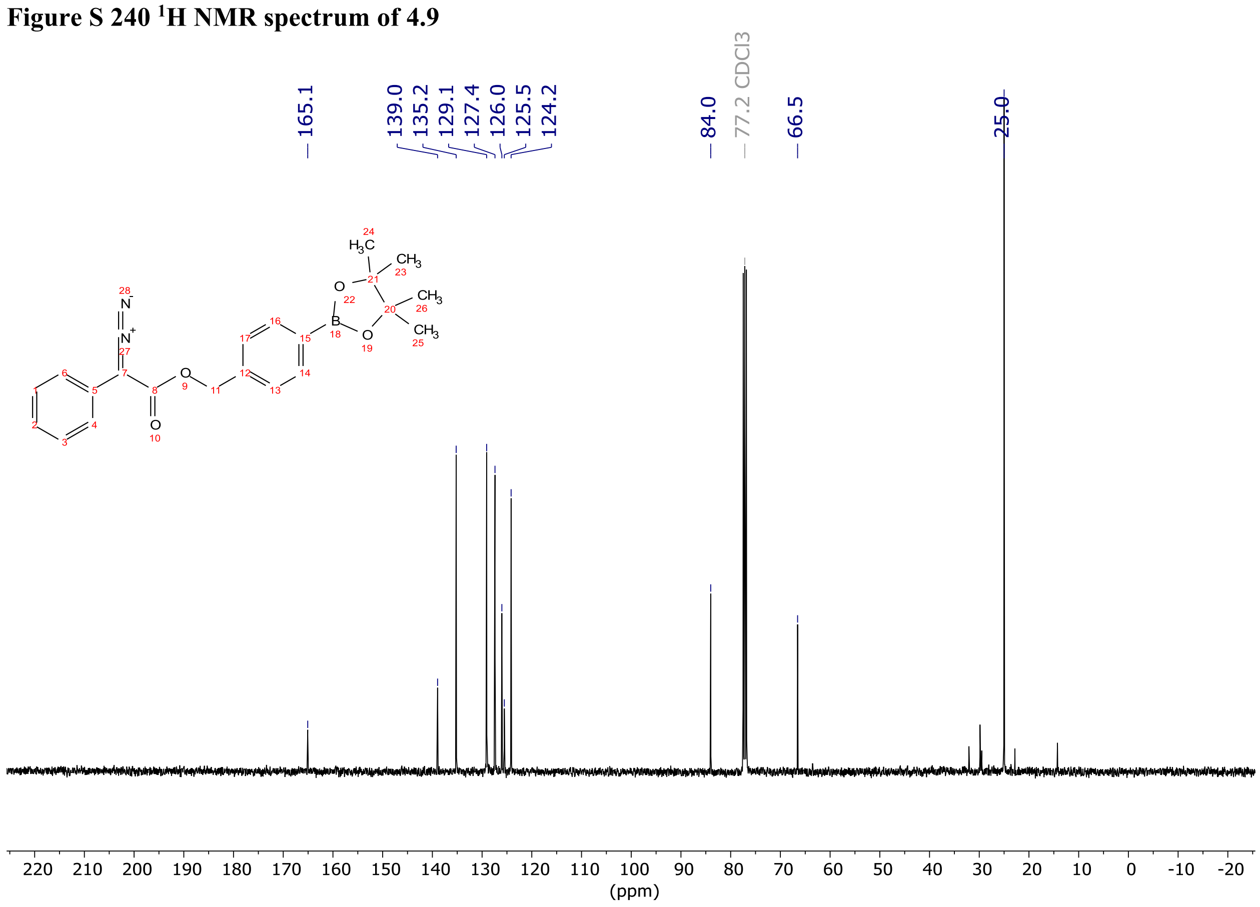

Figure S 241 ^{13}C NMR spectrum of 4.9

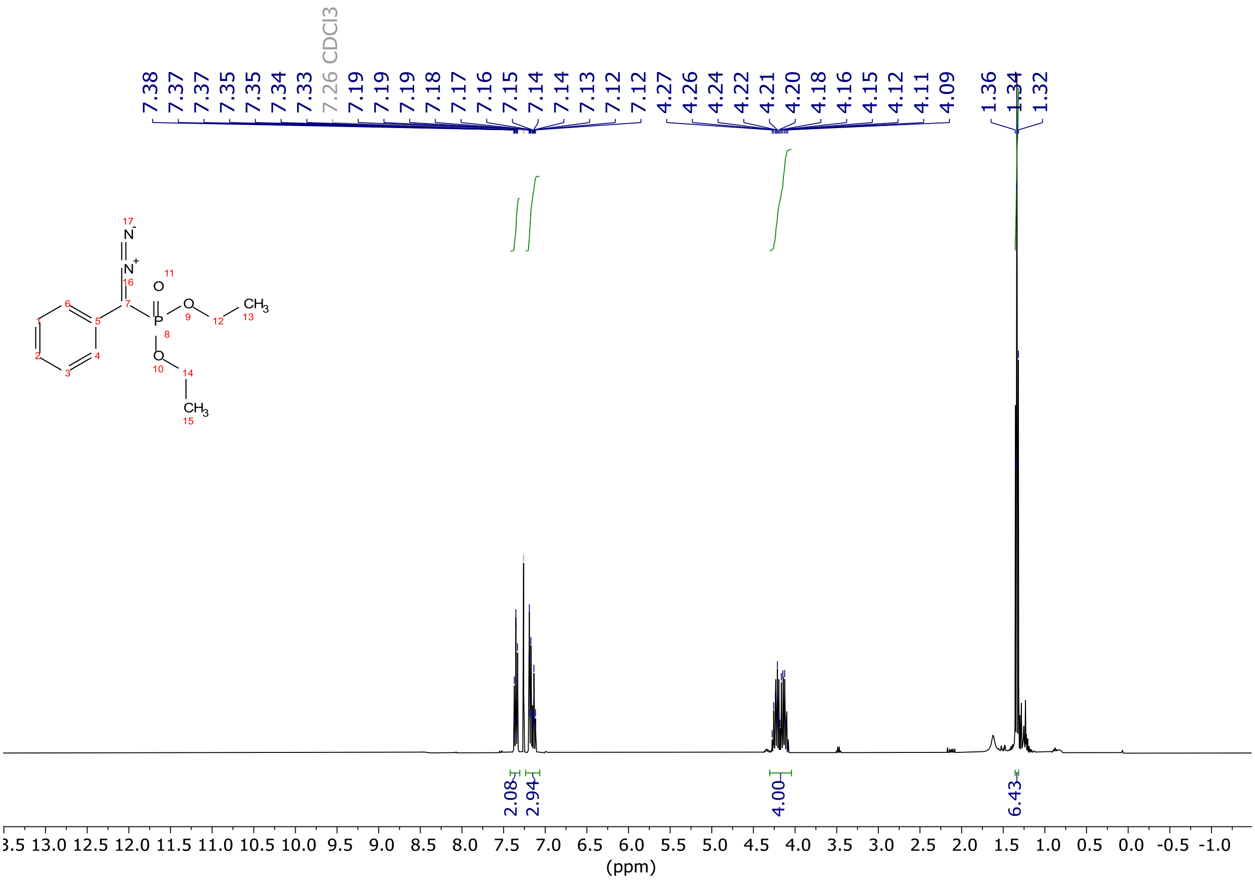

Figure S 242 ^{1}H NMR spectrum of 4.10

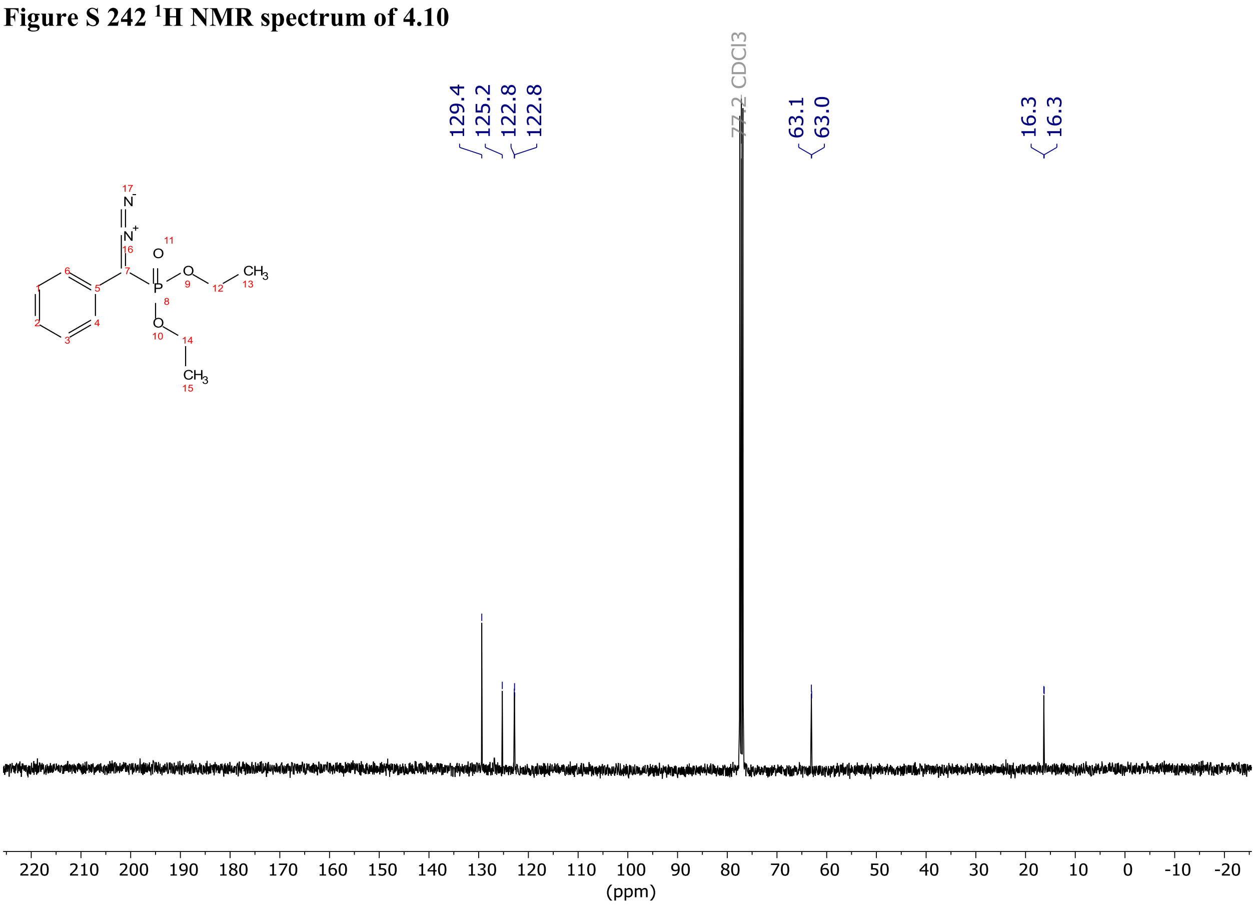

Figure S 243 ^{13}C NMR spectrum of 4.10

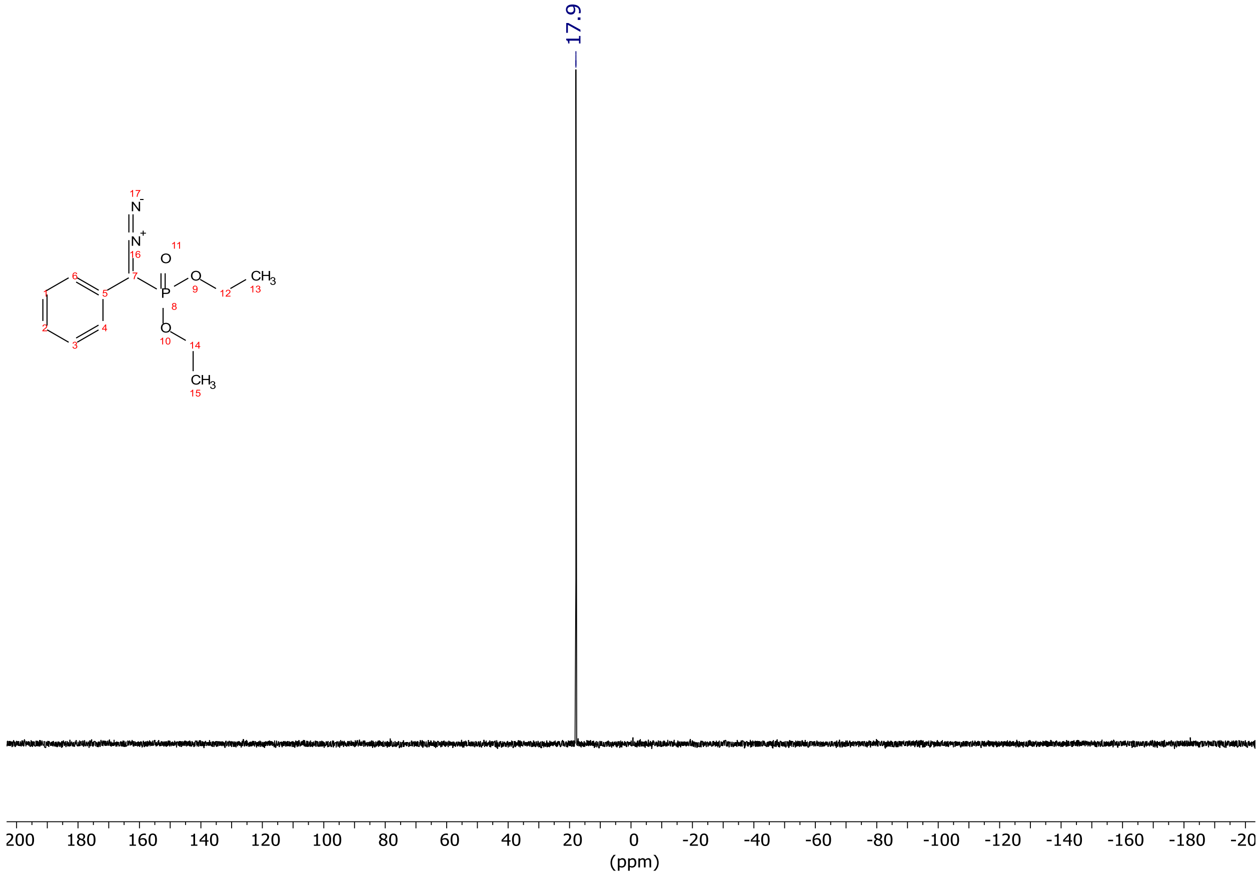

Figure S 244 3^{1}P NMR spectrum of 4.10
